# Incarceration of Youths in an Adult Correctional Facility and Risk of Premature Death

**DOI:** 10.1001/jamanetworkopen.2023.21805

**Published:** 2023-07-05

**Authors:** Ian A. Silver, Daniel C. Semenza, Joseph L. Nedelec

**Affiliations:** 1 Center for Legal Systems Research, RTI International, Research Triangle Park, North Carolina; 2 Department of Sociology, Anthropology, and Criminal Justice, Rutgers University, Camden, New Jersey; 3 New Jersey Gun Violence Research Center, Department of Urban-Global Health, School of Public Health, Rutgers University, New Brunswick, New Jersey; 4 School of Criminal Justice, University of Cincinnati, Cincinnati, Ohio

## Abstract

**Importance:**

Youths incarcerated in adult correctional facilities are exposed to a variety of adverse circumstances that could diminish psychological and physical health, potentially leading to early mortality.

**Objective:**

To evaluate whether being incarcerated in an adult correctional facility as a youth was associated with mortality between 18 and 39 years of age.

**Design, Setting, and Participants:**

This cohort study relied on longitudinal data collected from 1997 to 2019 as part of the National Longitudinal Survey of Youth–1997, a nationally representative sample of 8984 individuals born in the United States between January 1, 1980, and December 1, 1984. The data analyzed for the current study were derived from annual interviews between 1997 and 2011 and interviews every other year from 2013 to 2019 (19 interviews in total). Participants were limited to respondents aged 17 years or younger during the 1997 interview and alive during their 18th birthday (8951 individuals; >99% of the original sample). Statistical analysis was performed from November 2022 to May 2023.

**Intervention:**

Incarceration in an adult correctional facility before the age of 18 years compared with being arrested before the age of 18 years or never arrested or incarcerated before the age of 18 years.

**Main Outcomes and Measures:**

The main outcome for the study was age at mortality between 18 and 39 years of age.

**Results:**

The sample of 8951 individuals included 4582 male participants (51%), 61 American Indian or Alaska Native participants (1%), 157 Asian participants (2%), 2438 Black participants (27%), 1895 Hispanic participants (21%), 1065 participants of other race (12%), and 5233 White participants (59%). A total of 225 participants (3%) died during the study period, with a mean (SD) age at death of 27.7 (5.9) years. Incarceration in an adult correctional facility before the age of 18 years was associated with an increased risk of earlier mortality between 18 and 39 years of age compared with individuals who were never arrested or incarcerated before the age of 18 years (time ratio, 0.67; 95% CI, 0.47-0.95). Being arrested before the age of 18 years was associated with an increased risk of earlier mortality between 18 and 39 years of age when compared with individuals who were never arrested or incarcerated before the age of 18 years (time ratio, 0.82; 95% CI, 0.73-0.93).

**Conclusions and Relevance:**

In this cohort study of 8951 youths, the survival model suggested that being incarcerated in an adult correctional facility may be associated with an increased risk of early mortality between 18 and 39 years of age.

## Key Points

**Question:**

Is incarceration of youths in adult correctional facilities associated with an increased risk of mortality through 39 years of age?

**Findings:**

In this cohort study of 8951 youths, incarceration in an adult correctional facility before the age of 18 years was associated with a 33% increase in the risk of mortality between 18 and 39 years of age.

**Meaning:**

This study suggests that incarceration in an adult correctional facility as a youth was associated with early mortality, potentially through diminished psychological and physical health.

## Teaser

This cohort study uses data from the National Longitudinal Survey of Youth–1997 to evaluate whether being incarcerated in an adult correctional facility as a youth was associated with mortality between 18 and 39 years of age.

## Introduction

Incarceration exposure is associated with early mortality.^1,2,3^ Research documents a dose-response association such that more time served in prison corresponds to greater reductions in life span.^4^ Mortality excesses associated with incarceration translate to losses of life expectancy of 4 to 5 years, or roughly 13% of the average US life expectancy at the age of 45 years.^5^ Numerous mechanisms have been shown to link incarceration to early mortality, including greater risk for violent victimization and homicide,^6^ substance use and overdose,^7,8^ and higher incidence of chronic and infectious disease.^9,10^

A smaller body of research documents the association of incarceration exposure with early mortality specifically among youths, legally defined as children younger than 18 years.^11^ Youths with a history of incarceration have an all-cause mortality rate roughly 5.9 times higher than observed in nonincarcerated Medicaid-enrolled youths of the same age, associated largely with exposure to violence and homicide victimization.^6^ Incarcerated youths face greater risk for early death than nonincarcerated youths as involvement in the criminal legal system becomes more protracted and severe.^12^ Youths who are incarcerated experience health challenges related to dental care, sexual and reproductive health, risk-taking behaviors, and mental well-being that heighten the likelihood of early death.^13^

In most US states, youths can be transferred and sentenced in adult court, resulting in detention in adult prison facilities.^14,15^ Incarceration in juvenile vs adult correctional facilities represents vastly different experiences. Adult facilities are often much larger, have higher resident to staff ratios, and place less emphasis on treatment, counseling, and education.^16^ Even though only approximately 1% of formally processed juvenile delinquency cases are transferred to an adult criminal court,^17^ research documents serious extralegal consequences for youths incarcerated in adult facilities. Youths incarcerated in adult facilities report substantially greater rates of posttraumatic stress disorder and depression compared with those in juvenile facilities.^18,19,20^ In Texas, youths in adult facilities reported more distress and higher rates of psychiatric symptoms than those in juvenile centers.^18^ Most salient to the present study, researchers have observed that detention in jail and transfer to adult court were associated with early mortality among a sample of youths in Indiana.^12^

Young people housed in adult correctional facilities report being more afraid for their safety.^14^ Youths incarcerated in adult facilities are more likely to engage with antisocial adult peers in prison environments, exposing them to greater risk for sexual and physical assault.^18,21,22^ Youths in adult facilities often struggle to adjust to prison life and display heightened rates of disciplinary misconduct and violence, which can lead to further isolation and loss of socialization during critical developmental periods.^23^ Incarceration among adults is likely to have damaging iatrogenic effects for youths as a result of more harmful exposures in prison and disruption to key social, psychological, and developmental processes. Poorer mental and physical health, greater risk for violence and victimization, and higher engagement in risky behaviors, such as substance abuse, may be associated with early mortality after release for those who have been incarcerated as a youth in an adult facility.

We hypothesize that juvenile incarceration in an adult correctional facility will be associated with greater risk for early mortality after accounting for broader exposure to the criminal legal system via arrests, associated risk factors, and demographic differences.

## Methods

### Analytical Sample

The current study follows the Strengthening the Reporting of Observational Studies in Epidemiology (STROBE) reporting guideline for reporting by satisfying all the applicable items (1-21) in terms of reporting on a secondary publicly available cohort study. We did not seek institutional review board approval as the study relied on publicly available deidentified data that can be retrieved by anyone. The approval for the data collection was obtained by the institutional review boards at The Ohio State University and National Opinion Research Center at the University of Chicago by the NLSY97 research team, with which we have no affiliation. All data documentation associated with the National Longitudinal Study of Youth–1997 (NLSY97) is provided on the website of the US Bureau of Labor Statistics. The data for the current study are derived from the publicly available version of the NLSY97, a nationally representative sample of individuals born in the United States between January 1, 1980, and December 1, 1984.^24^ The 8984 respondents agreed to participate in yearly interviews from 1997 to 2011 and in interviews every 2 years from 2013 to 2019. In total, 19 interviews have been completed, with the respondents ranging in age from 12 to 18 years during the 1997 interview and from 34 to 39 years during the 2019 interview. If a respondent was incarcerated at the time of the interview, the interview was completed in person in a visitation room or via telephone at no cost to the respondent. The analytical sample included respondents who were 17 years of age or younger during the 1997 interview and alive during their 18th birthday (8951 individuals; >99% of the original sample). Because the study uses publicly available deidentified data, consent was not obtained from any respondents.

### Measures

#### Dependent Variables: Death and Age at Death

Respondents who did not complete an interview because they died were identified as part of the nonresponse tracking for the NLSY97. With the use of this information, 2 variables were created. First, a variable for death was created to indicate if a respondent died between 18 and 39 years of age (0 = no; 1 = yes). Of the 8951 individuals included in the sample, 225 (3%) died between 18 and 39 years of age. The mortality rate observed in the NLSY97 is consistent with the estimates produced by the US Social Security Office.^25^ Second, a respondent’s age at death was recorded as their age during the year they died, as reported by family members or friends. The NLSY97 research team did request and confirm death records for all respondents using official data sources from state agencies. Consistent with the implementation of a survival analysis, the age at the final interview was also recorded in the age at death measure for individuals who did not die between 18 and 39 years of age.

#### Key Independent Variables: Arrest and Incarceration in Adult Correctional Facilities

The respondents were requested to report the date of each arrest and the start and end date for each incarceration period since their last interview. The NLSY97 research team developed monthly arrays identifying the number of arrests a respondent experienced and if a respondent was incarcerated at any point during a specified month from 1992 to 2019. Due to concerns associated with privacy, the NLSY97 research team considered an individual incarcerated during a specified month if they spent 1 or more days in the correctional facility during that month. With the use of the monthly arrays and the age of the respondent each year, 2 variables were created to measure whether a respondent was arrested or incarcerated before the age of 18 years (0 = no; 1 = yes). Two continuous variables were created to measure the number of arrests and the number of months incarcerated that each respondent experienced before the age of 18 years. Periods of confinement associated with status offenses (eg, truancy), holds for DUI (driving under the influence of alcohol or drugs), holds for public intoxication, pretrial detention, and confinement in juvenile detention centers were excluded from the monthly arrays for incarceration created by the NLSY97 research team. The measures created for the current study capture data on whether an adolescent was incarcerated in an adult correctional facility (jail or prison) and the number of months spent in an adult correctional facility.^26^

#### Control Variables: General Risk Factors and Demographic Characteristics

Study variables were selected to limit the potential bias associated with colliders and confounders—and exclude mediators—following the development of a theoretically and empirically formed, directed acyclic graph (eAppendix 1 and eFigure in Supplement 1).^1,10,12,13^ Four covariates known to be associated with contact with the criminal legal system and early mortality were included in the survival model.^27,28^ These covariates include items measuring the self-rated general health of the respondent before the age of 18 years (0 = poor; 1 = fair; 2 = good; 3 = very good; 4 = excellent; measured in 1997),^29,30^ whether 1 or both of the respondent’s parents were incarcerated before the respondent turned 16 years of age, whether the respondent experienced childhood adversity (eg, experienced homelessness, lived in a place without water or electricity, or resided in emergency housing) before the age of 18 years, and the respondent’s household net worth before the age of 18 years.

In addition to these risk factors, demographic characteristics were adjusted for the inclusion of male sex (reference = female sex), ethnicity (Hispanic; reference = non-Hispanic), and race (American Indian or Alaska Native, Asian, Black, White; reference was other race [including individuals of multiple races and individuals who did not identify a race after initially identifying as Hispanic]). A dichotomous indicator (supplemental sample) for the NLSY97 sample in which the respondent participated (0 = initial sample; 1 = supplemental sample) was also included in the model to adjust for possible differences in the sampling procedures.^24^

### Statistical Analysis

Statistical analysis was performed from November 2022 to May 2023. A 4-part analytical strategy was implemented. First, a missing data analysis was conducted, and multiple imputation using the random forest method (continuous constructs) and the logistic regression method (dichotomous constructs) was implemented to impute missing values on the covariates (eAppendix 2, eTable 1, and eTable 2 in Supplement 1).^31^ The imputed values for the covariates represent the combination of 5 imputed data sets with 10 iterations each (pulled using the complete function from the mice package in R, version 4.2.3 [R Project for Statistical Computing]).^31^ No information was missing on the number of arrests, the number of months incarcerated, or the death status of the respondents. Second, descriptive statistics were produced. Third, death status and the age at death were regressed on the independent and control variables using a parametric survival model. The parametric survival model was estimated using a lognormal distribution given the distributional properties of age at death. An intercept-only model was estimated with each distributional specification—lognormal, exponential, Weibull, gaussian, logistic, and loglogistic—and the resulting Akaike information criteria were compared. A parametric survival model was preferred for the current analysis because the association between the independent variables and the dependent variable did not satisfy the proportional hazard assumption of a Cox proportional hazards regression survival model.^32,33,34^ The time ratio (TR) represents the risk of an event occurring across all time periods, where values higher than 1 indicate an increased odds of survival and values lower than 1 indicate an increased risk of death. The TR is calculated as the exponentiated value of the slope coefficient (exp [*b*]). The cumulative probability of death was calculated and plotted to permit a visual evaluation of the results. All *P* values were from 2-sided tests and results were deemed statistically significant at *P* < .05. All analyses were estimated using the survival^35^ and the SurvMetrics packages in R, version 4.2.3.^36^ To maintain open science, the R script used to clean the data and estimate all of the statistical analyses is provided in eAppendix 4 in Supplement 1.

## Results

The analytical sample of 8951 individuals included 4582 male participants (51%), 61 American Indian or Alaska Native participants (1%), 157 Asian participants (2%), 2438 Black participants (27%), 1895 Hispanic participants (ethnicity; 21%), 1065 participants of other race (12%), and 5233 White participants (59%) (Table 1). A total of 225 respondents (3%) died during the study period, with a mean (SD) age at death of 27.7 (5.9) years. A total of 1597 of the respondents (18%) in the analytical sample were arrested before the age of 18 years, while 109 respondents (1%) were incarcerated as youths in an adult correctional facility. Some youths experienced as many as 17 arrests and were incarcerated for up to 3.5 years in an adult facility.

**Table 1.** Descriptive Statistics for the Analytical Sample (N = 8951)

Characteristic	No. (%) (N = 8951)	Mean (SD)	Range
Key dependent variable (aged 18-39 y)			
Death	225 (3)	0.03 (0.16)	0 to 1
Age at death, y	NA	27.7 (5.9)	18 to 39
Key independent variables (before age 18 y)			
Incarcerated in adult facility	109 (1)	0.01 (0.11)	0 to 1
No. of months incarcerated in adult facility	NA	0.08 (1.12)	0 to 42
Arrested	1597 (18)	0.17 (0.37)	0 to 1
No. of arrests	NA	0.38 (1.19)	0 to 17
General risk factors			
Self-rated health before age 18 y	NA	3.06 (0.91)	0 to 4
Parental incarceration before age 16 y	623 (7)	0.07 (0.25)	0 to 1
Childhood adversity	489 (5)	0.05 (0.23)	0 to 1
Household net worth before the age of 18 y, $	NA	92 288 (139 363)	−935 251 to 600 000
Demographics			
Sex			
Male	4582 (51)	0.51 (0.50)	0 to 1
Female	4369 (49)	0.49 (0.50)	0 to 1
Ethnicity			
Hispanic	1895 (21)	0.21 (0.41)	0 to 1
Non-Hispanic	7056 (79)	0.79 (0.41)	0 to 1
Race			
American Indian or Alaska Native	61 (1)	0.006 (0.08)	0 to 1
Asian	157 (2)	0.02 (0.13)	0 to 1
Black	2438 (27)	0.27 (0.45)	0 to 1
Other^a^	1065 (12)	0.12 (0.32)	0 to 1
White	5233 (58)	0.58 (0.49)	0 to 1
Supplemental sample	2230 (25)	0.25 (0.43)	0 to 1

Abbreviation: NA, not applicable.

^a^ Includes individuals of multiple races and individuals who did not identify a race after initially identifying as Hispanic.

Table 2 provides the results of the parametric survival model that assessed the likelihood of death before the age of 39 years. Incarceration of youths in an adult correctional facility was associated with an approximate 33% increase in the risk of death (TR, 0.67; 95% CI, 0.47-0.95) compared with nonincarcerated youths. In addition, being arrested before the age of 18 years was associated with an increase in the risk of death until the age of 39 years (TR, 0.82; 95% CI, 0.73-0.93) compared with not being arrested. Neither the number of months of incarceration in an adult facility (TR, 1.02; 95% CI, 0.99-1.06) nor the number of arrests (TR, 1.00; 95% CI, 0.97-1.04) were associated with the risk of death prior to 39 years of age. Better general health before 18 years of age was associated with a higher likelihood of survival (TR, 1.10; 95% CI, 1.05-1.15), while being male was associated with a higher risk of early death (TR, 0.84; 95% CI, 0.77-0.91).

**Table 2.** Parametric Survival Model Regressing Age at Death on Arrest as a Youth and Incarceration in an Adult Facility as a Youth^a^

Dependent variable: age at death (18-39 y)	*b* (SE) [95% CI]	Time ratio (95% CI)
Key independent variables (before age of 18 y)		
Incarceration in adult facility^b^	−0.40 (0.18) [−0.75 to −0.05]	0.67 (0.47 to 0.95)
No. of months incarcerated in adult facility	0.02 (0.02) [−0.01 to 0.06]	1.02 (0.99 to 1.06)
Arrested^b^	−0.20 (0.06) [−0.32 to −0.07]	0.82 (0.73 to 0.93)
No. of arrests	0.00 (0.02) [−0.03 to 0.04]	1.00 (0.97 to 1.04)
General risk factors		
Self-rated health^b^	0.09 (0.02) [0.05 to 0.14]	1.10 (1.05 to 1.15)
Parental incarceration before 16 y^b^	−0.17 (0.07) [−0.31 to −0.04]	0.84 (0.74 to 0.96)
Childhood adversity	0.00 (0.09) [−0.17 to 0.17]	1.00 (0.84 to 1.18)
Household net worth before 18 y^b^	0.00 (0.00) [0.00 to 0.00]	1.00 (1.00 to 1.00)
Demographic characteristics		
Male^b^	−0.17 (0.04) [−0.26 to −0.09]	0.84 (0.77 to 0.91)
American Indian or Alaska Native (reference: other)	0.02 (0.30) [−0.57 to 0.61]	1.02 (0.56 to 1.84)
Asian (reference: other)	0.27 (0.28) [−0.28 to 0.81]	1.31 (0.76 to 2.26)
Black (reference: other)	−0.16 (0.10) [−0.36 to 0.04]	0.85 (0.70 to 1.04)
Hispanic (reference: non-Hispanic)	0.08 (0.07) [−0.06 to 0.22]	1.08 (0.94 to 1.25)
White (reference: other)	−0.07 (0.10) [−0.25 to 0.12]	0.94 (0.78 to 1.13)
Supplemental sample	0.05 (0.06) [−0.06 to 0.16]	1.05 (0.94 to 1.17)
Log (scale)	−0.32 (0.05) [NA]	NA
Model log likelihood	−1684.7	
Intercept only log likelihood	−1737.1	
Scale	0.723	
No.	8951	

Abbreviation: NA, not applicable.

^a^ Following the Akaike information criterion, it was determined that the parametric survival model should be estimated using a lognormal distribution given the distributional properties of age at death.

^b^ Indicates *P* < .05.

We plotted the cumulative probability of death for youths incarcerated in adult correctional facilities, arrested youths, and respondents without legal system contact before 18 years of age. As shown in the Figure, approximately 8% of youths incarcerated in adult correctional facilities were estimated to die by the age of 39 years. In comparison, just over 5% of youths arrested before 18 years of age and just over 2% of youths without legal system contact before the age of 18 years were estimated to have died by the age of 39 years. Overall, the findings suggest that contact with the legal system as a youth—both arrest and, especially, incarceration in adult correctional facilities—was associated with an increased risk of death between 18 and 39 years of age. Supplemental analyses (eAppendix 3, eTable 3, and eTable 4 in Supplement 1) assessed whether the findings held after accounting for time served in a juvenile facility. The association between incarceration in an adult correctional facility and an increased risk of death remained virtually identical (TR, 0.67; 95% CI, 0.47-0.95) (eTable 4 in Supplement 1).

**Figure.**
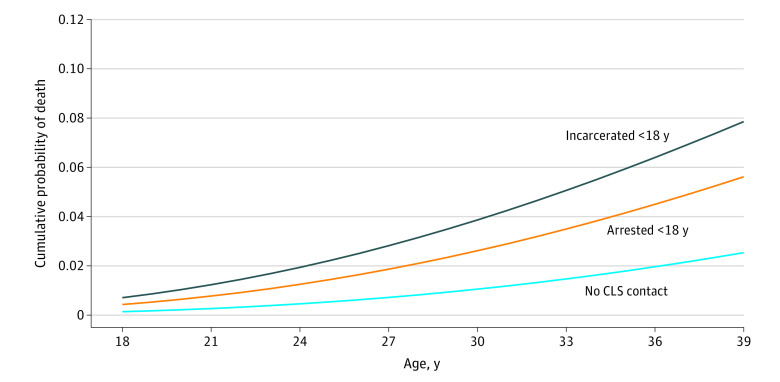
Cumulative Probability of Dying by Age for Varying Types of Contact With the Legal System The cumulative probability of dying by the corresponding age was estimated using the results from the model presented in Table 2. Incarcerated <18 y indicates ever spending time in an adult correctional facility as a youth. CLS indicates criminal legal system.

## Discussion

Although a relatively large body of literature highlights the detrimental associations of incarceration exposure with health overall, a much smaller segment has focused on such associations for youths imprisoned in adult facilities. Youths incarcerated in adult facilities experience a system that is not designed for the crucial developmental years of adolescence, where neuronal and social factors interact to affect personality and behavioral outcomes across the life course.^37^ Instead, such youths encounter a system intended exclusively for socially matured individuals. Within such a system, youths may not only engage in risky and harmful behaviors^37^ but they may directly experience risk factors associated with the likelihood of early mortality. Experiences of incarceration have been associated with a variety of detrimental outcomes related to well-being; however, to date, no study has examined the extent to which these experiences may be associated with premature death in a national sample, to our knowledge. The current study addressed this gap in the literature using a longitudinal, nationally representative sample of US youths.

The analyses revealed 3 key findings. First, while a relatively small proportion of youths in the analytical sample were incarcerated in an adult facility, there was substantial variability in the length of exposure. Second, our multivariable models accounted for several general risk factors associated with health, family background, and socioeconomic status, yet the association between exposure to an adult correctional facility and early mortality remained. This association is illustrated in the Figure, where the proportion of youths who were estimated to die by the age of 39 years appeared to be approximately 3 times higher for those who were incarcerated in an adult facility compared with those without contact with the legal system. Third, while it appears that any formal contact with the legal system was associated with an increased risk of premature death, being incarcerated in an adult facility as a youth evinced the highest risk for early mortality. The findings of the supplemental models further suggest that the circumstances to which youths are exposed during incarceration in adult correctional facilities could be associated with detrimental health outcomes because incarceration in juvenile correctional facilities was not associated with early mortality (eAppendix 3, eTable 3, and eTable 4 in Supplement 1).

Overall, these results point to a handful of potential explanations. First, any exposure to an adult correctional facility as a youth may have an association with early mortality beyond other risk factors, such as general health, being male, and early contact with the legal system. Several potential mechanisms are plausible that align with prior research on the detrimental effects of incarceration (eg, increased risk for violent victimization, substance use, disease, and harmful behaviors).^6,7,9^ These associations, however, were examined in prior literature using adult prisoners in adult facilities. Thus, it is conceivable that the associations are exacerbated when experienced by youths in an adult facility. Future research should further assess potential mechanisms of the association observed here.

Second, the observed association may not be causal but instead indicative of a process unrelated or tangentially related to the legal system. Youths who are incarcerated tend to possess health-related risk factors at much higher rates than youths who do not experience incarceration.^38,39^ Consequently, the mortality risk factors that youths bring with them to the incarceration process may be amplified by experiences in adult correctional facilities. Whether the association is causal or a function of the risk factors that youths bring with them (or both), the findings observed in the current study highlight the need for rethinking the practice of transferring youths to adult facilities to avoid the potential lethality of such exposure.

### Limitations

Although the current study illustrated an increased likelihood of early mortality for youths incarcerated in adult facilities, at least 5 limitations should temper the findings. First, there could be aspects of the NLSY97 cohort used to form the analytical sample that were associated with the observed findings.^40^ Second, the data do not provide for a nuanced assessment of the potential mechanisms for the observed patterns. For example, heterogeneity in the experience of adult facilities among youths transferred to such prisons in the analytical sample is a potential factor associated with the increased likelihood of early death. Third, due to data limitations, the NLSY97 does not identify the cause of death. Fourth, a limited number of individuals died between 18 and 39 years of age (n = 225), and the current study can be generalized only to the birth cohort of individuals born in the US between 1980 and 1984. Fifth, due to the existing literature and the measures available in the NLSY97, the current study adjusted only for mechanisms known to confound the association of interest. Future studies should consider additional confounders not measured or available in the NLSY97.

## Conclusions

Our cohort study illustrates that incarcerating youths in adult correctional facilities is potentially very harmful. The observed association between youth imprisonment in adult correctional facilities and increased risk of mortality further illustrates a need for reassessment of this practice. Furthermore, the results emphasize the importance of considering the health-related needs of youths while pursuing rehabilitation in prison.^9,10,18^ Prevention and intervention efforts should be directed at factors to ameliorate the potential extralegal harm—including lethal impacts—of placing youths in adult correctional facilities.

## Notes

Supplement 1. **eAppendix 1.** Directed Acyclic Graph to Guide Model Selection
eReferences.
**eFigure.** Directed Acyclic Graph Guiding the Selection of Covariates **eAppendix 2.** Missing Case Information **eTable 1.** Descriptive Statistics for the Analytical Sample Before Imputation (N = 8,951) **eTable 2.** Missing Case Comparison for Key Constructs Before Imputation (n = 8,951) **eAppendix 3.** Model Replications With Youth Incarcerated in Juvenile Facilities **eTable 3.** Parametric Survival Model Regressing Age at Death on Being Incarcerated in a Juvenile Facility **eTable 4.** Parametric Survival Model Regressing Age at Death on Being Incarcerated in a Juvenile Facility, Arrested as a Juvenile, and Incarcerated in an Adult Facility as a Juvenile **eAppendix 4.** R Script

Supplement 2.
Data Sharing Statement
