# Error in Wording

**DOI:** 10.1001/jamanetworkopen.2023.26692

**Published:** 2023-07-24

In the Original Investigation titled “Incarceration of Youths in an Adult Correctional Facility and Risk of Premature Death,”^1^ published July 5, 2023, there was an error in the Methods section; in the fourth sentence of the Statistical Analysis subsection, “combination” should be changed to “first” (the imputed values for the covariates represent the first of 5 imputed data sets). This article has been corrected.^1^
