# Supplementary material for: Incarceration of Youths in an Adult Correctional Facility and Risk of Premature Death
Source: JAMA Netw Open. 2023 Jul 5;6(7):e2321805. doi: 10.1001/jamanetworkopen.2023.21805 (PMC10323704; doi:10.1001/jamanetworkopen.2023.21805)
Supplement: Supplement 1. — eAppendix 1. Directed Acyclic Graph to Guide Model Selection eReferences. eFigure. Directed Acyclic Graph Guiding the Selection of Covariates eAppendix 2. Missing Case Information eTable 1. Descriptive Statistics for the Analytical Sample Before Imputation (N = 8,951) eTable 2. Missing Case Comparison for Key Constructs Before Imputation (n = 8,951) eAppendix 3. Model Replications With Youth Incarcerated in Juvenile Facilities eTable 3. Parametric Survival Model Regressing Age at Death on Being Incarcerated in a Juvenile Facility eTable 4. Parametric Survival Model Regressing Age at Death on Being Incarcerated in a Juvenile Facility, Arrested as a Juvenile, and Incarcerated in an Adult Facility as a Juvenile eAppendix 4. R Script [file jamanetwopen-e2321805-s001.pdf]

Supplementary Online Content

Silver IA, Semenza DC, Nedelec JL. Incarceration of youths in an adult correctional facility and risk of premature death. *JAMA Netw Open.* 2023;6(7):e2321805. doi:10.1001/jamanetworkopen.2023.21805

eAppendix 1. Directed Acyclic Graph to Guide Model Selection
eReferences.
eFigure. Directed Acyclic Graph Guiding the Selection of Covariates
eAppendix 2. Missing Case Information
eTable 1. Descriptive Statistics for the Analytical Sample Before Imputation (N = 8,951)
eTable 2. Missing Case Comparison for Key Constructs Before Imputation (n = 8,951)
eAppendix 3. Model Replications With Youth Incarcerated in Juvenile Facilities
eTable 3. Parametric Survival Model Regressing Age at Death on Being Incarcerated in a Juvenile Facility
eTable 4. Parametric Survival Model Regressing Age at Death on Being Incarcerated in a Juvenile Facility, Arrested as a Juvenile, and Incarcerated in an Adult Facility as a Juvenile
eAppendix 4. R Script

This supplementary material has been provided by the authors to give readers additional information about their work.

© 2023 Silver IA et al. *JAMA Network Open.*

eAppendix 1. Directed Acyclic Graph to Guide Model Selection

Given theory and prior findings in the literature, we postulated the mechanisms that could confound the association between youth incarceration in adult correctional facilities and early life death could be behaviorally related before the age of 12 including risk seeking, substance use, etc. Further, the mechanisms could also be associated with the environment in which the youth grew up before juvenile incarceration in an adult facility was measured. Unfortunately, due to data limitations we did not have access to proclivity for involvement in risky behaviors before the age of 12. Nonetheless, we believe that *arrested before the age of 18*, *number of arrests before the age of 18*, and *general health* serve as a proxy measures for related risky behaviors. In particular, being arrested as a youth represents involvement in serious risky behaviors that result in contact with law enforcement, which could influence both incarceration and the likelihood of early mortality. Similarly, general health serves as a self-reported indicator of risky health behaviors, which could influence both incarceration and the likelihood of early mortality.

While we cannot directly measure the environment in which the youth grew up, we believe childhood adversity, household income, parental incarceration, and the demographic characteristics of the youth serve as proxies and can be used to adjust for the potential confounding effects of the developmental environment. Childhood adversity and household income provide an indication of the likelihood of a youth being exposed to negative environmental circumstances, while parental incarceration is a direct indicator of having faced adverse childhood experiences. The demographics of the youth provide an indication of how they may be perceived by broader society.

In addition to the literature in the primary manuscript, the literature below helped guide the development of the Directed Acyclic Graph (DAG).

© 2023 Silver IA et al. *JAMA Network Open.*

eReferences.

Barrett DE, Katsiyannis A, Zhang D. Predictors of offense severity, adjudication, incarceration, and repeat referrals for juvenile offenders: A multicohort replication study. Remedial and Special Education. 2010 Jul;31(4):261-75.

Barnert ES, Perry R, Shetgiri R, Steers N, Dudovitz R, Heard-Garris NJ, Zima B, Chung PJ. Adolescent protective and risk factors for incarceration through early adulthood. Journal of Child and Family Studies. 2021 Jun;30:1428-40.

Costello BJ, Laub JH. Social control theory: The legacy of Travis Hirschi's causes of delinquency. Annual Review of Criminology. 2020 Jan 13;3:21-41.

Graf GH, Chihuri S, Blow M, Li G. Adverse childhood experiences and justice system contact: A systematic review. Pediatrics. 2021 Jan 1;147(1).

Huizinga D, Elliott DS. Juvenile offenders: Prevalence, offender incidence, and arrest rates by race. Crime & Delinquency. 1987 Apr;33(2):206-23.

Krisberg B, Schwartz I, Fishman G, Eisikovits Z, Guttman E, Joe K. The incarceration of minority youth. Crime & Delinquency. 1987 Apr;33(2):173-205.

Murray, J., Loeber, R., & Pardini, D. (2012). Parental involvement in the criminal justice system and the development of youth theft, marijuana use, depression, and poor academic performance. Criminology, 50(1), 255-302.

NeMoyer A, Wang Y, Alvarez K, Canino G, Duarte CS, Bird H, Alegría M. Parental incarceration during childhood and later delinquent outcomes among Puerto Rican adolescents and young adults in two contexts. Law and human behavior. 2020 Apr;44(2):143.

Swisher RR, Roettger ME. Father's incarceration and youth delinquency and depression: Examining differences by race and ethnicity. Journal of research on adolescence. 2012 Dec;22(4):597-603.

Testa A, Jackson DB, Ganson KT, Nagata JM. Adverse childhood experiences and criminal justice contact in adulthood. Academic pediatrics. 2022 Aug 1;22(6):972-80.

Turney, K. (2018). Adverse childhood experiences among children of incarcerated parents. Children and Youth Services Review, 89, 218-225.

© 2023 Silver IA et al. *JAMA Network Open.*

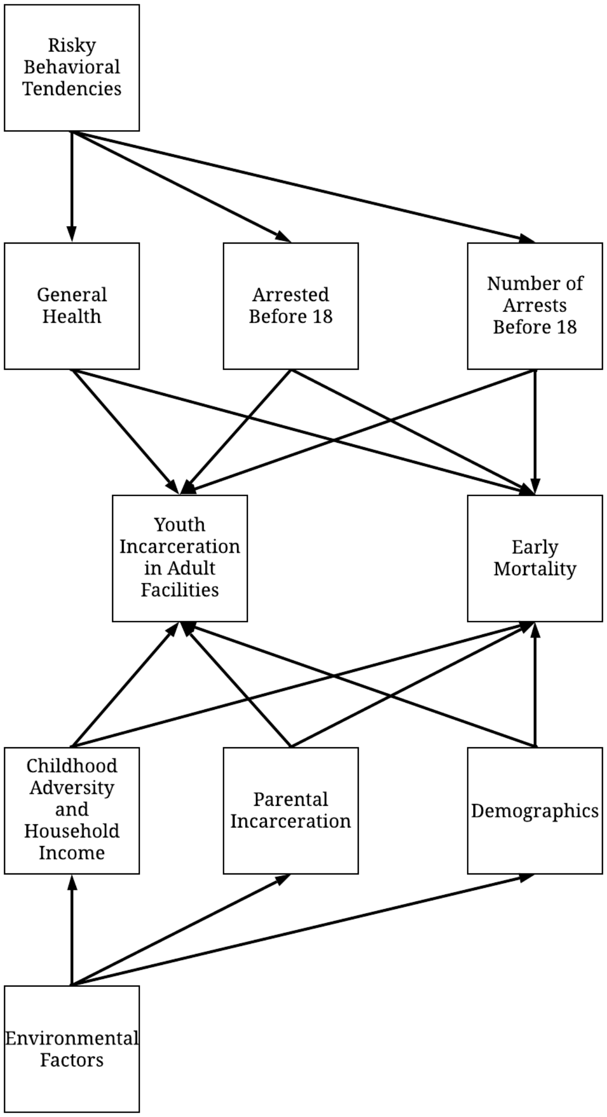

eFigure. Directed Acyclic Graph Guiding the Selection of Covariates

© 2023 Silver IA et al. *JAMA Network Open.*

eAppendix 2. Missing Case Information

eTable 1. Descriptive Statistics for the Analytical Sample Before Imputation (N = 8,951)

	n	*n* Missing	Mean (%)	SD	Min	Max
Key Dependent Variables (18-39 years old)						
Age at Death	225	0	27.84	5.93	18	39
Death	225	0	0.03	0.16	0	1
Key Independent Variables (Before Age 18)						
Incarcerated In Adult Facility	109	0	0.01	0.12	0	1
Number of Months Incarcerated in Adult Facility	--	0	0.08	1.14	0	42
Arrested	1,597	0	0.17	0.37	0	1
Number of Arrests	--	0	0.39	1.19	0	17
General Risk Factors						
Self-Rated Health	--	5	3.06	0.91	0	4
Parental Incarceration Before 16	537	1262	0.07	0.25	0	1
Childhood Adversity	435	1099	0.06	0.23	0	1
Household Net Worth Before 18	--	2291	89,717	137,100	-935251	600000
Demographics						
Male	4582	0	0.51	0.50	0	1
Ethnicity						
Hispanic	1892	23	0.21	0.41	0	1
Non-Hispanic	7036	23	79	0.41	0	1
Race						
American Indian	61	79	0.007	0.08	0	1
Asian	157	79	0.02	0.13	0	1
Black	2377	79	0.27	0.44	0	1
Other	1059	79	0.12	0.32	0	1
White	5218	79	0.59	0.49	0	1
Supplemental Sample	2,230	0	0.25	0.43	0	1

© 2023 Silver IA et al. *JAMA Network Open.*

eTable 2. Missing Case Comparison for Key Constructs Before Imputation (n = 8,951)

	Complete Cases		Cases with Missing Information		*t*	Cohen's D
	n	Mean	*n*	Mean		
Key Dependent Variables (18-39 years old)						
Age at Death	5656	25.77	3295	35.93	-17.03	-1.76
Death	5656	0.01	3295	0.05	10.49	0.28
Key Independent Variables (Before Age 18)						
Incarcerated In Adult Facility	5656	0.02	3295	0.01	-1.38	-0.03
Number of Months Incarcerated in Adult Facility	5656	0.09	3295	0.06	-1.49	-0.03
Arrested	5656	0.18	3295	0.16	-2.56	-0.05
Number of Arrests	5656	0.40	3295	0.34	-2.50	-0.05
General Risk Factors						
Self-Rated Health	5656	3.04	3295	3.09	2.92	0.06
Parental Incarceration Before 16	5656	0.07	2037	0.06	-2.34	-0.06
Childhood Adversity	5656	0.06	2197	0.05	-2.15	-0.05
Household Net Worth Before 18	5656	87926	1004	99811	2.51	0.09
Demographics						
Male	5656	0.50	3221	0.53	2.69	0.06
American Indian	5656	0.01	3221	0.01	-0.86	-0.02
Asian	5656	0.01	3221	0.03	4.42	0.11
Black	5656	0.27	3221	0.26	-0.58	-0.01
Hispanic	5656	0.21	3221	0.22	0.84	0.02
White	5656	0.59	3221	0.59	-0.11	0.00
Supplemental Sample	5656	0.25	3221	0.24	-1.01	0.02

© 2023 Silver IA et al. *JAMA Network Open.*

eAppendix 3. Model Replications With Youth Incarcerated in Juvenile Facilities

eTable 3. Parametric Survival Model Regressing Age at Death on Being Incarcerated in a Juvenile Facility

DV: Age At Death (18-39)	b	SE	b 95% CI		Time Ratio	Time Ratio 95% CI	
**Primary Independent Variable**							
Incarcerated in a Juvenile Facility	-0.18	0.11	-0.40	0.03	0.83	0.67	1.03
**General Risk Factors**							
Self-Rated Health*	0.10	0.02	0.05	0.14	1.10	1.06	1.15
Parental Incarceration Before 16*	-0.19	0.07	-0.33	-0.06	0.82	0.72	0.94
Childhood Adversity	-0.01	0.09	-0.18	0.16	0.99	0.84	1.17
Household Net Worth Before 18*	0.00	0.00	0.00	0.00	1.00	1.00	1.00
**Demographics**							
Male*	-0.20	0.04	-0.28	-0.11	0.82	0.75	0.89
American Indian (ref: Other)	0.05	0.30	-0.55	0.64	1.05	0.58	1.90
Asian (ref: Other)	0.29	0.28	-0.26	0.83	1.33	0.77	2.29
Black (ref: Other)	-0.15	0.10	-0.35	0.04	0.86	0.70	1.05
Hispanic (ref: non-Hispanic)	0.09	0.07	-0.05	0.23	1.09	0.95	1.26
White (ref: Other)	-0.06	0.09	-0.25	0.12	0.94	0.78	1.13
Supplemental Sample	0.05	0.06	-0.06	0.16	1.05	0.94	1.18
**Log(scale)**	-0.32	0.05					
Model LogLikelihood				-1692.7			
Intercept Only LogLikelihood				-1737.1			
Scale				0.73			
n				8951			

Notes: * $p < .05$; Incarcerated in a Juvenile Facility *n* = 189; mean(%) = 0.02(2); SD = 0.14; min, max = 0,1

© 2023 Silver IA et al. *JAMA Network Open.*

eTable 4. Parametric Survival Model Regressing Age at Death on Being Incarcerated in a Juvenile Facility, Arrested as a Juvenile, and Incarcerated in an Adult Facility as a Juvenile

DV: Age At Death (18-39)	b	SE	b 95% CI		Time Ratio	Time Ratio 95% CI	
**Primary Independent Variable**							
Incarcerated In Adult Facility*	-0.40	0.18	-0.76	-0.05	0.67	0.47	0.95
Number of Months Incarcerated in Adult Facility	0.02	0.02	-0.01	0.06	1.03	0.99	1.06
Arrested*	-0.19	0.06	-0.32	-0.07	0.82	0.73	0.93
Number of Arrests	0.00	0.02	-0.03	0.04	1.00	0.97	1.04
Incarcerated in a Juvenile Facility	-0.06	0.12	-0.29	0.18	0.95	0.75	1.19
**General Risk Factors**							
Self-Rated Health*	0.09	0.02	0.05	0.14	1.10	1.05	1.15
Parental Incarceration Before 16*	-0.17	0.07	-0.30	-0.04	0.84	0.74	0.96
Childhood Adversity	0.00	0.09	-0.17	0.17	1.00	0.84	1.18
Household Net Worth Before 18*	0.00	0.00	0.00	0.00	1.00	1.00	1.00
**Demographics**							
Male*	-0.17	0.04	-0.26	-0.09	0.84	0.77	0.92
American Indian (ref: Other)	0.02	0.30	-0.57	0.61	1.02	0.56	1.84
Asian (ref: Other)	0.27	0.28	-0.28	0.81	1.31	0.76	2.25
Black (ref: Other)	-0.16	0.10	-0.36	0.04	0.85	0.70	1.04
Hispanic (ref: non-Hispanic)	0.08	0.07	-0.06	0.22	1.09	0.94	1.25
White (ref: Other)	-0.07	0.10	-0.25	0.12	0.94	0.78	1.13
Supplemental Sample	0.05	0.06	-0.06	0.16	1.05	0.94	1.17
**Log(scale)**	-0.32	0.05					
Model LogLikelihood	-1684.6						
Intercept Only LogLikelihood	-1737.1						
Scale	0.72						
n	8951						

Notes: * $p < .05$

© 2023 Silver IA et al. *JAMA Network Open.*

eAppendix 4. R Script

```
# Getting Started ####

library(psych)
library(lavaan)
library("moments")
library("pastecs")
library("ggplot2")
library(survival)
library("data.table")
library(tidyverse)
library(dplyr)
library(ggridges)
library(extrafont)
library(Hmisc)
library(effsize)
library(pROC)
library(car)
library(parallel)
library(foreach)
library(doRNG)
library("ggpubr")
library("mice")
library(doParallel)
library(lm.beta)
library("ggfortify")
library("ranger")
library("survminer")
library("pec")
library(SurvMetrics)
library("lcmm")
detectCores()
registerDoParallel(12)
options(scipen=100, digits=12)
options(max.print = 500000000)


# Items Needed ####
#"R0000100","E8021201","E8021202","E8021203","E8021204","E8021205","E8021206"
,"E8021207","E8021208","E8021209","E8021210","E8021211","E8021212",
#"E8021301","E8021302","E8021303","E8021304","E8021305","E8021306","E8021307"
,"E8021308","E8021309","E8021310","E8021311","E8021312",
#"E8021401","E8021402","E8021403","E8021404","E8021405","E8021406","E8021407"
,"E8021408","E8021409","E8021410","E8021411","E8021412",
#"E8021501","E8021502","E8021503","E8021504","E8021505","E8021506","E8021507"
,"E8021508","E8021509","E8021510","E8021511","E8021512",
#"E8021601","E8021602","E8021603","E8021604","E8021605","E8021606","E8021607"
,"E8021608","E8021609","E8021610","E8021611","E8021612",
#"E8021701","E8021702","E8021703","E8021704","E8021705","E8021706","E8021707"
,"E8021708","E8021709","E8021710","E8021711","E8021712",
#"E8021801","E8021802","E8021803","E8021804","E8021805","E8021806","E8021807"
,"E8021808","E8021809","E8021810","E8021811","E8021812",
#"E8021901","E8021902","E8021903","E8021904","E8021905","E8021906","E8021907"
,"E8021908","E8021909","E8021910","E8021911","E8021912",
```

© 2023 Silver IA et al. *JAMA Network Open.*

```
#"E8022001","E8022002","E8022003","E8022004","E8022005","E8022006","E8022007"
,"E8022008","E8022009","E8022010","E8022011","E8022012",
#"E8022101","E8022102","E8022103","E8022104","E8022105","E8022106","E8022107"
,"E8022108","E8022109","E8022110","E8022111","E8022112",
#"E8022201","E8022202","E8022203","E8022204","E8022205","E8022206","E8022207"
,"E8022208","E8022209","E8022210","E8022211","E8022212",
#"E8022301","E8022302","E8022303","E8022304","E8022305","E8022306","E8022307"
,"E8022308","E8022309","E8022310","E8022311","E8022312",
#"E8022401","E8022402","E8022403","E8022404","E8022405","E8022406","E8022407"
,"E8022408","E8022409","E8022410","E8022411","E8022412",
#"E8022501","E8022502","E8022503","E8022504","E8022505","E8022506","E8022507"
,"E8022508","E8022509","E8022510","E8022511","E8022512",
#"E8022601","E8022602","E8022603","E8022604","E8022605","E8022606","E8022607"
,"E8022608","E8022609","E8022610","E8022611","E8022612",
#"E8022701","E8022702","E8022703","E8022704","E8022705","E8022706","E8022707"
,"E8022708","E8022709","E8022710","E8022711","E8022712",
#"E8022801","E8022802","E8022803","E8022804","E8022805","E8022806","E8022807"
,"E8022808","E8022809","E8022810","E8022811","E8022812",
#"E8022901","E8022902","E8022903","E8022904","E8022905","E8022906","E8022907"
,"E8022908","E8022909","E8022910","E8022911","E8022912",
#"E8023001","E8023002","E8023003","E8023004","E8023005","E8023006","E8023007"
,"E8023008","E8023009","E8023010","E8023011","E8023012",
#"E8023101","E8023102","E8023103","E8023104","E8023105","E8023106","E8023107"
,"E8023108","E8023109","E8023110","E8023111","E8023112",
#"E8023201","E8023202","E8023203","E8023204","E8023205","E8023206","E8023207"
,"E8023208","E8023209","E8023210","E8023211","E8023212",
#"E8023301","E8023302","E8023303","E8023304","E8023305","E8023306","E8023307"
,"E8023308","E8023309","E8023310","E8023311","E8023312",
#"E8023401","E8023402","E8023403","E8023404","E8023405","E8023406","E8023407"
,"E8023408","E8023409","E8023410","E8023411","E8023412",
#"E8023501","E8023502","E8023503","E8023504","E8023505","E8023506","E8023507"
,"E8023508","E8023509","E8023510","E8023511","E8023512",
#"E8023601","E8023602","E8023603","E8023604","E8023605","E8023606","E8023607"
,"E8023608","E8023609","E8023610","E8023611","E8023612",
#"E8023701","E8023702","E8023703","E8023704","E8023705","E8023706","E8023707"
,"E8023708","E8023709","E8023710","E8023711","E8023712",
#"E8033000","E8033100","E8033200","E8033900",
#"E8011201","E8011202","E8011203","E8011204","E8011205","E8011206","E8011207"
,"E8011208","E8011209","E8011210","E8011211","E8011212",
#"E8011301","E8011302","E8011303","E8011304","E8011305","E8011306","E8011307"
,"E8011308","E8011309","E8011310","E8011311","E8011312",
#"E8011401","E8011402","E8011403","E8011404","E8011405","E8011406","E8011407"
,"E8011408","E8011409","E8011410","E8011411","E8011412",
#"E8011501","E8011502","E8011503","E8011504","E8011505","E8011506","E8011507"
,"E8011508","E8011509","E8011510","E8011511","E8011512",
#"E8011601","E8011602","E8011603","E8011604","E8011605","E8011606","E8011607"
,"E8011608","E8011609","E8011610","E8011611","E8011612",
#"E8011701","E8011702","E8011703","E8011704","E8011705","E8011706","E8011707"
,"E8011708","E8011709","E8011710","E8011711","E8011712",
#"E8011801","E8011802","E8011803","E8011804","E8011805","E8011806","E8011807"
,"E8011808","E8011809","E8011810","E8011811","E8011812",
#"E8011901","E8011902","E8011903","E8011904","E8011905","E8011906","E8011907"
,"E8011908","E8011909","E8011910","E8011911","E8011912",
#"E8012001","E8012002","E8012003","E8012004","E8012005","E8012006","E8012007"
,"E8012008","E8012009","E8012010","E8012011","E8012012",
```

© 2023 Silver IA et al. *JAMA Network Open.*

```
#"E8012101","E8012102","E8012103","E8012104","E8012105","E8012106","E8012107"
,"E8012108","E8012109","E8012110","E8012111","E8012112",
#"E8012201","E8012202","E8012203","E8012204","E8012205","E8012206","E8012207"
,"E8012208","E8012209","E8012210","E8012211","E8012212",
#"E8012301","E8012302","E8012303","E8012304","E8012305","E8012306","E8012307"
,"E8012308","E8012309","E8012310","E8012311","E8012312",
#"E8012401","E8012402","E8012403","E8012404","E8012405","E8012406","E8012407"
,"E8012408","E8012409","E8012410","E8012411","E8012412",
#"E8012501","E8012502","E8012503","E8012504","E8012505","E8012506","E8012507"
,"E8012508","E8012509","E8012510","E8012511","E8012512",
#"E8012601","E8012602","E8012603","E8012604","E8012605","E8012606","E8012607"
,"E8012608","E8012609","E8012610","E8012611","E8012612",
#"E8012701","E8012702","E8012703","E8012704","E8012705","E8012706","E8012707"
,"E8012708","E8012709","E8012710","E8012711","E8012712",
#"E8012801","E8012802","E8012803","E8012804","E8012805","E8012806","E8012807"
,"E8012808","E8012809","E8012810","E8012811","E8012812",
#"E8012901","E8012902","E8012903","E8012904","E8012905","E8012906","E8012907"
,"E8012908","E8012909","E8012910","E8012911","E8012912",
#"E8013001","E8013002","E8013003","E8013004","E8013005","E8013006","E8013007"
,"E8013008","E8013009","E8013010","E8013011","E8013012",
#"E8013101","E8013102","E8013103","E8013104","E8013105","E8013106","E8013107"
,"E8013108","E8013109","E8013110","E8013111","E8013112",
#"E8013201","E8013202","E8013203","E8013204","E8013205","E8013206","E8013207"
,"E8013208","E8013209","E8013210","E8013211","E8013212",
#"E8013301","E8013302","E8013303","E8013304","E8013305","E8013306","E8013307"
,"E8013308","E8013309","E8013310","E8013311","E8013312",
#"E8013401","E8013402","E8013403","E8013404","E8013405","E8013406","E8013407"
,"E8013408","E8013409","E8013410","E8013411","E8013412",
#"E8013501","E8013502","E8013503","E8013504","E8013505","E8013506","E8013507"
,"E8013508","E8013509","E8013510","E8013511","E8013512",
#"E8013601","E8013602","E8013603","E8013604","E8013605","E8013606","E8013607"
,"E8013608","E8013609","E8013610","E8013611","E8013612",
#"E8013701","E8013702","E8013703","E8013704","E8013705","E8013706","E8013707"
,"E8013708","E8013709","E8013710","E8013711","E8013712",
#"R1194100","R2553500","R3876300","R5453700","R7216000","S1531400","S2001000"
,"S3801100","S5401000","S7501200","T0008500","T2011100",
#"R0536300","R0538700","R0538600","R1235800","R9829600","T8218800","T8219200"
,"U0113400","U0113800","U1966600","U1967000","R1204500","R1204600","R1204700"
,"R1204800",
#"R0532200","R0649100","R0648900",
#"R2510200","R3827700","R5341500","R7085400","S1524700","S3590300","S4966600"
,"S6706700","S8679600","T1099500","T3176800","T4587900",
#"T6221000","T7718200","T9118900",
#"U1110400","U1700600","U3300300","R0320600","R1204500","R1204600","R1204700"
,"R1204800","R1193900","R0536401","R0536402"

# Data Cleaning ####


## Age Variables

## Age each year
A$R1193900
table(A$R1193900/12)

A$MAGE92<-A$R1193900-(5*12)
```

© 2023 Silver IA et al. *JAMA Network Open.*

```
table(A$MAGE92)
A$MAGE93<-A$R1193900-(4*12)
table(A$MAGE93)
A$MAGE94<-A$R1193900-(3*12)
table(A$MAGE94)
A$MAGE95<-A$R1193900-(2*12)
table(A$MAGE95)
A$MAGE96<-A$R1193900-(1*12)
table(A$MAGE96)
A$MAGE97<-A$R1193900
table(A$MAGE97)
A$MAGE98<-A$R1193900+(1*12)
table(A$MAGE98)
A$MAGE99<-A$R1193900+(2*12)
table(A$MAGE99)
A$MAGE00<-A$R1193900+(3*12)
table(A$MAGE00)
A$MAGE01<-A$R1193900+(4*12)
table(A$MAGE01)
A$MAGE02<-A$R1193900+(5*12)
table(A$MAGE02)
A$MAGE03<-A$R1193900+(6*12)
table(A$MAGE03)
A$MAGE04<-A$R1193900+(7*12)
table(A$MAGE04)
A$MAGE05<-A$R1193900+(8*12)
table(A$MAGE05)
A$MAGE06<-A$R1193900+(9*12)
table(A$MAGE06)
A$MAGE07<-A$R1193900+(10*12)
table(A$MAGE07)
A$MAGE08<-A$R1193900+(11*12)
table(A$MAGE08)
A$MAGE09<-A$R1193900+(12*12)
table(A$MAGE09)
A$MAGE10<-A$R1193900+(13*12)
table(A$MAGE10)
A$MAGE11<-A$R1193900+(14*12)
table(A$MAGE11)
A$MAGE12<-A$R1193900+(15*12)
table(A$MAGE12, useNA = "always")
A$MAGE13<-A$R1193900+(16*12)
table(A$MAGE13, useNA = "always")
A$MAGE14<-A$R1193900+(17*12)
table(A$MAGE14)
A$MAGE15<-A$R1193900+(18*12)
table(A$MAGE15)
A$MAGE16<-A$R1193900+(19*12)
table(A$MAGE16)
A$MAGE17<-A$R1193900+(20*12)
table(A$MAGE17)
A$MAGE18<-A$R1193900+(21*12)
table(A$MAGE18)
A$MAGE19<-A$R1193900+(22*12)
table(A$MAGE19)
```

© 2023 Silver IA et al. *JAMA Network Open.*

```
summary(A$MAGE01)
summary(A$MAGE02)
summary(A$MAGE03)
summary(A$MAGE04)
summary(A$MAGE05)
summary(A$MAGE06)
summary(A$MAGE07)
summary(A$MAGE08)
summary(A$MAGE09)
summary(A$MAGE10)



A$AGE92<-trunc(A$MAGE92/12)
A$AGE93<-trunc(A$MAGE93/12)
A$AGE94<-trunc(A$MAGE94/12)
A$AGE95<-trunc(A$MAGE95/12)
A$AGE96<-trunc(A$MAGE96/12)
A$AGE97<-trunc(A$MAGE97/12)
A$AGE98<-trunc(A$MAGE98/12)
A$AGE99<-trunc(A$MAGE99/12)
A$AGE00<-trunc(A$MAGE00/12)
A$AGE01<-trunc(A$MAGE01/12)
A$AGE02<-trunc(A$MAGE02/12)
A$AGE03<-trunc(A$MAGE03/12)
A$AGE04<-trunc(A$MAGE04/12)
A$AGE05<-trunc(A$MAGE05/12)
A$AGE06<-trunc(A$MAGE06/12)
A$AGE07<-trunc(A$MAGE07/12)
A$AGE08<-trunc(A$MAGE08/12)
A$AGE09<-trunc(A$MAGE09/12)
A$AGE10<-trunc(A$MAGE10/12)
A$AGE11<-trunc(A$MAGE11/12)
A$AGE12<-trunc(A$MAGE12/12)
A$AGE13<-trunc(A$MAGE13/12)
A$AGE14<-trunc(A$MAGE14/12)
A$AGE15<-trunc(A$MAGE15/12)
A$AGE16<-trunc(A$MAGE16/12)
A$AGE17<-trunc(A$MAGE17/12)
A$AGE18<-trunc(A$MAGE18/12)
A$AGE19<-trunc(A$MAGE19/12)

summary(A$AGE92)
summary(A$MAGE02)
summary(A$MAGE03)
summary(A$MAGE04)
summary(A$MAGE05)
summary(A$MAGE06)
summary(A$MAGE07)
summary(A$MAGE08)
summary(A$MAGE09)
summary(A$MAGE10)

## Date Born
```

© 2023 Silver IA et al. *JAMA Network Open.*

```
table(A$R0536401)
table(A$R0536402)
A$Day_Born[A$R0536401 == 1 & A$R0536402== 1980]<-"1980-01-01"
A$Day_Born[A$R0536401 == 2 & A$R0536402== 1980]<-"1980-02-01"
A$Day_Born[A$R0536401 == 3 & A$R0536402== 1980]<-"1980-03-01"
A$Day_Born[A$R0536401 == 4 & A$R0536402== 1980]<-"1980-04-01"
A$Day_Born[A$R0536401 == 5 & A$R0536402== 1980]<-"1980-05-01"
A$Day_Born[A$R0536401 == 6 & A$R0536402== 1980]<-"1980-06-01"
A$Day_Born[A$R0536401 == 7 & A$R0536402== 1980]<-"1980-07-01"
A$Day_Born[A$R0536401 == 8 & A$R0536402== 1980]<-"1980-08-01"
A$Day_Born[A$R0536401 == 9 & A$R0536402== 1980]<-"1980-09-01"
A$Day_Born[A$R0536401 == 10 & A$R0536402== 1980]<-"1980-10-01"
A$Day_Born[A$R0536401 == 11 & A$R0536402== 1980]<-"1980-11-01"
A$Day_Born[A$R0536401 == 12 & A$R0536402== 1980]<-"1980-12-01"
A$Day_Born[A$R0536401 == 1 & A$R0536402== 1981]<-"1981-01-01"
A$Day_Born[A$R0536401 == 2 & A$R0536402== 1981]<-"1981-02-01"
A$Day_Born[A$R0536401 == 3 & A$R0536402== 1981]<-"1981-03-01"
A$Day_Born[A$R0536401 == 4 & A$R0536402== 1981]<-"1981-04-01"
A$Day_Born[A$R0536401 == 5 & A$R0536402== 1981]<-"1981-05-01"
A$Day_Born[A$R0536401 == 6 & A$R0536402== 1981]<-"1981-06-01"
A$Day_Born[A$R0536401 == 7 & A$R0536402== 1981]<-"1981-07-01"
A$Day_Born[A$R0536401 == 8 & A$R0536402== 1981]<-"1981-08-01"
A$Day_Born[A$R0536401 == 9 & A$R0536402== 1981]<-"1981-09-01"
A$Day_Born[A$R0536401 == 10 & A$R0536402== 1981]<-"1981-10-01"
A$Day_Born[A$R0536401 == 11 & A$R0536402== 1981]<-"1981-11-01"
A$Day_Born[A$R0536401 == 12 & A$R0536402== 1981]<-"1981-12-01"
A$Day_Born[A$R0536401 == 1 & A$R0536402== 1982]<-"1982-01-01"
A$Day_Born[A$R0536401 == 2 & A$R0536402== 1982]<-"1982-02-01"
A$Day_Born[A$R0536401 == 3 & A$R0536402== 1982]<-"1982-03-01"
A$Day_Born[A$R0536401 == 4 & A$R0536402== 1982]<-"1982-04-01"
A$Day_Born[A$R0536401 == 5 & A$R0536402== 1982]<-"1982-05-01"
A$Day_Born[A$R0536401 == 6 & A$R0536402== 1982]<-"1982-06-01"
A$Day_Born[A$R0536401 == 7 & A$R0536402== 1982]<-"1982-07-01"
A$Day_Born[A$R0536401 == 8 & A$R0536402== 1982]<-"1982-08-01"
A$Day_Born[A$R0536401 == 9 & A$R0536402== 1982]<-"1982-09-01"
A$Day_Born[A$R0536401 == 10 & A$R0536402== 1982]<-"1982-10-01"
A$Day_Born[A$R0536401 == 11 & A$R0536402== 1982]<-"1982-11-01"
A$Day_Born[A$R0536401 == 12 & A$R0536402== 1982]<-"1982-12-01"
A$Day_Born[A$R0536401 == 1 & A$R0536402== 1983]<-"1983-01-01"
A$Day_Born[A$R0536401 == 2 & A$R0536402== 1983]<-"1983-02-01"
A$Day_Born[A$R0536401 == 3 & A$R0536402== 1983]<-"1983-03-01"
A$Day_Born[A$R0536401 == 4 & A$R0536402== 1983]<-"1983-04-01"
A$Day_Born[A$R0536401 == 5 & A$R0536402== 1983]<-"1983-05-01"
A$Day_Born[A$R0536401 == 6 & A$R0536402== 1983]<-"1983-06-01"
A$Day_Born[A$R0536401 == 7 & A$R0536402== 1983]<-"1983-07-01"
A$Day_Born[A$R0536401 == 8 & A$R0536402== 1983]<-"1983-08-01"
A$Day_Born[A$R0536401 == 9 & A$R0536402== 1983]<-"1983-09-01"
A$Day_Born[A$R0536401 == 10 & A$R0536402== 1983]<-"1983-10-01"
A$Day_Born[A$R0536401 == 11 & A$R0536402== 1983]<-"1983-11-01"
A$Day_Born[A$R0536401 == 12 & A$R0536402== 1983]<-"1983-12-01"
A$Day_Born[A$R0536401 == 1 & A$R0536402== 1984]<-"1984-01-01"
A$Day_Born[A$R0536401 == 2 & A$R0536402== 1984]<-"1984-02-01"
A$Day_Born[A$R0536401 == 3 & A$R0536402== 1984]<-"1984-03-01"
A$Day_Born[A$R0536401 == 4 & A$R0536402== 1984]<-"1984-04-01"
A$Day_Born[A$R0536401 == 5 & A$R0536402== 1984]<-"1984-05-01"
A$Day_Born[A$R0536401 == 6 & A$R0536402== 1984]<-"1984-06-01"
```

```
A$Day_Born[A$R0536401 == 7 & A$R0536402== 1984]<-"1984-07-01"
A$Day_Born[A$R0536401 == 8 & A$R0536402== 1984]<-"1984-08-01"
A$Day_Born[A$R0536401 == 9 & A$R0536402== 1984]<-"1984-09-01"
A$Day_Born[A$R0536401 == 10 & A$R0536402== 1984]<-"1984-10-01"
A$Day_Born[A$R0536401 == 11 & A$R0536402== 1984]<-"1984-11-01"
A$Day_Born[A$R0536401 == 12 & A$R0536402== 1984]<-"1984-12-01"
A$Day_Born<-as.Date(A$Day_Born, format = "%Y-%m-%d")
class(A$Day_Born)
table(A$Day_Born)

## Date turned 18
A$Month_T18<-A$Day_Born + years(18)

## Deceased Identifier #
### Deceased by year (98)
A$Dec98<-0
A$Dec98[A$R2510200 == 80 | A$R2510200 == 98]<-1
table(A$Dec98)
### Deceased by year (99)
A$Dec99<-0
A$Dec99[A$R3827700 == 80 | A$R3827700 == 98]<-1
table(A$Dec99)
### Deceased by year (00)
A$Dec00<-0
A$Dec00[A$R5341500 == 80 | A$R5341500 == 98]<-1
table(A$Dec00)
### Deceased by year (01)
A$Dec01<-0
A$Dec01[A$R7085400 == 80 | A$R7085400 == 98]<-1
table(A$Dec01)
### Deceased by year (02)
A$Dec02<-0
A$Dec02[A$S1524700 == 80 | A$S1524700 == 98]<-1
table(A$Dec02)
### Deceased by year (03)
A$Dec03<-0
A$Dec03[A$S3590300 == 80 | A$S3590300 == 98]<-1
table(A$Dec03)
### Deceased by year (04)
A$Dec04<-0
A$Dec04[A$S4966600 == 80 | A$S4966600 == 98]<-1
table(A$Dec04)
### Deceased by year (05)
A$Dec05<-0
A$Dec05[A$S6706700 == 80 | A$S6706700 == 98]<-1
table(A$Dec05)
### Deceased by year (06)
A$Dec06<-0
A$Dec06[A$S8679600 == 80 | A$S8679600 == 98]<-1
table(A$Dec06)
### Deceased by year (07)
A$Dec07<-0
A$Dec07[A$T1099500 == 80 | A$T1099500 == 98]<-1
table(A$Dec07)
### Deceased by year (08)
A$Dec08<-0
```

© 2023 Silver IA et al. *JAMA Network Open.*

```
A$Dec08[A$T3176800 == 80 | A$T3176800 == 98]<-1
table(A$Dec08)
### Deceased by year (09)
A$Dec09<-0
A$Dec09[A$T4587900 == 80 | A$T4587900 == 98]<-1
table(A$Dec09)
### Deceased by year (10)
A$Dec10<-0
A$Dec10[A$T6221000 == 80 | A$T6221000 == 98]<-1
table(A$Dec10)
### Deceased by year (11)
A$Dec11<-0
A$Dec11[A$T7718200 == 80 | A$T7718200 == 98]<-1
table(A$Dec11)
### Deceased by year (13)
A$Dec13<-0
A$Dec13[A$T9118900 == 80 | A$T9118900 == 98]<-1
table(A$Dec13)
### Deceased by year (15)
A$Dec15<-0
A$Dec15[A$U1110400 == 80 | A$U1110400 == 98]<-1
table(A$Dec15)
### Deceased by year (17)
A$Dec17<-0
A$Dec17[A$U1700600 == 80 | A$U1700600 == 98]<-1
table(A$Dec17)
### Deceased by year (19)
A$Dec19<-0
A$Dec19[A$U3300300 == 80 | A$U3300300 == 98]<-1
table(A$Dec19)
## Deceased by Age
### Deceased Age 7
A$Dec_Age7<-NA
A$Dec_Age7[is.na(A$Dec_Age7) &A$AGE98==7]<-A$Dec98[is.na(A$Dec_Age7)
&A$AGE98==7]
table(A$Dec_Age7,useNA = "always")
A$Dec_Age7[is.na(A$Dec_Age7) &A$AGE99==7]<-A$Dec99[is.na(A$Dec_Age7)
&A$AGE99==7]
table(A$Dec_Age7,useNA = "always")
A$Dec_Age7[is.na(A$Dec_Age7) & A$AGE00==7]<-A$Dec00[is.na(A$Dec_Age7)
&A$AGE00==7]
table(A$Dec_Age7,useNA = "always")
A$Dec_Age7[is.na(A$Dec_Age7) & A$AGE01==7]<-A$Dec01[is.na(A$Dec_Age7)
&A$AGE01==7]
table(A$Dec_Age7,useNA = "always")
A$Dec_Age7[is.na(A$Dec_Age7) & A$AGE02==7]<-A$Dec02[is.na(A$Dec_Age7)
&A$AGE02==7]
table(A$Dec_Age7,useNA = "always")
A$Dec_Age7[is.na(A$Dec_Age7) & A$AGE03==7]<-A$Dec03[is.na(A$Dec_Age7)
&A$AGE03==7]
table(A$Dec_Age7,useNA = "always")
A$Dec_Age7[is.na(A$Dec_Age7) & A$AGE04==7]<-A$Dec04[is.na(A$Dec_Age7)
&A$AGE04==7]
table(A$Dec_Age7,useNA = "always")
A$Dec_Age7[is.na(A$Dec_Age7) & A$AGE05==7]<-A$Dec05[is.na(A$Dec_Age7)
&A$AGE05==7]
```

© 2023 Silver IA et al. *JAMA Network Open.*

```
table(A$Dec_Age7,useNA = "always")
A$Dec_Age7[is.na(A$Dec_Age7) & A$AGE06==7]<-A$Dec06[is.na(A$Dec_Age7)
&A$AGE06==7]
table(A$Dec_Age7,useNA = "always")
A$Dec_Age7[is.na(A$Dec_Age7) & A$AGE07==7]<-A$Dec07[is.na(A$Dec_Age7)
&A$AGE07==7]
table(A$Dec_Age7,useNA = "always")
A$Dec_Age7[is.na(A$Dec_Age7) & A$AGE08==7]<-A$Dec08[is.na(A$Dec_Age7)
&A$AGE08==7]
table(A$Dec_Age7,useNA = "always")
A$Dec_Age7[is.na(A$Dec_Age7) & A$AGE09==7]<-A$Dec09[is.na(A$Dec_Age7)
&A$AGE09==7]
table(A$Dec_Age7,useNA = "always")
A$Dec_Age7[is.na(A$Dec_Age7) & A$AGE10==7]<-A$Dec10[is.na(A$Dec_Age7)
&A$AGE10==7]
table(A$Dec_Age7,useNA = "always")
A$Dec_Age7[is.na(A$Dec_Age7) & A$AGE11==7]<-A$Dec11[is.na(A$Dec_Age7)
&A$AGE11==7]
table(A$Dec_Age7,useNA = "always")
A$Dec_Age7[is.na(A$Dec_Age7) & A$AGE12==7]<-A$Dec12[is.na(A$Dec_Age7)
&A$AGE12==7]
table(A$Dec_Age7,useNA = "always")
A$Dec_Age7[is.na(A$Dec_Age7) & A$AGE13==7]<-A$Dec13[is.na(A$Dec_Age7)
&A$AGE13==7]
table(A$Dec_Age7,useNA = "always")
A$Dec_Age7[is.na(A$Dec_Age7) & A$AGE14==7]<-A$Dec14[is.na(A$Dec_Age7)
&A$AGE14==7]
table(A$Dec_Age7,useNA = "always")
A$Dec_Age7[is.na(A$Dec_Age7) & A$AGE15==7]<-A$Dec15[is.na(A$Dec_Age7)
&A$AGE15==7]
table(A$Dec_Age7,useNA = "always")
A$Dec_Age7[is.na(A$Dec_Age7) & A$AGE16==7]<-A$Dec16[is.na(A$Dec_Age7)
&A$AGE16==7]
table(A$Dec_Age7,useNA = "always")
A$Dec_Age7[is.na(A$Dec_Age7) & A$AGE17==7]<-A$Dec17[is.na(A$Dec_Age7)
&A$AGE17==7]
table(A$Dec_Age7,useNA = "always")
### Deceased Age 8
A$Dec_Age8<-NA
A$Dec_Age8[A$Dec_Age7==1]<-1
A$Dec_Age8[is.na(A$Dec_Age8) &A$AGE98==8]<-A$Dec98[is.na(A$Dec_Age8)
&A$AGE98==8]
table(A$Dec_Age8,useNA = "always")
A$Dec_Age8[is.na(A$Dec_Age8) &A$AGE99==8]<-A$Dec99[is.na(A$Dec_Age8)
&A$AGE99==8]
table(A$Dec_Age8,useNA = "always")
A$Dec_Age8[is.na(A$Dec_Age8) & A$AGE00==8]<-A$Dec00[is.na(A$Dec_Age8)
&A$AGE00==8]
table(A$Dec_Age8,useNA = "always")
A$Dec_Age8[is.na(A$Dec_Age8) & A$AGE01==8]<-A$Dec01[is.na(A$Dec_Age8)
&A$AGE01==8]
table(A$Dec_Age8,useNA = "always")
A$Dec_Age8[is.na(A$Dec_Age8) & A$AGE02==8]<-A$Dec02[is.na(A$Dec_Age8)
&A$AGE02==8]
table(A$Dec_Age8,useNA = "always")
```

```
A$Dec_Age8[is.na(A$Dec_Age8) & A$AGE03==8]<-A$Dec03[is.na(A$Dec_Age8)
&A$AGE03==8]
table(A$Dec_Age8,useNA = "always")
A$Dec_Age8[is.na(A$Dec_Age8) & A$AGE04==8]<-A$Dec04[is.na(A$Dec_Age8)
&A$AGE04==8]
table(A$Dec_Age8,useNA = "always")
A$Dec_Age8[is.na(A$Dec_Age8) & A$AGE05==8]<-A$Dec05[is.na(A$Dec_Age8)
&A$AGE05==8]
table(A$Dec_Age8,useNA = "always")
A$Dec_Age8[is.na(A$Dec_Age8) & A$AGE06==8]<-A$Dec06[is.na(A$Dec_Age8)
&A$AGE06==8]
table(A$Dec_Age8,useNA = "always")
A$Dec_Age8[is.na(A$Dec_Age8) & A$AGE07==8]<-A$Dec07[is.na(A$Dec_Age8)
&A$AGE07==8]
table(A$Dec_Age8,useNA = "always")
A$Dec_Age8[is.na(A$Dec_Age8) & A$AGE08==8]<-A$Dec08[is.na(A$Dec_Age8)
&A$AGE08==8]
table(A$Dec_Age8,useNA = "always")
A$Dec_Age8[is.na(A$Dec_Age8) & A$AGE09==8]<-A$Dec09[is.na(A$Dec_Age8)
&A$AGE09==8]
table(A$Dec_Age8,useNA = "always")
A$Dec_Age8[is.na(A$Dec_Age8) & A$AGE10==8]<-A$Dec10[is.na(A$Dec_Age8)
&A$AGE10==8]
table(A$Dec_Age8,useNA = "always")
A$Dec_Age8[is.na(A$Dec_Age8) & A$AGE11==8]<-A$Dec11[is.na(A$Dec_Age8)
&A$AGE11==8]
table(A$Dec_Age8,useNA = "always")
A$Dec_Age8[is.na(A$Dec_Age8) & A$AGE12==8]<-A$Dec12[is.na(A$Dec_Age8)
&A$AGE12==8]
table(A$Dec_Age8,useNA = "always")
A$Dec_Age8[is.na(A$Dec_Age8) & A$AGE13==8]<-A$Dec13[is.na(A$Dec_Age8)
&A$AGE13==8]
table(A$Dec_Age8,useNA = "always")
A$Dec_Age8[is.na(A$Dec_Age8) & A$AGE14==8]<-A$Dec14[is.na(A$Dec_Age8)
&A$AGE14==8]
table(A$Dec_Age8,useNA = "always")
A$Dec_Age8[is.na(A$Dec_Age8) & A$AGE15==8]<-A$Dec15[is.na(A$Dec_Age8)
&A$AGE15==8]
table(A$Dec_Age8,useNA = "always")
A$Dec_Age8[is.na(A$Dec_Age8) & A$AGE16==8]<-A$Dec16[is.na(A$Dec_Age8)
&A$AGE16==8]
table(A$Dec_Age8,useNA = "always")
A$Dec_Age8[is.na(A$Dec_Age8) & A$AGE17==8]<-A$Dec17[is.na(A$Dec_Age8)
&A$AGE17==8]
table(A$Dec_Age8,useNA = "always")

### Deceased Age 9
A$Dec_Age9<-NA
A$Dec_Age9[A$Dec_Age8==1]<-1
A$Dec_Age9[is.na(A$Dec_Age9) &A$AGE98==9]<-A$Dec98[is.na(A$Dec_Age9)
&A$AGE98==9]
table(A$Dec_Age9,useNA = "always")
A$Dec_Age9[is.na(A$Dec_Age9) &A$AGE99==9]<-A$Dec99[is.na(A$Dec_Age9)
&A$AGE99==9]
table(A$Dec_Age9,useNA = "always")
```

© 2023 Silver IA et al. *JAMA Network Open.*

```
A$Dec_Age9[is.na(A$Dec_Age9) & A$AGE00==9]<-A$Dec00[is.na(A$Dec_Age9)
&A$AGE00==9]
table(A$Dec_Age9,useNA = "always")
A$Dec_Age9[is.na(A$Dec_Age9) & A$AGE01==9]<-A$Dec01[is.na(A$Dec_Age9)
&A$AGE01==9]
table(A$Dec_Age9,useNA = "always")
A$Dec_Age9[is.na(A$Dec_Age9) & A$AGE02==9]<-A$Dec02[is.na(A$Dec_Age9)
&A$AGE02==9]
table(A$Dec_Age9,useNA = "always")
A$Dec_Age9[is.na(A$Dec_Age9) & A$AGE03==9]<-A$Dec03[is.na(A$Dec_Age9)
&A$AGE03==9]
table(A$Dec_Age9,useNA = "always")
A$Dec_Age9[is.na(A$Dec_Age9) & A$AGE04==9]<-A$Dec04[is.na(A$Dec_Age9)
&A$AGE04==9]
table(A$Dec_Age9,useNA = "always")
A$Dec_Age9[is.na(A$Dec_Age9) & A$AGE05==9]<-A$Dec05[is.na(A$Dec_Age9)
&A$AGE05==9]
table(A$Dec_Age9,useNA = "always")
A$Dec_Age9[is.na(A$Dec_Age9) & A$AGE06==9]<-A$Dec06[is.na(A$Dec_Age9)
&A$AGE06==9]
table(A$Dec_Age9,useNA = "always")
A$Dec_Age9[is.na(A$Dec_Age9) & A$AGE07==9]<-A$Dec07[is.na(A$Dec_Age9)
&A$AGE07==9]
table(A$Dec_Age9,useNA = "always")
A$Dec_Age9[is.na(A$Dec_Age9) & A$AGE08==9]<-A$Dec08[is.na(A$Dec_Age9)
&A$AGE08==9]
table(A$Dec_Age9,useNA = "always")
A$Dec_Age9[is.na(A$Dec_Age9) & A$AGE09==9]<-A$Dec09[is.na(A$Dec_Age9)
&A$AGE09==9]
table(A$Dec_Age9,useNA = "always")
A$Dec_Age9[is.na(A$Dec_Age9) & A$AGE10==9]<-A$Dec10[is.na(A$Dec_Age9)
&A$AGE10==9]
table(A$Dec_Age9,useNA = "always")
A$Dec_Age9[is.na(A$Dec_Age9) & A$AGE11==9]<-A$Dec11[is.na(A$Dec_Age9)
&A$AGE11==9]
table(A$Dec_Age9,useNA = "always")
A$Dec_Age9[is.na(A$Dec_Age9) & A$AGE12==9]<-A$Dec12[is.na(A$Dec_Age9)
&A$AGE12==9]
table(A$Dec_Age9,useNA = "always")
A$Dec_Age9[is.na(A$Dec_Age9) & A$AGE13==9]<-A$Dec13[is.na(A$Dec_Age9)
&A$AGE13==9]
table(A$Dec_Age9,useNA = "always")
A$Dec_Age9[is.na(A$Dec_Age9) & A$AGE14==9]<-A$Dec14[is.na(A$Dec_Age9)
&A$AGE14==9]
table(A$Dec_Age9,useNA = "always")
A$Dec_Age9[is.na(A$Dec_Age9) & A$AGE15==9]<-A$Dec15[is.na(A$Dec_Age9)
&A$AGE15==9]
table(A$Dec_Age9,useNA = "always")
A$Dec_Age9[is.na(A$Dec_Age9) & A$AGE16==9]<-A$Dec16[is.na(A$Dec_Age9)
&A$AGE16==9]
table(A$Dec_Age9,useNA = "always")
A$Dec_Age9[is.na(A$Dec_Age9) & A$AGE17==9]<-A$Dec17[is.na(A$Dec_Age9)
&A$AGE17==9]
table(A$Dec_Age9,useNA = "always")

### Deceased Age 10
```

© 2023 Silver IA et al. *JAMA Network Open.*

```
A$Dec_Age10<-NA
A$Dec_Age10[A$Dec_Age9==1]<-1
A$Dec_Age10[is.na(A$Dec_Age10) &A$AGE98==10]<-A$Dec98[is.na(A$Dec_Age10)
&A$AGE98==10]
table(A$Dec_Age10,useNA = "always")
A$Dec_Age10[is.na(A$Dec_Age10) &A$AGE99==10]<-A$Dec99[is.na(A$Dec_Age10)
&A$AGE09==10]
table(A$Dec_Age10,useNA = "always")
A$Dec_Age10[is.na(A$Dec_Age10) & A$AGE00==10]<-A$Dec00[is.na(A$Dec_Age10)
&A$AGE00==10]
table(A$Dec_Age10,useNA = "always")
A$Dec_Age10[is.na(A$Dec_Age10) & A$AGE01==10]<-A$Dec01[is.na(A$Dec_Age10)
&A$AGE01==10]
table(A$Dec_Age10,useNA = "always")
A$Dec_Age10[is.na(A$Dec_Age10) & A$AGE02==10]<-A$Dec02[is.na(A$Dec_Age10)
&A$AGE02==10]
table(A$Dec_Age10,useNA = "always")
A$Dec_Age10[is.na(A$Dec_Age10) & A$AGE03==10]<-A$Dec03[is.na(A$Dec_Age10)
&A$AGE03==10]
table(A$Dec_Age10,useNA = "always")
A$Dec_Age10[is.na(A$Dec_Age10) & A$AGE04==10]<-A$Dec04[is.na(A$Dec_Age10)
&A$AGE04==10]
table(A$Dec_Age10,useNA = "always")
A$Dec_Age10[is.na(A$Dec_Age10) & A$AGE05==10]<-A$Dec05[is.na(A$Dec_Age10)
&A$AGE05==10]
table(A$Dec_Age10,useNA = "always")
A$Dec_Age10[is.na(A$Dec_Age10) & A$AGE06==10]<-A$Dec06[is.na(A$Dec_Age10)
&A$AGE06==10]
table(A$Dec_Age10,useNA = "always")
A$Dec_Age10[is.na(A$Dec_Age10) & A$AGE07==10]<-A$Dec07[is.na(A$Dec_Age10)
&A$AGE07==10]
table(A$Dec_Age10,useNA = "always")
A$Dec_Age10[is.na(A$Dec_Age10) & A$AGE08==10]<-A$Dec08[is.na(A$Dec_Age10)
&A$AGE08==10]
table(A$Dec_Age10,useNA = "always")
A$Dec_Age10[is.na(A$Dec_Age10) & A$AGE09==10]<-A$Dec09[is.na(A$Dec_Age10)
&A$AGE09==10]
table(A$Dec_Age10,useNA = "always")
A$Dec_Age10[is.na(A$Dec_Age10) & A$AGE10==10]<-A$Dec10[is.na(A$Dec_Age10)
&A$AGE10==10]
table(A$Dec_Age10,useNA = "always")
A$Dec_Age10[is.na(A$Dec_Age10) & A$AGE11==10]<-A$Dec11[is.na(A$Dec_Age10)
&A$AGE11==10]
table(A$Dec_Age10,useNA = "always")
A$Dec_Age10[is.na(A$Dec_Age10) & A$AGE12==10]<-A$Dec12[is.na(A$Dec_Age10)
&A$AGE12==10]
table(A$Dec_Age10,useNA = "always")
A$Dec_Age10[is.na(A$Dec_Age10) & A$AGE13==10]<-A$Dec13[is.na(A$Dec_Age10)
&A$AGE13==10]
table(A$Dec_Age10,useNA = "always")
A$Dec_Age10[is.na(A$Dec_Age10) & A$AGE14==10]<-A$Dec14[is.na(A$Dec_Age10)
&A$AGE14==10]
table(A$Dec_Age10,useNA = "always")
A$Dec_Age10[is.na(A$Dec_Age10) & A$AGE15==10]<-A$Dec15[is.na(A$Dec_Age10)
&A$AGE15==10]
table(A$Dec_Age10,useNA = "always")
```

© 2023 Silver IA et al. *JAMA Network Open.*

```
A$Dec_Age10[is.na(A$Dec_Age10) & A$AGE16==10]<-A$Dec16[is.na(A$Dec_Age10)
&A$AGE16==10]
table(A$Dec_Age10,useNA = "always")
A$Dec_Age10[is.na(A$Dec_Age10) & A$AGE17==10]<-A$Dec17[is.na(A$Dec_Age10)
&A$AGE17==10]
table(A$Dec_Age10,useNA = "always")

### Deceased Age 11
A$Dec_Age11<-NA
A$Dec_Age11[A$Dec_Age10==1]<-1
A$Dec_Age11[is.na(A$Dec_Age11) &A$AGE98==11]<-A$Dec98[is.na(A$Dec_Age11)
&A$AGE98==11]
table(A$Dec_Age11,useNA = "always")
A$Dec_Age11[is.na(A$Dec_Age11) &A$AGE99==11]<-A$Dec99[is.na(A$Dec_Age11)
&A$AGE99==11]
table(A$Dec_Age11,useNA = "always")
A$Dec_Age11[is.na(A$Dec_Age11) & A$AGE00==11]<-A$Dec00[is.na(A$Dec_Age11)
&A$AGE00==11]
table(A$Dec_Age11,useNA = "always")
A$Dec_Age11[is.na(A$Dec_Age11) & A$AGE01==11]<-A$Dec01[is.na(A$Dec_Age11)
&A$AGE01==11]
table(A$Dec_Age11,useNA = "always")
A$Dec_Age11[is.na(A$Dec_Age11) & A$AGE02==11]<-A$Dec02[is.na(A$Dec_Age11)
&A$AGE02==11]
table(A$Dec_Age11,useNA = "always")
A$Dec_Age11[is.na(A$Dec_Age11) & A$AGE03==11]<-A$Dec03[is.na(A$Dec_Age11)
&A$AGE03==11]
table(A$Dec_Age11,useNA = "always")
A$Dec_Age11[is.na(A$Dec_Age11) & A$AGE04==11]<-A$Dec04[is.na(A$Dec_Age11)
&A$AGE04==11]
table(A$Dec_Age11,useNA = "always")
A$Dec_Age11[is.na(A$Dec_Age11) & A$AGE05==11]<-A$Dec05[is.na(A$Dec_Age11)
&A$AGE05==11]
table(A$Dec_Age11,useNA = "always")
A$Dec_Age11[is.na(A$Dec_Age11) & A$AGE06==11]<-A$Dec06[is.na(A$Dec_Age11)
&A$AGE06==11]
table(A$Dec_Age11,useNA = "always")
A$Dec_Age11[is.na(A$Dec_Age11) & A$AGE07==11]<-A$Dec07[is.na(A$Dec_Age11)
&A$AGE07==11]
table(A$Dec_Age11,useNA = "always")
A$Dec_Age11[is.na(A$Dec_Age11) & A$AGE08==11]<-A$Dec08[is.na(A$Dec_Age11)
&A$AGE08==11]
table(A$Dec_Age11,useNA = "always")
A$Dec_Age11[is.na(A$Dec_Age11) & A$AGE09==11]<-A$Dec09[is.na(A$Dec_Age11)
&A$AGE09==11]
table(A$Dec_Age11,useNA = "always")
A$Dec_Age11[is.na(A$Dec_Age11) & A$Age10==11]<-A$Dec10[is.na(A$Dec_Age11)
&A$Age10==11]
table(A$Dec_Age11,useNA = "always")
A$Dec_Age11[is.na(A$Dec_Age11) & A$AGE11==11]<-A$Dec11[is.na(A$Dec_Age11)
&A$AGE11==11]
table(A$Dec_Age11,useNA = "always")
A$Dec_Age11[is.na(A$Dec_Age11) & A$AGE12==11]<-A$Dec12[is.na(A$Dec_Age11)
&A$AGE12==11]
table(A$Dec_Age11,useNA = "always")
```

© 2023 Silver IA et al. *JAMA Network Open.*

```
A$Dec_Age11[is.na(A$Dec_Age11) & A$AGE13==11]<-A$Dec13[is.na(A$Dec_Age11)
&A$AGE13==11]
table(A$Dec_Age11,useNA = "always")
A$Dec_Age11[is.na(A$Dec_Age11) & A$AGE14==11]<-A$Dec14[is.na(A$Dec_Age11)
&A$AGE14==11]
table(A$Dec_Age11,useNA = "always")
A$Dec_Age11[is.na(A$Dec_Age11) & A$AGE15==11]<-A$Dec15[is.na(A$Dec_Age11)
&A$AGE15==11]
table(A$Dec_Age11,useNA = "always")
A$Dec_Age11[is.na(A$Dec_Age11) & A$AGE16==11]<-A$Dec16[is.na(A$Dec_Age11)
&A$AGE16==11]
table(A$Dec_Age11,useNA = "always")
A$Dec_Age11[is.na(A$Dec_Age11) & A$AGE17==11]<-A$Dec17[is.na(A$Dec_Age11)
&A$AGE17==11]
table(A$Dec_Age11,useNA = "always")

### Deceased Age 12
A$Dec_Age12<-NA
A$Dec_Age12[A$Dec_Age11==1]<-1
A$Dec_Age12[is.na(A$Dec_Age12) &A$AGE98==12]<-A$Dec98[is.na(A$Dec_Age12)
&A$AGE98==12]
table(A$Dec_Age12,useNA = "always")
A$Dec_Age12[is.na(A$Dec_Age12) &A$AGE99==12]<-A$Dec99[is.na(A$Dec_Age12)
&A$AGE99==12]
table(A$Dec_Age12,useNA = "always")
A$Dec_Age12[is.na(A$Dec_Age12) & A$AGE00==12]<-A$Dec00[is.na(A$Dec_Age12)
&A$AGE00==12]
table(A$Dec_Age12,useNA = "always")
A$Dec_Age12[is.na(A$Dec_Age12) & A$AGE01==12]<-A$Dec01[is.na(A$Dec_Age12)
&A$AGE01==12]
table(A$Dec_Age12,useNA = "always")
A$Dec_Age12[is.na(A$Dec_Age12) & A$AGE02==12]<-A$Dec02[is.na(A$Dec_Age12)
&A$AGE02==12]
table(A$Dec_Age12,useNA = "always")
A$Dec_Age12[is.na(A$Dec_Age12) & A$AGE03==12]<-A$Dec03[is.na(A$Dec_Age12)
&A$AGE03==12]
table(A$Dec_Age12,useNA = "always")
A$Dec_Age12[is.na(A$Dec_Age12) & A$AGE04==12]<-A$Dec04[is.na(A$Dec_Age12)
&A$AGE04==12]
table(A$Dec_Age12,useNA = "always")
A$Dec_Age12[is.na(A$Dec_Age12) & A$AGE05==12]<-A$Dec05[is.na(A$Dec_Age12)
&A$AGE05==12]
table(A$Dec_Age12,useNA = "always")
A$Dec_Age12[is.na(A$Dec_Age12) & A$AGE06==12]<-A$Dec06[is.na(A$Dec_Age12)
&A$AGE06==12]
table(A$Dec_Age12,useNA = "always")
A$Dec_Age12[is.na(A$Dec_Age12) & A$AGE07==12]<-A$Dec07[is.na(A$Dec_Age12)
&A$AGE07==12]
table(A$Dec_Age12,useNA = "always")
A$Dec_Age12[is.na(A$Dec_Age12) & A$AGE08==12]<-A$Dec08[is.na(A$Dec_Age12)
&A$AGE08==12]
table(A$Dec_Age12,useNA = "always")
A$Dec_Age12[is.na(A$Dec_Age12) & A$AGE09==12]<-A$Dec09[is.na(A$Dec_Age12)
&A$AGE09==12]
table(A$Dec_Age12,useNA = "always")
```

© 2023 Silver IA et al. *JAMA Network Open.*

```
A$Dec_Age12[is.na(A$Dec_Age12) & A$AGE10==12]<-A$Dec10[is.na(A$Dec_Age12)
&A$AGE10==12]
table(A$Dec_Age12,useNA = "always")
A$Dec_Age12[is.na(A$Dec_Age12) & A$AGE11==12]<-A$Dec11[is.na(A$Dec_Age12)
&A$AGE11==12]
table(A$Dec_Age12,useNA = "always")
A$Dec_Age12[is.na(A$Dec_Age12) & A$AGE12==12]<-A$Dec12[is.na(A$Dec_Age12)
&A$AGE12==12]
table(A$Dec_Age12,useNA = "always")
A$Dec_Age12[is.na(A$Dec_Age12) & A$AGE13==12]<-A$Dec13[is.na(A$Dec_Age12)
&A$AGE13==12]
table(A$Dec_Age12,useNA = "always")
A$Dec_Age12[is.na(A$Dec_Age12) & A$AGE14==12]<-A$Dec14[is.na(A$Dec_Age12)
&A$AGE14==12]
table(A$Dec_Age12,useNA = "always")
A$Dec_Age12[is.na(A$Dec_Age12) & A$AGE15==12]<-A$Dec15[is.na(A$Dec_Age12)
&A$AGE15==12]
table(A$Dec_Age12,useNA = "always")
A$Dec_Age12[is.na(A$Dec_Age12) & A$AGE16==12]<-A$Dec16[is.na(A$Dec_Age12)
&A$AGE16==12]
table(A$Dec_Age12,useNA = "always")
A$Dec_Age12[is.na(A$Dec_Age12) & A$AGE17==12]<-A$Dec17[is.na(A$Dec_Age12)
&A$AGE17==12]
table(A$Dec_Age12,useNA = "always")

### Deceased Age 13
A$Dec_Age13<-NA
A$Dec_Age13[A$Dec_Age12==1]<-1
A$Dec_Age13[is.na(A$Dec_Age13) &A$AGE98==13]<-A$Dec98[is.na(A$Dec_Age13)
&A$AGE98==13]
table(A$Dec_Age13,useNA = "always")
A$Dec_Age13[is.na(A$Dec_Age13) &A$AGE99==13]<-A$Dec99[is.na(A$Dec_Age13)
&A$AGE99==13]
table(A$Dec_Age13,useNA = "always")
A$Dec_Age13[is.na(A$Dec_Age13) & A$AGE00==13]<-A$Dec00[is.na(A$Dec_Age13)
&A$AGE00==13]
table(A$Dec_Age13,useNA = "always")
A$Dec_Age13[is.na(A$Dec_Age13) & A$AGE01==13]<-A$Dec01[is.na(A$Dec_Age13)
&A$AGE01==13]
table(A$Dec_Age13,useNA = "always")
A$Dec_Age13[is.na(A$Dec_Age13) & A$AGE02==13]<-A$Dec02[is.na(A$Dec_Age13)
&A$AGE02==13]
table(A$Dec_Age13,useNA = "always")
A$Dec_Age13[is.na(A$Dec_Age13) & A$AGE03==13]<-A$Dec03[is.na(A$Dec_Age13)
&A$AGE03==13]
table(A$Dec_Age13,useNA = "always")
A$Dec_Age13[is.na(A$Dec_Age13) & A$AGE04==13]<-A$Dec04[is.na(A$Dec_Age13)
&A$AGE04==13]
table(A$Dec_Age13,useNA = "always")
A$Dec_Age13[is.na(A$Dec_Age13) & A$AGE05==13]<-A$Dec05[is.na(A$Dec_Age13)
&A$AGE05==13]
table(A$Dec_Age13,useNA = "always")
A$Dec_Age13[is.na(A$Dec_Age13) & A$AGE06==13]<-A$Dec06[is.na(A$Dec_Age13)
&A$AGE06==13]
table(A$Dec_Age13,useNA = "always")
```

© 2023 Silver IA et al. *JAMA Network Open.*

```
A$Dec_Age13[is.na(A$Dec_Age13) & A$AGE07==13]<-A$Dec07[is.na(A$Dec_Age13)
&A$AGE07==13]
table(A$Dec_Age13,useNA = "always")
A$Dec_Age13[is.na(A$Dec_Age13) & A$AGE08==13]<-A$Dec08[is.na(A$Dec_Age13)
&A$AGE08==13]
table(A$Dec_Age13,useNA = "always")
A$Dec_Age13[is.na(A$Dec_Age13) & A$AGE09==13]<-A$Dec09[is.na(A$Dec_Age13)
&A$AGE09==13]
table(A$Dec_Age13,useNA = "always")
A$Dec_Age13[is.na(A$Dec_Age13) & A$AGE10==13]<-A$Dec10[is.na(A$Dec_Age13)
&A$AGE10==13]
table(A$Dec_Age13,useNA = "always")
A$Dec_Age13[is.na(A$Dec_Age13) & A$AGE11==13]<-A$Dec11[is.na(A$Dec_Age13)
&A$AGE11==13]
table(A$Dec_Age13,useNA = "always")
A$Dec_Age13[is.na(A$Dec_Age13) & A$AGE12==13]<-A$Dec12[is.na(A$Dec_Age13)
&A$AGE12==13]
table(A$Dec_Age13,useNA = "always")
A$Dec_Age13[is.na(A$Dec_Age13) & A$AGE13==13]<-A$Dec13[is.na(A$Dec_Age13)
&A$AGE13==13]
table(A$Dec_Age13,useNA = "always")
A$Dec_Age13[is.na(A$Dec_Age13) & A$AGE14==13]<-A$Dec14[is.na(A$Dec_Age13)
&A$AGE14==13]
table(A$Dec_Age13,useNA = "always")
A$Dec_Age13[is.na(A$Dec_Age13) & A$AGE15==13]<-A$Dec15[is.na(A$Dec_Age13)
&A$AGE15==13]
table(A$Dec_Age13,useNA = "always")
A$Dec_Age13[is.na(A$Dec_Age13) & A$AGE16==13]<-A$Dec16[is.na(A$Dec_Age13)
&A$AGE16==13]
table(A$Dec_Age13,useNA = "always")
A$Dec_Age13[is.na(A$Dec_Age13) & A$AGE17==13]<-A$Dec17[is.na(A$Dec_Age13)
&A$AGE17==13]
table(A$Dec_Age13,useNA = "always")

### Deceased Age 14
A$Dec_Age14<-NA
A$Dec_Age14[A$Dec_Age13==1]<-1
A$Dec_Age14[is.na(A$Dec_Age14) &A$AGE98==14]<-A$Dec98[is.na(A$Dec_Age14)
&A$AGE98==14]
table(A$Dec_Age14,useNA = "always")
A$Dec_Age14[is.na(A$Dec_Age14) &A$AGE99==14]<-A$Dec99[is.na(A$Dec_Age14)
&A$AGE99==14]
table(A$Dec_Age14,useNA = "always")
A$Dec_Age14[is.na(A$Dec_Age14) & A$AGE00==14]<-A$Dec00[is.na(A$Dec_Age14)
&A$AGE00==14]
table(A$Dec_Age14,useNA = "always")
A$Dec_Age14[is.na(A$Dec_Age14) & A$AGE01==14]<-A$Dec01[is.na(A$Dec_Age14)
&A$AGE01==14]
table(A$Dec_Age14,useNA = "always")
A$Dec_Age14[is.na(A$Dec_Age14) & A$AGE02==14]<-A$Dec02[is.na(A$Dec_Age14)
&A$AGE02==14]
table(A$Dec_Age14,useNA = "always")
A$Dec_Age14[is.na(A$Dec_Age14) & A$AGE03==14]<-A$Dec03[is.na(A$Dec_Age14)
&A$AGE03==14]
table(A$Dec_Age14,useNA = "always")
```

© 2023 Silver IA et al. *JAMA Network Open.*

```
A$Dec_Age14[is.na(A$Dec_Age14) & A$AGE04==14]<-A$Dec04[is.na(A$Dec_Age14)
&A$AGE04==14]
table(A$Dec_Age14,useNA = "always")
A$Dec_Age14[is.na(A$Dec_Age14) & A$AGE05==14]<-A$Dec05[is.na(A$Dec_Age14)
&A$AGE05==14]
table(A$Dec_Age14,useNA = "always")
A$Dec_Age14[is.na(A$Dec_Age14) & A$AGE06==14]<-A$Dec06[is.na(A$Dec_Age14)
&A$AGE06==14]
table(A$Dec_Age14,useNA = "always")
A$Dec_Age14[is.na(A$Dec_Age14) & A$AGE07==14]<-A$Dec07[is.na(A$Dec_Age14)
&A$AGE07==14]
table(A$Dec_Age14,useNA = "always")
A$Dec_Age14[is.na(A$Dec_Age14) & A$AGE08==14]<-A$Dec08[is.na(A$Dec_Age14)
&A$AGE08==14]
table(A$Dec_Age14,useNA = "always")
A$Dec_Age14[is.na(A$Dec_Age14) & A$AGE09==14]<-A$Dec09[is.na(A$Dec_Age14)
&A$AGE09==14]
table(A$Dec_Age14,useNA = "always")
A$Dec_Age14[is.na(A$Dec_Age14) & A$AGE10==14]<-A$Dec10[is.na(A$Dec_Age14)
&A$AGE10==14]
table(A$Dec_Age14,useNA = "always")
A$Dec_Age14[is.na(A$Dec_Age14) & A$AGE11==14]<-A$Dec11[is.na(A$Dec_Age14)
&A$AGE11==14]
table(A$Dec_Age14,useNA = "always")
A$Dec_Age14[is.na(A$Dec_Age14) & A$AGE12==14]<-A$Dec12[is.na(A$Dec_Age14)
&A$AGE12==14]
table(A$Dec_Age14,useNA = "always")
A$Dec_Age14[is.na(A$Dec_Age14) & A$AGE13==14]<-A$Dec13[is.na(A$Dec_Age14)
&A$AGE13==14]
table(A$Dec_Age14,useNA = "always")
A$Dec_Age14[is.na(A$Dec_Age14) & A$AGE14==14]<-A$Dec14[is.na(A$Dec_Age14)
&A$AGE14==14]
table(A$Dec_Age14,useNA = "always")
A$Dec_Age14[is.na(A$Dec_Age14) & A$AGE15==14]<-A$Dec15[is.na(A$Dec_Age14)
&A$AGE15==14]
table(A$Dec_Age14,useNA = "always")
A$Dec_Age14[is.na(A$Dec_Age14) & A$AGE16==14]<-A$Dec16[is.na(A$Dec_Age14)
&A$AGE16==14]
table(A$Dec_Age14,useNA = "always")
A$Dec_Age14[is.na(A$Dec_Age14) & A$AGE17==14]<-A$Dec17[is.na(A$Dec_Age14)
&A$AGE17==14]
table(A$Dec_Age14,useNA = "always")
### Deceased Age 15
A$Dec_Age15<-NA
A$Dec_Age15[A$Dec_Age14==1]<-1
A$Dec_Age15[is.na(A$Dec_Age15) &A$AGE98==15]<-A$Dec98[is.na(A$Dec_Age15)
&A$AGE98==15]
table(A$Dec_Age15,useNA = "always")
A$Dec_Age15[is.na(A$Dec_Age15) &A$AGE99==15]<-A$Dec99[is.na(A$Dec_Age15)
&A$AGE99==15]
table(A$Dec_Age15,useNA = "always")
A$Dec_Age15[is.na(A$Dec_Age15) & A$AGE00==15]<-A$Dec00[is.na(A$Dec_Age15)
&A$AGE00==15]
table(A$Dec_Age15,useNA = "always")
A$Dec_Age15[is.na(A$Dec_Age15) & A$AGE01==15]<-A$Dec01[is.na(A$Dec_Age15)
&A$AGE01==15]
```

© 2023 Silver IA et al. *JAMA Network Open.*

```
table(A$Dec_Age15,useNA = "always")
A$Dec_Age15[is.na(A$Dec_Age15) & A$AGE02==15]<-A$Dec02[is.na(A$Dec_Age15)
&A$AGE02==15]
table(A$Dec_Age15,useNA = "always")
A$Dec_Age15[is.na(A$Dec_Age15) & A$AGE03==15]<-A$Dec03[is.na(A$Dec_Age15)
&A$AGE03==15]
table(A$Dec_Age15,useNA = "always")
A$Dec_Age15[is.na(A$Dec_Age15) & A$AGE04==15]<-A$Dec04[is.na(A$Dec_Age15)
&A$AGE04==15]
table(A$Dec_Age15,useNA = "always")
A$Dec_Age15[is.na(A$Dec_Age15) & A$AGE05==15]<-A$Dec05[is.na(A$Dec_Age15)
&A$AGE05==15]
table(A$Dec_Age15,useNA = "always")
A$Dec_Age15[is.na(A$Dec_Age15) & A$AGE06==15]<-A$Dec06[is.na(A$Dec_Age15)
&A$AGE06==15]
table(A$Dec_Age15,useNA = "always")
A$Dec_Age15[is.na(A$Dec_Age15) & A$AGE07==15]<-A$Dec07[is.na(A$Dec_Age15)
&A$AGE07==15]
table(A$Dec_Age15,useNA = "always")
A$Dec_Age15[is.na(A$Dec_Age15) & A$AGE08==15]<-A$Dec08[is.na(A$Dec_Age15)
&A$AGE08==15]
table(A$Dec_Age15,useNA = "always")
A$Dec_Age15[is.na(A$Dec_Age15) & A$AGE09==15]<-A$Dec09[is.na(A$Dec_Age15)
&A$AGE09==15]
table(A$Dec_Age15,useNA = "always")
A$Dec_Age15[is.na(A$Dec_Age15) & A$AGE10==15]<-A$Dec10[is.na(A$Dec_Age15)
&A$AGE10==15]
table(A$Dec_Age15,useNA = "always")
A$Dec_Age15[is.na(A$Dec_Age15) & A$AGE11==15]<-A$Dec11[is.na(A$Dec_Age15)
&A$AGE11==15]
table(A$Dec_Age15,useNA = "always")
A$Dec_Age15[is.na(A$Dec_Age15) & A$AGE12==15]<-A$Dec12[is.na(A$Dec_Age15)
&A$AGE12==15]
table(A$Dec_Age15,useNA = "always")
A$Dec_Age15[is.na(A$Dec_Age15) & A$AGE13==15]<-A$Dec13[is.na(A$Dec_Age15)
&A$AGE13==15]
table(A$Dec_Age15,useNA = "always")
A$Dec_Age15[is.na(A$Dec_Age15) & A$AGE14==15]<-A$Dec14[is.na(A$Dec_Age15)
&A$AGE14==15]
table(A$Dec_Age15,useNA = "always")
A$Dec_Age15[is.na(A$Dec_Age15) & A$AGE15==15]<-A$Dec15[is.na(A$Dec_Age15)
&A$AGE15==15]
table(A$Dec_Age15,useNA = "always")
A$Dec_Age15[is.na(A$Dec_Age15) & A$AGE16==15]<-A$Dec16[is.na(A$Dec_Age15)
&A$AGE16==15]
table(A$Dec_Age15,useNA = "always")
A$Dec_Age15[is.na(A$Dec_Age15) & A$AGE17==15]<-A$Dec17[is.na(A$Dec_Age15)
&A$AGE17==15]
table(A$Dec_Age15,useNA = "always")
### Deceased Age 16
A$Dec_Age16<-NA
A$Dec_Age16[A$Dec_Age15==1]<-1
A$Dec_Age16[is.na(A$Dec_Age16) &A$AGE98==16]<-A$Dec98[is.na(A$Dec_Age16)
&A$AGE98==16]
table(A$Dec_Age16,useNA = "always")
```

© 2023 Silver IA et al. *JAMA Network Open.*

```
A$Dec_Age16[is.na(A$Dec_Age16) &A$AGE99==16]<-A$Dec99[is.na(A$Dec_Age16)
&A$AGE99==16]
table(A$Dec_Age16,useNA = "always")
A$Dec_Age16[is.na(A$Dec_Age16) & A$AGE00==16]<-A$Dec00[is.na(A$Dec_Age16)
&A$AGE00==16]
table(A$Dec_Age16,useNA = "always")
A$Dec_Age16[is.na(A$Dec_Age16) & A$AGE01==16]<-A$Dec01[is.na(A$Dec_Age16)
&A$AGE01==16]
table(A$Dec_Age16,useNA = "always")
A$Dec_Age16[is.na(A$Dec_Age16) & A$AGE02==16]<-A$Dec02[is.na(A$Dec_Age16)
&A$AGE02==16]
table(A$Dec_Age16,useNA = "always")
A$Dec_Age16[is.na(A$Dec_Age16) & A$AGE03==16]<-A$Dec03[is.na(A$Dec_Age16)
&A$AGE03==16]
table(A$Dec_Age16,useNA = "always")
A$Dec_Age16[is.na(A$Dec_Age16) & A$AGE04==16]<-A$Dec04[is.na(A$Dec_Age16)
&A$AGE04==16]
table(A$Dec_Age16,useNA = "always")
A$Dec_Age16[is.na(A$Dec_Age16) & A$AGE05==16]<-A$Dec05[is.na(A$Dec_Age16)
&A$AGE05==16]
table(A$Dec_Age16,useNA = "always")
A$Dec_Age16[is.na(A$Dec_Age16) & A$AGE06==16]<-A$Dec06[is.na(A$Dec_Age16)
&A$AGE06==16]
table(A$Dec_Age16,useNA = "always")
A$Dec_Age16[is.na(A$Dec_Age16) & A$AGE07==16]<-A$Dec07[is.na(A$Dec_Age16)
&A$AGE07==16]
table(A$Dec_Age16,useNA = "always")
A$Dec_Age16[is.na(A$Dec_Age16) & A$AGE08==16]<-A$Dec08[is.na(A$Dec_Age16)
&A$AGE08==16]
table(A$Dec_Age16,useNA = "always")
A$Dec_Age16[is.na(A$Dec_Age16) & A$AGE09==16]<-A$Dec09[is.na(A$Dec_Age16)
&A$AGE09==16]
table(A$Dec_Age16,useNA = "always")
A$Dec_Age16[is.na(A$Dec_Age16) & A$AGE10==16]<-A$Dec10[is.na(A$Dec_Age16)
&A$AGE10==16]
table(A$Dec_Age16,useNA = "always")
A$Dec_Age16[is.na(A$Dec_Age16) & A$AGE11==16]<-A$Dec11[is.na(A$Dec_Age16)
&A$AGE11==16]
table(A$Dec_Age16,useNA = "always")
A$Dec_Age16[is.na(A$Dec_Age16) & A$AGE12==16]<-A$Dec12[is.na(A$Dec_Age16)
&A$AGE12==16]
table(A$Dec_Age16,useNA = "always")
A$Dec_Age16[is.na(A$Dec_Age16) & A$AGE13==16]<-A$Dec13[is.na(A$Dec_Age16)
&A$AGE13==16]
table(A$Dec_Age16,useNA = "always")
A$Dec_Age16[is.na(A$Dec_Age16) & A$AGE14==16]<-A$Dec14[is.na(A$Dec_Age16)
&A$AGE14==16]
table(A$Dec_Age16,useNA = "always")
A$Dec_Age16[is.na(A$Dec_Age16) & A$AGE15==16]<-A$Dec15[is.na(A$Dec_Age16)
&A$AGE15==16]
table(A$Dec_Age16,useNA = "always")
A$Dec_Age16[is.na(A$Dec_Age16) & A$AGE16==16]<-A$Dec16[is.na(A$Dec_Age16)
&A$AGE16==16]
table(A$Dec_Age16,useNA = "always")
A$Dec_Age16[is.na(A$Dec_Age16) & A$AGE17==16]<-A$Dec17[is.na(A$Dec_Age16)
&A$AGE17==16]
```

© 2023 Silver IA et al. *JAMA Network Open.*

```
table(A$Dec_Age16,useNA = "always")
### Deceased Age 17
A$Dec_Age17<-NA
A$Dec_Age17[A$Dec_Age16==1]<-1
A$Dec_Age17[is.na(A$Dec_Age17) &A$AGE98==17]<-A$Dec98[is.na(A$Dec_Age17)
&A$AGE98==17]
table(A$Dec_Age17,useNA = "always")
A$Dec_Age17[is.na(A$Dec_Age17) &A$AGE99==17]<-A$Dec99[is.na(A$Dec_Age17)
&A$AGE99==17]
table(A$Dec_Age17,useNA = "always")
A$Dec_Age17[is.na(A$Dec_Age17) & A$AGE00==17]<-A$Dec00[is.na(A$Dec_Age17)
&A$AGE00==17]
table(A$Dec_Age17,useNA = "always")
A$Dec_Age17[is.na(A$Dec_Age17) & A$AGE01==17]<-A$Dec01[is.na(A$Dec_Age17)
&A$AGE01==17]
table(A$Dec_Age17,useNA = "always")
A$Dec_Age17[is.na(A$Dec_Age17) & A$AGE02==17]<-A$Dec02[is.na(A$Dec_Age17)
&A$AGE02==17]
table(A$Dec_Age17,useNA = "always")
A$Dec_Age17[is.na(A$Dec_Age17) & A$AGE03==17]<-A$Dec03[is.na(A$Dec_Age17)
&A$AGE03==17]
table(A$Dec_Age17,useNA = "always")
A$Dec_Age17[is.na(A$Dec_Age17) & A$AGE04==17]<-A$Dec04[is.na(A$Dec_Age17)
&A$AGE04==17]
table(A$Dec_Age17,useNA = "always")
A$Dec_Age17[is.na(A$Dec_Age17) & A$AGE05==17]<-A$Dec05[is.na(A$Dec_Age17)
&A$AGE05==17]
table(A$Dec_Age17,useNA = "always")
A$Dec_Age17[is.na(A$Dec_Age17) & A$AGE06==17]<-A$Dec06[is.na(A$Dec_Age17)
&A$AGE06==17]
table(A$Dec_Age17,useNA = "always")
A$Dec_Age17[is.na(A$Dec_Age17) & A$AGE07==17]<-A$Dec07[is.na(A$Dec_Age17)
&A$AGE07==17]
table(A$Dec_Age17,useNA = "always")
A$Dec_Age17[is.na(A$Dec_Age17) & A$AGE08==17]<-A$Dec08[is.na(A$Dec_Age17)
&A$AGE08==17]
table(A$Dec_Age17,useNA = "always")
A$Dec_Age17[is.na(A$Dec_Age17) & A$AGE09==17]<-A$Dec09[is.na(A$Dec_Age17)
&A$AGE09==17]
table(A$Dec_Age17,useNA = "always")
A$Dec_Age17[is.na(A$Dec_Age17) & A$AGE10==17]<-A$Dec10[is.na(A$Dec_Age17)
&A$AGE10==17]
table(A$Dec_Age17,useNA = "always")
A$Dec_Age17[is.na(A$Dec_Age17) & A$AGE11==17]<-A$Dec11[is.na(A$Dec_Age17)
&A$AGE11==17]
table(A$Dec_Age17,useNA = "always")
A$Dec_Age17[is.na(A$Dec_Age17) & A$AGE12==17]<-A$Dec12[is.na(A$Dec_Age17)
&A$AGE12==17]
table(A$Dec_Age17,useNA = "always")
A$Dec_Age17[is.na(A$Dec_Age17) & A$AGE13==17]<-A$Dec13[is.na(A$Dec_Age17)
&A$AGE13==17]
table(A$Dec_Age17,useNA = "always")
A$Dec_Age17[is.na(A$Dec_Age17) & A$AGE14==17]<-A$Dec14[is.na(A$Dec_Age17)
&A$AGE14==17]
table(A$Dec_Age17,useNA = "always")
```

© 2023 Silver IA et al. *JAMA Network Open.*

```
A$Dec_Age17[is.na(A$Dec_Age17) & A$AGE15==17]<-A$Dec15[is.na(A$Dec_Age17)
&A$AGE15==17]
table(A$Dec_Age17,useNA = "always")
A$Dec_Age17[is.na(A$Dec_Age17) & A$AGE16==17]<-A$Dec16[is.na(A$Dec_Age17)
&A$AGE16==17]
table(A$Dec_Age17,useNA = "always")
A$Dec_Age17[is.na(A$Dec_Age17) & A$AGE17==17]<-A$Dec17[is.na(A$Dec_Age17)
&A$AGE17==17]
table(A$Dec_Age17,useNA = "always")
### Deceased Age 18
A$Dec_Age18<-NA
A$Dec_Age18[A$Dec_Age17==1]<-1
A$Dec_Age18[is.na(A$Dec_Age18) &A$AGE98==18]<-A$Dec98[is.na(A$Dec_Age18)
&A$AGE98==18]
table(A$Dec_Age18,useNA = "always")
A$Dec_Age18[is.na(A$Dec_Age18) &A$AGE99==18]<-A$Dec99[is.na(A$Dec_Age18)
&A$AGE99==18]
table(A$Dec_Age18,useNA = "always")
A$Dec_Age18[is.na(A$Dec_Age18) & A$AGE00==18]<-A$Dec00[is.na(A$Dec_Age18)
&A$AGE00==18]
table(A$Dec_Age18,useNA = "always")
A$Dec_Age18[is.na(A$Dec_Age18) & A$AGE01==18]<-A$Dec01[is.na(A$Dec_Age18)
&A$AGE01==18]
table(A$Dec_Age18,useNA = "always")
A$Dec_Age18[is.na(A$Dec_Age18) & A$AGE02==18]<-A$Dec02[is.na(A$Dec_Age18)
&A$AGE02==18]
table(A$Dec_Age18,useNA = "always")
A$Dec_Age18[is.na(A$Dec_Age18) & A$AGE03==18]<-A$Dec03[is.na(A$Dec_Age18)
&A$AGE03==18]
table(A$Dec_Age18,useNA = "always")
A$Dec_Age18[is.na(A$Dec_Age18) & A$AGE04==18]<-A$Dec04[is.na(A$Dec_Age18)
&A$AGE04==18]
table(A$Dec_Age18,useNA = "always")
A$Dec_Age18[is.na(A$Dec_Age18) & A$AGE05==18]<-A$Dec05[is.na(A$Dec_Age18)
&A$AGE05==18]
table(A$Dec_Age18,useNA = "always")
A$Dec_Age18[is.na(A$Dec_Age18) & A$AGE06==18]<-A$Dec06[is.na(A$Dec_Age18)
&A$AGE06==18]
table(A$Dec_Age18,useNA = "always")
A$Dec_Age18[is.na(A$Dec_Age18) & A$AGE07==18]<-A$Dec07[is.na(A$Dec_Age18)
&A$AGE07==18]
table(A$Dec_Age18,useNA = "always")
A$Dec_Age18[is.na(A$Dec_Age18) & A$AGE08==18]<-A$Dec08[is.na(A$Dec_Age18)
&A$AGE08==18]
table(A$Dec_Age18,useNA = "always")
A$Dec_Age18[is.na(A$Dec_Age18) & A$AGE09==18]<-A$Dec09[is.na(A$Dec_Age18)
&A$AGE09==18]
table(A$Dec_Age18,useNA = "always")
A$Dec_Age18[is.na(A$Dec_Age18) & A$AGE10==18]<-A$Dec10[is.na(A$Dec_Age18)
&A$AGE10==18]
table(A$Dec_Age18,useNA = "always")
A$Dec_Age18[is.na(A$Dec_Age18) & A$AGE11==18]<-A$Dec11[is.na(A$Dec_Age18)
&A$AGE11==18]
table(A$Dec_Age18,useNA = "always")
A$Dec_Age18[is.na(A$Dec_Age18) & A$AGE12==18]<-A$Dec12[is.na(A$Dec_Age18)
&A$AGE12==18]
```

© 2023 Silver IA et al. *JAMA Network Open.*

```
table(A$Dec_Age18,useNA = "always")
A$Dec_Age18[is.na(A$Dec_Age18) & A$AGE13==18]<-A$Dec13[is.na(A$Dec_Age18)
&A$AGE13==18]
table(A$Dec_Age18,useNA = "always")
A$Dec_Age18[is.na(A$Dec_Age18) & A$AGE14==18]<-A$Dec14[is.na(A$Dec_Age18)
&A$AGE14==18]
table(A$Dec_Age18,useNA = "always")
A$Dec_Age18[is.na(A$Dec_Age18) & A$AGE15==18]<-A$Dec15[is.na(A$Dec_Age18)
&A$AGE15==18]
table(A$Dec_Age18,useNA = "always")
A$Dec_Age18[is.na(A$Dec_Age18) & A$AGE16==18]<-A$Dec16[is.na(A$Dec_Age18)
&A$AGE16==18]
table(A$Dec_Age18,useNA = "always")
A$Dec_Age18[is.na(A$Dec_Age18) & A$AGE17==18]<-A$Dec17[is.na(A$Dec_Age18)
&A$AGE17==18]
table(A$Dec_Age18,useNA = "always")
### Deceased Age 19
A$Dec_Age19<-NA
A$Dec_Age19[A$Dec_Age18==1]<-1
A$Dec_Age19[is.na(A$Dec_Age19) &A$AGE98==19]<-A$Dec98[is.na(A$Dec_Age19)
&A$AGE98==19]
table(A$Dec_Age19,useNA = "always")
A$Dec_Age19[is.na(A$Dec_Age19) &A$AGE99==19]<-A$Dec99[is.na(A$Dec_Age19)
&A$AGE99==19]
table(A$Dec_Age19,useNA = "always")
A$Dec_Age19[is.na(A$Dec_Age19) & A$AGE00==19]<-A$Dec00[is.na(A$Dec_Age19)
&A$AGE00==19]
table(A$Dec_Age19,useNA = "always")
A$Dec_Age19[is.na(A$Dec_Age19) & A$AGE01==19]<-A$Dec01[is.na(A$Dec_Age19)
&A$AGE01==19]
table(A$Dec_Age19,useNA = "always")
A$Dec_Age19[is.na(A$Dec_Age19) & A$AGE02==19]<-A$Dec02[is.na(A$Dec_Age19)
&A$AGE02==19]
table(A$Dec_Age19,useNA = "always")
A$Dec_Age19[is.na(A$Dec_Age19) & A$AGE03==19]<-A$Dec03[is.na(A$Dec_Age19)
&A$AGE03==19]
table(A$Dec_Age19,useNA = "always")
A$Dec_Age19[is.na(A$Dec_Age19) & A$AGE04==19]<-A$Dec04[is.na(A$Dec_Age19)
&A$AGE04==19]
table(A$Dec_Age19,useNA = "always")
A$Dec_Age19[is.na(A$Dec_Age19) & A$AGE05==19]<-A$Dec05[is.na(A$Dec_Age19)
&A$AGE05==19]
table(A$Dec_Age19,useNA = "always")
A$Dec_Age19[is.na(A$Dec_Age19) & A$AGE06==19]<-A$Dec06[is.na(A$Dec_Age19)
&A$AGE06==19]
table(A$Dec_Age19,useNA = "always")
A$Dec_Age19[is.na(A$Dec_Age19) & A$AGE07==19]<-A$Dec07[is.na(A$Dec_Age19)
&A$AGE07==19]
table(A$Dec_Age19,useNA = "always")
A$Dec_Age19[is.na(A$Dec_Age19) & A$AGE08==19]<-A$Dec08[is.na(A$Dec_Age19)
&A$AGE08==19]
table(A$Dec_Age19,useNA = "always")
A$Dec_Age19[is.na(A$Dec_Age19) & A$AGE09==19]<-A$Dec09[is.na(A$Dec_Age19)
&A$AGE09==19]
table(A$Dec_Age19,useNA = "always")
```

© 2023 Silver IA et al. *JAMA Network Open.*

```
A$Dec_Age19[is.na(A$Dec_Age19) & A$AGE10==19]<-A$Dec10[is.na(A$Dec_Age19)
&A$AGE10==19]
table(A$Dec_Age19,useNA = "always")
A$Dec_Age19[is.na(A$Dec_Age19) & A$AGE11==19]<-A$Dec11[is.na(A$Dec_Age19)
&A$AGE11==19]
table(A$Dec_Age19,useNA = "always")
A$Dec_Age19[is.na(A$Dec_Age19) & A$AGE12==19]<-A$Dec12[is.na(A$Dec_Age19)
&A$AGE12==19]
table(A$Dec_Age19,useNA = "always")
A$Dec_Age19[is.na(A$Dec_Age19) & A$AGE13==19]<-A$Dec13[is.na(A$Dec_Age19)
&A$AGE13==19]
table(A$Dec_Age19,useNA = "always")
A$Dec_Age19[is.na(A$Dec_Age19) & A$AGE14==19]<-A$Dec14[is.na(A$Dec_Age19)
&A$AGE14==19]
table(A$Dec_Age19,useNA = "always")
A$Dec_Age19[is.na(A$Dec_Age19) & A$AGE15==19]<-A$Dec15[is.na(A$Dec_Age19)
&A$AGE15==19]
table(A$Dec_Age19,useNA = "always")
A$Dec_Age19[is.na(A$Dec_Age19) & A$AGE16==19]<-A$Dec16[is.na(A$Dec_Age19)
&A$AGE16==19]
table(A$Dec_Age19,useNA = "always")
A$Dec_Age19[is.na(A$Dec_Age19) & A$AGE17==19]<-A$Dec17[is.na(A$Dec_Age19)
&A$AGE17==19]
table(A$Dec_Age19,useNA = "always")
### Deceased Age 20
A$Dec_Age20<-NA
A$Dec_Age20[A$Dec_Age19==1]<-1
A$Dec_Age20[is.na(A$Dec_Age20) &A$AGE98==20]<-A$Dec98[is.na(A$Dec_Age20)
&A$AGE98==20]
table(A$Dec_Age20,useNA = "always")
A$Dec_Age20[is.na(A$Dec_Age20) &A$AGE99==20]<-A$Dec99[is.na(A$Dec_Age20)
&A$AGE99==20]
table(A$Dec_Age20,useNA = "always")
A$Dec_Age20[is.na(A$Dec_Age20) & A$AGE00==20]<-A$Dec00[is.na(A$Dec_Age20)
&A$AGE00==20]
table(A$Dec_Age20,useNA = "always")
A$Dec_Age20[is.na(A$Dec_Age20) & A$AGE01==20]<-A$Dec01[is.na(A$Dec_Age20)
&A$AGE01==20]
table(A$Dec_Age20,useNA = "always")
A$Dec_Age20[is.na(A$Dec_Age20) & A$AGE02==20]<-A$Dec02[is.na(A$Dec_Age20)
&A$AGE02==20]
table(A$Dec_Age20,useNA = "always")
A$Dec_Age20[is.na(A$Dec_Age20) & A$AGE03==20]<-A$Dec03[is.na(A$Dec_Age20)
&A$AGE03==20]
table(A$Dec_Age20,useNA = "always")
A$Dec_Age20[is.na(A$Dec_Age20) & A$AGE04==20]<-A$Dec04[is.na(A$Dec_Age20)
&A$AGE04==20]
table(A$Dec_Age20,useNA = "always")
A$Dec_Age20[is.na(A$Dec_Age20) & A$AGE05==20]<-A$Dec05[is.na(A$Dec_Age20)
&A$AGE05==20]
table(A$Dec_Age20,useNA = "always")
A$Dec_Age20[is.na(A$Dec_Age20) & A$AGE06==20]<-A$Dec06[is.na(A$Dec_Age20)
&A$AGE06==20]
table(A$Dec_Age20,useNA = "always")
A$Dec_Age20[is.na(A$Dec_Age20) & A$AGE07==20]<-A$Dec07[is.na(A$Dec_Age20)
&A$AGE07==20]
```

© 2023 Silver IA et al. *JAMA Network Open.*

```
table(A$Dec_Age20,useNA = "always")
A$Dec_Age20[is.na(A$Dec_Age20) & A$AGE08==20]<-A$Dec08[is.na(A$Dec_Age20)
&A$AGE08==20]
table(A$Dec_Age20,useNA = "always")
A$Dec_Age20[is.na(A$Dec_Age20) & A$AGE09==20]<-A$Dec09[is.na(A$Dec_Age20)
&A$AGE09==20]
table(A$Dec_Age20,useNA = "always")
A$Dec_Age20[is.na(A$Dec_Age20) & A$AGE10==20]<-A$Dec10[is.na(A$Dec_Age20)
&A$AGE10==20]
table(A$Dec_Age20,useNA = "always")
A$Dec_Age20[is.na(A$Dec_Age20) & A$AGE11==20]<-A$Dec11[is.na(A$Dec_Age20)
&A$AGE11==20]
table(A$Dec_Age20,useNA = "always")
A$Dec_Age20[is.na(A$Dec_Age20) & A$AGE12==20]<-A$Dec12[is.na(A$Dec_Age20)
&A$AGE12==20]
table(A$Dec_Age20,useNA = "always")
A$Dec_Age20[is.na(A$Dec_Age20) & A$AGE13==20]<-A$Dec13[is.na(A$Dec_Age20)
&A$AGE13==20]
table(A$Dec_Age20,useNA = "always")
A$Dec_Age20[is.na(A$Dec_Age20) & A$AGE14==20]<-A$Dec14[is.na(A$Dec_Age20)
&A$AGE14==20]
table(A$Dec_Age20,useNA = "always")
A$Dec_Age20[is.na(A$Dec_Age20) & A$AGE15==20]<-A$Dec15[is.na(A$Dec_Age20)
&A$AGE15==20]
table(A$Dec_Age20,useNA = "always")
A$Dec_Age20[is.na(A$Dec_Age20) & A$AGE16==20]<-A$Dec16[is.na(A$Dec_Age20)
&A$AGE16==20]
table(A$Dec_Age20,useNA = "always")
A$Dec_Age20[is.na(A$Dec_Age20) & A$AGE17==20]<-A$Dec17[is.na(A$Dec_Age20)
&A$AGE17==20]
table(A$Dec_Age20,useNA = "always")
### Deceased Age 21
A$Dec_Age21<-NA
A$Dec_Age21[A$Dec_Age20==1]<-1
A$Dec_Age21[is.na(A$Dec_Age21) &A$AGE98==21]<-A$Dec98[is.na(A$Dec_Age21)
&A$AGE98==21]
table(A$Dec_Age21,useNA = "always")
A$Dec_Age21[is.na(A$Dec_Age21) &A$AGE99==21]<-A$Dec99[is.na(A$Dec_Age21)
&A$AGE99==21]
table(A$Dec_Age21,useNA = "always")
A$Dec_Age21[is.na(A$Dec_Age21) & A$AGE00==21]<-A$Dec00[is.na(A$Dec_Age21)
&A$AGE00==21]
table(A$Dec_Age21,useNA = "always")
A$Dec_Age21[is.na(A$Dec_Age21) & A$AGE01==21]<-A$Dec01[is.na(A$Dec_Age21)
&A$AGE01==21]
table(A$Dec_Age21,useNA = "always")
A$Dec_Age21[is.na(A$Dec_Age21) & A$AGE02==21]<-A$Dec02[is.na(A$Dec_Age21)
&A$AGE02==21]
table(A$Dec_Age21,useNA = "always")
A$Dec_Age21[is.na(A$Dec_Age21) & A$AGE03==21]<-A$Dec03[is.na(A$Dec_Age21)
&A$AGE03==21]
table(A$Dec_Age21,useNA = "always")
A$Dec_Age21[is.na(A$Dec_Age21) & A$AGE04==21]<-A$Dec04[is.na(A$Dec_Age21)
&A$AGE04==21]
table(A$Dec_Age21,useNA = "always")
```

© 2023 Silver IA et al. *JAMA Network Open.*

```
A$Dec_Age21[is.na(A$Dec_Age21) & A$AGE05==21]<-A$Dec05[is.na(A$Dec_Age21)
&A$AGE05==21]
table(A$Dec_Age21,useNA = "always")
A$Dec_Age21[is.na(A$Dec_Age21) & A$AGE06==21]<-A$Dec06[is.na(A$Dec_Age21)
&A$AGE06==21]
table(A$Dec_Age21,useNA = "always")
A$Dec_Age21[is.na(A$Dec_Age21) & A$AGE07==21]<-A$Dec07[is.na(A$Dec_Age21)
&A$AGE07==21]
table(A$Dec_Age21,useNA = "always")
A$Dec_Age21[is.na(A$Dec_Age21) & A$AGE08==21]<-A$Dec08[is.na(A$Dec_Age21)
&A$AGE08==21]
table(A$Dec_Age21,useNA = "always")
A$Dec_Age21[is.na(A$Dec_Age21) & A$AGE09==21]<-A$Dec09[is.na(A$Dec_Age21)
&A$AGE09==21]
table(A$Dec_Age21,useNA = "always")
A$Dec_Age21[is.na(A$Dec_Age21) & A$AGE10==21]<-A$Dec10[is.na(A$Dec_Age21)
&A$AGE10==21]
table(A$Dec_Age21,useNA = "always")
A$Dec_Age21[is.na(A$Dec_Age21) & A$AGE11==21]<-A$Dec11[is.na(A$Dec_Age21)
&A$AGE11==21]
table(A$Dec_Age21,useNA = "always")
A$Dec_Age21[is.na(A$Dec_Age21) & A$AGE12==21]<-A$Dec12[is.na(A$Dec_Age21)
&A$AGE12==21]
table(A$Dec_Age21,useNA = "always")
A$Dec_Age21[is.na(A$Dec_Age21) & A$AGE13==21]<-A$Dec13[is.na(A$Dec_Age21)
&A$AGE13==21]
table(A$Dec_Age21,useNA = "always")
A$Dec_Age21[is.na(A$Dec_Age21) & A$AGE14==21]<-A$Dec14[is.na(A$Dec_Age21)
&A$AGE14==21]
table(A$Dec_Age21,useNA = "always")
A$Dec_Age21[is.na(A$Dec_Age21) & A$AGE15==21]<-A$Dec15[is.na(A$Dec_Age21)
&A$AGE15==21]
table(A$Dec_Age21,useNA = "always")
A$Dec_Age21[is.na(A$Dec_Age21) & A$AGE16==21]<-A$Dec16[is.na(A$Dec_Age21)
&A$AGE16==21]
table(A$Dec_Age21,useNA = "always")
A$Dec_Age21[is.na(A$Dec_Age21) & A$AGE17==21]<-A$Dec17[is.na(A$Dec_Age21)
&A$AGE17==21]
table(A$Dec_Age21,useNA = "always")
### Deceased Age 22
A$Dec_Age22<-NA
A$Dec_Age22[A$Dec_Age21==1]<-1
A$Dec_Age22[is.na(A$Dec_Age22) &A$AGE98==22]<-A$Dec98[is.na(A$Dec_Age22)
&A$AGE98==22]
table(A$Dec_Age22,useNA = "always")
A$Dec_Age22[is.na(A$Dec_Age22) &A$AGE99==22]<-A$Dec99[is.na(A$Dec_Age22)
&A$AGE99==22]
table(A$Dec_Age22,useNA = "always")
A$Dec_Age22[is.na(A$Dec_Age22) & A$AGE00==22]<-A$Dec00[is.na(A$Dec_Age22)
&A$AGE00==22]
table(A$Dec_Age22,useNA = "always")
A$Dec_Age22[is.na(A$Dec_Age22) & A$AGE01==22]<-A$Dec01[is.na(A$Dec_Age22)
&A$AGE01==22]
table(A$Dec_Age22,useNA = "always")
A$Dec_Age22[is.na(A$Dec_Age22) & A$AGE02==22]<-A$Dec02[is.na(A$Dec_Age22)
&A$AGE02==22]
```

© 2023 Silver IA et al. *JAMA Network Open.*

```
table(A$Dec_Age22,useNA = "always")
A$Dec_Age22[is.na(A$Dec_Age22) & A$AGE03==22]<-A$Dec03[is.na(A$Dec_Age22)
&A$AGE03==22]
table(A$Dec_Age22,useNA = "always")
A$Dec_Age22[is.na(A$Dec_Age22) & A$AGE04==22]<-A$Dec04[is.na(A$Dec_Age22)
&A$AGE04==22]
table(A$Dec_Age22,useNA = "always")
A$Dec_Age22[is.na(A$Dec_Age22) & A$AGE05==22]<-A$Dec05[is.na(A$Dec_Age22)
&A$AGE05==22]
table(A$Dec_Age22,useNA = "always")
A$Dec_Age22[is.na(A$Dec_Age22) & A$AGE06==22]<-A$Dec06[is.na(A$Dec_Age22)
&A$AGE06==22]
table(A$Dec_Age22,useNA = "always")
A$Dec_Age22[is.na(A$Dec_Age22) & A$AGE07==22]<-A$Dec07[is.na(A$Dec_Age22)
&A$AGE07==22]
table(A$Dec_Age22,useNA = "always")
A$Dec_Age22[is.na(A$Dec_Age22) & A$AGE08==22]<-A$Dec08[is.na(A$Dec_Age22)
&A$AGE08==22]
table(A$Dec_Age22,useNA = "always")
A$Dec_Age22[is.na(A$Dec_Age22) & A$AGE09==22]<-A$Dec09[is.na(A$Dec_Age22)
&A$AGE09==22]
table(A$Dec_Age22,useNA = "always")
A$Dec_Age22[is.na(A$Dec_Age22) & A$AGE10==22]<-A$Dec10[is.na(A$Dec_Age22)
&A$AGE10==22]
table(A$Dec_Age22,useNA = "always")
A$Dec_Age22[is.na(A$Dec_Age22) & A$AGE11==22]<-A$Dec11[is.na(A$Dec_Age22)
&A$AGE11==22]
table(A$Dec_Age22,useNA = "always")
A$Dec_Age22[is.na(A$Dec_Age22) & A$AGE12==22]<-A$Dec12[is.na(A$Dec_Age22)
&A$AGE12==22]
table(A$Dec_Age22,useNA = "always")
A$Dec_Age22[is.na(A$Dec_Age22) & A$AGE13==22]<-A$Dec13[is.na(A$Dec_Age22)
&A$AGE13==22]
table(A$Dec_Age22,useNA = "always")
A$Dec_Age22[is.na(A$Dec_Age22) & A$AGE14==22]<-A$Dec14[is.na(A$Dec_Age22)
&A$AGE14==22]
table(A$Dec_Age22,useNA = "always")
A$Dec_Age22[is.na(A$Dec_Age22) & A$AGE15==22]<-A$Dec15[is.na(A$Dec_Age22)
&A$AGE15==22]
table(A$Dec_Age22,useNA = "always")
A$Dec_Age22[is.na(A$Dec_Age22) & A$AGE16==22]<-A$Dec16[is.na(A$Dec_Age22)
&A$AGE16==22]
table(A$Dec_Age22,useNA = "always")
A$Dec_Age22[is.na(A$Dec_Age22) & A$AGE17==22]<-A$Dec17[is.na(A$Dec_Age22)
&A$AGE17==22]
table(A$Dec_Age22,useNA = "always")
### Deceased Age 23
A$Dec_Age23<-NA
A$Dec_Age23[A$Dec_Age22==1]<-1
A$Dec_Age23[is.na(A$Dec_Age23) &A$AGE98==23]<-A$Dec98[is.na(A$Dec_Age23)
&A$AGE98==23]
table(A$Dec_Age23,useNA = "always")
A$Dec_Age23[is.na(A$Dec_Age23) &A$AGE99==23]<-A$Dec99[is.na(A$Dec_Age23)
&A$AGE99==23]
table(A$Dec_Age23,useNA = "always")
```

© 2023 Silver IA et al. *JAMA Network Open.*

```
A$Dec_Age23[is.na(A$Dec_Age23) & A$AGE00==23]<-A$Dec00[is.na(A$Dec_Age23)
&A$AGE00==23]
table(A$Dec_Age23,useNA = "always")
A$Dec_Age23[is.na(A$Dec_Age23) & A$AGE01==23]<-A$Dec01[is.na(A$Dec_Age23)
&A$AGE01==23]
table(A$Dec_Age23,useNA = "always")
A$Dec_Age23[is.na(A$Dec_Age23) & A$AGE02==23]<-A$Dec02[is.na(A$Dec_Age23)
&A$AGE02==23]
table(A$Dec_Age23,useNA = "always")
A$Dec_Age23[is.na(A$Dec_Age23) & A$AGE03==23]<-A$Dec03[is.na(A$Dec_Age23)
&A$AGE03==23]
table(A$Dec_Age23,useNA = "always")
A$Dec_Age23[is.na(A$Dec_Age23) & A$AGE04==23]<-A$Dec04[is.na(A$Dec_Age23)
&A$AGE04==23]
table(A$Dec_Age23,useNA = "always")
A$Dec_Age23[is.na(A$Dec_Age23) & A$AGE05==23]<-A$Dec05[is.na(A$Dec_Age23)
&A$AGE05==23]
table(A$Dec_Age23,useNA = "always")
A$Dec_Age23[is.na(A$Dec_Age23) & A$AGE06==23]<-A$Dec06[is.na(A$Dec_Age23)
&A$AGE06==23]
table(A$Dec_Age23,useNA = "always")
A$Dec_Age23[is.na(A$Dec_Age23) & A$AGE07==23]<-A$Dec07[is.na(A$Dec_Age23)
&A$AGE07==23]
table(A$Dec_Age23,useNA = "always")
A$Dec_Age23[is.na(A$Dec_Age23) & A$AGE08==23]<-A$Dec08[is.na(A$Dec_Age23)
&A$AGE08==23]
table(A$Dec_Age23,useNA = "always")
A$Dec_Age23[is.na(A$Dec_Age23) & A$AGE09==23]<-A$Dec09[is.na(A$Dec_Age23)
&A$AGE09==23]
table(A$Dec_Age23,useNA = "always")
A$Dec_Age23[is.na(A$Dec_Age23) & A$AGE10==23]<-A$Dec10[is.na(A$Dec_Age23)
&A$AGE10==23]
table(A$Dec_Age23,useNA = "always")
A$Dec_Age23[is.na(A$Dec_Age23) & A$AGE11==23]<-A$Dec11[is.na(A$Dec_Age23)
&A$AGE11==23]
table(A$Dec_Age23,useNA = "always")
A$Dec_Age23[is.na(A$Dec_Age23) & A$AGE12==23]<-A$Dec12[is.na(A$Dec_Age23)
&A$AGE12==23]
table(A$Dec_Age23,useNA = "always")
A$Dec_Age23[is.na(A$Dec_Age23) & A$AGE13==23]<-A$Dec13[is.na(A$Dec_Age23)
&A$AGE13==23]
table(A$Dec_Age23,useNA = "always")
A$Dec_Age23[is.na(A$Dec_Age23) & A$AGE14==23]<-A$Dec14[is.na(A$Dec_Age23)
&A$AGE14==23]
table(A$Dec_Age23,useNA = "always")
A$Dec_Age23[is.na(A$Dec_Age23) & A$AGE15==23]<-A$Dec15[is.na(A$Dec_Age23)
&A$AGE15==23]
table(A$Dec_Age23,useNA = "always")
A$Dec_Age23[is.na(A$Dec_Age23) & A$AGE16==23]<-A$Dec16[is.na(A$Dec_Age23)
&A$AGE16==23]
table(A$Dec_Age23,useNA = "always")
A$Dec_Age23[is.na(A$Dec_Age23) & A$AGE17==23]<-A$Dec17[is.na(A$Dec_Age23)
&A$AGE17==23]
table(A$Dec_Age23,useNA = "always")
### Deceased Age 24
A$Dec_Age24<-NA
```

© 2023 Silver IA et al. *JAMA Network Open.*

```
A$Dec_Age24[A$Dec_Age23==1]<-1
A$Dec_Age24[is.na(A$Dec_Age24) &A$AGE98==24]<-A$Dec98[is.na(A$Dec_Age24)
&A$AGE98==24]
table(A$Dec_Age24,useNA = "always")
A$Dec_Age24[is.na(A$Dec_Age24) &A$AGE99==24]<-A$Dec99[is.na(A$Dec_Age24)
&A$AGE99==24]
table(A$Dec_Age24,useNA = "always")
A$Dec_Age24[is.na(A$Dec_Age24) & A$AGE00==24]<-A$Dec00[is.na(A$Dec_Age24)
&A$AGE00==24]
table(A$Dec_Age24,useNA = "always")
A$Dec_Age24[is.na(A$Dec_Age24) & A$AGE01==24]<-A$Dec01[is.na(A$Dec_Age24)
&A$AGE01==24]
table(A$Dec_Age24,useNA = "always")
A$Dec_Age24[is.na(A$Dec_Age24) & A$AGE02==24]<-A$Dec02[is.na(A$Dec_Age24)
&A$AGE02==24]
table(A$Dec_Age24,useNA = "always")
A$Dec_Age24[is.na(A$Dec_Age24) & A$AGE03==24]<-A$Dec03[is.na(A$Dec_Age24)
&A$AGE03==24]
table(A$Dec_Age24,useNA = "always")
A$Dec_Age24[is.na(A$Dec_Age24) & A$AGE04==24]<-A$Dec04[is.na(A$Dec_Age24)
&A$AGE04==24]
table(A$Dec_Age24,useNA = "always")
A$Dec_Age24[is.na(A$Dec_Age24) & A$AGE05==24]<-A$Dec05[is.na(A$Dec_Age24)
&A$AGE05==24]
table(A$Dec_Age24,useNA = "always")
A$Dec_Age24[is.na(A$Dec_Age24) & A$AGE06==24]<-A$Dec06[is.na(A$Dec_Age24)
&A$AGE06==24]
table(A$Dec_Age24,useNA = "always")
A$Dec_Age24[is.na(A$Dec_Age24) & A$AGE07==24]<-A$Dec07[is.na(A$Dec_Age24)
&A$AGE07==24]
table(A$Dec_Age24,useNA = "always")
A$Dec_Age24[is.na(A$Dec_Age24) & A$AGE08==24]<-A$Dec08[is.na(A$Dec_Age24)
&A$AGE08==24]
table(A$Dec_Age24,useNA = "always")
A$Dec_Age24[is.na(A$Dec_Age24) & A$AGE09==24]<-A$Dec09[is.na(A$Dec_Age24)
&A$AGE09==24]
table(A$Dec_Age24,useNA = "always")
A$Dec_Age24[is.na(A$Dec_Age24) & A$AGE10==24]<-A$Dec10[is.na(A$Dec_Age24)
&A$AGE10==24]
table(A$Dec_Age24,useNA = "always")
A$Dec_Age24[is.na(A$Dec_Age24) & A$AGE11==24]<-A$Dec11[is.na(A$Dec_Age24)
&A$AGE11==24]
table(A$Dec_Age24,useNA = "always")
A$Dec_Age24[is.na(A$Dec_Age24) & A$AGE12==24]<-A$Dec12[is.na(A$Dec_Age24)
&A$AGE12==24]
table(A$Dec_Age24,useNA = "always")
A$Dec_Age24[is.na(A$Dec_Age24) & A$AGE13==24]<-A$Dec13[is.na(A$Dec_Age24)
&A$AGE13==24]
table(A$Dec_Age24,useNA = "always")
A$Dec_Age24[is.na(A$Dec_Age24) & A$AGE14==24]<-A$Dec14[is.na(A$Dec_Age24)
&A$AGE14==24]
table(A$Dec_Age24,useNA = "always")
A$Dec_Age24[is.na(A$Dec_Age24) & A$AGE15==24]<-A$Dec15[is.na(A$Dec_Age24)
&A$AGE15==24]
table(A$Dec_Age24,useNA = "always")
```

© 2023 Silver IA et al. *JAMA Network Open.*

```
A$Dec_Age24[is.na(A$Dec_Age24) & A$AGE16==24]<-A$Dec16[is.na(A$Dec_Age24)
&A$AGE16==24]
table(A$Dec_Age24,useNA = "always")
A$Dec_Age24[is.na(A$Dec_Age24) & A$AGE17==24]<-A$Dec17[is.na(A$Dec_Age24)
&A$AGE17==24]
table(A$Dec_Age24,useNA = "always")
### Deceased Age 25
A$Dec_Age25<-NA
A$Dec_Age25[A$Dec_Age24==1]<-1
A$Dec_Age25[is.na(A$Dec_Age25) &A$AGE98==25]<-A$Dec98[is.na(A$Dec_Age25)
&A$AGE98==25]
table(A$Dec_Age25,useNA = "always")
A$Dec_Age25[is.na(A$Dec_Age25) &A$AGE99==25]<-A$Dec99[is.na(A$Dec_Age25)
&A$AGE99==25]
table(A$Dec_Age25,useNA = "always")
A$Dec_Age25[is.na(A$Dec_Age25) & A$AGE00==25]<-A$Dec00[is.na(A$Dec_Age25)
&A$AGE00==25]
table(A$Dec_Age25,useNA = "always")
A$Dec_Age25[is.na(A$Dec_Age25) & A$AGE01==25]<-A$Dec01[is.na(A$Dec_Age25)
&A$AGE01==25]
table(A$Dec_Age25,useNA = "always")
A$Dec_Age25[is.na(A$Dec_Age25) & A$AGE02==25]<-A$Dec02[is.na(A$Dec_Age25)
&A$AGE02==25]
table(A$Dec_Age25,useNA = "always")
A$Dec_Age25[is.na(A$Dec_Age25) & A$AGE03==25]<-A$Dec03[is.na(A$Dec_Age25)
&A$AGE03==25]
table(A$Dec_Age25,useNA = "always")
A$Dec_Age25[is.na(A$Dec_Age25) & A$AGE04==25]<-A$Dec04[is.na(A$Dec_Age25)
&A$AGE04==25]
table(A$Dec_Age25,useNA = "always")
A$Dec_Age25[is.na(A$Dec_Age25) & A$AGE05==25]<-A$Dec05[is.na(A$Dec_Age25)
&A$AGE05==25]
table(A$Dec_Age25,useNA = "always")
A$Dec_Age25[is.na(A$Dec_Age25) & A$AGE06==25]<-A$Dec06[is.na(A$Dec_Age25)
&A$AGE06==25]
table(A$Dec_Age25,useNA = "always")
A$Dec_Age25[is.na(A$Dec_Age25) & A$AGE07==25]<-A$Dec07[is.na(A$Dec_Age25)
&A$AGE07==25]
table(A$Dec_Age25,useNA = "always")
A$Dec_Age25[is.na(A$Dec_Age25) & A$AGE08==25]<-A$Dec08[is.na(A$Dec_Age25)
&A$AGE08==25]
table(A$Dec_Age25,useNA = "always")
A$Dec_Age25[is.na(A$Dec_Age25) & A$AGE09==25]<-A$Dec09[is.na(A$Dec_Age25)
&A$AGE09==25]
table(A$Dec_Age25,useNA = "always")
A$Dec_Age25[is.na(A$Dec_Age25) & A$AGE10==25]<-A$Dec10[is.na(A$Dec_Age25)
&A$AGE10==25]
table(A$Dec_Age25,useNA = "always")
A$Dec_Age25[is.na(A$Dec_Age25) & A$AGE11==25]<-A$Dec11[is.na(A$Dec_Age25)
&A$AGE11==25]
table(A$Dec_Age25,useNA = "always")
A$Dec_Age25[is.na(A$Dec_Age25) & A$AGE12==25]<-A$Dec12[is.na(A$Dec_Age25)
&A$AGE12==25]
table(A$Dec_Age25,useNA = "always")
A$Dec_Age25[is.na(A$Dec_Age25) & A$AGE13==25]<-A$Dec13[is.na(A$Dec_Age25)
&A$AGE13==25]
```

© 2023 Silver IA et al. *JAMA Network Open.*

```
table(A$Dec_Age25,useNA = "always")
A$Dec_Age25[is.na(A$Dec_Age25) & A$AGE14==25]<-A$Dec14[is.na(A$Dec_Age25)
&A$AGE14==25]
table(A$Dec_Age25,useNA = "always")
A$Dec_Age25[is.na(A$Dec_Age25) & A$AGE15==25]<-A$Dec15[is.na(A$Dec_Age25)
&A$AGE15==25]
table(A$Dec_Age25,useNA = "always")
A$Dec_Age25[is.na(A$Dec_Age25) & A$AGE16==25]<-A$Dec16[is.na(A$Dec_Age25)
&A$AGE16==25]
table(A$Dec_Age25,useNA = "always")
A$Dec_Age25[is.na(A$Dec_Age25) & A$AGE17==25]<-A$Dec17[is.na(A$Dec_Age25)
&A$AGE17==25]
table(A$Dec_Age25,useNA = "always")
### Deceased Age 26
A$Dec_Age26<-NA
A$Dec_Age26[A$Dec_Age25==1]<-1
A$Dec_Age26[is.na(A$Dec_Age26) &A$AGE98==26]<-A$Dec98[is.na(A$Dec_Age26)
&A$AGE98==26]
table(A$Dec_Age26,useNA = "always")
A$Dec_Age26[is.na(A$Dec_Age26) &A$AGE99==26]<-A$Dec99[is.na(A$Dec_Age26)
&A$AGE99==26]
table(A$Dec_Age26,useNA = "always")
A$Dec_Age26[is.na(A$Dec_Age26) & A$AGE00==26]<-A$Dec00[is.na(A$Dec_Age26)
&A$AGE00==26]
table(A$Dec_Age26,useNA = "always")
A$Dec_Age26[is.na(A$Dec_Age26) & A$AGE01==26]<-A$Dec01[is.na(A$Dec_Age26)
&A$AGE01==26]
table(A$Dec_Age26,useNA = "always")
A$Dec_Age26[is.na(A$Dec_Age26) & A$AGE02==26]<-A$Dec02[is.na(A$Dec_Age26)
&A$AGE02==26]
table(A$Dec_Age26,useNA = "always")
A$Dec_Age26[is.na(A$Dec_Age26) & A$AGE03==26]<-A$Dec03[is.na(A$Dec_Age26)
&A$AGE03==26]
table(A$Dec_Age26,useNA = "always")
A$Dec_Age26[is.na(A$Dec_Age26) & A$AGE04==26]<-A$Dec04[is.na(A$Dec_Age26)
&A$AGE04==26]
table(A$Dec_Age26,useNA = "always")
A$Dec_Age26[is.na(A$Dec_Age26) & A$AGE05==26]<-A$Dec05[is.na(A$Dec_Age26)
&A$AGE05==26]
table(A$Dec_Age26,useNA = "always")
A$Dec_Age26[is.na(A$Dec_Age26) & A$AGE06==26]<-A$Dec06[is.na(A$Dec_Age26)
&A$AGE06==26]
table(A$Dec_Age26,useNA = "always")
A$Dec_Age26[is.na(A$Dec_Age26) & A$AGE07==26]<-A$Dec07[is.na(A$Dec_Age26)
&A$AGE07==26]
table(A$Dec_Age26,useNA = "always")
A$Dec_Age26[is.na(A$Dec_Age26) & A$AGE08==26]<-A$Dec08[is.na(A$Dec_Age26)
&A$AGE08==26]
table(A$Dec_Age26,useNA = "always")
A$Dec_Age26[is.na(A$Dec_Age26) & A$AGE09==26]<-A$Dec09[is.na(A$Dec_Age26)
&A$AGE09==26]
table(A$Dec_Age26,useNA = "always")
A$Dec_Age26[is.na(A$Dec_Age26) & A$AGE10==26]<-A$Dec10[is.na(A$Dec_Age26)
&A$AGE10==26]
table(A$Dec_Age26,useNA = "always")
```

© 2023 Silver IA et al. *JAMA Network Open.*

```
A$Dec_Age26[is.na(A$Dec_Age26) & A$AGE11==26]<-A$Dec11[is.na(A$Dec_Age26)
&A$AGE11==26]
table(A$Dec_Age26,useNA = "always")
A$Dec_Age26[is.na(A$Dec_Age26) & A$AGE12==26]<-A$Dec12[is.na(A$Dec_Age26)
&A$AGE12==26]
table(A$Dec_Age26,useNA = "always")
A$Dec_Age26[is.na(A$Dec_Age26) & A$AGE13==26]<-A$Dec13[is.na(A$Dec_Age26)
&A$AGE13==26]
table(A$Dec_Age26,useNA = "always")
A$Dec_Age26[is.na(A$Dec_Age26) & A$AGE14==26]<-A$Dec14[is.na(A$Dec_Age26)
&A$AGE14==26]
table(A$Dec_Age26,useNA = "always")
A$Dec_Age26[is.na(A$Dec_Age26) & A$AGE15==26]<-A$Dec15[is.na(A$Dec_Age26)
&A$AGE15==26]
table(A$Dec_Age26,useNA = "always")
A$Dec_Age26[is.na(A$Dec_Age26) & A$AGE16==26]<-A$Dec16[is.na(A$Dec_Age26)
&A$AGE16==26]
table(A$Dec_Age26,useNA = "always")
A$Dec_Age26[is.na(A$Dec_Age26) & A$AGE17==26]<-A$Dec17[is.na(A$Dec_Age26)
&A$AGE17==26]
table(A$Dec_Age26,useNA = "always")
### Deceased Age 27
A$Dec_Age27<-NA
A$Dec_Age27[A$Dec_Age26==1]<-1
A$Dec_Age27[is.na(A$Dec_Age27) &A$AGE98==27]<-A$Dec98[is.na(A$Dec_Age27)
&A$AGE98==27]
table(A$Dec_Age27,useNA = "always")
A$Dec_Age27[is.na(A$Dec_Age27) &A$AGE99==27]<-A$Dec99[is.na(A$Dec_Age27)
&A$AGE99==27]
table(A$Dec_Age27,useNA = "always")
A$Dec_Age27[is.na(A$Dec_Age27) & A$AGE00==27]<-A$Dec00[is.na(A$Dec_Age27)
&A$AGE00==27]
table(A$Dec_Age27,useNA = "always")
A$Dec_Age27[is.na(A$Dec_Age27) & A$AGE01==27]<-A$Dec01[is.na(A$Dec_Age27)
&A$AGE01==27]
table(A$Dec_Age27,useNA = "always")
A$Dec_Age27[is.na(A$Dec_Age27) & A$AGE02==27]<-A$Dec02[is.na(A$Dec_Age27)
&A$AGE02==27]
table(A$Dec_Age27,useNA = "always")
A$Dec_Age27[is.na(A$Dec_Age27) & A$AGE03==27]<-A$Dec03[is.na(A$Dec_Age27)
&A$AGE03==27]
table(A$Dec_Age27,useNA = "always")
A$Dec_Age27[is.na(A$Dec_Age27) & A$AGE04==27]<-A$Dec04[is.na(A$Dec_Age27)
&A$AGE04==27]
table(A$Dec_Age27,useNA = "always")
A$Dec_Age27[is.na(A$Dec_Age27) & A$AGE05==27]<-A$Dec05[is.na(A$Dec_Age27)
&A$AGE05==27]
table(A$Dec_Age27,useNA = "always")
A$Dec_Age27[is.na(A$Dec_Age27) & A$AGE06==27]<-A$Dec06[is.na(A$Dec_Age27)
&A$AGE06==27]
table(A$Dec_Age27,useNA = "always")
A$Dec_Age27[is.na(A$Dec_Age27) & A$AGE07==27]<-A$Dec07[is.na(A$Dec_Age27)
&A$AGE07==27]
table(A$Dec_Age27,useNA = "always")
A$Dec_Age27[is.na(A$Dec_Age27) & A$AGE08==27]<-A$Dec08[is.na(A$Dec_Age27)
&A$AGE08==27]
```

```
table(A$Dec_Age27,useNA = "always")
A$Dec_Age27[is.na(A$Dec_Age27) & A$AGE09==27]<-A$Dec09[is.na(A$Dec_Age27)
&A$AGE09==27]
table(A$Dec_Age27,useNA = "always")
A$Dec_Age27[is.na(A$Dec_Age27) & A$AGE10==27]<-A$Dec10[is.na(A$Dec_Age27)
&A$AGE10==27]
table(A$Dec_Age27,useNA = "always")
A$Dec_Age27[is.na(A$Dec_Age27) & A$AGE11==27]<-A$Dec11[is.na(A$Dec_Age27)
&A$AGE11==27]
table(A$Dec_Age27,useNA = "always")
A$Dec_Age27[is.na(A$Dec_Age27) & A$AGE13==27]<-A$Dec13[is.na(A$Dec_Age27)
&A$AGE13==27]
table(A$Dec_Age27,useNA = "always")
A$Dec_Age27[is.na(A$Dec_Age27) & A$AGE15==27]<-A$Dec15[is.na(A$Dec_Age27)
&A$AGE15==27]
table(A$Dec_Age27,useNA = "always")
A$Dec_Age27[is.na(A$Dec_Age27) & A$AGE17==27]<-A$Dec17[is.na(A$Dec_Age27)
&A$AGE17==27]
table(A$Dec_Age27,useNA = "always")
### Deceased Age 28
A$Dec_Age28<-NA
A$Dec_Age28[A$Dec_Age27==1]<-1
A$Dec_Age28[is.na(A$Dec_Age28) &A$AGE98==28]<-A$Dec98[is.na(A$Dec_Age28)
&A$AGE98==28]
table(A$Dec_Age28,useNA = "always")
A$Dec_Age28[is.na(A$Dec_Age28) &A$AGE99==28]<-A$Dec99[is.na(A$Dec_Age28)
&A$AGE99==28]
table(A$Dec_Age28,useNA = "always")
A$Dec_Age28[is.na(A$Dec_Age28) & A$AGE00==28]<-A$Dec00[is.na(A$Dec_Age28)
&A$AGE00==28]
table(A$Dec_Age28,useNA = "always")
A$Dec_Age28[is.na(A$Dec_Age28) & A$AGE01==28]<-A$Dec01[is.na(A$Dec_Age28)
&A$AGE01==28]
table(A$Dec_Age28,useNA = "always")
A$Dec_Age28[is.na(A$Dec_Age28) & A$AGE02==28]<-A$Dec02[is.na(A$Dec_Age28)
&A$AGE02==28]
table(A$Dec_Age28,useNA = "always")
A$Dec_Age28[is.na(A$Dec_Age28) & A$AGE03==28]<-A$Dec03[is.na(A$Dec_Age28)
&A$AGE03==28]
table(A$Dec_Age28,useNA = "always")
A$Dec_Age28[is.na(A$Dec_Age28) & A$AGE04==28]<-A$Dec04[is.na(A$Dec_Age28)
&A$AGE04==28]
table(A$Dec_Age28,useNA = "always")
A$Dec_Age28[is.na(A$Dec_Age28) & A$AGE05==28]<-A$Dec05[is.na(A$Dec_Age28)
&A$AGE05==28]
table(A$Dec_Age28,useNA = "always")
A$Dec_Age28[is.na(A$Dec_Age28) & A$AGE06==28]<-A$Dec06[is.na(A$Dec_Age28)
&A$AGE06==28]
table(A$Dec_Age28,useNA = "always")
A$Dec_Age28[is.na(A$Dec_Age28) & A$AGE07==28]<-A$Dec07[is.na(A$Dec_Age28)
&A$AGE07==28]
table(A$Dec_Age28,useNA = "always")
A$Dec_Age28[is.na(A$Dec_Age28) & A$AGE08==28]<-A$Dec08[is.na(A$Dec_Age28)
&A$AGE08==28]
table(A$Dec_Age28,useNA = "always")
```

© 2023 Silver IA et al. *JAMA Network Open.*

```
A$Dec_Age28[is.na(A$Dec_Age28) & A$AGE09==28]<-A$Dec09[is.na(A$Dec_Age28)
&A$AGE09==28]
table(A$Dec_Age28,useNA = "always")
A$Dec_Age28[is.na(A$Dec_Age28) & A$AGE10==28]<-A$Dec10[is.na(A$Dec_Age28)
&A$AGE10==28]
table(A$Dec_Age28,useNA = "always")
A$Dec_Age28[is.na(A$Dec_Age28) & A$AGE11==28]<-A$Dec11[is.na(A$Dec_Age28)
&A$AGE11==28]
table(A$Dec_Age28,useNA = "always")
A$Dec_Age28[is.na(A$Dec_Age28) & A$AGE13==28]<-A$Dec13[is.na(A$Dec_Age28)
&A$AGE13==28]
table(A$Dec_Age28,useNA = "always")
A$Dec_Age28[is.na(A$Dec_Age28) & A$AGE15==28]<-A$Dec15[is.na(A$Dec_Age28)
&A$AGE15==28]
table(A$Dec_Age28,useNA = "always")
A$Dec_Age28[is.na(A$Dec_Age28) & A$AGE17==28]<-A$Dec17[is.na(A$Dec_Age28)
&A$AGE17==28]
table(A$Dec_Age28,useNA = "always")
### Deceased Age 29
A$Dec_Age29<-NA
A$Dec_Age29[A$Dec_Age28==1]<-1
A$Dec_Age29[is.na(A$Dec_Age29) &A$AGE98==29]<-A$Dec98[is.na(A$Dec_Age29)
&A$AGE98==29]
table(A$Dec_Age29,useNA = "always")
A$Dec_Age29[is.na(A$Dec_Age29) &A$AGE99==29]<-A$Dec99[is.na(A$Dec_Age29)
&A$AGE99==29]
table(A$Dec_Age29,useNA = "always")
A$Dec_Age29[is.na(A$Dec_Age29) & A$AGE00==29]<-A$Dec00[is.na(A$Dec_Age29)
&A$AGE00==29]
table(A$Dec_Age29,useNA = "always")
A$Dec_Age29[is.na(A$Dec_Age29) & A$AGE01==29]<-A$Dec01[is.na(A$Dec_Age29)
&A$AGE01==29]
table(A$Dec_Age29,useNA = "always")
A$Dec_Age29[is.na(A$Dec_Age29) & A$AGE02==29]<-A$Dec02[is.na(A$Dec_Age29)
&A$AGE02==29]
table(A$Dec_Age29,useNA = "always")
A$Dec_Age29[is.na(A$Dec_Age29) & A$AGE03==29]<-A$Dec03[is.na(A$Dec_Age29)
&A$AGE03==29]
table(A$Dec_Age29,useNA = "always")
A$Dec_Age29[is.na(A$Dec_Age29) & A$AGE04==29]<-A$Dec04[is.na(A$Dec_Age29)
&A$AGE04==29]
table(A$Dec_Age29,useNA = "always")
A$Dec_Age29[is.na(A$Dec_Age29) & A$AGE05==29]<-A$Dec05[is.na(A$Dec_Age29)
&A$AGE05==29]
table(A$Dec_Age29,useNA = "always")
A$Dec_Age29[is.na(A$Dec_Age29) & A$AGE06==29]<-A$Dec06[is.na(A$Dec_Age29)
&A$AGE06==29]
table(A$Dec_Age29,useNA = "always")
A$Dec_Age29[is.na(A$Dec_Age29) & A$AGE07==29]<-A$Dec07[is.na(A$Dec_Age29)
&A$AGE07==29]
table(A$Dec_Age29,useNA = "always")
A$Dec_Age29[is.na(A$Dec_Age29) & A$AGE08==29]<-A$Dec08[is.na(A$Dec_Age29)
&A$AGE08==29]
table(A$Dec_Age29,useNA = "always")
A$Dec_Age29[is.na(A$Dec_Age29) & A$AGE09==29]<-A$Dec09[is.na(A$Dec_Age29)
&A$AGE09==29]
```

© 2023 Silver IA et al. *JAMA Network Open.*

```
table(A$Dec_Age29,useNA = "always")
A$Dec_Age29[is.na(A$Dec_Age29) & A$AGE10==29]<-A$Dec10[is.na(A$Dec_Age29)
&A$AGE10==29]
table(A$Dec_Age29,useNA = "always")
A$Dec_Age29[is.na(A$Dec_Age29) & A$AGE11==29]<-A$Dec11[is.na(A$Dec_Age29)
&A$AGE11==29]
table(A$Dec_Age29,useNA = "always")
A$Dec_Age29[is.na(A$Dec_Age29) & A$AGE13==29]<-A$Dec13[is.na(A$Dec_Age29)
&A$AGE13==29]
table(A$Dec_Age29,useNA = "always")
A$Dec_Age29[is.na(A$Dec_Age29) & A$AGE15==29]<-A$Dec15[is.na(A$Dec_Age29)
&A$AGE15==29]
table(A$Dec_Age29,useNA = "always")
A$Dec_Age29[is.na(A$Dec_Age29) & A$AGE17==29]<-A$Dec17[is.na(A$Dec_Age29)
&A$AGE17==29]
table(A$Dec_Age29,useNA = "always")
### Deceased Age 30
A$Dec_Age30<-NA
A$Dec_Age30[A$Dec_Age29==1]<-1
A$Dec_Age30[is.na(A$Dec_Age30) &A$AGE98==30]<-A$Dec98[is.na(A$Dec_Age30)
&A$AGE98==30]
table(A$Dec_Age30,useNA = "always")
A$Dec_Age30[is.na(A$Dec_Age30) &A$AGE99==30]<-A$Dec99[is.na(A$Dec_Age30)
&A$AGE99==30]
table(A$Dec_Age30,useNA = "always")
A$Dec_Age30[is.na(A$Dec_Age30) & A$AGE00==30]<-A$Dec00[is.na(A$Dec_Age30)
&A$AGE00==30]
table(A$Dec_Age30,useNA = "always")
A$Dec_Age30[is.na(A$Dec_Age30) & A$AGE01==30]<-A$Dec01[is.na(A$Dec_Age30)
&A$AGE01==30]
table(A$Dec_Age30,useNA = "always")
A$Dec_Age30[is.na(A$Dec_Age30) & A$AGE02==30]<-A$Dec02[is.na(A$Dec_Age30)
&A$AGE02==30]
table(A$Dec_Age30,useNA = "always")
A$Dec_Age30[is.na(A$Dec_Age30) & A$AGE03==30]<-A$Dec03[is.na(A$Dec_Age30)
&A$AGE03==30]
table(A$Dec_Age30,useNA = "always")
A$Dec_Age30[is.na(A$Dec_Age30) & A$AGE04==30]<-A$Dec04[is.na(A$Dec_Age30)
&A$AGE04==30]
table(A$Dec_Age30,useNA = "always")
A$Dec_Age30[is.na(A$Dec_Age30) & A$AGE05==30]<-A$Dec05[is.na(A$Dec_Age30)
&A$AGE05==30]
table(A$Dec_Age30,useNA = "always")
A$Dec_Age30[is.na(A$Dec_Age30) & A$AGE06==30]<-A$Dec06[is.na(A$Dec_Age30)
&A$AGE06==30]
table(A$Dec_Age30,useNA = "always")
A$Dec_Age30[is.na(A$Dec_Age30) & A$AGE07==30]<-A$Dec07[is.na(A$Dec_Age30)
&A$AGE07==30]
table(A$Dec_Age30,useNA = "always")
A$Dec_Age30[is.na(A$Dec_Age30) & A$AGE08==30]<-A$Dec08[is.na(A$Dec_Age30)
&A$AGE08==30]
table(A$Dec_Age30,useNA = "always")
A$Dec_Age30[is.na(A$Dec_Age30) & A$AGE09==30]<-A$Dec09[is.na(A$Dec_Age30)
&A$AGE09==30]
table(A$Dec_Age30,useNA = "always")
```

© 2023 Silver IA et al. *JAMA Network Open.*

```
A$Dec_Age30[is.na(A$Dec_Age30) & A$AGE10==30]<-A$Dec10[is.na(A$Dec_Age30)
&A$AGE10==30]
table(A$Dec_Age30,useNA = "always")
A$Dec_Age30[is.na(A$Dec_Age30) & A$AGE11==30]<-A$Dec11[is.na(A$Dec_Age30)
&A$AGE11==30]
table(A$Dec_Age30,useNA = "always")
A$Dec_Age30[is.na(A$Dec_Age30) & A$AGE13==30]<-A$Dec13[is.na(A$Dec_Age30)
&A$AGE13==30]
table(A$Dec_Age30,useNA = "always")
A$Dec_Age30[is.na(A$Dec_Age30) & A$AGE15==30]<-A$Dec15[is.na(A$Dec_Age30)
&A$AGE15==30]
table(A$Dec_Age30,useNA = "always")
A$Dec_Age30[is.na(A$Dec_Age30) & A$AGE17==30]<-A$Dec17[is.na(A$Dec_Age30)
&A$AGE17==30]
table(A$Dec_Age30,useNA = "always")
### Deceased Age 31
A$Dec_Age31<-NA
A$Dec_Age31[A$Dec_Age30==1]<-1
A$Dec_Age31[is.na(A$Dec_Age31) &A$AGE98==31]<-A$Dec98[is.na(A$Dec_Age31)
&A$AGE98==31]
table(A$Dec_Age31,useNA = "always")
A$Dec_Age31[is.na(A$Dec_Age31) &A$AGE99==31]<-A$Dec99[is.na(A$Dec_Age31)
&A$AGE99==31]
table(A$Dec_Age31,useNA = "always")
A$Dec_Age31[is.na(A$Dec_Age31) & A$AGE00==31]<-A$Dec00[is.na(A$Dec_Age31)
&A$AGE00==31]
table(A$Dec_Age31,useNA = "always")
A$Dec_Age31[is.na(A$Dec_Age31) & A$AGE01==31]<-A$Dec01[is.na(A$Dec_Age31)
&A$AGE01==31]
table(A$Dec_Age31,useNA = "always")
A$Dec_Age31[is.na(A$Dec_Age31) & A$AGE02==31]<-A$Dec02[is.na(A$Dec_Age31)
&A$AGE02==31]
table(A$Dec_Age31,useNA = "always")
A$Dec_Age31[is.na(A$Dec_Age31) & A$AGE03==31]<-A$Dec03[is.na(A$Dec_Age31)
&A$AGE03==31]
table(A$Dec_Age31,useNA = "always")
A$Dec_Age31[is.na(A$Dec_Age31) & A$AGE04==31]<-A$Dec04[is.na(A$Dec_Age31)
&A$AGE04==31]
table(A$Dec_Age31,useNA = "always")
A$Dec_Age31[is.na(A$Dec_Age31) & A$AGE05==31]<-A$Dec05[is.na(A$Dec_Age31)
&A$AGE05==31]
table(A$Dec_Age31,useNA = "always")
A$Dec_Age31[is.na(A$Dec_Age31) & A$AGE06==31]<-A$Dec06[is.na(A$Dec_Age31)
&A$AGE06==31]
table(A$Dec_Age31,useNA = "always")
A$Dec_Age31[is.na(A$Dec_Age31) & A$AGE07==31]<-A$Dec07[is.na(A$Dec_Age31)
&A$AGE07==31]
table(A$Dec_Age31,useNA = "always")
A$Dec_Age31[is.na(A$Dec_Age31) & A$AGE08==31]<-A$Dec08[is.na(A$Dec_Age31)
&A$AGE08==31]
table(A$Dec_Age31,useNA = "always")
A$Dec_Age31[is.na(A$Dec_Age31) & A$AGE09==31]<-A$Dec09[is.na(A$Dec_Age31)
&A$AGE09==31]
table(A$Dec_Age31,useNA = "always")
A$Dec_Age31[is.na(A$Dec_Age31) & A$AGE10==31]<-A$Dec10[is.na(A$Dec_Age31)
&A$AGE10==31]
```

© 2023 Silver IA et al. *JAMA Network Open.*

```
table(A$Dec_Age31,useNA = "always")
A$Dec_Age31[is.na(A$Dec_Age31) & A$AGE11==31]<-A$Dec11[is.na(A$Dec_Age31)
&A$AGE11==31]
table(A$Dec_Age31,useNA = "always")
A$Dec_Age31[is.na(A$Dec_Age31) & A$AGE13==31]<-A$Dec13[is.na(A$Dec_Age31)
&A$AGE13==31]
table(A$Dec_Age31,useNA = "always")
A$Dec_Age31[is.na(A$Dec_Age31) & A$AGE15==31]<-A$Dec15[is.na(A$Dec_Age31)
&A$AGE15==31]
table(A$Dec_Age31,useNA = "always")
A$Dec_Age31[is.na(A$Dec_Age31) & A$AGE17==31]<-A$Dec17[is.na(A$Dec_Age31)
&A$AGE17==31]
table(A$Dec_Age31,useNA = "always")
### Deceased Age 32
A$Dec_Age32<-NA
A$Dec_Age32[A$Dec_Age31==1]<-1
A$Dec_Age32[is.na(A$Dec_Age32) &A$AGE98==32]<-A$Dec98[is.na(A$Dec_Age32)
&A$AGE98==32]
table(A$Dec_Age32,useNA = "always")
A$Dec_Age32[is.na(A$Dec_Age32) &A$AGE99==32]<-A$Dec99[is.na(A$Dec_Age32)
&A$AGE99==32]
table(A$Dec_Age32,useNA = "always")
A$Dec_Age32[is.na(A$Dec_Age32) & A$AGE00==32]<-A$Dec00[is.na(A$Dec_Age32)
&A$AGE00==32]
table(A$Dec_Age32,useNA = "always")
A$Dec_Age32[is.na(A$Dec_Age32) & A$AGE01==32]<-A$Dec01[is.na(A$Dec_Age32)
&A$AGE01==32]
table(A$Dec_Age32,useNA = "always")
A$Dec_Age32[is.na(A$Dec_Age32) & A$AGE02==32]<-A$Dec02[is.na(A$Dec_Age32)
&A$AGE02==32]
table(A$Dec_Age32,useNA = "always")
A$Dec_Age32[is.na(A$Dec_Age32) & A$AGE03==32]<-A$Dec03[is.na(A$Dec_Age32)
&A$AGE03==32]
table(A$Dec_Age32,useNA = "always")
A$Dec_Age32[is.na(A$Dec_Age32) & A$AGE04==32]<-A$Dec04[is.na(A$Dec_Age32)
&A$AGE04==32]
table(A$Dec_Age32,useNA = "always")
A$Dec_Age32[is.na(A$Dec_Age32) & A$AGE05==32]<-A$Dec05[is.na(A$Dec_Age32)
&A$AGE05==32]
table(A$Dec_Age32,useNA = "always")
A$Dec_Age32[is.na(A$Dec_Age32) & A$AGE06==32]<-A$Dec06[is.na(A$Dec_Age32)
&A$AGE06==32]
table(A$Dec_Age32,useNA = "always")
A$Dec_Age32[is.na(A$Dec_Age32) & A$AGE07==32]<-A$Dec07[is.na(A$Dec_Age32)
&A$AGE07==32]
table(A$Dec_Age32,useNA = "always")
A$Dec_Age32[is.na(A$Dec_Age32) & A$AGE08==32]<-A$Dec08[is.na(A$Dec_Age32)
&A$AGE08==32]
table(A$Dec_Age32,useNA = "always")
A$Dec_Age32[is.na(A$Dec_Age32) & A$AGE09==32]<-A$Dec09[is.na(A$Dec_Age32)
&A$AGE09==32]
table(A$Dec_Age32,useNA = "always")
A$Dec_Age32[is.na(A$Dec_Age32) & A$AGE10==32]<-A$Dec10[is.na(A$Dec_Age32)
&A$AGE10==32]
table(A$Dec_Age32,useNA = "always")
```

© 2023 Silver IA et al. *JAMA Network Open.*

```
A$Dec_Age32[is.na(A$Dec_Age32) & A$AGE11==32]<-A$Dec11[is.na(A$Dec_Age32)
&A$AGE11==32]
table(A$Dec_Age32,useNA = "always")
A$Dec_Age32[is.na(A$Dec_Age32) & A$AGE13==32]<-A$Dec13[is.na(A$Dec_Age32)
&A$AGE13==32]
table(A$Dec_Age32,useNA = "always")
A$Dec_Age32[is.na(A$Dec_Age32) & A$AGE15==32]<-A$Dec15[is.na(A$Dec_Age32)
&A$AGE15==32]
table(A$Dec_Age32,useNA = "always")
A$Dec_Age32[is.na(A$Dec_Age32) & A$AGE17==32]<-A$Dec17[is.na(A$Dec_Age32)
&A$AGE17==32]
table(A$Dec_Age32,useNA = "always")
### Deceased Age 33
A$Dec_Age33<-NA
A$Dec_Age33[A$Dec_Age32==1]<-1
A$Dec_Age33[is.na(A$Dec_Age33) &A$AGE98==33]<-A$Dec98[is.na(A$Dec_Age33)
&A$AGE98==33]
table(A$Dec_Age33,useNA = "always")
A$Dec_Age33[is.na(A$Dec_Age33) &A$AGE99==33]<-A$Dec99[is.na(A$Dec_Age33)
&A$AGE99==33]
table(A$Dec_Age33,useNA = "always")
A$Dec_Age33[is.na(A$Dec_Age33) & A$AGE00==33]<-A$Dec00[is.na(A$Dec_Age33)
&A$AGE00==33]
table(A$Dec_Age33,useNA = "always")
A$Dec_Age33[is.na(A$Dec_Age33) & A$AGE01==33]<-A$Dec01[is.na(A$Dec_Age33)
&A$AGE01==33]
table(A$Dec_Age33,useNA = "always")
A$Dec_Age33[is.na(A$Dec_Age33) & A$AGE02==33]<-A$Dec02[is.na(A$Dec_Age33)
&A$AGE02==33]
table(A$Dec_Age33,useNA = "always")
A$Dec_Age33[is.na(A$Dec_Age33) & A$AGE03==33]<-A$Dec03[is.na(A$Dec_Age33)
&A$AGE03==33]
table(A$Dec_Age33,useNA = "always")
A$Dec_Age33[is.na(A$Dec_Age33) & A$AGE04==33]<-A$Dec04[is.na(A$Dec_Age33)
&A$AGE04==33]
table(A$Dec_Age33,useNA = "always")
A$Dec_Age33[is.na(A$Dec_Age33) & A$AGE05==33]<-A$Dec05[is.na(A$Dec_Age33)
&A$AGE05==33]
table(A$Dec_Age33,useNA = "always")
A$Dec_Age33[is.na(A$Dec_Age33) & A$AGE06==33]<-A$Dec06[is.na(A$Dec_Age33)
&A$AGE06==33]
table(A$Dec_Age33,useNA = "always")
A$Dec_Age33[is.na(A$Dec_Age33) & A$AGE07==33]<-A$Dec07[is.na(A$Dec_Age33)
&A$AGE07==33]
table(A$Dec_Age33,useNA = "always")
A$Dec_Age33[is.na(A$Dec_Age33) & A$AGE08==33]<-A$Dec08[is.na(A$Dec_Age33)
&A$AGE08==33]
table(A$Dec_Age33,useNA = "always")
A$Dec_Age33[is.na(A$Dec_Age33) & A$AGE09==33]<-A$Dec09[is.na(A$Dec_Age33)
&A$AGE09==33]
table(A$Dec_Age33,useNA = "always")
A$Dec_Age33[is.na(A$Dec_Age33) & A$AGE10==33]<-A$Dec10[is.na(A$Dec_Age33)
&A$AGE10==33]
table(A$Dec_Age33,useNA = "always")
A$Dec_Age33[is.na(A$Dec_Age33) & A$AGE11==33]<-A$Dec11[is.na(A$Dec_Age33)
&A$AGE11==33]
```

© 2023 Silver IA et al. *JAMA Network Open.*

```
table(A$Dec_Age33,useNA = "always")
A$Dec_Age33[is.na(A$Dec_Age33) & A$AGE13==33]<-A$Dec13[is.na(A$Dec_Age33)
&A$AGE13==33]
table(A$Dec_Age33,useNA = "always")
A$Dec_Age33[is.na(A$Dec_Age33) & A$AGE15==33]<-A$Dec15[is.na(A$Dec_Age33)
&A$AGE15==33]
table(A$Dec_Age33,useNA = "always")
A$Dec_Age33[is.na(A$Dec_Age33) & A$AGE17==33]<-A$Dec17[is.na(A$Dec_Age33)
&A$AGE17==33]
table(A$Dec_Age33,useNA = "always")
### Deceased Age 34
A$Dec_Age34<-NA
A$Dec_Age34[A$Dec_Age33==1]<-1
A$Dec_Age34[is.na(A$Dec_Age34) &A$AGE98==34]<-A$Dec98[is.na(A$Dec_Age34)
&A$AGE98==34]
table(A$Dec_Age34,useNA = "always")
A$Dec_Age34[is.na(A$Dec_Age34) &A$AGE99==34]<-A$Dec99[is.na(A$Dec_Age34)
&A$AGE99==34]
table(A$Dec_Age34,useNA = "always")
A$Dec_Age34[is.na(A$Dec_Age34) & A$AGE00==34]<-A$Dec00[is.na(A$Dec_Age34)
&A$AGE00==34]
table(A$Dec_Age34,useNA = "always")
A$Dec_Age34[is.na(A$Dec_Age34) & A$AGE01==34]<-A$Dec01[is.na(A$Dec_Age34)
&A$AGE01==34]
table(A$Dec_Age34,useNA = "always")
A$Dec_Age34[is.na(A$Dec_Age34) & A$AGE02==34]<-A$Dec02[is.na(A$Dec_Age34)
&A$AGE02==34]
table(A$Dec_Age34,useNA = "always")
A$Dec_Age34[is.na(A$Dec_Age34) & A$AGE03==34]<-A$Dec03[is.na(A$Dec_Age34)
&A$AGE03==34]
table(A$Dec_Age34,useNA = "always")
A$Dec_Age34[is.na(A$Dec_Age34) & A$AGE04==34]<-A$Dec04[is.na(A$Dec_Age34)
&A$AGE04==34]
table(A$Dec_Age34,useNA = "always")
A$Dec_Age34[is.na(A$Dec_Age34) & A$AGE05==34]<-A$Dec05[is.na(A$Dec_Age34)
&A$AGE05==34]
table(A$Dec_Age34,useNA = "always")
A$Dec_Age34[is.na(A$Dec_Age34) & A$AGE06==34]<-A$Dec06[is.na(A$Dec_Age34)
&A$AGE06==34]
table(A$Dec_Age34,useNA = "always")
A$Dec_Age34[is.na(A$Dec_Age34) & A$AGE07==34]<-A$Dec07[is.na(A$Dec_Age34)
&A$AGE07==34]
table(A$Dec_Age34,useNA = "always")
A$Dec_Age34[is.na(A$Dec_Age34) & A$AGE08==34]<-A$Dec08[is.na(A$Dec_Age34)
&A$AGE08==34]
table(A$Dec_Age34,useNA = "always")
A$Dec_Age34[is.na(A$Dec_Age34) & A$AGE09==34]<-A$Dec09[is.na(A$Dec_Age34)
&A$AGE09==34]
table(A$Dec_Age34,useNA = "always")
A$Dec_Age34[is.na(A$Dec_Age34) & A$AGE10==34]<-A$Dec10[is.na(A$Dec_Age34)
&A$AGE10==34]
table(A$Dec_Age34,useNA = "always")
A$Dec_Age34[is.na(A$Dec_Age34) & A$AGE11==34]<-A$Dec11[is.na(A$Dec_Age34)
&A$AGE11==34]
table(A$Dec_Age34,useNA = "always")
```

```
A$Dec_Age34[is.na(A$Dec_Age34) & A$AGE13==34]<-A$Dec13[is.na(A$Dec_Age34)
&A$AGE13==34]
table(A$Dec_Age34,useNA = "always")
A$Dec_Age34[is.na(A$Dec_Age34) & A$AGE15==34]<-A$Dec15[is.na(A$Dec_Age34)
&A$AGE15==34]
table(A$Dec_Age34,useNA = "always")
A$Dec_Age34[is.na(A$Dec_Age34) & A$AGE17==34]<-A$Dec17[is.na(A$Dec_Age34)
&A$AGE17==34]
table(A$Dec_Age34,useNA = "always")
A$Dec_Age34[is.na(A$Dec_Age34) & A$AGE19==34]<-A$Dec19[is.na(A$Dec_Age34)
&A$AGE19==34]
table(A$Dec_Age34,useNA = "always")
### Deceased Age 35
A$Dec_Age35<-NA
A$Dec_Age35[A$Dec_Age34==1]<-1
A$Dec_Age35[is.na(A$Dec_Age35) &A$AGE98==35]<-A$Dec98[is.na(A$Dec_Age35)
&A$AGE98==35]
table(A$Dec_Age35,useNA = "always")
A$Dec_Age35[is.na(A$Dec_Age35) &A$AGE99==35]<-A$Dec99[is.na(A$Dec_Age35)
&A$AGE99==35]
table(A$Dec_Age35,useNA = "always")
A$Dec_Age35[is.na(A$Dec_Age35) & A$AGE00==35]<-A$Dec00[is.na(A$Dec_Age35)
&A$AGE00==35]
table(A$Dec_Age35,useNA = "always")
A$Dec_Age35[is.na(A$Dec_Age35) & A$AGE01==35]<-A$Dec01[is.na(A$Dec_Age35)
&A$AGE01==35]
table(A$Dec_Age35,useNA = "always")
A$Dec_Age35[is.na(A$Dec_Age35) & A$AGE02==35]<-A$Dec02[is.na(A$Dec_Age35)
&A$AGE02==35]
table(A$Dec_Age35,useNA = "always")
A$Dec_Age35[is.na(A$Dec_Age35) & A$AGE03==35]<-A$Dec03[is.na(A$Dec_Age35)
&A$AGE03==35]
table(A$Dec_Age35,useNA = "always")
A$Dec_Age35[is.na(A$Dec_Age35) & A$AGE04==35]<-A$Dec04[is.na(A$Dec_Age35)
&A$AGE04==35]
table(A$Dec_Age35,useNA = "always")
A$Dec_Age35[is.na(A$Dec_Age35) & A$AGE05==35]<-A$Dec05[is.na(A$Dec_Age35)
&A$AGE05==35]
table(A$Dec_Age35,useNA = "always")
A$Dec_Age35[is.na(A$Dec_Age35) & A$AGE06==35]<-A$Dec06[is.na(A$Dec_Age35)
&A$AGE06==35]
table(A$Dec_Age35,useNA = "always")
A$Dec_Age35[is.na(A$Dec_Age35) & A$AGE07==35]<-A$Dec07[is.na(A$Dec_Age35)
&A$AGE07==35]
table(A$Dec_Age35,useNA = "always")
A$Dec_Age35[is.na(A$Dec_Age35) & A$AGE08==35]<-A$Dec08[is.na(A$Dec_Age35)
&A$AGE08==35]
table(A$Dec_Age35,useNA = "always")
A$Dec_Age35[is.na(A$Dec_Age35) & A$AGE09==35]<-A$Dec09[is.na(A$Dec_Age35)
&A$AGE09==35]
table(A$Dec_Age35,useNA = "always")
A$Dec_Age35[is.na(A$Dec_Age35) & A$AGE10==35]<-A$Dec10[is.na(A$Dec_Age35)
&A$AGE10==35]
table(A$Dec_Age35,useNA = "always")
A$Dec_Age35[is.na(A$Dec_Age35) & A$AGE11==35]<-A$Dec11[is.na(A$Dec_Age35)
&A$AGE11==35]
```

© 2023 Silver IA et al. *JAMA Network Open.*

```
table(A$Dec_Age35,useNA = "always")
A$Dec_Age35[is.na(A$Dec_Age35) & A$AGE13==35]<-A$Dec13[is.na(A$Dec_Age35)
&A$AGE13==35]
table(A$Dec_Age35,useNA = "always")
A$Dec_Age35[is.na(A$Dec_Age35) & A$AGE15==35]<-A$Dec15[is.na(A$Dec_Age35)
&A$AGE15==35]
table(A$Dec_Age35,useNA = "always")
A$Dec_Age35[is.na(A$Dec_Age35) & A$AGE17==35]<-A$Dec17[is.na(A$Dec_Age35)
&A$AGE17==35]
table(A$Dec_Age35,useNA = "always")
A$Dec_Age35[is.na(A$Dec_Age35) & A$AGE19==35]<-A$Dec19[is.na(A$Dec_Age35)
&A$AGE19==35]
table(A$Dec_Age35,useNA = "always")
### Deceased Age 36
A$Dec_Age36<-NA
A$Dec_Age36[A$Dec_Age35==1]<-1
A$Dec_Age36[is.na(A$Dec_Age36) &A$AGE98==36]<-A$Dec98[is.na(A$Dec_Age36)
&A$AGE98==36]
table(A$Dec_Age36,useNA = "always")
A$Dec_Age36[is.na(A$Dec_Age36) &A$AGE99==36]<-A$Dec99[is.na(A$Dec_Age36)
&A$AGE99==36]
table(A$Dec_Age36,useNA = "always")
A$Dec_Age36[is.na(A$Dec_Age36) & A$AGE00==36]<-A$Dec00[is.na(A$Dec_Age36)
&A$AGE00==36]
table(A$Dec_Age36,useNA = "always")
A$Dec_Age36[is.na(A$Dec_Age36) & A$AGE01==36]<-A$Dec01[is.na(A$Dec_Age36)
&A$AGE01==36]
table(A$Dec_Age36,useNA = "always")
A$Dec_Age36[is.na(A$Dec_Age36) & A$AGE02==36]<-A$Dec02[is.na(A$Dec_Age36)
&A$AGE02==36]
table(A$Dec_Age36,useNA = "always")
A$Dec_Age36[is.na(A$Dec_Age36) & A$AGE03==36]<-A$Dec03[is.na(A$Dec_Age36)
&A$AGE03==36]
table(A$Dec_Age36,useNA = "always")
A$Dec_Age36[is.na(A$Dec_Age36) & A$AGE04==36]<-A$Dec04[is.na(A$Dec_Age36)
&A$AGE04==36]
table(A$Dec_Age36,useNA = "always")
A$Dec_Age36[is.na(A$Dec_Age36) & A$AGE05==36]<-A$Dec05[is.na(A$Dec_Age36)
&A$AGE05==36]
table(A$Dec_Age36,useNA = "always")
A$Dec_Age36[is.na(A$Dec_Age36) & A$AGE06==36]<-A$Dec06[is.na(A$Dec_Age36)
&A$AGE06==36]
table(A$Dec_Age36,useNA = "always")
A$Dec_Age36[is.na(A$Dec_Age36) & A$AGE07==36]<-A$Dec07[is.na(A$Dec_Age36)
&A$AGE07==36]
table(A$Dec_Age36,useNA = "always")
A$Dec_Age36[is.na(A$Dec_Age36) & A$AGE08==36]<-A$Dec08[is.na(A$Dec_Age36)
&A$AGE08==36]
table(A$Dec_Age36,useNA = "always")
A$Dec_Age36[is.na(A$Dec_Age36) & A$AGE09==36]<-A$Dec09[is.na(A$Dec_Age36)
&A$AGE09==36]
table(A$Dec_Age36,useNA = "always")
A$Dec_Age36[is.na(A$Dec_Age36) & A$AGE10==36]<-A$Dec10[is.na(A$Dec_Age36)
&A$AGE10==36]
table(A$Dec_Age36,useNA = "always")
```

© 2023 Silver IA et al. *JAMA Network Open.*

```
A$Dec_Age36[is.na(A$Dec_Age36) & A$AGE11==36]<-A$Dec11[is.na(A$Dec_Age36)
&A$AGE11==36]
table(A$Dec_Age36,useNA = "always")
A$Dec_Age36[is.na(A$Dec_Age36) & A$AGE13==36]<-A$Dec13[is.na(A$Dec_Age36)
&A$AGE13==36]
table(A$Dec_Age36,useNA = "always")
A$Dec_Age36[is.na(A$Dec_Age36) & A$AGE15==36]<-A$Dec15[is.na(A$Dec_Age36)
&A$AGE15==36]
table(A$Dec_Age36,useNA = "always")
A$Dec_Age36[is.na(A$Dec_Age36) & A$AGE17==36]<-A$Dec17[is.na(A$Dec_Age36)
&A$AGE17==36]
table(A$Dec_Age36,useNA = "always")
A$Dec_Age36[is.na(A$Dec_Age36) & A$AGE19==36]<-A$Dec19[is.na(A$Dec_Age36)
&A$AGE19==36]
table(A$Dec_Age36,useNA = "always")
### Deceased Age 37
A$Dec_Age37<-NA
A$Dec_Age37[A$Dec_Age36==1]<-1
A$Dec_Age37[is.na(A$Dec_Age37) &A$AGE98==37]<-A$Dec98[is.na(A$Dec_Age37)
&A$AGE98==37]
table(A$Dec_Age37,useNA = "always")
A$Dec_Age37[is.na(A$Dec_Age37) &A$AGE99==37]<-A$Dec99[is.na(A$Dec_Age37)
&A$AGE99==37]
table(A$Dec_Age37,useNA = "always")
A$Dec_Age37[is.na(A$Dec_Age37) & A$AGE00==37]<-A$Dec00[is.na(A$Dec_Age37)
&A$AGE00==37]
table(A$Dec_Age37,useNA = "always")
A$Dec_Age37[is.na(A$Dec_Age37) & A$AGE01==37]<-A$Dec01[is.na(A$Dec_Age37)
&A$AGE01==37]
table(A$Dec_Age37,useNA = "always")
A$Dec_Age37[is.na(A$Dec_Age37) & A$AGE02==37]<-A$Dec02[is.na(A$Dec_Age37)
&A$AGE02==37]
table(A$Dec_Age37,useNA = "always")
A$Dec_Age37[is.na(A$Dec_Age37) & A$AGE03==37]<-A$Dec03[is.na(A$Dec_Age37)
&A$AGE03==37]
table(A$Dec_Age37,useNA = "always")
A$Dec_Age37[is.na(A$Dec_Age37) & A$AGE04==37]<-A$Dec04[is.na(A$Dec_Age37)
&A$AGE04==37]
table(A$Dec_Age37,useNA = "always")
A$Dec_Age37[is.na(A$Dec_Age37) & A$AGE05==37]<-A$Dec05[is.na(A$Dec_Age37)
&A$AGE05==37]
table(A$Dec_Age37,useNA = "always")
A$Dec_Age37[is.na(A$Dec_Age37) & A$AGE06==37]<-A$Dec06[is.na(A$Dec_Age37)
&A$AGE06==37]
table(A$Dec_Age37,useNA = "always")
A$Dec_Age37[is.na(A$Dec_Age37) & A$AGE07==37]<-A$Dec07[is.na(A$Dec_Age37)
&A$AGE07==37]
table(A$Dec_Age37,useNA = "always")
A$Dec_Age37[is.na(A$Dec_Age37) & A$AGE08==37]<-A$Dec08[is.na(A$Dec_Age37)
&A$AGE08==37]
table(A$Dec_Age37,useNA = "always")
A$Dec_Age37[is.na(A$Dec_Age37) & A$AGE09==37]<-A$Dec09[is.na(A$Dec_Age37)
&A$AGE09==37]
table(A$Dec_Age37,useNA = "always")
A$Dec_Age37[is.na(A$Dec_Age37) & A$AGE10==37]<-A$Dec10[is.na(A$Dec_Age37)
&A$AGE10==37]
```

© 2023 Silver IA et al. *JAMA Network Open.*

```
table(A$Dec_Age37,useNA = "always")
A$Dec_Age37[is.na(A$Dec_Age37) & A$AGE11==37]<-A$Dec11[is.na(A$Dec_Age37)
&A$AGE11==37]
table(A$Dec_Age37,useNA = "always")
A$Dec_Age37[is.na(A$Dec_Age37) & A$AGE13==37]<-A$Dec13[is.na(A$Dec_Age37)
&A$AGE13==37]
table(A$Dec_Age37,useNA = "always")
A$Dec_Age37[is.na(A$Dec_Age37) & A$AGE15==37]<-A$Dec15[is.na(A$Dec_Age37)
&A$AGE15==37]
table(A$Dec_Age37,useNA = "always")
A$Dec_Age37[is.na(A$Dec_Age37) & A$AGE17==37]<-A$Dec17[is.na(A$Dec_Age37)
&A$AGE17==37]
table(A$Dec_Age37,useNA = "always")
A$Dec_Age37[is.na(A$Dec_Age37) & A$AGE19==37]<-A$Dec19[is.na(A$Dec_Age37)
&A$AGE19==37]
table(A$Dec_Age37,useNA = "always")
### Deceased Age 38
A$Dec_Age38<-NA
A$Dec_Age38[A$Dec_Age37==1]<-1
A$Dec_Age38[is.na(A$Dec_Age38) &A$AGE98==38]<-A$Dec98[is.na(A$Dec_Age38)
&A$AGE98==38]
table(A$Dec_Age38,useNA = "always")
A$Dec_Age38[is.na(A$Dec_Age38) &A$AGE99==38]<-A$Dec99[is.na(A$Dec_Age38)
&A$AGE99==38]
table(A$Dec_Age38,useNA = "always")
A$Dec_Age38[is.na(A$Dec_Age38) & A$AGE00==38]<-A$Dec00[is.na(A$Dec_Age38)
&A$AGE00==38]
table(A$Dec_Age38,useNA = "always")
A$Dec_Age38[is.na(A$Dec_Age38) & A$AGE01==38]<-A$Dec01[is.na(A$Dec_Age38)
&A$AGE01==38]
table(A$Dec_Age38,useNA = "always")
A$Dec_Age38[is.na(A$Dec_Age38) & A$AGE02==38]<-A$Dec02[is.na(A$Dec_Age38)
&A$AGE02==38]
table(A$Dec_Age38,useNA = "always")
A$Dec_Age38[is.na(A$Dec_Age38) & A$AGE03==38]<-A$Dec03[is.na(A$Dec_Age38)
&A$AGE03==38]
table(A$Dec_Age38,useNA = "always")
A$Dec_Age38[is.na(A$Dec_Age38) & A$AGE04==38]<-A$Dec04[is.na(A$Dec_Age38)
&A$AGE04==38]
table(A$Dec_Age38,useNA = "always")
A$Dec_Age38[is.na(A$Dec_Age38) & A$AGE05==38]<-A$Dec05[is.na(A$Dec_Age38)
&A$AGE05==38]
table(A$Dec_Age38,useNA = "always")
A$Dec_Age38[is.na(A$Dec_Age38) & A$AGE06==38]<-A$Dec06[is.na(A$Dec_Age38)
&A$AGE06==38]
table(A$Dec_Age38,useNA = "always")
A$Dec_Age38[is.na(A$Dec_Age38) & A$AGE07==38]<-A$Dec07[is.na(A$Dec_Age38)
&A$AGE07==38]
table(A$Dec_Age38,useNA = "always")
A$Dec_Age38[is.na(A$Dec_Age38) & A$AGE08==38]<-A$Dec08[is.na(A$Dec_Age38)
&A$AGE08==38]
table(A$Dec_Age38,useNA = "always")
A$Dec_Age38[is.na(A$Dec_Age38) & A$AGE09==38]<-A$Dec09[is.na(A$Dec_Age38)
&A$AGE09==38]
table(A$Dec_Age38,useNA = "always")
```

© 2023 Silver IA et al. *JAMA Network Open.*

```
A$Dec_Age38[is.na(A$Dec_Age38) & A$AGE10==38]<-A$Dec10[is.na(A$Dec_Age38)
&A$AGE10==38]
table(A$Dec_Age38,useNA = "always")
A$Dec_Age38[is.na(A$Dec_Age38) & A$AGE11==38]<-A$Dec11[is.na(A$Dec_Age38)
&A$AGE11==38]
table(A$Dec_Age38,useNA = "always")
A$Dec_Age38[is.na(A$Dec_Age38) & A$AGE13==38]<-A$Dec13[is.na(A$Dec_Age38)
&A$AGE13==38]
table(A$Dec_Age38,useNA = "always")
A$Dec_Age38[is.na(A$Dec_Age38) & A$AGE15==38]<-A$Dec15[is.na(A$Dec_Age38)
&A$AGE15==38]
table(A$Dec_Age38,useNA = "always")
A$Dec_Age38[is.na(A$Dec_Age38) & A$AGE17==38]<-A$Dec17[is.na(A$Dec_Age38)
&A$AGE17==38]
table(A$Dec_Age38,useNA = "always")
A$Dec_Age38[is.na(A$Dec_Age38) & A$AGE19==38]<-A$Dec19[is.na(A$Dec_Age38)
&A$AGE19==38]
table(A$Dec_Age38,useNA = "always")
### Deceased Age 39
A$Dec_Age39<-NA
A$Dec_Age39[A$Dec_Age38==1]<-1
A$Dec_Age39[is.na(A$Dec_Age39) &A$AGE98==39]<-A$Dec98[is.na(A$Dec_Age39)
&A$AGE98==39]
table(A$Dec_Age39,useNA = "always")
A$Dec_Age39[is.na(A$Dec_Age39) &A$AGE99==39]<-A$Dec99[is.na(A$Dec_Age39)
&A$AGE99==39]
table(A$Dec_Age39,useNA = "always")
A$Dec_Age39[is.na(A$Dec_Age39) & A$AGE00==39]<-A$Dec00[is.na(A$Dec_Age39)
&A$AGE00==39]
table(A$Dec_Age39,useNA = "always")
A$Dec_Age39[is.na(A$Dec_Age39) & A$AGE01==39]<-A$Dec01[is.na(A$Dec_Age39)
&A$AGE01==39]
table(A$Dec_Age39,useNA = "always")
A$Dec_Age39[is.na(A$Dec_Age39) & A$AGE02==39]<-A$Dec02[is.na(A$Dec_Age39)
&A$AGE02==39]
table(A$Dec_Age39,useNA = "always")
A$Dec_Age39[is.na(A$Dec_Age39) & A$AGE03==39]<-A$Dec03[is.na(A$Dec_Age39)
&A$AGE03==39]
table(A$Dec_Age39,useNA = "always")
A$Dec_Age39[is.na(A$Dec_Age39) & A$AGE04==39]<-A$Dec04[is.na(A$Dec_Age39)
&A$AGE04==39]
table(A$Dec_Age39,useNA = "always")
A$Dec_Age39[is.na(A$Dec_Age39) & A$AGE05==39]<-A$Dec05[is.na(A$Dec_Age39)
&A$AGE05==39]
table(A$Dec_Age39,useNA = "always")
A$Dec_Age39[is.na(A$Dec_Age39) & A$AGE06==39]<-A$Dec06[is.na(A$Dec_Age39)
&A$AGE06==39]
table(A$Dec_Age39,useNA = "always")
A$Dec_Age39[is.na(A$Dec_Age39) & A$AGE07==39]<-A$Dec07[is.na(A$Dec_Age39)
&A$AGE07==39]
table(A$Dec_Age39,useNA = "always")
A$Dec_Age39[is.na(A$Dec_Age39) & A$AGE08==39]<-A$Dec08[is.na(A$Dec_Age39)
&A$AGE08==39]
table(A$Dec_Age39,useNA = "always")
A$Dec_Age39[is.na(A$Dec_Age39) & A$AGE09==39]<-A$Dec09[is.na(A$Dec_Age39)
&A$AGE09==39]
```

© 2023 Silver IA et al. *JAMA Network Open.*

```
table(A$Dec_Age39,useNA = "always")
A$Dec_Age39[is.na(A$Dec_Age39) & A$AGE10==39]<-A$Dec10[is.na(A$Dec_Age39)
&A$AGE10==39]
table(A$Dec_Age39,useNA = "always")
A$Dec_Age39[is.na(A$Dec_Age39) & A$AGE11==39]<-A$Dec11[is.na(A$Dec_Age39)
&A$AGE11==39]
table(A$Dec_Age39,useNA = "always")
A$Dec_Age39[is.na(A$Dec_Age39) & A$AGE13==39]<-A$Dec13[is.na(A$Dec_Age39)
&A$AGE13==39]
table(A$Dec_Age39,useNA = "always")
A$Dec_Age39[is.na(A$Dec_Age39) & A$AGE15==39]<-A$Dec15[is.na(A$Dec_Age39)
&A$AGE15==39]
table(A$Dec_Age39,useNA = "always")
A$Dec_Age39[is.na(A$Dec_Age39) & A$AGE17==39]<-A$Dec17[is.na(A$Dec_Age39)
&A$AGE17==39]
table(A$Dec_Age39,useNA = "always")
A$Dec_Age39[is.na(A$Dec_Age39) & A$AGE19==39]<-A$Dec19[is.na(A$Dec_Age39)
&A$AGE19==39]
table(A$Dec_Age39,useNA = "always")
## Age Deceased (And Age at Last Observation)
A$Age_DEC<-NA
A$Age_DEC[is.na(A$Age_DEC) & A$Dec_Age7 ==1]<-7
A$Age_DEC[is.na(A$Age_DEC) & A$Dec_Age8 ==1]<-8
A$Age_DEC[is.na(A$Age_DEC) & A$Dec_Age9 ==1]<-9
A$Age_DEC[is.na(A$Age_DEC) & A$Dec_Age10 ==1]<-10
A$Age_DEC[is.na(A$Age_DEC) & A$Dec_Age11 ==1]<-11
A$Age_DEC[is.na(A$Age_DEC) & A$Dec_Age12 ==1]<-12
A$Age_DEC[is.na(A$Age_DEC) & A$Dec_Age13 ==1]<-13
A$Age_DEC[is.na(A$Age_DEC) & A$Dec_Age14 ==1]<-14
A$Age_DEC[is.na(A$Age_DEC) & A$Dec_Age15 ==1]<-15
A$Age_DEC[is.na(A$Age_DEC) & A$Dec_Age16 ==1]<-16
A$Age_DEC[is.na(A$Age_DEC) & A$Dec_Age17 ==1]<-17
A$Age_DEC[is.na(A$Age_DEC) & A$Dec_Age18 ==1]<-18
A$Age_DEC[is.na(A$Age_DEC) & A$Dec_Age19 ==1]<-19
A$Age_DEC[is.na(A$Age_DEC) & A$Dec_Age20 ==1]<-20
A$Age_DEC[is.na(A$Age_DEC) & A$Dec_Age21 ==1]<-21
A$Age_DEC[is.na(A$Age_DEC) & A$Dec_Age22 ==1]<-22
A$Age_DEC[is.na(A$Age_DEC) & A$Dec_Age23 ==1]<-23
A$Age_DEC[is.na(A$Age_DEC) & A$Dec_Age24 ==1]<-24
A$Age_DEC[is.na(A$Age_DEC) & A$Dec_Age25 ==1]<-25
A$Age_DEC[is.na(A$Age_DEC) & A$Dec_Age26 ==1]<-26
A$Age_DEC[is.na(A$Age_DEC) & A$Dec_Age27 ==1]<-27
A$Age_DEC[is.na(A$Age_DEC) & A$Dec_Age28 ==1]<-28
A$Age_DEC[is.na(A$Age_DEC) & A$Dec_Age29 ==1]<-29
A$Age_DEC[is.na(A$Age_DEC) & A$Dec_Age30 ==1]<-30
A$Age_DEC[is.na(A$Age_DEC) & A$Dec_Age31 ==1]<-31
A$Age_DEC[is.na(A$Age_DEC) & A$Dec_Age32 ==1]<-32
A$Age_DEC[is.na(A$Age_DEC) & A$Dec_Age33 ==1]<-33
A$Age_DEC[is.na(A$Age_DEC) & A$Dec_Age34 ==1]<-34
A$Age_DEC[is.na(A$Age_DEC) & A$Dec_Age35 ==1]<-35
A$Age_DEC[is.na(A$Age_DEC) & A$Dec_Age36 ==1]<-36
A$Age_DEC[is.na(A$Age_DEC) & A$Dec_Age37 ==1]<-37
A$Age_DEC[is.na(A$Age_DEC) & A$Dec_Age38 ==1]<-38
A$Age_DEC[is.na(A$Age_DEC) & A$Dec_Age39 ==1]<-39
table(A$Age_DEC, useNA = "always")
# Deceased censorship
```

© 2023 Silver IA et al. *JAMA Network Open.*

```
A$DEC_CEN<-NA
A$DEC_CEN[is.na(A$Age_DEC)]<-0 # 0 = censored data
A$DEC_CEN[is.na(A$DEC_CEN)]<-1 # 1 = Not Censored
table(A$DEC_CEN)
table(A$DEC_CEN,A$Age_DEC, useNA = "always")

# Filling in the remaining ages
A$Age_DEC[is.na(A$Age_DEC)]<-A$AGE19[is.na(A$Age_DEC)]
table(A$DEC_CEN,A$Age_DEC, useNA = "always")

## Arrest Measures
### Arrest (1992)

table(A$E8011201, useNA="always")
table(A$E8011202)
table(A$E8011203)
table(A$E8011204)
table(A$E8011205)
table(A$E8011206)
table(A$E8011207)
table(A$E8011208)
table(A$E8011209)
table(A$E8011210)
table(A$E8011211)
table(A$E8011212)

A$A9201<-NA
A$A9201[A$E8011201==0]<-0
A$A9201[A$E8011201==99]<-0
A$A9201[A$E8011201>=1 & A$E8011201<99]<-A$E8011201[A$E8011201>=1 &
A$E8011201<99]
A$A9201[is.na(A$A9201) & A$E8033000==-4]<-0
A$A9201[is.na(A$A9201) & A$E8033000==-3]<-0
A$A9201[is.na(A$A9201) & A$E8033000 >= 199300]<-0
A$A9201[is.na(A$A9201) & A$E8033100==0]<-0
A$A9201[is.na(A$A9201) & A$E8033100==-3]<-0
table(A$E8011201, useNA="always")
table(A$A9201, useNA="always")
A$A9202<-NA
A$A9202[A$E8011202==0]<-0
A$A9202[A$E8011202==99]<-0
A$A9202[A$E8011202>=1 & A$E8011202<99]<-A$E8011202[A$E8011202>=1 &
A$E8011202<99]
A$A9202[is.na(A$A9202) & A$E8033000==-4]<-0
A$A9202[is.na(A$A9202) & A$E8033000==-3]<-0
A$A9202[is.na(A$A9202) & A$E8033000 >= 199300]<-0
A$A9202[is.na(A$A9202) & A$E8033100==0]<-0
A$A9202[is.na(A$A9202) & A$E8033100==-3]<-0
table(A$E8011202, useNA="always")
table(A$A9202, useNA="always")
A$A9203<-NA
A$A9203[A$E8011203==0]<-0
A$A9203[A$E8011203==99]<-0
A$A9203[A$E8011203>=1 & A$E8011203<99]<-A$E8011203[A$E8011203>=1 &
A$E8011203<99]
A$A9203[is.na(A$A9203) & A$E8033000==-4]<-0
```

© 2023 Silver IA et al. *JAMA Network Open.*

```
A$A9203[is.na(A$A9203) & A$E8033000==-3]<-0
A$A9203[is.na(A$A9203) & A$E8033000 >= 199300]<-0
A$A9203[is.na(A$A9203) & A$E8033100==0]<-0
A$A9203[is.na(A$A9203) & A$E8033100==-3]<-0
table(A$E8011203, useNA="always")
table(A$A9203, useNA="always")
A$A9204<-NA
A$A9204[A$E8011204==0]<-0
A$A9204[A$E8011204==99]<-0
A$A9204[A$E8011204>=1 & A$E8011204<99]<-A$E8011204[A$E8011204>=1 &
A$E8011204<99]
A$A9204[is.na(A$A9204) & A$E8033000==-4]<-0
A$A9204[is.na(A$A9204) & A$E8033000==-3]<-0
A$A9204[is.na(A$A9204) & A$E8033000 >= 199300]<-0
A$A9204[is.na(A$A9204) & A$E8033100==0]<-0
A$A9204[is.na(A$A9204) & A$E8033100==-3]<-0
table(A$E8011204, useNA="always")
table(A$A9204, useNA="always")
A$A9205<-NA
A$A9205[A$E8011205==0]<-0
A$A9205[A$E8011205==99]<-0
A$A9205[A$E8011205>=1 & A$E8011205<99]<-A$E8011205[A$E8011205>=1 &
A$E8011205<99]
A$A9205[is.na(A$A9205) & A$E8033000==-4]<-0
A$A9205[is.na(A$A9205) & A$E8033000==-3]<-0
A$A9205[is.na(A$A9205) & A$E8033000 >= 199300]<-0
A$A9205[is.na(A$A9205) & A$E8033100==0]<-0
A$A9205[is.na(A$A9205) & A$E8033100==-3]<-0
table(A$E8011205, useNA="always")
table(A$A9205, useNA="always")
A$A9206<-NA
A$A9206[A$E8011206==0]<-0
A$A9206[A$E8011206==99]<-0
A$A9206[A$E8011206>=1 & A$E8011206<99]<-A$E8011206[A$E8011206>=1 &
A$E8011206<99]
A$A9206[is.na(A$A9206) & A$E8033000==-4]<-0
A$A9206[is.na(A$A9206) & A$E8033000==-3]<-0
A$A9206[is.na(A$A9206) & A$E8033000 >= 199300]<-0
A$A9206[is.na(A$A9206) & A$E8033100==0]<-0
A$A9206[is.na(A$A9206) & A$E8033100==-3]<-0
table(A$E8011206, useNA="always")
table(A$A9206, useNA="always")
A$A9207<-NA
A$A9207[A$E8011207==0]<-0
A$A9207[A$E8011207==99]<-0
A$A9207[A$E8011207>=1 & A$E8011207<99]<-A$E8011207[A$E8011207>=1 &
A$E8011207<99]
A$A9207[is.na(A$A9207) & A$E8033000==-4]<-0
A$A9207[is.na(A$A9207) & A$E8033000==-3]<-0
A$A9207[is.na(A$A9207) & A$E8033000 >= 199300]<-0
A$A9207[is.na(A$A9207) & A$E8033100==0]<-0
A$A9207[is.na(A$A9207) & A$E8033100==-3]<-0
table(A$E8011207, useNA="always")
table(A$A9207, useNA="always")
A$A9208<-NA
A$A9208[A$E8011208==0]<-0
```

© 2023 Silver IA et al. *JAMA Network Open.*

```
A$A9208[A$E8011208==99]<-0
A$A9208[A$E8011208>=1 & A$E8011208<99]<-A$E8011208[A$E8011208>=1 &
A$E8011208<99]
A$A9208[is.na(A$A9208) & A$E8033000==-4]<-0
A$A9208[is.na(A$A9208) & A$E8033000==-3]<-0
A$A9208[is.na(A$A9208) & A$E8033000 >= 199300]<-0
A$A9208[is.na(A$A9208) & A$E8033100==0]<-0
A$A9208[is.na(A$A9208) & A$E8033100==-3]<-0
table(A$E8011208, useNA="always")
table(A$A9208, useNA="always")
A$A9209<-NA
A$A9209[A$E8011209==0]<-0
A$A9209[A$E8011209==99]<-0
A$A9209[A$E8011209>=1 & A$E8011209<99]<-A$E8011209[A$E8011209>=1 &
A$E8011209<99]
A$A9209[is.na(A$A9209) & A$E8033000==-4]<-0
A$A9209[is.na(A$A9209) & A$E8033000==-3]<-0
A$A9209[is.na(A$A9209) & A$E8033000 >= 199300]<-0
A$A9209[is.na(A$A9209) & A$E8033100==0]<-0
A$A9209[is.na(A$A9209) & A$E8033100==-3]<-0
table(A$E8011209, useNA="always")
table(A$A9209, useNA="always")
A$A9210<-NA
A$A9210[A$E8011210==0]<-0
A$A9210[A$E8011210==99]<-0
A$A9210[A$E8011210>=1 & A$E8011210<99]<-A$E8011210[A$E8011210>=1 &
A$E8011210<99]
A$A9210[is.na(A$A9210) & A$E8033000==-4]<-0
A$A9210[is.na(A$A9210) & A$E8033000==-3]<-0
A$A9210[is.na(A$A9210) & A$E8033000 >= 199300]<-0
A$A9210[is.na(A$A9210) & A$E8033100==0]<-0
A$A9210[is.na(A$A9210) & A$E8033100==-3]<-0
table(A$E8011210, useNA="always")
table(A$A9210, useNA="always")
A$A9211<-NA
A$A9211[A$E8011211==0]<-0
A$A9211[A$E8011211==99]<-0
A$A9211[A$E8011211>=1 & A$E8011211<99]<-A$E8011211[A$E8011211>=1 &
A$E8011211<99]
A$A9211[is.na(A$A9211) & A$E8033000==-4]<-0
A$A9211[is.na(A$A9211) & A$E8033000==-3]<-0
A$A9211[is.na(A$A9211) & A$E8033000 >= 199300]<-0
A$A9211[is.na(A$A9211) & A$E8033100==0]<-0
A$A9211[is.na(A$A9211) & A$E8033100==-3]<-0
table(A$E8011211, useNA="always")
table(A$A9211, useNA="always")
A$A9212<-NA
A$A9212[A$E8011212==0]<-0
A$A9212[A$E8011212==99]<-0
A$A9212[A$E8011212>=1 & A$E8011212<99]<-A$E8011212[A$E8011212>=1 &
A$E8011212<99]
A$A9212[is.na(A$A9212) & A$E8033000==-4]<-0
A$A9212[is.na(A$A9212) & A$E8033000==-3]<-0
A$A9212[is.na(A$A9212) & A$E8033000 >= 199300]<-0
A$A9212[is.na(A$A9212) & A$E8033100==0]<-0
A$A9212[is.na(A$A9212) & A$E8033100==-3]<-0
```

© 2023 Silver IA et al. *JAMA Network Open.*

```
table(A$E8011212, useNA="always")
table(A$A9212, useNA="always")




A$A92<-
rowSums(data.frame(A$A9201,A$A9202,A$A9203,A$A9204,A$A9205,A$A9206,A$A9207,A$
A9208,A$A9209,A$A9210,A$A9211,A$A9212), na.rm = T)
table(A$A92)



### Arrest (1993)

table(A$E8011301, useNA="always")
table(A$E8011302)
table(A$E8011303)
table(A$E8011304)
table(A$E8011305)
table(A$E8011306)
table(A$E8011307)
table(A$E8011308)
table(A$E8011309)
table(A$E8011310)
table(A$E8011311)
table(A$E8011312)

A$A9301<-NA
A$A9301[A$E8011301==0]<-0
A$A9301[A$E8011301==99]<-0
A$A9301[A$E8011301>=1 & A$E8011301<99]<-A$E8011301[A$E8011301>=1 &
A$E8011301<99]
A$A9301[is.na(A$A9301) & A$E8033000==-4]<-0
A$A9301[is.na(A$A9301) & A$E8033000==-3]<-0
A$A9301[is.na(A$A9301) & A$E8033000 >= 199300]<-0
A$A9301[is.na(A$A9301) & A$E8033100==0]<-0
A$A9301[is.na(A$A9301) & A$E8033100==-3]<-0
table(A$E8011301, useNA="always")
table(A$A9301, useNA="always")
A$A9302<-NA
A$A9302[A$E8011302==0]<-0
A$A9302[A$E8011302==99]<-0
A$A9302[A$E8011302>=1 & A$E8011302<99]<-A$E8011302[A$E8011302>=1 &
A$E8011302<99]
A$A9302[is.na(A$A9302) & A$E8033000==-4]<-0
A$A9302[is.na(A$A9302) & A$E8033000==-3]<-0
A$A9302[is.na(A$A9302) & A$E8033000 >= 199300]<-0
A$A9302[is.na(A$A9302) & A$E8033100==0]<-0
A$A9302[is.na(A$A9302) & A$E8033100==-3]<-0
table(A$E8011302, useNA="always")
table(A$A9302, useNA="always")
A$A9303<-NA
A$A9303[A$E8011303==0]<-0
A$A9303[A$E8011303==99]<-0
```

© 2023 Silver IA et al. *JAMA Network Open.*

```
A$A9303[A$E8011303>=1 & A$E8011303<99]<-A$E8011303[A$E8011303>=1 &
A$E8011303<99]
A$A9303[is.na(A$A9303) & A$E8033000==-4]<-0
A$A9303[is.na(A$A9303) & A$E8033000==-3]<-0
A$A9303[is.na(A$A9303) & A$E8033000 >= 199300]<-0
A$A9303[is.na(A$A9303) & A$E8033100==0]<-0
A$A9303[is.na(A$A9303) & A$E8033100==-3]<-0
table(A$E8011303, useNA="always")
table(A$A9303, useNA="always")
A$A9304<-NA
A$A9304[A$E8011304==0]<-0
A$A9304[A$E8011304==99]<-0
A$A9304[A$E8011304>=1 & A$E8011304<99]<-A$E8011304[A$E8011304>=1 &
A$E8011304<99]
A$A9304[is.na(A$A9304) & A$E8033000==-4]<-0
A$A9304[is.na(A$A9304) & A$E8033000==-3]<-0
A$A9304[is.na(A$A9304) & A$E8033000 >= 199300]<-0
A$A9304[is.na(A$A9304) & A$E8033100==0]<-0
A$A9304[is.na(A$A9304) & A$E8033100==-3]<-0
table(A$E8011304, useNA="always")
table(A$A9304, useNA="always")
A$A9305<-NA
A$A9305[A$E8011305==0]<-0
A$A9305[A$E8011305==99]<-0
A$A9305[A$E8011305>=1 & A$E8011305<99]<-A$E8011305[A$E8011305>=1 &
A$E8011305<99]
A$A9305[is.na(A$A9305) & A$E8033000==-4]<-0
A$A9305[is.na(A$A9305) & A$E8033000==-3]<-0
A$A9305[is.na(A$A9305) & A$E8033000 >= 199300]<-0
A$A9305[is.na(A$A9305) & A$E8033100==0]<-0
A$A9305[is.na(A$A9305) & A$E8033100==-3]<-0
table(A$E8011305, useNA="always")
table(A$A9305, useNA="always")
A$A9306<-NA
A$A9306[A$E8011306==0]<-0
A$A9306[A$E8011306==99]<-0
A$A9306[A$E8011306>=1 & A$E8011306<99]<-A$E8011306[A$E8011306>=1 &
A$E8011306<99]
A$A9306[is.na(A$A9306) & A$E8033000==-4]<-0
A$A9306[is.na(A$A9306) & A$E8033000==-3]<-0
A$A9306[is.na(A$A9306) & A$E8033000 >= 199300]<-0
A$A9306[is.na(A$A9306) & A$E8033100==0]<-0
A$A9306[is.na(A$A9306) & A$E8033100==-3]<-0
table(A$E8011306, useNA="always")
table(A$A9306, useNA="always")
A$A9307<-NA
A$A9307[A$E8011307==0]<-0
A$A9307[A$E8011307==99]<-0
A$A9307[A$E8011307>=1 & A$E8011307<99]<-A$E8011307[A$E8011307>=1 &
A$E8011307<99]
A$A9307[is.na(A$A9307) & A$E8033000==-4]<-0
A$A9307[is.na(A$A9307) & A$E8033000==-3]<-0
A$A9307[is.na(A$A9307) & A$E8033000 >= 199300]<-0
A$A9307[is.na(A$A9307) & A$E8033100==0]<-0
A$A9307[is.na(A$A9307) & A$E8033100==-3]<-0
table(A$E8011307, useNA="always")
```

```
table(A$A9307, useNA="always")
A$A9308<-NA
A$A9308[A$E8011308==0]<-0
A$A9308[A$E8011308==99]<-0
A$A9308[A$E8011308>=1 & A$E8011308<99]<-A$E8011308[A$E8011308>=1 &
A$E8011308<99]
A$A9308[is.na(A$A9308) & A$E8033000==-4]<-0
A$A9308[is.na(A$A9308) & A$E8033000==-3]<-0
A$A9308[is.na(A$A9308) & A$E8033000 >= 199300]<-0
A$A9308[is.na(A$A9308) & A$E8033100==0]<-0
A$A9308[is.na(A$A9308) & A$E8033100==-3]<-0
table(A$E8011308, useNA="always")
table(A$A9308, useNA="always")
A$A9309<-NA
A$A9309[A$E8011309==0]<-0
A$A9309[A$E8011309==99]<-0
A$A9309[A$E8011309>=1 & A$E8011309<99]<-A$E8011309[A$E8011309>=1 &
A$E8011309<99]
A$A9309[is.na(A$A9309) & A$E8033000==-4]<-0
A$A9309[is.na(A$A9309) & A$E8033000==-3]<-0
A$A9309[is.na(A$A9309) & A$E8033000 >= 199300]<-0
A$A9309[is.na(A$A9309) & A$E8033100==0]<-0
A$A9309[is.na(A$A9309) & A$E8033100==-3]<-0
table(A$E8011309, useNA="always")
table(A$A9309, useNA="always")
A$A9310<-NA
A$A9310[A$E8011310==0]<-0
A$A9310[A$E8011310==99]<-0
A$A9310[A$E8011310>=1 & A$E8011310<99]<-A$E8011310[A$E8011310>=1 &
A$E8011310<99]
A$A9310[is.na(A$A9310) & A$E8033000==-4]<-0
A$A9310[is.na(A$A9310) & A$E8033000==-3]<-0
A$A9310[is.na(A$A9310) & A$E8033000 >= 199300]<-0
A$A9310[is.na(A$A9310) & A$E8033100==0]<-0
A$A9310[is.na(A$A9310) & A$E8033100==-3]<-0
table(A$E8011310, useNA="always")
table(A$A9310, useNA="always")
A$A9311<-NA
A$A9311[A$E8011311==0]<-0
A$A9311[A$E8011311==99]<-0
A$A9311[A$E8011311>=1 & A$E8011311<99]<-A$E8011311[A$E8011311>=1 &
A$E8011311<99]
A$A9311[is.na(A$A9311) & A$E8033000==-4]<-0
A$A9311[is.na(A$A9311) & A$E8033000==-3]<-0
A$A9311[is.na(A$A9311) & A$E8033000 >= 199300]<-0
A$A9311[is.na(A$A9311) & A$E8033100==0]<-0
A$A9311[is.na(A$A9311) & A$E8033100==-3]<-0
table(A$E8011311, useNA="always")
table(A$A9311, useNA="always")
A$A9312<-NA
A$A9312[A$E8011312==0]<-0
A$A9312[A$E8011312==99]<-0
A$A9312[A$E8011312>=1 & A$E8011312<99]<-A$E8011312[A$E8011312>=1 &
A$E8011312<99]
A$A9312[is.na(A$A9312) & A$E8033000==-4]<-0
A$A9312[is.na(A$A9312) & A$E8033000==-3]<-0
```

© 2023 Silver IA et al. *JAMA Network Open.*

```
A$A9312[is.na(A$A9312) & A$E8033000 >= 199300]<-0
A$A9312[is.na(A$A9312) & A$E8033100==0]<-0
A$A9312[is.na(A$A9312) & A$E8033100==-3]<-0
table(A$E8011312, useNA="always")
table(A$A9312, useNA="always")




A$A93<-
rowSums(data.frame(A$A9301,A$A9302,A$A9303,A$A9304,A$A9305,A$A9306,A$A9307,A$
A9308,A$A9309,A$A9310,A$A9311,A$A9312), na.rm = T)
table(A$A93)




### Arrest (1994)

table(A$E8011401, useNA="always")
table(A$E8011402)
table(A$E8011403)
table(A$E8011404)
table(A$E8011405)
table(A$E8011406)
table(A$E8011407)
table(A$E8011408)
table(A$E8011409)
table(A$E8011410)
table(A$E8011411)
table(A$E8011412)

A$A9401<-NA
A$A9401[A$E8011401==0]<-0
A$A9401[A$E8011401==99]<-0
A$A9401[A$E8011401>=1 & A$E8011401<99]<-A$E8011401[A$E8011401>=1 &
A$E8011401<99]
A$A9401[is.na(A$A9401) & A$E8033000==-4]<-0
A$A9401[is.na(A$A9401) & A$E8033000==-3]<-0
A$A9401[is.na(A$A9401) & A$E8033000 >= 199300]<-0
A$A9401[is.na(A$A9401) & A$E8033100==0]<-0
A$A9401[is.na(A$A9401) & A$E8033100==-3]<-0
table(A$E8011401, useNA="always")
table(A$A9401, useNA="always")
A$A9402<-NA
A$A9402[A$E8011402==0]<-0
A$A9402[A$E8011402==99]<-0
A$A9402[A$E8011402>=1 & A$E8011402<99]<-A$E8011402[A$E8011402>=1 &
A$E8011402<99]
A$A9402[is.na(A$A9402) & A$E8033000==-4]<-0
A$A9402[is.na(A$A9402) & A$E8033000==-3]<-0
A$A9402[is.na(A$A9402) & A$E8033000 >= 199300]<-0
A$A9402[is.na(A$A9402) & A$E8033100==0]<-0
A$A9402[is.na(A$A9402) & A$E8033100==-3]<-0
table(A$E8011402, useNA="always")
table(A$A9402, useNA="always")
```

© 2023 Silver IA et al. *JAMA Network Open.*

```
A$A9403<-NA
A$A9403[A$E8011403==0]<-0
A$A9403[A$E8011403==99]<-0
A$A9403[A$E8011403>=1 & A$E8011403<99]<-A$E8011403[A$E8011403>=1 &
A$E8011403<99]
A$A9403[is.na(A$A9403) & A$E8033000==-4]<-0
A$A9403[is.na(A$A9403) & A$E8033000==-3]<-0
A$A9403[is.na(A$A9403) & A$E8033000 >= 199300]<-0
A$A9403[is.na(A$A9403) & A$E8033100==0]<-0
A$A9403[is.na(A$A9403) & A$E8033100==-3]<-0
table(A$E8011403, useNA="always")
table(A$A9403, useNA="always")
A$A9404<-NA
A$A9404[A$E8011404==0]<-0
A$A9404[A$E8011404==99]<-0
A$A9404[A$E8011404>=1 & A$E8011404<99]<-A$E8011404[A$E8011404>=1 &
A$E8011404<99]
A$A9404[is.na(A$A9404) & A$E8033000==-4]<-0
A$A9404[is.na(A$A9404) & A$E8033000==-3]<-0
A$A9404[is.na(A$A9404) & A$E8033000 >= 199300]<-0
A$A9404[is.na(A$A9404) & A$E8033100==0]<-0
A$A9404[is.na(A$A9404) & A$E8033100==-3]<-0
table(A$E8011404, useNA="always")
table(A$A9404, useNA="always")
A$A9405<-NA
A$A9405[A$E8011405==0]<-0
A$A9405[A$E8011405==99]<-0
A$A9405[A$E8011405>=1 & A$E8011405<99]<-A$E8011405[A$E8011405>=1 &
A$E8011405<99]
A$A9405[is.na(A$A9405) & A$E8033000==-4]<-0
A$A9405[is.na(A$A9405) & A$E8033000==-3]<-0
A$A9405[is.na(A$A9405) & A$E8033000 >= 199300]<-0
A$A9405[is.na(A$A9405) & A$E8033100==0]<-0
A$A9405[is.na(A$A9405) & A$E8033100==-3]<-0
table(A$E8011405, useNA="always")
table(A$A9405, useNA="always")
A$A9406<-NA
A$A9406[A$E8011406==0]<-0
A$A9406[A$E8011406==99]<-0
A$A9406[A$E8011406>=1 & A$E8011406<99]<-A$E8011406[A$E8011406>=1 &
A$E8011406<99]
A$A9406[is.na(A$A9406) & A$E8033000==-4]<-0
A$A9406[is.na(A$A9406) & A$E8033000==-3]<-0
A$A9406[is.na(A$A9406) & A$E8033000 >= 199300]<-0
A$A9406[is.na(A$A9406) & A$E8033100==0]<-0
A$A9406[is.na(A$A9406) & A$E8033100==-3]<-0
table(A$E8011406, useNA="always")
table(A$A9406, useNA="always")
A$A9407<-NA
A$A9407[A$E8011407==0]<-0
A$A9407[A$E8011407==99]<-0
A$A9407[A$E8011407>=1 & A$E8011407<99]<-A$E8011407[A$E8011407>=1 &
A$E8011407<99]
A$A9407[is.na(A$A9407) & A$E8033000==-4]<-0
A$A9407[is.na(A$A9407) & A$E8033000==-3]<-0
A$A9407[is.na(A$A9407) & A$E8033000 >= 199300]<-0
```

© 2023 Silver IA et al. *JAMA Network Open.*

```
A$A9407[is.na(A$A9407) & A$E8033100==0]<-0
A$A9407[is.na(A$A9407) & A$E8033100==-3]<-0
table(A$E8011407, useNA="always")
table(A$A9407, useNA="always")
A$A9408<-NA
A$A9408[A$E8011408==0]<-0
A$A9408[A$E8011408==99]<-0
A$A9408[A$E8011408>=1 & A$E8011408<99]<-A$E8011408[A$E8011408>=1 &
A$E8011408<99]
A$A9408[is.na(A$A9408) & A$E8033000==-4]<-0
A$A9408[is.na(A$A9408) & A$E8033000==-3]<-0
A$A9408[is.na(A$A9408) & A$E8033000 >= 199300]<-0
A$A9408[is.na(A$A9408) & A$E8033100==0]<-0
A$A9408[is.na(A$A9408) & A$E8033100==-3]<-0
table(A$E8011408, useNA="always")
table(A$A9408, useNA="always")
A$A9409<-NA
A$A9409[A$E8011409==0]<-0
A$A9409[A$E8011409==99]<-0
A$A9409[A$E8011409>=1 & A$E8011409<99]<-A$E8011409[A$E8011409>=1 &
A$E8011409<99]
A$A9409[is.na(A$A9409) & A$E8033000==-4]<-0
A$A9409[is.na(A$A9409) & A$E8033000==-3]<-0
A$A9409[is.na(A$A9409) & A$E8033000 >= 199300]<-0
A$A9409[is.na(A$A9409) & A$E8033100==0]<-0
A$A9409[is.na(A$A9409) & A$E8033100==-3]<-0
table(A$E8011409, useNA="always")
table(A$A9409, useNA="always")
A$A9410<-NA
A$A9410[A$E8011410==0]<-0
A$A9410[A$E8011410==99]<-0
A$A9410[A$E8011410>=1 & A$E8011410<99]<-A$E8011410[A$E8011410>=1 &
A$E8011410<99]
A$A9410[is.na(A$A9410) & A$E8033000==-4]<-0
A$A9410[is.na(A$A9410) & A$E8033000==-3]<-0
A$A9410[is.na(A$A9410) & A$E8033000 >= 199300]<-0
A$A9410[is.na(A$A9410) & A$E8033100==0]<-0
A$A9410[is.na(A$A9410) & A$E8033100==-3]<-0
table(A$E8011410, useNA="always")
table(A$A9410, useNA="always")
A$A9411<-NA
A$A9411[A$E8011411==0]<-0
A$A9411[A$E8011411==99]<-0
A$A9411[A$E8011411>=1 & A$E8011411<99]<-A$E8011411[A$E8011411>=1 &
A$E8011411<99]
A$A9411[is.na(A$A9411) & A$E8033000==-4]<-0
A$A9411[is.na(A$A9411) & A$E8033000==-3]<-0
A$A9411[is.na(A$A9411) & A$E8033000 >= 199300]<-0
A$A9411[is.na(A$A9411) & A$E8033100==0]<-0
A$A9411[is.na(A$A9411) & A$E8033100==-3]<-0
table(A$E8011411, useNA="always")
table(A$A9411, useNA="always")
A$A9412<-NA
A$A9412[A$E8011412==0]<-0
A$A9412[A$E8011412==99]<-0
```

© 2023 Silver IA et al. *JAMA Network Open.*

```
A$A9412[A$E8011412>=1 & A$E8011412<99]<-A$E8011412[A$E8011412>=1 &
A$E8011412<99]
A$A9412[is.na(A$A9412) & A$E8033000==-4]<-0
A$A9412[is.na(A$A9412) & A$E8033000==-3]<-0
A$A9412[is.na(A$A9412) & A$E8033000 >= 199300]<-0
A$A9412[is.na(A$A9412) & A$E8033100==0]<-0
A$A9412[is.na(A$A9412) & A$E8033100==-3]<-0
table(A$E8011412, useNA="always")
table(A$A9412, useNA="always")




A$A94<-
rowSums(data.frame(A$A9401,A$A9402,A$A9403,A$A9404,A$A9405,A$A9406,A$A9407,A$
A9408,A$A9409,A$A9410,A$A9411,A$A9412), na.rm = T)
table(A$A94)




### Arrest (1995)

table(A$E8011501, useNA="always")
table(A$E8011502)
table(A$E8011503)
table(A$E8011504)
table(A$E8011505)
table(A$E8011506)
table(A$E8011507)
table(A$E8011508)
table(A$E8011509)
table(A$E8011510)
table(A$E8011511)
table(A$E8011512)

A$A9501<-NA
A$A9501[A$E8011501==0]<-0
A$A9501[A$E8011501==99]<-0
A$A9501[A$E8011501>=1 & A$E8011501<99]<-A$E8011501[A$E8011501>=1 &
A$E8011501<99]
A$A9501[is.na(A$A9501) & A$E8033000==-4]<-0
A$A9501[is.na(A$A9501) & A$E8033000==-3]<-0
A$A9501[is.na(A$A9501) & A$E8033000 >= 199300]<-0
A$A9501[is.na(A$A9501) & A$E8033100==0]<-0
A$A9501[is.na(A$A9501) & A$E8033100==-3]<-0
table(A$E8011501, useNA="always")
table(A$A9501, useNA="always")
A$A9502<-NA
A$A9502[A$E8011502==0]<-0
A$A9502[A$E8011502==99]<-0
A$A9502[A$E8011502>=1 & A$E8011502<99]<-A$E8011502[A$E8011502>=1 &
A$E8011502<99]
A$A9502[is.na(A$A9502) & A$E8033000==-4]<-0
A$A9502[is.na(A$A9502) & A$E8033000==-3]<-0
A$A9502[is.na(A$A9502) & A$E8033000 >= 199300]<-0
```

© 2023 Silver IA et al. *JAMA Network Open.*

```
A$A9502[is.na(A$A9502) & A$E8033100==0]<-0
A$A9502[is.na(A$A9502) & A$E8033100==-3]<-0
table(A$E8011502, useNA="always")
table(A$A9502, useNA="always")
A$A9503<-NA
A$A9503[A$E8011503==0]<-0
A$A9503[A$E8011503==99]<-0
A$A9503[A$E8011503>=1 & A$E8011503<99]<-A$E8011503[A$E8011503>=1 &
A$E8011503<99]
A$A9503[is.na(A$A9503) & A$E8033000==-4]<-0
A$A9503[is.na(A$A9503) & A$E8033000==-3]<-0
A$A9503[is.na(A$A9503) & A$E8033000 >= 199300]<-0
A$A9503[is.na(A$A9503) & A$E8033100==0]<-0
A$A9503[is.na(A$A9503) & A$E8033100==-3]<-0
table(A$E8011503, useNA="always")
table(A$A9503, useNA="always")
A$A9504<-NA
A$A9504[A$E8011504==0]<-0
A$A9504[A$E8011504==99]<-0
A$A9504[A$E8011504>=1 & A$E8011504<99]<-A$E8011504[A$E8011504>=1 &
A$E8011504<99]
A$A9504[is.na(A$A9504) & A$E8033000==-4]<-0
A$A9504[is.na(A$A9504) & A$E8033000==-3]<-0
A$A9504[is.na(A$A9504) & A$E8033000 >= 199300]<-0
A$A9504[is.na(A$A9504) & A$E8033100==0]<-0
A$A9504[is.na(A$A9504) & A$E8033100==-3]<-0
table(A$E8011504, useNA="always")
table(A$A9504, useNA="always")
A$A9505<-NA
A$A9505[A$E8011505==0]<-0
A$A9505[A$E8011505==99]<-0
A$A9505[A$E8011505>=1 & A$E8011505<99]<-A$E8011505[A$E8011505>=1 &
A$E8011505<99]
A$A9505[is.na(A$A9505) & A$E8033000==-4]<-0
A$A9505[is.na(A$A9505) & A$E8033000==-3]<-0
A$A9505[is.na(A$A9505) & A$E8033000 >= 199300]<-0
A$A9505[is.na(A$A9505) & A$E8033100==0]<-0
A$A9505[is.na(A$A9505) & A$E8033100==-3]<-0
table(A$E8011505, useNA="always")
table(A$A9505, useNA="always")
A$A9506<-NA
A$A9506[A$E8011506==0]<-0
A$A9506[A$E8011506==99]<-0
A$A9506[A$E8011506>=1 & A$E8011506<99]<-A$E8011506[A$E8011506>=1 &
A$E8011506<99]
A$A9506[is.na(A$A9506) & A$E8033000==-4]<-0
A$A9506[is.na(A$A9506) & A$E8033000==-3]<-0
A$A9506[is.na(A$A9506) & A$E8033000 >= 199300]<-0
A$A9506[is.na(A$A9506) & A$E8033100==0]<-0
A$A9506[is.na(A$A9506) & A$E8033100==-3]<-0
table(A$E8011506, useNA="always")
table(A$A9506, useNA="always")
A$A9507<-NA
A$A9507[A$E8011507==0]<-0
A$A9507[A$E8011507==99]<-0
```

© 2023 Silver IA et al. *JAMA Network Open.*

```
A$A9507[A$E8011507>=1 & A$E8011507<99]<-A$E8011507[A$E8011507>=1 &
A$E8011507<99]
A$A9507[is.na(A$A9507) & A$E8033000==-4]<-0
A$A9507[is.na(A$A9507) & A$E8033000==-3]<-0
A$A9507[is.na(A$A9507) & A$E8033000 >= 199300]<-0
A$A9507[is.na(A$A9507) & A$E8033100==0]<-0
A$A9507[is.na(A$A9507) & A$E8033100==-3]<-0
table(A$E8011507, useNA="always")
table(A$A9507, useNA="always")
A$A9508<-NA
A$A9508[A$E8011508==0]<-0
A$A9508[A$E8011508==99]<-0
A$A9508[A$E8011508>=1 & A$E8011508<99]<-A$E8011508[A$E8011508>=1 &
A$E8011508<99]
A$A9508[is.na(A$A9508) & A$E8033000==-4]<-0
A$A9508[is.na(A$A9508) & A$E8033000==-3]<-0
A$A9508[is.na(A$A9508) & A$E8033000 >= 199300]<-0
A$A9508[is.na(A$A9508) & A$E8033100==0]<-0
A$A9508[is.na(A$A9508) & A$E8033100==-3]<-0
table(A$E8011508, useNA="always")
table(A$A9508, useNA="always")
A$A9509<-NA
A$A9509[A$E8011509==0]<-0
A$A9509[A$E8011509==99]<-0
A$A9509[A$E8011509>=1 & A$E8011509<99]<-A$E8011509[A$E8011509>=1 &
A$E8011509<99]
A$A9509[is.na(A$A9509) & A$E8033000==-4]<-0
A$A9509[is.na(A$A9509) & A$E8033000==-3]<-0
A$A9509[is.na(A$A9509) & A$E8033000 >= 199300]<-0
A$A9509[is.na(A$A9509) & A$E8033100==0]<-0
A$A9509[is.na(A$A9509) & A$E8033100==-3]<-0
table(A$E8011509, useNA="always")
table(A$A9509, useNA="always")
A$A9510<-NA
A$A9510[A$E8011510==0]<-0
A$A9510[A$E8011510==99]<-0
A$A9510[A$E8011510>=1 & A$E8011510<99]<-A$E8011510[A$E8011510>=1 &
A$E8011510<99]
A$A9510[is.na(A$A9510) & A$E8033000==-4]<-0
A$A9510[is.na(A$A9510) & A$E8033000==-3]<-0
A$A9510[is.na(A$A9510) & A$E8033000 >= 199300]<-0
A$A9510[is.na(A$A9510) & A$E8033100==0]<-0
A$A9510[is.na(A$A9510) & A$E8033100==-3]<-0
table(A$E8011510, useNA="always")
table(A$A9510, useNA="always")
A$A9511<-NA
A$A9511[A$E8011511==0]<-0
A$A9511[A$E8011511==99]<-0
A$A9511[A$E8011511>=1 & A$E8011511<99]<-A$E8011511[A$E8011511>=1 &
A$E8011511<99]
A$A9511[is.na(A$A9511) & A$E8033000==-4]<-0
A$A9511[is.na(A$A9511) & A$E8033000==-3]<-0
A$A9511[is.na(A$A9511) & A$E8033000 >= 199300]<-0
A$A9511[is.na(A$A9511) & A$E8033100==0]<-0
A$A9511[is.na(A$A9511) & A$E8033100==-3]<-0
table(A$E8011511, useNA="always")
```

© 2023 Silver IA et al. *JAMA Network Open.*

```
table(A$A9511, useNA="always")
A$A9512<-NA
A$A9512[A$E8011512==0]<-0
A$A9512[A$E8011512==99]<-0
A$A9512[A$E8011512>=1 & A$E8011512<99]<-A$E8011512[A$E8011512>=1 &
A$E8011512<99]
A$A9512[is.na(A$A9512) & A$E8033000==-4]<-0
A$A9512[is.na(A$A9512) & A$E8033000==-3]<-0
A$A9512[is.na(A$A9512) & A$E8033000 >= 199300]<-0
A$A9512[is.na(A$A9512) & A$E8033100==0]<-0
A$A9512[is.na(A$A9512) & A$E8033100==-3]<-0
table(A$E8011512, useNA="always")
table(A$A9512, useNA="always")



A$A95<-
rowSums(data.frame(A$A9501,A$A9502,A$A9503,A$A9504,A$A9505,A$A9506,A$A9507,A$
A9508,A$A9509,A$A9510,A$A9511,A$A9512), na.rm = T)
table(A$A95)



### Arrest (1996)

table(A$E8011601, useNA="always")
table(A$E8011602)
table(A$E8011603)
table(A$E8011604)
table(A$E8011605)
table(A$E8011606)
table(A$E8011607)
table(A$E8011608)
table(A$E8011609)
table(A$E8011610)
table(A$E8011611)
table(A$E8011612)

A$A9601<-NA
A$A9601[A$E8011601==0]<-0
A$A9601[A$E8011601==99]<-0
A$A9601[A$E8011601>=1 & A$E8011601<99]<-A$E8011601[A$E8011601>=1 &
A$E8011601<99]
A$A9601[is.na(A$A9601) & A$E8033000==-4]<-0
A$A9601[is.na(A$A9601) & A$E8033000==-3]<-0
A$A9601[is.na(A$A9601) & A$E8033000 >= 199300]<-0
A$A9601[is.na(A$A9601) & A$E8033100==0]<-0
A$A9601[is.na(A$A9601) & A$E8033100==-3]<-0
table(A$E8011601, useNA="always")
table(A$A9601, useNA="always")
A$A9602<-NA
A$A9602[A$E8011602==0]<-0
A$A9602[A$E8011602==99]<-0
```

© 2023 Silver IA et al. *JAMA Network Open.*

```
A$A9602[A$E8011602>=1 & A$E8011602<99]<-A$E8011602[A$E8011602>=1 &
A$E8011602<99]
A$A9602[is.na(A$A9602) & A$E8033000==-4]<-0
A$A9602[is.na(A$A9602) & A$E8033000==-3]<-0
A$A9602[is.na(A$A9602) & A$E8033000 >= 199300]<-0
A$A9602[is.na(A$A9602) & A$E8033100==0]<-0
A$A9602[is.na(A$A9602) & A$E8033100==-3]<-0
table(A$E8011602, useNA="always")
table(A$A9602, useNA="always")
A$A9603<-NA
A$A9603[A$E8011603==0]<-0
A$A9603[A$E8011603==99]<-0
A$A9603[A$E8011603>=1 & A$E8011603<99]<-A$E8011603[A$E8011603>=1 &
A$E8011603<99]
A$A9603[is.na(A$A9603) & A$E8033000==-4]<-0
A$A9603[is.na(A$A9603) & A$E8033000==-3]<-0
A$A9603[is.na(A$A9603) & A$E8033000 >= 199300]<-0
A$A9603[is.na(A$A9603) & A$E8033100==0]<-0
A$A9603[is.na(A$A9603) & A$E8033100==-3]<-0
table(A$E8011603, useNA="always")
table(A$A9603, useNA="always")
A$A9604<-NA
A$A9604[A$E8011604==0]<-0
A$A9604[A$E8011604==99]<-0
A$A9604[A$E8011604>=1 & A$E8011604<99]<-A$E8011604[A$E8011604>=1 &
A$E8011604<99]
A$A9604[is.na(A$A9604) & A$E8033000==-4]<-0
A$A9604[is.na(A$A9604) & A$E8033000==-3]<-0
A$A9604[is.na(A$A9604) & A$E8033000 >= 199300]<-0
A$A9604[is.na(A$A9604) & A$E8033100==0]<-0
A$A9604[is.na(A$A9604) & A$E8033100==-3]<-0
table(A$E8011604, useNA="always")
table(A$A9604, useNA="always")
A$A9605<-NA
A$A9605[A$E8011605==0]<-0
A$A9605[A$E8011605==99]<-0
A$A9605[A$E8011605>=1 & A$E8011605<99]<-A$E8011605[A$E8011605>=1 &
A$E8011605<99]
A$A9605[is.na(A$A9605) & A$E8033000==-4]<-0
A$A9605[is.na(A$A9605) & A$E8033000==-3]<-0
A$A9605[is.na(A$A9605) & A$E8033000 >= 199300]<-0
A$A9605[is.na(A$A9605) & A$E8033100==0]<-0
A$A9605[is.na(A$A9605) & A$E8033100==-3]<-0
table(A$E8011605, useNA="always")
table(A$A9605, useNA="always")
A$A9606<-NA
A$A9606[A$E8011606==0]<-0
A$A9606[A$E8011606==99]<-0
A$A9606[A$E8011606>=1 & A$E8011606<99]<-A$E8011606[A$E8011606>=1 &
A$E8011606<99]
A$A9606[is.na(A$A9606) & A$E8033000==-4]<-0
A$A9606[is.na(A$A9606) & A$E8033000==-3]<-0
A$A9606[is.na(A$A9606) & A$E8033000 >= 199300]<-0
A$A9606[is.na(A$A9606) & A$E8033100==0]<-0
A$A9606[is.na(A$A9606) & A$E8033100==-3]<-0
table(A$E8011606, useNA="always")
```

```
table(A$A9606, useNA="always")
A$A9607<-NA
A$A9607[A$E8011607==0]<-0
A$A9607[A$E8011607==99]<-0
A$A9607[A$E8011607>=1 & A$E8011607<99]<-A$E8011607[A$E8011607>=1 &
A$E8011607<99]
A$A9607[is.na(A$A9607) & A$E8033000==-4]<-0
A$A9607[is.na(A$A9607) & A$E8033000==-3]<-0
A$A9607[is.na(A$A9607) & A$E8033000 >= 199300]<-0
A$A9607[is.na(A$A9607) & A$E8033100==0]<-0
A$A9607[is.na(A$A9607) & A$E8033100==-3]<-0
table(A$E8011607, useNA="always")
table(A$A9607, useNA="always")
A$A9608<-NA
A$A9608[A$E8011608==0]<-0
A$A9608[A$E8011608==99]<-0
A$A9608[A$E8011608>=1 & A$E8011608<99]<-A$E8011608[A$E8011608>=1 &
A$E8011608<99]
A$A9608[is.na(A$A9608) & A$E8033000==-4]<-0
A$A9608[is.na(A$A9608) & A$E8033000==-3]<-0
A$A9608[is.na(A$A9608) & A$E8033000 >= 199300]<-0
A$A9608[is.na(A$A9608) & A$E8033100==0]<-0
A$A9608[is.na(A$A9608) & A$E8033100==-3]<-0
table(A$E8011608, useNA="always")
table(A$A9608, useNA="always")
A$A9609<-NA
A$A9609[A$E8011609==0]<-0
A$A9609[A$E8011609==99]<-0
A$A9609[A$E8011609>=1 & A$E8011609<99]<-A$E8011609[A$E8011609>=1 &
A$E8011609<99]
A$A9609[is.na(A$A9609) & A$E8033000==-4]<-0
A$A9609[is.na(A$A9609) & A$E8033000==-3]<-0
A$A9609[is.na(A$A9609) & A$E8033000 >= 199300]<-0
A$A9609[is.na(A$A9609) & A$E8033100==0]<-0
A$A9609[is.na(A$A9609) & A$E8033100==-3]<-0
table(A$E8011609, useNA="always")
table(A$A9609, useNA="always")
A$A9610<-NA
A$A9610[A$E8011610==0]<-0
A$A9610[A$E8011610==99]<-0
A$A9610[A$E8011610>=1 & A$E8011610<99]<-A$E8011610[A$E8011610>=1 &
A$E8011610<99]
A$A9610[is.na(A$A9610) & A$E8033000==-4]<-0
A$A9610[is.na(A$A9610) & A$E8033000==-3]<-0
A$A9610[is.na(A$A9610) & A$E8033000 >= 199300]<-0
A$A9610[is.na(A$A9610) & A$E8033100==0]<-0
A$A9610[is.na(A$A9610) & A$E8033100==-3]<-0
table(A$E8011610, useNA="always")
table(A$A9610, useNA="always")
A$A9611<-NA
A$A9611[A$E8011611==0]<-0
A$A9611[A$E8011611==99]<-0
A$A9611[A$E8011611>=1 & A$E8011611<99]<-A$E8011611[A$E8011611>=1 &
A$E8011611<99]
A$A9611[is.na(A$A9611) & A$E8033000==-4]<-0
A$A9611[is.na(A$A9611) & A$E8033000==-3]<-0
```

© 2023 Silver IA et al. *JAMA Network Open.*

```
A$A9611[is.na(A$A9611) & A$E8033000 >= 199300]<-0
A$A9611[is.na(A$A9611) & A$E8033100==0]<-0
A$A9611[is.na(A$A9611) & A$E8033100==-3]<-0
table(A$E8011611, useNA="always")
table(A$A9611, useNA="always")
A$A9612<-NA
A$A9612[A$E8011612==0]<-0
A$A9612[A$E8011612==99]<-0
A$A9612[A$E8011612>=1 & A$E8011612<99]<-A$E8011612[A$E8011612>=1 &
A$E8011612<99]
A$A9612[is.na(A$A9612) & A$E8033000==-4]<-0
A$A9612[is.na(A$A9612) & A$E8033000==-3]<-0
A$A9612[is.na(A$A9612) & A$E8033000 >= 199300]<-0
A$A9612[is.na(A$A9612) & A$E8033100==0]<-0
A$A9612[is.na(A$A9612) & A$E8033100==-3]<-0
table(A$E8011612, useNA="always")
table(A$A9612, useNA="always")




A$A96<-
rowSums(data.frame(A$A9601,A$A9602,A$A9603,A$A9604,A$A9605,A$A9606,A$A9607,A$
A9608,A$A9609,A$A9610,A$A9611,A$A9612), na.rm = T)
table(A$A96)




### Arrest (1997)

table(A$E8011701, useNA="always")
table(A$E8011702)
table(A$E8011703)
table(A$E8011704)
table(A$E8011705)
table(A$E8011706)
table(A$E8011707)
table(A$E8011708)
table(A$E8011709)
table(A$E8011710)
table(A$E8011711)
table(A$E8011712)

A$A9701<-NA
A$A9701[A$E8011701==0]<-0
A$A9701[A$E8011701==99]<-0
A$A9701[A$E8011701>=1 & A$E8011701<99]<-A$E8011701[A$E8011701>=1 &
A$E8011701<99]
A$A9701[is.na(A$A9701) & A$E8033000==-4]<-0
A$A9701[is.na(A$A9701) & A$E8033000==-3]<-0
A$A9701[is.na(A$A9701) & A$E8033000 >= 199300]<-0
A$A9701[is.na(A$A9701) & A$E8033100==0]<-0
A$A9701[is.na(A$A9701) & A$E8033100==-3]<-0
table(A$E8011701, useNA="always")
table(A$A9701, useNA="always")
```

© 2023 Silver IA et al. *JAMA Network Open.*

```
A$A9702<-NA
A$A9702[A$E8011702==0]<-0
A$A9702[A$E8011702==99]<-0
A$A9702[A$E8011702>=1 & A$E8011702<99]<-A$E8011702[A$E8011702>=1 &
A$E8011702<99]
A$A9702[is.na(A$A9702) & A$E8033000==-4]<-0
A$A9702[is.na(A$A9702) & A$E8033000==-3]<-0
A$A9702[is.na(A$A9702) & A$E8033000 >= 199300]<-0
A$A9702[is.na(A$A9702) & A$E8033100==0]<-0
A$A9702[is.na(A$A9702) & A$E8033100==-3]<-0
table(A$E8011702, useNA="always")
table(A$A9702, useNA="always")
A$A9703<-NA
A$A9703[A$E8011703==0]<-0
A$A9703[A$E8011703==99]<-0
A$A9703[A$E8011703>=1 & A$E8011703<99]<-A$E8011703[A$E8011703>=1 &
A$E8011703<99]
A$A9703[is.na(A$A9703) & A$E8033000==-4]<-0
A$A9703[is.na(A$A9703) & A$E8033000==-3]<-0
A$A9703[is.na(A$A9703) & A$E8033000 >= 199300]<-0
A$A9703[is.na(A$A9703) & A$E8033100==0]<-0
A$A9703[is.na(A$A9703) & A$E8033100==-3]<-0
table(A$E8011703, useNA="always")
table(A$A9703, useNA="always")
A$A9704<-NA
A$A9704[A$E8011704==0]<-0
A$A9704[A$E8011704==99]<-0
A$A9704[A$E8011704>=1 & A$E8011704<99]<-A$E8011704[A$E8011704>=1 &
A$E8011704<99]
A$A9704[is.na(A$A9704) & A$E8033000==-4]<-0
A$A9704[is.na(A$A9704) & A$E8033000==-3]<-0
A$A9704[is.na(A$A9704) & A$E8033000 >= 199300]<-0
A$A9704[is.na(A$A9704) & A$E8033100==0]<-0
A$A9704[is.na(A$A9704) & A$E8033100==-3]<-0
table(A$E8011704, useNA="always")
table(A$A9704, useNA="always")
A$A9705<-NA
A$A9705[A$E8011705==0]<-0
A$A9705[A$E8011705==99]<-0
A$A9705[A$E8011705>=1 & A$E8011705<99]<-A$E8011705[A$E8011705>=1 &
A$E8011705<99]
A$A9705[is.na(A$A9705) & A$E8033000==-4]<-0
A$A9705[is.na(A$A9705) & A$E8033000==-3]<-0
A$A9705[is.na(A$A9705) & A$E8033000 >= 199300]<-0
A$A9705[is.na(A$A9705) & A$E8033100==0]<-0
A$A9705[is.na(A$A9705) & A$E8033100==-3]<-0
table(A$E8011705, useNA="always")
table(A$A9705, useNA="always")
A$A9706<-NA
A$A9706[A$E8011706==0]<-0
A$A9706[A$E8011706==99]<-0
A$A9706[A$E8011706>=1 & A$E8011706<99]<-A$E8011706[A$E8011706>=1 &
A$E8011706<99]
A$A9706[is.na(A$A9706) & A$E8033000==-4]<-0
A$A9706[is.na(A$A9706) & A$E8033000==-3]<-0
A$A9706[is.na(A$A9706) & A$E8033000 >= 199300]<-0
```

```
A$A9706[is.na(A$A9706) & A$E8033100==0]<-0
A$A9706[is.na(A$A9706) & A$E8033100==-3]<-0
table(A$E8011706, useNA="always")
table(A$A9706, useNA="always")
A$A9707<-NA
A$A9707[A$E8011707==0]<-0
A$A9707[A$E8011707==99]<-0
A$A9707[A$E8011707>=1 & A$E8011707<99]<-A$E8011707[A$E8011707>=1 &
A$E8011707<99]
A$A9707[is.na(A$A9707) & A$E8033000==-4]<-0
A$A9707[is.na(A$A9707) & A$E8033000==-3]<-0
A$A9707[is.na(A$A9707) & A$E8033000 >= 199300]<-0
A$A9707[is.na(A$A9707) & A$E8033100==0]<-0
A$A9707[is.na(A$A9707) & A$E8033100==-3]<-0
table(A$E8011707, useNA="always")
table(A$A9707, useNA="always")
A$A9708<-NA
A$A9708[A$E8011708==0]<-0
A$A9708[A$E8011708==99]<-0
A$A9708[A$E8011708>=1 & A$E8011708<99]<-A$E8011708[A$E8011708>=1 &
A$E8011708<99]
A$A9708[is.na(A$A9708) & A$E8033000==-4]<-0
A$A9708[is.na(A$A9708) & A$E8033000==-3]<-0
A$A9708[is.na(A$A9708) & A$E8033000 >= 199300]<-0
A$A9708[is.na(A$A9708) & A$E8033100==0]<-0
A$A9708[is.na(A$A9708) & A$E8033100==-3]<-0
table(A$E8011708, useNA="always")
table(A$A9708, useNA="always")
A$A9709<-NA
A$A9709[A$E8011709==0]<-0
A$A9709[A$E8011709==99]<-0
A$A9709[A$E8011709>=1 & A$E8011709<99]<-A$E8011709[A$E8011709>=1 &
A$E8011709<99]
A$A9709[is.na(A$A9709) & A$E8033000==-4]<-0
A$A9709[is.na(A$A9709) & A$E8033000==-3]<-0
A$A9709[is.na(A$A9709) & A$E8033000 >= 199300]<-0
A$A9709[is.na(A$A9709) & A$E8033100==0]<-0
A$A9709[is.na(A$A9709) & A$E8033100==-3]<-0
table(A$E8011709, useNA="always")
table(A$A9709, useNA="always")
A$A9710<-NA
A$A9710[A$E8011710==0]<-0
A$A9710[A$E8011710==99]<-0
A$A9710[A$E8011710>=1 & A$E8011710<99]<-A$E8011710[A$E8011710>=1 &
A$E8011710<99]
A$A9710[is.na(A$A9710) & A$E8033000==-4]<-0
A$A9710[is.na(A$A9710) & A$E8033000==-3]<-0
A$A9710[is.na(A$A9710) & A$E8033000 >= 199300]<-0
A$A9710[is.na(A$A9710) & A$E8033100==0]<-0
A$A9710[is.na(A$A9710) & A$E8033100==-3]<-0
table(A$E8011710, useNA="always")
table(A$A9710, useNA="always")
A$A9711<-NA
A$A9711[A$E8011711==0]<-0
A$A9711[A$E8011711==99]<-0
```

© 2023 Silver IA et al. *JAMA Network Open.*

```
A$A9711[A$E8011711>=1 & A$E8011711<99]<-A$E8011711[A$E8011711>=1 &
A$E8011711<99]
A$A9711[is.na(A$A9711) & A$E8033000==-4]<-0
A$A9711[is.na(A$A9711) & A$E8033000==-3]<-0
A$A9711[is.na(A$A9711) & A$E8033000 >= 199300]<-0
A$A9711[is.na(A$A9711) & A$E8033100==0]<-0
A$A9711[is.na(A$A9711) & A$E8033100==-3]<-0
table(A$E8011711, useNA="always")
table(A$A9711, useNA="always")
A$A9712<-NA
A$A9712[A$E8011712==0]<-0
A$A9712[A$E8011712==99]<-0
A$A9712[A$E8011712>=1 & A$E8011712<99]<-A$E8011712[A$E8011712>=1 &
A$E8011712<99]
A$A9712[is.na(A$A9712) & A$E8033000==-4]<-0
A$A9712[is.na(A$A9712) & A$E8033000==-3]<-0
A$A9712[is.na(A$A9712) & A$E8033000 >= 199300]<-0
A$A9712[is.na(A$A9712) & A$E8033100==0]<-0
A$A9712[is.na(A$A9712) & A$E8033100==-3]<-0
table(A$E8011712, useNA="always")
table(A$A9712, useNA="always")




A$A97<-
rowSums(data.frame(A$A9701,A$A9702,A$A9703,A$A9704,A$A9705,A$A9706,A$A9707,A$
A9708,A$A9709,A$A9710,A$A9711,A$A9712), na.rm = T)
table(A$A97)




### Arrest (1998)

table(A$E8011801, useNA="always")
table(A$E8011802)
table(A$E8011803)
table(A$E8011804)
table(A$E8011805)
table(A$E8011806)
table(A$E8011807)
table(A$E8011808)
table(A$E8011809)
table(A$E8011810)
table(A$E8011811)
table(A$E8011812)

A$A9801<-NA
A$A9801[A$E8011801==0]<-0
A$A9801[A$E8011801==99]<-0
A$A9801[A$E8011801>=1 & A$E8011801<99]<-A$E8011801[A$E8011801>=1 &
A$E8011801<99]
A$A9801[is.na(A$A9801) & A$E8033000==-4]<-0
A$A9801[is.na(A$A9801) & A$E8033000==-3]<-0
A$A9801[is.na(A$A9801) & A$E8033000 >= 199300]<-0
```

```
A$A9801[is.na(A$A9801) & A$E8033100==0]<-0
A$A9801[is.na(A$A9801) & A$E8033100==-3]<-0
table(A$E8011801, useNA="always")
table(A$A9801, useNA="always")
A$A9802<-NA
A$A9802[A$E8011802==0]<-0
A$A9802[A$E8011802==99]<-0
A$A9802[A$E8011802>=1 & A$E8011802<99]<-A$E8011802[A$E8011802>=1 &
A$E8011802<99]
A$A9802[is.na(A$A9802) & A$E8033000==-4]<-0
A$A9802[is.na(A$A9802) & A$E8033000==-3]<-0
A$A9802[is.na(A$A9802) & A$E8033000 >= 199300]<-0
A$A9802[is.na(A$A9802) & A$E8033100==0]<-0
A$A9802[is.na(A$A9802) & A$E8033100==-3]<-0
table(A$E8011802, useNA="always")
table(A$A9802, useNA="always")
A$A9803<-NA
A$A9803[A$E8011803==0]<-0
A$A9803[A$E8011803==99]<-0
A$A9803[A$E8011803>=1 & A$E8011803<99]<-A$E8011803[A$E8011803>=1 &
A$E8011803<99]
A$A9803[is.na(A$A9803) & A$E8033000==-4]<-0
A$A9803[is.na(A$A9803) & A$E8033000==-3]<-0
A$A9803[is.na(A$A9803) & A$E8033000 >= 199300]<-0
A$A9803[is.na(A$A9803) & A$E8033100==0]<-0
A$A9803[is.na(A$A9803) & A$E8033100==-3]<-0
table(A$E8011803, useNA="always")
table(A$A9803, useNA="always")
A$A9804<-NA
A$A9804[A$E8011804==0]<-0
A$A9804[A$E8011804==99]<-0
A$A9804[A$E8011804>=1 & A$E8011804<99]<-A$E8011804[A$E8011804>=1 &
A$E8011804<99]
A$A9804[is.na(A$A9804) & A$E8033000==-4]<-0
A$A9804[is.na(A$A9804) & A$E8033000==-3]<-0
A$A9804[is.na(A$A9804) & A$E8033000 >= 199300]<-0
A$A9804[is.na(A$A9804) & A$E8033100==0]<-0
A$A9804[is.na(A$A9804) & A$E8033100==-3]<-0
table(A$E8011804, useNA="always")
table(A$A9804, useNA="always")
A$A9805<-NA
A$A9805[A$E8011805==0]<-0
A$A9805[A$E8011805==99]<-0
A$A9805[A$E8011805>=1 & A$E8011805<99]<-A$E8011805[A$E8011805>=1 &
A$E8011805<99]
A$A9805[is.na(A$A9805) & A$E8033000==-4]<-0
A$A9805[is.na(A$A9805) & A$E8033000==-3]<-0
A$A9805[is.na(A$A9805) & A$E8033000 >= 199300]<-0
A$A9805[is.na(A$A9805) & A$E8033100==0]<-0
A$A9805[is.na(A$A9805) & A$E8033100==-3]<-0
table(A$E8011805, useNA="always")
table(A$A9805, useNA="always")
A$A9806<-NA
A$A9806[A$E8011806==0]<-0
A$A9806[A$E8011806==99]<-0
```

© 2023 Silver IA et al. *JAMA Network Open.*

```
A$A9806[A$E8011806>=1 & A$E8011806<99]<-A$E8011806[A$E8011806>=1 &
A$E8011806<99]
A$A9806[is.na(A$A9806) & A$E8033000==-4]<-0
A$A9806[is.na(A$A9806) & A$E8033000==-3]<-0
A$A9806[is.na(A$A9806) & A$E8033000 >= 199300]<-0
A$A9806[is.na(A$A9806) & A$E8033100==0]<-0
A$A9806[is.na(A$A9806) & A$E8033100==-3]<-0
table(A$E8011806, useNA="always")
table(A$A9806, useNA="always")
A$A9807<-NA
A$A9807[A$E8011807==0]<-0
A$A9807[A$E8011807==99]<-0
A$A9807[A$E8011807>=1 & A$E8011807<99]<-A$E8011807[A$E8011807>=1 &
A$E8011807<99]
A$A9807[is.na(A$A9807) & A$E8033000==-4]<-0
A$A9807[is.na(A$A9807) & A$E8033000==-3]<-0
A$A9807[is.na(A$A9807) & A$E8033000 >= 199300]<-0
A$A9807[is.na(A$A9807) & A$E8033100==0]<-0
A$A9807[is.na(A$A9807) & A$E8033100==-3]<-0
table(A$E8011807, useNA="always")
table(A$A9807, useNA="always")
A$A9808<-NA
A$A9808[A$E8011808==0]<-0
A$A9808[A$E8011808==99]<-0
A$A9808[A$E8011808>=1 & A$E8011808<99]<-A$E8011808[A$E8011808>=1 &
A$E8011808<99]
A$A9808[is.na(A$A9808) & A$E8033000==-4]<-0
A$A9808[is.na(A$A9808) & A$E8033000==-3]<-0
A$A9808[is.na(A$A9808) & A$E8033000 >= 199300]<-0
A$A9808[is.na(A$A9808) & A$E8033100==0]<-0
A$A9808[is.na(A$A9808) & A$E8033100==-3]<-0
table(A$E8011808, useNA="always")
table(A$A9808, useNA="always")
A$A9809<-NA
A$A9809[A$E8011809==0]<-0
A$A9809[A$E8011809==99]<-0
A$A9809[A$E8011809>=1 & A$E8011809<99]<-A$E8011809[A$E8011809>=1 &
A$E8011809<99]
A$A9809[is.na(A$A9809) & A$E8033000==-4]<-0
A$A9809[is.na(A$A9809) & A$E8033000==-3]<-0
A$A9809[is.na(A$A9809) & A$E8033000 >= 199300]<-0
A$A9809[is.na(A$A9809) & A$E8033100==0]<-0
A$A9809[is.na(A$A9809) & A$E8033100==-3]<-0
table(A$E8011809, useNA="always")
table(A$A9809, useNA="always")
A$A9810<-NA
A$A9810[A$E8011810==0]<-0
A$A9810[A$E8011810==99]<-0
A$A9810[A$E8011810>=1 & A$E8011810<99]<-A$E8011810[A$E8011810>=1 &
A$E8011810<99]
A$A9810[is.na(A$A9810) & A$E8033000==-4]<-0
A$A9810[is.na(A$A9810) & A$E8033000==-3]<-0
A$A9810[is.na(A$A9810) & A$E8033000 >= 199300]<-0
A$A9810[is.na(A$A9810) & A$E8033100==0]<-0
A$A9810[is.na(A$A9810) & A$E8033100==-3]<-0
table(A$E8011810, useNA="always")
```

```
table(A$A9810, useNA="always")
A$A9811<-NA
A$A9811[A$E8011811==0]<-0
A$A9811[A$E8011811==99]<-0
A$A9811[A$E8011811>=1 & A$E8011811<99]<-A$E8011811[A$E8011811>=1 &
A$E8011811<99]
A$A9811[is.na(A$A9811) & A$E8033000==-4]<-0
A$A9811[is.na(A$A9811) & A$E8033000==-3]<-0
A$A9811[is.na(A$A9811) & A$E8033000 >= 199300]<-0
A$A9811[is.na(A$A9811) & A$E8033100==0]<-0
A$A9811[is.na(A$A9811) & A$E8033100==-3]<-0
table(A$E8011811, useNA="always")
table(A$A9811, useNA="always")
A$A9812<-NA
A$A9812[A$E8011812==0]<-0
A$A9812[A$E8011812==99]<-0
A$A9812[A$E8011812>=1 & A$E8011812<99]<-A$E8011812[A$E8011812>=1 &
A$E8011812<99]
A$A9812[is.na(A$A9812) & A$E8033000==-4]<-0
A$A9812[is.na(A$A9812) & A$E8033000==-3]<-0
A$A9812[is.na(A$A9812) & A$E8033000 >= 199300]<-0
A$A9812[is.na(A$A9812) & A$E8033100==0]<-0
A$A9812[is.na(A$A9812) & A$E8033100==-3]<-0
table(A$E8011812, useNA="always")
table(A$A9812, useNA="always")




A$A98<-
rowSums(data.frame(A$A9801,A$A9802,A$A9803,A$A9804,A$A9805,A$A9806,A$A9807,A$
A9808,A$A9809,A$A9810,A$A9811,A$A9812), na.rm = T)
table(A$A98)




### Arrest (1999)

table(A$E8011901, useNA="always")
table(A$E8011902)
table(A$E8011903)
table(A$E8011904)
table(A$E8011905)
table(A$E8011906)
table(A$E8011907)
table(A$E8011908)
table(A$E8011909)
table(A$E8011910)
table(A$E8011911)
table(A$E8011912)

A$A9901<-NA
A$A9901[A$E8011901==0]<-0
A$A9901[A$E8011901==99]<-0
```

© 2023 Silver IA et al. *JAMA Network Open.*

```
A$A9901[A$E8011901>=1 & A$E8011901<99]<-A$E8011901[A$E8011901>=1 &
A$E8011901<99]
A$A9901[is.na(A$A9901) & A$E8033000==-4]<-0
A$A9901[is.na(A$A9901) & A$E8033000==-3]<-0
A$A9901[is.na(A$A9901) & A$E8033000 >= 199300]<-0
A$A9901[is.na(A$A9901) & A$E8033100==0]<-0
A$A9901[is.na(A$A9901) & A$E8033100==-3]<-0
table(A$E8011901, useNA="always")
table(A$A9901, useNA="always")
A$A9902<-NA
A$A9902[A$E8011902==0]<-0
A$A9902[A$E8011902==99]<-0
A$A9902[A$E8011902>=1 & A$E8011902<99]<-A$E8011902[A$E8011902>=1 &
A$E8011902<99]
A$A9902[is.na(A$A9902) & A$E8033000==-4]<-0
A$A9902[is.na(A$A9902) & A$E8033000==-3]<-0
A$A9902[is.na(A$A9902) & A$E8033000 >= 199300]<-0
A$A9902[is.na(A$A9902) & A$E8033100==0]<-0
A$A9902[is.na(A$A9902) & A$E8033100==-3]<-0
table(A$E8011902, useNA="always")
table(A$A9902, useNA="always")
A$A9903<-NA
A$A9903[A$E8011903==0]<-0
A$A9903[A$E8011903==99]<-0
A$A9903[A$E8011903>=1 & A$E8011903<99]<-A$E8011903[A$E8011903>=1 &
A$E8011903<99]
A$A9903[is.na(A$A9903) & A$E8033000==-4]<-0
A$A9903[is.na(A$A9903) & A$E8033000==-3]<-0
A$A9903[is.na(A$A9903) & A$E8033000 >= 199300]<-0
A$A9903[is.na(A$A9903) & A$E8033100==0]<-0
A$A9903[is.na(A$A9903) & A$E8033100==-3]<-0
table(A$E8011903, useNA="always")
table(A$A9903, useNA="always")
A$A9904<-NA
A$A9904[A$E8011904==0]<-0
A$A9904[A$E8011904==99]<-0
A$A9904[A$E8011904>=1 & A$E8011904<99]<-A$E8011904[A$E8011904>=1 &
A$E8011904<99]
A$A9904[is.na(A$A9904) & A$E8033000==-4]<-0
A$A9904[is.na(A$A9904) & A$E8033000==-3]<-0
A$A9904[is.na(A$A9904) & A$E8033000 >= 199300]<-0
A$A9904[is.na(A$A9904) & A$E8033100==0]<-0
A$A9904[is.na(A$A9904) & A$E8033100==-3]<-0
table(A$E8011904, useNA="always")
table(A$A9904, useNA="always")
A$A9905<-NA
A$A9905[A$E8011905==0]<-0
A$A9905[A$E8011905==99]<-0
A$A9905[A$E8011905>=1 & A$E8011905<99]<-A$E8011905[A$E8011905>=1 &
A$E8011905<99]
A$A9905[is.na(A$A9905) & A$E8033000==-4]<-0
A$A9905[is.na(A$A9905) & A$E8033000==-3]<-0
A$A9905[is.na(A$A9905) & A$E8033000 >= 199300]<-0
A$A9905[is.na(A$A9905) & A$E8033100==0]<-0
A$A9905[is.na(A$A9905) & A$E8033100==-3]<-0
table(A$E8011905, useNA="always")
```

© 2023 Silver IA et al. *JAMA Network Open.*

```
table(A$A9905, useNA="always")
A$A9906<-NA
A$A9906[A$E8011906==0]<-0
A$A9906[A$E8011906==99]<-0
A$A9906[A$E8011906>=1 & A$E8011906<99]<-A$E8011906[A$E8011906>=1 &
A$E8011906<99]
A$A9906[is.na(A$A9906) & A$E8033000==-4]<-0
A$A9906[is.na(A$A9906) & A$E8033000==-3]<-0
A$A9906[is.na(A$A9906) & A$E8033000 >= 199300]<-0
A$A9906[is.na(A$A9906) & A$E8033100==0]<-0
A$A9906[is.na(A$A9906) & A$E8033100==-3]<-0
table(A$E8011906, useNA="always")
table(A$A9906, useNA="always")
A$A9907<-NA
A$A9907[A$E8011907==0]<-0
A$A9907[A$E8011907==99]<-0
A$A9907[A$E8011907>=1 & A$E8011907<99]<-A$E8011907[A$E8011907>=1 &
A$E8011907<99]
A$A9907[is.na(A$A9907) & A$E8033000==-4]<-0
A$A9907[is.na(A$A9907) & A$E8033000==-3]<-0
A$A9907[is.na(A$A9907) & A$E8033000 >= 199300]<-0
A$A9907[is.na(A$A9907) & A$E8033100==0]<-0
A$A9907[is.na(A$A9907) & A$E8033100==-3]<-0
table(A$E8011907, useNA="always")
table(A$A9907, useNA="always")
A$A9908<-NA
A$A9908[A$E8011908==0]<-0
A$A9908[A$E8011908==99]<-0
A$A9908[A$E8011908>=1 & A$E8011908<99]<-A$E8011908[A$E8011908>=1 &
A$E8011908<99]
A$A9908[is.na(A$A9908) & A$E8033000==-4]<-0
A$A9908[is.na(A$A9908) & A$E8033000==-3]<-0
A$A9908[is.na(A$A9908) & A$E8033000 >= 199300]<-0
A$A9908[is.na(A$A9908) & A$E8033100==0]<-0
A$A9908[is.na(A$A9908) & A$E8033100==-3]<-0
table(A$E8011908, useNA="always")
table(A$A9908, useNA="always")
A$A9909<-NA
A$A9909[A$E8011909==0]<-0
A$A9909[A$E8011909==99]<-0
A$A9909[A$E8011909>=1 & A$E8011909<99]<-A$E8011909[A$E8011909>=1 &
A$E8011909<99]
A$A9909[is.na(A$A9909) & A$E8033000==-4]<-0
A$A9909[is.na(A$A9909) & A$E8033000==-3]<-0
A$A9909[is.na(A$A9909) & A$E8033000 >= 199300]<-0
A$A9909[is.na(A$A9909) & A$E8033100==0]<-0
A$A9909[is.na(A$A9909) & A$E8033100==-3]<-0
table(A$E8011909, useNA="always")
table(A$A9909, useNA="always")
A$A9910<-NA
A$A9910[A$E8011910==0]<-0
A$A9910[A$E8011910==99]<-0
A$A9910[A$E8011910>=1 & A$E8011910<99]<-A$E8011910[A$E8011910>=1 &
A$E8011910<99]
A$A9910[is.na(A$A9910) & A$E8033000==-4]<-0
A$A9910[is.na(A$A9910) & A$E8033000==-3]<-0
```

© 2023 Silver IA et al. *JAMA Network Open.*

```
A$A9910[is.na(A$A9910) & A$E8033000 >= 199300]<-0
A$A9910[is.na(A$A9910) & A$E8033100==0]<-0
A$A9910[is.na(A$A9910) & A$E8033100==-3]<-0
table(A$E8011910, useNA="always")
table(A$A9910, useNA="always")
A$A9911<-NA
A$A9911[A$E8011911==0]<-0
A$A9911[A$E8011911==99]<-0
A$A9911[A$E8011911>=1 & A$E8011911<99]<-A$E8011911[A$E8011911>=1 &
A$E8011911<99]
A$A9911[is.na(A$A9911) & A$E8033000==-4]<-0
A$A9911[is.na(A$A9911) & A$E8033000==-3]<-0
A$A9911[is.na(A$A9911) & A$E8033000 >= 199300]<-0
A$A9911[is.na(A$A9911) & A$E8033100==0]<-0
A$A9911[is.na(A$A9911) & A$E8033100==-3]<-0
table(A$E8011911, useNA="always")
table(A$A9911, useNA="always")
A$A9912<-NA
A$A9912[A$E8011912==0]<-0
A$A9912[A$E8011912==99]<-0
A$A9912[A$E8011912>=1 & A$E8011912<99]<-A$E8011912[A$E8011912>=1 &
A$E8011912<99]
A$A9912[is.na(A$A9912) & A$E8033000==-4]<-0
A$A9912[is.na(A$A9912) & A$E8033000==-3]<-0
A$A9912[is.na(A$A9912) & A$E8033000 >= 199300]<-0
A$A9912[is.na(A$A9912) & A$E8033100==0]<-0
A$A9912[is.na(A$A9912) & A$E8033100==-3]<-0
table(A$E8011912, useNA="always")
table(A$A9912, useNA="always")




A$A99<-
rowSums(data.frame(A$A9901,A$A9902,A$A9903,A$A9904,A$A9905,A$A9906,A$A9907,A$
A9908,A$A9909,A$A9910,A$A9911,A$A9912), na.rm = T)
table(A$A99)




### Arrest (2000)

table(A$E8012001, useNA="always")
table(A$E8012002)
table(A$E8012003)
table(A$E8012004)
table(A$E8012005)
table(A$E8012006)
table(A$E8012007)
table(A$E8012008)
table(A$E8012009)
table(A$E8012010)
table(A$E8012011)
table(A$E8012012)
```

© 2023 Silver IA et al. *JAMA Network Open.*

```
A$A0001<-NA
A$A0001[A$E8012001==0]<-0
A$A0001[A$E8012001==99]<-0
A$A0001[A$E8012001>=1 & A$E8012001<99]<-A$E8012001[A$E8012001>=1 &
A$E8012001<99]
A$A0001[is.na(A$A0001) & A$E8033000==-4]<-0
A$A0001[is.na(A$A0001) & A$E8033000==-3]<-0
A$A0001[is.na(A$A0001) & A$E8033000 >= 199300]<-0
A$A0001[is.na(A$A0001) & A$E8033100==0]<-0
A$A0001[is.na(A$A0001) & A$E8033100==-3]<-0
table(A$E8012001, useNA="always")
table(A$A0001, useNA="always")
A$A0002<-NA
A$A0002[A$E8012002==0]<-0
A$A0002[A$E8012002==99]<-0
A$A0002[A$E8012002>=1 & A$E8012002<99]<-A$E8012002[A$E8012002>=1 &
A$E8012002<99]
A$A0002[is.na(A$A0002) & A$E8033000==-4]<-0
A$A0002[is.na(A$A0002) & A$E8033000==-3]<-0
A$A0002[is.na(A$A0002) & A$E8033000 >= 199300]<-0
A$A0002[is.na(A$A0002) & A$E8033100==0]<-0
A$A0002[is.na(A$A0002) & A$E8033100==-3]<-0
table(A$E8012002, useNA="always")
table(A$A0002, useNA="always")
A$A0003<-NA
A$A0003[A$E8012003==0]<-0
A$A0003[A$E8012003==99]<-0
A$A0003[A$E8012003>=1 & A$E8012003<99]<-A$E8012003[A$E8012003>=1 &
A$E8012003<99]
A$A0003[is.na(A$A0003) & A$E8033000==-4]<-0
A$A0003[is.na(A$A0003) & A$E8033000==-3]<-0
A$A0003[is.na(A$A0003) & A$E8033000 >= 199300]<-0
A$A0003[is.na(A$A0003) & A$E8033100==0]<-0
A$A0003[is.na(A$A0003) & A$E8033100==-3]<-0
table(A$E8012003, useNA="always")
table(A$A0003, useNA="always")
A$A0004<-NA
A$A0004[A$E8012004==0]<-0
A$A0004[A$E8012004==99]<-0
A$A0004[A$E8012004>=1 & A$E8012004<99]<-A$E8012004[A$E8012004>=1 &
A$E8012004<99]
A$A0004[is.na(A$A0004) & A$E8033000==-4]<-0
A$A0004[is.na(A$A0004) & A$E8033000==-3]<-0
A$A0004[is.na(A$A0004) & A$E8033000 >= 199300]<-0
A$A0004[is.na(A$A0004) & A$E8033100==0]<-0
A$A0004[is.na(A$A0004) & A$E8033100==-3]<-0
table(A$E8012004, useNA="always")
table(A$A0004, useNA="always")
A$A0005<-NA
A$A0005[A$E8012005==0]<-0
A$A0005[A$E8012005==99]<-0
A$A0005[A$E8012005>=1 & A$E8012005<99]<-A$E8012005[A$E8012005>=1 &
A$E8012005<99]
A$A0005[is.na(A$A0005) & A$E8033000==-4]<-0
A$A0005[is.na(A$A0005) & A$E8033000==-3]<-0
A$A0005[is.na(A$A0005) & A$E8033000 >= 199300]<-0
```

```
A$A0005[is.na(A$A0005) & A$E8033100==0]<-0
A$A0005[is.na(A$A0005) & A$E8033100==-3]<-0
table(A$E8012005, useNA="always")
table(A$A0005, useNA="always")
A$A0006<-NA
A$A0006[A$E8012006==0]<-0
A$A0006[A$E8012006==99]<-0
A$A0006[A$E8012006>=1 & A$E8012006<99]<-A$E8012006[A$E8012006>=1 &
A$E8012006<99]
A$A0006[is.na(A$A0006) & A$E8033000==-4]<-0
A$A0006[is.na(A$A0006) & A$E8033000==-3]<-0
A$A0006[is.na(A$A0006) & A$E8033000 >= 199300]<-0
A$A0006[is.na(A$A0006) & A$E8033100==0]<-0
A$A0006[is.na(A$A0006) & A$E8033100==-3]<-0
table(A$E8012006, useNA="always")
table(A$A0006, useNA="always")
A$A0007<-NA
A$A0007[A$E8012007==0]<-0
A$A0007[A$E8012007==99]<-0
A$A0007[A$E8012007>=1 & A$E8012007<99]<-A$E8012007[A$E8012007>=1 &
A$E8012007<99]
A$A0007[is.na(A$A0007) & A$E8033000==-4]<-0
A$A0007[is.na(A$A0007) & A$E8033000==-3]<-0
A$A0007[is.na(A$A0007) & A$E8033000 >= 199300]<-0
A$A0007[is.na(A$A0007) & A$E8033100==0]<-0
A$A0007[is.na(A$A0007) & A$E8033100==-3]<-0
table(A$E8012007, useNA="always")
table(A$A0007, useNA="always")
A$A0008<-NA
A$A0008[A$E8012008==0]<-0
A$A0008[A$E8012008==99]<-0
A$A0008[A$E8012008>=1 & A$E8012008<99]<-A$E8012008[A$E8012008>=1 &
A$E8012008<99]
A$A0008[is.na(A$A0008) & A$E8033000==-4]<-0
A$A0008[is.na(A$A0008) & A$E8033000==-3]<-0
A$A0008[is.na(A$A0008) & A$E8033000 >= 199300]<-0
A$A0008[is.na(A$A0008) & A$E8033100==0]<-0
A$A0008[is.na(A$A0008) & A$E8033100==-3]<-0
table(A$E8012008, useNA="always")
table(A$A0008, useNA="always")
A$A0009<-NA
A$A0009[A$E8012009==0]<-0
A$A0009[A$E8012009==99]<-0
A$A0009[A$E8012009>=1 & A$E8012009<99]<-A$E8012009[A$E8012009>=1 &
A$E8012009<99]
A$A0009[is.na(A$A0009) & A$E8033000==-4]<-0
A$A0009[is.na(A$A0009) & A$E8033000==-3]<-0
A$A0009[is.na(A$A0009) & A$E8033000 >= 199300]<-0
A$A0009[is.na(A$A0009) & A$E8033100==0]<-0
A$A0009[is.na(A$A0009) & A$E8033100==-3]<-0
table(A$E8012009, useNA="always")
table(A$A0009, useNA="always")
A$A0010<-NA
A$A0010[A$E8012010==0]<-0
A$A0010[A$E8012010==99]<-0
```

```
A$A0010[A$E8012010>=1 & A$E8012010<99]<-A$E8012010[A$E8012010>=1 &
A$E8012010<99]
A$A0010[is.na(A$A0010) & A$E8033000==-4]<-0
A$A0010[is.na(A$A0010) & A$E8033000==-3]<-0
A$A0010[is.na(A$A0010) & A$E8033000 >= 199300]<-0
A$A0010[is.na(A$A0010) & A$E8033100==0]<-0
A$A0010[is.na(A$A0010) & A$E8033100==-3]<-0
table(A$E8012010, useNA="always")
table(A$A0010, useNA="always")
A$A0011<-NA
A$A0011[A$E8012011==0]<-0
A$A0011[A$E8012011==99]<-0
A$A0011[A$E8012011>=1 & A$E8012011<99]<-A$E8012011[A$E8012011>=1 &
A$E8012011<99]
A$A0011[is.na(A$A0011) & A$E8033000==-4]<-0
A$A0011[is.na(A$A0011) & A$E8033000==-3]<-0
A$A0011[is.na(A$A0011) & A$E8033000 >= 199300]<-0
A$A0011[is.na(A$A0011) & A$E8033100==0]<-0
A$A0011[is.na(A$A0011) & A$E8033100==-3]<-0
table(A$E8012011, useNA="always")
table(A$A0011, useNA="always")
A$A0012<-NA
A$A0012[A$E8012012==0]<-0
A$A0012[A$E8012012==99]<-0
A$A0012[A$E8012012>=1 & A$E8012012<99]<-A$E8012012[A$E8012012>=1 &
A$E8012012<99]
A$A0012[is.na(A$A0012) & A$E8033000==-4]<-0
A$A0012[is.na(A$A0012) & A$E8033000==-3]<-0
A$A0012[is.na(A$A0012) & A$E8033000 >= 199300]<-0
A$A0012[is.na(A$A0012) & A$E8033100==0]<-0
A$A0012[is.na(A$A0012) & A$E8033100==-3]<-0
table(A$E8012012, useNA="always")
table(A$A0012, useNA="always")




A$A00<-
rowSums(data.frame(A$A0001,A$A0002,A$A0003,A$A0004,A$A0005,A$A0006,A$A0007,A$
A0008,A$A0009,A$A0010,A$A0011,A$A0012), na.rm = T)
table(A$A00)



### Arrest (2001)

table(A$E8012101, useNA="always")
table(A$E8012102)
table(A$E8012103)
table(A$E8012104)
table(A$E8012105)
table(A$E8012106)
table(A$E8012107)
table(A$E8012108)
table(A$E8012109)
table(A$E8012110)
```

© 2023 Silver IA et al. *JAMA Network Open.*

```
table(A$E8012111)
table(A$E8012112)

A$A0101<-NA
A$A0101[A$E8012101==0]<-0
A$A0101[A$E8012101==99]<-0
A$A0101[A$E8012101>=1 & A$E8012101<99]<-A$E8012101[A$E8012101>=1 &
A$E8012101<99]
A$A0101[is.na(A$A0101) & A$E8033000==-4]<-0
A$A0101[is.na(A$A0101) & A$E8033000==-3]<-0
A$A0101[is.na(A$A0101) & A$E8033000 >= 199300]<-0
A$A0101[is.na(A$A0101) & A$E8033100==0]<-0
A$A0101[is.na(A$A0101) & A$E8033100==-3]<-0
table(A$E8012101, useNA="always")
table(A$A0101, useNA="always")
A$A0102<-NA
A$A0102[A$E8012102==0]<-0
A$A0102[A$E8012102==99]<-0
A$A0102[A$E8012102>=1 & A$E8012102<99]<-A$E8012102[A$E8012102>=1 &
A$E8012102<99]
A$A0102[is.na(A$A0102) & A$E8033000==-4]<-0
A$A0102[is.na(A$A0102) & A$E8033000==-3]<-0
A$A0102[is.na(A$A0102) & A$E8033000 >= 199300]<-0
A$A0102[is.na(A$A0102) & A$E8033100==0]<-0
A$A0102[is.na(A$A0102) & A$E8033100==-3]<-0
table(A$E8012102, useNA="always")
table(A$A0102, useNA="always")
A$A0103<-NA
A$A0103[A$E8012103==0]<-0
A$A0103[A$E8012103==99]<-0
A$A0103[A$E8012103>=1 & A$E8012103<99]<-A$E8012103[A$E8012103>=1 &
A$E8012103<99]
A$A0103[is.na(A$A0103) & A$E8033000==-4]<-0
A$A0103[is.na(A$A0103) & A$E8033000==-3]<-0
A$A0103[is.na(A$A0103) & A$E8033000 >= 199300]<-0
A$A0103[is.na(A$A0103) & A$E8033100==0]<-0
A$A0103[is.na(A$A0103) & A$E8033100==-3]<-0
table(A$E8012103, useNA="always")
table(A$A0103, useNA="always")
A$A0104<-NA
A$A0104[A$E8012104==0]<-0
A$A0104[A$E8012104==99]<-0
A$A0104[A$E8012104>=1 & A$E8012104<99]<-A$E8012104[A$E8012104>=1 &
A$E8012104<99]
A$A0104[is.na(A$A0104) & A$E8033000==-4]<-0
A$A0104[is.na(A$A0104) & A$E8033000==-3]<-0
A$A0104[is.na(A$A0104) & A$E8033000 >= 199300]<-0
A$A0104[is.na(A$A0104) & A$E8033100==0]<-0
A$A0104[is.na(A$A0104) & A$E8033100==-3]<-0
table(A$E8012104, useNA="always")
table(A$A0104, useNA="always")
A$A0105<-NA
A$A0105[A$E8012105==0]<-0
A$A0105[A$E8012105==99]<-0
A$A0105[A$E8012105>=1 & A$E8012105<99]<-A$E8012105[A$E8012105>=1 &
A$E8012105<99]
```

© 2023 Silver IA et al. *JAMA Network Open.*

```
A$A0105[is.na(A$A0105) & A$E8033000==-4]<-0
A$A0105[is.na(A$A0105) & A$E8033000==-3]<-0
A$A0105[is.na(A$A0105) & A$E8033000 >= 199300]<-0
A$A0105[is.na(A$A0105) & A$E8033100==0]<-0
A$A0105[is.na(A$A0105) & A$E8033100==-3]<-0
table(A$E8012105, useNA="always")
table(A$A0105, useNA="always")
A$A0106<-NA
A$A0106[A$E8012106==0]<-0
A$A0106[A$E8012106==99]<-0
A$A0106[A$E8012106>=1 & A$E8012106<99]<-A$E8012106[A$E8012106>=1 &
A$E8012106<99]
A$A0106[is.na(A$A0106) & A$E8033000==-4]<-0
A$A0106[is.na(A$A0106) & A$E8033000==-3]<-0
A$A0106[is.na(A$A0106) & A$E8033000 >= 199300]<-0
A$A0106[is.na(A$A0106) & A$E8033100==0]<-0
A$A0106[is.na(A$A0106) & A$E8033100==-3]<-0
table(A$E8012106, useNA="always")
table(A$A0106, useNA="always")
A$A0107<-NA
A$A0107[A$E8012107==0]<-0
A$A0107[A$E8012107==99]<-0
A$A0107[A$E8012107>=1 & A$E8012107<99]<-A$E8012107[A$E8012107>=1 &
A$E8012107<99]
A$A0107[is.na(A$A0107) & A$E8033000==-4]<-0
A$A0107[is.na(A$A0107) & A$E8033000==-3]<-0
A$A0107[is.na(A$A0107) & A$E8033000 >= 199300]<-0
A$A0107[is.na(A$A0107) & A$E8033100==0]<-0
A$A0107[is.na(A$A0107) & A$E8033100==-3]<-0
table(A$E8012107, useNA="always")
table(A$A0107, useNA="always")
A$A0108<-NA
A$A0108[A$E8012108==0]<-0
A$A0108[A$E8012108==99]<-0
A$A0108[A$E8012108>=1 & A$E8012108<99]<-A$E8012108[A$E8012108>=1 &
A$E8012108<99]
A$A0108[is.na(A$A0108) & A$E8033000==-4]<-0
A$A0108[is.na(A$A0108) & A$E8033000==-3]<-0
A$A0108[is.na(A$A0108) & A$E8033000 >= 199300]<-0
A$A0108[is.na(A$A0108) & A$E8033100==0]<-0
A$A0108[is.na(A$A0108) & A$E8033100==-3]<-0
table(A$E8012108, useNA="always")
table(A$A0108, useNA="always")
A$A0109<-NA
A$A0109[A$E8012109==0]<-0
A$A0109[A$E8012109==99]<-0
A$A0109[A$E8012109>=1 & A$E8012109<99]<-A$E8012109[A$E8012109>=1 &
A$E8012109<99]
A$A0109[is.na(A$A0109) & A$E8033000==-4]<-0
A$A0109[is.na(A$A0109) & A$E8033000==-3]<-0
A$A0109[is.na(A$A0109) & A$E8033000 >= 199300]<-0
A$A0109[is.na(A$A0109) & A$E8033100==0]<-0
A$A0109[is.na(A$A0109) & A$E8033100==-3]<-0
table(A$E8012109, useNA="always")
table(A$A0109, useNA="always")
A$A0110<-NA
```

© 2023 Silver IA et al. *JAMA Network Open.*

```
A$A0110[A$E8012110==0]<-0
A$A0110[A$E8012110==99]<-0
A$A0110[A$E8012110>=1 & A$E8012110<99]<-A$E8012110[A$E8012110>=1 &
A$E8012110<99]
A$A0110[is.na(A$A0110) & A$E8033000==-4]<-0
A$A0110[is.na(A$A0110) & A$E8033000==-3]<-0
A$A0110[is.na(A$A0110) & A$E8033000 >= 199300]<-0
A$A0110[is.na(A$A0110) & A$E8033100==0]<-0
A$A0110[is.na(A$A0110) & A$E8033100==-3]<-0
table(A$E8012110, useNA="always")
table(A$A0110, useNA="always")
A$A0111<-NA
A$A0111[A$E8012111==0]<-0
A$A0111[A$E8012111==99]<-0
A$A0111[A$E8012111>=1 & A$E8012111<99]<-A$E8012111[A$E8012111>=1 &
A$E8012111<99]
A$A0111[is.na(A$A0111) & A$E8033000==-4]<-0
A$A0111[is.na(A$A0111) & A$E8033000==-3]<-0
A$A0111[is.na(A$A0111) & A$E8033000 >= 199300]<-0
A$A0111[is.na(A$A0111) & A$E8033100==0]<-0
A$A0111[is.na(A$A0111) & A$E8033100==-3]<-0
table(A$E8012111, useNA="always")
table(A$A0111, useNA="always")
A$A0112<-NA
A$A0112[A$E8012112==0]<-0
A$A0112[A$E8012112==99]<-0
A$A0112[A$E8012112>=1 & A$E8012112<99]<-A$E8012112[A$E8012112>=1 &
A$E8012112<99]
A$A0112[is.na(A$A0112) & A$E8033000==-4]<-0
A$A0112[is.na(A$A0112) & A$E8033000==-3]<-0
A$A0112[is.na(A$A0112) & A$E8033000 >= 199300]<-0
A$A0112[is.na(A$A0112) & A$E8033100==0]<-0
A$A0112[is.na(A$A0112) & A$E8033100==-3]<-0
table(A$E8012112, useNA="always")
table(A$A0112, useNA="always")



A$A01<-
rowSums(data.frame(A$A0101,A$A0102,A$A0103,A$A0104,A$A0105,A$A0106,A$A0107,A$
A0108,A$A0109,A$A0110,A$A0111,A$A0112), na.rm = T)
table(A$A01)



### Arrest (2002)

table(A$E8012201, useNA="always")
table(A$E8012202)
table(A$E8012203)
table(A$E8012204)
table(A$E8012205)
table(A$E8012206)
table(A$E8012207)
table(A$E8012208)
```

© 2023 Silver IA et al. *JAMA Network Open.*

```
table(A$E8012209)
table(A$E8012210)
table(A$E8012211)
table(A$E8012212)

A$A0201<-NA
A$A0201[A$E8012201==0]<-0
A$A0201[A$E8012201==99]<-0
A$A0201[A$E8012201>=1 & A$E8012201<99]<-A$E8012201[A$E8012201>=1 &
A$E8012201<99]
A$A0201[is.na(A$A0201) & A$E8033000==-4]<-0
A$A0201[is.na(A$A0201) & A$E8033000==-3]<-0
A$A0201[is.na(A$A0201) & A$E8033000 >= 199300]<-0
A$A0201[is.na(A$A0201) & A$E8033100==0]<-0
A$A0201[is.na(A$A0201) & A$E8033100==-3]<-0
table(A$E8012201, useNA="always")
table(A$A0201, useNA="always")
A$A0202<-NA
A$A0202[A$E8012202==0]<-0
A$A0202[A$E8012202==99]<-0
A$A0202[A$E8012202>=1 & A$E8012202<99]<-A$E8012202[A$E8012202>=1 &
A$E8012202<99]
A$A0202[is.na(A$A0202) & A$E8033000==-4]<-0
A$A0202[is.na(A$A0202) & A$E8033000==-3]<-0
A$A0202[is.na(A$A0202) & A$E8033000 >= 199300]<-0
A$A0202[is.na(A$A0202) & A$E8033100==0]<-0
A$A0202[is.na(A$A0202) & A$E8033100==-3]<-0
table(A$E8012202, useNA="always")
table(A$A0202, useNA="always")
A$A0203<-NA
A$A0203[A$E8012203==0]<-0
A$A0203[A$E8012203==99]<-0
A$A0203[A$E8012203>=1 & A$E8012203<99]<-A$E8012203[A$E8012203>=1 &
A$E8012203<99]
A$A0203[is.na(A$A0203) & A$E8033000==-4]<-0
A$A0203[is.na(A$A0203) & A$E8033000==-3]<-0
A$A0203[is.na(A$A0203) & A$E8033000 >= 199300]<-0
A$A0203[is.na(A$A0203) & A$E8033100==0]<-0
A$A0203[is.na(A$A0203) & A$E8033100==-3]<-0
table(A$E8012203, useNA="always")
table(A$A0203, useNA="always")
A$A0204<-NA
A$A0204[A$E8012204==0]<-0
A$A0204[A$E8012204==99]<-0
A$A0204[A$E8012204>=1 & A$E8012204<99]<-A$E8012204[A$E8012204>=1 &
A$E8012204<99]
A$A0204[is.na(A$A0204) & A$E8033000==-4]<-0
A$A0204[is.na(A$A0204) & A$E8033000==-3]<-0
A$A0204[is.na(A$A0204) & A$E8033000 >= 199300]<-0
A$A0204[is.na(A$A0204) & A$E8033100==0]<-0
A$A0204[is.na(A$A0204) & A$E8033100==-3]<-0
table(A$E8012204, useNA="always")
table(A$A0204, useNA="always")
A$A0205<-NA
A$A0205[A$E8012205==0]<-0
A$A0205[A$E8012205==99]<-0
```

```
A$A0205[A$E8012205>=1 & A$E8012205<99]<-A$E8012205[A$E8012205>=1 &
A$E8012205<99]
A$A0205[is.na(A$A0205) & A$E8033000==-4]<-0
A$A0205[is.na(A$A0205) & A$E8033000==-3]<-0
A$A0205[is.na(A$A0205) & A$E8033000 >= 199300]<-0
A$A0205[is.na(A$A0205) & A$E8033100==0]<-0
A$A0205[is.na(A$A0205) & A$E8033100==-3]<-0
table(A$E8012205, useNA="always")
table(A$A0205, useNA="always")
A$A0206<-NA
A$A0206[A$E8012206==0]<-0
A$A0206[A$E8012206==99]<-0
A$A0206[A$E8012206>=1 & A$E8012206<99]<-A$E8012206[A$E8012206>=1 &
A$E8012206<99]
A$A0206[is.na(A$A0206) & A$E8033000==-4]<-0
A$A0206[is.na(A$A0206) & A$E8033000==-3]<-0
A$A0206[is.na(A$A0206) & A$E8033000 >= 199300]<-0
A$A0206[is.na(A$A0206) & A$E8033100==0]<-0
A$A0206[is.na(A$A0206) & A$E8033100==-3]<-0
table(A$E8012206, useNA="always")
table(A$A0206, useNA="always")
A$A0207<-NA
A$A0207[A$E8012207==0]<-0
A$A0207[A$E8012207==99]<-0
A$A0207[A$E8012207>=1 & A$E8012207<99]<-A$E8012207[A$E8012207>=1 &
A$E8012207<99]
A$A0207[is.na(A$A0207) & A$E8033000==-4]<-0
A$A0207[is.na(A$A0207) & A$E8033000==-3]<-0
A$A0207[is.na(A$A0207) & A$E8033000 >= 199300]<-0
A$A0207[is.na(A$A0207) & A$E8033100==0]<-0
A$A0207[is.na(A$A0207) & A$E8033100==-3]<-0
table(A$E8012207, useNA="always")
table(A$A0207, useNA="always")
A$A0208<-NA
A$A0208[A$E8012208==0]<-0
A$A0208[A$E8012208==99]<-0
A$A0208[A$E8012208>=1 & A$E8012208<99]<-A$E8012208[A$E8012208>=1 &
A$E8012208<99]
A$A0208[is.na(A$A0208) & A$E8033000==-4]<-0
A$A0208[is.na(A$A0208) & A$E8033000==-3]<-0
A$A0208[is.na(A$A0208) & A$E8033000 >= 199300]<-0
A$A0208[is.na(A$A0208) & A$E8033100==0]<-0
A$A0208[is.na(A$A0208) & A$E8033100==-3]<-0
table(A$E8012208, useNA="always")
table(A$A0208, useNA="always")
A$A0209<-NA
A$A0209[A$E8012209==0]<-0
A$A0209[A$E8012209==99]<-0
A$A0209[A$E8012209>=1 & A$E8012209<99]<-A$E8012209[A$E8012209>=1 &
A$E8012209<99]
A$A0209[is.na(A$A0209) & A$E8033000==-4]<-0
A$A0209[is.na(A$A0209) & A$E8033000==-3]<-0
A$A0209[is.na(A$A0209) & A$E8033000 >= 199300]<-0
A$A0209[is.na(A$A0209) & A$E8033100==0]<-0
A$A0209[is.na(A$A0209) & A$E8033100==-3]<-0
table(A$E8012209, useNA="always")
```

© 2023 Silver IA et al. *JAMA Network Open.*

```
table(A$A0209, useNA="always")
A$A0210<-NA
A$A0210[A$E8012210==0]<-0
A$A0210[A$E8012210==99]<-0
A$A0210[A$E8012210>=1 & A$E8012210<99]<-A$E8012210[A$E8012210>=1 &
A$E8012210<99]
A$A0210[is.na(A$A0210) & A$E8033000==-4]<-0
A$A0210[is.na(A$A0210) & A$E8033000==-3]<-0
A$A0210[is.na(A$A0210) & A$E8033000 >= 199300]<-0
A$A0210[is.na(A$A0210) & A$E8033100==0]<-0
A$A0210[is.na(A$A0210) & A$E8033100==-3]<-0
table(A$E8012210, useNA="always")
table(A$A0210, useNA="always")
A$A0211<-NA
A$A0211[A$E8012211==0]<-0
A$A0211[A$E8012211==99]<-0
A$A0211[A$E8012211>=1 & A$E8012211<99]<-A$E8012211[A$E8012211>=1 &
A$E8012211<99]
A$A0211[is.na(A$A0211) & A$E8033000==-4]<-0
A$A0211[is.na(A$A0211) & A$E8033000==-3]<-0
A$A0211[is.na(A$A0211) & A$E8033000 >= 199300]<-0
A$A0211[is.na(A$A0211) & A$E8033100==0]<-0
A$A0211[is.na(A$A0211) & A$E8033100==-3]<-0
table(A$E8012211, useNA="always")
table(A$A0211, useNA="always")
A$A0212<-NA
A$A0212[A$E8012212==0]<-0
A$A0212[A$E8012212==99]<-0
A$A0212[A$E8012212>=1 & A$E8012212<99]<-A$E8012212[A$E8012212>=1 &
A$E8012212<99]
A$A0212[is.na(A$A0212) & A$E8033000==-4]<-0
A$A0212[is.na(A$A0212) & A$E8033000==-3]<-0
A$A0212[is.na(A$A0212) & A$E8033000 >= 199300]<-0
A$A0212[is.na(A$A0212) & A$E8033100==0]<-0
A$A0212[is.na(A$A0212) & A$E8033100==-3]<-0
table(A$E8012212, useNA="always")
table(A$A0212, useNA="always")




A$A02<-
rowSums(data.frame(A$A0201,A$A0202,A$A0203,A$A0204,A$A0205,A$A0206,A$A0207,A$
A0208,A$A0209,A$A0210,A$A0211,A$A0212), na.rm = T)
table(A$A02)



### Arrest (2003)

table(A$E8012301, useNA="always")
table(A$E8012302)
table(A$E8012303)
table(A$E8012304)
table(A$E8012305)
table(A$E8012306)
```

© 2023 Silver IA et al. *JAMA Network Open.*

```
table(A$E8012307)
table(A$E8012308)
table(A$E8012309)
table(A$E8012310)
table(A$E8012311)
table(A$E8012312)

A$A0301<-NA
A$A0301[A$E8012301==0]<-0
A$A0301[A$E8012301==99]<-0
A$A0301[A$E8012301>=1 & A$E8012301<99]<-A$E8012301[A$E8012301>=1 &
A$E8012301<99]
A$A0301[is.na(A$A0301) & A$E8033000==-4]<-0
A$A0301[is.na(A$A0301) & A$E8033000==-3]<-0
A$A0301[is.na(A$A0301) & A$E8033000 >= 199300]<-0
A$A0301[is.na(A$A0301) & A$E8033100==0]<-0
A$A0301[is.na(A$A0301) & A$E8033100==-3]<-0
table(A$E8012301, useNA="always")
table(A$A0301, useNA="always")
A$A0302<-NA
A$A0302[A$E8012302==0]<-0
A$A0302[A$E8012302==99]<-0
A$A0302[A$E8012302>=1 & A$E8012302<99]<-A$E8012302[A$E8012302>=1 &
A$E8012302<99]
A$A0302[is.na(A$A0302) & A$E8033000==-4]<-0
A$A0302[is.na(A$A0302) & A$E8033000==-3]<-0
A$A0302[is.na(A$A0302) & A$E8033000 >= 199300]<-0
A$A0302[is.na(A$A0302) & A$E8033100==0]<-0
A$A0302[is.na(A$A0302) & A$E8033100==-3]<-0
table(A$E8012302, useNA="always")
table(A$A0302, useNA="always")
A$A0303<-NA
A$A0303[A$E8012303==0]<-0
A$A0303[A$E8012303==99]<-0
A$A0303[A$E8012303>=1 & A$E8012303<99]<-A$E8012303[A$E8012303>=1 &
A$E8012303<99]
A$A0303[is.na(A$A0303) & A$E8033000==-4]<-0
A$A0303[is.na(A$A0303) & A$E8033000==-3]<-0
A$A0303[is.na(A$A0303) & A$E8033000 >= 199300]<-0
A$A0303[is.na(A$A0303) & A$E8033100==0]<-0
A$A0303[is.na(A$A0303) & A$E8033100==-3]<-0
table(A$E8012303, useNA="always")
table(A$A0303, useNA="always")
A$A0304<-NA
A$A0304[A$E8012304==0]<-0
A$A0304[A$E8012304==99]<-0
A$A0304[A$E8012304>=1 & A$E8012304<99]<-A$E8012304[A$E8012304>=1 &
A$E8012304<99]
A$A0304[is.na(A$A0304) & A$E8033000==-4]<-0
A$A0304[is.na(A$A0304) & A$E8033000==-3]<-0
A$A0304[is.na(A$A0304) & A$E8033000 >= 199300]<-0
A$A0304[is.na(A$A0304) & A$E8033100==0]<-0
A$A0304[is.na(A$A0304) & A$E8033100==-3]<-0
table(A$E8012304, useNA="always")
table(A$A0304, useNA="always")
A$A0305<-NA
```

```
A$A0305[A$E8012305==0]<-0
A$A0305[A$E8012305==99]<-0
A$A0305[A$E8012305>=1 & A$E8012305<99]<-A$E8012305[A$E8012305>=1 &
A$E8012305<99]
A$A0305[is.na(A$A0305) & A$E8033000==-4]<-0
A$A0305[is.na(A$A0305) & A$E8033000==-3]<-0
A$A0305[is.na(A$A0305) & A$E8033000 >= 199300]<-0
A$A0305[is.na(A$A0305) & A$E8033100==0]<-0
A$A0305[is.na(A$A0305) & A$E8033100==-3]<-0
table(A$E8012305, useNA="always")
table(A$A0305, useNA="always")
A$A0306<-NA
A$A0306[A$E8012306==0]<-0
A$A0306[A$E8012306==99]<-0
A$A0306[A$E8012306>=1 & A$E8012306<99]<-A$E8012306[A$E8012306>=1 &
A$E8012306<99]
A$A0306[is.na(A$A0306) & A$E8033000==-4]<-0
A$A0306[is.na(A$A0306) & A$E8033000==-3]<-0
A$A0306[is.na(A$A0306) & A$E8033000 >= 199300]<-0
A$A0306[is.na(A$A0306) & A$E8033100==0]<-0
A$A0306[is.na(A$A0306) & A$E8033100==-3]<-0
table(A$E8012306, useNA="always")
table(A$A0306, useNA="always")
A$A0307<-NA
A$A0307[A$E8012307==0]<-0
A$A0307[A$E8012307==99]<-0
A$A0307[A$E8012307>=1 & A$E8012307<99]<-A$E8012307[A$E8012307>=1 &
A$E8012307<99]
A$A0307[is.na(A$A0307) & A$E8033000==-4]<-0
A$A0307[is.na(A$A0307) & A$E8033000==-3]<-0
A$A0307[is.na(A$A0307) & A$E8033000 >= 199300]<-0
A$A0307[is.na(A$A0307) & A$E8033100==0]<-0
A$A0307[is.na(A$A0307) & A$E8033100==-3]<-0
table(A$E8012307, useNA="always")
table(A$A0307, useNA="always")
A$A0308<-NA
A$A0308[A$E8012308==0]<-0
A$A0308[A$E8012308==99]<-0
A$A0308[A$E8012308>=1 & A$E8012308<99]<-A$E8012308[A$E8012308>=1 &
A$E8012308<99]
A$A0308[is.na(A$A0308) & A$E8033000==-4]<-0
A$A0308[is.na(A$A0308) & A$E8033000==-3]<-0
A$A0308[is.na(A$A0308) & A$E8033000 >= 199300]<-0
A$A0308[is.na(A$A0308) & A$E8033100==0]<-0
A$A0308[is.na(A$A0308) & A$E8033100==-3]<-0
table(A$E8012308, useNA="always")
table(A$A0308, useNA="always")
A$A0309<-NA
A$A0309[A$E8012309==0]<-0
A$A0309[A$E8012309==99]<-0
A$A0309[A$E8012309>=1 & A$E8012309<99]<-A$E8012309[A$E8012309>=1 &
A$E8012309<99]
A$A0309[is.na(A$A0309) & A$E8033000==-4]<-0
A$A0309[is.na(A$A0309) & A$E8033000==-3]<-0
A$A0309[is.na(A$A0309) & A$E8033000 >= 199300]<-0
A$A0309[is.na(A$A0309) & A$E8033100==0]<-0
```

```
A$A0309[is.na(A$A0309) & A$E8033100==-3]<-0
table(A$E8012309, useNA="always")
table(A$A0309, useNA="always")
A$A0310<-NA
A$A0310[A$E8012310==0]<-0
A$A0310[A$E8012310==99]<-0
A$A0310[A$E8012310>=1 & A$E8012310<99]<-A$E8012310[A$E8012310>=1 &
A$E8012310<99]
A$A0310[is.na(A$A0310) & A$E8033000==-4]<-0
A$A0310[is.na(A$A0310) & A$E8033000==-3]<-0
A$A0310[is.na(A$A0310) & A$E8033000 >= 199300]<-0
A$A0310[is.na(A$A0310) & A$E8033100==0]<-0
A$A0310[is.na(A$A0310) & A$E8033100==-3]<-0
table(A$E8012310, useNA="always")
table(A$A0310, useNA="always")
A$A0311<-NA
A$A0311[A$E8012311==0]<-0
A$A0311[A$E8012311==99]<-0
A$A0311[A$E8012311>=1 & A$E8012311<99]<-A$E8012311[A$E8012311>=1 &
A$E8012311<99]
A$A0311[is.na(A$A0311) & A$E8033000==-4]<-0
A$A0311[is.na(A$A0311) & A$E8033000==-3]<-0
A$A0311[is.na(A$A0311) & A$E8033000 >= 199300]<-0
A$A0311[is.na(A$A0311) & A$E8033100==0]<-0
A$A0311[is.na(A$A0311) & A$E8033100==-3]<-0
table(A$E8012311, useNA="always")
table(A$A0311, useNA="always")
A$A0312<-NA
A$A0312[A$E8012312==0]<-0
A$A0312[A$E8012312==99]<-0
A$A0312[A$E8012312>=1 & A$E8012312<99]<-A$E8012312[A$E8012312>=1 &
A$E8012312<99]
A$A0312[is.na(A$A0312) & A$E8033000==-4]<-0
A$A0312[is.na(A$A0312) & A$E8033000==-3]<-0
A$A0312[is.na(A$A0312) & A$E8033000 >= 199300]<-0
A$A0312[is.na(A$A0312) & A$E8033100==0]<-0
A$A0312[is.na(A$A0312) & A$E8033100==-3]<-0
table(A$E8012312, useNA="always")
table(A$A0312, useNA="always")



A$A03<-
rowSums(data.frame(A$A0301,A$A0302,A$A0303,A$A0304,A$A0305,A$A0306,A$A0307,A$
A0308,A$A0309,A$A0310,A$A0311,A$A0312), na.rm = T)
table(A$A03)



### Arrest (2004)

table(A$E8012401, useNA="always")
table(A$E8012402)
table(A$E8012403)
table(A$E8012404)
```

© 2023 Silver IA et al. *JAMA Network Open.*

```
table(A$E8012405)
table(A$E8012406)
table(A$E8012407)
table(A$E8012408)
table(A$E8012409)
table(A$E8012410)
table(A$E8012411)
table(A$E8012412)

A$A0401<-NA
A$A0401[A$E8012401==0]<-0
A$A0401[A$E8012401==99]<-0
A$A0401[A$E8012401>=1 & A$E8012401<99]<-A$E8012401[A$E8012401>=1 &
A$E8012401<99]
A$A0401[is.na(A$A0401) & A$E8033000==-4]<-0
A$A0401[is.na(A$A0401) & A$E8033000==-3]<-0
A$A0401[is.na(A$A0401) & A$E8033000 >= 199300]<-0
A$A0401[is.na(A$A0401) & A$E8033100==0]<-0
A$A0401[is.na(A$A0401) & A$E8033100==-3]<-0
table(A$E8012401, useNA="always")
table(A$A0401, useNA="always")
A$A0402<-NA
A$A0402[A$E8012402==0]<-0
A$A0402[A$E8012402==99]<-0
A$A0402[A$E8012402>=1 & A$E8012402<99]<-A$E8012402[A$E8012402>=1 &
A$E8012402<99]
A$A0402[is.na(A$A0402) & A$E8033000==-4]<-0
A$A0402[is.na(A$A0402) & A$E8033000==-3]<-0
A$A0402[is.na(A$A0402) & A$E8033000 >= 199300]<-0
A$A0402[is.na(A$A0402) & A$E8033100==0]<-0
A$A0402[is.na(A$A0402) & A$E8033100==-3]<-0
table(A$E8012402, useNA="always")
table(A$A0402, useNA="always")
A$A0403<-NA
A$A0403[A$E8012403==0]<-0
A$A0403[A$E8012403==99]<-0
A$A0403[A$E8012403>=1 & A$E8012403<99]<-A$E8012403[A$E8012403>=1 &
A$E8012403<99]
A$A0403[is.na(A$A0403) & A$E8033000==-4]<-0
A$A0403[is.na(A$A0403) & A$E8033000==-3]<-0
A$A0403[is.na(A$A0403) & A$E8033000 >= 199300]<-0
A$A0403[is.na(A$A0403) & A$E8033100==0]<-0
A$A0403[is.na(A$A0403) & A$E8033100==-3]<-0
table(A$E8012403, useNA="always")
table(A$A0403, useNA="always")
A$A0404<-NA
A$A0404[A$E8012404==0]<-0
A$A0404[A$E8012404==99]<-0
A$A0404[A$E8012404>=1 & A$E8012404<99]<-A$E8012404[A$E8012404>=1 &
A$E8012404<99]
A$A0404[is.na(A$A0404) & A$E8033000==-4]<-0
A$A0404[is.na(A$A0404) & A$E8033000==-3]<-0
A$A0404[is.na(A$A0404) & A$E8033000 >= 199300]<-0
A$A0404[is.na(A$A0404) & A$E8033100==0]<-0
A$A0404[is.na(A$A0404) & A$E8033100==-3]<-0
table(A$E8012404, useNA="always")
```

© 2023 Silver IA et al. *JAMA Network Open.*

```
table(A$A0404, useNA="always")
A$A0405<-NA
A$A0405[A$E8012405==0]<-0
A$A0405[A$E8012405==99]<-0
A$A0405[A$E8012405>=1 & A$E8012405<99]<-A$E8012405[A$E8012405>=1 &
A$E8012405<99]
A$A0405[is.na(A$A0405) & A$E8033000==-4]<-0
A$A0405[is.na(A$A0405) & A$E8033000==-3]<-0
A$A0405[is.na(A$A0405) & A$E8033000 >= 199300]<-0
A$A0405[is.na(A$A0405) & A$E8033100==0]<-0
A$A0405[is.na(A$A0405) & A$E8033100==-3]<-0
table(A$E8012405, useNA="always")
table(A$A0405, useNA="always")
A$A0406<-NA
A$A0406[A$E8012406==0]<-0
A$A0406[A$E8012406==99]<-0
A$A0406[A$E8012406>=1 & A$E8012406<99]<-A$E8012406[A$E8012406>=1 &
A$E8012406<99]
A$A0406[is.na(A$A0406) & A$E8033000==-4]<-0
A$A0406[is.na(A$A0406) & A$E8033000==-3]<-0
A$A0406[is.na(A$A0406) & A$E8033000 >= 199300]<-0
A$A0406[is.na(A$A0406) & A$E8033100==0]<-0
A$A0406[is.na(A$A0406) & A$E8033100==-3]<-0
table(A$E8012406, useNA="always")
table(A$A0406, useNA="always")
A$A0407<-NA
A$A0407[A$E8012407==0]<-0
A$A0407[A$E8012407==99]<-0
A$A0407[A$E8012407>=1 & A$E8012407<99]<-A$E8012407[A$E8012407>=1 &
A$E8012407<99]
A$A0407[is.na(A$A0407) & A$E8033000==-4]<-0
A$A0407[is.na(A$A0407) & A$E8033000==-3]<-0
A$A0407[is.na(A$A0407) & A$E8033000 >= 199300]<-0
A$A0407[is.na(A$A0407) & A$E8033100==0]<-0
A$A0407[is.na(A$A0407) & A$E8033100==-3]<-0
table(A$E8012407, useNA="always")
table(A$A0407, useNA="always")
A$A0408<-NA
A$A0408[A$E8012408==0]<-0
A$A0408[A$E8012408==99]<-0
A$A0408[A$E8012408>=1 & A$E8012408<99]<-A$E8012408[A$E8012408>=1 &
A$E8012408<99]
A$A0408[is.na(A$A0408) & A$E8033000==-4]<-0
A$A0408[is.na(A$A0408) & A$E8033000==-3]<-0
A$A0408[is.na(A$A0408) & A$E8033000 >= 199300]<-0
A$A0408[is.na(A$A0408) & A$E8033100==0]<-0
A$A0408[is.na(A$A0408) & A$E8033100==-3]<-0
table(A$E8012408, useNA="always")
table(A$A0408, useNA="always")
A$A0409<-NA
A$A0409[A$E8012409==0]<-0
A$A0409[A$E8012409==99]<-0
A$A0409[A$E8012409>=1 & A$E8012409<99]<-A$E8012409[A$E8012409>=1 &
A$E8012409<99]
A$A0409[is.na(A$A0409) & A$E8033000==-4]<-0
A$A0409[is.na(A$A0409) & A$E8033000==-3]<-0
```

```
A$A0409[is.na(A$A0409) & A$E8033000 >= 199300]<-0
A$A0409[is.na(A$A0409) & A$E8033100==0]<-0
A$A0409[is.na(A$A0409) & A$E8033100==-3]<-0
table(A$E8012409, useNA="always")
table(A$A0409, useNA="always")
A$A0410<-NA
A$A0410[A$E8012410==0]<-0
A$A0410[A$E8012410==99]<-0
A$A0410[A$E8012410>=1 & A$E8012410<99]<-A$E8012410[A$E8012410>=1 &
A$E8012410<99]
A$A0410[is.na(A$A0410) & A$E8033000==-4]<-0
A$A0410[is.na(A$A0410) & A$E8033000==-3]<-0
A$A0410[is.na(A$A0410) & A$E8033000 >= 199300]<-0
A$A0410[is.na(A$A0410) & A$E8033100==0]<-0
A$A0410[is.na(A$A0410) & A$E8033100==-3]<-0
table(A$E8012410, useNA="always")
table(A$A0410, useNA="always")
A$A0411<-NA
A$A0411[A$E8012411==0]<-0
A$A0411[A$E8012411==99]<-0
A$A0411[A$E8012411>=1 & A$E8012411<99]<-A$E8012411[A$E8012411>=1 &
A$E8012411<99]
A$A0411[is.na(A$A0411) & A$E8033000==-4]<-0
A$A0411[is.na(A$A0411) & A$E8033000==-3]<-0
A$A0411[is.na(A$A0411) & A$E8033000 >= 199300]<-0
A$A0411[is.na(A$A0411) & A$E8033100==0]<-0
A$A0411[is.na(A$A0411) & A$E8033100==-3]<-0
table(A$E8012411, useNA="always")
table(A$A0411, useNA="always")
A$A0412<-NA
A$A0412[A$E8012412==0]<-0
A$A0412[A$E8012412==99]<-0
A$A0412[A$E8012412>=1 & A$E8012412<99]<-A$E8012412[A$E8012412>=1 &
A$E8012412<99]
A$A0412[is.na(A$A0412) & A$E8033000==-4]<-0
A$A0412[is.na(A$A0412) & A$E8033000==-3]<-0
A$A0412[is.na(A$A0412) & A$E8033000 >= 199300]<-0
A$A0412[is.na(A$A0412) & A$E8033100==0]<-0
A$A0412[is.na(A$A0412) & A$E8033100==-3]<-0
table(A$E8012412, useNA="always")
table(A$A0412, useNA="always")




A$A04<-
rowSums(data.frame(A$A0401,A$A0402,A$A0403,A$A0404,A$A0405,A$A0406,A$A0407,A$
A0408,A$A0409,A$A0410,A$A0411,A$A0412), na.rm = T)
table(A$A04)



### Arrest (2005)

table(A$E8012501, useNA="always")
table(A$E8012502)
```

© 2023 Silver IA et al. *JAMA Network Open.*

```
table(A$E8012503)
table(A$E8012504)
table(A$E8012505)
table(A$E8012506)
table(A$E8012507)
table(A$E8012508)
table(A$E8012509)
table(A$E8012510)
table(A$E8012511)
table(A$E8012512)

A$A0501<-NA
A$A0501[A$E8012501==0]<-0
A$A0501[A$E8012501==99]<-0
A$A0501[A$E8012501>=1 & A$E8012501<99]<-A$E8012501[A$E8012501>=1 &
A$E8012501<99]
A$A0501[is.na(A$A0501) & A$E8033000==-4]<-0
A$A0501[is.na(A$A0501) & A$E8033000==-3]<-0
A$A0501[is.na(A$A0501) & A$E8033000 >= 199300]<-0
A$A0501[is.na(A$A0501) & A$E8033100==0]<-0
A$A0501[is.na(A$A0501) & A$E8033100==-3]<-0
table(A$E8012501, useNA="always")
table(A$A0501, useNA="always")
A$A0502<-NA
A$A0502[A$E8012502==0]<-0
A$A0502[A$E8012502==99]<-0
A$A0502[A$E8012502>=1 & A$E8012502<99]<-A$E8012502[A$E8012502>=1 &
A$E8012502<99]
A$A0502[is.na(A$A0502) & A$E8033000==-4]<-0
A$A0502[is.na(A$A0502) & A$E8033000==-3]<-0
A$A0502[is.na(A$A0502) & A$E8033000 >= 199300]<-0
A$A0502[is.na(A$A0502) & A$E8033100==0]<-0
A$A0502[is.na(A$A0502) & A$E8033100==-3]<-0
table(A$E8012502, useNA="always")
table(A$A0502, useNA="always")
A$A0503<-NA
A$A0503[A$E8012503==0]<-0
A$A0503[A$E8012503==99]<-0
A$A0503[A$E8012503>=1 & A$E8012503<99]<-A$E8012503[A$E8012503>=1 &
A$E8012503<99]
A$A0503[is.na(A$A0503) & A$E8033000==-4]<-0
A$A0503[is.na(A$A0503) & A$E8033000==-3]<-0
A$A0503[is.na(A$A0503) & A$E8033000 >= 199300]<-0
A$A0503[is.na(A$A0503) & A$E8033100==0]<-0
A$A0503[is.na(A$A0503) & A$E8033100==-3]<-0
table(A$E8012503, useNA="always")
table(A$A0503, useNA="always")
A$A0504<-NA
A$A0504[A$E8012504==0]<-0
A$A0504[A$E8012504==99]<-0
A$A0504[A$E8012504>=1 & A$E8012504<99]<-A$E8012504[A$E8012504>=1 &
A$E8012504<99]
A$A0504[is.na(A$A0504) & A$E8033000==-4]<-0
A$A0504[is.na(A$A0504) & A$E8033000==-3]<-0
A$A0504[is.na(A$A0504) & A$E8033000 >= 199300]<-0
A$A0504[is.na(A$A0504) & A$E8033100==0]<-0
```

```
A$A0504[is.na(A$A0504) & A$E8033100==-3]<-0
table(A$E8012504, useNA="always")
table(A$A0504, useNA="always")
A$A0505<-NA
A$A0505[A$E8012505==0]<-0
A$A0505[A$E8012505==99]<-0
A$A0505[A$E8012505>=1 & A$E8012505<99]<-A$E8012505[A$E8012505>=1 &
A$E8012505<99]
A$A0505[is.na(A$A0505) & A$E8033000==-4]<-0
A$A0505[is.na(A$A0505) & A$E8033000==-3]<-0
A$A0505[is.na(A$A0505) & A$E8033000 >= 199300]<-0
A$A0505[is.na(A$A0505) & A$E8033100==0]<-0
A$A0505[is.na(A$A0505) & A$E8033100==-3]<-0
table(A$E8012505, useNA="always")
table(A$A0505, useNA="always")
A$A0506<-NA
A$A0506[A$E8012506==0]<-0
A$A0506[A$E8012506==99]<-0
A$A0506[A$E8012506>=1 & A$E8012506<99]<-A$E8012506[A$E8012506>=1 &
A$E8012506<99]
A$A0506[is.na(A$A0506) & A$E8033000==-4]<-0
A$A0506[is.na(A$A0506) & A$E8033000==-3]<-0
A$A0506[is.na(A$A0506) & A$E8033000 >= 199300]<-0
A$A0506[is.na(A$A0506) & A$E8033100==0]<-0
A$A0506[is.na(A$A0506) & A$E8033100==-3]<-0
table(A$E8012506, useNA="always")
table(A$A0506, useNA="always")
A$A0507<-NA
A$A0507[A$E8012507==0]<-0
A$A0507[A$E8012507==99]<-0
A$A0507[A$E8012507>=1 & A$E8012507<99]<-A$E8012507[A$E8012507>=1 &
A$E8012507<99]
A$A0507[is.na(A$A0507) & A$E8033000==-4]<-0
A$A0507[is.na(A$A0507) & A$E8033000==-3]<-0
A$A0507[is.na(A$A0507) & A$E8033000 >= 199300]<-0
A$A0507[is.na(A$A0507) & A$E8033100==0]<-0
A$A0507[is.na(A$A0507) & A$E8033100==-3]<-0
table(A$E8012507, useNA="always")
table(A$A0507, useNA="always")
A$A0508<-NA
A$A0508[A$E8012508==0]<-0
A$A0508[A$E8012508==99]<-0
A$A0508[A$E8012508>=1 & A$E8012508<99]<-A$E8012508[A$E8012508>=1 &
A$E8012508<99]
A$A0508[is.na(A$A0508) & A$E8033000==-4]<-0
A$A0508[is.na(A$A0508) & A$E8033000==-3]<-0
A$A0508[is.na(A$A0508) & A$E8033000 >= 199300]<-0
A$A0508[is.na(A$A0508) & A$E8033100==0]<-0
A$A0508[is.na(A$A0508) & A$E8033100==-3]<-0
table(A$E8012508, useNA="always")
table(A$A0508, useNA="always")
A$A0509<-NA
A$A0509[A$E8012509==0]<-0
A$A0509[A$E8012509==99]<-0
A$A0509[A$E8012509>=1 & A$E8012509<99]<-A$E8012509[A$E8012509>=1 &
A$E8012509<99]
```

© 2023 Silver IA et al. *JAMA Network Open.*

```
A$A0509[is.na(A$A0509) & A$E8033000==-4]<-0
A$A0509[is.na(A$A0509) & A$E8033000==-3]<-0
A$A0509[is.na(A$A0509) & A$E8033000 >= 199300]<-0
A$A0509[is.na(A$A0509) & A$E8033100==0]<-0
A$A0509[is.na(A$A0509) & A$E8033100==-3]<-0
table(A$E8012509, useNA="always")
table(A$A0509, useNA="always")
A$A0510<-NA
A$A0510[A$E8012510==0]<-0
A$A0510[A$E8012510==99]<-0
A$A0510[A$E8012510>=1 & A$E8012510<99]<-A$E8012510[A$E8012510>=1 &
A$E8012510<99]
A$A0510[is.na(A$A0510) & A$E8033000==-4]<-0
A$A0510[is.na(A$A0510) & A$E8033000==-3]<-0
A$A0510[is.na(A$A0510) & A$E8033000 >= 199300]<-0
A$A0510[is.na(A$A0510) & A$E8033100==0]<-0
A$A0510[is.na(A$A0510) & A$E8033100==-3]<-0
table(A$E8012510, useNA="always")
table(A$A0510, useNA="always")
A$A0511<-NA
A$A0511[A$E8012511==0]<-0
A$A0511[A$E8012511==99]<-0
A$A0511[A$E8012511>=1 & A$E8012511<99]<-A$E8012511[A$E8012511>=1 &
A$E8012511<99]
A$A0511[is.na(A$A0511) & A$E8033000==-4]<-0
A$A0511[is.na(A$A0511) & A$E8033000==-3]<-0
A$A0511[is.na(A$A0511) & A$E8033000 >= 199300]<-0
A$A0511[is.na(A$A0511) & A$E8033100==0]<-0
A$A0511[is.na(A$A0511) & A$E8033100==-3]<-0
table(A$E8012511, useNA="always")
table(A$A0511, useNA="always")
A$A0512<-NA
A$A0512[A$E8012512==0]<-0
A$A0512[A$E8012512==99]<-0
A$A0512[A$E8012512>=1 & A$E8012512<99]<-A$E8012512[A$E8012512>=1 &
A$E8012512<99]
A$A0512[is.na(A$A0512) & A$E8033000==-4]<-0
A$A0512[is.na(A$A0512) & A$E8033000==-3]<-0
A$A0512[is.na(A$A0512) & A$E8033000 >= 199300]<-0
A$A0512[is.na(A$A0512) & A$E8033100==0]<-0
A$A0512[is.na(A$A0512) & A$E8033100==-3]<-0
table(A$E8012512, useNA="always")
table(A$A0512, useNA="always")




A$A05<-
rowSums(data.frame(A$A0501,A$A0502,A$A0503,A$A0504,A$A0505,A$A0506,A$A0507,A$
A0508,A$A0509,A$A0510,A$A0511,A$A0512), na.rm = T)
table(A$A05)



### Arrest (2006)
```

© 2023 Silver IA et al. *JAMA Network Open.*

```
table(A$E8012601, useNA="always")
table(A$E8012602)
table(A$E8012603)
table(A$E8012604)
table(A$E8012605)
table(A$E8012606)
table(A$E8012607)
table(A$E8012608)
table(A$E8012609)
table(A$E8012610)
table(A$E8012611)
table(A$E8012612)

A$A0601<-NA
A$A0601[A$E8012601==0]<-0
A$A0601[A$E8012601==99]<-0
A$A0601[A$E8012601>=1 & A$E8012601<99]<-A$E8012601[A$E8012601>=1 &
A$E8012601<99]
A$A0601[is.na(A$A0601) & A$E8033000==-4]<-0
A$A0601[is.na(A$A0601) & A$E8033000==-3]<-0
A$A0601[is.na(A$A0601) & A$E8033000 >= 199300]<-0
A$A0601[is.na(A$A0601) & A$E8033100==0]<-0
A$A0601[is.na(A$A0601) & A$E8033100==-3]<-0
table(A$E8012601, useNA="always")
table(A$A0601, useNA="always")
A$A0602<-NA
A$A0602[A$E8012602==0]<-0
A$A0602[A$E8012602==99]<-0
A$A0602[A$E8012602>=1 & A$E8012602<99]<-A$E8012602[A$E8012602>=1 &
A$E8012602<99]
A$A0602[is.na(A$A0602) & A$E8033000==-4]<-0
A$A0602[is.na(A$A0602) & A$E8033000==-3]<-0
A$A0602[is.na(A$A0602) & A$E8033000 >= 199300]<-0
A$A0602[is.na(A$A0602) & A$E8033100==0]<-0
A$A0602[is.na(A$A0602) & A$E8033100==-3]<-0
table(A$E8012602, useNA="always")
table(A$A0602, useNA="always")
A$A0603<-NA
A$A0603[A$E8012603==0]<-0
A$A0603[A$E8012603==99]<-0
A$A0603[A$E8012603>=1 & A$E8012603<99]<-A$E8012603[A$E8012603>=1 &
A$E8012603<99]
A$A0603[is.na(A$A0603) & A$E8033000==-4]<-0
A$A0603[is.na(A$A0603) & A$E8033000==-3]<-0
A$A0603[is.na(A$A0603) & A$E8033000 >= 199300]<-0
A$A0603[is.na(A$A0603) & A$E8033100==0]<-0
A$A0603[is.na(A$A0603) & A$E8033100==-3]<-0
table(A$E8012603, useNA="always")
table(A$A0603, useNA="always")
A$A0604<-NA
A$A0604[A$E8012604==0]<-0
A$A0604[A$E8012604==99]<-0
A$A0604[A$E8012604>=1 & A$E8012604<99]<-A$E8012604[A$E8012604>=1 &
A$E8012604<99]
A$A0604[is.na(A$A0604) & A$E8033000==-4]<-0
A$A0604[is.na(A$A0604) & A$E8033000==-3]<-0
```

```
A$A0604[is.na(A$A0604) & A$E8033000 >= 199300]<-0
A$A0604[is.na(A$A0604) & A$E8033100==0]<-0
A$A0604[is.na(A$A0604) & A$E8033100==-3]<-0
table(A$E8012604, useNA="always")
table(A$A0604, useNA="always")
A$A0605<-NA
A$A0605[A$E8012605==0]<-0
A$A0605[A$E8012605==99]<-0
A$A0605[A$E8012605>=1 & A$E8012605<99]<-A$E8012605[A$E8012605>=1 &
A$E8012605<99]
A$A0605[is.na(A$A0605) & A$E8033000==-4]<-0
A$A0605[is.na(A$A0605) & A$E8033000==-3]<-0
A$A0605[is.na(A$A0605) & A$E8033000 >= 199300]<-0
A$A0605[is.na(A$A0605) & A$E8033100==0]<-0
A$A0605[is.na(A$A0605) & A$E8033100==-3]<-0
table(A$E8012605, useNA="always")
table(A$A0605, useNA="always")
A$A0606<-NA
A$A0606[A$E8012606==0]<-0
A$A0606[A$E8012606==99]<-0
A$A0606[A$E8012606>=1 & A$E8012606<99]<-A$E8012606[A$E8012606>=1 &
A$E8012606<99]
A$A0606[is.na(A$A0606) & A$E8033000==-4]<-0
A$A0606[is.na(A$A0606) & A$E8033000==-3]<-0
A$A0606[is.na(A$A0606) & A$E8033000 >= 199300]<-0
A$A0606[is.na(A$A0606) & A$E8033100==0]<-0
A$A0606[is.na(A$A0606) & A$E8033100==-3]<-0
table(A$E8012606, useNA="always")
table(A$A0606, useNA="always")
A$A0607<-NA
A$A0607[A$E8012607==0]<-0
A$A0607[A$E8012607==99]<-0
A$A0607[A$E8012607>=1 & A$E8012607<99]<-A$E8012607[A$E8012607>=1 &
A$E8012607<99]
A$A0607[is.na(A$A0607) & A$E8033000==-4]<-0
A$A0607[is.na(A$A0607) & A$E8033000==-3]<-0
A$A0607[is.na(A$A0607) & A$E8033000 >= 199300]<-0
A$A0607[is.na(A$A0607) & A$E8033100==0]<-0
A$A0607[is.na(A$A0607) & A$E8033100==-3]<-0
table(A$E8012607, useNA="always")
table(A$A0607, useNA="always")
A$A0608<-NA
A$A0608[A$E8012608==0]<-0
A$A0608[A$E8012608==99]<-0
A$A0608[A$E8012608>=1 & A$E8012608<99]<-A$E8012608[A$E8012608>=1 &
A$E8012608<99]
A$A0608[is.na(A$A0608) & A$E8033000==-4]<-0
A$A0608[is.na(A$A0608) & A$E8033000==-3]<-0
A$A0608[is.na(A$A0608) & A$E8033000 >= 199300]<-0
A$A0608[is.na(A$A0608) & A$E8033100==0]<-0
A$A0608[is.na(A$A0608) & A$E8033100==-3]<-0
table(A$E8012608, useNA="always")
table(A$A0608, useNA="always")
A$A0609<-NA
A$A0609[A$E8012609==0]<-0
A$A0609[A$E8012609==99]<-0
```

© 2023 Silver IA et al. *JAMA Network Open.*

```
A$A0609[A$E8012609>=1 & A$E8012609<99]<-A$E8012609[A$E8012609>=1 &
A$E8012609<99]
A$A0609[is.na(A$A0609) & A$E8033000==-4]<-0
A$A0609[is.na(A$A0609) & A$E8033000==-3]<-0
A$A0609[is.na(A$A0609) & A$E8033000 >= 199300]<-0
A$A0609[is.na(A$A0609) & A$E8033100==0]<-0
A$A0609[is.na(A$A0609) & A$E8033100==-3]<-0
table(A$E8012609, useNA="always")
table(A$A0609, useNA="always")
A$A0610<-NA
A$A0610[A$E8012610==0]<-0
A$A0610[A$E8012610==99]<-0
A$A0610[A$E8012610>=1 & A$E8012610<99]<-A$E8012610[A$E8012610>=1 &
A$E8012610<99]
A$A0610[is.na(A$A0610) & A$E8033000==-4]<-0
A$A0610[is.na(A$A0610) & A$E8033000==-3]<-0
A$A0610[is.na(A$A0610) & A$E8033000 >= 199300]<-0
A$A0610[is.na(A$A0610) & A$E8033100==0]<-0
A$A0610[is.na(A$A0610) & A$E8033100==-3]<-0
table(A$E8012610, useNA="always")
table(A$A0610, useNA="always")
A$A0611<-NA
A$A0611[A$E8012611==0]<-0
A$A0611[A$E8012611==99]<-0
A$A0611[A$E8012611>=1 & A$E8012611<99]<-A$E8012611[A$E8012611>=1 &
A$E8012611<99]
A$A0611[is.na(A$A0611) & A$E8033000==-4]<-0
A$A0611[is.na(A$A0611) & A$E8033000==-3]<-0
A$A0611[is.na(A$A0611) & A$E8033000 >= 199300]<-0
A$A0611[is.na(A$A0611) & A$E8033100==0]<-0
A$A0611[is.na(A$A0611) & A$E8033100==-3]<-0
table(A$E8012611, useNA="always")
table(A$A0611, useNA="always")
A$A0612<-NA
A$A0612[A$E8012612==0]<-0
A$A0612[A$E8012612==99]<-0
A$A0612[A$E8012612>=1 & A$E8012612<99]<-A$E8012612[A$E8012612>=1 &
A$E8012612<99]
A$A0612[is.na(A$A0612) & A$E8033000==-4]<-0
A$A0612[is.na(A$A0612) & A$E8033000==-3]<-0
A$A0612[is.na(A$A0612) & A$E8033000 >= 199300]<-0
A$A0612[is.na(A$A0612) & A$E8033100==0]<-0
A$A0612[is.na(A$A0612) & A$E8033100==-3]<-0
table(A$E8012612, useNA="always")
table(A$A0612, useNA="always")



A$A06<-
rowSums(data.frame(A$A0601,A$A0602,A$A0603,A$A0604,A$A0605,A$A0606,A$A0607,A$
A0608,A$A0609,A$A0610,A$A0611,A$A0612), na.rm = T)
table(A$A06)
```

© 2023 Silver IA et al. *JAMA Network Open.*

```
### Arrest (2007)

table(A$E8012701, useNA="always")
table(A$E8012702)
table(A$E8012703)
table(A$E8012704)
table(A$E8012705)
table(A$E8012706)
table(A$E8012707)
table(A$E8012708)
table(A$E8012709)
table(A$E8012710)
table(A$E8012711)
table(A$E8012712)

A$A0701<-NA
A$A0701[A$E8012701==0]<-0
A$A0701[A$E8012701==99]<-0
A$A0701[A$E8012701>=1 & A$E8012701<99]<-A$E8012701[A$E8012701>=1 &
A$E8012701<99]
A$A0701[is.na(A$A0701) & A$E8033000==-4]<-0
A$A0701[is.na(A$A0701) & A$E8033000==-3]<-0
A$A0701[is.na(A$A0701) & A$E8033000 >= 199300]<-0
A$A0701[is.na(A$A0701) & A$E8033100==0]<-0
A$A0701[is.na(A$A0701) & A$E8033100==-3]<-0
table(A$E8012701, useNA="always")
table(A$A0701, useNA="always")
A$A0702<-NA
A$A0702[A$E8012702==0]<-0
A$A0702[A$E8012702==99]<-0
A$A0702[A$E8012702>=1 & A$E8012702<99]<-A$E8012702[A$E8012702>=1 &
A$E8012702<99]
A$A0702[is.na(A$A0702) & A$E8033000==-4]<-0
A$A0702[is.na(A$A0702) & A$E8033000==-3]<-0
A$A0702[is.na(A$A0702) & A$E8033000 >= 199300]<-0
A$A0702[is.na(A$A0702) & A$E8033100==0]<-0
A$A0702[is.na(A$A0702) & A$E8033100==-3]<-0
table(A$E8012702, useNA="always")
table(A$A0702, useNA="always")
A$A0703<-NA
A$A0703[A$E8012703==0]<-0
A$A0703[A$E8012703==99]<-0
A$A0703[A$E8012703>=1 & A$E8012703<99]<-A$E8012703[A$E8012703>=1 &
A$E8012703<99]
A$A0703[is.na(A$A0703) & A$E8033000==-4]<-0
A$A0703[is.na(A$A0703) & A$E8033000==-3]<-0
A$A0703[is.na(A$A0703) & A$E8033000 >= 199300]<-0
A$A0703[is.na(A$A0703) & A$E8033100==0]<-0
A$A0703[is.na(A$A0703) & A$E8033100==-3]<-0
table(A$E8012703, useNA="always")
table(A$A0703, useNA="always")
A$A0704<-NA
A$A0704[A$E8012704==0]<-0
A$A0704[A$E8012704==99]<-0
A$A0704[A$E8012704>=1 & A$E8012704<99]<-A$E8012704[A$E8012704>=1 &
A$E8012704<99]
```

© 2023 Silver IA et al. *JAMA Network Open.*

```
A$A0704[is.na(A$A0704) & A$E8033000==-4]<-0
A$A0704[is.na(A$A0704) & A$E8033000==-3]<-0
A$A0704[is.na(A$A0704) & A$E8033000 >= 199300]<-0
A$A0704[is.na(A$A0704) & A$E8033100==0]<-0
A$A0704[is.na(A$A0704) & A$E8033100==-3]<-0
table(A$E8012704, useNA="always")
table(A$A0704, useNA="always")
A$A0705<-NA
A$A0705[A$E8012705==0]<-0
A$A0705[A$E8012705==99]<-0
A$A0705[A$E8012705>=1 & A$E8012705<99]<-A$E8012705[A$E8012705>=1 &
A$E8012705<99]
A$A0705[is.na(A$A0705) & A$E8033000==-4]<-0
A$A0705[is.na(A$A0705) & A$E8033000==-3]<-0
A$A0705[is.na(A$A0705) & A$E8033000 >= 199300]<-0
A$A0705[is.na(A$A0705) & A$E8033100==0]<-0
A$A0705[is.na(A$A0705) & A$E8033100==-3]<-0
table(A$E8012705, useNA="always")
table(A$A0705, useNA="always")
A$A0706<-NA
A$A0706[A$E8012706==0]<-0
A$A0706[A$E8012706==99]<-0
A$A0706[A$E8012706>=1 & A$E8012706<99]<-A$E8012706[A$E8012706>=1 &
A$E8012706<99]
A$A0706[is.na(A$A0706) & A$E8033000==-4]<-0
A$A0706[is.na(A$A0706) & A$E8033000==-3]<-0
A$A0706[is.na(A$A0706) & A$E8033000 >= 199300]<-0
A$A0706[is.na(A$A0706) & A$E8033100==0]<-0
A$A0706[is.na(A$A0706) & A$E8033100==-3]<-0
table(A$E8012706, useNA="always")
table(A$A0706, useNA="always")
A$A0707<-NA
A$A0707[A$E8012707==0]<-0
A$A0707[A$E8012707==99]<-0
A$A0707[A$E8012707>=1 & A$E8012707<99]<-A$E8012707[A$E8012707>=1 &
A$E8012707<99]
A$A0707[is.na(A$A0707) & A$E8033000==-4]<-0
A$A0707[is.na(A$A0707) & A$E8033000==-3]<-0
A$A0707[is.na(A$A0707) & A$E8033000 >= 199300]<-0
A$A0707[is.na(A$A0707) & A$E8033100==0]<-0
A$A0707[is.na(A$A0707) & A$E8033100==-3]<-0
table(A$E8012707, useNA="always")
table(A$A0707, useNA="always")
A$A0708<-NA
A$A0708[A$E8012708==0]<-0
A$A0708[A$E8012708==99]<-0
A$A0708[A$E8012708>=1 & A$E8012708<99]<-A$E8012708[A$E8012708>=1 &
A$E8012708<99]
A$A0708[is.na(A$A0708) & A$E8033000==-4]<-0
A$A0708[is.na(A$A0708) & A$E8033000==-3]<-0
A$A0708[is.na(A$A0708) & A$E8033000 >= 199300]<-0
A$A0708[is.na(A$A0708) & A$E8033100==0]<-0
A$A0708[is.na(A$A0708) & A$E8033100==-3]<-0
table(A$E8012708, useNA="always")
table(A$A0708, useNA="always")
A$A0709<-NA
```

© 2023 Silver IA et al. *JAMA Network Open.*

```
A$A0709[A$E8012709==0]<-0
A$A0709[A$E8012709==99]<-0
A$A0709[A$E8012709>=1 & A$E8012709<99]<-A$E8012709[A$E8012709>=1 &
A$E8012709<99]
A$A0709[is.na(A$A0709) & A$E8033000==-4]<-0
A$A0709[is.na(A$A0709) & A$E8033000==-3]<-0
A$A0709[is.na(A$A0709) & A$E8033000 >= 199300]<-0
A$A0709[is.na(A$A0709) & A$E8033100==0]<-0
A$A0709[is.na(A$A0709) & A$E8033100==-3]<-0
table(A$E8012709, useNA="always")
table(A$A0709, useNA="always")
A$A0710<-NA
A$A0710[A$E8012710==0]<-0
A$A0710[A$E8012710==99]<-0
A$A0710[A$E8012710>=1 & A$E8012710<99]<-A$E8012710[A$E8012710>=1 &
A$E8012710<99]
A$A0710[is.na(A$A0710) & A$E8033000==-4]<-0
A$A0710[is.na(A$A0710) & A$E8033000==-3]<-0
A$A0710[is.na(A$A0710) & A$E8033000 >= 199300]<-0
A$A0710[is.na(A$A0710) & A$E8033100==0]<-0
A$A0710[is.na(A$A0710) & A$E8033100==-3]<-0
table(A$E8012710, useNA="always")
table(A$A0710, useNA="always")
A$A0711<-NA
A$A0711[A$E8012711==0]<-0
A$A0711[A$E8012711==99]<-0
A$A0711[A$E8012711>=1 & A$E8012711<99]<-A$E8012711[A$E8012711>=1 &
A$E8012711<99]
A$A0711[is.na(A$A0711) & A$E8033000==-4]<-0
A$A0711[is.na(A$A0711) & A$E8033000==-3]<-0
A$A0711[is.na(A$A0711) & A$E8033000 >= 199300]<-0
A$A0711[is.na(A$A0711) & A$E8033100==0]<-0
A$A0711[is.na(A$A0711) & A$E8033100==-3]<-0
table(A$E8012711, useNA="always")
table(A$A0711, useNA="always")
A$A0712<-NA
A$A0712[A$E8012712==0]<-0
A$A0712[A$E8012712==99]<-0
A$A0712[A$E8012712>=1 & A$E8012712<99]<-A$E8012712[A$E8012712>=1 &
A$E8012712<99]
A$A0712[is.na(A$A0712) & A$E8033000==-4]<-0
A$A0712[is.na(A$A0712) & A$E8033000==-3]<-0
A$A0712[is.na(A$A0712) & A$E8033000 >= 199300]<-0
A$A0712[is.na(A$A0712) & A$E8033100==0]<-0
A$A0712[is.na(A$A0712) & A$E8033100==-3]<-0
table(A$E8012712, useNA="always")
table(A$A0712, useNA="always")



A$A07<-
rowSums(data.frame(A$A0701,A$A0702,A$A0703,A$A0704,A$A0705,A$A0706,A$A0707,A$
A0708,A$A0709,A$A0710,A$A0711,A$A0712), na.rm = T)
table(A$A07)
```

© 2023 Silver IA et al. *JAMA Network Open.*

```
### Arrest (2008)

table(A$E8012801, useNA="always")
table(A$E8012802)
table(A$E8012803)
table(A$E8012804)
table(A$E8012805)
table(A$E8012806)
table(A$E8012807)
table(A$E8012808)
table(A$E8012809)
table(A$E8012810)
table(A$E8012811)
table(A$E8012812)

A$A0801<-NA
A$A0801[A$E8012801==0]<-0
A$A0801[A$E8012801==99]<-0
A$A0801[A$E8012801>=1 & A$E8012801<99]<-A$E8012801[A$E8012801>=1 &
A$E8012801<99]
A$A0801[is.na(A$A0801) & A$E8033000==-4]<-0
A$A0801[is.na(A$A0801) & A$E8033000==-3]<-0
A$A0801[is.na(A$A0801) & A$E8033000 >= 199300]<-0
A$A0801[is.na(A$A0801) & A$E8033100==0]<-0
A$A0801[is.na(A$A0801) & A$E8033100==-3]<-0
table(A$E8012801, useNA="always")
table(A$A0801, useNA="always")
A$A0802<-NA
A$A0802[A$E8012802==0]<-0
A$A0802[A$E8012802==99]<-0
A$A0802[A$E8012802>=1 & A$E8012802<99]<-A$E8012802[A$E8012802>=1 &
A$E8012802<99]
A$A0802[is.na(A$A0802) & A$E8033000==-4]<-0
A$A0802[is.na(A$A0802) & A$E8033000==-3]<-0
A$A0802[is.na(A$A0802) & A$E8033000 >= 199300]<-0
A$A0802[is.na(A$A0802) & A$E8033100==0]<-0
A$A0802[is.na(A$A0802) & A$E8033100==-3]<-0
table(A$E8012802, useNA="always")
table(A$A0802, useNA="always")
A$A0803<-NA
A$A0803[A$E8012803==0]<-0
A$A0803[A$E8012803==99]<-0
A$A0803[A$E8012803>=1 & A$E8012803<99]<-A$E8012803[A$E8012803>=1 &
A$E8012803<99]
A$A0803[is.na(A$A0803) & A$E8033000==-4]<-0
A$A0803[is.na(A$A0803) & A$E8033000==-3]<-0
A$A0803[is.na(A$A0803) & A$E8033000 >= 199300]<-0
A$A0803[is.na(A$A0803) & A$E8033100==0]<-0
A$A0803[is.na(A$A0803) & A$E8033100==-3]<-0
table(A$E8012803, useNA="always")
table(A$A0803, useNA="always")
A$A0804<-NA
A$A0804[A$E8012804==0]<-0
A$A0804[A$E8012804==99]<-0
```

© 2023 Silver IA et al. *JAMA Network Open.*

```
A$A0804[A$E8012804>=1 & A$E8012804<99]<-A$E8012804[A$E8012804>=1 &
A$E8012804<99]
A$A0804[is.na(A$A0804) & A$E8033000==-4]<-0
A$A0804[is.na(A$A0804) & A$E8033000==-3]<-0
A$A0804[is.na(A$A0804) & A$E8033000 >= 199300]<-0
A$A0804[is.na(A$A0804) & A$E8033100==0]<-0
A$A0804[is.na(A$A0804) & A$E8033100==-3]<-0
table(A$E8012804, useNA="always")
table(A$A0804, useNA="always")
A$A0805<-NA
A$A0805[A$E8012805==0]<-0
A$A0805[A$E8012805==99]<-0
A$A0805[A$E8012805>=1 & A$E8012805<99]<-A$E8012805[A$E8012805>=1 &
A$E8012805<99]
A$A0805[is.na(A$A0805) & A$E8033000==-4]<-0
A$A0805[is.na(A$A0805) & A$E8033000==-3]<-0
A$A0805[is.na(A$A0805) & A$E8033000 >= 199300]<-0
A$A0805[is.na(A$A0805) & A$E8033100==0]<-0
A$A0805[is.na(A$A0805) & A$E8033100==-3]<-0
table(A$E8012805, useNA="always")
table(A$A0805, useNA="always")
A$A0806<-NA
A$A0806[A$E8012806==0]<-0
A$A0806[A$E8012806==99]<-0
A$A0806[A$E8012806>=1 & A$E8012806<99]<-A$E8012806[A$E8012806>=1 &
A$E8012806<99]
A$A0806[is.na(A$A0806) & A$E8033000==-4]<-0
A$A0806[is.na(A$A0806) & A$E8033000==-3]<-0
A$A0806[is.na(A$A0806) & A$E8033000 >= 199300]<-0
A$A0806[is.na(A$A0806) & A$E8033100==0]<-0
A$A0806[is.na(A$A0806) & A$E8033100==-3]<-0
table(A$E8012806, useNA="always")
table(A$A0806, useNA="always")
A$A0807<-NA
A$A0807[A$E8012807==0]<-0
A$A0807[A$E8012807==99]<-0
A$A0807[A$E8012807>=1 & A$E8012807<99]<-A$E8012807[A$E8012807>=1 &
A$E8012807<99]
A$A0807[is.na(A$A0807) & A$E8033000==-4]<-0
A$A0807[is.na(A$A0807) & A$E8033000==-3]<-0
A$A0807[is.na(A$A0807) & A$E8033000 >= 199300]<-0
A$A0807[is.na(A$A0807) & A$E8033100==0]<-0
A$A0807[is.na(A$A0807) & A$E8033100==-3]<-0
table(A$E8012807, useNA="always")
table(A$A0807, useNA="always")
A$A0808<-NA
A$A0808[A$E8012808==0]<-0
A$A0808[A$E8012808==99]<-0
A$A0808[A$E8012808>=1 & A$E8012808<99]<-A$E8012808[A$E8012808>=1 &
A$E8012808<99]
A$A0808[is.na(A$A0808) & A$E8033000==-4]<-0
A$A0808[is.na(A$A0808) & A$E8033000==-3]<-0
A$A0808[is.na(A$A0808) & A$E8033000 >= 199300]<-0
A$A0808[is.na(A$A0808) & A$E8033100==0]<-0
A$A0808[is.na(A$A0808) & A$E8033100==-3]<-0
table(A$E8012808, useNA="always")
```

© 2023 Silver IA et al. *JAMA Network Open.*

```
table(A$A0808, useNA="always")
A$A0809<-NA
A$A0809[A$E8012809==0]<-0
A$A0809[A$E8012809==99]<-0
A$A0809[A$E8012809>=1 & A$E8012809<99]<-A$E8012809[A$E8012809>=1 &
A$E8012809<99]
A$A0809[is.na(A$A0809) & A$E8033000==-4]<-0
A$A0809[is.na(A$A0809) & A$E8033000==-3]<-0
A$A0809[is.na(A$A0809) & A$E8033000 >= 199300]<-0
A$A0809[is.na(A$A0809) & A$E8033100==0]<-0
A$A0809[is.na(A$A0809) & A$E8033100==-3]<-0
table(A$E8012809, useNA="always")
table(A$A0809, useNA="always")
A$A0810<-NA
A$A0810[A$E8012810==0]<-0
A$A0810[A$E8012810==99]<-0
A$A0810[A$E8012810>=1 & A$E8012810<99]<-A$E8012810[A$E8012810>=1 &
A$E8012810<99]
A$A0810[is.na(A$A0810) & A$E8033000==-4]<-0
A$A0810[is.na(A$A0810) & A$E8033000==-3]<-0
A$A0810[is.na(A$A0810) & A$E8033000 >= 199300]<-0
A$A0810[is.na(A$A0810) & A$E8033100==0]<-0
A$A0810[is.na(A$A0810) & A$E8033100==-3]<-0
table(A$E8012810, useNA="always")
table(A$A0810, useNA="always")
A$A0811<-NA
A$A0811[A$E8012811==0]<-0
A$A0811[A$E8012811==99]<-0
A$A0811[A$E8012811>=1 & A$E8012811<99]<-A$E8012811[A$E8012811>=1 &
A$E8012811<99]
A$A0811[is.na(A$A0811) & A$E8033000==-4]<-0
A$A0811[is.na(A$A0811) & A$E8033000==-3]<-0
A$A0811[is.na(A$A0811) & A$E8033000 >= 199300]<-0
A$A0811[is.na(A$A0811) & A$E8033100==0]<-0
A$A0811[is.na(A$A0811) & A$E8033100==-3]<-0
table(A$E8012811, useNA="always")
table(A$A0811, useNA="always")
A$A0812<-NA
A$A0812[A$E8012812==0]<-0
A$A0812[A$E8012812==99]<-0
A$A0812[A$E8012812>=1 & A$E8012812<99]<-A$E8012812[A$E8012812>=1 &
A$E8012812<99]
A$A0812[is.na(A$A0812) & A$E8033000==-4]<-0
A$A0812[is.na(A$A0812) & A$E8033000==-3]<-0
A$A0812[is.na(A$A0812) & A$E8033000 >= 199300]<-0
A$A0812[is.na(A$A0812) & A$E8033100==0]<-0
A$A0812[is.na(A$A0812) & A$E8033100==-3]<-0
table(A$E8012812, useNA="always")
table(A$A0812, useNA="always")



A$A08<-
rowSums(data.frame(A$A0801,A$A0802,A$A0803,A$A0804,A$A0805,A$A0806,A$A0807,A$
A0808,A$A0809,A$A0810,A$A0811,A$A0812), na.rm = T)
```

© 2023 Silver IA et al. *JAMA Network Open.*

```
table(A$A08)



### Arrest (2009)

table(A$E8012901, useNA="always")
table(A$E8012902)
table(A$E8012903)
table(A$E8012904)
table(A$E8012905)
table(A$E8012906)
table(A$E8012907)
table(A$E8012908)
table(A$E8012909)
table(A$E8012910)
table(A$E8012911)
table(A$E8012912)

A$A0901<-NA
A$A0901[A$E8012901==0]<-0
A$A0901[A$E8012901==99]<-0
A$A0901[A$E8012901>=1 & A$E8012901<99]<-A$E8012901[A$E8012901>=1 &
A$E8012901<99]
A$A0901[is.na(A$A0901) & A$E8033000==-4]<-0
A$A0901[is.na(A$A0901) & A$E8033000==-3]<-0
A$A0901[is.na(A$A0901) & A$E8033000 >= 199300]<-0
A$A0901[is.na(A$A0901) & A$E8033100==0]<-0
A$A0901[is.na(A$A0901) & A$E8033100==-3]<-0
table(A$E8012901, useNA="always")
table(A$A0901, useNA="always")
A$A0902<-NA
A$A0902[A$E8012902==0]<-0
A$A0902[A$E8012902==99]<-0
A$A0902[A$E8012902>=1 & A$E8012902<99]<-A$E8012902[A$E8012902>=1 &
A$E8012902<99]
A$A0902[is.na(A$A0902) & A$E8033000==-4]<-0
A$A0902[is.na(A$A0902) & A$E8033000==-3]<-0
A$A0902[is.na(A$A0902) & A$E8033000 >= 199300]<-0
A$A0902[is.na(A$A0902) & A$E8033100==0]<-0
A$A0902[is.na(A$A0902) & A$E8033100==-3]<-0
table(A$E8012902, useNA="always")
table(A$A0902, useNA="always")
A$A0903<-NA
A$A0903[A$E8012903==0]<-0
A$A0903[A$E8012903==99]<-0
A$A0903[A$E8012903>=1 & A$E8012903<99]<-A$E8012903[A$E8012903>=1 &
A$E8012903<99]
A$A0903[is.na(A$A0903) & A$E8033000==-4]<-0
A$A0903[is.na(A$A0903) & A$E8033000==-3]<-0
A$A0903[is.na(A$A0903) & A$E8033000 >= 199300]<-0
A$A0903[is.na(A$A0903) & A$E8033100==0]<-0
A$A0903[is.na(A$A0903) & A$E8033100==-3]<-0
table(A$E8012903, useNA="always")
table(A$A0903, useNA="always")
A$A0904<-NA
```

© 2023 Silver IA et al. *JAMA Network Open.*

```
A$A0904[A$E8012904==0]<-0
A$A0904[A$E8012904==99]<-0
A$A0904[A$E8012904>=1 & A$E8012904<99]<-A$E8012904[A$E8012904>=1 &
A$E8012904<99]
A$A0904[is.na(A$A0904) & A$E8033000==-4]<-0
A$A0904[is.na(A$A0904) & A$E8033000==-3]<-0
A$A0904[is.na(A$A0904) & A$E8033000 >= 199300]<-0
A$A0904[is.na(A$A0904) & A$E8033100==0]<-0
A$A0904[is.na(A$A0904) & A$E8033100==-3]<-0
table(A$E8012904, useNA="always")
table(A$A0904, useNA="always")
A$A0905<-NA
A$A0905[A$E8012905==0]<-0
A$A0905[A$E8012905==99]<-0
A$A0905[A$E8012905>=1 & A$E8012905<99]<-A$E8012905[A$E8012905>=1 &
A$E8012905<99]
A$A0905[is.na(A$A0905) & A$E8033000==-4]<-0
A$A0905[is.na(A$A0905) & A$E8033000==-3]<-0
A$A0905[is.na(A$A0905) & A$E8033000 >= 199300]<-0
A$A0905[is.na(A$A0905) & A$E8033100==0]<-0
A$A0905[is.na(A$A0905) & A$E8033100==-3]<-0
table(A$E8012905, useNA="always")
table(A$A0905, useNA="always")
A$A0906<-NA
A$A0906[A$E8012906==0]<-0
A$A0906[A$E8012906==99]<-0
A$A0906[A$E8012906>=1 & A$E8012906<99]<-A$E8012906[A$E8012906>=1 &
A$E8012906<99]
A$A0906[is.na(A$A0906) & A$E8033000==-4]<-0
A$A0906[is.na(A$A0906) & A$E8033000==-3]<-0
A$A0906[is.na(A$A0906) & A$E8033000 >= 199300]<-0
A$A0906[is.na(A$A0906) & A$E8033100==0]<-0
A$A0906[is.na(A$A0906) & A$E8033100==-3]<-0
table(A$E8012906, useNA="always")
table(A$A0906, useNA="always")
A$A0907<-NA
A$A0907[A$E8012907==0]<-0
A$A0907[A$E8012907==99]<-0
A$A0907[A$E8012907>=1 & A$E8012907<99]<-A$E8012907[A$E8012907>=1 &
A$E8012907<99]
A$A0907[is.na(A$A0907) & A$E8033000==-4]<-0
A$A0907[is.na(A$A0907) & A$E8033000==-3]<-0
A$A0907[is.na(A$A0907) & A$E8033000 >= 199300]<-0
A$A0907[is.na(A$A0907) & A$E8033100==0]<-0
A$A0907[is.na(A$A0907) & A$E8033100==-3]<-0
table(A$E8012907, useNA="always")
table(A$A0907, useNA="always")
A$A0908<-NA
A$A0908[A$E8012908==0]<-0
A$A0908[A$E8012908==99]<-0
A$A0908[A$E8012908>=1 & A$E8012908<99]<-A$E8012908[A$E8012908>=1 &
A$E8012908<99]
A$A0908[is.na(A$A0908) & A$E8033000==-4]<-0
A$A0908[is.na(A$A0908) & A$E8033000==-3]<-0
A$A0908[is.na(A$A0908) & A$E8033000 >= 199300]<-0
A$A0908[is.na(A$A0908) & A$E8033100==0]<-0
```

© 2023 Silver IA et al. *JAMA Network Open.*

```
A$A0908[is.na(A$A0908) & A$E8033100==-3]<-0
table(A$E8012908, useNA="always")
table(A$A0908, useNA="always")
A$A0909<-NA
A$A0909[A$E8012909==0]<-0
A$A0909[A$E8012909==99]<-0
A$A0909[A$E8012909>=1 & A$E8012909<99]<-A$E8012909[A$E8012909>=1 &
A$E8012909<99]
A$A0909[is.na(A$A0909) & A$E8033000==-4]<-0
A$A0909[is.na(A$A0909) & A$E8033000==-3]<-0
A$A0909[is.na(A$A0909) & A$E8033000 >= 199300]<-0
A$A0909[is.na(A$A0909) & A$E8033100==0]<-0
A$A0909[is.na(A$A0909) & A$E8033100==-3]<-0
table(A$E8012909, useNA="always")
table(A$A0909, useNA="always")
A$A0910<-NA
A$A0910[A$E8012910==0]<-0
A$A0910[A$E8012910==99]<-0
A$A0910[A$E8012910>=1 & A$E8012910<99]<-A$E8012910[A$E8012910>=1 &
A$E8012910<99]
A$A0910[is.na(A$A0910) & A$E8033000==-4]<-0
A$A0910[is.na(A$A0910) & A$E8033000==-3]<-0
A$A0910[is.na(A$A0910) & A$E8033000 >= 199300]<-0
A$A0910[is.na(A$A0910) & A$E8033100==0]<-0
A$A0910[is.na(A$A0910) & A$E8033100==-3]<-0
table(A$E8012910, useNA="always")
table(A$A0910, useNA="always")
A$A0911<-NA
A$A0911[A$E8012911==0]<-0
A$A0911[A$E8012911==99]<-0
A$A0911[A$E8012911>=1 & A$E8012911<99]<-A$E8012911[A$E8012911>=1 &
A$E8012911<99]
A$A0911[is.na(A$A0911) & A$E8033000==-4]<-0
A$A0911[is.na(A$A0911) & A$E8033000==-3]<-0
A$A0911[is.na(A$A0911) & A$E8033000 >= 199300]<-0
A$A0911[is.na(A$A0911) & A$E8033100==0]<-0
A$A0911[is.na(A$A0911) & A$E8033100==-3]<-0
table(A$E8012911, useNA="always")
table(A$A0911, useNA="always")
A$A0912<-NA
A$A0912[A$E8012912==0]<-0
A$A0912[A$E8012912==99]<-0
A$A0912[A$E8012912>=1 & A$E8012912<99]<-A$E8012912[A$E8012912>=1 &
A$E8012912<99]
A$A0912[is.na(A$A0912) & A$E8033000==-4]<-0
A$A0912[is.na(A$A0912) & A$E8033000==-3]<-0
A$A0912[is.na(A$A0912) & A$E8033000 >= 199300]<-0
A$A0912[is.na(A$A0912) & A$E8033100==0]<-0
A$A0912[is.na(A$A0912) & A$E8033100==-3]<-0
table(A$E8012912, useNA="always")
table(A$A0912, useNA="always")
```

© 2023 Silver IA et al. *JAMA Network Open.*

```
A$A09<-
rowSums(data.frame(A$A0901,A$A0902,A$A0903,A$A0904,A$A0905,A$A0906,A$A0907,A$
A0908,A$A0909,A$A0910,A$A0911,A$A0912), na.rm = T)
table(A$A09)




### Arrest (2010)

table(A$E8013001, useNA="always")
table(A$E8013002)
table(A$E8013003)
table(A$E8013004)
table(A$E8013005)
table(A$E8013006)
table(A$E8013007)
table(A$E8013008)
table(A$E8013009)
table(A$E8013010)
table(A$E8013011)
table(A$E8013012)

A$A1001<-NA
A$A1001[A$E8013001==0]<-0
A$A1001[A$E8013001==99]<-0
A$A1001[A$E8013001>=1 & A$E8013001<99]<-A$E8013001[A$E8013001>=1 &
A$E8013001<99]
A$A1001[is.na(A$A1001) & A$E8033000==-4]<-0
A$A1001[is.na(A$A1001) & A$E8033000==-3]<-0
A$A1001[is.na(A$A1001) & A$E8033000 >= 199300]<-0
A$A1001[is.na(A$A1001) & A$E8033100==0]<-0
A$A1001[is.na(A$A1001) & A$E8033100==-3]<-0
table(A$E8013001, useNA="always")
table(A$A1001, useNA="always")
A$A1002<-NA
A$A1002[A$E8013002==0]<-0
A$A1002[A$E8013002==99]<-0
A$A1002[A$E8013002>=1 & A$E8013002<99]<-A$E8013002[A$E8013002>=1 &
A$E8013002<99]
A$A1002[is.na(A$A1002) & A$E8033000==-4]<-0
A$A1002[is.na(A$A1002) & A$E8033000==-3]<-0
A$A1002[is.na(A$A1002) & A$E8033000 >= 199300]<-0
A$A1002[is.na(A$A1002) & A$E8033100==0]<-0
A$A1002[is.na(A$A1002) & A$E8033100==-3]<-0
table(A$E8013002, useNA="always")
table(A$A1002, useNA="always")
A$A1003<-NA
A$A1003[A$E8013003==0]<-0
A$A1003[A$E8013003==99]<-0
A$A1003[A$E8013003>=1 & A$E8013003<99]<-A$E8013003[A$E8013003>=1 &
A$E8013003<99]
A$A1003[is.na(A$A1003) & A$E8033000==-4]<-0
A$A1003[is.na(A$A1003) & A$E8033000==-3]<-0
A$A1003[is.na(A$A1003) & A$E8033000 >= 199300]<-0
A$A1003[is.na(A$A1003) & A$E8033100==0]<-0
```

```
A$A1003[is.na(A$A1003) & A$E8033100==-3]<-0
table(A$E8013003, useNA="always")
table(A$A1003, useNA="always")
A$A1004<-NA
A$A1004[A$E8013004==0]<-0
A$A1004[A$E8013004==99]<-0
A$A1004[A$E8013004>=1 & A$E8013004<99]<-A$E8013004[A$E8013004>=1 &
A$E8013004<99]
A$A1004[is.na(A$A1004) & A$E8033000==-4]<-0
A$A1004[is.na(A$A1004) & A$E8033000==-3]<-0
A$A1004[is.na(A$A1004) & A$E8033000 >= 199300]<-0
A$A1004[is.na(A$A1004) & A$E8033100==0]<-0
A$A1004[is.na(A$A1004) & A$E8033100==-3]<-0
table(A$E8013004, useNA="always")
table(A$A1004, useNA="always")
A$A1005<-NA
A$A1005[A$E8013005==0]<-0
A$A1005[A$E8013005==99]<-0
A$A1005[A$E8013005>=1 & A$E8013005<99]<-A$E8013005[A$E8013005>=1 &
A$E8013005<99]
A$A1005[is.na(A$A1005) & A$E8033000==-4]<-0
A$A1005[is.na(A$A1005) & A$E8033000==-3]<-0
A$A1005[is.na(A$A1005) & A$E8033000 >= 199300]<-0
A$A1005[is.na(A$A1005) & A$E8033100==0]<-0
A$A1005[is.na(A$A1005) & A$E8033100==-3]<-0
table(A$E8013005, useNA="always")
table(A$A1005, useNA="always")
A$A1006<-NA
A$A1006[A$E8013006==0]<-0
A$A1006[A$E8013006==99]<-0
A$A1006[A$E8013006>=1 & A$E8013006<99]<-A$E8013006[A$E8013006>=1 &
A$E8013006<99]
A$A1006[is.na(A$A1006) & A$E8033000==-4]<-0
A$A1006[is.na(A$A1006) & A$E8033000==-3]<-0
A$A1006[is.na(A$A1006) & A$E8033000 >= 199300]<-0
A$A1006[is.na(A$A1006) & A$E8033100==0]<-0
A$A1006[is.na(A$A1006) & A$E8033100==-3]<-0
table(A$E8013006, useNA="always")
table(A$A1006, useNA="always")
A$A1007<-NA
A$A1007[A$E8013007==0]<-0
A$A1007[A$E8013007==99]<-0
A$A1007[A$E8013007>=1 & A$E8013007<99]<-A$E8013007[A$E8013007>=1 &
A$E8013007<99]
A$A1007[is.na(A$A1007) & A$E8033000==-4]<-0
A$A1007[is.na(A$A1007) & A$E8033000==-3]<-0
A$A1007[is.na(A$A1007) & A$E8033000 >= 199300]<-0
A$A1007[is.na(A$A1007) & A$E8033100==0]<-0
A$A1007[is.na(A$A1007) & A$E8033100==-3]<-0
table(A$E8013007, useNA="always")
table(A$A1007, useNA="always")
A$A1008<-NA
A$A1008[A$E8013008==0]<-0
A$A1008[A$E8013008==99]<-0
A$A1008[A$E8013008>=1 & A$E8013008<99]<-A$E8013008[A$E8013008>=1 &
A$E8013008<99]
```

© 2023 Silver IA et al. *JAMA Network Open.*

```
A$A1008[is.na(A$A1008) & A$E8033000==-4]<-0
A$A1008[is.na(A$A1008) & A$E8033000==-3]<-0
A$A1008[is.na(A$A1008) & A$E8033000 >= 199300]<-0
A$A1008[is.na(A$A1008) & A$E8033100==0]<-0
A$A1008[is.na(A$A1008) & A$E8033100==-3]<-0
table(A$E8013008, useNA="always")
table(A$A1008, useNA="always")
A$A1009<-NA
A$A1009[A$E8013009==0]<-0
A$A1009[A$E8013009==99]<-0
A$A1009[A$E8013009>=1 & A$E8013009<99]<-A$E8013009[A$E8013009>=1 &
A$E8013009<99]
A$A1009[is.na(A$A1009) & A$E8033000==-4]<-0
A$A1009[is.na(A$A1009) & A$E8033000==-3]<-0
A$A1009[is.na(A$A1009) & A$E8033000 >= 199300]<-0
A$A1009[is.na(A$A1009) & A$E8033100==0]<-0
A$A1009[is.na(A$A1009) & A$E8033100==-3]<-0
table(A$E8013009, useNA="always")
table(A$A1009, useNA="always")
A$A1010<-NA
A$A1010[A$E8013010==0]<-0
A$A1010[A$E8013010==99]<-0
A$A1010[A$E8013010>=1 & A$E8013010<99]<-A$E8013010[A$E8013010>=1 &
A$E8013010<99]
A$A1010[is.na(A$A1010) & A$E8033000==-4]<-0
A$A1010[is.na(A$A1010) & A$E8033000==-3]<-0
A$A1010[is.na(A$A1010) & A$E8033000 >= 199300]<-0
A$A1010[is.na(A$A1010) & A$E8033100==0]<-0
A$A1010[is.na(A$A1010) & A$E8033100==-3]<-0
table(A$E8013010, useNA="always")
table(A$A1010, useNA="always")
A$A1011<-NA
A$A1011[A$E8013011==0]<-0
A$A1011[A$E8013011==99]<-0
A$A1011[A$E8013011>=1 & A$E8013011<99]<-A$E8013011[A$E8013011>=1 &
A$E8013011<99]
A$A1011[is.na(A$A1011) & A$E8033000==-4]<-0
A$A1011[is.na(A$A1011) & A$E8033000==-3]<-0
A$A1011[is.na(A$A1011) & A$E8033000 >= 199300]<-0
A$A1011[is.na(A$A1011) & A$E8033100==0]<-0
A$A1011[is.na(A$A1011) & A$E8033100==-3]<-0
table(A$E8013011, useNA="always")
table(A$A1011, useNA="always")
A$A1012<-NA
A$A1012[A$E8013012==0]<-0
A$A1012[A$E8013012==99]<-0
A$A1012[A$E8013012>=1 & A$E8013012<99]<-A$E8013012[A$E8013012>=1 &
A$E8013012<99]
A$A1012[is.na(A$A1012) & A$E8033000==-4]<-0
A$A1012[is.na(A$A1012) & A$E8033000==-3]<-0
A$A1012[is.na(A$A1012) & A$E8033000 >= 199300]<-0
A$A1012[is.na(A$A1012) & A$E8033100==0]<-0
A$A1012[is.na(A$A1012) & A$E8033100==-3]<-0
table(A$E8013012, useNA="always")
table(A$A1012, useNA="always")
```

© 2023 Silver IA et al. *JAMA Network Open.*

```
A$A10<-
rowSums(data.frame(A$A1001,A$A1002,A$A1003,A$A1004,A$A1005,A$A1006,A$A1007,A$
A1008,A$A1009,A$A1010,A$A1011,A$A1012), na.rm = T)
table(A$A10)



### Arrest (2011)

table(A$E8013101, useNA="always")
table(A$E8013102)
table(A$E8013103)
table(A$E8013104)
table(A$E8013105)
table(A$E8013106)
table(A$E8013107)
table(A$E8013108)
table(A$E8013109)
table(A$E8013110)
table(A$E8013111)
table(A$E8013112)

A$A1101<-NA
A$A1101[A$E8013101==0]<-0
A$A1101[A$E8013101==99]<-0
A$A1101[A$E8013101>=1 & A$E8013101<99]<-A$E8013101[A$E8013101>=1 &
A$E8013101<99]
A$A1101[is.na(A$A1101) & A$E8033000==-4]<-0
A$A1101[is.na(A$A1101) & A$E8033000==-3]<-0
A$A1101[is.na(A$A1101) & A$E8033000 >= 199300]<-0
A$A1101[is.na(A$A1101) & A$E8033100==0]<-0
A$A1101[is.na(A$A1101) & A$E8033100==-3]<-0
table(A$E8013101, useNA="always")
table(A$A1101, useNA="always")
A$A1102<-NA
A$A1102[A$E8013102==0]<-0
A$A1102[A$E8013102==99]<-0
A$A1102[A$E8013102>=1 & A$E8013102<99]<-A$E8013102[A$E8013102>=1 &
A$E8013102<99]
A$A1102[is.na(A$A1102) & A$E8033000==-4]<-0
A$A1102[is.na(A$A1102) & A$E8033000==-3]<-0
A$A1102[is.na(A$A1102) & A$E8033000 >= 199300]<-0
A$A1102[is.na(A$A1102) & A$E8033100==0]<-0
A$A1102[is.na(A$A1102) & A$E8033100==-3]<-0
table(A$E8013102, useNA="always")
table(A$A1102, useNA="always")
A$A1103<-NA
A$A1103[A$E8013103==0]<-0
A$A1103[A$E8013103==99]<-0
A$A1103[A$E8013103>=1 & A$E8013103<99]<-A$E8013103[A$E8013103>=1 &
A$E8013103<99]
A$A1103[is.na(A$A1103) & A$E8033000==-4]<-0
A$A1103[is.na(A$A1103) & A$E8033000==-3]<-0
```

© 2023 Silver IA et al. *JAMA Network Open.*

```
A$A1103[is.na(A$A1103) & A$E8033000 >= 199300]<-0
A$A1103[is.na(A$A1103) & A$E8033100==0]<-0
A$A1103[is.na(A$A1103) & A$E8033100==-3]<-0
table(A$E8013103, useNA="always")
table(A$A1103, useNA="always")
A$A1104<-NA
A$A1104[A$E8013104==0]<-0
A$A1104[A$E8013104==99]<-0
A$A1104[A$E8013104>=1 & A$E8013104<99]<-A$E8013104[A$E8013104>=1 &
A$E8013104<99]
A$A1104[is.na(A$A1104) & A$E8033000==-4]<-0
A$A1104[is.na(A$A1104) & A$E8033000==-3]<-0
A$A1104[is.na(A$A1104) & A$E8033000 >= 199300]<-0
A$A1104[is.na(A$A1104) & A$E8033100==0]<-0
A$A1104[is.na(A$A1104) & A$E8033100==-3]<-0
table(A$E8013104, useNA="always")
table(A$A1104, useNA="always")
A$A1105<-NA
A$A1105[A$E8013105==0]<-0
A$A1105[A$E8013105==99]<-0
A$A1105[A$E8013105>=1 & A$E8013105<99]<-A$E8013105[A$E8013105>=1 &
A$E8013105<99]
A$A1105[is.na(A$A1105) & A$E8033000==-4]<-0
A$A1105[is.na(A$A1105) & A$E8033000==-3]<-0
A$A1105[is.na(A$A1105) & A$E8033000 >= 199300]<-0
A$A1105[is.na(A$A1105) & A$E8033100==0]<-0
A$A1105[is.na(A$A1105) & A$E8033100==-3]<-0
table(A$E8013105, useNA="always")
table(A$A1105, useNA="always")
A$A1106<-NA
A$A1106[A$E8013106==0]<-0
A$A1106[A$E8013106==99]<-0
A$A1106[A$E8013106>=1 & A$E8013106<99]<-A$E8013106[A$E8013106>=1 &
A$E8013106<99]
A$A1106[is.na(A$A1106) & A$E8033000==-4]<-0
A$A1106[is.na(A$A1106) & A$E8033000==-3]<-0
A$A1106[is.na(A$A1106) & A$E8033000 >= 199300]<-0
A$A1106[is.na(A$A1106) & A$E8033100==0]<-0
A$A1106[is.na(A$A1106) & A$E8033100==-3]<-0
table(A$E8013106, useNA="always")
table(A$A1106, useNA="always")
A$A1107<-NA
A$A1107[A$E8013107==0]<-0
A$A1107[A$E8013107==99]<-0
A$A1107[A$E8013107>=1 & A$E8013107<99]<-A$E8013107[A$E8013107>=1 &
A$E8013107<99]
A$A1107[is.na(A$A1107) & A$E8033000==-4]<-0
A$A1107[is.na(A$A1107) & A$E8033000==-3]<-0
A$A1107[is.na(A$A1107) & A$E8033000 >= 199300]<-0
A$A1107[is.na(A$A1107) & A$E8033100==0]<-0
A$A1107[is.na(A$A1107) & A$E8033100==-3]<-0
table(A$E8013107, useNA="always")
table(A$A1107, useNA="always")
A$A1108<-NA
A$A1108[A$E8013108==0]<-0
A$A1108[A$E8013108==99]<-0
```

© 2023 Silver IA et al. *JAMA Network Open.*

```
A$A1108[A$E8013108>=1 & A$E8013108<99]<-A$E8013108[A$E8013108>=1 &
A$E8013108<99]
A$A1108[is.na(A$A1108) & A$E8033000==-4]<-0
A$A1108[is.na(A$A1108) & A$E8033000==-3]<-0
A$A1108[is.na(A$A1108) & A$E8033000 >= 199300]<-0
A$A1108[is.na(A$A1108) & A$E8033100==0]<-0
A$A1108[is.na(A$A1108) & A$E8033100==-3]<-0
table(A$E8013108, useNA="always")
table(A$A1108, useNA="always")
A$A1109<-NA
A$A1109[A$E8013109==0]<-0
A$A1109[A$E8013109==99]<-0
A$A1109[A$E8013109>=1 & A$E8013109<99]<-A$E8013109[A$E8013109>=1 &
A$E8013109<99]
A$A1109[is.na(A$A1109) & A$E8033000==-4]<-0
A$A1109[is.na(A$A1109) & A$E8033000==-3]<-0
A$A1109[is.na(A$A1109) & A$E8033000 >= 199300]<-0
A$A1109[is.na(A$A1109) & A$E8033100==0]<-0
A$A1109[is.na(A$A1109) & A$E8033100==-3]<-0
table(A$E8013109, useNA="always")
table(A$A1109, useNA="always")
A$A1110<-NA
A$A1110[A$E8013110==0]<-0
A$A1110[A$E8013110==99]<-0
A$A1110[A$E8013110>=1 & A$E8013110<99]<-A$E8013110[A$E8013110>=1 &
A$E8013110<99]
A$A1110[is.na(A$A1110) & A$E8033000==-4]<-0
A$A1110[is.na(A$A1110) & A$E8033000==-3]<-0
A$A1110[is.na(A$A1110) & A$E8033000 >= 199300]<-0
A$A1110[is.na(A$A1110) & A$E8033100==0]<-0
A$A1110[is.na(A$A1110) & A$E8033100==-3]<-0
table(A$E8013110, useNA="always")
table(A$A1110, useNA="always")
A$A1111<-NA
A$A1111[A$E8013111==0]<-0
A$A1111[A$E8013111==99]<-0
A$A1111[A$E8013111>=1 & A$E8013111<99]<-A$E8013111[A$E8013111>=1 &
A$E8013111<99]
A$A1111[is.na(A$A1111) & A$E8033000==-4]<-0
A$A1111[is.na(A$A1111) & A$E8033000==-3]<-0
A$A1111[is.na(A$A1111) & A$E8033000 >= 199300]<-0
A$A1111[is.na(A$A1111) & A$E8033100==0]<-0
A$A1111[is.na(A$A1111) & A$E8033100==-3]<-0
table(A$E8013111, useNA="always")
table(A$A1111, useNA="always")
A$A1112<-NA
A$A1112[A$E8013112==0]<-0
A$A1112[A$E8013112==99]<-0
A$A1112[A$E8013112>=1 & A$E8013112<99]<-A$E8013112[A$E8013112>=1 &
A$E8013112<99]
A$A1112[is.na(A$A1112) & A$E8033000==-4]<-0
A$A1112[is.na(A$A1112) & A$E8033000==-3]<-0
A$A1112[is.na(A$A1112) & A$E8033000 >= 199300]<-0
A$A1112[is.na(A$A1112) & A$E8033100==0]<-0
A$A1112[is.na(A$A1112) & A$E8033100==-3]<-0
table(A$E8013112, useNA="always")
```

© 2023 Silver IA et al. *JAMA Network Open.*

```
table(A$A1112, useNA="always")



A$A11<-
rowSums(data.frame(A$A1101,A$A1102,A$A1103,A$A1104,A$A1105,A$A1106,A$A1107,A$
A1108,A$A1109,A$A1110,A$A1111,A$A1112), na.rm = T)
table(A$A11)



### Arrest (2012)

table(A$E8013201, useNA="always")
table(A$E8013202)
table(A$E8013203)
table(A$E8013204)
table(A$E8013205)
table(A$E8013206)
table(A$E8013207)
table(A$E8013208)
table(A$E8013209)
table(A$E8013210)
table(A$E8013211)
table(A$E8013212)

A$A1201<-NA
A$A1201[A$E8013201==0]<-0
A$A1201[A$E8013201==99]<-0
A$A1201[A$E8013201>=1 & A$E8013201<99]<-A$E8013201[A$E8013201>=1 &
A$E8013201<99]
A$A1201[is.na(A$A1201) & A$E8033000==-4]<-0
A$A1201[is.na(A$A1201) & A$E8033000==-3]<-0
A$A1201[is.na(A$A1201) & A$E8033000 >= 199300]<-0
A$A1201[is.na(A$A1201) & A$E8033100==0]<-0
A$A1201[is.na(A$A1201) & A$E8033100==-3]<-0
table(A$E8013201, useNA="always")
table(A$A1201, useNA="always")
A$A1202<-NA
A$A1202[A$E8013202==0]<-0
A$A1202[A$E8013202==99]<-0
A$A1202[A$E8013202>=1 & A$E8013202<99]<-A$E8013202[A$E8013202>=1 &
A$E8013202<99]
A$A1202[is.na(A$A1202) & A$E8033000==-4]<-0
A$A1202[is.na(A$A1202) & A$E8033000==-3]<-0
A$A1202[is.na(A$A1202) & A$E8033000 >= 199300]<-0
A$A1202[is.na(A$A1202) & A$E8033100==0]<-0
A$A1202[is.na(A$A1202) & A$E8033100==-3]<-0
table(A$E8013202, useNA="always")
table(A$A1202, useNA="always")
A$A1203<-NA
A$A1203[A$E8013203==0]<-0
A$A1203[A$E8013203==99]<-0
A$A1203[A$E8013203>=1 & A$E8013203<99]<-A$E8013203[A$E8013203>=1 &
A$E8013203<99]
```

© 2023 Silver IA et al. *JAMA Network Open.*

```
A$A1203[is.na(A$A1203) & A$E8033000==-4]<-0
A$A1203[is.na(A$A1203) & A$E8033000==-3]<-0
A$A1203[is.na(A$A1203) & A$E8033000 >= 199300]<-0
A$A1203[is.na(A$A1203) & A$E8033100==0]<-0
A$A1203[is.na(A$A1203) & A$E8033100==-3]<-0
table(A$E8013203, useNA="always")
table(A$A1203, useNA="always")
A$A1204<-NA
A$A1204[A$E8013204==0]<-0
A$A1204[A$E8013204==99]<-0
A$A1204[A$E8013204>=1 & A$E8013204<99]<-A$E8013204[A$E8013204>=1 &
A$E8013204<99]
A$A1204[is.na(A$A1204) & A$E8033000==-4]<-0
A$A1204[is.na(A$A1204) & A$E8033000==-3]<-0
A$A1204[is.na(A$A1204) & A$E8033000 >= 199300]<-0
A$A1204[is.na(A$A1204) & A$E8033100==0]<-0
A$A1204[is.na(A$A1204) & A$E8033100==-3]<-0
table(A$E8013204, useNA="always")
table(A$A1204, useNA="always")
A$A1205<-NA
A$A1205[A$E8013205==0]<-0
A$A1205[A$E8013205==99]<-0
A$A1205[A$E8013205>=1 & A$E8013205<99]<-A$E8013205[A$E8013205>=1 &
A$E8013205<99]
A$A1205[is.na(A$A1205) & A$E8033000==-4]<-0
A$A1205[is.na(A$A1205) & A$E8033000==-3]<-0
A$A1205[is.na(A$A1205) & A$E8033000 >= 199300]<-0
A$A1205[is.na(A$A1205) & A$E8033100==0]<-0
A$A1205[is.na(A$A1205) & A$E8033100==-3]<-0
table(A$E8013205, useNA="always")
table(A$A1205, useNA="always")
A$A1206<-NA
A$A1206[A$E8013206==0]<-0
A$A1206[A$E8013206==99]<-0
A$A1206[A$E8013206>=1 & A$E8013206<99]<-A$E8013206[A$E8013206>=1 &
A$E8013206<99]
A$A1206[is.na(A$A1206) & A$E8033000==-4]<-0
A$A1206[is.na(A$A1206) & A$E8033000==-3]<-0
A$A1206[is.na(A$A1206) & A$E8033000 >= 199300]<-0
A$A1206[is.na(A$A1206) & A$E8033100==0]<-0
A$A1206[is.na(A$A1206) & A$E8033100==-3]<-0
table(A$E8013206, useNA="always")
table(A$A1206, useNA="always")
A$A1207<-NA
A$A1207[A$E8013207==0]<-0
A$A1207[A$E8013207==99]<-0
A$A1207[A$E8013207>=1 & A$E8013207<99]<-A$E8013207[A$E8013207>=1 &
A$E8013207<99]
A$A1207[is.na(A$A1207) & A$E8033000==-4]<-0
A$A1207[is.na(A$A1207) & A$E8033000==-3]<-0
A$A1207[is.na(A$A1207) & A$E8033000 >= 199300]<-0
A$A1207[is.na(A$A1207) & A$E8033100==0]<-0
A$A1207[is.na(A$A1207) & A$E8033100==-3]<-0
table(A$E8013207, useNA="always")
table(A$A1207, useNA="always")
A$A1208<-NA
```

© 2023 Silver IA et al. *JAMA Network Open.*

```
A$A1208[A$E8013208==0]<-0
A$A1208[A$E8013208==99]<-0
A$A1208[A$E8013208>=1 & A$E8013208<99]<-A$E8013208[A$E8013208>=1 &
A$E8013208<99]
A$A1208[is.na(A$A1208) & A$E8033000==-4]<-0
A$A1208[is.na(A$A1208) & A$E8033000==-3]<-0
A$A1208[is.na(A$A1208) & A$E8033000 >= 199300]<-0
A$A1208[is.na(A$A1208) & A$E8033100==0]<-0
A$A1208[is.na(A$A1208) & A$E8033100==-3]<-0
table(A$E8013208, useNA="always")
table(A$A1208, useNA="always")
A$A1209<-NA
A$A1209[A$E8013209==0]<-0
A$A1209[A$E8013209==99]<-0
A$A1209[A$E8013209>=1 & A$E8013209<99]<-A$E8013209[A$E8013209>=1 &
A$E8013209<99]
A$A1209[is.na(A$A1209) & A$E8033000==-4]<-0
A$A1209[is.na(A$A1209) & A$E8033000==-3]<-0
A$A1209[is.na(A$A1209) & A$E8033000 >= 199300]<-0
A$A1209[is.na(A$A1209) & A$E8033100==0]<-0
A$A1209[is.na(A$A1209) & A$E8033100==-3]<-0
table(A$E8013209, useNA="always")
table(A$A1209, useNA="always")
A$A1210<-NA
A$A1210[A$E8013210==0]<-0
A$A1210[A$E8013210==99]<-0
A$A1210[A$E8013210>=1 & A$E8013210<99]<-A$E8013210[A$E8013210>=1 &
A$E8013210<99]
A$A1210[is.na(A$A1210) & A$E8033000==-4]<-0
A$A1210[is.na(A$A1210) & A$E8033000==-3]<-0
A$A1210[is.na(A$A1210) & A$E8033000 >= 199300]<-0
A$A1210[is.na(A$A1210) & A$E8033100==0]<-0
A$A1210[is.na(A$A1210) & A$E8033100==-3]<-0
table(A$E8013210, useNA="always")
table(A$A1210, useNA="always")
A$A1211<-NA
A$A1211[A$E8013211==0]<-0
A$A1211[A$E8013211==99]<-0
A$A1211[A$E8013211>=1 & A$E8013211<99]<-A$E8013211[A$E8013211>=1 &
A$E8013211<99]
A$A1211[is.na(A$A1211) & A$E8033000==-4]<-0
A$A1211[is.na(A$A1211) & A$E8033000==-3]<-0
A$A1211[is.na(A$A1211) & A$E8033000 >= 199300]<-0
A$A1211[is.na(A$A1211) & A$E8033100==0]<-0
A$A1211[is.na(A$A1211) & A$E8033100==-3]<-0
table(A$E8013211, useNA="always")
table(A$A1211, useNA="always")
A$A1212<-NA
A$A1212[A$E8013212==0]<-0
A$A1212[A$E8013212==99]<-0
A$A1212[A$E8013212>=1 & A$E8013212<99]<-A$E8013212[A$E8013212>=1 &
A$E8013212<99]
A$A1212[is.na(A$A1212) & A$E8033000==-4]<-0
A$A1212[is.na(A$A1212) & A$E8033000==-3]<-0
A$A1212[is.na(A$A1212) & A$E8033000 >= 199300]<-0
A$A1212[is.na(A$A1212) & A$E8033100==0]<-0
```

```
A$A1212[is.na(A$A1212) & A$E8033100==-3]<-0
table(A$E8013212, useNA="always")
table(A$A1212, useNA="always")




A$A12<-
rowSums(data.frame(A$A1201,A$A1202,A$A1203,A$A1204,A$A1205,A$A1206,A$A1207,A$
A1208,A$A1209,A$A1210,A$A1211,A$A1212), na.rm = T)
table(A$A12)



### Arrest (2013)

table(A$E8013301, useNA="always")
table(A$E8013302)
table(A$E8013303)
table(A$E8013304)
table(A$E8013305)
table(A$E8013306)
table(A$E8013307)
table(A$E8013308)
table(A$E8013309)
table(A$E8013310)
table(A$E8013311)
table(A$E8013312)

A$A1301<-NA
A$A1301[A$E8013301==0]<-0
A$A1301[A$E8013301==99]<-0
A$A1301[A$E8013301>=1 & A$E8013301<99]<-A$E8013301[A$E8013301>=1 &
A$E8013301<99]
A$A1301[is.na(A$A1301) & A$E8033000==-4]<-0
A$A1301[is.na(A$A1301) & A$E8033000==-3]<-0
A$A1301[is.na(A$A1301) & A$E8033000 >= 199300]<-0
A$A1301[is.na(A$A1301) & A$E8033100==0]<-0
A$A1301[is.na(A$A1301) & A$E8033100==-3]<-0
table(A$E8013301, useNA="always")
table(A$A1301, useNA="always")
A$A1302<-NA
A$A1302[A$E8013302==0]<-0
A$A1302[A$E8013302==99]<-0
A$A1302[A$E8013302>=1 & A$E8013302<99]<-A$E8013302[A$E8013302>=1 &
A$E8013302<99]
A$A1302[is.na(A$A1302) & A$E8033000==-4]<-0
A$A1302[is.na(A$A1302) & A$E8033000==-3]<-0
A$A1302[is.na(A$A1302) & A$E8033000 >= 199300]<-0
A$A1302[is.na(A$A1302) & A$E8033100==0]<-0
A$A1302[is.na(A$A1302) & A$E8033100==-3]<-0
table(A$E8013302, useNA="always")
table(A$A1302, useNA="always")
A$A1303<-NA
A$A1303[A$E8013303==0]<-0
A$A1303[A$E8013303==99]<-0
```

© 2023 Silver IA et al. *JAMA Network Open.*

```
A$A1303[A$E8013303>=1 & A$E8013303<99]<-A$E8013303[A$E8013303>=1 &
A$E8013303<99]
A$A1303[is.na(A$A1303) & A$E8033000==-4]<-0
A$A1303[is.na(A$A1303) & A$E8033000==-3]<-0
A$A1303[is.na(A$A1303) & A$E8033000 >= 199300]<-0
A$A1303[is.na(A$A1303) & A$E8033100==0]<-0
A$A1303[is.na(A$A1303) & A$E8033100==-3]<-0
table(A$E8013303, useNA="always")
table(A$A1303, useNA="always")
A$A1304<-NA
A$A1304[A$E8013304==0]<-0
A$A1304[A$E8013304==99]<-0
A$A1304[A$E8013304>=1 & A$E8013304<99]<-A$E8013304[A$E8013304>=1 &
A$E8013304<99]
A$A1304[is.na(A$A1304) & A$E8033000==-4]<-0
A$A1304[is.na(A$A1304) & A$E8033000==-3]<-0
A$A1304[is.na(A$A1304) & A$E8033000 >= 199300]<-0
A$A1304[is.na(A$A1304) & A$E8033100==0]<-0
A$A1304[is.na(A$A1304) & A$E8033100==-3]<-0
table(A$E8013304, useNA="always")
table(A$A1304, useNA="always")
A$A1305<-NA
A$A1305[A$E8013305==0]<-0
A$A1305[A$E8013305==99]<-0
A$A1305[A$E8013305>=1 & A$E8013305<99]<-A$E8013305[A$E8013305>=1 &
A$E8013305<99]
A$A1305[is.na(A$A1305) & A$E8033000==-4]<-0
A$A1305[is.na(A$A1305) & A$E8033000==-3]<-0
A$A1305[is.na(A$A1305) & A$E8033000 >= 199300]<-0
A$A1305[is.na(A$A1305) & A$E8033100==0]<-0
A$A1305[is.na(A$A1305) & A$E8033100==-3]<-0
table(A$E8013305, useNA="always")
table(A$A1305, useNA="always")
A$A1306<-NA
A$A1306[A$E8013306==0]<-0
A$A1306[A$E8013306==99]<-0
A$A1306[A$E8013306>=1 & A$E8013306<99]<-A$E8013306[A$E8013306>=1 &
A$E8013306<99]
A$A1306[is.na(A$A1306) & A$E8033000==-4]<-0
A$A1306[is.na(A$A1306) & A$E8033000==-3]<-0
A$A1306[is.na(A$A1306) & A$E8033000 >= 199300]<-0
A$A1306[is.na(A$A1306) & A$E8033100==0]<-0
A$A1306[is.na(A$A1306) & A$E8033100==-3]<-0
table(A$E8013306, useNA="always")
table(A$A1306, useNA="always")
A$A1307<-NA
A$A1307[A$E8013307==0]<-0
A$A1307[A$E8013307==99]<-0
A$A1307[A$E8013307>=1 & A$E8013307<99]<-A$E8013307[A$E8013307>=1 &
A$E8013307<99]
A$A1307[is.na(A$A1307) & A$E8033000==-4]<-0
A$A1307[is.na(A$A1307) & A$E8033000==-3]<-0
A$A1307[is.na(A$A1307) & A$E8033000 >= 199300]<-0
A$A1307[is.na(A$A1307) & A$E8033100==0]<-0
A$A1307[is.na(A$A1307) & A$E8033100==-3]<-0
table(A$E8013307, useNA="always")
```

© 2023 Silver IA et al. *JAMA Network Open.*

```
table(A$A1307, useNA="always")
A$A1308<-NA
A$A1308[A$E8013308==0]<-0
A$A1308[A$E8013308==99]<-0
A$A1308[A$E8013308>=1 & A$E8013308<99]<-A$E8013308[A$E8013308>=1 &
A$E8013308<99]
A$A1308[is.na(A$A1308) & A$E8033000==-4]<-0
A$A1308[is.na(A$A1308) & A$E8033000==-3]<-0
A$A1308[is.na(A$A1308) & A$E8033000 >= 199300]<-0
A$A1308[is.na(A$A1308) & A$E8033100==0]<-0
A$A1308[is.na(A$A1308) & A$E8033100==-3]<-0
table(A$E8013308, useNA="always")
table(A$A1308, useNA="always")
A$A1309<-NA
A$A1309[A$E8013309==0]<-0
A$A1309[A$E8013309==99]<-0
A$A1309[A$E8013309>=1 & A$E8013309<99]<-A$E8013309[A$E8013309>=1 &
A$E8013309<99]
A$A1309[is.na(A$A1309) & A$E8033000==-4]<-0
A$A1309[is.na(A$A1309) & A$E8033000==-3]<-0
A$A1309[is.na(A$A1309) & A$E8033000 >= 199300]<-0
A$A1309[is.na(A$A1309) & A$E8033100==0]<-0
A$A1309[is.na(A$A1309) & A$E8033100==-3]<-0
table(A$E8013309, useNA="always")
table(A$A1309, useNA="always")
A$A1310<-NA
A$A1310[A$E8013310==0]<-0
A$A1310[A$E8013310==99]<-0
A$A1310[A$E8013310>=1 & A$E8013310<99]<-A$E8013310[A$E8013310>=1 &
A$E8013310<99]
A$A1310[is.na(A$A1310) & A$E8033000==-4]<-0
A$A1310[is.na(A$A1310) & A$E8033000==-3]<-0
A$A1310[is.na(A$A1310) & A$E8033000 >= 199300]<-0
A$A1310[is.na(A$A1310) & A$E8033100==0]<-0
A$A1310[is.na(A$A1310) & A$E8033100==-3]<-0
table(A$E8013310, useNA="always")
table(A$A1310, useNA="always")
A$A1311<-NA
A$A1311[A$E8013311==0]<-0
A$A1311[A$E8013311==99]<-0
A$A1311[A$E8013311>=1 & A$E8013311<99]<-A$E8013311[A$E8013311>=1 &
A$E8013311<99]
A$A1311[is.na(A$A1311) & A$E8033000==-4]<-0
A$A1311[is.na(A$A1311) & A$E8033000==-3]<-0
A$A1311[is.na(A$A1311) & A$E8033000 >= 199300]<-0
A$A1311[is.na(A$A1311) & A$E8033100==0]<-0
A$A1311[is.na(A$A1311) & A$E8033100==-3]<-0
table(A$E8013311, useNA="always")
table(A$A1311, useNA="always")
A$A1312<-NA
A$A1312[A$E8013312==0]<-0
A$A1312[A$E8013312==99]<-0
A$A1312[A$E8013312>=1 & A$E8013312<99]<-A$E8013312[A$E8013312>=1 &
A$E8013312<99]
A$A1312[is.na(A$A1312) & A$E8033000==-4]<-0
A$A1312[is.na(A$A1312) & A$E8033000==-3]<-0
```

© 2023 Silver IA et al. *JAMA Network Open.*

```
A$A1312[is.na(A$A1312) & A$E8033000 >= 199300]<-0
A$A1312[is.na(A$A1312) & A$E8033100==0]<-0
A$A1312[is.na(A$A1312) & A$E8033100==-3]<-0
table(A$E8013312, useNA="always")
table(A$A1312, useNA="always")




A$A13<-
rowSums(data.frame(A$A1301,A$A1302,A$A1303,A$A1304,A$A1305,A$A1306,A$A1307,A$
A1308,A$A1309,A$A1310,A$A1311,A$A1312), na.rm = T)
table(A$A13)



### Arrest (2014)

table(A$E8013401, useNA="always")
table(A$E8013402)
table(A$E8013403)
table(A$E8013404)
table(A$E8013405)
table(A$E8013406)
table(A$E8013407)
table(A$E8013408)
table(A$E8013409)
table(A$E8013410)
table(A$E8013411)
table(A$E8013412)

A$A1401<-NA
A$A1401[A$E8013401==0]<-0
A$A1401[A$E8013401==99]<-0
A$A1401[A$E8013401>=1 & A$E8013401<99]<-A$E8013401[A$E8013401>=1 &
A$E8013401<99]
A$A1401[is.na(A$A1401) & A$E8033000==-4]<-0
A$A1401[is.na(A$A1401) & A$E8033000==-3]<-0
A$A1401[is.na(A$A1401) & A$E8033000 >= 199300]<-0
A$A1401[is.na(A$A1401) & A$E8033100==0]<-0
A$A1401[is.na(A$A1401) & A$E8033100==-3]<-0
table(A$E8013401, useNA="always")
table(A$A1401, useNA="always")
A$A1402<-NA
A$A1402[A$E8013402==0]<-0
A$A1402[A$E8013402==99]<-0
A$A1402[A$E8013402>=1 & A$E8013402<99]<-A$E8013402[A$E8013402>=1 &
A$E8013402<99]
A$A1402[is.na(A$A1402) & A$E8033000==-4]<-0
A$A1402[is.na(A$A1402) & A$E8033000==-3]<-0
A$A1402[is.na(A$A1402) & A$E8033000 >= 199300]<-0
A$A1402[is.na(A$A1402) & A$E8033100==0]<-0
A$A1402[is.na(A$A1402) & A$E8033100==-3]<-0
table(A$E8013402, useNA="always")
table(A$A1402, useNA="always")
A$A1403<-NA
```

```
A$A1403[A$E8013403==0]<-0
A$A1403[A$E8013403==99]<-0
A$A1403[A$E8013403>=1 & A$E8013403<99]<-A$E8013403[A$E8013403>=1 &
A$E8013403<99]
A$A1403[is.na(A$A1403) & A$E8033000==-4]<-0
A$A1403[is.na(A$A1403) & A$E8033000==-3]<-0
A$A1403[is.na(A$A1403) & A$E8033000 >= 199300]<-0
A$A1403[is.na(A$A1403) & A$E8033100==0]<-0
A$A1403[is.na(A$A1403) & A$E8033100==-3]<-0
table(A$E8013403, useNA="always")
table(A$A1403, useNA="always")
A$A1404<-NA
A$A1404[A$E8013404==0]<-0
A$A1404[A$E8013404==99]<-0
A$A1404[A$E8013404>=1 & A$E8013404<99]<-A$E8013404[A$E8013404>=1 &
A$E8013404<99]
A$A1404[is.na(A$A1404) & A$E8033000==-4]<-0
A$A1404[is.na(A$A1404) & A$E8033000==-3]<-0
A$A1404[is.na(A$A1404) & A$E8033000 >= 199300]<-0
A$A1404[is.na(A$A1404) & A$E8033100==0]<-0
A$A1404[is.na(A$A1404) & A$E8033100==-3]<-0
table(A$E8013404, useNA="always")
table(A$A1404, useNA="always")
A$A1405<-NA
A$A1405[A$E8013405==0]<-0
A$A1405[A$E8013405==99]<-0
A$A1405[A$E8013405>=1 & A$E8013405<99]<-A$E8013405[A$E8013405>=1 &
A$E8013405<99]
A$A1405[is.na(A$A1405) & A$E8033000==-4]<-0
A$A1405[is.na(A$A1405) & A$E8033000==-3]<-0
A$A1405[is.na(A$A1405) & A$E8033000 >= 199300]<-0
A$A1405[is.na(A$A1405) & A$E8033100==0]<-0
A$A1405[is.na(A$A1405) & A$E8033100==-3]<-0
table(A$E8013405, useNA="always")
table(A$A1405, useNA="always")
A$A1406<-NA
A$A1406[A$E8013406==0]<-0
A$A1406[A$E8013406==99]<-0
A$A1406[A$E8013406>=1 & A$E8013406<99]<-A$E8013406[A$E8013406>=1 &
A$E8013406<99]
A$A1406[is.na(A$A1406) & A$E8033000==-4]<-0
A$A1406[is.na(A$A1406) & A$E8033000==-3]<-0
A$A1406[is.na(A$A1406) & A$E8033000 >= 199300]<-0
A$A1406[is.na(A$A1406) & A$E8033100==0]<-0
A$A1406[is.na(A$A1406) & A$E8033100==-3]<-0
table(A$E8013406, useNA="always")
table(A$A1406, useNA="always")
A$A1407<-NA
A$A1407[A$E8013407==0]<-0
A$A1407[A$E8013407==99]<-0
A$A1407[A$E8013407>=1 & A$E8013407<99]<-A$E8013407[A$E8013407>=1 &
A$E8013407<99]
A$A1407[is.na(A$A1407) & A$E8033000==-4]<-0
A$A1407[is.na(A$A1407) & A$E8033000==-3]<-0
A$A1407[is.na(A$A1407) & A$E8033000 >= 199300]<-0
A$A1407[is.na(A$A1407) & A$E8033100==0]<-0
```

© 2023 Silver IA et al. *JAMA Network Open.*

```
A$A1407[is.na(A$A1407) & A$E8033100==-3]<-0
table(A$E8013407, useNA="always")
table(A$A1407, useNA="always")
A$A1408<-NA
A$A1408[A$E8013408==0]<-0
A$A1408[A$E8013408==99]<-0
A$A1408[A$E8013408>=1 & A$E8013408<99]<-A$E8013408[A$E8013408>=1 &
A$E8013408<99]
A$A1408[is.na(A$A1408) & A$E8033000==-4]<-0
A$A1408[is.na(A$A1408) & A$E8033000==-3]<-0
A$A1408[is.na(A$A1408) & A$E8033000 >= 199300]<-0
A$A1408[is.na(A$A1408) & A$E8033100==0]<-0
A$A1408[is.na(A$A1408) & A$E8033100==-3]<-0
table(A$E8013408, useNA="always")
table(A$A1408, useNA="always")
A$A1409<-NA
A$A1409[A$E8013409==0]<-0
A$A1409[A$E8013409==99]<-0
A$A1409[A$E8013409>=1 & A$E8013409<99]<-A$E8013409[A$E8013409>=1 &
A$E8013409<99]
A$A1409[is.na(A$A1409) & A$E8033000==-4]<-0
A$A1409[is.na(A$A1409) & A$E8033000==-3]<-0
A$A1409[is.na(A$A1409) & A$E8033000 >= 199300]<-0
A$A1409[is.na(A$A1409) & A$E8033100==0]<-0
A$A1409[is.na(A$A1409) & A$E8033100==-3]<-0
table(A$E8013409, useNA="always")
table(A$A1409, useNA="always")
A$A1410<-NA
A$A1410[A$E8013410==0]<-0
A$A1410[A$E8013410==99]<-0
A$A1410[A$E8013410>=1 & A$E8013410<99]<-A$E8013410[A$E8013410>=1 &
A$E8013410<99]
A$A1410[is.na(A$A1410) & A$E8033000==-4]<-0
A$A1410[is.na(A$A1410) & A$E8033000==-3]<-0
A$A1410[is.na(A$A1410) & A$E8033000 >= 199300]<-0
A$A1410[is.na(A$A1410) & A$E8033100==0]<-0
A$A1410[is.na(A$A1410) & A$E8033100==-3]<-0
table(A$E8013410, useNA="always")
table(A$A1410, useNA="always")
A$A1411<-NA
A$A1411[A$E8013411==0]<-0
A$A1411[A$E8013411==99]<-0
A$A1411[A$E8013411>=1 & A$E8013411<99]<-A$E8013411[A$E8013411>=1 &
A$E8013411<99]
A$A1411[is.na(A$A1411) & A$E8033000==-4]<-0
A$A1411[is.na(A$A1411) & A$E8033000==-3]<-0
A$A1411[is.na(A$A1411) & A$E8033000 >= 199300]<-0
A$A1411[is.na(A$A1411) & A$E8033100==0]<-0
A$A1411[is.na(A$A1411) & A$E8033100==-3]<-0
table(A$E8013411, useNA="always")
table(A$A1411, useNA="always")
A$A1412<-NA
A$A1412[A$E8013412==0]<-0
A$A1412[A$E8013412==99]<-0
A$A1412[A$E8013412>=1 & A$E8013412<99]<-A$E8013412[A$E8013412>=1 &
A$E8013412<99]
```

```
A$A1412[is.na(A$A1412) & A$E8033000==-4]<-0
A$A1412[is.na(A$A1412) & A$E8033000==-3]<-0
A$A1412[is.na(A$A1412) & A$E8033000 >= 199300]<-0
A$A1412[is.na(A$A1412) & A$E8033100==0]<-0
A$A1412[is.na(A$A1412) & A$E8033100==-3]<-0
table(A$E8013412, useNA="always")
table(A$A1412, useNA="always")




A$A14<-
rowSums(data.frame(A$A1401,A$A1402,A$A1403,A$A1404,A$A1405,A$A1406,A$A1407,A$
A1408,A$A1409,A$A1410,A$A1411,A$A1412), na.rm = T)
table(A$A14)


### Arrest (2015)

table(A$E8013501, useNA="always")
table(A$E8013502)
table(A$E8013503)
table(A$E8013504)
table(A$E8013505)
table(A$E8013506)
table(A$E8013507)
table(A$E8013508)
table(A$E8013509)
table(A$E8013510)
table(A$E8013511)
table(A$E8013512)

A$A1501<-NA
A$A1501[A$E8013501==0]<-0
A$A1501[A$E8013501==99]<-0
A$A1501[A$E8013501>=1 & A$E8013501<99]<-A$E8013501[A$E8013501>=1 &
A$E8013501<99]
A$A1501[is.na(A$A1501) & A$E8033000==-4]<-0
A$A1501[is.na(A$A1501) & A$E8033000==-3]<-0
A$A1501[is.na(A$A1501) & A$E8033000 >= 199300]<-0
A$A1501[is.na(A$A1501) & A$E8033100==0]<-0
A$A1501[is.na(A$A1501) & A$E8033100==-3]<-0
table(A$E8013501, useNA="always")
table(A$A1501, useNA="always")
A$A1502<-NA
A$A1502[A$E8013502==0]<-0
A$A1502[A$E8013502==99]<-0
A$A1502[A$E8013502>=1 & A$E8013502<99]<-A$E8013502[A$E8013502>=1 &
A$E8013502<99]
A$A1502[is.na(A$A1502) & A$E8033000==-4]<-0
A$A1502[is.na(A$A1502) & A$E8033000==-3]<-0
A$A1502[is.na(A$A1502) & A$E8033000 >= 199300]<-0
A$A1502[is.na(A$A1502) & A$E8033100==0]<-0
A$A1502[is.na(A$A1502) & A$E8033100==-3]<-0
table(A$E8013502, useNA="always")
table(A$A1502, useNA="always")
```

© 2023 Silver IA et al. *JAMA Network Open.*

```
A$A1503<-NA
A$A1503[A$E8013503==0]<-0
A$A1503[A$E8013503==99]<-0
A$A1503[A$E8013503>=1 & A$E8013503<99]<-A$E8013503[A$E8013503>=1 &
A$E8013503<99]
A$A1503[is.na(A$A1503) & A$E8033000==-4]<-0
A$A1503[is.na(A$A1503) & A$E8033000==-3]<-0
A$A1503[is.na(A$A1503) & A$E8033000 >= 199300]<-0
A$A1503[is.na(A$A1503) & A$E8033100==0]<-0
A$A1503[is.na(A$A1503) & A$E8033100==-3]<-0
table(A$E8013503, useNA="always")
table(A$A1503, useNA="always")
A$A1504<-NA
A$A1504[A$E8013504==0]<-0
A$A1504[A$E8013504==99]<-0
A$A1504[A$E8013504>=1 & A$E8013504<99]<-A$E8013504[A$E8013504>=1 &
A$E8013504<99]
A$A1504[is.na(A$A1504) & A$E8033000==-4]<-0
A$A1504[is.na(A$A1504) & A$E8033000==-3]<-0
A$A1504[is.na(A$A1504) & A$E8033000 >= 199300]<-0
A$A1504[is.na(A$A1504) & A$E8033100==0]<-0
A$A1504[is.na(A$A1504) & A$E8033100==-3]<-0
table(A$E8013504, useNA="always")
table(A$A1504, useNA="always")
A$A1505<-NA
A$A1505[A$E8013505==0]<-0
A$A1505[A$E8013505==99]<-0
A$A1505[A$E8013505>=1 & A$E8013505<99]<-A$E8013505[A$E8013505>=1 &
A$E8013505<99]
A$A1505[is.na(A$A1505) & A$E8033000==-4]<-0
A$A1505[is.na(A$A1505) & A$E8033000==-3]<-0
A$A1505[is.na(A$A1505) & A$E8033000 >= 199300]<-0
A$A1505[is.na(A$A1505) & A$E8033100==0]<-0
A$A1505[is.na(A$A1505) & A$E8033100==-3]<-0
table(A$E8013505, useNA="always")
table(A$A1505, useNA="always")
A$A1506<-NA
A$A1506[A$E8013506==0]<-0
A$A1506[A$E8013506==99]<-0
A$A1506[A$E8013506>=1 & A$E8013506<99]<-A$E8013506[A$E8013506>=1 &
A$E8013506<99]
A$A1506[is.na(A$A1506) & A$E8033000==-4]<-0
A$A1506[is.na(A$A1506) & A$E8033000==-3]<-0
A$A1506[is.na(A$A1506) & A$E8033000 >= 199300]<-0
A$A1506[is.na(A$A1506) & A$E8033100==0]<-0
A$A1506[is.na(A$A1506) & A$E8033100==-3]<-0
table(A$E8013506, useNA="always")
table(A$A1506, useNA="always")
A$A1507<-NA
A$A1507[A$E8013507==0]<-0
A$A1507[A$E8013507==99]<-0
A$A1507[A$E8013507>=1 & A$E8013507<99]<-A$E8013507[A$E8013507>=1 &
A$E8013507<99]
A$A1507[is.na(A$A1507) & A$E8033000==-4]<-0
A$A1507[is.na(A$A1507) & A$E8033000==-3]<-0
A$A1507[is.na(A$A1507) & A$E8033000 >= 199300]<-0
```

© 2023 Silver IA et al. *JAMA Network Open.*

```
A$A1507[is.na(A$A1507) & A$E8033100==0]<-0
A$A1507[is.na(A$A1507) & A$E8033100==-3]<-0
table(A$E8013507, useNA="always")
table(A$A1507, useNA="always")
A$A1508<-NA
A$A1508[A$E8013508==0]<-0
A$A1508[A$E8013508==99]<-0
A$A1508[A$E8013508>=1 & A$E8013508<99]<-A$E8013508[A$E8013508>=1 &
A$E8013508<99]
A$A1508[is.na(A$A1508) & A$E8033000==-4]<-0
A$A1508[is.na(A$A1508) & A$E8033000==-3]<-0
A$A1508[is.na(A$A1508) & A$E8033000 >= 199300]<-0
A$A1508[is.na(A$A1508) & A$E8033100==0]<-0
A$A1508[is.na(A$A1508) & A$E8033100==-3]<-0
table(A$E8013508, useNA="always")
table(A$A1508, useNA="always")
A$A1509<-NA
A$A1509[A$E8013509==0]<-0
A$A1509[A$E8013509==99]<-0
A$A1509[A$E8013509>=1 & A$E8013509<99]<-A$E8013509[A$E8013509>=1 &
A$E8013509<99]
A$A1509[is.na(A$A1509) & A$E8033000==-4]<-0
A$A1509[is.na(A$A1509) & A$E8033000==-3]<-0
A$A1509[is.na(A$A1509) & A$E8033000 >= 199300]<-0
A$A1509[is.na(A$A1509) & A$E8033100==0]<-0
A$A1509[is.na(A$A1509) & A$E8033100==-3]<-0
table(A$E8013509, useNA="always")
table(A$A1509, useNA="always")
A$A1510<-NA
A$A1510[A$E8013510==0]<-0
A$A1510[A$E8013510==99]<-0
A$A1510[A$E8013510>=1 & A$E8013510<99]<-A$E8013510[A$E8013510>=1 &
A$E8013510<99]
A$A1510[is.na(A$A1510) & A$E8033000==-4]<-0
A$A1510[is.na(A$A1510) & A$E8033000==-3]<-0
A$A1510[is.na(A$A1510) & A$E8033000 >= 199300]<-0
A$A1510[is.na(A$A1510) & A$E8033100==0]<-0
A$A1510[is.na(A$A1510) & A$E8033100==-3]<-0
table(A$E8013510, useNA="always")
table(A$A1510, useNA="always")
A$A1511<-NA
A$A1511[A$E8013511==0]<-0
A$A1511[A$E8013511==99]<-0
A$A1511[A$E8013511>=1 & A$E8013511<99]<-A$E8013511[A$E8013511>=1 &
A$E8013511<99]
A$A1511[is.na(A$A1511) & A$E8033000==-4]<-0
A$A1511[is.na(A$A1511) & A$E8033000==-3]<-0
A$A1511[is.na(A$A1511) & A$E8033000 >= 199300]<-0
A$A1511[is.na(A$A1511) & A$E8033100==0]<-0
A$A1511[is.na(A$A1511) & A$E8033100==-3]<-0
table(A$E8013511, useNA="always")
table(A$A1511, useNA="always")
A$A1512<-NA
A$A1512[A$E8013512==0]<-0
A$A1512[A$E8013512==99]<-0
```

© 2023 Silver IA et al. *JAMA Network Open.*

```
A$A1512[A$E8013512>=1 & A$E8013512<99]<-A$E8013512[A$E8013512>=1 &
A$E8013512<99]
A$A1512[is.na(A$A1512) & A$E8033000==-4]<-0
A$A1512[is.na(A$A1512) & A$E8033000==-3]<-0
A$A1512[is.na(A$A1512) & A$E8033000 >= 199300]<-0
A$A1512[is.na(A$A1512) & A$E8033100==0]<-0
A$A1512[is.na(A$A1512) & A$E8033100==-3]<-0
table(A$E8013512, useNA="always")
table(A$A1512, useNA="always")




A$A15<-
rowSums(data.frame(A$A1501,A$A1502,A$A1503,A$A1504,A$A1505,A$A1506,A$A1507,A$
A1508,A$A1509,A$A1510,A$A1511,A$A1512), na.rm = T)
table(A$A15)



### Arrest (2016)

table(A$E8013601, useNA="always")
table(A$E8013602)
table(A$E8013603)
table(A$E8013604)
table(A$E8013605)
table(A$E8013606)
table(A$E8013607)
table(A$E8013608)
table(A$E8013609)
table(A$E8013610)
table(A$E8013611)
table(A$E8013612)

A$A1601<-NA
A$A1601[A$E8013601==0]<-0
A$A1601[A$E8013601==99]<-0
A$A1601[A$E8013601>=1 & A$E8013601<99]<-A$E8013601[A$E8013601>=1 &
A$E8013601<99]
A$A1601[is.na(A$A1601) & A$E8033000==-4]<-0
A$A1601[is.na(A$A1601) & A$E8033000==-3]<-0
A$A1601[is.na(A$A1601) & A$E8033000 >= 199300]<-0
A$A1601[is.na(A$A1601) & A$E8033100==0]<-0
A$A1601[is.na(A$A1601) & A$E8033100==-3]<-0
table(A$E8013601, useNA="always")
table(A$A1601, useNA="always")
A$A1602<-NA
A$A1602[A$E8013602==0]<-0
A$A1602[A$E8013602==99]<-0
A$A1602[A$E8013602>=1 & A$E8013602<99]<-A$E8013602[A$E8013602>=1 &
A$E8013602<99]
A$A1602[is.na(A$A1602) & A$E8033000==-4]<-0
A$A1602[is.na(A$A1602) & A$E8033000==-3]<-0
A$A1602[is.na(A$A1602) & A$E8033000 >= 199300]<-0
A$A1602[is.na(A$A1602) & A$E8033100==0]<-0
```

© 2023 Silver IA et al. *JAMA Network Open.*

```
A$A1602[is.na(A$A1602) & A$E8033100==-3]<-0
table(A$E8013602, useNA="always")
table(A$A1602, useNA="always")
A$A1603<-NA
A$A1603[A$E8013603==0]<-0
A$A1603[A$E8013603==99]<-0
A$A1603[A$E8013603>=1 & A$E8013603<99]<-A$E8013603[A$E8013603>=1 &
A$E8013603<99]
A$A1603[is.na(A$A1603) & A$E8033000==-4]<-0
A$A1603[is.na(A$A1603) & A$E8033000==-3]<-0
A$A1603[is.na(A$A1603) & A$E8033000 >= 199300]<-0
A$A1603[is.na(A$A1603) & A$E8033100==0]<-0
A$A1603[is.na(A$A1603) & A$E8033100==-3]<-0
table(A$E8013603, useNA="always")
table(A$A1603, useNA="always")
A$A1604<-NA
A$A1604[A$E8013604==0]<-0
A$A1604[A$E8013604==99]<-0
A$A1604[A$E8013604>=1 & A$E8013604<99]<-A$E8013604[A$E8013604>=1 &
A$E8013604<99]
A$A1604[is.na(A$A1604) & A$E8033000==-4]<-0
A$A1604[is.na(A$A1604) & A$E8033000==-3]<-0
A$A1604[is.na(A$A1604) & A$E8033000 >= 199300]<-0
A$A1604[is.na(A$A1604) & A$E8033100==0]<-0
A$A1604[is.na(A$A1604) & A$E8033100==-3]<-0
table(A$E8013604, useNA="always")
table(A$A1604, useNA="always")
A$A1605<-NA
A$A1605[A$E8013605==0]<-0
A$A1605[A$E8013605==99]<-0
A$A1605[A$E8013605>=1 & A$E8013605<99]<-A$E8013605[A$E8013605>=1 &
A$E8013605<99]
A$A1605[is.na(A$A1605) & A$E8033000==-4]<-0
A$A1605[is.na(A$A1605) & A$E8033000==-3]<-0
A$A1605[is.na(A$A1605) & A$E8033000 >= 199300]<-0
A$A1605[is.na(A$A1605) & A$E8033100==0]<-0
A$A1605[is.na(A$A1605) & A$E8033100==-3]<-0
table(A$E8013605, useNA="always")
table(A$A1605, useNA="always")
A$A1606<-NA
A$A1606[A$E8013606==0]<-0
A$A1606[A$E8013606==99]<-0
A$A1606[A$E8013606>=1 & A$E8013606<99]<-A$E8013606[A$E8013606>=1 &
A$E8013606<99]
A$A1606[is.na(A$A1606) & A$E8033000==-4]<-0
A$A1606[is.na(A$A1606) & A$E8033000==-3]<-0
A$A1606[is.na(A$A1606) & A$E8033000 >= 199300]<-0
A$A1606[is.na(A$A1606) & A$E8033100==0]<-0
A$A1606[is.na(A$A1606) & A$E8033100==-3]<-0
table(A$E8013606, useNA="always")
table(A$A1606, useNA="always")
A$A1607<-NA
A$A1607[A$E8013607==0]<-0
A$A1607[A$E8013607==99]<-0
A$A1607[A$E8013607>=1 & A$E8013607<99]<-A$E8013607[A$E8013607>=1 &
A$E8013607<99]
```

© 2023 Silver IA et al. *JAMA Network Open.*

```
A$A1607[is.na(A$A1607) & A$E8033000==-4]<-0
A$A1607[is.na(A$A1607) & A$E8033000==-3]<-0
A$A1607[is.na(A$A1607) & A$E8033000 >= 199300]<-0
A$A1607[is.na(A$A1607) & A$E8033100==0]<-0
A$A1607[is.na(A$A1607) & A$E8033100==-3]<-0
table(A$E8013607, useNA="always")
table(A$A1607, useNA="always")
A$A1608<-NA
A$A1608[A$E8013608==0]<-0
A$A1608[A$E8013608==99]<-0
A$A1608[A$E8013608>=1 & A$E8013608<99]<-A$E8013608[A$E8013608>=1 &
A$E8013608<99]
A$A1608[is.na(A$A1608) & A$E8033000==-4]<-0
A$A1608[is.na(A$A1608) & A$E8033000==-3]<-0
A$A1608[is.na(A$A1608) & A$E8033000 >= 199300]<-0
A$A1608[is.na(A$A1608) & A$E8033100==0]<-0
A$A1608[is.na(A$A1608) & A$E8033100==-3]<-0
table(A$E8013608, useNA="always")
table(A$A1608, useNA="always")
A$A1609<-NA
A$A1609[A$E8013609==0]<-0
A$A1609[A$E8013609==99]<-0
A$A1609[A$E8013609>=1 & A$E8013609<99]<-A$E8013609[A$E8013609>=1 &
A$E8013609<99]
A$A1609[is.na(A$A1609) & A$E8033000==-4]<-0
A$A1609[is.na(A$A1609) & A$E8033000==-3]<-0
A$A1609[is.na(A$A1609) & A$E8033000 >= 199300]<-0
A$A1609[is.na(A$A1609) & A$E8033100==0]<-0
A$A1609[is.na(A$A1609) & A$E8033100==-3]<-0
table(A$E8013609, useNA="always")
table(A$A1609, useNA="always")
A$A1610<-NA
A$A1610[A$E8013610==0]<-0
A$A1610[A$E8013610==99]<-0
A$A1610[A$E8013610>=1 & A$E8013610<99]<-A$E8013610[A$E8013610>=1 &
A$E8013610<99]
A$A1610[is.na(A$A1610) & A$E8033000==-4]<-0
A$A1610[is.na(A$A1610) & A$E8033000==-3]<-0
A$A1610[is.na(A$A1610) & A$E8033000 >= 199300]<-0
A$A1610[is.na(A$A1610) & A$E8033100==0]<-0
A$A1610[is.na(A$A1610) & A$E8033100==-3]<-0
table(A$E8013610, useNA="always")
table(A$A1610, useNA="always")
A$A1611<-NA
A$A1611[A$E8013611==0]<-0
A$A1611[A$E8013611==99]<-0
A$A1611[A$E8013611>=1 & A$E8013611<99]<-A$E8013611[A$E8013611>=1 &
A$E8013611<99]
A$A1611[is.na(A$A1611) & A$E8033000==-4]<-0
A$A1611[is.na(A$A1611) & A$E8033000==-3]<-0
A$A1611[is.na(A$A1611) & A$E8033000 >= 199300]<-0
A$A1611[is.na(A$A1611) & A$E8033100==0]<-0
A$A1611[is.na(A$A1611) & A$E8033100==-3]<-0
table(A$E8013611, useNA="always")
table(A$A1611, useNA="always")
A$A1612<-NA
```

© 2023 Silver IA et al. *JAMA Network Open.*

```
A$A1612[A$E8013612==0]<-0
A$A1612[A$E8013612==99]<-0
A$A1612[A$E8013612>=1 & A$E8013612<99]<-A$E8013612[A$E8013612>=1 &
A$E8013612<99]
A$A1612[is.na(A$A1612) & A$E8033000==-4]<-0
A$A1612[is.na(A$A1612) & A$E8033000==-3]<-0
A$A1612[is.na(A$A1612) & A$E8033000 >= 199300]<-0
A$A1612[is.na(A$A1612) & A$E8033100==0]<-0
A$A1612[is.na(A$A1612) & A$E8033100==-3]<-0
table(A$E8013612, useNA="always")
table(A$A1612, useNA="always")




A$A16<-
rowSums(data.frame(A$A1601,A$A1602,A$A1603,A$A1604,A$A1605,A$A1606,A$A1607,A$
A1608,A$A1609,A$A1610,A$A1611,A$A1612), na.rm = T)
table(A$A16)



### Arrest (2017)

table(A$E8013701, useNA="always")
table(A$E8013702)
table(A$E8013703)
table(A$E8013704)
table(A$E8013705)
table(A$E8013706)
table(A$E8013707)
table(A$E8013708)
table(A$E8013709)
table(A$E8013710)
table(A$E8013711)
table(A$E8013712)

A$A1701<-NA
A$A1701[A$E8013701==0]<-0
A$A1701[A$E8013701==99]<-0
A$A1701[A$E8013701>=1 & A$E8013701<99]<-A$E8013701[A$E8013701>=1 &
A$E8013701<99]
A$A1701[is.na(A$A1701) & A$E8033000==-4]<-0
A$A1701[is.na(A$A1701) & A$E8033000==-3]<-0
A$A1701[is.na(A$A1701) & A$E8033000 >= 199300]<-0
A$A1701[is.na(A$A1701) & A$E8033100==0]<-0
A$A1701[is.na(A$A1701) & A$E8033100==-3]<-0
table(A$E8013701, useNA="always")
table(A$A1701, useNA="always")
A$A1702<-NA
A$A1702[A$E8013702==0]<-0
A$A1702[A$E8013702==99]<-0
A$A1702[A$E8013702>=1 & A$E8013702<99]<-A$E8013702[A$E8013702>=1 &
A$E8013702<99]
A$A1702[is.na(A$A1702) & A$E8033000==-4]<-0
A$A1702[is.na(A$A1702) & A$E8033000==-3]<-0
```

© 2023 Silver IA et al. *JAMA Network Open.*

```
A$A1702[is.na(A$A1702) & A$E8033000 >= 199300]<-0
A$A1702[is.na(A$A1702) & A$E8033100==0]<-0
A$A1702[is.na(A$A1702) & A$E8033100==-3]<-0
table(A$E8013702, useNA="always")
table(A$A1702, useNA="always")
A$A1703<-NA
A$A1703[A$E8013703==0]<-0
A$A1703[A$E8013703==99]<-0
A$A1703[A$E8013703>=1 & A$E8013703<99]<-A$E8013703[A$E8013703>=1 &
A$E8013703<99]
A$A1703[is.na(A$A1703) & A$E8033000==-4]<-0
A$A1703[is.na(A$A1703) & A$E8033000==-3]<-0
A$A1703[is.na(A$A1703) & A$E8033000 >= 199300]<-0
A$A1703[is.na(A$A1703) & A$E8033100==0]<-0
A$A1703[is.na(A$A1703) & A$E8033100==-3]<-0
table(A$E8013703, useNA="always")
table(A$A1703, useNA="always")
A$A1704<-NA
A$A1704[A$E8013704==0]<-0
A$A1704[A$E8013704==99]<-0
A$A1704[A$E8013704>=1 & A$E8013704<99]<-A$E8013704[A$E8013704>=1 &
A$E8013704<99]
A$A1704[is.na(A$A1704) & A$E8033000==-4]<-0
A$A1704[is.na(A$A1704) & A$E8033000==-3]<-0
A$A1704[is.na(A$A1704) & A$E8033000 >= 199300]<-0
A$A1704[is.na(A$A1704) & A$E8033100==0]<-0
A$A1704[is.na(A$A1704) & A$E8033100==-3]<-0
table(A$E8013704, useNA="always")
table(A$A1704, useNA="always")
A$A1705<-NA
A$A1705[A$E8013705==0]<-0
A$A1705[A$E8013705==99]<-0
A$A1705[A$E8013705>=1 & A$E8013705<99]<-A$E8013705[A$E8013705>=1 &
A$E8013705<99]
A$A1705[is.na(A$A1705) & A$E8033000==-4]<-0
A$A1705[is.na(A$A1705) & A$E8033000==-3]<-0
A$A1705[is.na(A$A1705) & A$E8033000 >= 199300]<-0
A$A1705[is.na(A$A1705) & A$E8033100==0]<-0
A$A1705[is.na(A$A1705) & A$E8033100==-3]<-0
table(A$E8013705, useNA="always")
table(A$A1705, useNA="always")
A$A1706<-NA
A$A1706[A$E8013706==0]<-0
A$A1706[A$E8013706==99]<-0
A$A1706[A$E8013706>=1 & A$E8013706<99]<-A$E8013706[A$E8013706>=1 &
A$E8013706<99]
A$A1706[is.na(A$A1706) & A$E8033000==-4]<-0
A$A1706[is.na(A$A1706) & A$E8033000==-3]<-0
A$A1706[is.na(A$A1706) & A$E8033000 >= 199300]<-0
A$A1706[is.na(A$A1706) & A$E8033100==0]<-0
A$A1706[is.na(A$A1706) & A$E8033100==-3]<-0
table(A$E8013706, useNA="always")
table(A$A1706, useNA="always")
A$A1707<-NA
A$A1707[A$E8013707==0]<-0
A$A1707[A$E8013707==99]<-0
```

© 2023 Silver IA et al. *JAMA Network Open.*

```
A$A1707[A$E8013707>=1 & A$E8013707<99]<-A$E8013707[A$E8013707>=1 &
A$E8013707<99]
A$A1707[is.na(A$A1707) & A$E8033000==-4]<-0
A$A1707[is.na(A$A1707) & A$E8033000==-3]<-0
A$A1707[is.na(A$A1707) & A$E8033000 >= 199300]<-0
A$A1707[is.na(A$A1707) & A$E8033100==0]<-0
A$A1707[is.na(A$A1707) & A$E8033100==-3]<-0
table(A$E8013707, useNA="always")
table(A$A1707, useNA="always")
A$A1708<-NA
A$A1708[A$E8013708==0]<-0
A$A1708[A$E8013708==99]<-0
A$A1708[A$E8013708>=1 & A$E8013708<99]<-A$E8013708[A$E8013708>=1 &
A$E8013708<99]
A$A1708[is.na(A$A1708) & A$E8033000==-4]<-0
A$A1708[is.na(A$A1708) & A$E8033000==-3]<-0
A$A1708[is.na(A$A1708) & A$E8033000 >= 199300]<-0
A$A1708[is.na(A$A1708) & A$E8033100==0]<-0
A$A1708[is.na(A$A1708) & A$E8033100==-3]<-0
table(A$E8013708, useNA="always")
table(A$A1708, useNA="always")
A$A1709<-NA
A$A1709[A$E8013709==0]<-0
A$A1709[A$E8013709==99]<-0
A$A1709[A$E8013709>=1 & A$E8013709<99]<-A$E8013709[A$E8013709>=1 &
A$E8013709<99]
A$A1709[is.na(A$A1709) & A$E8033000==-4]<-0
A$A1709[is.na(A$A1709) & A$E8033000==-3]<-0
A$A1709[is.na(A$A1709) & A$E8033000 >= 199300]<-0
A$A1709[is.na(A$A1709) & A$E8033100==0]<-0
A$A1709[is.na(A$A1709) & A$E8033100==-3]<-0
table(A$E8013709, useNA="always")
table(A$A1709, useNA="always")
A$A1710<-NA
A$A1710[A$E8013710==0]<-0
A$A1710[A$E8013710==99]<-0
A$A1710[A$E8013710>=1 & A$E8013710<99]<-A$E8013710[A$E8013710>=1 &
A$E8013710<99]
A$A1710[is.na(A$A1710) & A$E8033000==-4]<-0
A$A1710[is.na(A$A1710) & A$E8033000==-3]<-0
A$A1710[is.na(A$A1710) & A$E8033000 >= 199300]<-0
A$A1710[is.na(A$A1710) & A$E8033100==0]<-0
A$A1710[is.na(A$A1710) & A$E8033100==-3]<-0
table(A$E8013710, useNA="always")
table(A$A1710, useNA="always")
A$A1711<-NA
A$A1711[A$E8013711==0]<-0
A$A1711[A$E8013711==99]<-0
A$A1711[A$E8013711>=1 & A$E8013711<99]<-A$E8013711[A$E8013711>=1 &
A$E8013711<99]
A$A1711[is.na(A$A1711) & A$E8033000==-4]<-0
A$A1711[is.na(A$A1711) & A$E8033000==-3]<-0
A$A1711[is.na(A$A1711) & A$E8033000 >= 199300]<-0
A$A1711[is.na(A$A1711) & A$E8033100==0]<-0
A$A1711[is.na(A$A1711) & A$E8033100==-3]<-0
table(A$E8013711, useNA="always")
```

© 2023 Silver IA et al. *JAMA Network Open.*

```
table(A$A1711, useNA="always")
A$A1712<-NA
A$A1712[A$E8013712==0]<-0
A$A1712[A$E8013712==99]<-0
A$A1712[A$E8013712>=1 & A$E8013712<99]<-A$E8013712[A$E8013712>=1 &
A$E8013712<99]
A$A1712[is.na(A$A1712) & A$E8033000==-4]<-0
A$A1712[is.na(A$A1712) & A$E8033000==-3]<-0
A$A1712[is.na(A$A1712) & A$E8033000 >= 199300]<-0
A$A1712[is.na(A$A1712) & A$E8033100==0]<-0
A$A1712[is.na(A$A1712) & A$E8033100==-3]<-0
table(A$E8013712, useNA="always")
table(A$A1712, useNA="always")




A$A17<-
rowSums(data.frame(A$A1701,A$A1702,A$A1703,A$A1704,A$A1705,A$A1706,A$A1707,A$
A1708,A$A1709,A$A1710,A$A1711,A$A1712), na.rm = T)
table(A$A17)



## Incarceration measures

table(A$E8043000)
table(A$E8043100)
table(A$E8043500)

names(A)

### Incarceration (1992)

table(A$E8021201, useNA="always")
table(A$E8021202)
table(A$E8021203)
table(A$E8021204)
table(A$E8021205)
table(A$E8021206)
table(A$E8021207)
table(A$E8021208)
table(A$E8021209)
table(A$E8021210)
table(A$E8021211)
table(A$E8021212)

A$I9201<-NA
A$I9201[A$E8021201==0]<-0
A$I9201[A$E8021201==99]<-0
A$I9201[A$E8021201==1]<-1
A$I9201[is.na(A$I9201) & A$E8043000==-4]<-0
A$I9201[is.na(A$I9201) & A$E8043000==-3]<-0
A$I9201[is.na(A$I9201) & A$E8043000 >= 199300]<-0
A$I9201[is.na(A$I9201) & A$E8043500==0]<-0
A$I9201[is.na(A$I9201) & A$E8043500==-3]<-0
```

© 2023 Silver IA et al. *JAMA Network Open.*

```
table(A$E8021201, useNA="always")
table(A$I9201, useNA="always")
A$I9202<-NA
A$I9202[A$E8021202==0]<-0
A$I9202[A$E8021202==99]<-0
A$I9202[A$E8021202==1]<-1
A$I9202[is.na(A$I9202) & A$E8043000==-4]<-0
A$I9202[is.na(A$I9202) & A$E8043000==-3]<-0
A$I9202[is.na(A$I9202) & A$E8043000 >= 199300]<-0
A$I9202[is.na(A$I9202) & A$E8043500==0]<-0
A$I9202[is.na(A$I9202) & A$E8043500==-3]<-0
table(A$E8021202, useNA="always")
table(A$I9202, useNA="always")
A$I9203<-NA
A$I9203[A$E8021203==0]<-0
A$I9203[A$E8021203==99]<-0
A$I9203[A$E8021203==1]<-1
A$I9203[is.na(A$I9203) & A$E8043000==-4]<-0
A$I9203[is.na(A$I9203) & A$E8043000==-3]<-0
A$I9203[is.na(A$I9203) & A$E8043000 >= 199300]<-0
A$I9203[is.na(A$I9203) & A$E8043500==0]<-0
A$I9203[is.na(A$I9203) & A$E8043500==-3]<-0
table(A$E8021203, useNA="always")
table(A$I9203, useNA="always")
A$I9204<-NA
A$I9204[A$E8021204==0]<-0
A$I9204[A$E8021204==99]<-0
A$I9204[A$E8021204==1]<-1
A$I9204[is.na(A$I9204) & A$E8043000==-4]<-0
A$I9204[is.na(A$I9204) & A$E8043000==-3]<-0
A$I9204[is.na(A$I9204) & A$E8043000 >= 199300]<-0
A$I9204[is.na(A$I9204) & A$E8043500==0]<-0
A$I9204[is.na(A$I9204) & A$E8043500==-3]<-0
table(A$E8021204, useNA="always")
table(A$I9204, useNA="always")
A$I9205<-NA
A$I9205[A$E8021205==0]<-0
A$I9205[A$E8021205==99]<-0
A$I9205[A$E8021205==1]<-1
A$I9205[is.na(A$I9205) & A$E8043000==-4]<-0
A$I9205[is.na(A$I9205) & A$E8043000==-3]<-0
A$I9205[is.na(A$I9205) & A$E8043000 >= 199300]<-0
A$I9205[is.na(A$I9205) & A$E8043500==0]<-0
A$I9205[is.na(A$I9205) & A$E8043500==-3]<-0
table(A$E8021205, useNA="always")
table(A$I9205, useNA="always")
A$I9206<-NA
A$I9206[A$E8021206==0]<-0
A$I9206[A$E8021206==99]<-0
A$I9206[A$E8021206==1]<-1
A$I9206[is.na(A$I9206) & A$E8043000==-4]<-0
A$I9206[is.na(A$I9206) & A$E8043000==-3]<-0
A$I9206[is.na(A$I9206) & A$E8043000 >= 199300]<-0
A$I9206[is.na(A$I9206) & A$E8043500==0]<-0
A$I9206[is.na(A$I9206) & A$E8043500==-3]<-0
table(A$E8021206, useNA="always")
```

© 2023 Silver IA et al. *JAMA Network Open.*

```
table(A$I9206, useNA="always")
A$I9207<-NA
A$I9207[A$E8021207==0]<-0
A$I9207[A$E8021207==99]<-0
A$I9207[A$E8021207==1]<-1
A$I9207[is.na(A$I9207) & A$E8043000==-4]<-0
A$I9207[is.na(A$I9207) & A$E8043000==-3]<-0
A$I9207[is.na(A$I9207) & A$E8043000 >= 199300]<-0
A$I9207[is.na(A$I9207) & A$E8043500==0]<-0
A$I9207[is.na(A$I9207) & A$E8043500==-3]<-0
table(A$E8021207, useNA="always")
table(A$I9207, useNA="always")
A$I9208<-NA
A$I9208[A$E8021208==0]<-0
A$I9208[A$E8021208==99]<-0
A$I9208[A$E8021208==1]<-1
A$I9208[is.na(A$I9208) & A$E8043000==-4]<-0
A$I9208[is.na(A$I9208) & A$E8043000==-3]<-0
A$I9208[is.na(A$I9208) & A$E8043000 >= 199300]<-0
A$I9208[is.na(A$I9208) & A$E8043500==0]<-0
A$I9208[is.na(A$I9208) & A$E8043500==-3]<-0
table(A$E8021208, useNA="always")
table(A$I9208, useNA="always")
A$I9209<-NA
A$I9209[A$E8021209==0]<-0
A$I9209[A$E8021209==99]<-0
A$I9209[A$E8021209==1]<-1
A$I9209[is.na(A$I9209) & A$E8043000==-4]<-0
A$I9209[is.na(A$I9209) & A$E8043000==-3]<-0
A$I9209[is.na(A$I9209) & A$E8043000 >= 199300]<-0
A$I9209[is.na(A$I9209) & A$E8043500==0]<-0
A$I9209[is.na(A$I9209) & A$E8043500==-3]<-0
table(A$E8021209, useNA="always")
table(A$I9209, useNA="always")
A$I9210<-NA
A$I9210[A$E8021210==0]<-0
A$I9210[A$E8021210==99]<-0
A$I9210[A$E8021210==1]<-1
A$I9210[is.na(A$I9210) & A$E8043000==-4]<-0
A$I9210[is.na(A$I9210) & A$E8043000==-3]<-0
A$I9210[is.na(A$I9210) & A$E8043000 >= 199300]<-0
A$I9210[is.na(A$I9210) & A$E8043500==0]<-0
A$I9210[is.na(A$I9210) & A$E8043500==-3]<-0
table(A$E8021210, useNA="always")
table(A$I9210, useNA="always")
A$I9211<-NA
A$I9211[A$E8021211==0]<-0
A$I9211[A$E8021211==99]<-0
A$I9211[A$E8021211==1]<-1
A$I9211[is.na(A$I9211) & A$E8043000==-4]<-0
A$I9211[is.na(A$I9211) & A$E8043000==-3]<-0
A$I9211[is.na(A$I9211) & A$E8043000 >= 199300]<-0
A$I9211[is.na(A$I9211) & A$E8043500==0]<-0
A$I9211[is.na(A$I9211) & A$E8043500==-3]<-0
table(A$E8021211, useNA="always")
table(A$I9211, useNA="always")
```

© 2023 Silver IA et al. *JAMA Network Open.*

```
A$I9212<-NA
A$I9212[A$E8021212==0]<-0
A$I9212[A$E8021212==99]<-0
A$I9212[A$E8021212==1]<-1
A$I9212[is.na(A$I9212) & A$E8043000==-4]<-0
A$I9212[is.na(A$I9212) & A$E8043000==-3]<-0
A$I9212[is.na(A$I9212) & A$E8043000 >= 199300]<-0
A$I9212[is.na(A$I9212) & A$E8043500==0]<-0
A$I9212[is.na(A$I9212) & A$E8043500==-3]<-0
table(A$E8021212, useNA="always")
table(A$I9212, useNA="always")




A$I92<-
rowSums(data.frame(A$I9201,A$I9202,A$I9203,A$I9204,A$I9205,A$I9206,A$I9207,A$
I9208,A$I9209,A$I9210,A$I9211,A$I9212), na.rm = T)
table(A$I92)



### Incarceration (1993)

table(A$E8021301, useNA="always")
table(A$E8021302)
table(A$E8021303)
table(A$E8021304)
table(A$E8021305)
table(A$E8021306)
table(A$E8021307)
table(A$E8021308)
table(A$E8021309)
table(A$E8021310)
table(A$E8021311)
table(A$E8021312)

A$I9301<-NA
A$I9301[A$E8021301==0]<-0
A$I9301[A$E8021301==99]<-0
A$I9301[A$E8021301==1]<-1
A$I9301[is.na(A$I9301) & A$E8043000==-4]<-0
A$I9301[is.na(A$I9301) & A$E8043000==-3]<-0
A$I9301[is.na(A$I9301) & A$E8043000 >= 199400]<-0
A$I9301[is.na(A$I9301) & A$E8043500==0]<-0
A$I9301[is.na(A$I9301) & A$E8043500==-3]<-0
table(A$E8021301, useNA="always")
table(A$I9301, useNA="always")
A$I9302<-NA
A$I9302[A$E8021302==0]<-0
A$I9302[A$E8021302==99]<-0
A$I9302[A$E8021302==1]<-1
A$I9302[is.na(A$I9302) & A$E8043000==-4]<-0
A$I9302[is.na(A$I9302) & A$E8043000==-3]<-0
A$I9302[is.na(A$I9302) & A$E8043000 >= 199400]<-0
A$I9302[is.na(A$I9302) & A$E8043500==0]<-0
```

© 2023 Silver IA et al. *JAMA Network Open.*

```
A$I9302[is.na(A$I9302) & A$E8043500==-3]<-0
table(A$E8021302, useNA="always")
table(A$I9302, useNA="always")
A$I9303<-NA
A$I9303[A$E8021303==0]<-0
A$I9303[A$E8021303==99]<-0
A$I9303[A$E8021303==1]<-1
A$I9303[is.na(A$I9303) & A$E8043000==-4]<-0
A$I9303[is.na(A$I9303) & A$E8043000==-3]<-0
A$I9303[is.na(A$I9303) & A$E8043000 >= 199400]<-0
A$I9303[is.na(A$I9303) & A$E8043500==0]<-0
A$I9303[is.na(A$I9303) & A$E8043500==-3]<-0
table(A$E8021303, useNA="always")
table(A$I9303, useNA="always")
A$I9304<-NA
A$I9304[A$E8021304==0]<-0
A$I9304[A$E8021304==99]<-0
A$I9304[A$E8021304==1]<-1
A$I9304[is.na(A$I9304) & A$E8043000==-4]<-0
A$I9304[is.na(A$I9304) & A$E8043000==-3]<-0
A$I9304[is.na(A$I9304) & A$E8043000 >= 199400]<-0
A$I9304[is.na(A$I9304) & A$E8043500==0]<-0
A$I9304[is.na(A$I9304) & A$E8043500==-3]<-0
table(A$E8021304, useNA="always")
table(A$I9304, useNA="always")
A$I9305<-NA
A$I9305[A$E8021305==0]<-0
A$I9305[A$E8021305==99]<-0
A$I9305[A$E8021305==1]<-1
A$I9305[is.na(A$I9305) & A$E8043000==-4]<-0
A$I9305[is.na(A$I9305) & A$E8043000==-3]<-0
A$I9305[is.na(A$I9305) & A$E8043000 >= 199400]<-0
A$I9305[is.na(A$I9305) & A$E8043500==0]<-0
A$I9305[is.na(A$I9305) & A$E8043500==-3]<-0
table(A$E8021305, useNA="always")
table(A$I9305, useNA="always")
A$I9306<-NA
A$I9306[A$E8021306==0]<-0
A$I9306[A$E8021306==99]<-0
A$I9306[A$E8021306==1]<-1
A$I9306[is.na(A$I9306) & A$E8043000==-4]<-0
A$I9306[is.na(A$I9306) & A$E8043000==-3]<-0
A$I9306[is.na(A$I9306) & A$E8043000 >= 199400]<-0
A$I9306[is.na(A$I9306) & A$E8043500==0]<-0
A$I9306[is.na(A$I9306) & A$E8043500==-3]<-0
table(A$E8021306, useNA="always")
table(A$I9306, useNA="always")
A$I9307<-NA
A$I9307[A$E8021307==0]<-0
A$I9307[A$E8021307==99]<-0
A$I9307[A$E8021307==1]<-1
A$I9307[is.na(A$I9307) & A$E8043000==-4]<-0
A$I9307[is.na(A$I9307) & A$E8043000==-3]<-0
A$I9307[is.na(A$I9307) & A$E8043000 >= 199400]<-0
A$I9307[is.na(A$I9307) & A$E8043500==0]<-0
A$I9307[is.na(A$I9307) & A$E8043500==-3]<-0
```

© 2023 Silver IA et al. *JAMA Network Open.*

```
table(A$E8021307, useNA="always")
table(A$I9307, useNA="always")
A$I9308<-NA
A$I9308[A$E8021308==0]<-0
A$I9308[A$E8021308==99]<-0
A$I9308[A$E8021308==1]<-1
A$I9308[is.na(A$I9308) & A$E8043000==-4]<-0
A$I9308[is.na(A$I9308) & A$E8043000==-3]<-0
A$I9308[is.na(A$I9308) & A$E8043000 >= 199400]<-0
A$I9308[is.na(A$I9308) & A$E8043500==0]<-0
A$I9308[is.na(A$I9308) & A$E8043500==-3]<-0
table(A$E8021308, useNA="always")
table(A$I9308, useNA="always")
A$I9309<-NA
A$I9309[A$E8021309==0]<-0
A$I9309[A$E8021309==99]<-0
A$I9309[A$E8021309==1]<-1
A$I9309[is.na(A$I9309) & A$E8043000==-4]<-0
A$I9309[is.na(A$I9309) & A$E8043000==-3]<-0
A$I9309[is.na(A$I9309) & A$E8043000 >= 199400]<-0
A$I9309[is.na(A$I9309) & A$E8043500==0]<-0
A$I9309[is.na(A$I9309) & A$E8043500==-3]<-0
table(A$E8021309, useNA="always")
table(A$I9309, useNA="always")
A$I9310<-NA
A$I9310[A$E8021310==0]<-0
A$I9310[A$E8021310==99]<-0
A$I9310[A$E8021310==1]<-1
A$I9310[is.na(A$I9310) & A$E8043000==-4]<-0
A$I9310[is.na(A$I9310) & A$E8043000==-3]<-0
A$I9310[is.na(A$I9310) & A$E8043000 >= 199400]<-0
A$I9310[is.na(A$I9310) & A$E8043500==0]<-0
A$I9310[is.na(A$I9310) & A$E8043500==-3]<-0
table(A$E8021310, useNA="always")
table(A$I9310, useNA="always")
A$I9311<-NA
A$I9311[A$E8021311==0]<-0
A$I9311[A$E8021311==99]<-0
A$I9311[A$E8021311==1]<-1
A$I9311[is.na(A$I9311) & A$E8043000==-4]<-0
A$I9311[is.na(A$I9311) & A$E8043000==-3]<-0
A$I9311[is.na(A$I9311) & A$E8043000 >= 199400]<-0
A$I9311[is.na(A$I9311) & A$E8043500==0]<-0
A$I9311[is.na(A$I9311) & A$E8043500==-3]<-0
table(A$E8021311, useNA="always")
table(A$I9311, useNA="always")
A$I9312<-NA
A$I9312[A$E8021312==0]<-0
A$I9312[A$E8021312==99]<-0
A$I9312[A$E8021312==1]<-1
A$I9312[is.na(A$I9312) & A$E8043000==-4]<-0
A$I9312[is.na(A$I9312) & A$E8043000==-3]<-0
A$I9312[is.na(A$I9312) & A$E8043000 >= 199400]<-0
A$I9312[is.na(A$I9312) & A$E8043500==0]<-0
A$I9312[is.na(A$I9312) & A$E8043500==-3]<-0
table(A$E8021312, useNA="always")
```

© 2023 Silver IA et al. *JAMA Network Open.*

```
table(A$I9312, useNA="always")




A$I93<-
rowSums(data.frame(A$I9301,A$I9302,A$I9303,A$I9304,A$I9305,A$I9306,A$I9307,A$
I9308,A$I9309,A$I9310,A$I9311,A$I9312), na.rm = T)
table(A$I93)
### Incarceration (1994)

table(A$E8021401, useNA="always")
table(A$E8021402)
table(A$E8021403)
table(A$E8021404)
table(A$E8021405)
table(A$E8021406)
table(A$E8021407)
table(A$E8021408)
table(A$E8021409)
table(A$E8021410)
table(A$E8021411)
table(A$E8021412)

A$I9401<-NA
A$I9401[A$E8021401==0]<-0
A$I9401[A$E8021401==99]<-0
A$I9401[A$E8021401==1]<-1
A$I9401[is.na(A$I9401) & A$E8043000==-4]<-0
A$I9401[is.na(A$I9401) & A$E8043000==-3]<-0
A$I9401[is.na(A$I9401) & A$E8043000 >= 199500]<-0
A$I9401[is.na(A$I9401) & A$E8043500==0]<-0
A$I9401[is.na(A$I9401) & A$E8043500==-3]<-0
table(A$E8021401, useNA="always")
table(A$I9401, useNA="always")
A$I9402<-NA
A$I9402[A$E8021402==0]<-0
A$I9402[A$E8021402==99]<-0
A$I9402[A$E8021402==1]<-1
A$I9402[is.na(A$I9402) & A$E8043000==-4]<-0
A$I9402[is.na(A$I9402) & A$E8043000==-3]<-0
A$I9402[is.na(A$I9402) & A$E8043000 >= 199500]<-0
A$I9402[is.na(A$I9402) & A$E8043500==0]<-0
A$I9402[is.na(A$I9402) & A$E8043500==-3]<-0
table(A$E8021402, useNA="always")
table(A$I9402, useNA="always")
A$I9403<-NA
A$I9403[A$E8021403==0]<-0
A$I9403[A$E8021403==99]<-0
A$I9403[A$E8021403==1]<-1
A$I9403[is.na(A$I9403) & A$E8043000==-4]<-0
A$I9403[is.na(A$I9403) & A$E8043000==-3]<-0
A$I9403[is.na(A$I9403) & A$E8043000 >= 199500]<-0
A$I9403[is.na(A$I9403) & A$E8043500==0]<-0
A$I9403[is.na(A$I9403) & A$E8043500==-3]<-0
table(A$E8021403, useNA="always")
```

© 2023 Silver IA et al. *JAMA Network Open.*

```
table(A$I9403, useNA="always")
A$I9404<-NA
A$I9404[A$E8021404==0]<-0
A$I9404[A$E8021404==99]<-0
A$I9404[A$E8021404==1]<-1
A$I9404[is.na(A$I9404) & A$E8043000==-4]<-0
A$I9404[is.na(A$I9404) & A$E8043000==-3]<-0
A$I9404[is.na(A$I9404) & A$E8043000 >= 199500]<-0
A$I9404[is.na(A$I9404) & A$E8043500==0]<-0
A$I9404[is.na(A$I9404) & A$E8043500==-3]<-0
table(A$E8021404, useNA="always")
table(A$I9404, useNA="always")
A$I9405<-NA
A$I9405[A$E8021405==0]<-0
A$I9405[A$E8021405==99]<-0
A$I9405[A$E8021405==1]<-1
A$I9405[is.na(A$I9405) & A$E8043000==-4]<-0
A$I9405[is.na(A$I9405) & A$E8043000==-3]<-0
A$I9405[is.na(A$I9405) & A$E8043000 >= 199500]<-0
A$I9405[is.na(A$I9405) & A$E8043500==0]<-0
A$I9405[is.na(A$I9405) & A$E8043500==-3]<-0
table(A$E8021405, useNA="always")
table(A$I9405, useNA="always")
A$I9406<-NA
A$I9406[A$E8021406==0]<-0
A$I9406[A$E8021406==99]<-0
A$I9406[A$E8021406==1]<-1
A$I9406[is.na(A$I9406) & A$E8043000==-4]<-0
A$I9406[is.na(A$I9406) & A$E8043000==-3]<-0
A$I9406[is.na(A$I9406) & A$E8043000 >= 199500]<-0
A$I9406[is.na(A$I9406) & A$E8043500==0]<-0
A$I9406[is.na(A$I9406) & A$E8043500==-3]<-0
table(A$E8021406, useNA="always")
table(A$I9406, useNA="always")
A$I9407<-NA
A$I9407[A$E8021407==0]<-0
A$I9407[A$E8021407==99]<-0
A$I9407[A$E8021407==1]<-1
A$I9407[is.na(A$I9407) & A$E8043000==-4]<-0
A$I9407[is.na(A$I9407) & A$E8043000==-3]<-0
A$I9407[is.na(A$I9407) & A$E8043000 >= 199500]<-0
A$I9407[is.na(A$I9407) & A$E8043500==0]<-0
A$I9407[is.na(A$I9407) & A$E8043500==-3]<-0
table(A$E8021407, useNA="always")
table(A$I9407, useNA="always")
A$I9408<-NA
A$I9408[A$E8021408==0]<-0
A$I9408[A$E8021408==99]<-0
A$I9408[A$E8021408==1]<-1
A$I9408[is.na(A$I9408) & A$E8043000==-4]<-0
A$I9408[is.na(A$I9408) & A$E8043000==-3]<-0
A$I9408[is.na(A$I9408) & A$E8043000 >= 199500]<-0
A$I9408[is.na(A$I9408) & A$E8043500==0]<-0
A$I9408[is.na(A$I9408) & A$E8043500==-3]<-0
table(A$E8021408, useNA="always")
table(A$I9408, useNA="always")
```

```
A$I9409<-NA
A$I9409[A$E8021409==0]<-0
A$I9409[A$E8021409==99]<-0
A$I9409[A$E8021409==1]<-1
A$I9409[is.na(A$I9409) & A$E8043000==-4]<-0
A$I9409[is.na(A$I9409) & A$E8043000==-3]<-0
A$I9409[is.na(A$I9409) & A$E8043000 >= 199500]<-0
A$I9409[is.na(A$I9409) & A$E8043500==0]<-0
A$I9409[is.na(A$I9409) & A$E8043500==-3]<-0
table(A$E8021409, useNA="always")
table(A$I9409, useNA="always")
A$I9410<-NA
A$I9410[A$E8021410==0]<-0
A$I9410[A$E8021410==99]<-0
A$I9410[A$E8021410==1]<-1
A$I9410[is.na(A$I9410) & A$E8043000==-4]<-0
A$I9410[is.na(A$I9410) & A$E8043000==-3]<-0
A$I9410[is.na(A$I9410) & A$E8043000 >= 199500]<-0
A$I9410[is.na(A$I9410) & A$E8043500==0]<-0
A$I9410[is.na(A$I9410) & A$E8043500==-3]<-0
table(A$E8021410, useNA="always")
table(A$I9410, useNA="always")
A$I9411<-NA
A$I9411[A$E8021411==0]<-0
A$I9411[A$E8021411==99]<-0
A$I9411[A$E8021411==1]<-1
A$I9411[is.na(A$I9411) & A$E8043000==-4]<-0
A$I9411[is.na(A$I9411) & A$E8043000==-3]<-0
A$I9411[is.na(A$I9411) & A$E8043000 >= 199500]<-0
A$I9411[is.na(A$I9411) & A$E8043500==0]<-0
A$I9411[is.na(A$I9411) & A$E8043500==-3]<-0
table(A$E8021411, useNA="always")
table(A$I9411, useNA="always")
A$I9412<-NA
A$I9412[A$E8021412==0]<-0
A$I9412[A$E8021412==99]<-0
A$I9412[A$E8021412==1]<-1
A$I9412[is.na(A$I9412) & A$E8043000==-4]<-0
A$I9412[is.na(A$I9412) & A$E8043000==-3]<-0
A$I9412[is.na(A$I9412) & A$E8043000 >= 199500]<-0
A$I9412[is.na(A$I9412) & A$E8043500==0]<-0
A$I9412[is.na(A$I9412) & A$E8043500==-3]<-0
table(A$E8021412, useNA="always")
table(A$I9412, useNA="always")




A$I94<-
rowSums(data.frame(A$I9401,A$I9402,A$I9403,A$I9404,A$I9405,A$I9406,A$I9407,A$
I9408,A$I9409,A$I9410,A$I9411,A$I9412), na.rm = T)
table(A$I94)
### Incarceration (1995)

table(A$E8021501, useNA="always")
table(A$E8021502)
```

© 2023 Silver IA et al. *JAMA Network Open.*

```
table(A$E8021503)
table(A$E8021504)
table(A$E8021505)
table(A$E8021506)
table(A$E8021507)
table(A$E8021508)
table(A$E8021509)
table(A$E8021510)
table(A$E8021511)
table(A$E8021512)

A$I9501<-NA
A$I9501[A$E8021501==0]<-0
A$I9501[A$E8021501==99]<-0
A$I9501[A$E8021501==1]<-1
A$I9501[is.na(A$I9501) & A$E8043000==-4]<-0
A$I9501[is.na(A$I9501) & A$E8043000==-3]<-0
A$I9501[is.na(A$I9501) & A$E8043000 >= 199600]<-0
A$I9501[is.na(A$I9501) & A$E8043500==0]<-0
A$I9501[is.na(A$I9501) & A$E8043500==-3]<-0
table(A$E8021501, useNA="always")
table(A$I9501, useNA="always")
A$I9502<-NA
A$I9502[A$E8021502==0]<-0
A$I9502[A$E8021502==99]<-0
A$I9502[A$E8021502==1]<-1
A$I9502[is.na(A$I9502) & A$E8043000==-4]<-0
A$I9502[is.na(A$I9502) & A$E8043000==-3]<-0
A$I9502[is.na(A$I9502) & A$E8043000 >= 199600]<-0
A$I9502[is.na(A$I9502) & A$E8043500==0]<-0
A$I9502[is.na(A$I9502) & A$E8043500==-3]<-0
table(A$E8021502, useNA="always")
table(A$I9502, useNA="always")
A$I9503<-NA
A$I9503[A$E8021503==0]<-0
A$I9503[A$E8021503==99]<-0
A$I9503[A$E8021503==1]<-1
A$I9503[is.na(A$I9503) & A$E8043000==-4]<-0
A$I9503[is.na(A$I9503) & A$E8043000==-3]<-0
A$I9503[is.na(A$I9503) & A$E8043000 >= 199600]<-0
A$I9503[is.na(A$I9503) & A$E8043500==0]<-0
A$I9503[is.na(A$I9503) & A$E8043500==-3]<-0
table(A$E8021503, useNA="always")
table(A$I9503, useNA="always")
A$I9504<-NA
A$I9504[A$E8021504==0]<-0
A$I9504[A$E8021504==99]<-0
A$I9504[A$E8021504==1]<-1
A$I9504[is.na(A$I9504) & A$E8043000==-4]<-0
A$I9504[is.na(A$I9504) & A$E8043000==-3]<-0
A$I9504[is.na(A$I9504) & A$E8043000 >= 199600]<-0
A$I9504[is.na(A$I9504) & A$E8043500==0]<-0
A$I9504[is.na(A$I9504) & A$E8043500==-3]<-0
table(A$E8021504, useNA="always")
table(A$I9504, useNA="always")
A$I9505<-NA
```

```
A$I9505[A$E8021505==0]<-0
A$I9505[A$E8021505==99]<-0
A$I9505[A$E8021505==1]<-1
A$I9505[is.na(A$I9505) & A$E8043000==-4]<-0
A$I9505[is.na(A$I9505) & A$E8043000==-3]<-0
A$I9505[is.na(A$I9505) & A$E8043000 >= 199600]<-0
A$I9505[is.na(A$I9505) & A$E8043500==0]<-0
A$I9505[is.na(A$I9505) & A$E8043500==-3]<-0
table(A$E8021505, useNA="always")
table(A$I9505, useNA="always")
A$I9506<-NA
A$I9506[A$E8021506==0]<-0
A$I9506[A$E8021506==99]<-0
A$I9506[A$E8021506==1]<-1
A$I9506[is.na(A$I9506) & A$E8043000==-4]<-0
A$I9506[is.na(A$I9506) & A$E8043000==-3]<-0
A$I9506[is.na(A$I9506) & A$E8043000 >= 199600]<-0
A$I9506[is.na(A$I9506) & A$E8043500==0]<-0
A$I9506[is.na(A$I9506) & A$E8043500==-3]<-0
table(A$E8021506, useNA="always")
table(A$I9506, useNA="always")
A$I9507<-NA
A$I9507[A$E8021507==0]<-0
A$I9507[A$E8021507==99]<-0
A$I9507[A$E8021507==1]<-1
A$I9507[is.na(A$I9507) & A$E8043000==-4]<-0
A$I9507[is.na(A$I9507) & A$E8043000==-3]<-0
A$I9507[is.na(A$I9507) & A$E8043000 >= 199600]<-0
A$I9507[is.na(A$I9507) & A$E8043500==0]<-0
A$I9507[is.na(A$I9507) & A$E8043500==-3]<-0
table(A$E8021507, useNA="always")
table(A$I9507, useNA="always")
A$I9508<-NA
A$I9508[A$E8021508==0]<-0
A$I9508[A$E8021508==99]<-0
A$I9508[A$E8021508==1]<-1
A$I9508[is.na(A$I9508) & A$E8043000==-4]<-0
A$I9508[is.na(A$I9508) & A$E8043000==-3]<-0
A$I9508[is.na(A$I9508) & A$E8043000 >= 199600]<-0
A$I9508[is.na(A$I9508) & A$E8043500==0]<-0
A$I9508[is.na(A$I9508) & A$E8043500==-3]<-0
table(A$E8021508, useNA="always")
table(A$I9508, useNA="always")
A$I9509<-NA
A$I9509[A$E8021509==0]<-0
A$I9509[A$E8021509==99]<-0
A$I9509[A$E8021509==1]<-1
A$I9509[is.na(A$I9509) & A$E8043000==-4]<-0
A$I9509[is.na(A$I9509) & A$E8043000==-3]<-0
A$I9509[is.na(A$I9509) & A$E8043000 >= 199600]<-0
A$I9509[is.na(A$I9509) & A$E8043500==0]<-0
A$I9509[is.na(A$I9509) & A$E8043500==-3]<-0
table(A$E8021509, useNA="always")
table(A$I9509, useNA="always")
A$I9510<-NA
A$I9510[A$E8021510==0]<-0
```

© 2023 Silver IA et al. *JAMA Network Open.*

```
A$I9510[A$E8021510==99]<-0
A$I9510[A$E8021510==1]<-1
A$I9510[is.na(A$I9510) & A$E8043000==-4]<-0
A$I9510[is.na(A$I9510) & A$E8043000==-3]<-0
A$I9510[is.na(A$I9510) & A$E8043000 >= 199600]<-0
A$I9510[is.na(A$I9510) & A$E8043500==0]<-0
A$I9510[is.na(A$I9510) & A$E8043500==-3]<-0
table(A$E8021510, useNA="always")
table(A$I9510, useNA="always")
A$I9511<-NA
A$I9511[A$E8021511==0]<-0
A$I9511[A$E8021511==99]<-0
A$I9511[A$E8021511==1]<-1
A$I9511[is.na(A$I9511) & A$E8043000==-4]<-0
A$I9511[is.na(A$I9511) & A$E8043000==-3]<-0
A$I9511[is.na(A$I9511) & A$E8043000 >= 199600]<-0
A$I9511[is.na(A$I9511) & A$E8043500==0]<-0
A$I9511[is.na(A$I9511) & A$E8043500==-3]<-0
table(A$E8021511, useNA="always")
table(A$I9511, useNA="always")
A$I9512<-NA
A$I9512[A$E8021512==0]<-0
A$I9512[A$E8021512==99]<-0
A$I9512[A$E8021512==1]<-1
A$I9512[is.na(A$I9512) & A$E8043000==-4]<-0
A$I9512[is.na(A$I9512) & A$E8043000==-3]<-0
A$I9512[is.na(A$I9512) & A$E8043000 >= 199600]<-0
A$I9512[is.na(A$I9512) & A$E8043500==0]<-0
A$I9512[is.na(A$I9512) & A$E8043500==-3]<-0
table(A$E8021512, useNA="always")
table(A$I9512, useNA="always")



A$I95<-
rowSums(data.frame(A$I9501,A$I9502,A$I9503,A$I9504,A$I9505,A$I9506,A$I9507,A$
I9508,A$I9509,A$I9510,A$I9511,A$I9512), na.rm = T)
table(A$I95)
### Incarceration (1996)

table(A$E8021601, useNA="always")
table(A$E8021602)
table(A$E8021603)
table(A$E8021604)
table(A$E8021605)
table(A$E8021606)
table(A$E8021607)
table(A$E8021608)
table(A$E8021609)
table(A$E8021610)
table(A$E8021611)
table(A$E8021612)

A$I9601<-NA
A$I9601[A$E8021601==0]<-0
```

© 2023 Silver IA et al. *JAMA Network Open.*

```
A$I9601[A$E8021601==99]<-0
A$I9601[A$E8021601==1]<-1
A$I9601[is.na(A$I9601) & A$E8043000==-4]<-0
A$I9601[is.na(A$I9601) & A$E8043000==-3]<-0
A$I9601[is.na(A$I9601) & A$E8043000 >= 199700]<-0
A$I9601[is.na(A$I9601) & A$E8043500==0]<-0
A$I9601[is.na(A$I9601) & A$E8043500==-3]<-0
table(A$E8021601, useNA="always")
table(A$I9601, useNA="always")
A$I9602<-NA
A$I9602[A$E8021602==0]<-0
A$I9602[A$E8021602==99]<-0
A$I9602[A$E8021602==1]<-1
A$I9602[is.na(A$I9602) & A$E8043000==-4]<-0
A$I9602[is.na(A$I9602) & A$E8043000==-3]<-0
A$I9602[is.na(A$I9602) & A$E8043000 >= 199700]<-0
A$I9602[is.na(A$I9602) & A$E8043500==0]<-0
A$I9602[is.na(A$I9602) & A$E8043500==-3]<-0
table(A$E8021602, useNA="always")
table(A$I9602, useNA="always")
A$I9603<-NA
A$I9603[A$E8021603==0]<-0
A$I9603[A$E8021603==99]<-0
A$I9603[A$E8021603==1]<-1
A$I9603[is.na(A$I9603) & A$E8043000==-4]<-0
A$I9603[is.na(A$I9603) & A$E8043000==-3]<-0
A$I9603[is.na(A$I9603) & A$E8043000 >= 199700]<-0
A$I9603[is.na(A$I9603) & A$E8043500==0]<-0
A$I9603[is.na(A$I9603) & A$E8043500==-3]<-0
table(A$E8021603, useNA="always")
table(A$I9603, useNA="always")
A$I9604<-NA
A$I9604[A$E8021604==0]<-0
A$I9604[A$E8021604==99]<-0
A$I9604[A$E8021604==1]<-1
A$I9604[is.na(A$I9604) & A$E8043000==-4]<-0
A$I9604[is.na(A$I9604) & A$E8043000==-3]<-0
A$I9604[is.na(A$I9604) & A$E8043000 >= 199700]<-0
A$I9604[is.na(A$I9604) & A$E8043500==0]<-0
A$I9604[is.na(A$I9604) & A$E8043500==-3]<-0
table(A$E8021604, useNA="always")
table(A$I9604, useNA="always")
A$I9605<-NA
A$I9605[A$E8021605==0]<-0
A$I9605[A$E8021605==99]<-0
A$I9605[A$E8021605==1]<-1
A$I9605[is.na(A$I9605) & A$E8043000==-4]<-0
A$I9605[is.na(A$I9605) & A$E8043000==-3]<-0
A$I9605[is.na(A$I9605) & A$E8043000 >= 199700]<-0
A$I9605[is.na(A$I9605) & A$E8043500==0]<-0
A$I9605[is.na(A$I9605) & A$E8043500==-3]<-0
table(A$E8021605, useNA="always")
table(A$I9605, useNA="always")
A$I9606<-NA
A$I9606[A$E8021606==0]<-0
A$I9606[A$E8021606==99]<-0
```

© 2023 Silver IA et al. *JAMA Network Open.*

```
A$I9606[A$E8021606==1]<-1
A$I9606[is.na(A$I9606) & A$E8043000==-4]<-0
A$I9606[is.na(A$I9606) & A$E8043000==-3]<-0
A$I9606[is.na(A$I9606) & A$E8043000 >= 199700]<-0
A$I9606[is.na(A$I9606) & A$E8043500==0]<-0
A$I9606[is.na(A$I9606) & A$E8043500==-3]<-0
table(A$E8021606, useNA="always")
table(A$I9606, useNA="always")
A$I9607<-NA
A$I9607[A$E8021607==0]<-0
A$I9607[A$E8021607==99]<-0
A$I9607[A$E8021607==1]<-1
A$I9607[is.na(A$I9607) & A$E8043000==-4]<-0
A$I9607[is.na(A$I9607) & A$E8043000==-3]<-0
A$I9607[is.na(A$I9607) & A$E8043000 >= 199700]<-0
A$I9607[is.na(A$I9607) & A$E8043500==0]<-0
A$I9607[is.na(A$I9607) & A$E8043500==-3]<-0
table(A$E8021607, useNA="always")
table(A$I9607, useNA="always")
A$I9608<-NA
A$I9608[A$E8021608==0]<-0
A$I9608[A$E8021608==99]<-0
A$I9608[A$E8021608==1]<-1
A$I9608[is.na(A$I9608) & A$E8043000==-4]<-0
A$I9608[is.na(A$I9608) & A$E8043000==-3]<-0
A$I9608[is.na(A$I9608) & A$E8043000 >= 199700]<-0
A$I9608[is.na(A$I9608) & A$E8043500==0]<-0
A$I9608[is.na(A$I9608) & A$E8043500==-3]<-0
table(A$E8021608, useNA="always")
table(A$I9608, useNA="always")
A$I9609<-NA
A$I9609[A$E8021609==0]<-0
A$I9609[A$E8021609==99]<-0
A$I9609[A$E8021609==1]<-1
A$I9609[is.na(A$I9609) & A$E8043000==-4]<-0
A$I9609[is.na(A$I9609) & A$E8043000==-3]<-0
A$I9609[is.na(A$I9609) & A$E8043000 >= 199700]<-0
A$I9609[is.na(A$I9609) & A$E8043500==0]<-0
A$I9609[is.na(A$I9609) & A$E8043500==-3]<-0
table(A$E8021609, useNA="always")
table(A$I9609, useNA="always")
A$I9610<-NA
A$I9610[A$E8021610==0]<-0
A$I9610[A$E8021610==99]<-0
A$I9610[A$E8021610==1]<-1
A$I9610[is.na(A$I9610) & A$E8043000==-4]<-0
A$I9610[is.na(A$I9610) & A$E8043000==-3]<-0
A$I9610[is.na(A$I9610) & A$E8043000 >= 199700]<-0
A$I9610[is.na(A$I9610) & A$E8043500==0]<-0
A$I9610[is.na(A$I9610) & A$E8043500==-3]<-0
table(A$E8021610, useNA="always")
table(A$I9610, useNA="always")
A$I9611<-NA
A$I9611[A$E8021611==0]<-0
A$I9611[A$E8021611==99]<-0
A$I9611[A$E8021611==1]<-1
```

```
A$I9611[is.na(A$I9611) & A$E8043000==-4]<-0
A$I9611[is.na(A$I9611) & A$E8043000==-3]<-0
A$I9611[is.na(A$I9611) & A$E8043000 >= 199700]<-0
A$I9611[is.na(A$I9611) & A$E8043500==0]<-0
A$I9611[is.na(A$I9611) & A$E8043500==-3]<-0
table(A$E8021611, useNA="always")
table(A$I9611, useNA="always")
A$I9612<-NA
A$I9612[A$E8021612==0]<-0
A$I9612[A$E8021612==99]<-0
A$I9612[A$E8021612==1]<-1
A$I9612[is.na(A$I9612) & A$E8043000==-4]<-0
A$I9612[is.na(A$I9612) & A$E8043000==-3]<-0
A$I9612[is.na(A$I9612) & A$E8043000 >= 199700]<-0
A$I9612[is.na(A$I9612) & A$E8043500==0]<-0
A$I9612[is.na(A$I9612) & A$E8043500==-3]<-0
table(A$E8021612, useNA="always")
table(A$I9612, useNA="always")




A$I96<-
rowSums(data.frame(A$I9601,A$I9602,A$I9603,A$I9604,A$I9605,A$I9606,A$I9607,A$
I9608,A$I9609,A$I9610,A$I9611,A$I9612), na.rm = T)
table(A$I96)
### Incarceration (1997)

table(A$E8021701, useNA="always")
table(A$E8021702)
table(A$E8021703)
table(A$E8021704)
table(A$E8021705)
table(A$E8021706)
table(A$E8021707)
table(A$E8021708)
table(A$E8021709)
table(A$E8021710)
table(A$E8021711)
table(A$E8021712)

A$I9701<-NA
A$I9701[A$E8021701==0]<-0
A$I9701[A$E8021701==99]<-0
A$I9701[A$E8021701==1]<-1
A$I9701[is.na(A$I9701) & A$E8043000==-4]<-0
A$I9701[is.na(A$I9701) & A$E8043000==-3]<-0
A$I9701[is.na(A$I9701) & A$E8043000 >= 199800]<-0
A$I9701[is.na(A$I9701) & A$E8043500==0]<-0
A$I9701[is.na(A$I9701) & A$E8043500==-3]<-0
table(A$E8021701, useNA="always")
table(A$I9701, useNA="always")
A$I9702<-NA
A$I9702[A$E8021702==0]<-0
A$I9702[A$E8021702==99]<-0
A$I9702[A$E8021702==1]<-1
```

```
A$I9702[is.na(A$I9702) & A$E8043000==-4]<-0
A$I9702[is.na(A$I9702) & A$E8043000==-3]<-0
A$I9702[is.na(A$I9702) & A$E8043000 >= 199800]<-0
A$I9702[is.na(A$I9702) & A$E8043500==0]<-0
A$I9702[is.na(A$I9702) & A$E8043500==-3]<-0
table(A$E8021702, useNA="always")
table(A$I9702, useNA="always")
A$I9703<-NA
A$I9703[A$E8021703==0]<-0
A$I9703[A$E8021703==99]<-0
A$I9703[A$E8021703==1]<-1
A$I9703[is.na(A$I9703) & A$E8043000==-4]<-0
A$I9703[is.na(A$I9703) & A$E8043000==-3]<-0
A$I9703[is.na(A$I9703) & A$E8043000 >= 199800]<-0
A$I9703[is.na(A$I9703) & A$E8043500==0]<-0
A$I9703[is.na(A$I9703) & A$E8043500==-3]<-0
table(A$E8021703, useNA="always")
table(A$I9703, useNA="always")
A$I9704<-NA
A$I9704[A$E8021704==0]<-0
A$I9704[A$E8021704==99]<-0
A$I9704[A$E8021704==1]<-1
A$I9704[is.na(A$I9704) & A$E8043000==-4]<-0
A$I9704[is.na(A$I9704) & A$E8043000==-3]<-0
A$I9704[is.na(A$I9704) & A$E8043000 >= 199800]<-0
A$I9704[is.na(A$I9704) & A$E8043500==0]<-0
A$I9704[is.na(A$I9704) & A$E8043500==-3]<-0
table(A$E8021704, useNA="always")
table(A$I9704, useNA="always")
A$I9705<-NA
A$I9705[A$E8021705==0]<-0
A$I9705[A$E8021705==99]<-0
A$I9705[A$E8021705==1]<-1
A$I9705[is.na(A$I9705) & A$E8043000==-4]<-0
A$I9705[is.na(A$I9705) & A$E8043000==-3]<-0
A$I9705[is.na(A$I9705) & A$E8043000 >= 199800]<-0
A$I9705[is.na(A$I9705) & A$E8043500==0]<-0
A$I9705[is.na(A$I9705) & A$E8043500==-3]<-0
table(A$E8021705, useNA="always")
table(A$I9705, useNA="always")
A$I9706<-NA
A$I9706[A$E8021706==0]<-0
A$I9706[A$E8021706==99]<-0
A$I9706[A$E8021706==1]<-1
A$I9706[is.na(A$I9706) & A$E8043000==-4]<-0
A$I9706[is.na(A$I9706) & A$E8043000==-3]<-0
A$I9706[is.na(A$I9706) & A$E8043000 >= 199800]<-0
A$I9706[is.na(A$I9706) & A$E8043500==0]<-0
A$I9706[is.na(A$I9706) & A$E8043500==-3]<-0
table(A$E8021706, useNA="always")
table(A$I9706, useNA="always")
A$I9707<-NA
A$I9707[A$E8021707==0]<-0
A$I9707[A$E8021707==99]<-0
A$I9707[A$E8021707==1]<-1
A$I9707[is.na(A$I9707) & A$E8043000==-4]<-0
```

© 2023 Silver IA et al. *JAMA Network Open.*

```
A$I9707[is.na(A$I9707) & A$E8043000==-3]<-0
A$I9707[is.na(A$I9707) & A$E8043000 >= 199800]<-0
A$I9707[is.na(A$I9707) & A$E8043500==0]<-0
A$I9707[is.na(A$I9707) & A$E8043500==-3]<-0
table(A$E8021707, useNA="always")
table(A$I9707, useNA="always")
A$I9708<-NA
A$I9708[A$E8021708==0]<-0
A$I9708[A$E8021708==99]<-0
A$I9708[A$E8021708==1]<-1
A$I9708[is.na(A$I9708) & A$E8043000==-4]<-0
A$I9708[is.na(A$I9708) & A$E8043000==-3]<-0
A$I9708[is.na(A$I9708) & A$E8043000 >= 199800]<-0
A$I9708[is.na(A$I9708) & A$E8043500==0]<-0
A$I9708[is.na(A$I9708) & A$E8043500==-3]<-0
table(A$E8021708, useNA="always")
table(A$I9708, useNA="always")
A$I9709<-NA
A$I9709[A$E8021709==0]<-0
A$I9709[A$E8021709==99]<-0
A$I9709[A$E8021709==1]<-1
A$I9709[is.na(A$I9709) & A$E8043000==-4]<-0
A$I9709[is.na(A$I9709) & A$E8043000==-3]<-0
A$I9709[is.na(A$I9709) & A$E8043000 >= 199800]<-0
A$I9709[is.na(A$I9709) & A$E8043500==0]<-0
A$I9709[is.na(A$I9709) & A$E8043500==-3]<-0
table(A$E8021709, useNA="always")
table(A$I9709, useNA="always")
A$I9710<-NA
A$I9710[A$E8021710==0]<-0
A$I9710[A$E8021710==99]<-0
A$I9710[A$E8021710==1]<-1
A$I9710[is.na(A$I9710) & A$E8043000==-4]<-0
A$I9710[is.na(A$I9710) & A$E8043000==-3]<-0
A$I9710[is.na(A$I9710) & A$E8043000 >= 199800]<-0
A$I9710[is.na(A$I9710) & A$E8043500==0]<-0
A$I9710[is.na(A$I9710) & A$E8043500==-3]<-0
table(A$E8021710, useNA="always")
table(A$I9710, useNA="always")
A$I9711<-NA
A$I9711[A$E8021711==0]<-0
A$I9711[A$E8021711==99]<-0
A$I9711[A$E8021711==1]<-1
A$I9711[is.na(A$I9711) & A$E8043000==-4]<-0
A$I9711[is.na(A$I9711) & A$E8043000==-3]<-0
A$I9711[is.na(A$I9711) & A$E8043000 >= 199800]<-0
A$I9711[is.na(A$I9711) & A$E8043500==0]<-0
A$I9711[is.na(A$I9711) & A$E8043500==-3]<-0
table(A$E8021711, useNA="always")
table(A$I9711, useNA="always")
A$I9712<-NA
A$I9712[A$E8021712==0]<-0
A$I9712[A$E8021712==99]<-0
A$I9712[A$E8021712==1]<-1
A$I9712[is.na(A$I9712) & A$E8043000==-4]<-0
A$I9712[is.na(A$I9712) & A$E8043000==-3]<-0
```

© 2023 Silver IA et al. *JAMA Network Open.*

```
A$I9712[is.na(A$I9712) & A$E8043000 >= 199800]<-0
A$I9712[is.na(A$I9712) & A$E8043500==0]<-0
A$I9712[is.na(A$I9712) & A$E8043500==-3]<-0
table(A$E8021712, useNA="always")
table(A$I9712, useNA="always")




A$I97<-
rowSums(data.frame(A$I9701,A$I9702,A$I9703,A$I9704,A$I9705,A$I9706,A$I9707,A$
I9708,A$I9709,A$I9710,A$I9711,A$I9712), na.rm = T)
table(A$I97)
### Incarceration (1998)

table(A$E8021801, useNA="always")
table(A$E8021802)
table(A$E8021803)
table(A$E8021804)
table(A$E8021805)
table(A$E8021806)
table(A$E8021807)
table(A$E8021808)
table(A$E8021809)
table(A$E8021810)
table(A$E8021811)
table(A$E8021812)

A$I9801<-NA
A$I9801[A$E8021801==0]<-0
A$I9801[A$E8021801==99]<-0
A$I9801[A$E8021801==1]<-1
A$I9801[is.na(A$I9801) & A$E8043000==-4]<-0
A$I9801[is.na(A$I9801) & A$E8043000==-3]<-0
A$I9801[is.na(A$I9801) & A$E8043000 >= 199900]<-0
A$I9801[is.na(A$I9801) & A$E8043500==0]<-0
A$I9801[is.na(A$I9801) & A$E8043500==-3]<-0
table(A$E8021801, useNA="always")
table(A$I9801, useNA="always")
A$I9802<-NA
A$I9802[A$E8021802==0]<-0
A$I9802[A$E8021802==99]<-0
A$I9802[A$E8021802==1]<-1
A$I9802[is.na(A$I9802) & A$E8043000==-4]<-0
A$I9802[is.na(A$I9802) & A$E8043000==-3]<-0
A$I9802[is.na(A$I9802) & A$E8043000 >= 199900]<-0
A$I9802[is.na(A$I9802) & A$E8043500==0]<-0
A$I9802[is.na(A$I9802) & A$E8043500==-3]<-0
table(A$E8021802, useNA="always")
table(A$I9802, useNA="always")
A$I9803<-NA
A$I9803[A$E8021803==0]<-0
A$I9803[A$E8021803==99]<-0
A$I9803[A$E8021803==1]<-1
A$I9803[is.na(A$I9803) & A$E8043000==-4]<-0
A$I9803[is.na(A$I9803) & A$E8043000==-3]<-0
```

© 2023 Silver IA et al. *JAMA Network Open.*

```
A$I9803[is.na(A$I9803) & A$E8043000 >= 199900]<-0
A$I9803[is.na(A$I9803) & A$E8043500==0]<-0
A$I9803[is.na(A$I9803) & A$E8043500==-3]<-0
table(A$E8021803, useNA="always")
table(A$I9803, useNA="always")
A$I9804<-NA
A$I9804[A$E8021804==0]<-0
A$I9804[A$E8021804==99]<-0
A$I9804[A$E8021804==1]<-1
A$I9804[is.na(A$I9804) & A$E8043000==-4]<-0
A$I9804[is.na(A$I9804) & A$E8043000==-3]<-0
A$I9804[is.na(A$I9804) & A$E8043000 >= 199900]<-0
A$I9804[is.na(A$I9804) & A$E8043500==0]<-0
A$I9804[is.na(A$I9804) & A$E8043500==-3]<-0
table(A$E8021804, useNA="always")
table(A$I9804, useNA="always")
A$I9805<-NA
A$I9805[A$E8021805==0]<-0
A$I9805[A$E8021805==99]<-0
A$I9805[A$E8021805==1]<-1
A$I9805[is.na(A$I9805) & A$E8043000==-4]<-0
A$I9805[is.na(A$I9805) & A$E8043000==-3]<-0
A$I9805[is.na(A$I9805) & A$E8043000 >= 199900]<-0
A$I9805[is.na(A$I9805) & A$E8043500==0]<-0
A$I9805[is.na(A$I9805) & A$E8043500==-3]<-0
table(A$E8021805, useNA="always")
table(A$I9805, useNA="always")
A$I9806<-NA
A$I9806[A$E8021806==0]<-0
A$I9806[A$E8021806==99]<-0
A$I9806[A$E8021806==1]<-1
A$I9806[is.na(A$I9806) & A$E8043000==-4]<-0
A$I9806[is.na(A$I9806) & A$E8043000==-3]<-0
A$I9806[is.na(A$I9806) & A$E8043000 >= 199900]<-0
A$I9806[is.na(A$I9806) & A$E8043500==0]<-0
A$I9806[is.na(A$I9806) & A$E8043500==-3]<-0
table(A$E8021806, useNA="always")
table(A$I9806, useNA="always")
A$I9807<-NA
A$I9807[A$E8021807==0]<-0
A$I9807[A$E8021807==99]<-0
A$I9807[A$E8021807==1]<-1
A$I9807[is.na(A$I9807) & A$E8043000==-4]<-0
A$I9807[is.na(A$I9807) & A$E8043000==-3]<-0
A$I9807[is.na(A$I9807) & A$E8043000 >= 199900]<-0
A$I9807[is.na(A$I9807) & A$E8043500==0]<-0
A$I9807[is.na(A$I9807) & A$E8043500==-3]<-0
table(A$E8021807, useNA="always")
table(A$I9807, useNA="always")
A$I9808<-NA
A$I9808[A$E8021808==0]<-0
A$I9808[A$E8021808==99]<-0
A$I9808[A$E8021808==1]<-1
A$I9808[is.na(A$I9808) & A$E8043000==-4]<-0
A$I9808[is.na(A$I9808) & A$E8043000==-3]<-0
A$I9808[is.na(A$I9808) & A$E8043000 >= 199900]<-0
```

© 2023 Silver IA et al. *JAMA Network Open.*

```
A$I9808[is.na(A$I9808) & A$E8043500==0]<-0
A$I9808[is.na(A$I9808) & A$E8043500==-3]<-0
table(A$E8021808, useNA="always")
table(A$I9808, useNA="always")
A$I9809<-NA
A$I9809[A$E8021809==0]<-0
A$I9809[A$E8021809==99]<-0
A$I9809[A$E8021809==1]<-1
A$I9809[is.na(A$I9809) & A$E8043000==-4]<-0
A$I9809[is.na(A$I9809) & A$E8043000==-3]<-0
A$I9809[is.na(A$I9809) & A$E8043000 >= 199900]<-0
A$I9809[is.na(A$I9809) & A$E8043500==0]<-0
A$I9809[is.na(A$I9809) & A$E8043500==-3]<-0
table(A$E8021809, useNA="always")
table(A$I9809, useNA="always")
A$I9810<-NA
A$I9810[A$E8021810==0]<-0
A$I9810[A$E8021810==99]<-0
A$I9810[A$E8021810==1]<-1
A$I9810[is.na(A$I9810) & A$E8043000==-4]<-0
A$I9810[is.na(A$I9810) & A$E8043000==-3]<-0
A$I9810[is.na(A$I9810) & A$E8043000 >= 199900]<-0
A$I9810[is.na(A$I9810) & A$E8043500==0]<-0
A$I9810[is.na(A$I9810) & A$E8043500==-3]<-0
table(A$E8021810, useNA="always")
table(A$I9810, useNA="always")
A$I9811<-NA
A$I9811[A$E8021811==0]<-0
A$I9811[A$E8021811==99]<-0
A$I9811[A$E8021811==1]<-1
A$I9811[is.na(A$I9811) & A$E8043000==-4]<-0
A$I9811[is.na(A$I9811) & A$E8043000==-3]<-0
A$I9811[is.na(A$I9811) & A$E8043000 >= 199900]<-0
A$I9811[is.na(A$I9811) & A$E8043500==0]<-0
A$I9811[is.na(A$I9811) & A$E8043500==-3]<-0
table(A$E8021811, useNA="always")
table(A$I9811, useNA="always")
A$I9812<-NA
A$I9812[A$E8021812==0]<-0
A$I9812[A$E8021812==99]<-0
A$I9812[A$E8021812==1]<-1
A$I9812[is.na(A$I9812) & A$E8043000==-4]<-0
A$I9812[is.na(A$I9812) & A$E8043000==-3]<-0
A$I9812[is.na(A$I9812) & A$E8043000 >= 199900]<-0
A$I9812[is.na(A$I9812) & A$E8043500==0]<-0
A$I9812[is.na(A$I9812) & A$E8043500==-3]<-0
table(A$E8021812, useNA="always")
table(A$I9812, useNA="always")



A$I98<-
rowSums(data.frame(A$I9801,A$I9802,A$I9803,A$I9804,A$I9805,A$I9806,A$I9807,A$
I9808,A$I9809,A$I9810,A$I9811,A$I9812), na.rm = T)
table(A$I98)
```

© 2023 Silver IA et al. *JAMA Network Open.*

```
### Incarceration (1999)

table(A$E8021901, useNA="always")
table(A$E8021902)
table(A$E8021903)
table(A$E8021904)
table(A$E8021905)
table(A$E8021906)
table(A$E8021907)
table(A$E8021908)
table(A$E8021909)
table(A$E8021910)
table(A$E8021911)
table(A$E8021912)

A$I9901<-NA
A$I9901[A$E8021901==0]<-0
A$I9901[A$E8021901==99]<-0
A$I9901[A$E8021901==1]<-1
A$I9901[is.na(A$I9901) & A$E8043000==-4]<-0
A$I9901[is.na(A$I9901) & A$E8043000==-3]<-0
A$I9901[is.na(A$I9901) & A$E8043000 >= 200000]<-0
A$I9901[is.na(A$I9901) & A$E8043500==0]<-0
A$I9901[is.na(A$I9901) & A$E8043500==-3]<-0
table(A$E8021901, useNA="always")
table(A$I9901, useNA="always")
A$I9902<-NA
A$I9902[A$E8021902==0]<-0
A$I9902[A$E8021902==99]<-0
A$I9902[A$E8021902==1]<-1
A$I9902[is.na(A$I9902) & A$E8043000==-4]<-0
A$I9902[is.na(A$I9902) & A$E8043000==-3]<-0
A$I9902[is.na(A$I9902) & A$E8043000 >= 200000]<-0
A$I9902[is.na(A$I9902) & A$E8043500==0]<-0
A$I9902[is.na(A$I9902) & A$E8043500==-3]<-0
table(A$E8021902, useNA="always")
table(A$I9902, useNA="always")
A$I9903<-NA
A$I9903[A$E8021903==0]<-0
A$I9903[A$E8021903==99]<-0
A$I9903[A$E8021903==1]<-1
A$I9903[is.na(A$I9903) & A$E8043000==-4]<-0
A$I9903[is.na(A$I9903) & A$E8043000==-3]<-0
A$I9903[is.na(A$I9903) & A$E8043000 >= 200000]<-0
A$I9903[is.na(A$I9903) & A$E8043500==0]<-0
A$I9903[is.na(A$I9903) & A$E8043500==-3]<-0
table(A$E8021903, useNA="always")
table(A$I9903, useNA="always")
A$I9904<-NA
A$I9904[A$E8021904==0]<-0
A$I9904[A$E8021904==99]<-0
A$I9904[A$E8021904==1]<-1
A$I9904[is.na(A$I9904) & A$E8043000==-4]<-0
A$I9904[is.na(A$I9904) & A$E8043000==-3]<-0
A$I9904[is.na(A$I9904) & A$E8043000 >= 200000]<-0
A$I9904[is.na(A$I9904) & A$E8043500==0]<-0
```

© 2023 Silver IA et al. *JAMA Network Open.*

```
A$I9904[is.na(A$I9904) & A$E8043500==-3]<-0
table(A$E8021904, useNA="always")
table(A$I9904, useNA="always")
A$I9905<-NA
A$I9905[A$E8021905==0]<-0
A$I9905[A$E8021905==99]<-0
A$I9905[A$E8021905==1]<-1
A$I9905[is.na(A$I9905) & A$E8043000==-4]<-0
A$I9905[is.na(A$I9905) & A$E8043000==-3]<-0
A$I9905[is.na(A$I9905) & A$E8043000 >= 200000]<-0
A$I9905[is.na(A$I9905) & A$E8043500==0]<-0
A$I9905[is.na(A$I9905) & A$E8043500==-3]<-0
table(A$E8021905, useNA="always")
table(A$I9905, useNA="always")
A$I9906<-NA
A$I9906[A$E8021906==0]<-0
A$I9906[A$E8021906==99]<-0
A$I9906[A$E8021906==1]<-1
A$I9906[is.na(A$I9906) & A$E8043000==-4]<-0
A$I9906[is.na(A$I9906) & A$E8043000==-3]<-0
A$I9906[is.na(A$I9906) & A$E8043000 >= 200000]<-0
A$I9906[is.na(A$I9906) & A$E8043500==0]<-0
A$I9906[is.na(A$I9906) & A$E8043500==-3]<-0
table(A$E8021906, useNA="always")
table(A$I9906, useNA="always")
A$I9907<-NA
A$I9907[A$E8021907==0]<-0
A$I9907[A$E8021907==99]<-0
A$I9907[A$E8021907==1]<-1
A$I9907[is.na(A$I9907) & A$E8043000==-4]<-0
A$I9907[is.na(A$I9907) & A$E8043000==-3]<-0
A$I9907[is.na(A$I9907) & A$E8043000 >= 200000]<-0
A$I9907[is.na(A$I9907) & A$E8043500==0]<-0
A$I9907[is.na(A$I9907) & A$E8043500==-3]<-0
table(A$E8021907, useNA="always")
table(A$I9907, useNA="always")
A$I9908<-NA
A$I9908[A$E8021908==0]<-0
A$I9908[A$E8021908==99]<-0
A$I9908[A$E8021908==1]<-1
A$I9908[is.na(A$I9908) & A$E8043000==-4]<-0
A$I9908[is.na(A$I9908) & A$E8043000==-3]<-0
A$I9908[is.na(A$I9908) & A$E8043000 >= 200000]<-0
A$I9908[is.na(A$I9908) & A$E8043500==0]<-0
A$I9908[is.na(A$I9908) & A$E8043500==-3]<-0
table(A$E8021908, useNA="always")
table(A$I9908, useNA="always")
A$I9909<-NA
A$I9909[A$E8021909==0]<-0
A$I9909[A$E8021909==99]<-0
A$I9909[A$E8021909==1]<-1
A$I9909[is.na(A$I9909) & A$E8043000==-4]<-0
A$I9909[is.na(A$I9909) & A$E8043000==-3]<-0
A$I9909[is.na(A$I9909) & A$E8043000 >= 200000]<-0
A$I9909[is.na(A$I9909) & A$E8043500==0]<-0
A$I9909[is.na(A$I9909) & A$E8043500==-3]<-0
```

© 2023 Silver IA et al. *JAMA Network Open.*

```
table(A$E8021909, useNA="always")
table(A$I9909, useNA="always")
A$I9910<-NA
A$I9910[A$E8021910==0]<-0
A$I9910[A$E8021910==99]<-0
A$I9910[A$E8021910==1]<-1
A$I9910[is.na(A$I9910) & A$E8043000==-4]<-0
A$I9910[is.na(A$I9910) & A$E8043000==-3]<-0
A$I9910[is.na(A$I9910) & A$E8043000 >= 200000]<-0
A$I9910[is.na(A$I9910) & A$E8043500==0]<-0
A$I9910[is.na(A$I9910) & A$E8043500==-3]<-0
table(A$E8021910, useNA="always")
table(A$I9910, useNA="always")
A$I9911<-NA
A$I9911[A$E8021911==0]<-0
A$I9911[A$E8021911==99]<-0
A$I9911[A$E8021911==1]<-1
A$I9911[is.na(A$I9911) & A$E8043000==-4]<-0
A$I9911[is.na(A$I9911) & A$E8043000==-3]<-0
A$I9911[is.na(A$I9911) & A$E8043000 >= 200000]<-0
A$I9911[is.na(A$I9911) & A$E8043500==0]<-0
A$I9911[is.na(A$I9911) & A$E8043500==-3]<-0
table(A$E8021911, useNA="always")
table(A$I9911, useNA="always")
A$I9912<-NA
A$I9912[A$E8021912==0]<-0
A$I9912[A$E8021912==99]<-0
A$I9912[A$E8021912==1]<-1
A$I9912[is.na(A$I9912) & A$E8043000==-4]<-0
A$I9912[is.na(A$I9912) & A$E8043000==-3]<-0
A$I9912[is.na(A$I9912) & A$E8043000 >= 200000]<-0
A$I9912[is.na(A$I9912) & A$E8043500==0]<-0
A$I9912[is.na(A$I9912) & A$E8043500==-3]<-0
table(A$E8021912, useNA="always")
table(A$I9912, useNA="always")



A$I99<-
rowSums(data.frame(A$I9901,A$I9902,A$I9903,A$I9904,A$I9905,A$I9906,A$I9907,A$
I9908,A$I9909,A$I9910,A$I9911,A$I9912), na.rm = T)
table(A$I99)
### Incarceration (2000)

table(A$E8022001, useNA="always")
table(A$E8022002)
table(A$E8022003)
table(A$E8022004)
table(A$E8022005)
table(A$E8022006)
table(A$E8022007)
table(A$E8022008)
table(A$E8022009)
table(A$E8022010)
table(A$E8022011)
```

© 2023 Silver IA et al. *JAMA Network Open.*

```
table(A$E8022012)

A$I0001<-NA
A$I0001[A$E8022001==0]<-0
A$I0001[A$E8022001==99]<-0
A$I0001[A$E8022001==1]<-1
A$I0001[is.na(A$I0001) & A$E8043000==-4]<-0
A$I0001[is.na(A$I0001) & A$E8043000==-3]<-0
A$I0001[is.na(A$I0001) & A$E8043000 >= 200100]<-0
A$I0001[is.na(A$I0001) & A$E8043500==0]<-0
A$I0001[is.na(A$I0001) & A$E8043500==-3]<-0
table(A$E8022001, useNA="always")
table(A$I0001, useNA="always")
A$I0002<-NA
A$I0002[A$E8022002==0]<-0
A$I0002[A$E8022002==99]<-0
A$I0002[A$E8022002==1]<-1
A$I0002[is.na(A$I0002) & A$E8043000==-4]<-0
A$I0002[is.na(A$I0002) & A$E8043000==-3]<-0
A$I0002[is.na(A$I0002) & A$E8043000 >= 200100]<-0
A$I0002[is.na(A$I0002) & A$E8043500==0]<-0
A$I0002[is.na(A$I0002) & A$E8043500==-3]<-0
table(A$E8022002, useNA="always")
table(A$I0002, useNA="always")
A$I0003<-NA
A$I0003[A$E8022003==0]<-0
A$I0003[A$E8022003==99]<-0
A$I0003[A$E8022003==1]<-1
A$I0003[is.na(A$I0003) & A$E8043000==-4]<-0
A$I0003[is.na(A$I0003) & A$E8043000==-3]<-0
A$I0003[is.na(A$I0003) & A$E8043000 >= 200100]<-0
A$I0003[is.na(A$I0003) & A$E8043500==0]<-0
A$I0003[is.na(A$I0003) & A$E8043500==-3]<-0
table(A$E8022003, useNA="always")
table(A$I0003, useNA="always")
A$I0004<-NA
A$I0004[A$E8022004==0]<-0
A$I0004[A$E8022004==99]<-0
A$I0004[A$E8022004==1]<-1
A$I0004[is.na(A$I0004) & A$E8043000==-4]<-0
A$I0004[is.na(A$I0004) & A$E8043000==-3]<-0
A$I0004[is.na(A$I0004) & A$E8043000 >= 200100]<-0
A$I0004[is.na(A$I0004) & A$E8043500==0]<-0
A$I0004[is.na(A$I0004) & A$E8043500==-3]<-0
table(A$E8022004, useNA="always")
table(A$I0004, useNA="always")
A$I0005<-NA
A$I0005[A$E8022005==0]<-0
A$I0005[A$E8022005==99]<-0
A$I0005[A$E8022005==1]<-1
A$I0005[is.na(A$I0005) & A$E8043000==-4]<-0
A$I0005[is.na(A$I0005) & A$E8043000==-3]<-0
A$I0005[is.na(A$I0005) & A$E8043000 >= 200100]<-0
A$I0005[is.na(A$I0005) & A$E8043500==0]<-0
A$I0005[is.na(A$I0005) & A$E8043500==-3]<-0
table(A$E8022005, useNA="always")
```

© 2023 Silver IA et al. *JAMA Network Open.*

```
table(A$I0005, useNA="always")
A$I0006<-NA
A$I0006[A$E8022006==0]<-0
A$I0006[A$E8022006==99]<-0
A$I0006[A$E8022006==1]<-1
A$I0006[is.na(A$I0006) & A$E8043000==-4]<-0
A$I0006[is.na(A$I0006) & A$E8043000==-3]<-0
A$I0006[is.na(A$I0006) & A$E8043000 >= 200100]<-0
A$I0006[is.na(A$I0006) & A$E8043500==0]<-0
A$I0006[is.na(A$I0006) & A$E8043500==-3]<-0
table(A$E8022006, useNA="always")
table(A$I0006, useNA="always")
A$I0007<-NA
A$I0007[A$E8022007==0]<-0
A$I0007[A$E8022007==99]<-0
A$I0007[A$E8022007==1]<-1
A$I0007[is.na(A$I0007) & A$E8043000==-4]<-0
A$I0007[is.na(A$I0007) & A$E8043000==-3]<-0
A$I0007[is.na(A$I0007) & A$E8043000 >= 200100]<-0
A$I0007[is.na(A$I0007) & A$E8043500==0]<-0
A$I0007[is.na(A$I0007) & A$E8043500==-3]<-0
table(A$E8022007, useNA="always")
table(A$I0007, useNA="always")
A$I0008<-NA
A$I0008[A$E8022008==0]<-0
A$I0008[A$E8022008==99]<-0
A$I0008[A$E8022008==1]<-1
A$I0008[is.na(A$I0008) & A$E8043000==-4]<-0
A$I0008[is.na(A$I0008) & A$E8043000==-3]<-0
A$I0008[is.na(A$I0008) & A$E8043000 >= 200100]<-0
A$I0008[is.na(A$I0008) & A$E8043500==0]<-0
A$I0008[is.na(A$I0008) & A$E8043500==-3]<-0
table(A$E8022008, useNA="always")
table(A$I0008, useNA="always")
A$I0009<-NA
A$I0009[A$E8022009==0]<-0
A$I0009[A$E8022009==99]<-0
A$I0009[A$E8022009==1]<-1
A$I0009[is.na(A$I0009) & A$E8043000==-4]<-0
A$I0009[is.na(A$I0009) & A$E8043000==-3]<-0
A$I0009[is.na(A$I0009) & A$E8043000 >= 200100]<-0
A$I0009[is.na(A$I0009) & A$E8043500==0]<-0
A$I0009[is.na(A$I0009) & A$E8043500==-3]<-0
table(A$E8022009, useNA="always")
table(A$I0009, useNA="always")
A$I0010<-NA
A$I0010[A$E8022010==0]<-0
A$I0010[A$E8022010==99]<-0
A$I0010[A$E8022010==1]<-1
A$I0010[is.na(A$I0010) & A$E8043000==-4]<-0
A$I0010[is.na(A$I0010) & A$E8043000==-3]<-0
A$I0010[is.na(A$I0010) & A$E8043000 >= 200100]<-0
A$I0010[is.na(A$I0010) & A$E8043500==0]<-0
A$I0010[is.na(A$I0010) & A$E8043500==-3]<-0
table(A$E8022010, useNA="always")
table(A$I0010, useNA="always")
```

© 2023 Silver IA et al. *JAMA Network Open.*

```
A$I0011<-NA
A$I0011[A$E8022011==0]<-0
A$I0011[A$E8022011==99]<-0
A$I0011[A$E8022011==1]<-1
A$I0011[is.na(A$I0011) & A$E8043000==-4]<-0
A$I0011[is.na(A$I0011) & A$E8043000==-3]<-0
A$I0011[is.na(A$I0011) & A$E8043000 >= 200100]<-0
A$I0011[is.na(A$I0011) & A$E8043500==0]<-0
A$I0011[is.na(A$I0011) & A$E8043500==-3]<-0
table(A$E8022011, useNA="always")
table(A$I0011, useNA="always")
A$I0012<-NA
A$I0012[A$E8022012==0]<-0
A$I0012[A$E8022012==99]<-0
A$I0012[A$E8022012==1]<-1
A$I0012[is.na(A$I0012) & A$E8043000==-4]<-0
A$I0012[is.na(A$I0012) & A$E8043000==-3]<-0
A$I0012[is.na(A$I0012) & A$E8043000 >= 200100]<-0
A$I0012[is.na(A$I0012) & A$E8043500==0]<-0
A$I0012[is.na(A$I0012) & A$E8043500==-3]<-0
table(A$E8022012, useNA="always")
table(A$I0012, useNA="always")




A$I00<-
rowSums(data.frame(A$I0001,A$I0002,A$I0003,A$I0004,A$I0005,A$I0006,A$I0007,A$
I0008,A$I0009,A$I0010,A$I0011,A$I0012), na.rm = T)
table(A$I00)
### Incarceration (2001)

table(A$E8022101, useNA="always")
table(A$E8022102)
table(A$E8022103)
table(A$E8022104)
table(A$E8022105)
table(A$E8022106)
table(A$E8022107)
table(A$E8022108)
table(A$E8022109)
table(A$E8022110)
table(A$E8022111)
table(A$E8022112)

A$I0101<-NA
A$I0101[A$E8022101==0]<-0
A$I0101[A$E8022101==99]<-0
A$I0101[A$E8022101==1]<-1
A$I0101[is.na(A$I0101) & A$E8043000==-4]<-0
A$I0101[is.na(A$I0101) & A$E8043000==-3]<-0
A$I0101[is.na(A$I0101) & A$E8043000 >= 200200]<-0
A$I0101[is.na(A$I0101) & A$E8043500==0]<-0
A$I0101[is.na(A$I0101) & A$E8043500==-3]<-0
table(A$E8022101, useNA="always")
table(A$I0101, useNA="always")
```

© 2023 Silver IA et al. *JAMA Network Open.*

```
A$I0102<-NA
A$I0102[A$E8022102==0]<-0
A$I0102[A$E8022102==99]<-0
A$I0102[A$E8022102==1]<-1
A$I0102[is.na(A$I0102) & A$E8043000==-4]<-0
A$I0102[is.na(A$I0102) & A$E8043000==-3]<-0
A$I0102[is.na(A$I0102) & A$E8043000 >= 200200]<-0
A$I0102[is.na(A$I0102) & A$E8043500==0]<-0
A$I0102[is.na(A$I0102) & A$E8043500==-3]<-0
table(A$E8022102, useNA="always")
table(A$I0102, useNA="always")
A$I0103<-NA
A$I0103[A$E8022103==0]<-0
A$I0103[A$E8022103==99]<-0
A$I0103[A$E8022103==1]<-1
A$I0103[is.na(A$I0103) & A$E8043000==-4]<-0
A$I0103[is.na(A$I0103) & A$E8043000==-3]<-0
A$I0103[is.na(A$I0103) & A$E8043000 >= 200200]<-0
A$I0103[is.na(A$I0103) & A$E8043500==0]<-0
A$I0103[is.na(A$I0103) & A$E8043500==-3]<-0
table(A$E8022103, useNA="always")
table(A$I0103, useNA="always")
A$I0104<-NA
A$I0104[A$E8022104==0]<-0
A$I0104[A$E8022104==99]<-0
A$I0104[A$E8022104==1]<-1
A$I0104[is.na(A$I0104) & A$E8043000==-4]<-0
A$I0104[is.na(A$I0104) & A$E8043000==-3]<-0
A$I0104[is.na(A$I0104) & A$E8043000 >= 200200]<-0
A$I0104[is.na(A$I0104) & A$E8043500==0]<-0
A$I0104[is.na(A$I0104) & A$E8043500==-3]<-0
table(A$E8022104, useNA="always")
table(A$I0104, useNA="always")
A$I0105<-NA
A$I0105[A$E8022105==0]<-0
A$I0105[A$E8022105==99]<-0
A$I0105[A$E8022105==1]<-1
A$I0105[is.na(A$I0105) & A$E8043000==-4]<-0
A$I0105[is.na(A$I0105) & A$E8043000==-3]<-0
A$I0105[is.na(A$I0105) & A$E8043000 >= 200200]<-0
A$I0105[is.na(A$I0105) & A$E8043500==0]<-0
A$I0105[is.na(A$I0105) & A$E8043500==-3]<-0
table(A$E8022105, useNA="always")
table(A$I0105, useNA="always")
A$I0106<-NA
A$I0106[A$E8022106==0]<-0
A$I0106[A$E8022106==99]<-0
A$I0106[A$E8022106==1]<-1
A$I0106[is.na(A$I0106) & A$E8043000==-4]<-0
A$I0106[is.na(A$I0106) & A$E8043000==-3]<-0
A$I0106[is.na(A$I0106) & A$E8043000 >= 200200]<-0
A$I0106[is.na(A$I0106) & A$E8043500==0]<-0
A$I0106[is.na(A$I0106) & A$E8043500==-3]<-0
table(A$E8022106, useNA="always")
table(A$I0106, useNA="always")
A$I0107<-NA
```

© 2023 Silver IA et al. *JAMA Network Open.*

```
A$I0107[A$E8022107==0]<-0
A$I0107[A$E8022107==99]<-0
A$I0107[A$E8022107==1]<-1
A$I0107[is.na(A$I0107) & A$E8043000==-4]<-0
A$I0107[is.na(A$I0107) & A$E8043000==-3]<-0
A$I0107[is.na(A$I0107) & A$E8043000 >= 200200]<-0
A$I0107[is.na(A$I0107) & A$E8043500==0]<-0
A$I0107[is.na(A$I0107) & A$E8043500==-3]<-0
table(A$E8022107, useNA="always")
table(A$I0107, useNA="always")
A$I0108<-NA
A$I0108[A$E8022108==0]<-0
A$I0108[A$E8022108==99]<-0
A$I0108[A$E8022108==1]<-1
A$I0108[is.na(A$I0108) & A$E8043000==-4]<-0
A$I0108[is.na(A$I0108) & A$E8043000==-3]<-0
A$I0108[is.na(A$I0108) & A$E8043000 >= 200200]<-0
A$I0108[is.na(A$I0108) & A$E8043500==0]<-0
A$I0108[is.na(A$I0108) & A$E8043500==-3]<-0
table(A$E8022108, useNA="always")
table(A$I0108, useNA="always")
A$I0109<-NA
A$I0109[A$E8022109==0]<-0
A$I0109[A$E8022109==99]<-0
A$I0109[A$E8022109==1]<-1
A$I0109[is.na(A$I0109) & A$E8043000==-4]<-0
A$I0109[is.na(A$I0109) & A$E8043000==-3]<-0
A$I0109[is.na(A$I0109) & A$E8043000 >= 200200]<-0
A$I0109[is.na(A$I0109) & A$E8043500==0]<-0
A$I0109[is.na(A$I0109) & A$E8043500==-3]<-0
table(A$E8022109, useNA="always")
table(A$I0109, useNA="always")
A$I0110<-NA
A$I0110[A$E8022110==0]<-0
A$I0110[A$E8022110==99]<-0
A$I0110[A$E8022110==1]<-1
A$I0110[is.na(A$I0110) & A$E8043000==-4]<-0
A$I0110[is.na(A$I0110) & A$E8043000==-3]<-0
A$I0110[is.na(A$I0110) & A$E8043000 >= 200200]<-0
A$I0110[is.na(A$I0110) & A$E8043500==0]<-0
A$I0110[is.na(A$I0110) & A$E8043500==-3]<-0
table(A$E8022110, useNA="always")
table(A$I0110, useNA="always")
A$I0111<-NA
A$I0111[A$E8022111==0]<-0
A$I0111[A$E8022111==99]<-0
A$I0111[A$E8022111==1]<-1
A$I0111[is.na(A$I0111) & A$E8043000==-4]<-0
A$I0111[is.na(A$I0111) & A$E8043000==-3]<-0
A$I0111[is.na(A$I0111) & A$E8043000 >= 200200]<-0
A$I0111[is.na(A$I0111) & A$E8043500==0]<-0
A$I0111[is.na(A$I0111) & A$E8043500==-3]<-0
table(A$E8022111, useNA="always")
table(A$I0111, useNA="always")
A$I0112<-NA
A$I0112[A$E8022112==0]<-0
```

© 2023 Silver IA et al. *JAMA Network Open.*

```
A$I0112[A$E8022112==99]<-0
A$I0112[A$E8022112==1]<-1
A$I0112[is.na(A$I0112) & A$E8043000==-4]<-0
A$I0112[is.na(A$I0112) & A$E8043000==-3]<-0
A$I0112[is.na(A$I0112) & A$E8043000 >= 200200]<-0
A$I0112[is.na(A$I0112) & A$E8043500==0]<-0
A$I0112[is.na(A$I0112) & A$E8043500==-3]<-0
table(A$E8022112, useNA="always")
table(A$I0112, useNA="always")




A$I01<-
rowSums(data.frame(A$I0101,A$I0102,A$I0103,A$I0104,A$I0105,A$I0106,A$I0107,A$
I0108,A$I0109,A$I0110,A$I0111,A$I0112), na.rm = T)
A$I01[is.na(A$I01)]<-0
table(A$I01, useNA="always")
### Incarceration (2002)

table(A$E8022201, useNA="always")
table(A$E8022202)
table(A$E8022203)
table(A$E8022204)
table(A$E8022205)
table(A$E8022206)
table(A$E8022207)
table(A$E8022208)
table(A$E8022209)
table(A$E8022210)
table(A$E8022211)
table(A$E8022212)

A$I0201<-NA
A$I0201[A$E8022201==0]<-0
A$I0201[A$E8022201==99]<-0
A$I0201[A$E8022201==1]<-1
A$I0201[is.na(A$I0201) & A$E8043000==-4]<-0
A$I0201[is.na(A$I0201) & A$E8043000==-3]<-0
A$I0201[is.na(A$I0201) & A$E8043000 >= 200300]<-0
A$I0201[is.na(A$I0201) & A$E8043500==0]<-0
A$I0201[is.na(A$I0201) & A$E8043500==-3]<-0
table(A$E8022201, useNA="always")
table(A$I0201, useNA="always")
A$I0202<-NA
A$I0202[A$E8022202==0]<-0
A$I0202[A$E8022202==99]<-0
A$I0202[A$E8022202==1]<-1
A$I0202[is.na(A$I0202) & A$E8043000==-4]<-0
A$I0202[is.na(A$I0202) & A$E8043000==-3]<-0
A$I0202[is.na(A$I0202) & A$E8043000 >= 200300]<-0
A$I0202[is.na(A$I0202) & A$E8043500==0]<-0
A$I0202[is.na(A$I0202) & A$E8043500==-3]<-0
table(A$E8022202, useNA="always")
table(A$I0202, useNA="always")
A$I0203<-NA
```

© 2023 Silver IA et al. *JAMA Network Open.*

```
A$I0203[A$E8022203==0]<-0
A$I0203[A$E8022203==99]<-0
A$I0203[A$E8022203==1]<-1
A$I0203[is.na(A$I0203) & A$E8043000==-4]<-0
A$I0203[is.na(A$I0203) & A$E8043000==-3]<-0
A$I0203[is.na(A$I0203) & A$E8043000 >= 200300]<-0
A$I0203[is.na(A$I0203) & A$E8043500==0]<-0
A$I0203[is.na(A$I0203) & A$E8043500==-3]<-0
table(A$E8022203, useNA="always")
table(A$I0203, useNA="always")
A$I0204<-NA
A$I0204[A$E8022204==0]<-0
A$I0204[A$E8022204==99]<-0
A$I0204[A$E8022204==1]<-1
A$I0204[is.na(A$I0204) & A$E8043000==-4]<-0
A$I0204[is.na(A$I0204) & A$E8043000==-3]<-0
A$I0204[is.na(A$I0204) & A$E8043000 >= 200300]<-0
A$I0204[is.na(A$I0204) & A$E8043500==0]<-0
A$I0204[is.na(A$I0204) & A$E8043500==-3]<-0
table(A$E8022204, useNA="always")
table(A$I0204, useNA="always")
A$I0205<-NA
A$I0205[A$E8022205==0]<-0
A$I0205[A$E8022205==99]<-0
A$I0205[A$E8022205==1]<-1
A$I0205[is.na(A$I0205) & A$E8043000==-4]<-0
A$I0205[is.na(A$I0205) & A$E8043000==-3]<-0
A$I0205[is.na(A$I0205) & A$E8043000 >= 200300]<-0
A$I0205[is.na(A$I0205) & A$E8043500==0]<-0
A$I0205[is.na(A$I0205) & A$E8043500==-3]<-0
table(A$E8022205, useNA="always")
table(A$I0205, useNA="always")
A$I0206<-NA
A$I0206[A$E8022206==0]<-0
A$I0206[A$E8022206==99]<-0
A$I0206[A$E8022206==1]<-1
A$I0206[is.na(A$I0206) & A$E8043000==-4]<-0
A$I0206[is.na(A$I0206) & A$E8043000==-3]<-0
A$I0206[is.na(A$I0206) & A$E8043000 >= 200300]<-0
A$I0206[is.na(A$I0206) & A$E8043500==0]<-0
A$I0206[is.na(A$I0206) & A$E8043500==-3]<-0
table(A$E8022206, useNA="always")
table(A$I0206, useNA="always")
A$I0207<-NA
A$I0207[A$E8022207==0]<-0
A$I0207[A$E8022207==99]<-0
A$I0207[A$E8022207==1]<-1
A$I0207[is.na(A$I0207) & A$E8043000==-4]<-0
A$I0207[is.na(A$I0207) & A$E8043000==-3]<-0
A$I0207[is.na(A$I0207) & A$E8043000 >= 200300]<-0
A$I0207[is.na(A$I0207) & A$E8043500==0]<-0
A$I0207[is.na(A$I0207) & A$E8043500==-3]<-0
table(A$E8022207, useNA="always")
table(A$I0207, useNA="always")
A$I0208<-NA
A$I0208[A$E8022208==0]<-0
```

© 2023 Silver IA et al. *JAMA Network Open.*

```
A$I0208[A$E8022208==99]<-0
A$I0208[A$E8022208==1]<-1
A$I0208[is.na(A$I0208) & A$E8043000==-4]<-0
A$I0208[is.na(A$I0208) & A$E8043000==-3]<-0
A$I0208[is.na(A$I0208) & A$E8043000 >= 200300]<-0
A$I0208[is.na(A$I0208) & A$E8043500==0]<-0
A$I0208[is.na(A$I0208) & A$E8043500==-3]<-0
table(A$E8022208, useNA="always")
table(A$I0208, useNA="always")
A$I0209<-NA
A$I0209[A$E8022209==0]<-0
A$I0209[A$E8022209==99]<-0
A$I0209[A$E8022209==1]<-1
A$I0209[is.na(A$I0209) & A$E8043000==-4]<-0
A$I0209[is.na(A$I0209) & A$E8043000==-3]<-0
A$I0209[is.na(A$I0209) & A$E8043000 >= 200300]<-0
A$I0209[is.na(A$I0209) & A$E8043500==0]<-0
A$I0209[is.na(A$I0209) & A$E8043500==-3]<-0
table(A$E8022209, useNA="always")
table(A$I0209, useNA="always")
A$I0210<-NA
A$I0210[A$E8022210==0]<-0
A$I0210[A$E8022210==99]<-0
A$I0210[A$E8022210==1]<-1
A$I0210[is.na(A$I0210) & A$E8043000==-4]<-0
A$I0210[is.na(A$I0210) & A$E8043000==-3]<-0
A$I0210[is.na(A$I0210) & A$E8043000 >= 200300]<-0
A$I0210[is.na(A$I0210) & A$E8043500==0]<-0
A$I0210[is.na(A$I0210) & A$E8043500==-3]<-0
table(A$E8022210, useNA="always")
table(A$I0210, useNA="always")
A$I0211<-NA
A$I0211[A$E8022211==0]<-0
A$I0211[A$E8022211==99]<-0
A$I0211[A$E8022211==1]<-1
A$I0211[is.na(A$I0211) & A$E8043000==-4]<-0
A$I0211[is.na(A$I0211) & A$E8043000==-3]<-0
A$I0211[is.na(A$I0211) & A$E8043000 >= 200300]<-0
A$I0211[is.na(A$I0211) & A$E8043500==0]<-0
A$I0211[is.na(A$I0211) & A$E8043500==-3]<-0
table(A$E8022211, useNA="always")
table(A$I0211, useNA="always")
A$I0212<-NA
A$I0212[A$E8022212==0]<-0
A$I0212[A$E8022212==99]<-0
A$I0212[A$E8022212==1]<-1
A$I0212[is.na(A$I0212) & A$E8043000==-4]<-0
A$I0212[is.na(A$I0212) & A$E8043000==-3]<-0
A$I0212[is.na(A$I0212) & A$E8043000 >= 200300]<-0
A$I0212[is.na(A$I0212) & A$E8043500==0]<-0
A$I0212[is.na(A$I0212) & A$E8043500==-3]<-0
table(A$E8022212, useNA="always")
table(A$I0212, useNA="always")
```

© 2023 Silver IA et al. *JAMA Network Open.*

```
A$I02<-
rowSums(data.frame(A$I0201,A$I0202,A$I0203,A$I0204,A$I0205,A$I0206,A$I0207,A$
I0208,A$I0209,A$I0210,A$I0211,A$I0212), na.rm = T)
A$I02[is.na(A$I02)]<-0
table(A$I02, useNA="always")
### Incarceration (2003)

table(A$E8022301, useNA="always")
table(A$E8022302)
table(A$E8022303)
table(A$E8022304)
table(A$E8022305)
table(A$E8022306)
table(A$E8022307)
table(A$E8022308)
table(A$E8022309)
table(A$E8022310)
table(A$E8022311)
table(A$E8022312)

A$I0301<-NA
A$I0301[A$E8022301==0]<-0
A$I0301[A$E8022301==99]<-0
A$I0301[A$E8022301==1]<-1
A$I0301[is.na(A$I0301) & A$E8043000==-4]<-0
A$I0301[is.na(A$I0301) & A$E8043000==-3]<-0
A$I0301[is.na(A$I0301) & A$E8043000 >= 200400]<-0
A$I0301[is.na(A$I0301) & A$E8043500==0]<-0
A$I0301[is.na(A$I0301) & A$E8043500==-3]<-0
table(A$E8022301, useNA="always")
table(A$I0301, useNA="always")
A$I0302<-NA
A$I0302[A$E8022302==0]<-0
A$I0302[A$E8022302==99]<-0
A$I0302[A$E8022302==1]<-1
A$I0302[is.na(A$I0302) & A$E8043000==-4]<-0
A$I0302[is.na(A$I0302) & A$E8043000==-3]<-0
A$I0302[is.na(A$I0302) & A$E8043000 >= 200400]<-0
A$I0302[is.na(A$I0302) & A$E8043500==0]<-0
A$I0302[is.na(A$I0302) & A$E8043500==-3]<-0
table(A$E8022302, useNA="always")
table(A$I0302, useNA="always")
A$I0303<-NA
A$I0303[A$E8022303==0]<-0
A$I0303[A$E8022303==99]<-0
A$I0303[A$E8022303==1]<-1
A$I0303[is.na(A$I0303) & A$E8043000==-4]<-0
A$I0303[is.na(A$I0303) & A$E8043000==-3]<-0
A$I0303[is.na(A$I0303) & A$E8043000 >= 200400]<-0
A$I0303[is.na(A$I0303) & A$E8043500==0]<-0
A$I0303[is.na(A$I0303) & A$E8043500==-3]<-0
table(A$E8022303, useNA="always")
table(A$I0303, useNA="always")
A$I0304<-NA
A$I0304[A$E8022304==0]<-0
```

© 2023 Silver IA et al. *JAMA Network Open.*

```
A$I0304[A$E8022304==99]<-0
A$I0304[A$E8022304==1]<-1
A$I0304[is.na(A$I0304) & A$E8043000==-4]<-0
A$I0304[is.na(A$I0304) & A$E8043000==-3]<-0
A$I0304[is.na(A$I0304) & A$E8043000 >= 200400]<-0
A$I0304[is.na(A$I0304) & A$E8043500==0]<-0
A$I0304[is.na(A$I0304) & A$E8043500==-3]<-0
table(A$E8022304, useNA="always")
table(A$I0304, useNA="always")
A$I0305<-NA
A$I0305[A$E8022305==0]<-0
A$I0305[A$E8022305==99]<-0
A$I0305[A$E8022305==1]<-1
A$I0305[is.na(A$I0305) & A$E8043000==-4]<-0
A$I0305[is.na(A$I0305) & A$E8043000==-3]<-0
A$I0305[is.na(A$I0305) & A$E8043000 >= 200400]<-0
A$I0305[is.na(A$I0305) & A$E8043500==0]<-0
A$I0305[is.na(A$I0305) & A$E8043500==-3]<-0
table(A$E8022305, useNA="always")
table(A$I0305, useNA="always")
A$I0306<-NA
A$I0306[A$E8022306==0]<-0
A$I0306[A$E8022306==99]<-0
A$I0306[A$E8022306==1]<-1
A$I0306[is.na(A$I0306) & A$E8043000==-4]<-0
A$I0306[is.na(A$I0306) & A$E8043000==-3]<-0
A$I0306[is.na(A$I0306) & A$E8043000 >= 200400]<-0
A$I0306[is.na(A$I0306) & A$E8043500==0]<-0
A$I0306[is.na(A$I0306) & A$E8043500==-3]<-0
table(A$E8022306, useNA="always")
table(A$I0306, useNA="always")
A$I0307<-NA
A$I0307[A$E8022307==0]<-0
A$I0307[A$E8022307==99]<-0
A$I0307[A$E8022307==1]<-1
A$I0307[is.na(A$I0307) & A$E8043000==-4]<-0
A$I0307[is.na(A$I0307) & A$E8043000==-3]<-0
A$I0307[is.na(A$I0307) & A$E8043000 >= 200400]<-0
A$I0307[is.na(A$I0307) & A$E8043500==0]<-0
A$I0307[is.na(A$I0307) & A$E8043500==-3]<-0
table(A$E8022307, useNA="always")
table(A$I0307, useNA="always")
A$I0308<-NA
A$I0308[A$E8022308==0]<-0
A$I0308[A$E8022308==99]<-0
A$I0308[A$E8022308==1]<-1
A$I0308[is.na(A$I0308) & A$E8043000==-4]<-0
A$I0308[is.na(A$I0308) & A$E8043000==-3]<-0
A$I0308[is.na(A$I0308) & A$E8043000 >= 200400]<-0
A$I0308[is.na(A$I0308) & A$E8043500==0]<-0
A$I0308[is.na(A$I0308) & A$E8043500==-3]<-0
table(A$E8022308, useNA="always")
table(A$I0308, useNA="always")
A$I0309<-NA
A$I0309[A$E8022309==0]<-0
A$I0309[A$E8022309==99]<-0
```

© 2023 Silver IA et al. *JAMA Network Open.*

```
A$I0309[A$E8022309==1]<-1
A$I0309[is.na(A$I0309) & A$E8043000==-4]<-0
A$I0309[is.na(A$I0309) & A$E8043000==-3]<-0
A$I0309[is.na(A$I0309) & A$E8043000 >= 200400]<-0
A$I0309[is.na(A$I0309) & A$E8043500==0]<-0
A$I0309[is.na(A$I0309) & A$E8043500==-3]<-0
table(A$E8022309, useNA="always")
table(A$I0309, useNA="always")
A$I0310<-NA
A$I0310[A$E8022310==0]<-0
A$I0310[A$E8022310==99]<-0
A$I0310[A$E8022310==1]<-1
A$I0310[is.na(A$I0310) & A$E8043000==-4]<-0
A$I0310[is.na(A$I0310) & A$E8043000==-3]<-0
A$I0310[is.na(A$I0310) & A$E8043000 >= 200400]<-0
A$I0310[is.na(A$I0310) & A$E8043500==0]<-0
A$I0310[is.na(A$I0310) & A$E8043500==-3]<-0
table(A$E8022310, useNA="always")
table(A$I0310, useNA="always")
A$I0311<-NA
A$I0311[A$E8022311==0]<-0
A$I0311[A$E8022311==99]<-0
A$I0311[A$E8022311==1]<-1
A$I0311[is.na(A$I0311) & A$E8043000==-4]<-0
A$I0311[is.na(A$I0311) & A$E8043000==-3]<-0
A$I0311[is.na(A$I0311) & A$E8043000 >= 200400]<-0
A$I0311[is.na(A$I0311) & A$E8043500==0]<-0
A$I0311[is.na(A$I0311) & A$E8043500==-3]<-0
table(A$E8022311, useNA="always")
table(A$I0311, useNA="always")
A$I0312<-NA
A$I0312[A$E8022312==0]<-0
A$I0312[A$E8022312==99]<-0
A$I0312[A$E8022312==1]<-1
A$I0312[is.na(A$I0312) & A$E8043000==-4]<-0
A$I0312[is.na(A$I0312) & A$E8043000==-3]<-0
A$I0312[is.na(A$I0312) & A$E8043000 >= 200400]<-0
A$I0312[is.na(A$I0312) & A$E8043500==0]<-0
A$I0312[is.na(A$I0312) & A$E8043500==-3]<-0
table(A$E8022312, useNA="always")
table(A$I0312, useNA="always")




A$I03<-
rowSums(data.frame(A$I0301,A$I0302,A$I0303,A$I0304,A$I0305,A$I0306,A$I0307,A$
I0308,A$I0309,A$I0310,A$I0311,A$I0312),na.rm = T)
A$I03[is.na(A$I03)]<-0
table(A$I03, useNA="always")

### Incarceration (2004)

table(A$E8022401, useNA="always")
table(A$E8022402)
table(A$E8022403)
```

© 2023 Silver IA et al. *JAMA Network Open.*

```
table(A$E8022404)
table(A$E8022405)
table(A$E8022406)
table(A$E8022407)
table(A$E8022408)
table(A$E8022409)
table(A$E8022410)
table(A$E8022411)
table(A$E8022412)

A$I0401<-NA
A$I0401[A$E8022401==0]<-0
A$I0401[A$E8022401==99]<-0
A$I0401[A$E8022401==1]<-1
A$I0401[is.na(A$I0401) & A$E8043000==-4]<-0
A$I0401[is.na(A$I0401) & A$E8043000==-3]<-0
A$I0401[is.na(A$I0401) & A$E8043000 >= 200500]<-0
A$I0401[is.na(A$I0401) & A$E8043500==0]<-0
A$I0401[is.na(A$I0401) & A$E8043500==-3]<-0
table(A$E8022401, useNA="always")
table(A$I0401, useNA="always")
A$I0402<-NA
A$I0402[A$E8022402==0]<-0
A$I0402[A$E8022402==99]<-0
A$I0402[A$E8022402==1]<-1
A$I0402[is.na(A$I0402) & A$E8043000==-4]<-0
A$I0402[is.na(A$I0402) & A$E8043000==-3]<-0
A$I0402[is.na(A$I0402) & A$E8043000 >= 200500]<-0
A$I0402[is.na(A$I0402) & A$E8043500==0]<-0
A$I0402[is.na(A$I0402) & A$E8043500==-3]<-0
table(A$E8022402, useNA="always")
table(A$I0402, useNA="always")
A$I0403<-NA
A$I0403[A$E8022403==0]<-0
A$I0403[A$E8022403==99]<-0
A$I0403[A$E8022403==1]<-1
A$I0403[is.na(A$I0403) & A$E8043000==-4]<-0
A$I0403[is.na(A$I0403) & A$E8043000==-3]<-0
A$I0403[is.na(A$I0403) & A$E8043000 >= 200500]<-0
A$I0403[is.na(A$I0403) & A$E8043500==0]<-0
A$I0403[is.na(A$I0403) & A$E8043500==-3]<-0
table(A$E8022403, useNA="always")
table(A$I0403, useNA="always")
A$I0404<-NA
A$I0404[A$E8022404==0]<-0
A$I0404[A$E8022404==99]<-0
A$I0404[A$E8022404==1]<-1
A$I0404[is.na(A$I0404) & A$E8043000==-4]<-0
A$I0404[is.na(A$I0404) & A$E8043000==-3]<-0
A$I0404[is.na(A$I0404) & A$E8043000 >= 200500]<-0
A$I0404[is.na(A$I0404) & A$E8043500==0]<-0
A$I0404[is.na(A$I0404) & A$E8043500==-3]<-0
table(A$E8022404, useNA="always")
table(A$I0404, useNA="always")
A$I0405<-NA
A$I0405[A$E8022405==0]<-0
```

© 2023 Silver IA et al. *JAMA Network Open.*

```
A$I0405[A$E8022405==99]<-0
A$I0405[A$E8022405==1]<-1
A$I0405[is.na(A$I0405) & A$E8043000==-4]<-0
A$I0405[is.na(A$I0405) & A$E8043000==-3]<-0
A$I0405[is.na(A$I0405) & A$E8043000 >= 200500]<-0
A$I0405[is.na(A$I0405) & A$E8043500==0]<-0
A$I0405[is.na(A$I0405) & A$E8043500==-3]<-0
table(A$E8022405, useNA="always")
table(A$I0405, useNA="always")
A$I0406<-NA
A$I0406[A$E8022406==0]<-0
A$I0406[A$E8022406==99]<-0
A$I0406[A$E8022406==1]<-1
A$I0406[is.na(A$I0406) & A$E8043000==-4]<-0
A$I0406[is.na(A$I0406) & A$E8043000==-3]<-0
A$I0406[is.na(A$I0406) & A$E8043000 >= 200500]<-0
A$I0406[is.na(A$I0406) & A$E8043500==0]<-0
A$I0406[is.na(A$I0406) & A$E8043500==-3]<-0
table(A$E8022406, useNA="always")
table(A$I0406, useNA="always")
A$I0407<-NA
A$I0407[A$E8022407==0]<-0
A$I0407[A$E8022407==99]<-0
A$I0407[A$E8022407==1]<-1
A$I0407[is.na(A$I0407) & A$E8043000==-4]<-0
A$I0407[is.na(A$I0407) & A$E8043000==-3]<-0
A$I0407[is.na(A$I0407) & A$E8043000 >= 200500]<-0
A$I0407[is.na(A$I0407) & A$E8043500==0]<-0
A$I0407[is.na(A$I0407) & A$E8043500==-3]<-0
table(A$E8022407, useNA="always")
table(A$I0407, useNA="always")
A$I0408<-NA
A$I0408[A$E8022408==0]<-0
A$I0408[A$E8022408==99]<-0
A$I0408[A$E8022408==1]<-1
A$I0408[is.na(A$I0408) & A$E8043000==-4]<-0
A$I0408[is.na(A$I0408) & A$E8043000==-3]<-0
A$I0408[is.na(A$I0408) & A$E8043000 >= 200500]<-0
A$I0408[is.na(A$I0408) & A$E8043500==0]<-0
A$I0408[is.na(A$I0408) & A$E8043500==-3]<-0
table(A$E8022408, useNA="always")
table(A$I0408, useNA="always")
A$I0409<-NA
A$I0409[A$E8022409==0]<-0
A$I0409[A$E8022409==99]<-0
A$I0409[A$E8022409==1]<-1
A$I0409[is.na(A$I0409) & A$E8043000==-4]<-0
A$I0409[is.na(A$I0409) & A$E8043000==-3]<-0
A$I0409[is.na(A$I0409) & A$E8043000 >= 200500]<-0
A$I0409[is.na(A$I0409) & A$E8043500==0]<-0
A$I0409[is.na(A$I0409) & A$E8043500==-3]<-0
table(A$E8022409, useNA="always")
table(A$I0409, useNA="always")
A$I0410<-NA
A$I0410[A$E8022410==0]<-0
A$I0410[A$E8022410==99]<-0
```

```
A$I0410[A$E8022410==1]<-1
A$I0410[is.na(A$I0410) & A$E8043000==-4]<-0
A$I0410[is.na(A$I0410) & A$E8043000==-3]<-0
A$I0410[is.na(A$I0410) & A$E8043000 >= 200500]<-0
A$I0410[is.na(A$I0410) & A$E8043500==0]<-0
A$I0410[is.na(A$I0410) & A$E8043500==-3]<-0
table(A$E8022410, useNA="always")
table(A$I0410, useNA="always")
A$I0411<-NA
A$I0411[A$E8022411==0]<-0
A$I0411[A$E8022411==99]<-0
A$I0411[A$E8022411==1]<-1
A$I0411[is.na(A$I0411) & A$E8043000==-4]<-0
A$I0411[is.na(A$I0411) & A$E8043000==-3]<-0
A$I0411[is.na(A$I0411) & A$E8043000 >= 200500]<-0
A$I0411[is.na(A$I0411) & A$E8043500==0]<-0
A$I0411[is.na(A$I0411) & A$E8043500==-3]<-0
table(A$E8022411, useNA="always")
table(A$I0411, useNA="always")
A$I0412<-NA
A$I0412[A$E8022412==0]<-0
A$I0412[A$E8022412==99]<-0
A$I0412[A$E8022412==1]<-1
A$I0412[is.na(A$I0412) & A$E8043000==-4]<-0
A$I0412[is.na(A$I0412) & A$E8043000==-3]<-0
A$I0412[is.na(A$I0412) & A$E8043000 >= 200500]<-0
A$I0412[is.na(A$I0412) & A$E8043500==0]<-0
A$I0412[is.na(A$I0412) & A$E8043500==-3]<-0
table(A$E8022412, useNA="always")
table(A$I0412, useNA="always")




A$I04<-
rowSums(data.frame(A$I0401,A$I0402,A$I0403,A$I0404,A$I0405,A$I0406,A$I0407,A$
I0408,A$I0409,A$I0410,A$I0411,A$I0412), na.rm = T)
A$I04[is.na(A$I04)]<-0
table(A$I04, useNA="always")

### Incarceration (2005)

table(A$E8022501, useNA="always")
table(A$E8022502)
table(A$E8022503)
table(A$E8022504)
table(A$E8022505)
table(A$E8022506)
table(A$E8022507)
table(A$E8022508)
table(A$E8022509)
table(A$E8022510)
table(A$E8022511)
table(A$E8022512)

A$I0501<-NA
```

© 2023 Silver IA et al. *JAMA Network Open.*

```
A$I0501[A$E8022501==0]<-0
A$I0501[A$E8022501==99]<-0
A$I0501[A$E8022501==1]<-1
A$I0501[is.na(A$I0501) & A$E8043000==-4]<-0
A$I0501[is.na(A$I0501) & A$E8043000==-3]<-0
A$I0501[is.na(A$I0501) & A$E8043000 >= 200600]<-0
A$I0501[is.na(A$I0501) & A$E8043500==0]<-0
A$I0501[is.na(A$I0501) & A$E8043500==-3]<-0
table(A$E8022501, useNA="always")
table(A$I0501, useNA="always")
A$I0502<-NA
A$I0502[A$E8022502==0]<-0
A$I0502[A$E8022502==99]<-0
A$I0502[A$E8022502==1]<-1
A$I0502[is.na(A$I0502) & A$E8043000==-4]<-0
A$I0502[is.na(A$I0502) & A$E8043000==-3]<-0
A$I0502[is.na(A$I0502) & A$E8043000 >= 200600]<-0
A$I0502[is.na(A$I0502) & A$E8043500==0]<-0
A$I0502[is.na(A$I0502) & A$E8043500==-3]<-0
table(A$E8022502, useNA="always")
table(A$I0502, useNA="always")
A$I0503<-NA
A$I0503[A$E8022503==0]<-0
A$I0503[A$E8022503==99]<-0
A$I0503[A$E8022503==1]<-1
A$I0503[is.na(A$I0503) & A$E8043000==-4]<-0
A$I0503[is.na(A$I0503) & A$E8043000==-3]<-0
A$I0503[is.na(A$I0503) & A$E8043000 >= 200600]<-0
A$I0503[is.na(A$I0503) & A$E8043500==0]<-0
A$I0503[is.na(A$I0503) & A$E8043500==-3]<-0
table(A$E8022503, useNA="always")
table(A$I0503, useNA="always")
A$I0504<-NA
A$I0504[A$E8022504==0]<-0
A$I0504[A$E8022504==99]<-0
A$I0504[A$E8022504==1]<-1
A$I0504[is.na(A$I0504) & A$E8043000==-4]<-0
A$I0504[is.na(A$I0504) & A$E8043000==-3]<-0
A$I0504[is.na(A$I0504) & A$E8043000 >= 200600]<-0
A$I0504[is.na(A$I0504) & A$E8043500==0]<-0
A$I0504[is.na(A$I0504) & A$E8043500==-3]<-0
table(A$E8022504, useNA="always")
table(A$I0504, useNA="always")
A$I0505<-NA
A$I0505[A$E8022505==0]<-0
A$I0505[A$E8022505==99]<-0
A$I0505[A$E8022505==1]<-1
A$I0505[is.na(A$I0505) & A$E8043000==-4]<-0
A$I0505[is.na(A$I0505) & A$E8043000==-3]<-0
A$I0505[is.na(A$I0505) & A$E8043000 >= 200600]<-0
A$I0505[is.na(A$I0505) & A$E8043500==0]<-0
A$I0505[is.na(A$I0505) & A$E8043500==-3]<-0
table(A$E8022505, useNA="always")
table(A$I0505, useNA="always")
A$I0506<-NA
A$I0506[A$E8022506==0]<-0
```

© 2023 Silver IA et al. *JAMA Network Open.*

```
A$I0506[A$E8022506==99]<-0
A$I0506[A$E8022506==1]<-1
A$I0506[is.na(A$I0506) & A$E8043000==-4]<-0
A$I0506[is.na(A$I0506) & A$E8043000==-3]<-0
A$I0506[is.na(A$I0506) & A$E8043000 >= 200600]<-0
A$I0506[is.na(A$I0506) & A$E8043500==0]<-0
A$I0506[is.na(A$I0506) & A$E8043500==-3]<-0
table(A$E8022506, useNA="always")
table(A$I0506, useNA="always")
A$I0507<-NA
A$I0507[A$E8022507==0]<-0
A$I0507[A$E8022507==99]<-0
A$I0507[A$E8022507==1]<-1
A$I0507[is.na(A$I0507) & A$E8043000==-4]<-0
A$I0507[is.na(A$I0507) & A$E8043000==-3]<-0
A$I0507[is.na(A$I0507) & A$E8043000 >= 200600]<-0
A$I0507[is.na(A$I0507) & A$E8043500==0]<-0
A$I0507[is.na(A$I0507) & A$E8043500==-3]<-0
table(A$E8022507, useNA="always")
table(A$I0507, useNA="always")
A$I0508<-NA
A$I0508[A$E8022508==0]<-0
A$I0508[A$E8022508==99]<-0
A$I0508[A$E8022508==1]<-1
A$I0508[is.na(A$I0508) & A$E8043000==-4]<-0
A$I0508[is.na(A$I0508) & A$E8043000==-3]<-0
A$I0508[is.na(A$I0508) & A$E8043000 >= 200600]<-0
A$I0508[is.na(A$I0508) & A$E8043500==0]<-0
A$I0508[is.na(A$I0508) & A$E8043500==-3]<-0
table(A$E8022508, useNA="always")
table(A$I0508, useNA="always")
A$I0509<-NA
A$I0509[A$E8022509==0]<-0
A$I0509[A$E8022509==99]<-0
A$I0509[A$E8022509==1]<-1
A$I0509[is.na(A$I0509) & A$E8043000==-4]<-0
A$I0509[is.na(A$I0509) & A$E8043000==-3]<-0
A$I0509[is.na(A$I0509) & A$E8043000 >= 200600]<-0
A$I0509[is.na(A$I0509) & A$E8043500==0]<-0
A$I0509[is.na(A$I0509) & A$E8043500==-3]<-0
table(A$E8022509, useNA="always")
table(A$I0509, useNA="always")
A$I0510<-NA
A$I0510[A$E8022510==0]<-0
A$I0510[A$E8022510==99]<-0
A$I0510[A$E8022510==1]<-1
A$I0510[is.na(A$I0510) & A$E8043000==-4]<-0
A$I0510[is.na(A$I0510) & A$E8043000==-3]<-0
A$I0510[is.na(A$I0510) & A$E8043000 >= 200600]<-0
A$I0510[is.na(A$I0510) & A$E8043500==0]<-0
A$I0510[is.na(A$I0510) & A$E8043500==-3]<-0
table(A$E8022510, useNA="always")
table(A$I0510, useNA="always")
A$I0511<-NA
A$I0511[A$E8022511==0]<-0
A$I0511[A$E8022511==99]<-0
```

© 2023 Silver IA et al. *JAMA Network Open.*

```
A$I0511[A$E8022511==1]<-1
A$I0511[is.na(A$I0511) & A$E8043000==-4]<-0
A$I0511[is.na(A$I0511) & A$E8043000==-3]<-0
A$I0511[is.na(A$I0511) & A$E8043000 >= 200600]<-0
A$I0511[is.na(A$I0511) & A$E8043500==0]<-0
A$I0511[is.na(A$I0511) & A$E8043500==-3]<-0
table(A$E8022511, useNA="always")
table(A$I0511, useNA="always")
A$I0512<-NA
A$I0512[A$E8022512==0]<-0
A$I0512[A$E8022512==99]<-0
A$I0512[A$E8022512==1]<-1
A$I0512[is.na(A$I0512) & A$E8043000==-4]<-0
A$I0512[is.na(A$I0512) & A$E8043000==-3]<-0
A$I0512[is.na(A$I0512) & A$E8043000 >= 200600]<-0
A$I0512[is.na(A$I0512) & A$E8043500==0]<-0
A$I0512[is.na(A$I0512) & A$E8043500==-3]<-0
table(A$E8022512, useNA="always")
table(A$I0512, useNA="always")




A$I05<-
rowSums(data.frame(A$I0501,A$I0502,A$I0503,A$I0504,A$I0505,A$I0506,A$I0507,A$
I0508,A$I0509,A$I0510,A$I0511,A$I0512), na.rm = T)
A$I05[is.na(A$I05)]<-0
table(A$I05, useNA="always")

### Incarceration (2006)

table(A$E8022601, useNA="always")
table(A$E8022602)
table(A$E8022603)
table(A$E8022604)
table(A$E8022605)
table(A$E8022606)
table(A$E8022607)
table(A$E8022608)
table(A$E8022609)
table(A$E8022610)
table(A$E8022611)
table(A$E8022612)

A$I0601<-NA
A$I0601[A$E8022601==0]<-0
A$I0601[A$E8022601==99]<-0
A$I0601[A$E8022601==1]<-1
A$I0601[is.na(A$I0601) & A$E8043000==-4]<-0
A$I0601[is.na(A$I0601) & A$E8043000==-3]<-0
A$I0601[is.na(A$I0601) & A$E8043000 >= 200700]<-0
A$I0601[is.na(A$I0601) & A$E8043500==0]<-0
A$I0601[is.na(A$I0601) & A$E8043500==-3]<-0
table(A$E8022601, useNA="always")
table(A$I0601, useNA="always")
A$I0602<-NA
```

```
A$I0602[A$E8022602==0]<-0
A$I0602[A$E8022602==99]<-0
A$I0602[A$E8022602==1]<-1
A$I0602[is.na(A$I0602) & A$E8043000==-4]<-0
A$I0602[is.na(A$I0602) & A$E8043000==-3]<-0
A$I0602[is.na(A$I0602) & A$E8043000 >= 200700]<-0
A$I0602[is.na(A$I0602) & A$E8043500==0]<-0
A$I0602[is.na(A$I0602) & A$E8043500==-3]<-0
table(A$E8022602, useNA="always")
table(A$I0602, useNA="always")
A$I0603<-NA
A$I0603[A$E8022603==0]<-0
A$I0603[A$E8022603==99]<-0
A$I0603[A$E8022603==1]<-1
A$I0603[is.na(A$I0603) & A$E8043000==-4]<-0
A$I0603[is.na(A$I0603) & A$E8043000==-3]<-0
A$I0603[is.na(A$I0603) & A$E8043000 >= 200700]<-0
A$I0603[is.na(A$I0603) & A$E8043500==0]<-0
A$I0603[is.na(A$I0603) & A$E8043500==-3]<-0
table(A$E8022603, useNA="always")
table(A$I0603, useNA="always")
A$I0604<-NA
A$I0604[A$E8022604==0]<-0
A$I0604[A$E8022604==99]<-0
A$I0604[A$E8022604==1]<-1
A$I0604[is.na(A$I0604) & A$E8043000==-4]<-0
A$I0604[is.na(A$I0604) & A$E8043000==-3]<-0
A$I0604[is.na(A$I0604) & A$E8043000 >= 200700]<-0
A$I0604[is.na(A$I0604) & A$E8043500==0]<-0
A$I0604[is.na(A$I0604) & A$E8043500==-3]<-0
table(A$E8022604, useNA="always")
table(A$I0604, useNA="always")
A$I0605<-NA
A$I0605[A$E8022605==0]<-0
A$I0605[A$E8022605==99]<-0
A$I0605[A$E8022605==1]<-1
A$I0605[is.na(A$I0605) & A$E8043000==-4]<-0
A$I0605[is.na(A$I0605) & A$E8043000==-3]<-0
A$I0605[is.na(A$I0605) & A$E8043000 >= 200700]<-0
A$I0605[is.na(A$I0605) & A$E8043500==0]<-0
A$I0605[is.na(A$I0605) & A$E8043500==-3]<-0
table(A$E8022605, useNA="always")
table(A$I0605, useNA="always")
A$I0606<-NA
A$I0606[A$E8022606==0]<-0
A$I0606[A$E8022606==99]<-0
A$I0606[A$E8022606==1]<-1
A$I0606[is.na(A$I0606) & A$E8043000==-4]<-0
A$I0606[is.na(A$I0606) & A$E8043000==-3]<-0
A$I0606[is.na(A$I0606) & A$E8043000 >= 200700]<-0
A$I0606[is.na(A$I0606) & A$E8043500==0]<-0
A$I0606[is.na(A$I0606) & A$E8043500==-3]<-0
table(A$E8022606, useNA="always")
table(A$I0606, useNA="always")
A$I0607<-NA
A$I0607[A$E8022607==0]<-0
```

© 2023 Silver IA et al. *JAMA Network Open.*

```
A$I0607[A$E8022607==99]<-0
A$I0607[A$E8022607==1]<-1
A$I0607[is.na(A$I0607) & A$E8043000==-4]<-0
A$I0607[is.na(A$I0607) & A$E8043000==-3]<-0
A$I0607[is.na(A$I0607) & A$E8043000 >= 200700]<-0
A$I0607[is.na(A$I0607) & A$E8043500==0]<-0
A$I0607[is.na(A$I0607) & A$E8043500==-3]<-0
table(A$E8022607, useNA="always")
table(A$I0607, useNA="always")
A$I0608<-NA
A$I0608[A$E8022608==0]<-0
A$I0608[A$E8022608==99]<-0
A$I0608[A$E8022608==1]<-1
A$I0608[is.na(A$I0608) & A$E8043000==-4]<-0
A$I0608[is.na(A$I0608) & A$E8043000==-3]<-0
A$I0608[is.na(A$I0608) & A$E8043000 >= 200700]<-0
A$I0608[is.na(A$I0608) & A$E8043500==0]<-0
A$I0608[is.na(A$I0608) & A$E8043500==-3]<-0
table(A$E8022608, useNA="always")
table(A$I0608, useNA="always")
A$I0609<-NA
A$I0609[A$E8022609==0]<-0
A$I0609[A$E8022609==99]<-0
A$I0609[A$E8022609==1]<-1
A$I0609[is.na(A$I0609) & A$E8043000==-4]<-0
A$I0609[is.na(A$I0609) & A$E8043000==-3]<-0
A$I0609[is.na(A$I0609) & A$E8043000 >= 200700]<-0
A$I0609[is.na(A$I0609) & A$E8043500==0]<-0
A$I0609[is.na(A$I0609) & A$E8043500==-3]<-0
table(A$E8022609, useNA="always")
table(A$I0609, useNA="always")
A$I0610<-NA
A$I0610[A$E8022610==0]<-0
A$I0610[A$E8022610==99]<-0
A$I0610[A$E8022610==1]<-1
A$I0610[is.na(A$I0610) & A$E8043000==-4]<-0
A$I0610[is.na(A$I0610) & A$E8043000==-3]<-0
A$I0610[is.na(A$I0610) & A$E8043000 >= 200700]<-0
A$I0610[is.na(A$I0610) & A$E8043500==0]<-0
A$I0610[is.na(A$I0610) & A$E8043500==-3]<-0
table(A$E8022610, useNA="always")
table(A$I0610, useNA="always")
A$I0611<-NA
A$I0611[A$E8022611==0]<-0
A$I0611[A$E8022611==99]<-0
A$I0611[A$E8022611==1]<-1
A$I0611[is.na(A$I0611) & A$E8043000==-4]<-0
A$I0611[is.na(A$I0611) & A$E8043000==-3]<-0
A$I0611[is.na(A$I0611) & A$E8043000 >= 200700]<-0
A$I0611[is.na(A$I0611) & A$E8043500==0]<-0
A$I0611[is.na(A$I0611) & A$E8043500==-3]<-0
table(A$E8022611, useNA="always")
table(A$I0611, useNA="always")
A$I0612<-NA
A$I0612[A$E8022612==0]<-0
A$I0612[A$E8022612==99]<-0
```

© 2023 Silver IA et al. *JAMA Network Open.*

```
A$I0612[A$E8022612==1]<-1
A$I0612[is.na(A$I0612) & A$E8043000==-4]<-0
A$I0612[is.na(A$I0612) & A$E8043000==-3]<-0
A$I0612[is.na(A$I0612) & A$E8043000 >= 200700]<-0
A$I0612[is.na(A$I0612) & A$E8043500==0]<-0
A$I0612[is.na(A$I0612) & A$E8043500==-3]<-0
table(A$E8022612, useNA="always")
table(A$I0612, useNA="always")




A$I06<-
rowSums(data.frame(A$I0601,A$I0602,A$I0603,A$I0604,A$I0605,A$I0606,A$I0607,A$
I0608,A$I0609,A$I0610,A$I0611,A$I0612), na.rm = T)
A$I06[is.na(A$I06)]<-0
table(A$I06, useNA="always")

### Incarceration (2007)

table(A$E8022701, useNA="always")
table(A$E8022702)
table(A$E8022703)
table(A$E8022704)
table(A$E8022705)
table(A$E8022706)
table(A$E8022707)
table(A$E8022708)
table(A$E8022709)
table(A$E8022710)
table(A$E8022711)
table(A$E8022712)

A$I0701<-NA
A$I0701[A$E8022701==0]<-0
A$I0701[A$E8022701==99]<-0
A$I0701[A$E8022701==1]<-1
A$I0701[is.na(A$I0701) & A$E8043000==-4]<-0
A$I0701[is.na(A$I0701) & A$E8043000==-3]<-0
A$I0701[is.na(A$I0701) & A$E8043000 >= 200800]<-0
A$I0701[is.na(A$I0701) & A$E8043500==0]<-0
A$I0701[is.na(A$I0701) & A$E8043500==-3]<-0
table(A$E8022701, useNA="always")
table(A$I0701, useNA="always")
A$I0702<-NA
A$I0702[A$E8022702==0]<-0
A$I0702[A$E8022702==99]<-0
A$I0702[A$E8022702==1]<-1
A$I0702[is.na(A$I0702) & A$E8043000==-4]<-0
A$I0702[is.na(A$I0702) & A$E8043000==-3]<-0
A$I0702[is.na(A$I0702) & A$E8043000 >= 200800]<-0
A$I0702[is.na(A$I0702) & A$E8043500==0]<-0
A$I0702[is.na(A$I0702) & A$E8043500==-3]<-0
table(A$E8022702, useNA="always")
table(A$I0702, useNA="always")
A$I0703<-NA
```

© 2023 Silver IA et al. *JAMA Network Open.*

```
A$I0703[A$E8022703==0]<-0
A$I0703[A$E8022703==99]<-0
A$I0703[A$E8022703==1]<-1
A$I0703[is.na(A$I0703) & A$E8043000==-4]<-0
A$I0703[is.na(A$I0703) & A$E8043000==-3]<-0
A$I0703[is.na(A$I0703) & A$E8043000 >= 200800]<-0
A$I0703[is.na(A$I0703) & A$E8043500==0]<-0
A$I0703[is.na(A$I0703) & A$E8043500==-3]<-0
table(A$E8022703, useNA="always")
table(A$I0703, useNA="always")
A$I0704<-NA
A$I0704[A$E8022704==0]<-0
A$I0704[A$E8022704==99]<-0
A$I0704[A$E8022704==1]<-1
A$I0704[is.na(A$I0704) & A$E8043000==-4]<-0
A$I0704[is.na(A$I0704) & A$E8043000==-3]<-0
A$I0704[is.na(A$I0704) & A$E8043000 >= 200800]<-0
A$I0704[is.na(A$I0704) & A$E8043500==0]<-0
A$I0704[is.na(A$I0704) & A$E8043500==-3]<-0
table(A$E8022704, useNA="always")
table(A$I0704, useNA="always")
A$I0705<-NA
A$I0705[A$E8022705==0]<-0
A$I0705[A$E8022705==99]<-0
A$I0705[A$E8022705==1]<-1
A$I0705[is.na(A$I0705) & A$E8043000==-4]<-0
A$I0705[is.na(A$I0705) & A$E8043000==-3]<-0
A$I0705[is.na(A$I0705) & A$E8043000 >= 200800]<-0
A$I0705[is.na(A$I0705) & A$E8043500==0]<-0
A$I0705[is.na(A$I0705) & A$E8043500==-3]<-0
table(A$E8022705, useNA="always")
table(A$I0705, useNA="always")
A$I0706<-NA
A$I0706[A$E8022706==0]<-0
A$I0706[A$E8022706==99]<-0
A$I0706[A$E8022706==1]<-1
A$I0706[is.na(A$I0706) & A$E8043000==-4]<-0
A$I0706[is.na(A$I0706) & A$E8043000==-3]<-0
A$I0706[is.na(A$I0706) & A$E8043000 >= 200800]<-0
A$I0706[is.na(A$I0706) & A$E8043500==0]<-0
A$I0706[is.na(A$I0706) & A$E8043500==-3]<-0
table(A$E8022706, useNA="always")
table(A$I0706, useNA="always")
A$I0707<-NA
A$I0707[A$E8022707==0]<-0
A$I0707[A$E8022707==99]<-0
A$I0707[A$E8022707==1]<-1
A$I0707[is.na(A$I0707) & A$E8043000==-4]<-0
A$I0707[is.na(A$I0707) & A$E8043000==-3]<-0
A$I0707[is.na(A$I0707) & A$E8043000 >= 200800]<-0
A$I0707[is.na(A$I0707) & A$E8043500==0]<-0
A$I0707[is.na(A$I0707) & A$E8043500==-3]<-0
table(A$E8022707, useNA="always")
table(A$I0707, useNA="always")
A$I0708<-NA
A$I0708[A$E8022708==0]<-0
```

© 2023 Silver IA et al. *JAMA Network Open.*

```
A$I0708[A$E8022708==99]<-0
A$I0708[A$E8022708==1]<-1
A$I0708[is.na(A$I0708) & A$E8043000==-4]<-0
A$I0708[is.na(A$I0708) & A$E8043000==-3]<-0
A$I0708[is.na(A$I0708) & A$E8043000 >= 200800]<-0
A$I0708[is.na(A$I0708) & A$E8043500==0]<-0
A$I0708[is.na(A$I0708) & A$E8043500==-3]<-0
table(A$E8022708, useNA="always")
table(A$I0708, useNA="always")
A$I0709<-NA
A$I0709[A$E8022709==0]<-0
A$I0709[A$E8022709==99]<-0
A$I0709[A$E8022709==1]<-1
A$I0709[is.na(A$I0709) & A$E8043000==-4]<-0
A$I0709[is.na(A$I0709) & A$E8043000==-3]<-0
A$I0709[is.na(A$I0709) & A$E8043000 >= 200800]<-0
A$I0709[is.na(A$I0709) & A$E8043500==0]<-0
A$I0709[is.na(A$I0709) & A$E8043500==-3]<-0
table(A$E8022709, useNA="always")
table(A$I0709, useNA="always")
A$I0710<-NA
A$I0710[A$E8022710==0]<-0
A$I0710[A$E8022710==99]<-0
A$I0710[A$E8022710==1]<-1
A$I0710[is.na(A$I0710) & A$E8043000==-4]<-0
A$I0710[is.na(A$I0710) & A$E8043000==-3]<-0
A$I0710[is.na(A$I0710) & A$E8043000 >= 200800]<-0
A$I0710[is.na(A$I0710) & A$E8043500==0]<-0
A$I0710[is.na(A$I0710) & A$E8043500==-3]<-0
table(A$E8022710, useNA="always")
table(A$I0710, useNA="always")
A$I0711<-NA
A$I0711[A$E8022711==0]<-0
A$I0711[A$E8022711==99]<-0
A$I0711[A$E8022711==1]<-1
A$I0711[is.na(A$I0711) & A$E8043000==-4]<-0
A$I0711[is.na(A$I0711) & A$E8043000==-3]<-0
A$I0711[is.na(A$I0711) & A$E8043000 >= 200800]<-0
A$I0711[is.na(A$I0711) & A$E8043500==0]<-0
A$I0711[is.na(A$I0711) & A$E8043500==-3]<-0
table(A$E8022711, useNA="always")
table(A$I0711, useNA="always")
A$I0712<-NA
A$I0712[A$E8022712==0]<-0
A$I0712[A$E8022712==99]<-0
A$I0712[A$E8022712==1]<-1
A$I0712[is.na(A$I0712) & A$E8043000==-4]<-0
A$I0712[is.na(A$I0712) & A$E8043000==-3]<-0
A$I0712[is.na(A$I0712) & A$E8043000 >= 200800]<-0
A$I0712[is.na(A$I0712) & A$E8043500==0]<-0
A$I0712[is.na(A$I0712) & A$E8043500==-3]<-0
table(A$E8022712, useNA="always")
table(A$I0712, useNA="always")
```

© 2023 Silver IA et al. *JAMA Network Open.*

```
A$I07<-
rowSums(data.frame(A$I0701,A$I0702,A$I0703,A$I0704,A$I0705,A$I0706,A$I0707,A$
I0708,A$I0709,A$I0710,A$I0711,A$I0712), na.rm = T)
A$I07[is.na(A$I07) & A$E8043500 == 0]<-0
table(A$I07, useNA="always")

### Incarceration (2008)

table(A$E8022801, useNA="always")
table(A$E8022802)
table(A$E8022803)
table(A$E8022804)
table(A$E8022805)
table(A$E8022806)
table(A$E8022807)
table(A$E8022808)
table(A$E8022809)
table(A$E8022810)
table(A$E8022811)
table(A$E8022812)

A$I0801<-NA
A$I0801[A$E8022801==0]<-0
A$I0801[A$E8022801==99]<-0
A$I0801[A$E8022801==1]<-1
A$I0801[is.na(A$I0801) & A$E8043000==-4]<-0
A$I0801[is.na(A$I0801) & A$E8043000==-3]<-0
A$I0801[is.na(A$I0801) & A$E8043000 >= 200900]<-0
A$I0801[is.na(A$I0801) & A$E8043500==0]<-0
A$I0801[is.na(A$I0801) & A$E8043500==-3]<-0
table(A$E8022801, useNA="always")
table(A$I0801, useNA="always")
A$I0802<-NA
A$I0802[A$E8022802==0]<-0
A$I0802[A$E8022802==99]<-0
A$I0802[A$E8022802==1]<-1
A$I0802[is.na(A$I0802) & A$E8043000==-4]<-0
A$I0802[is.na(A$I0802) & A$E8043000==-3]<-0
A$I0802[is.na(A$I0802) & A$E8043000 >= 200900]<-0
A$I0802[is.na(A$I0802) & A$E8043500==0]<-0
A$I0802[is.na(A$I0802) & A$E8043500==-3]<-0
table(A$E8022802, useNA="always")
table(A$I0802, useNA="always")
A$I0803<-NA
A$I0803[A$E8022803==0]<-0
A$I0803[A$E8022803==99]<-0
A$I0803[A$E8022803==1]<-1
A$I0803[is.na(A$I0803) & A$E8043000==-4]<-0
A$I0803[is.na(A$I0803) & A$E8043000==-3]<-0
A$I0803[is.na(A$I0803) & A$E8043000 >= 200900]<-0
A$I0803[is.na(A$I0803) & A$E8043500==0]<-0
A$I0803[is.na(A$I0803) & A$E8043500==-3]<-0
table(A$E8022803, useNA="always")
table(A$I0803, useNA="always")
A$I0804<-NA
```

```
A$I0804[A$E8022804==0]<-0
A$I0804[A$E8022804==99]<-0
A$I0804[A$E8022804==1]<-1
A$I0804[is.na(A$I0804) & A$E8043000==-4]<-0
A$I0804[is.na(A$I0804) & A$E8043000==-3]<-0
A$I0804[is.na(A$I0804) & A$E8043000 >= 200900]<-0
A$I0804[is.na(A$I0804) & A$E8043500==0]<-0
A$I0804[is.na(A$I0804) & A$E8043500==-3]<-0
table(A$E8022804, useNA="always")
table(A$I0804, useNA="always")
A$I0805<-NA
A$I0805[A$E8022805==0]<-0
A$I0805[A$E8022805==99]<-0
A$I0805[A$E8022805==1]<-1
A$I0805[is.na(A$I0805) & A$E8043000==-4]<-0
A$I0805[is.na(A$I0805) & A$E8043000==-3]<-0
A$I0805[is.na(A$I0805) & A$E8043000 >= 200900]<-0
A$I0805[is.na(A$I0805) & A$E8043500==0]<-0
A$I0805[is.na(A$I0805) & A$E8043500==-3]<-0
table(A$E8022805, useNA="always")
table(A$I0805, useNA="always")
A$I0806<-NA
A$I0806[A$E8022806==0]<-0
A$I0806[A$E8022806==99]<-0
A$I0806[A$E8022806==1]<-1
A$I0806[is.na(A$I0806) & A$E8043000==-4]<-0
A$I0806[is.na(A$I0806) & A$E8043000==-3]<-0
A$I0806[is.na(A$I0806) & A$E8043000 >= 200900]<-0
A$I0806[is.na(A$I0806) & A$E8043500==0]<-0
A$I0806[is.na(A$I0806) & A$E8043500==-3]<-0
table(A$E8022806, useNA="always")
table(A$I0806, useNA="always")
A$I0807<-NA
A$I0807[A$E8022807==0]<-0
A$I0807[A$E8022807==99]<-0
A$I0807[A$E8022807==1]<-1
A$I0807[is.na(A$I0807) & A$E8043000==-4]<-0
A$I0807[is.na(A$I0807) & A$E8043000==-3]<-0
A$I0807[is.na(A$I0807) & A$E8043000 >= 200900]<-0
A$I0807[is.na(A$I0807) & A$E8043500==0]<-0
A$I0807[is.na(A$I0807) & A$E8043500==-3]<-0
table(A$E8022807, useNA="always")
table(A$I0807, useNA="always")
A$I0808<-NA
A$I0808[A$E8022808==0]<-0
A$I0808[A$E8022808==99]<-0
A$I0808[A$E8022808==1]<-1
A$I0808[is.na(A$I0808) & A$E8043000==-4]<-0
A$I0808[is.na(A$I0808) & A$E8043000==-3]<-0
A$I0808[is.na(A$I0808) & A$E8043000 >= 200900]<-0
A$I0808[is.na(A$I0808) & A$E8043500==0]<-0
A$I0808[is.na(A$I0808) & A$E8043500==-3]<-0
table(A$E8022808, useNA="always")
table(A$I0808, useNA="always")
A$I0809<-NA
A$I0809[A$E8022809==0]<-0
```

© 2023 Silver IA et al. *JAMA Network Open.*

```
A$I0809[A$E8022809==99]<-0
A$I0809[A$E8022809==1]<-1
A$I0809[is.na(A$I0809) & A$E8043000==-4]<-0
A$I0809[is.na(A$I0809) & A$E8043000==-3]<-0
A$I0809[is.na(A$I0809) & A$E8043000 >= 200900]<-0
A$I0809[is.na(A$I0809) & A$E8043500==0]<-0
A$I0809[is.na(A$I0809) & A$E8043500==-3]<-0
table(A$E8022809, useNA="always")
table(A$I0809, useNA="always")
A$I0810<-NA
A$I0810[A$E8022810==0]<-0
A$I0810[A$E8022810==99]<-0
A$I0810[A$E8022810==1]<-1
A$I0810[is.na(A$I0810) & A$E8043000==-4]<-0
A$I0810[is.na(A$I0810) & A$E8043000==-3]<-0
A$I0810[is.na(A$I0810) & A$E8043000 >= 200900]<-0
A$I0810[is.na(A$I0810) & A$E8043500==0]<-0
A$I0810[is.na(A$I0810) & A$E8043500==-3]<-0
table(A$E8022810, useNA="always")
table(A$I0810, useNA="always")
A$I0811<-NA
A$I0811[A$E8022811==0]<-0
A$I0811[A$E8022811==99]<-0
A$I0811[A$E8022811==1]<-1
A$I0811[is.na(A$I0811) & A$E8043000==-4]<-0
A$I0811[is.na(A$I0811) & A$E8043000==-3]<-0
A$I0811[is.na(A$I0811) & A$E8043000 >= 200900]<-0
A$I0811[is.na(A$I0811) & A$E8043500==0]<-0
A$I0811[is.na(A$I0811) & A$E8043500==-3]<-0
table(A$E8022811, useNA="always")
table(A$I0811, useNA="always")
A$I0812<-NA
A$I0812[A$E8022812==0]<-0
A$I0812[A$E8022812==99]<-0
A$I0812[A$E8022812==1]<-1
A$I0812[is.na(A$I0812) & A$E8043000==-4]<-0
A$I0812[is.na(A$I0812) & A$E8043000==-3]<-0
A$I0812[is.na(A$I0812) & A$E8043000 >= 200900]<-0
A$I0812[is.na(A$I0812) & A$E8043500==0]<-0
A$I0812[is.na(A$I0812) & A$E8043500==-3]<-0
table(A$E8022812, useNA="always")
table(A$I0812, useNA="always")




A$I08<-
rowSums(data.frame(A$I0801,A$I0802,A$I0803,A$I0804,A$I0805,A$I0806,A$I0807,A$
I0808,A$I0809,A$I0810,A$I0811,A$I0812), na.rm = T)
A$I08[is.na(A$I08)]<-0
table(A$I08, useNA="always")

### Incarceration (2009)

table(A$E8022901, useNA="always")
table(A$E8022902)
```

© 2023 Silver IA et al. *JAMA Network Open.*

```
table(A$E8022903)
table(A$E8022904)
table(A$E8022905)
table(A$E8022906)
table(A$E8022907)
table(A$E8022908)
table(A$E8022909)
table(A$E8022910)
table(A$E8022911)
table(A$E8022912)

A$I0901<-NA
A$I0901[A$E8022901==0]<-0
A$I0901[A$E8022901==99]<-0
A$I0901[A$E8022901==1]<-1
A$I0901[is.na(A$I0901) & A$E8043000==-4]<-0
A$I0901[is.na(A$I0901) & A$E8043000==-3]<-0
A$I0901[is.na(A$I0901) & A$E8043000 >= 201000]<-0
A$I0901[is.na(A$I0901) & A$E8043500==0]<-0
A$I0901[is.na(A$I0901) & A$E8043500==-3]<-0
table(A$E8022901, useNA="always")
table(A$I0901, useNA="always")
A$I0902<-NA
A$I0902[A$E8022902==0]<-0
A$I0902[A$E8022902==99]<-0
A$I0902[A$E8022902==1]<-1
A$I0902[is.na(A$I0902) & A$E8043000==-4]<-0
A$I0902[is.na(A$I0902) & A$E8043000==-3]<-0
A$I0902[is.na(A$I0902) & A$E8043000 >= 201000]<-0
A$I0902[is.na(A$I0902) & A$E8043500==0]<-0
A$I0902[is.na(A$I0902) & A$E8043500==-3]<-0
table(A$E8022902, useNA="always")
table(A$I0902, useNA="always")
A$I0903<-NA
A$I0903[A$E8022903==0]<-0
A$I0903[A$E8022903==99]<-0
A$I0903[A$E8022903==1]<-1
A$I0903[is.na(A$I0903) & A$E8043000==-4]<-0
A$I0903[is.na(A$I0903) & A$E8043000==-3]<-0
A$I0903[is.na(A$I0903) & A$E8043000 >= 201000]<-0
A$I0903[is.na(A$I0903) & A$E8043500==0]<-0
A$I0903[is.na(A$I0903) & A$E8043500==-3]<-0
table(A$E8022903, useNA="always")
table(A$I0903, useNA="always")
A$I0904<-NA
A$I0904[A$E8022904==0]<-0
A$I0904[A$E8022904==99]<-0
A$I0904[A$E8022904==1]<-1
A$I0904[is.na(A$I0904) & A$E8043000==-4]<-0
A$I0904[is.na(A$I0904) & A$E8043000==-3]<-0
A$I0904[is.na(A$I0904) & A$E8043000 >= 201000]<-0
A$I0904[is.na(A$I0904) & A$E8043500==0]<-0
A$I0904[is.na(A$I0904) & A$E8043500==-3]<-0
table(A$E8022904, useNA="always")
table(A$I0904, useNA="always")
A$I0905<-NA
```

© 2023 Silver IA et al. *JAMA Network Open.*

```
A$I0905[A$E8022905==0]<-0
A$I0905[A$E8022905==99]<-0
A$I0905[A$E8022905==1]<-1
A$I0905[is.na(A$I0905) & A$E8043000==-4]<-0
A$I0905[is.na(A$I0905) & A$E8043000==-3]<-0
A$I0905[is.na(A$I0905) & A$E8043000 >= 201000]<-0
A$I0905[is.na(A$I0905) & A$E8043500==0]<-0
A$I0905[is.na(A$I0905) & A$E8043500==-3]<-0
table(A$E8022905, useNA="always")
table(A$I0905, useNA="always")
A$I0906<-NA
A$I0906[A$E8022906==0]<-0
A$I0906[A$E8022906==99]<-0
A$I0906[A$E8022906==1]<-1
A$I0906[is.na(A$I0906) & A$E8043000==-4]<-0
A$I0906[is.na(A$I0906) & A$E8043000==-3]<-0
A$I0906[is.na(A$I0906) & A$E8043000 >= 201000]<-0
A$I0906[is.na(A$I0906) & A$E8043500==0]<-0
A$I0906[is.na(A$I0906) & A$E8043500==-3]<-0
table(A$E8022906, useNA="always")
table(A$I0906, useNA="always")
A$I0907<-NA
A$I0907[A$E8022907==0]<-0
A$I0907[A$E8022907==99]<-0
A$I0907[A$E8022907==1]<-1
A$I0907[is.na(A$I0907) & A$E8043000==-4]<-0
A$I0907[is.na(A$I0907) & A$E8043000==-3]<-0
A$I0907[is.na(A$I0907) & A$E8043000 >= 201000]<-0
A$I0907[is.na(A$I0907) & A$E8043500==0]<-0
A$I0907[is.na(A$I0907) & A$E8043500==-3]<-0
table(A$E8022907, useNA="always")
table(A$I0907, useNA="always")
A$I0908<-NA
A$I0908[A$E8022908==0]<-0
A$I0908[A$E8022908==99]<-0
A$I0908[A$E8022908==1]<-1
A$I0908[is.na(A$I0908) & A$E8043000==-4]<-0
A$I0908[is.na(A$I0908) & A$E8043000==-3]<-0
A$I0908[is.na(A$I0908) & A$E8043000 >= 201000]<-0
A$I0908[is.na(A$I0908) & A$E8043500==0]<-0
A$I0908[is.na(A$I0908) & A$E8043500==-3]<-0
table(A$E8022908, useNA="always")
table(A$I0908, useNA="always")
A$I0909<-NA
A$I0909[A$E8022909==0]<-0
A$I0909[A$E8022909==99]<-0
A$I0909[A$E8022909==1]<-1
A$I0909[is.na(A$I0909) & A$E8043000==-4]<-0
A$I0909[is.na(A$I0909) & A$E8043000==-3]<-0
A$I0909[is.na(A$I0909) & A$E8043000 >= 201000]<-0
A$I0909[is.na(A$I0909) & A$E8043500==0]<-0
A$I0909[is.na(A$I0909) & A$E8043500==-3]<-0
table(A$E8022909, useNA="always")
table(A$I0909, useNA="always")
A$I0910<-NA
A$I0910[A$E8022910==0]<-0
```

© 2023 Silver IA et al. *JAMA Network Open.*

```
A$I0910[A$E8022910==99]<-0
A$I0910[A$E8022910==1]<-1
A$I0910[is.na(A$I0910) & A$E8043000==-4]<-0
A$I0910[is.na(A$I0910) & A$E8043000==-3]<-0
A$I0910[is.na(A$I0910) & A$E8043000 >= 201000]<-0
A$I0910[is.na(A$I0910) & A$E8043500==0]<-0
A$I0910[is.na(A$I0910) & A$E8043500==-3]<-0
table(A$E8022910, useNA="always")
table(A$I0910, useNA="always")
A$I0911<-NA
A$I0911[A$E8022911==0]<-0
A$I0911[A$E8022911==99]<-0
A$I0911[A$E8022911==1]<-1
A$I0911[is.na(A$I0911) & A$E8043000==-4]<-0
A$I0911[is.na(A$I0911) & A$E8043000==-3]<-0
A$I0911[is.na(A$I0911) & A$E8043000 >= 201000]<-0
A$I0911[is.na(A$I0911) & A$E8043500==0]<-0
A$I0911[is.na(A$I0911) & A$E8043500==-3]<-0
table(A$E8022911, useNA="always")
table(A$I0911, useNA="always")
A$I0912<-NA
A$I0912[A$E8022912==0]<-0
A$I0912[A$E8022912==99]<-0
A$I0912[A$E8022912==1]<-1
A$I0912[is.na(A$I0912) & A$E8043000==-4]<-0
A$I0912[is.na(A$I0912) & A$E8043000==-3]<-0
A$I0912[is.na(A$I0912) & A$E8043000 >= 201000]<-0
A$I0912[is.na(A$I0912) & A$E8043500==0]<-0
A$I0912[is.na(A$I0912) & A$E8043500==-3]<-0
table(A$E8022912, useNA="always")
table(A$I0912, useNA="always")



A$I09<-
rowSums(data.frame(A$I0901,A$I0902,A$I0903,A$I0904,A$I0905,A$I0906,A$I0907,A$
I0908,A$I0909,A$I0910,A$I0911,A$I0912), na.rm = T)
A$I09[is.na(A$I09)]<-0
table(A$I09, useNA="always")

### Incarceration (2010)

table(A$E8023001, useNA="always")
table(A$E8023002)
table(A$E8023003)
table(A$E8023004)
table(A$E8023005)
table(A$E8023006)
table(A$E8023007)
table(A$E8023008)
table(A$E8023009)
table(A$E8023010)
table(A$E8023011)
table(A$E8023012)
```

© 2023 Silver IA et al. *JAMA Network Open.*

```
A$I1001<-NA
A$I1001[A$E8023001==0]<-0
A$I1001[A$E8023001==99]<-0
A$I1001[A$E8023001==1]<-1
A$I1001[is.na(A$I1001) & A$E8043000==-4]<-0
A$I1001[is.na(A$I1001) & A$E8043000==-3]<-0
A$I1001[is.na(A$I1001) & A$E8043000 >= 201100]<-0
A$I1001[is.na(A$I1001) & A$E8043500==0]<-0
A$I1001[is.na(A$I1001) & A$E8043500==-3]<-0
table(A$E8023001, useNA="always")
table(A$I1001, useNA="always")
A$I1002<-NA
A$I1002[A$E8023002==0]<-0
A$I1002[A$E8023002==99]<-0
A$I1002[A$E8023002==1]<-1
A$I1002[is.na(A$I1002) & A$E8043000==-4]<-0
A$I1002[is.na(A$I1002) & A$E8043000==-3]<-0
A$I1002[is.na(A$I1002) & A$E8043000 >= 201100]<-0
A$I1002[is.na(A$I1002) & A$E8043500==0]<-0
A$I1002[is.na(A$I1002) & A$E8043500==-3]<-0
table(A$E8023002, useNA="always")
table(A$I1002, useNA="always")
A$I1003<-NA
A$I1003[A$E8023003==0]<-0
A$I1003[A$E8023003==99]<-0
A$I1003[A$E8023003==1]<-1
A$I1003[is.na(A$I1003) & A$E8043000==-4]<-0
A$I1003[is.na(A$I1003) & A$E8043000==-3]<-0
A$I1003[is.na(A$I1003) & A$E8043000 >= 201100]<-0
A$I1003[is.na(A$I1003) & A$E8043500==0]<-0
A$I1003[is.na(A$I1003) & A$E8043500==-3]<-0
table(A$E8023003, useNA="always")
table(A$I1003, useNA="always")
A$I1004<-NA
A$I1004[A$E8023004==0]<-0
A$I1004[A$E8023004==99]<-0
A$I1004[A$E8023004==1]<-1
A$I1004[is.na(A$I1004) & A$E8043000==-4]<-0
A$I1004[is.na(A$I1004) & A$E8043000==-3]<-0
A$I1004[is.na(A$I1004) & A$E8043000 >= 201100]<-0
A$I1004[is.na(A$I1004) & A$E8043500==0]<-0
A$I1004[is.na(A$I1004) & A$E8043500==-3]<-0
table(A$E8023004, useNA="always")
table(A$I1004, useNA="always")
A$I1005<-NA
A$I1005[A$E8023005==0]<-0
A$I1005[A$E8023005==99]<-0
A$I1005[A$E8023005==1]<-1
A$I1005[is.na(A$I1005) & A$E8043000==-4]<-0
A$I1005[is.na(A$I1005) & A$E8043000==-3]<-0
A$I1005[is.na(A$I1005) & A$E8043000 >= 201100]<-0
A$I1005[is.na(A$I1005) & A$E8043500==0]<-0
A$I1005[is.na(A$I1005) & A$E8043500==-3]<-0
table(A$E8023005, useNA="always")
table(A$I1005, useNA="always")
A$I1006<-NA
```

```
A$I1006[A$E8023006==0]<-0
A$I1006[A$E8023006==99]<-0
A$I1006[A$E8023006==1]<-1
A$I1006[is.na(A$I1006) & A$E8043000==-4]<-0
A$I1006[is.na(A$I1006) & A$E8043000==-3]<-0
A$I1006[is.na(A$I1006) & A$E8043000 >= 201100]<-0
A$I1006[is.na(A$I1006) & A$E8043500==0]<-0
A$I1006[is.na(A$I1006) & A$E8043500==-3]<-0
table(A$E8023006, useNA="always")
table(A$I1006, useNA="always")
A$I1007<-NA
A$I1007[A$E8023007==0]<-0
A$I1007[A$E8023007==99]<-0
A$I1007[A$E8023007==1]<-1
A$I1007[is.na(A$I1007) & A$E8043000==-4]<-0
A$I1007[is.na(A$I1007) & A$E8043000==-3]<-0
A$I1007[is.na(A$I1007) & A$E8043000 >= 201100]<-0
A$I1007[is.na(A$I1007) & A$E8043500==0]<-0
A$I1007[is.na(A$I1007) & A$E8043500==-3]<-0
table(A$E8023007, useNA="always")
table(A$I1007, useNA="always")
A$I1008<-NA
A$I1008[A$E8023008==0]<-0
A$I1008[A$E8023008==99]<-0
A$I1008[A$E8023008==1]<-1
A$I1008[is.na(A$I1008) & A$E8043000==-4]<-0
A$I1008[is.na(A$I1008) & A$E8043000==-3]<-0
A$I1008[is.na(A$I1008) & A$E8043000 >= 201100]<-0
A$I1008[is.na(A$I1008) & A$E8043500==0]<-0
A$I1008[is.na(A$I1008) & A$E8043500==-3]<-0
table(A$E8023008, useNA="always")
table(A$I1008, useNA="always")
A$I1009<-NA
A$I1009[A$E8023009==0]<-0
A$I1009[A$E8023009==99]<-0
A$I1009[A$E8023009==1]<-1
A$I1009[is.na(A$I1009) & A$E8043000==-4]<-0
A$I1009[is.na(A$I1009) & A$E8043000==-3]<-0
A$I1009[is.na(A$I1009) & A$E8043000 >= 201100]<-0
A$I1009[is.na(A$I1009) & A$E8043500==0]<-0
A$I1009[is.na(A$I1009) & A$E8043500==-3]<-0
table(A$E8023009, useNA="always")
table(A$I1009, useNA="always")
A$I1010<-NA
A$I1010[A$E8023010==0]<-0
A$I1010[A$E8023010==99]<-0
A$I1010[A$E8023010==1]<-1
A$I1010[is.na(A$I1010) & A$E8043000==-4]<-0
A$I1010[is.na(A$I1010) & A$E8043000==-3]<-0
A$I1010[is.na(A$I1010) & A$E8043000 >= 201100]<-0
A$I1010[is.na(A$I1010) & A$E8043500==0]<-0
A$I1010[is.na(A$I1010) & A$E8043500==-3]<-0
table(A$E8023010, useNA="always")
table(A$I1010, useNA="always")
A$I1011<-NA
A$I1011[A$E8023011==0]<-0
```

© 2023 Silver IA et al. *JAMA Network Open.*

```
A$I1011[A$E8023011==99]<-0
A$I1011[A$E8023011==1]<-1
A$I1011[is.na(A$I1011) & A$E8043000==-4]<-0
A$I1011[is.na(A$I1011) & A$E8043000==-3]<-0
A$I1011[is.na(A$I1011) & A$E8043000 >= 201100]<-0
A$I1011[is.na(A$I1011) & A$E8043500==0]<-0
A$I1011[is.na(A$I1011) & A$E8043500==-3]<-0
table(A$E8023011, useNA="always")
table(A$I1011, useNA="always")
A$I1012<-NA
A$I1012[A$E8023012==0]<-0
A$I1012[A$E8023012==99]<-0
A$I1012[A$E8023012==1]<-1
A$I1012[is.na(A$I1012) & A$E8043000==-4]<-0
A$I1012[is.na(A$I1012) & A$E8043000==-3]<-0
A$I1012[is.na(A$I1012) & A$E8043000 >= 201100]<-0
A$I1012[is.na(A$I1012) & A$E8043500==0]<-0
A$I1012[is.na(A$I1012) & A$E8043500==-3]<-0
table(A$E8023012, useNA="always")
table(A$I1012, useNA="always")




A$I10<-
rowSums(data.frame(A$I1001,A$I1002,A$I1003,A$I1004,A$I1005,A$I1006,A$I1007,A$
I1008,A$I1009,A$I1010,A$I1011,A$I1012), na.rm = T)
A$I10[is.na(A$I10)]<-0
table(A$I10, useNA="always")

### Incarceration (2011)

table(A$E8023101, useNA="always")
table(A$E8023102)
table(A$E8023103)
table(A$E8023104)
table(A$E8023105)
table(A$E8023106)
table(A$E8023107)
table(A$E8023108)
table(A$E8023109)
table(A$E8023110)
table(A$E8023111)
table(A$E8023112)

A$I1101<-NA
A$I1101[A$E8023101==0]<-0
A$I1101[A$E8023101==99]<-0
A$I1101[A$E8023101==1]<-1
A$I1101[is.na(A$I1101) & A$E8043000==-4]<-0
A$I1101[is.na(A$I1101) & A$E8043000==-3]<-0
A$I1101[is.na(A$I1101) & A$E8043000 >= 201200]<-0
A$I1101[is.na(A$I1101) & A$E8043500==0]<-0
A$I1101[is.na(A$I1101) & A$E8043500==-3]<-0
table(A$E8023101, useNA="always")
table(A$I1101, useNA="always")
```

© 2023 Silver IA et al. *JAMA Network Open.*

```
A$I1102<-NA
A$I1102[A$E8023102==0]<-0
A$I1102[A$E8023102==99]<-0
A$I1102[A$E8023102==1]<-1
A$I1102[is.na(A$I1102) & A$E8043000==-4]<-0
A$I1102[is.na(A$I1102) & A$E8043000==-3]<-0
A$I1102[is.na(A$I1102) & A$E8043000 >= 201200]<-0
A$I1102[is.na(A$I1102) & A$E8043500==0]<-0
A$I1102[is.na(A$I1102) & A$E8043500==-3]<-0
table(A$E8023102, useNA="always")
table(A$I1102, useNA="always")
A$I1103<-NA
A$I1103[A$E8023103==0]<-0
A$I1103[A$E8023103==99]<-0
A$I1103[A$E8023103==1]<-1
A$I1103[is.na(A$I1103) & A$E8043000==-4]<-0
A$I1103[is.na(A$I1103) & A$E8043000==-3]<-0
A$I1103[is.na(A$I1103) & A$E8043000 >= 201200]<-0
A$I1103[is.na(A$I1103) & A$E8043500==0]<-0
A$I1103[is.na(A$I1103) & A$E8043500==-3]<-0
table(A$E8023103, useNA="always")
table(A$I1103, useNA="always")
A$I1104<-NA
A$I1104[A$E8023104==0]<-0
A$I1104[A$E8023104==99]<-0
A$I1104[A$E8023104==1]<-1
A$I1104[is.na(A$I1104) & A$E8043000==-4]<-0
A$I1104[is.na(A$I1104) & A$E8043000==-3]<-0
A$I1104[is.na(A$I1104) & A$E8043000 >= 201200]<-0
A$I1104[is.na(A$I1104) & A$E8043500==0]<-0
A$I1104[is.na(A$I1104) & A$E8043500==-3]<-0
table(A$E8023104, useNA="always")
table(A$I1104, useNA="always")
A$I1105<-NA
A$I1105[A$E8023105==0]<-0
A$I1105[A$E8023105==99]<-0
A$I1105[A$E8023105==1]<-1
A$I1105[is.na(A$I1105) & A$E8043000==-4]<-0
A$I1105[is.na(A$I1105) & A$E8043000==-3]<-0
A$I1105[is.na(A$I1105) & A$E8043000 >= 201200]<-0
A$I1105[is.na(A$I1105) & A$E8043500==0]<-0
A$I1105[is.na(A$I1105) & A$E8043500==-3]<-0
table(A$E8023105, useNA="always")
table(A$I1105, useNA="always")
A$I1106<-NA
A$I1106[A$E8023106==0]<-0
A$I1106[A$E8023106==99]<-0
A$I1106[A$E8023106==1]<-1
A$I1106[is.na(A$I1106) & A$E8043000==-4]<-0
A$I1106[is.na(A$I1106) & A$E8043000==-3]<-0
A$I1106[is.na(A$I1106) & A$E8043000 >= 201200]<-0
A$I1106[is.na(A$I1106) & A$E8043500==0]<-0
A$I1106[is.na(A$I1106) & A$E8043500==-3]<-0
table(A$E8023106, useNA="always")
table(A$I1106, useNA="always")
A$I1107<-NA
```

© 2023 Silver IA et al. *JAMA Network Open.*

```
A$I1107[A$E8023107==0]<-0
A$I1107[A$E8023107==99]<-0
A$I1107[A$E8023107==1]<-1
A$I1107[is.na(A$I1107) & A$E8043000==-4]<-0
A$I1107[is.na(A$I1107) & A$E8043000==-3]<-0
A$I1107[is.na(A$I1107) & A$E8043000 >= 201200]<-0
A$I1107[is.na(A$I1107) & A$E8043500==0]<-0
A$I1107[is.na(A$I1107) & A$E8043500==-3]<-0
table(A$E8023107, useNA="always")
table(A$I1107, useNA="always")
A$I1108<-NA
A$I1108[A$E8023108==0]<-0
A$I1108[A$E8023108==99]<-0
A$I1108[A$E8023108==1]<-1
A$I1108[is.na(A$I1108) & A$E8043000==-4]<-0
A$I1108[is.na(A$I1108) & A$E8043000==-3]<-0
A$I1108[is.na(A$I1108) & A$E8043000 >= 201200]<-0
A$I1108[is.na(A$I1108) & A$E8043500==0]<-0
A$I1108[is.na(A$I1108) & A$E8043500==-3]<-0
table(A$E8023108, useNA="always")
table(A$I1108, useNA="always")
A$I1109<-NA
A$I1109[A$E8023109==0]<-0
A$I1109[A$E8023109==99]<-0
A$I1109[A$E8023109==1]<-1
A$I1109[is.na(A$I1109) & A$E8043000==-4]<-0
A$I1109[is.na(A$I1109) & A$E8043000==-3]<-0
A$I1109[is.na(A$I1109) & A$E8043000 >= 201200]<-0
A$I1109[is.na(A$I1109) & A$E8043500==0]<-0
A$I1109[is.na(A$I1109) & A$E8043500==-3]<-0
table(A$E8023109, useNA="always")
table(A$I1109, useNA="always")
A$I1110<-NA
A$I1110[A$E8023110==0]<-0
A$I1110[A$E8023110==99]<-0
A$I1110[A$E8023110==1]<-1
A$I1110[is.na(A$I1110) & A$E8043000==-4]<-0
A$I1110[is.na(A$I1110) & A$E8043000==-3]<-0
A$I1110[is.na(A$I1110) & A$E8043000 >= 201200]<-0
A$I1110[is.na(A$I1110) & A$E8043500==0]<-0
A$I1110[is.na(A$I1110) & A$E8043500==-3]<-0
table(A$E8023110, useNA="always")
table(A$I1110, useNA="always")
A$I1111<-NA
A$I1111[A$E8023111==0]<-0
A$I1111[A$E8023111==99]<-0
A$I1111[A$E8023111==1]<-1
A$I1111[is.na(A$I1111) & A$E8043000==-4]<-0
A$I1111[is.na(A$I1111) & A$E8043000==-3]<-0
A$I1111[is.na(A$I1111) & A$E8043000 >= 201200]<-0
A$I1111[is.na(A$I1111) & A$E8043500==0]<-0
A$I1111[is.na(A$I1111) & A$E8043500==-3]<-0
table(A$E8023111, useNA="always")
table(A$I1111, useNA="always")
A$I1112<-NA
A$I1112[A$E8023112==0]<-0
```

© 2023 Silver IA et al. *JAMA Network Open.*

```
A$I1112[A$E8023112==99]<-0
A$I1112[A$E8023112==1]<-1
A$I1112[is.na(A$I1112) & A$E8043000==-4]<-0
A$I1112[is.na(A$I1112) & A$E8043000==-3]<-0
A$I1112[is.na(A$I1112) & A$E8043000 >= 201200]<-0
A$I1112[is.na(A$I1112) & A$E8043500==0]<-0
A$I1112[is.na(A$I1112) & A$E8043500==-3]<-0
table(A$E8023112, useNA="always")
table(A$I1112, useNA="always")




A$I11<-
rowSums(data.frame(A$I1101,A$I1102,A$I1103,A$I1104,A$I1105,A$I1106,A$I1107,A$
I1108,A$I1109,A$I1110,A$I1111,A$I1112), na.rm = T)
A$I11[is.na(A$I11)]<-0
table(A$I11, useNA="always")

### Incarceration (2012)

table(A$E8023201, useNA="always")
table(A$E8023202)
table(A$E8023203)
table(A$E8023204)
table(A$E8023205)
table(A$E8023206)
table(A$E8023207)
table(A$E8023208)
table(A$E8023209)
table(A$E8023210)
table(A$E8023211)
table(A$E8023212)

A$I1201<-NA
A$I1201[A$E8023201==0]<-0
A$I1201[A$E8023201==99]<-0
A$I1201[A$E8023201==1]<-1
A$I1201[is.na(A$I1201) & A$E8043000==-4]<-0
A$I1201[is.na(A$I1201) & A$E8043000==-3]<-0
A$I1201[is.na(A$I1201) & A$E8043000 >= 201300]<-0
A$I1201[is.na(A$I1201) & A$E8043500==0]<-0
A$I1201[is.na(A$I1201) & A$E8043500==-3]<-0
table(A$E8023201, useNA="always")
table(A$I1201, useNA="always")
A$I1202<-NA
A$I1202[A$E8023202==0]<-0
A$I1202[A$E8023202==99]<-0
A$I1202[A$E8023202==1]<-1
A$I1202[is.na(A$I1202) & A$E8043000==-4]<-0
A$I1202[is.na(A$I1202) & A$E8043000==-3]<-0
A$I1202[is.na(A$I1202) & A$E8043000 >= 201300]<-0
A$I1202[is.na(A$I1202) & A$E8043500==0]<-0
A$I1202[is.na(A$I1202) & A$E8043500==-3]<-0
table(A$E8023202, useNA="always")
table(A$I1202, useNA="always")
```

© 2023 Silver IA et al. *JAMA Network Open.*

```
A$I1203<-NA
A$I1203[A$E8023203==0]<-0
A$I1203[A$E8023203==99]<-0
A$I1203[A$E8023203==1]<-1
A$I1203[is.na(A$I1203) & A$E8043000==-4]<-0
A$I1203[is.na(A$I1203) & A$E8043000==-3]<-0
A$I1203[is.na(A$I1203) & A$E8043000 >= 201300]<-0
A$I1203[is.na(A$I1203) & A$E8043500==0]<-0
A$I1203[is.na(A$I1203) & A$E8043500==-3]<-0
table(A$E8023203, useNA="always")
table(A$I1203, useNA="always")
A$I1204<-NA
A$I1204[A$E8023204==0]<-0
A$I1204[A$E8023204==99]<-0
A$I1204[A$E8023204==1]<-1
A$I1204[is.na(A$I1204) & A$E8043000==-4]<-0
A$I1204[is.na(A$I1204) & A$E8043000==-3]<-0
A$I1204[is.na(A$I1204) & A$E8043000 >= 201300]<-0
A$I1204[is.na(A$I1204) & A$E8043500==0]<-0
A$I1204[is.na(A$I1204) & A$E8043500==-3]<-0
table(A$E8023204, useNA="always")
table(A$I1204, useNA="always")
A$I1205<-NA
A$I1205[A$E8023205==0]<-0
A$I1205[A$E8023205==99]<-0
A$I1205[A$E8023205==1]<-1
A$I1205[is.na(A$I1205) & A$E8043000==-4]<-0
A$I1205[is.na(A$I1205) & A$E8043000==-3]<-0
A$I1205[is.na(A$I1205) & A$E8043000 >= 201300]<-0
A$I1205[is.na(A$I1205) & A$E8043500==0]<-0
A$I1205[is.na(A$I1205) & A$E8043500==-3]<-0
table(A$E8023205, useNA="always")
table(A$I1205, useNA="always")
A$I1206<-NA
A$I1206[A$E8023206==0]<-0
A$I1206[A$E8023206==99]<-0
A$I1206[A$E8023206==1]<-1
A$I1206[is.na(A$I1206) & A$E8043000==-4]<-0
A$I1206[is.na(A$I1206) & A$E8043000==-3]<-0
A$I1206[is.na(A$I1206) & A$E8043000 >= 201300]<-0
A$I1206[is.na(A$I1206) & A$E8043500==0]<-0
A$I1206[is.na(A$I1206) & A$E8043500==-3]<-0
table(A$E8023206, useNA="always")
table(A$I1206, useNA="always")
A$I1207<-NA
A$I1207[A$E8023207==0]<-0
A$I1207[A$E8023207==99]<-0
A$I1207[A$E8023207==1]<-1
A$I1207[is.na(A$I1207) & A$E8043000==-4]<-0
A$I1207[is.na(A$I1207) & A$E8043000==-3]<-0
A$I1207[is.na(A$I1207) & A$E8043000 >= 201300]<-0
A$I1207[is.na(A$I1207) & A$E8043500==0]<-0
A$I1207[is.na(A$I1207) & A$E8043500==-3]<-0
table(A$E8023207, useNA="always")
table(A$I1207, useNA="always")
A$I1208<-NA
```

```
A$I1208[A$E8023208==0]<-0
A$I1208[A$E8023208==99]<-0
A$I1208[A$E8023208==1]<-1
A$I1208[is.na(A$I1208) & A$E8043000==-4]<-0
A$I1208[is.na(A$I1208) & A$E8043000==-3]<-0
A$I1208[is.na(A$I1208) & A$E8043000 >= 201300]<-0
A$I1208[is.na(A$I1208) & A$E8043500==0]<-0
A$I1208[is.na(A$I1208) & A$E8043500==-3]<-0
table(A$E8023208, useNA="always")
table(A$I1208, useNA="always")
A$I1209<-NA
A$I1209[A$E8023209==0]<-0
A$I1209[A$E8023209==99]<-0
A$I1209[A$E8023209==1]<-1
A$I1209[is.na(A$I1209) & A$E8043000==-4]<-0
A$I1209[is.na(A$I1209) & A$E8043000==-3]<-0
A$I1209[is.na(A$I1209) & A$E8043000 >= 201300]<-0
A$I1209[is.na(A$I1209) & A$E8043500==0]<-0
A$I1209[is.na(A$I1209) & A$E8043500==-3]<-0
table(A$E8023209, useNA="always")
table(A$I1209, useNA="always")
A$I1210<-NA
A$I1210[A$E8023210==0]<-0
A$I1210[A$E8023210==99]<-0
A$I1210[A$E8023210==1]<-1
A$I1210[is.na(A$I1210) & A$E8043000==-4]<-0
A$I1210[is.na(A$I1210) & A$E8043000==-3]<-0
A$I1210[is.na(A$I1210) & A$E8043000 >= 201300]<-0
A$I1210[is.na(A$I1210) & A$E8043500==0]<-0
A$I1210[is.na(A$I1210) & A$E8043500==-3]<-0
table(A$E8023210, useNA="always")
table(A$I1210, useNA="always")
A$I1211<-NA
A$I1211[A$E8023211==0]<-0
A$I1211[A$E8023211==99]<-0
A$I1211[A$E8023211==1]<-1
A$I1211[is.na(A$I1211) & A$E8043000==-4]<-0
A$I1211[is.na(A$I1211) & A$E8043000==-3]<-0
A$I1211[is.na(A$I1211) & A$E8043000 >= 201300]<-0
A$I1211[is.na(A$I1211) & A$E8043500==0]<-0
A$I1211[is.na(A$I1211) & A$E8043500==-3]<-0
table(A$E8023211, useNA="always")
table(A$I1211, useNA="always")
A$I1212<-NA
A$I1212[A$E8023212==0]<-0
A$I1212[A$E8023212==99]<-0
A$I1212[A$E8023212==1]<-1
A$I1212[is.na(A$I1212) & A$E8043000==-4]<-0
A$I1212[is.na(A$I1212) & A$E8043000==-3]<-0
A$I1212[is.na(A$I1212) & A$E8043000 >= 201300]<-0
A$I1212[is.na(A$I1212) & A$E8043500==0]<-0
A$I1212[is.na(A$I1212) & A$E8043500==-3]<-0
table(A$E8023212, useNA="always")
table(A$I1212, useNA="always")
```

© 2023 Silver IA et al. *JAMA Network Open.*

```
A$I12<-
rowSums(data.frame(A$I1201,A$I1202,A$I1203,A$I1204,A$I1205,A$I1206,A$I1207,A$
I1208,A$I1209,A$I1210,A$I1211,A$I1212), na.rm = T)
A$I12[is.na(A$I12)]<-0
table(A$I12, useNA="always")

### Incarceration (2013)

table(A$E8023301, useNA="always")
table(A$E8023302)
table(A$E8023303)
table(A$E8023304)
table(A$E8023305)
table(A$E8023306)
table(A$E8023307)
table(A$E8023308)
table(A$E8023309)
table(A$E8023310)
table(A$E8023311)
table(A$E8023312)

A$I1301<-NA
A$I1301[A$E8023301==0]<-0
A$I1301[A$E8023301==99]<-0
A$I1301[A$E8023301==1]<-1
A$I1301[is.na(A$I1301) & A$E8043000==-4]<-0
A$I1301[is.na(A$I1301) & A$E8043000==-3]<-0
A$I1301[is.na(A$I1301) & A$E8043000 >= 201400]<-0
A$I1301[is.na(A$I1301) & A$E8043500==0]<-0
A$I1301[is.na(A$I1301) & A$E8043500==-3]<-0
table(A$E8023301, useNA="always")
table(A$I1301, useNA="always")
A$I1302<-NA
A$I1302[A$E8023302==0]<-0
A$I1302[A$E8023302==99]<-0
A$I1302[A$E8023302==1]<-1
A$I1302[is.na(A$I1302) & A$E8043000==-4]<-0
A$I1302[is.na(A$I1302) & A$E8043000==-3]<-0
A$I1302[is.na(A$I1302) & A$E8043000 >= 201400]<-0
A$I1302[is.na(A$I1302) & A$E8043500==0]<-0
A$I1302[is.na(A$I1302) & A$E8043500==-3]<-0
table(A$E8023302, useNA="always")
table(A$I1302, useNA="always")
A$I1303<-NA
A$I1303[A$E8023303==0]<-0
A$I1303[A$E8023303==99]<-0
A$I1303[A$E8023303==1]<-1
A$I1303[is.na(A$I1303) & A$E8043000==-4]<-0
A$I1303[is.na(A$I1303) & A$E8043000==-3]<-0
A$I1303[is.na(A$I1303) & A$E8043000 >= 201400]<-0
A$I1303[is.na(A$I1303) & A$E8043500==0]<-0
A$I1303[is.na(A$I1303) & A$E8043500==-3]<-0
table(A$E8023303, useNA="always")
table(A$I1303, useNA="always")
```

© 2023 Silver IA et al. *JAMA Network Open.*

```
A$I1304<-NA
A$I1304[A$E8023304==0]<-0
A$I1304[A$E8023304==99]<-0
A$I1304[A$E8023304==1]<-1
A$I1304[is.na(A$I1304) & A$E8043000==-4]<-0
A$I1304[is.na(A$I1304) & A$E8043000==-3]<-0
A$I1304[is.na(A$I1304) & A$E8043000 >= 201400]<-0
A$I1304[is.na(A$I1304) & A$E8043500==0]<-0
A$I1304[is.na(A$I1304) & A$E8043500==-3]<-0
table(A$E8023304, useNA="always")
table(A$I1304, useNA="always")
A$I1305<-NA
A$I1305[A$E8023305==0]<-0
A$I1305[A$E8023305==99]<-0
A$I1305[A$E8023305==1]<-1
A$I1305[is.na(A$I1305) & A$E8043000==-4]<-0
A$I1305[is.na(A$I1305) & A$E8043000==-3]<-0
A$I1305[is.na(A$I1305) & A$E8043000 >= 201400]<-0
A$I1305[is.na(A$I1305) & A$E8043500==0]<-0
A$I1305[is.na(A$I1305) & A$E8043500==-3]<-0
table(A$E8023305, useNA="always")
table(A$I1305, useNA="always")
A$I1306<-NA
A$I1306[A$E8023306==0]<-0
A$I1306[A$E8023306==99]<-0
A$I1306[A$E8023306==1]<-1
A$I1306[is.na(A$I1306) & A$E8043000==-4]<-0
A$I1306[is.na(A$I1306) & A$E8043000==-3]<-0
A$I1306[is.na(A$I1306) & A$E8043000 >= 201400]<-0
A$I1306[is.na(A$I1306) & A$E8043500==0]<-0
A$I1306[is.na(A$I1306) & A$E8043500==-3]<-0
table(A$E8023306, useNA="always")
table(A$I1306, useNA="always")
A$I1307<-NA
A$I1307[A$E8023307==0]<-0
A$I1307[A$E8023307==99]<-0
A$I1307[A$E8023307==1]<-1
A$I1307[is.na(A$I1307) & A$E8043000==-4]<-0
A$I1307[is.na(A$I1307) & A$E8043000==-3]<-0
A$I1307[is.na(A$I1307) & A$E8043000 >= 201400]<-0
A$I1307[is.na(A$I1307) & A$E8043500==0]<-0
A$I1307[is.na(A$I1307) & A$E8043500==-3]<-0
table(A$E8023307, useNA="always")
table(A$I1307, useNA="always")
A$I1308<-NA
A$I1308[A$E8023308==0]<-0
A$I1308[A$E8023308==99]<-0
A$I1308[A$E8023308==1]<-1
A$I1308[is.na(A$I1308) & A$E8043000==-4]<-0
A$I1308[is.na(A$I1308) & A$E8043000==-3]<-0
A$I1308[is.na(A$I1308) & A$E8043000 >= 201400]<-0
A$I1308[is.na(A$I1308) & A$E8043500==0]<-0
A$I1308[is.na(A$I1308) & A$E8043500==-3]<-0
table(A$E8023308, useNA="always")
table(A$I1308, useNA="always")
A$I1309<-NA
```

© 2023 Silver IA et al. *JAMA Network Open.*

```
A$I1309[A$E8023309==0]<-0
A$I1309[A$E8023309==99]<-0
A$I1309[A$E8023309==1]<-1
A$I1309[is.na(A$I1309) & A$E8043000==-4]<-0
A$I1309[is.na(A$I1309) & A$E8043000==-3]<-0
A$I1309[is.na(A$I1309) & A$E8043000 >= 201400]<-0
A$I1309[is.na(A$I1309) & A$E8043500==0]<-0
A$I1309[is.na(A$I1309) & A$E8043500==-3]<-0
table(A$E8023309, useNA="always")
table(A$I1309, useNA="always")
A$I1310<-NA
A$I1310[A$E8023310==0]<-0
A$I1310[A$E8023310==99]<-0
A$I1310[A$E8023310==1]<-1
A$I1310[is.na(A$I1310) & A$E8043000==-4]<-0
A$I1310[is.na(A$I1310) & A$E8043000==-3]<-0
A$I1310[is.na(A$I1310) & A$E8043000 >= 201400]<-0
A$I1310[is.na(A$I1310) & A$E8043500==0]<-0
A$I1310[is.na(A$I1310) & A$E8043500==-3]<-0
table(A$E8023310, useNA="always")
table(A$I1310, useNA="always")
A$I1311<-NA
A$I1311[A$E8023311==0]<-0
A$I1311[A$E8023311==99]<-0
A$I1311[A$E8023311==1]<-1
A$I1311[is.na(A$I1311) & A$E8043000==-4]<-0
A$I1311[is.na(A$I1311) & A$E8043000==-3]<-0
A$I1311[is.na(A$I1311) & A$E8043000 >= 201400]<-0
A$I1311[is.na(A$I1311) & A$E8043500==0]<-0
A$I1311[is.na(A$I1311) & A$E8043500==-3]<-0
table(A$E8023311, useNA="always")
table(A$I1311, useNA="always")
A$I1312<-NA
A$I1312[A$E8023312==0]<-0
A$I1312[A$E8023312==99]<-0
A$I1312[A$E8023312==1]<-1
A$I1312[is.na(A$I1312) & A$E8043000==-4]<-0
A$I1312[is.na(A$I1312) & A$E8043000==-3]<-0
A$I1312[is.na(A$I1312) & A$E8043000 >= 201400]<-0
A$I1312[is.na(A$I1312) & A$E8043500==0]<-0
A$I1312[is.na(A$I1312) & A$E8043500==-3]<-0
table(A$E8023312, useNA="always")
table(A$I1312, useNA="always")




A$I13<-
rowSums(data.frame(A$I1301,A$I1302,A$I1303,A$I1304,A$I1305,A$I1306,A$I1307,A$
I1308,A$I1309,A$I1310,A$I1311,A$I1312), na.rm = T)
A$I13[is.na(A$I13)]<-0
table(A$I13, useNA="always")

### Incarceration (2014)

table(A$E8023401, useNA="always")
```

© 2023 Silver IA et al. *JAMA Network Open.*

```
table(A$E8023402)
table(A$E8023403)
table(A$E8023404)
table(A$E8023405)
table(A$E8023406)
table(A$E8023407)
table(A$E8023408)
table(A$E8023409)
table(A$E8023410)
table(A$E8023411)
table(A$E8023412)

A$I1401<-NA
A$I1401[A$E8023401==0]<-0
A$I1401[A$E8023401==99]<-0
A$I1401[A$E8023401==1]<-1
A$I1401[is.na(A$I1401) & A$E8043000==-4]<-0
A$I1401[is.na(A$I1401) & A$E8043000==-3]<-0
A$I1401[is.na(A$I1401) & A$E8043000 >= 201500]<-0
A$I1401[is.na(A$I1401) & A$E8043500==0]<-0
A$I1401[is.na(A$I1401) & A$E8043500==-3]<-0
table(A$E8023401, useNA="always")
table(A$I1401, useNA="always")
A$I1402<-NA
A$I1402[A$E8023402==0]<-0
A$I1402[A$E8023402==99]<-0
A$I1402[A$E8023402==1]<-1
A$I1402[is.na(A$I1402) & A$E8043000==-4]<-0
A$I1402[is.na(A$I1402) & A$E8043000==-3]<-0
A$I1402[is.na(A$I1402) & A$E8043000 >= 201500]<-0
A$I1402[is.na(A$I1402) & A$E8043500==0]<-0
A$I1402[is.na(A$I1402) & A$E8043500==-3]<-0
table(A$E8023402, useNA="always")
table(A$I1402, useNA="always")
A$I1403<-NA
A$I1403[A$E8023403==0]<-0
A$I1403[A$E8023403==99]<-0
A$I1403[A$E8023403==1]<-1
A$I1403[is.na(A$I1403) & A$E8043000==-4]<-0
A$I1403[is.na(A$I1403) & A$E8043000==-3]<-0
A$I1403[is.na(A$I1403) & A$E8043000 >= 201500]<-0
A$I1403[is.na(A$I1403) & A$E8043500==0]<-0
A$I1403[is.na(A$I1403) & A$E8043500==-3]<-0
table(A$E8023403, useNA="always")
table(A$I1403, useNA="always")
A$I1404<-NA
A$I1404[A$E8023404==0]<-0
A$I1404[A$E8023404==99]<-0
A$I1404[A$E8023404==1]<-1
A$I1404[is.na(A$I1404) & A$E8043000==-4]<-0
A$I1404[is.na(A$I1404) & A$E8043000==-3]<-0
A$I1404[is.na(A$I1404) & A$E8043000 >= 201500]<-0
A$I1404[is.na(A$I1404) & A$E8043500==0]<-0
A$I1404[is.na(A$I1404) & A$E8043500==-3]<-0
table(A$E8023404, useNA="always")
table(A$I1404, useNA="always")
```

© 2023 Silver IA et al. *JAMA Network Open.*

```
A$I1405<-NA
A$I1405[A$E8023405==0]<-0
A$I1405[A$E8023405==99]<-0
A$I1405[A$E8023405==1]<-1
A$I1405[is.na(A$I1405) & A$E8043000==-4]<-0
A$I1405[is.na(A$I1405) & A$E8043000==-3]<-0
A$I1405[is.na(A$I1405) & A$E8043000 >= 201500]<-0
A$I1405[is.na(A$I1405) & A$E8043500==0]<-0
A$I1405[is.na(A$I1405) & A$E8043500==-3]<-0
table(A$E8023405, useNA="always")
table(A$I1405, useNA="always")
A$I1406<-NA
A$I1406[A$E8023406==0]<-0
A$I1406[A$E8023406==99]<-0
A$I1406[A$E8023406==1]<-1
A$I1406[is.na(A$I1406) & A$E8043000==-4]<-0
A$I1406[is.na(A$I1406) & A$E8043000==-3]<-0
A$I1406[is.na(A$I1406) & A$E8043000 >= 201500]<-0
A$I1406[is.na(A$I1406) & A$E8043500==0]<-0
A$I1406[is.na(A$I1406) & A$E8043500==-3]<-0
table(A$E8023406, useNA="always")
table(A$I1406, useNA="always")
A$I1407<-NA
A$I1407[A$E8023407==0]<-0
A$I1407[A$E8023407==99]<-0
A$I1407[A$E8023407==1]<-1
A$I1407[is.na(A$I1407) & A$E8043000==-4]<-0
A$I1407[is.na(A$I1407) & A$E8043000==-3]<-0
A$I1407[is.na(A$I1407) & A$E8043000 >= 201500]<-0
A$I1407[is.na(A$I1407) & A$E8043500==0]<-0
A$I1407[is.na(A$I1407) & A$E8043500==-3]<-0
table(A$E8023407, useNA="always")
table(A$I1407, useNA="always")
A$I1408<-NA
A$I1408[A$E8023408==0]<-0
A$I1408[A$E8023408==99]<-0
A$I1408[A$E8023408==1]<-1
A$I1408[is.na(A$I1408) & A$E8043000==-4]<-0
A$I1408[is.na(A$I1408) & A$E8043000==-3]<-0
A$I1408[is.na(A$I1408) & A$E8043000 >= 201500]<-0
A$I1408[is.na(A$I1408) & A$E8043500==0]<-0
A$I1408[is.na(A$I1408) & A$E8043500==-3]<-0
table(A$E8023408, useNA="always")
table(A$I1408, useNA="always")
A$I1409<-NA
A$I1409[A$E8023409==0]<-0
A$I1409[A$E8023409==99]<-0
A$I1409[A$E8023409==1]<-1
A$I1409[is.na(A$I1409) & A$E8043000==-4]<-0
A$I1409[is.na(A$I1409) & A$E8043000==-3]<-0
A$I1409[is.na(A$I1409) & A$E8043000 >= 201500]<-0
A$I1409[is.na(A$I1409) & A$E8043500==0]<-0
A$I1409[is.na(A$I1409) & A$E8043500==-3]<-0
table(A$E8023409, useNA="always")
table(A$I1409, useNA="always")
A$I1410<-NA
```

© 2023 Silver IA et al. *JAMA Network Open.*

```
A$I1410[A$E8023410==0]<-0
A$I1410[A$E8023410==99]<-0
A$I1410[A$E8023410==1]<-1
A$I1410[is.na(A$I1410) & A$E8043000==-4]<-0
A$I1410[is.na(A$I1410) & A$E8043000==-3]<-0
A$I1410[is.na(A$I1410) & A$E8043000 >= 201500]<-0
A$I1410[is.na(A$I1410) & A$E8043500==0]<-0
A$I1410[is.na(A$I1410) & A$E8043500==-3]<-0
table(A$E8023410, useNA="always")
table(A$I1410, useNA="always")
A$I1411<-NA
A$I1411[A$E8023411==0]<-0
A$I1411[A$E8023411==99]<-0
A$I1411[A$E8023411==1]<-1
A$I1411[is.na(A$I1411) & A$E8043000==-4]<-0
A$I1411[is.na(A$I1411) & A$E8043000==-3]<-0
A$I1411[is.na(A$I1411) & A$E8043000 >= 201500]<-0
A$I1411[is.na(A$I1411) & A$E8043500==0]<-0
A$I1411[is.na(A$I1411) & A$E8043500==-3]<-0
table(A$E8023411, useNA="always")
table(A$I1411, useNA="always")
A$I1412<-NA
A$I1412[A$E8023412==0]<-0
A$I1412[A$E8023412==99]<-0
A$I1412[A$E8023412==1]<-1
A$I1412[is.na(A$I1412) & A$E8043000==-4]<-0
A$I1412[is.na(A$I1412) & A$E8043000==-3]<-0
A$I1412[is.na(A$I1412) & A$E8043000 >= 201500]<-0
A$I1412[is.na(A$I1412) & A$E8043500==0]<-0
A$I1412[is.na(A$I1412) & A$E8043500==-3]<-0
table(A$E8023412, useNA="always")
table(A$I1412, useNA="always")



A$I14<-
rowSums(data.frame(A$I1401,A$I1402,A$I1403,A$I1404,A$I1405,A$I1406,A$I1407,A$
I1408,A$I1409,A$I1410,A$I1411,A$I1412), na.rm = T)
A$I14[is.na(A$I14)]<-0
table(A$I14, useNA="always")

### Incarceration (2015)

table(A$E8023501, useNA="always")
table(A$E8023502)
table(A$E8023503)
table(A$E8023504)
table(A$E8023505)
table(A$E8023506)
table(A$E8023507)
table(A$E8023508)
table(A$E8023509)
table(A$E8023510)
table(A$E8023511)
table(A$E8023512)
```

© 2023 Silver IA et al. *JAMA Network Open.*

```
A$I1501<-NA
A$I1501[A$E8023501==0]<-0
A$I1501[A$E8023501==99]<-0
A$I1501[A$E8023501==1]<-1
A$I1501[is.na(A$I1501) & A$E8043000==-4]<-0
A$I1501[is.na(A$I1501) & A$E8043000==-3]<-0
A$I1501[is.na(A$I1501) & A$E8043000 >= 201600]<-0
A$I1501[is.na(A$I1501) & A$E8043500==0]<-0
A$I1501[is.na(A$I1501) & A$E8043500==-3]<-0
table(A$E8023501, useNA="always")
table(A$I1501, useNA="always")
A$I1502<-NA
A$I1502[A$E8023502==0]<-0
A$I1502[A$E8023502==99]<-0
A$I1502[A$E8023502==1]<-1
A$I1502[is.na(A$I1502) & A$E8043000==-4]<-0
A$I1502[is.na(A$I1502) & A$E8043000==-3]<-0
A$I1502[is.na(A$I1502) & A$E8043000 >= 201600]<-0
A$I1502[is.na(A$I1502) & A$E8043500==0]<-0
A$I1502[is.na(A$I1502) & A$E8043500==-3]<-0
table(A$E8023502, useNA="always")
table(A$I1502, useNA="always")
A$I1503<-NA
A$I1503[A$E8023503==0]<-0
A$I1503[A$E8023503==99]<-0
A$I1503[A$E8023503==1]<-1
A$I1503[is.na(A$I1503) & A$E8043000==-4]<-0
A$I1503[is.na(A$I1503) & A$E8043000==-3]<-0
A$I1503[is.na(A$I1503) & A$E8043000 >= 201600]<-0
A$I1503[is.na(A$I1503) & A$E8043500==0]<-0
A$I1503[is.na(A$I1503) & A$E8043500==-3]<-0
table(A$E8023503, useNA="always")
table(A$I1503, useNA="always")
A$I1504<-NA
A$I1504[A$E8023504==0]<-0
A$I1504[A$E8023504==99]<-0
A$I1504[A$E8023504==1]<-1
A$I1504[is.na(A$I1504) & A$E8043000==-4]<-0
A$I1504[is.na(A$I1504) & A$E8043000==-3]<-0
A$I1504[is.na(A$I1504) & A$E8043000 >= 201600]<-0
A$I1504[is.na(A$I1504) & A$E8043500==0]<-0
A$I1504[is.na(A$I1504) & A$E8043500==-3]<-0
table(A$E8023504, useNA="always")
table(A$I1504, useNA="always")
A$I1505<-NA
A$I1505[A$E8023505==0]<-0
A$I1505[A$E8023505==99]<-0
A$I1505[A$E8023505==1]<-1
A$I1505[is.na(A$I1505) & A$E8043000==-4]<-0
A$I1505[is.na(A$I1505) & A$E8043000==-3]<-0
A$I1505[is.na(A$I1505) & A$E8043000 >= 201600]<-0
A$I1505[is.na(A$I1505) & A$E8043500==0]<-0
A$I1505[is.na(A$I1505) & A$E8043500==-3]<-0
table(A$E8023505, useNA="always")
table(A$I1505, useNA="always")
```

© 2023 Silver IA et al. *JAMA Network Open.*

```
A$I1506<-NA
A$I1506[A$E8023506==0]<-0
A$I1506[A$E8023506==99]<-0
A$I1506[A$E8023506==1]<-1
A$I1506[is.na(A$I1506) & A$E8043000==-4]<-0
A$I1506[is.na(A$I1506) & A$E8043000==-3]<-0
A$I1506[is.na(A$I1506) & A$E8043000 >= 201600]<-0
A$I1506[is.na(A$I1506) & A$E8043500==0]<-0
A$I1506[is.na(A$I1506) & A$E8043500==-3]<-0
table(A$E8023506, useNA="always")
table(A$I1506, useNA="always")
A$I1507<-NA
A$I1507[A$E8023507==0]<-0
A$I1507[A$E8023507==99]<-0
A$I1507[A$E8023507==1]<-1
A$I1507[is.na(A$I1507) & A$E8043000==-4]<-0
A$I1507[is.na(A$I1507) & A$E8043000==-3]<-0
A$I1507[is.na(A$I1507) & A$E8043000 >= 201600]<-0
A$I1507[is.na(A$I1507) & A$E8043500==0]<-0
A$I1507[is.na(A$I1507) & A$E8043500==-3]<-0
table(A$E8023507, useNA="always")
table(A$I1507, useNA="always")
A$I1508<-NA
A$I1508[A$E8023508==0]<-0
A$I1508[A$E8023508==99]<-0
A$I1508[A$E8023508==1]<-1
A$I1508[is.na(A$I1508) & A$E8043000==-4]<-0
A$I1508[is.na(A$I1508) & A$E8043000==-3]<-0
A$I1508[is.na(A$I1508) & A$E8043000 >= 201600]<-0
A$I1508[is.na(A$I1508) & A$E8043500==0]<-0
A$I1508[is.na(A$I1508) & A$E8043500==-3]<-0
table(A$E8023508, useNA="always")
table(A$I1508, useNA="always")
A$I1509<-NA
A$I1509[A$E8023509==0]<-0
A$I1509[A$E8023509==99]<-0
A$I1509[A$E8023509==1]<-1
A$I1509[is.na(A$I1509) & A$E8043000==-4]<-0
A$I1509[is.na(A$I1509) & A$E8043000==-3]<-0
A$I1509[is.na(A$I1509) & A$E8043000 >= 201600]<-0
A$I1509[is.na(A$I1509) & A$E8043500==0]<-0
A$I1509[is.na(A$I1509) & A$E8043500==-3]<-0
table(A$E8023509, useNA="always")
table(A$I1509, useNA="always")
A$I1510<-NA
A$I1510[A$E8023510==0]<-0
A$I1510[A$E8023510==99]<-0
A$I1510[A$E8023510==1]<-1
A$I1510[is.na(A$I1510) & A$E8043000==-4]<-0
A$I1510[is.na(A$I1510) & A$E8043000==-3]<-0
A$I1510[is.na(A$I1510) & A$E8043000 >= 201600]<-0
A$I1510[is.na(A$I1510) & A$E8043500==0]<-0
A$I1510[is.na(A$I1510) & A$E8043500==-3]<-0
table(A$E8023510, useNA="always")
table(A$I1510, useNA="always")
A$I1511<-NA
```

© 2023 Silver IA et al. *JAMA Network Open.*

```
A$I1511[A$E8023511==0]<-0
A$I1511[A$E8023511==99]<-0
A$I1511[A$E8023511==1]<-1
A$I1511[is.na(A$I1511) & A$E8043000==-4]<-0
A$I1511[is.na(A$I1511) & A$E8043000==-3]<-0
A$I1511[is.na(A$I1511) & A$E8043000 >= 201600]<-0
A$I1511[is.na(A$I1511) & A$E8043500==0]<-0
A$I1511[is.na(A$I1511) & A$E8043500==-3]<-0
table(A$E8023511, useNA="always")
table(A$I1511, useNA="always")
A$I1512<-NA
A$I1512[A$E8023512==0]<-0
A$I1512[A$E8023512==99]<-0
A$I1512[A$E8023512==1]<-1
A$I1512[is.na(A$I1512) & A$E8043000==-4]<-0
A$I1512[is.na(A$I1512) & A$E8043000==-3]<-0
A$I1512[is.na(A$I1512) & A$E8043000 >= 201600]<-0
A$I1512[is.na(A$I1512) & A$E8043500==0]<-0
A$I1512[is.na(A$I1512) & A$E8043500==-3]<-0
table(A$E8023512, useNA="always")
table(A$I1512, useNA="always")




A$I15<-
rowSums(data.frame(A$I1501,A$I1502,A$I1503,A$I1504,A$I1505,A$I1506,A$I1507,A$
I1508,A$I1509,A$I1510,A$I1511,A$I1512), na.rm = T)
A$I15[is.na(A$I15)]<-0
table(A$I15, useNA="always")

### Incarceration (2016)

table(A$E8023601, useNA="always")
table(A$E8023602)
table(A$E8023603)
table(A$E8023604)
table(A$E8023605)
table(A$E8023606)
table(A$E8023607)
table(A$E8023608)
table(A$E8023609)
table(A$E8023610)
table(A$E8023611)
table(A$E8023612)

A$I1601<-NA
A$I1601[A$E8023601==0]<-0
A$I1601[A$E8023601==99]<-0
A$I1601[A$E8023601==1]<-1
A$I1601[is.na(A$I1601) & A$E8043000==-4]<-0
A$I1601[is.na(A$I1601) & A$E8043000==-3]<-0
A$I1601[is.na(A$I1601) & A$E8043000 >= 201700]<-0
A$I1601[is.na(A$I1601) & A$E8043500==0]<-0
A$I1601[is.na(A$I1601) & A$E8043500==-3]<-0
table(A$E8023601, useNA="always")
```

```
table(A$I1601, useNA="always")
A$I1602<-NA
A$I1602[A$E8023602==0]<-0
A$I1602[A$E8023602==99]<-0
A$I1602[A$E8023602==1]<-1
A$I1602[is.na(A$I1602) & A$E8043000==-4]<-0
A$I1602[is.na(A$I1602) & A$E8043000==-3]<-0
A$I1602[is.na(A$I1602) & A$E8043000 >= 201600]<-0
A$I1602[is.na(A$I1602) & A$E8043500==0]<-0
A$I1602[is.na(A$I1602) & A$E8043500==-3]<-0
table(A$E8023602, useNA="always")
table(A$I1602, useNA="always")
A$I1603<-NA
A$I1603[A$E8023603==0]<-0
A$I1603[A$E8023603==99]<-0
A$I1603[A$E8023603==1]<-1
A$I1603[is.na(A$I1603) & A$E8043000==-4]<-0
A$I1603[is.na(A$I1603) & A$E8043000==-3]<-0
A$I1603[is.na(A$I1603) & A$E8043000 >= 201600]<-0
A$I1603[is.na(A$I1603) & A$E8043500==0]<-0
A$I1603[is.na(A$I1603) & A$E8043500==-3]<-0
table(A$E8023603, useNA="always")
table(A$I1603, useNA="always")
A$I1604<-NA
A$I1604[A$E8023604==0]<-0
A$I1604[A$E8023604==99]<-0
A$I1604[A$E8023604==1]<-1
A$I1604[is.na(A$I1604) & A$E8043000==-4]<-0
A$I1604[is.na(A$I1604) & A$E8043000==-3]<-0
A$I1604[is.na(A$I1604) & A$E8043000 >= 201600]<-0
A$I1604[is.na(A$I1604) & A$E8043500==0]<-0
A$I1604[is.na(A$I1604) & A$E8043500==-3]<-0
table(A$E8023604, useNA="always")
table(A$I1604, useNA="always")
A$I1605<-NA
A$I1605[A$E8023605==0]<-0
A$I1605[A$E8023605==99]<-0
A$I1605[A$E8023605==1]<-1
A$I1605[is.na(A$I1605) & A$E8043000==-4]<-0
A$I1605[is.na(A$I1605) & A$E8043000==-3]<-0
A$I1605[is.na(A$I1605) & A$E8043000 >= 201600]<-0
A$I1605[is.na(A$I1605) & A$E8043500==0]<-0
A$I1605[is.na(A$I1605) & A$E8043500==-3]<-0
table(A$E8023605, useNA="always")
table(A$I1605, useNA="always")
A$I1606<-NA
A$I1606[A$E8023606==0]<-0
A$I1606[A$E8023606==99]<-0
A$I1606[A$E8023606==1]<-1
A$I1606[is.na(A$I1606) & A$E8043000==-4]<-0
A$I1606[is.na(A$I1606) & A$E8043000==-3]<-0
A$I1606[is.na(A$I1606) & A$E8043000 >= 201600]<-0
A$I1606[is.na(A$I1606) & A$E8043500==0]<-0
A$I1606[is.na(A$I1606) & A$E8043500==-3]<-0
table(A$E8023606, useNA="always")
table(A$I1606, useNA="always")
```

```
A$I1607<-NA
A$I1607[A$E8023607==0]<-0
A$I1607[A$E8023607==99]<-0
A$I1607[A$E8023607==1]<-1
A$I1607[is.na(A$I1607) & A$E8043000==-4]<-0
A$I1607[is.na(A$I1607) & A$E8043000==-3]<-0
A$I1607[is.na(A$I1607) & A$E8043000 >= 201600]<-0
A$I1607[is.na(A$I1607) & A$E8043500==0]<-0
A$I1607[is.na(A$I1607) & A$E8043500==-3]<-0
table(A$E8023607, useNA="always")
table(A$I1607, useNA="always")
A$I1608<-NA
A$I1608[A$E8023608==0]<-0
A$I1608[A$E8023608==99]<-0
A$I1608[A$E8023608==1]<-1
A$I1608[is.na(A$I1608) & A$E8043000==-4]<-0
A$I1608[is.na(A$I1608) & A$E8043000==-3]<-0
A$I1608[is.na(A$I1608) & A$E8043000 >= 201600]<-0
A$I1608[is.na(A$I1608) & A$E8043500==0]<-0
A$I1608[is.na(A$I1608) & A$E8043500==-3]<-0
table(A$E8023608, useNA="always")
table(A$I1608, useNA="always")
A$I1609<-NA
A$I1609[A$E8023609==0]<-0
A$I1609[A$E8023609==99]<-0
A$I1609[A$E8023609==1]<-1
A$I1609[is.na(A$I1609) & A$E8043000==-4]<-0
A$I1609[is.na(A$I1609) & A$E8043000==-3]<-0
A$I1609[is.na(A$I1609) & A$E8043000 >= 201600]<-0
A$I1609[is.na(A$I1609) & A$E8043500==0]<-0
A$I1609[is.na(A$I1609) & A$E8043500==-3]<-0
table(A$E8023609, useNA="always")
table(A$I1609, useNA="always")
A$I1610<-NA
A$I1610[A$E8023610==0]<-0
A$I1610[A$E8023610==99]<-0
A$I1610[A$E8023610==1]<-1
A$I1610[is.na(A$I1610) & A$E8043000==-4]<-0
A$I1610[is.na(A$I1610) & A$E8043000==-3]<-0
A$I1610[is.na(A$I1610) & A$E8043000 >= 201600]<-0
A$I1610[is.na(A$I1610) & A$E8043500==0]<-0
A$I1610[is.na(A$I1610) & A$E8043500==-3]<-0
table(A$E8023610, useNA="always")
table(A$I1610, useNA="always")
A$I1611<-NA
A$I1611[A$E8023611==0]<-0
A$I1611[A$E8023611==99]<-0
A$I1611[A$E8023611==1]<-1
A$I1611[is.na(A$I1611) & A$E8043000==-4]<-0
A$I1611[is.na(A$I1611) & A$E8043000==-3]<-0
A$I1611[is.na(A$I1611) & A$E8043000 >= 201600]<-0
A$I1611[is.na(A$I1611) & A$E8043500==0]<-0
A$I1611[is.na(A$I1611) & A$E8043500==-3]<-0
table(A$E8023611, useNA="always")
table(A$I1611, useNA="always")
A$I1612<-NA
```

© 2023 Silver IA et al. *JAMA Network Open.*

```
A$I1612[A$E8023612==0]<-0
A$I1612[A$E8023612==99]<-0
A$I1612[A$E8023612==1]<-1
A$I1612[is.na(A$I1612) & A$E8043000==-4]<-0
A$I1612[is.na(A$I1612) & A$E8043000==-3]<-0
A$I1612[is.na(A$I1612) & A$E8043000 >= 201600]<-0
A$I1612[is.na(A$I1612) & A$E8043500==0]<-0
A$I1612[is.na(A$I1612) & A$E8043500==-3]<-0
table(A$E8023612, useNA="always")
table(A$I1612, useNA="always")




A$I16<-
rowSums(data.frame(A$I1601,A$I1602,A$I1603,A$I1604,A$I1605,A$I1606,A$I1607,A$
I1608,A$I1609,A$I1610,A$I1611,A$I1612), na.rm = T)
A$I16[is.na(A$I16)]<-0
table(A$I16, useNA="always")

### Incarceration (2017)

table(A$E8023701, useNA="always")
table(A$E8023702)
table(A$E8023703)
table(A$E8023704)
table(A$E8023705)
table(A$E8023706)
table(A$E8023707)
table(A$E8023708)
table(A$E8023709)
table(A$E8023710)
table(A$E8023711)
table(A$E8023712)

A$I1701<-NA
A$I1701[A$E8023701==0]<-0
A$I1701[A$E8023701==99]<-0
A$I1701[A$E8023701==1]<-1
A$I1701[is.na(A$I1701) & A$E8043000==-4]<-0
A$I1701[is.na(A$I1701) & A$E8043000==-3]<-0
A$I1701[is.na(A$I1701) & A$E8043000 >= 201800]<-0
A$I1701[is.na(A$I1701) & A$E8043500==0]<-0
A$I1701[is.na(A$I1701) & A$E8043500==-3]<-0
table(A$E8023701, useNA="always")
table(A$I1701, useNA="always")
A$I1702<-NA
A$I1702[A$E8023702==0]<-0
A$I1702[A$E8023702==99]<-0
A$I1702[A$E8023702==1]<-1
A$I1702[is.na(A$I1702) & A$E8043000==-4]<-0
A$I1702[is.na(A$I1702) & A$E8043000==-3]<-0
A$I1702[is.na(A$I1702) & A$E8043000 >= 201600]<-0
A$I1702[is.na(A$I1702) & A$E8043500==0]<-0
A$I1702[is.na(A$I1702) & A$E8043500==-3]<-0
table(A$E8023702, useNA="always")
```

```
table(A$I1702, useNA="always")
A$I1703<-NA
A$I1703[A$E8023703==0]<-0
A$I1703[A$E8023703==99]<-0
A$I1703[A$E8023703==1]<-1
A$I1703[is.na(A$I1703) & A$E8043000==-4]<-0
A$I1703[is.na(A$I1703) & A$E8043000==-3]<-0
A$I1703[is.na(A$I1703) & A$E8043000 >= 201600]<-0
A$I1703[is.na(A$I1703) & A$E8043500==0]<-0
A$I1703[is.na(A$I1703) & A$E8043500==-3]<-0
table(A$E8023703, useNA="always")
table(A$I1703, useNA="always")
A$I1704<-NA
A$I1704[A$E8023704==0]<-0
A$I1704[A$E8023704==99]<-0
A$I1704[A$E8023704==1]<-1
A$I1704[is.na(A$I1704) & A$E8043000==-4]<-0
A$I1704[is.na(A$I1704) & A$E8043000==-3]<-0
A$I1704[is.na(A$I1704) & A$E8043000 >= 201600]<-0
A$I1704[is.na(A$I1704) & A$E8043500==0]<-0
A$I1704[is.na(A$I1704) & A$E8043500==-3]<-0
table(A$E8023704, useNA="always")
table(A$I1704, useNA="always")
A$I1705<-NA
A$I1705[A$E8023705==0]<-0
A$I1705[A$E8023705==99]<-0
A$I1705[A$E8023705==1]<-1
A$I1705[is.na(A$I1705) & A$E8043000==-4]<-0
A$I1705[is.na(A$I1705) & A$E8043000==-3]<-0
A$I1705[is.na(A$I1705) & A$E8043000 >= 201600]<-0
A$I1705[is.na(A$I1705) & A$E8043500==0]<-0
A$I1705[is.na(A$I1705) & A$E8043500==-3]<-0
table(A$E8023705, useNA="always")
table(A$I1705, useNA="always")
A$I1706<-NA
A$I1706[A$E8023706==0]<-0
A$I1706[A$E8023706==99]<-0
A$I1706[A$E8023706==1]<-1
A$I1706[is.na(A$I1706) & A$E8043000==-4]<-0
A$I1706[is.na(A$I1706) & A$E8043000==-3]<-0
A$I1706[is.na(A$I1706) & A$E8043000 >= 201600]<-0
A$I1706[is.na(A$I1706) & A$E8043500==0]<-0
A$I1706[is.na(A$I1706) & A$E8043500==-3]<-0
table(A$E8023706, useNA="always")
table(A$I1706, useNA="always")
A$I1707<-NA
A$I1707[A$E8023707==0]<-0
A$I1707[A$E8023707==99]<-0
A$I1707[A$E8023707==1]<-1
A$I1707[is.na(A$I1707) & A$E8043000==-4]<-0
A$I1707[is.na(A$I1707) & A$E8043000==-3]<-0
A$I1707[is.na(A$I1707) & A$E8043000 >= 201600]<-0
A$I1707[is.na(A$I1707) & A$E8043500==0]<-0
A$I1707[is.na(A$I1707) & A$E8043500==-3]<-0
table(A$E8023707, useNA="always")
table(A$I1707, useNA="always")
```

© 2023 Silver IA et al. *JAMA Network Open.*

```
A$I1708<-NA
A$I1708[A$E8023708==0]<-0
A$I1708[A$E8023708==99]<-0
A$I1708[A$E8023708==1]<-1
A$I1708[is.na(A$I1708) & A$E8043000==-4]<-0
A$I1708[is.na(A$I1708) & A$E8043000==-3]<-0
A$I1708[is.na(A$I1708) & A$E8043000 >= 201600]<-0
A$I1708[is.na(A$I1708) & A$E8043500==0]<-0
A$I1708[is.na(A$I1708) & A$E8043500==-3]<-0
table(A$E8023708, useNA="always")
table(A$I1708, useNA="always")
A$I1709<-NA
A$I1709[A$E8023709==0]<-0
A$I1709[A$E8023709==99]<-0
A$I1709[A$E8023709==1]<-1
A$I1709[is.na(A$I1709) & A$E8043000==-4]<-0
A$I1709[is.na(A$I1709) & A$E8043000==-3]<-0
A$I1709[is.na(A$I1709) & A$E8043000 >= 201600]<-0
A$I1709[is.na(A$I1709) & A$E8043500==0]<-0
A$I1709[is.na(A$I1709) & A$E8043500==-3]<-0
table(A$E8023709, useNA="always")
table(A$I1709, useNA="always")
A$I1710<-NA
A$I1710[A$E8023710==0]<-0
A$I1710[A$E8023710==99]<-0
A$I1710[A$E8023710==1]<-1
A$I1710[is.na(A$I1710) & A$E8043000==-4]<-0
A$I1710[is.na(A$I1710) & A$E8043000==-3]<-0
A$I1710[is.na(A$I1710) & A$E8043000 >= 201600]<-0
A$I1710[is.na(A$I1710) & A$E8043500==0]<-0
A$I1710[is.na(A$I1710) & A$E8043500==-3]<-0
table(A$E8023710, useNA="always")
table(A$I1710, useNA="always")
A$I1711<-NA
A$I1711[A$E8023711==0]<-0
A$I1711[A$E8023711==99]<-0
A$I1711[A$E8023711==1]<-1
A$I1711[is.na(A$I1711) & A$E8043000==-4]<-0
A$I1711[is.na(A$I1711) & A$E8043000==-3]<-0
A$I1711[is.na(A$I1711) & A$E8043000 >= 201600]<-0
A$I1711[is.na(A$I1711) & A$E8043500==0]<-0
A$I1711[is.na(A$I1711) & A$E8043500==-3]<-0
table(A$E8023711, useNA="always")
table(A$I1711, useNA="always")
A$I1712<-NA
A$I1712[A$E8023712==0]<-0
A$I1712[A$E8023712==99]<-0
A$I1712[A$E8023712==1]<-1
A$I1712[is.na(A$I1712) & A$E8043000==-4]<-0
A$I1712[is.na(A$I1712) & A$E8043000==-3]<-0
A$I1712[is.na(A$I1712) & A$E8043000 >= 201600]<-0
A$I1712[is.na(A$I1712) & A$E8043500==0]<-0
A$I1712[is.na(A$I1712) & A$E8043500==-3]<-0
table(A$E8023712, useNA="always")
table(A$I1712, useNA="always")
```

© 2023 Silver IA et al. *JAMA Network Open.*

```
A$I17<-
rowSums(data.frame(A$I1701,A$I1702,A$I1703,A$I1704,A$I1705,A$I1706,A$I1707,A$
I1708,A$I1709,A$I1710,A$I1711,A$I1712), na.rm = T)
A$I17[is.na(A$I17)]<-0
table(A$I17, useNA="always")


# Number of Arrests by Age Measures
## NA_Age7
names(A)
table(A$AGE92)
table(A$AGE93)
A$NA_Age7<-NA
A$NA_Age7[is.na(A$NA_Age7) & A$AGE92==7]<-A$A92[is.na(A$NA_Age7) &
A$AGE92==7]
table(A$NA_Age7,useNA = "always")
A$NA_Age7[A$Dec_Age7==1]<-NA
table(A$NA_Age7,useNA = "always")
## NA_Age8
table(A$AGE92)
table(A$AGE93)
table(A$AGE94)
A$NA_Age8<-NA
A$NA_Age8[is.na(A$NA_Age8) & A$AGE92==8]<-A$A92[is.na(A$NA_Age8) &
A$AGE92==8]
table(A$NA_Age8,useNA = "always")
A$NA_Age8[is.na(A$NA_Age8) & A$AGE93==8]<-A$A93[is.na(A$NA_Age8) &
A$AGE93==8]
table(A$NA_Age8,useNA = "always")
A$NA_Age8[A$Dec_Age8==1]<-NA
table(A$NA_Age8,useNA = "always")
## NA_Age9
table(A$AGE92)
table(A$AGE93)
table(A$AGE94)
A$NA_Age9<-NA
A$NA_Age9[is.na(A$NA_Age9) & A$AGE92==9]<-A$A92[is.na(A$NA_Age9) &
A$AGE92==9]
table(A$NA_Age9,useNA = "always")
A$NA_Age9[is.na(A$NA_Age9) & A$AGE93==9]<-A$A93[is.na(A$NA_Age9) &
A$AGE93==9]
table(A$NA_Age9,useNA = "always")
A$NA_Age9[is.na(A$NA_Age9) & A$AGE94==9]<-A$A94[is.na(A$NA_Age9) &
A$AGE94==9]
table(A$NA_Age9,useNA = "always")
A$NA_Age9[A$Dec_Age9==1]<-NA
table(A$NA_Age9,useNA = "always")
## NA_Age10
table(A$AGE92)
table(A$AGE93)
table(A$AGE94)
table(A$AGE95)
A$NA_Age10<-NA
```

© 2023 Silver IA et al. *JAMA Network Open.*

```
A$NA_Age10[is.na(A$NA_Age10) & A$AGE92==10]<-A$A92[is.na(A$NA_Age10) &
A$AGE92==10]
table(A$NA_Age10,useNA = "always")
A$NA_Age10[is.na(A$NA_Age10) &A$AGE93==10]<-A$A93[is.na(A$NA_Age10)
&A$AGE93==10]
table(A$NA_Age10,useNA = "always")
A$NA_Age10[is.na(A$NA_Age10) &A$AGE94==10]<-A$A94[is.na(A$NA_Age10)
&A$AGE94==10]
table(A$NA_Age10,useNA = "always")
A$NA_Age10[is.na(A$NA_Age10) &A$AGE95==10]<-A$A95[is.na(A$NA_Age10)
&A$AGE95==10]
table(A$NA_Age10,useNA = "always")
A$NA_Age10[A$Dec_Age10==1]<-NA
table(A$NA_Age10,useNA = "always")
## NA_Age11
table(A$AGE92)
table(A$AGE93)
table(A$AGE94)
table(A$AGE95)
table(A$AGE96)
A$NA_Age11<-NA
A$NA_Age11[is.na(A$NA_Age11) & A$AGE92==11]<-A$A92[is.na(A$NA_Age11) &
A$AGE92==11]
table(A$NA_Age11,useNA = "always")
A$NA_Age11[is.na(A$NA_Age11) &A$AGE93==11]<-A$A93[is.na(A$NA_Age11)
&A$AGE93==11]
table(A$NA_Age11,useNA = "always")
A$NA_Age11[is.na(A$NA_Age11) &A$AGE94==11]<-A$A94[is.na(A$NA_Age11)
&A$AGE94==11]
table(A$NA_Age11,useNA = "always")
A$NA_Age11[is.na(A$NA_Age11) &A$AGE95==11]<-A$A95[is.na(A$NA_Age11)
&A$AGE95==11]
table(A$NA_Age11,useNA = "always")
A$NA_Age11[is.na(A$NA_Age11) &A$AGE96==11]<-A$A96[is.na(A$NA_Age11)
&A$AGE96==11]
table(A$NA_Age11,useNA = "always")
A$NA_Age11[A$Dec_Age11==1]<-NA
table(A$NA_Age11,useNA = "always")
## NA_Age12
table(A$AGE92)
table(A$AGE93)
table(A$AGE94)
table(A$AGE95)
table(A$AGE96)
table(A$AGE97)
A$NA_Age12<-NA
A$NA_Age12[is.na(A$NA_Age12) & A$AGE92==12]<-A$A92[is.na(A$NA_Age12) &
A$AGE92==12]
table(A$NA_Age12,useNA = "always")
A$NA_Age12[is.na(A$NA_Age12) &A$AGE93==12]<-A$A93[is.na(A$NA_Age12)
&A$AGE93==12]
table(A$NA_Age12,useNA = "always")
A$NA_Age12[is.na(A$NA_Age12) &A$AGE94==12]<-A$A94[is.na(A$NA_Age12)
&A$AGE94==12]
table(A$NA_Age12,useNA = "always")
```

© 2023 Silver IA et al. *JAMA Network Open.*

```
A$NA_Age12[is.na(A$NA_Age12) &A$AGE95==12]<-A$A95[is.na(A$NA_Age12)
&A$AGE95==12]
table(A$NA_Age12,useNA = "always")
A$NA_Age12[is.na(A$NA_Age12) &A$AGE96==12]<-A$A96[is.na(A$NA_Age12)
&A$AGE96==12]
table(A$NA_Age12,useNA = "always")
A$NA_Age12[is.na(A$NA_Age12) &A$AGE97==12]<-A$A97[is.na(A$NA_Age12)
&A$AGE97==12]
table(A$NA_Age12,useNA = "always")
A$NA_Age12[A$Dec_Age12==1]<-NA
table(A$NA_Age12,useNA = "always")
## NA_Age13
table(A$AGE92)
table(A$AGE93)
table(A$AGE94)
table(A$AGE95)
table(A$AGE96)
table(A$AGE97)
table(A$AGE98)
A$NA_Age13<-NA
A$NA_Age13[is.na(A$NA_Age13) & A$AGE92==13]<-A$A92[is.na(A$NA_Age13) &
A$AGE92==13]
table(A$NA_Age13,useNA = "always")
A$NA_Age13[is.na(A$NA_Age13) &A$AGE93==13]<-A$A93[is.na(A$NA_Age13)
&A$AGE93==13]
table(A$NA_Age13,useNA = "always")
A$NA_Age13[is.na(A$NA_Age13) &A$AGE94==13]<-A$A94[is.na(A$NA_Age13)
&A$AGE94==13]
table(A$NA_Age13,useNA = "always")
A$NA_Age13[is.na(A$NA_Age13) &A$AGE95==13]<-A$A95[is.na(A$NA_Age13)
&A$AGE95==13]
table(A$NA_Age13,useNA = "always")
A$NA_Age13[is.na(A$NA_Age13) &A$AGE96==13]<-A$A96[is.na(A$NA_Age13)
&A$AGE96==13]
table(A$NA_Age13,useNA = "always")
A$NA_Age13[is.na(A$NA_Age13) &A$AGE97==13]<-A$A97[is.na(A$NA_Age13)
&A$AGE97==13]
table(A$NA_Age13,useNA = "always")
A$NA_Age13[is.na(A$NA_Age13) &A$AGE98==13]<-A$A98[is.na(A$NA_Age13)
&A$AGE98==13]
table(A$NA_Age13,useNA = "always")
A$NA_Age13[A$Dec_Age13==1]<-NA
table(A$NA_Age13,useNA = "always")
## NA_Age14
table(A$AGE92)
table(A$AGE93)
table(A$AGE94)
table(A$AGE95)
table(A$AGE96)
table(A$AGE97)
table(A$AGE98)
table(A$AGE99)
A$NA_Age14<-NA
A$NA_Age14[is.na(A$NA_Age14) & A$AGE92==14]<-A$A92[is.na(A$NA_Age14) &
A$AGE92==14]
table(A$NA_Age14,useNA = "always")
```

© 2023 Silver IA et al. *JAMA Network Open.*

```
A$NA_Age14[is.na(A$NA_Age14) &A$AGE93==14]<-A$A93[is.na(A$NA_Age14)
&A$AGE93==14]
table(A$NA_Age14,useNA = "always")
A$NA_Age14[is.na(A$NA_Age14) &A$AGE94==14]<-A$A94[is.na(A$NA_Age14)
&A$AGE94==14]
table(A$NA_Age14,useNA = "always")
A$NA_Age14[is.na(A$NA_Age14) &A$AGE95==14]<-A$A95[is.na(A$NA_Age14)
&A$AGE95==14]
table(A$NA_Age14,useNA = "always")
A$NA_Age14[is.na(A$NA_Age14) &A$AGE96==14]<-A$A96[is.na(A$NA_Age14)
&A$AGE96==14]
table(A$NA_Age14,useNA = "always")
A$NA_Age14[is.na(A$NA_Age14) &A$AGE97==14]<-A$A97[is.na(A$NA_Age14)
&A$AGE97==14]
table(A$NA_Age14,useNA = "always")
A$NA_Age14[is.na(A$NA_Age14) &A$AGE98==14]<-A$A98[is.na(A$NA_Age14)
&A$AGE98==14]
table(A$NA_Age14,useNA = "always")
A$NA_Age14[is.na(A$NA_Age14) &A$AGE99==14]<-A$A99[is.na(A$NA_Age14)
&A$AGE99==14]
table(A$NA_Age14,useNA = "always")
A$NA_Age14[A$Dec_Age14==1]<-NA
table(A$NA_Age14,useNA = "always")
## NA_Age15
table(A$AGE92)
table(A$AGE93)
table(A$AGE94)
table(A$AGE95)
table(A$AGE96)
table(A$AGE97)
table(A$AGE98)
table(A$AGE99)
table(A$AGE00)
A$NA_Age15<-NA
A$NA_Age15[is.na(A$NA_Age15) & A$AGE92==15]<-A$A92[is.na(A$NA_Age15) &
A$AGE92==15]
table(A$NA_Age15,useNA = "always")
A$NA_Age15[is.na(A$NA_Age15) &A$AGE93==15]<-A$A93[is.na(A$NA_Age15)
&A$AGE93==15]
table(A$NA_Age15,useNA = "always")
A$NA_Age15[is.na(A$NA_Age15) &A$AGE94==15]<-A$A94[is.na(A$NA_Age15)
&A$AGE94==15]
table(A$NA_Age15,useNA = "always")
A$NA_Age15[is.na(A$NA_Age15) &A$AGE95==15]<-A$A95[is.na(A$NA_Age15)
&A$AGE95==15]
table(A$NA_Age15,useNA = "always")
A$NA_Age15[is.na(A$NA_Age15) &A$AGE96==15]<-A$A96[is.na(A$NA_Age15)
&A$AGE96==15]
table(A$NA_Age15,useNA = "always")
A$NA_Age15[is.na(A$NA_Age15) &A$AGE97==15]<-A$A97[is.na(A$NA_Age15)
&A$AGE97==15]
table(A$NA_Age15,useNA = "always")
A$NA_Age15[is.na(A$NA_Age15) &A$AGE98==15]<-A$A98[is.na(A$NA_Age15)
&A$AGE98==15]
table(A$NA_Age15,useNA = "always")
```

© 2023 Silver IA et al. *JAMA Network Open.*

```
A$NA_Age15[is.na(A$NA_Age15) &A$AGE99==15]<-A$A99[is.na(A$NA_Age15)
&A$AGE99==15]
table(A$NA_Age15,useNA = "always")
A$NA_Age15[is.na(A$NA_Age15) & A$AGE00==15]<-A$A00[is.na(A$NA_Age15)
&A$AGE00==15]
table(A$NA_Age15,useNA = "always")
A$NA_Age15[A$Dec_Age15==1]<-NA
table(A$NA_Age15,useNA = "always")
## NA_Age16
table(A$AGE92)
table(A$AGE93)
table(A$AGE94)
table(A$AGE95)
table(A$AGE96)
table(A$AGE97)
table(A$AGE98)
table(A$AGE99)
table(A$AGE00)
table(A$AGE01)
A$NA_Age16<-NA
A$NA_Age16[is.na(A$NA_Age16) & A$AGE92==16]<-A$A92[is.na(A$NA_Age16) &
A$AGE92==16]
table(A$NA_Age16,useNA = "always")
A$NA_Age16[is.na(A$NA_Age16) &A$AGE93==16]<-A$A93[is.na(A$NA_Age16)
&A$AGE93==16]
table(A$NA_Age16,useNA = "always")
A$NA_Age16[is.na(A$NA_Age16) &A$AGE94==16]<-A$A94[is.na(A$NA_Age16)
&A$AGE94==16]
table(A$NA_Age16,useNA = "always")
A$NA_Age16[is.na(A$NA_Age16) &A$AGE95==16]<-A$A95[is.na(A$NA_Age16)
&A$AGE95==16]
table(A$NA_Age16,useNA = "always")
A$NA_Age16[is.na(A$NA_Age16) &A$AGE96==16]<-A$A96[is.na(A$NA_Age16)
&A$AGE96==16]
table(A$NA_Age16,useNA = "always")
A$NA_Age16[is.na(A$NA_Age16) &A$AGE97==16]<-A$A97[is.na(A$NA_Age16)
&A$AGE97==16]
table(A$NA_Age16,useNA = "always")
A$NA_Age16[is.na(A$NA_Age16) &A$AGE98==16]<-A$A98[is.na(A$NA_Age16)
&A$AGE98==16]
table(A$NA_Age16,useNA = "always")
A$NA_Age16[is.na(A$NA_Age16) &A$AGE99==16]<-A$A99[is.na(A$NA_Age16)
&A$AGE99==16]
table(A$NA_Age16,useNA = "always")
A$NA_Age16[is.na(A$NA_Age16) & A$AGE00==16]<-A$A00[is.na(A$NA_Age16)
&A$AGE00==16]
table(A$NA_Age16,useNA = "always")
A$NA_Age16[is.na(A$NA_Age16) & A$AGE01==16]<-A$A01[is.na(A$NA_Age16)
&A$AGE01==16]
table(A$NA_Age16,useNA = "always")
A$NA_Age16[A$Dec_Age16==1]<-NA
table(A$NA_Age16,useNA = "always")
## NA_Age17
table(A$AGE92)
table(A$AGE93)
table(A$AGE94)
```

© 2023 Silver IA et al. *JAMA Network Open.*

```
table(A$AGE95)
table(A$AGE96)
table(A$AGE97)
table(A$AGE98)
table(A$AGE99)
table(A$AGE00)
table(A$AGE01)
A$NA_Age17<-NA
A$NA_Age17[is.na(A$NA_Age17) & A$AGE92==17]<-A$A92[is.na(A$NA_Age17) &
A$AGE92==17]
table(A$NA_Age17,useNA = "always")
A$NA_Age17[is.na(A$NA_Age17) &A$AGE93==17]<-A$A93[is.na(A$NA_Age17)
&A$AGE93==17]
table(A$NA_Age17,useNA = "always")
A$NA_Age17[is.na(A$NA_Age17) &A$AGE94==17]<-A$A94[is.na(A$NA_Age17)
&A$AGE94==17]
table(A$NA_Age17,useNA = "always")
A$NA_Age17[is.na(A$NA_Age17) &A$AGE95==17]<-A$A95[is.na(A$NA_Age17)
&A$AGE95==17]
table(A$NA_Age17,useNA = "always")
A$NA_Age17[is.na(A$NA_Age17) &A$AGE96==17]<-A$A96[is.na(A$NA_Age17)
&A$AGE96==17]
table(A$NA_Age17,useNA = "always")
A$NA_Age17[is.na(A$NA_Age17) &A$AGE97==17]<-A$A97[is.na(A$NA_Age17)
&A$AGE97==17]
table(A$NA_Age17,useNA = "always")
A$NA_Age17[is.na(A$NA_Age17) &A$AGE98==17]<-A$A98[is.na(A$NA_Age17)
&A$AGE98==17]
table(A$NA_Age17,useNA = "always")
A$NA_Age17[is.na(A$NA_Age17) &A$AGE99==17]<-A$A99[is.na(A$NA_Age17)
&A$AGE99==17]
table(A$NA_Age17,useNA = "always")
A$NA_Age17[is.na(A$NA_Age17) & A$AGE00==17]<-A$A00[is.na(A$NA_Age17)
&A$AGE00==17]
table(A$NA_Age17,useNA = "always")
A$NA_Age17[is.na(A$NA_Age17) & A$AGE01==17]<-A$A01[is.na(A$NA_Age17)
&A$AGE01==17]
table(A$NA_Age17,useNA = "always")
A$NA_Age17[is.na(A$NA_Age17) & A$AGE02==17]<-A$A02[is.na(A$NA_Age17)
&A$AGE02==17]
table(A$NA_Age17,useNA = "always")
A$NA_Age17[A$Dec_Age17==1]<-NA
table(A$NA_Age17,useNA = "always")
## NA_Age18
table(A$AGE92)
table(A$AGE93)
table(A$AGE94)
table(A$AGE95)
table(A$AGE96)
table(A$AGE97)
table(A$AGE98)
table(A$AGE99)
table(A$AGE00)
table(A$AGE01)
A$NA_Age18<-NA
```

```
A$NA_Age18[is.na(A$NA_Age18) & A$AGE92==18]<-A$A92[is.na(A$NA_Age18) &
A$AGE92==18]
table(A$NA_Age18,useNA = "always")
A$NA_Age18[is.na(A$NA_Age18) &A$AGE93==18]<-A$A93[is.na(A$NA_Age18)
&A$AGE93==18]
table(A$NA_Age18,useNA = "always")
A$NA_Age18[is.na(A$NA_Age18) &A$AGE94==18]<-A$A94[is.na(A$NA_Age18)
&A$AGE94==18]
table(A$NA_Age18,useNA = "always")
A$NA_Age18[is.na(A$NA_Age18) &A$AGE95==18]<-A$A95[is.na(A$NA_Age18)
&A$AGE95==18]
table(A$NA_Age18,useNA = "always")
A$NA_Age18[is.na(A$NA_Age18) &A$AGE96==18]<-A$A96[is.na(A$NA_Age18)
&A$AGE96==18]
table(A$NA_Age18,useNA = "always")
A$NA_Age18[is.na(A$NA_Age18) &A$AGE97==18]<-A$A97[is.na(A$NA_Age18)
&A$AGE97==18]
table(A$NA_Age18,useNA = "always")
A$NA_Age18[is.na(A$NA_Age18) &A$AGE98==18]<-A$A98[is.na(A$NA_Age18)
&A$AGE98==18]
table(A$NA_Age18,useNA = "always")
A$NA_Age18[is.na(A$NA_Age18) &A$AGE99==18]<-A$A99[is.na(A$NA_Age18)
&A$AGE99==18]
table(A$NA_Age18,useNA = "always")
A$NA_Age18[is.na(A$NA_Age18) & A$AGE00==18]<-A$A00[is.na(A$NA_Age18)
&A$AGE00==18]
table(A$NA_Age18,useNA = "always")
A$NA_Age18[is.na(A$NA_Age18) & A$AGE01==18]<-A$A01[is.na(A$NA_Age18)
&A$AGE01==18]
table(A$NA_Age18,useNA = "always")
A$NA_Age18[is.na(A$NA_Age18) & A$AGE02==18]<-A$A02[is.na(A$NA_Age18)
&A$AGE02==18]
table(A$NA_Age18,useNA = "always")
A$NA_Age18[is.na(A$NA_Age18) & A$AGE03==18]<-A$A03[is.na(A$NA_Age18)
&A$AGE03==18]
table(A$NA_Age18,useNA = "always")
A$NA_Age18[A$Dec_Age18==1]<-NA
table(A$NA_Age18,useNA = "always")
## NA_Age19
A$NA_Age19<-NA
A$NA_Age19[is.na(A$NA_Age19) & A$AGE92==19]<-A$A92[is.na(A$NA_Age19) &
A$AGE92==19]
table(A$NA_Age19,useNA = "always")
A$NA_Age19[is.na(A$NA_Age19) &A$AGE93==19]<-A$A93[is.na(A$NA_Age19)
&A$AGE93==19]
table(A$NA_Age19,useNA = "always")
A$NA_Age19[is.na(A$NA_Age19) &A$AGE94==19]<-A$A94[is.na(A$NA_Age19)
&A$AGE94==19]
table(A$NA_Age19,useNA = "always")
A$NA_Age19[is.na(A$NA_Age19) &A$AGE95==19]<-A$A95[is.na(A$NA_Age19)
&A$AGE95==19]
table(A$NA_Age19,useNA = "always")
A$NA_Age19[is.na(A$NA_Age19) &A$AGE96==19]<-A$A96[is.na(A$NA_Age19)
&A$AGE96==19]
table(A$NA_Age19,useNA = "always")
```

© 2023 Silver IA et al. *JAMA Network Open.*

```
A$NA_Age19[is.na(A$NA_Age19) &A$AGE97==19]<-A$A97[is.na(A$NA_Age19)
&A$AGE97==19]
table(A$NA_Age19,useNA = "always")
A$NA_Age19[is.na(A$NA_Age19) &A$AGE98==19]<-A$A98[is.na(A$NA_Age19)
&A$AGE98==19]
table(A$NA_Age19,useNA = "always")
A$NA_Age19[is.na(A$NA_Age19) &A$AGE99==19]<-A$A99[is.na(A$NA_Age19)
&A$AGE99==19]
table(A$NA_Age19,useNA = "always")
A$NA_Age19[is.na(A$NA_Age19) & A$AGE00==19]<-A$A00[is.na(A$NA_Age19)
&A$AGE00==19]
table(A$NA_Age19,useNA = "always")
A$NA_Age19[is.na(A$NA_Age19) & A$AGE01==19]<-A$A01[is.na(A$NA_Age19)
&A$AGE01==19]
table(A$NA_Age19,useNA = "always")
A$NA_Age19[is.na(A$NA_Age19) & A$AGE02==19]<-A$A02[is.na(A$NA_Age19)
&A$AGE02==19]
table(A$NA_Age19,useNA = "always")
A$NA_Age19[is.na(A$NA_Age19) & A$AGE03==19]<-A$A03[is.na(A$NA_Age19)
&A$AGE03==19]
table(A$NA_Age19,useNA = "always")
A$NA_Age19[is.na(A$NA_Age19) & A$AGE04==19]<-A$A04[is.na(A$NA_Age19)
&A$AGE04==19]
table(A$NA_Age19,useNA = "always")
A$NA_Age19[A$Dec_Age19==1]<-NA
table(A$NA_Age19,useNA = "always")
## NA_Age20
A$NA_Age20<-NA
A$NA_Age20[is.na(A$NA_Age20) & A$AGE92==20]<-A$A92[is.na(A$NA_Age20) &
A$AGE92==20]
table(A$NA_Age20,useNA = "always")
A$NA_Age20[is.na(A$NA_Age20) &A$AGE93==20]<-A$A93[is.na(A$NA_Age20)
&A$AGE93==20]
table(A$NA_Age20,useNA = "always")
A$NA_Age20[is.na(A$NA_Age20) &A$AGE94==20]<-A$A94[is.na(A$NA_Age20)
&A$AGE94==20]
table(A$NA_Age20,useNA = "always")
A$NA_Age20[is.na(A$NA_Age20) &A$AGE95==20]<-A$A95[is.na(A$NA_Age20)
&A$AGE95==20]
table(A$NA_Age20,useNA = "always")
A$NA_Age20[is.na(A$NA_Age20) &A$AGE96==20]<-A$A96[is.na(A$NA_Age20)
&A$AGE96==20]
table(A$NA_Age20,useNA = "always")
A$NA_Age20[is.na(A$NA_Age20) &A$AGE97==20]<-A$A97[is.na(A$NA_Age20)
&A$AGE97==20]
table(A$NA_Age20,useNA = "always")
A$NA_Age20[is.na(A$NA_Age20) &A$AGE98==20]<-A$A98[is.na(A$NA_Age20)
&A$AGE98==20]
table(A$NA_Age20,useNA = "always")
A$NA_Age20[is.na(A$NA_Age20) &A$AGE99==20]<-A$A99[is.na(A$NA_Age20)
&A$AGE99==20]
table(A$NA_Age20,useNA = "always")
A$NA_Age20[is.na(A$NA_Age20) & A$AGE00==20]<-A$A00[is.na(A$NA_Age20)
&A$AGE00==20]
table(A$NA_Age20,useNA = "always")
```

© 2023 Silver IA et al. *JAMA Network Open.*

```
A$NA_Age20[is.na(A$NA_Age20) & A$AGE01==20]<-A$A01[is.na(A$NA_Age20)
&A$AGE01==20]
table(A$NA_Age20,useNA = "always")
A$NA_Age20[is.na(A$NA_Age20) & A$AGE02==20]<-A$A02[is.na(A$NA_Age20)
&A$AGE02==20]
table(A$NA_Age20,useNA = "always")
A$NA_Age20[is.na(A$NA_Age20) & A$AGE03==20]<-A$A03[is.na(A$NA_Age20)
&A$AGE03==20]
table(A$NA_Age20,useNA = "always")
A$NA_Age20[is.na(A$NA_Age20) & A$AGE04==20]<-A$A04[is.na(A$NA_Age20)
&A$AGE04==20]
table(A$NA_Age20,useNA = "always")
A$NA_Age20[is.na(A$NA_Age20) & A$AGE05==20]<-A$A05[is.na(A$NA_Age20)
&A$AGE05==20]
table(A$NA_Age20,useNA = "always")
A$NA_Age20[A$Dec_Age20==1]<-NA
table(A$NA_Age20,useNA = "always")
## NA_Age21
A$NA_Age21<-NA
A$NA_Age21[is.na(A$NA_Age21) & A$AGE92==21]<-A$A92[is.na(A$NA_Age21) &
A$AGE92==21]
table(A$NA_Age21,useNA = "always")
A$NA_Age21[is.na(A$NA_Age21) &A$AGE93==21]<-A$A93[is.na(A$NA_Age21)
&A$AGE93==21]
table(A$NA_Age21,useNA = "always")
A$NA_Age21[is.na(A$NA_Age21) &A$AGE94==21]<-A$A94[is.na(A$NA_Age21)
&A$AGE94==21]
table(A$NA_Age21,useNA = "always")
A$NA_Age21[is.na(A$NA_Age21) &A$AGE95==21]<-A$A95[is.na(A$NA_Age21)
&A$AGE95==21]
table(A$NA_Age21,useNA = "always")
A$NA_Age21[is.na(A$NA_Age21) &A$AGE96==21]<-A$A96[is.na(A$NA_Age21)
&A$AGE96==21]
table(A$NA_Age21,useNA = "always")
A$NA_Age21[is.na(A$NA_Age21) &A$AGE97==21]<-A$A97[is.na(A$NA_Age21)
&A$AGE97==21]
table(A$NA_Age21,useNA = "always")
A$NA_Age21[is.na(A$NA_Age21) &A$AGE98==21]<-A$A98[is.na(A$NA_Age21)
&A$AGE98==21]
table(A$NA_Age21,useNA = "always")
A$NA_Age21[is.na(A$NA_Age21) &A$AGE99==21]<-A$A99[is.na(A$NA_Age21)
&A$AGE99==21]
table(A$NA_Age21,useNA = "always")
A$NA_Age21[is.na(A$NA_Age21) & A$AGE00==21]<-A$A00[is.na(A$NA_Age21)
&A$AGE00==21]
table(A$NA_Age21,useNA = "always")
A$NA_Age21[is.na(A$NA_Age21) & A$AGE01==21]<-A$A01[is.na(A$NA_Age21)
&A$AGE01==21]
table(A$NA_Age21,useNA = "always")
A$NA_Age21[is.na(A$NA_Age21) & A$AGE02==21]<-A$A02[is.na(A$NA_Age21)
&A$AGE02==21]
table(A$NA_Age21,useNA = "always")
A$NA_Age21[is.na(A$NA_Age21) & A$AGE03==21]<-A$A03[is.na(A$NA_Age21)
&A$AGE03==21]
table(A$NA_Age21,useNA = "always")
```

© 2023 Silver IA et al. *JAMA Network Open.*

```
A$NA_Age21[is.na(A$NA_Age21) & A$AGE04==21]<-A$A04[is.na(A$NA_Age21)
&A$AGE04==21]
table(A$NA_Age21,useNA = "always")
A$NA_Age21[is.na(A$NA_Age21) & A$AGE05==21]<-A$A05[is.na(A$NA_Age21)
&A$AGE05==21]
table(A$NA_Age21,useNA = "always")
A$NA_Age21[is.na(A$NA_Age21) & A$AGE06==21]<-A$A06[is.na(A$NA_Age21)
&A$AGE06==21]
table(A$NA_Age21,useNA = "always")
A$NA_Age21[A$Dec_Age21==1]<-NA
table(A$NA_Age21,useNA = "always")
## NA_Age22
A$NA_Age22<-NA
A$NA_Age22[is.na(A$NA_Age22) & A$AGE92==22]<-A$A92[is.na(A$NA_Age22) &
A$AGE92==22]
table(A$NA_Age22,useNA = "always")
A$NA_Age22[is.na(A$NA_Age22) &A$AGE93==22]<-A$A93[is.na(A$NA_Age22)
&A$AGE93==22]
table(A$NA_Age22,useNA = "always")
A$NA_Age22[is.na(A$NA_Age22) &A$AGE94==22]<-A$A94[is.na(A$NA_Age22)
&A$AGE94==22]
table(A$NA_Age22,useNA = "always")
A$NA_Age22[is.na(A$NA_Age22) &A$AGE95==22]<-A$A95[is.na(A$NA_Age22)
&A$AGE95==22]
table(A$NA_Age22,useNA = "always")
A$NA_Age22[is.na(A$NA_Age22) &A$AGE96==22]<-A$A96[is.na(A$NA_Age22)
&A$AGE96==22]
table(A$NA_Age22,useNA = "always")
A$NA_Age22[is.na(A$NA_Age22) &A$AGE97==22]<-A$A97[is.na(A$NA_Age22)
&A$AGE97==22]
table(A$NA_Age22,useNA = "always")
A$NA_Age22[is.na(A$NA_Age22) &A$AGE98==22]<-A$A98[is.na(A$NA_Age22)
&A$AGE98==22]
table(A$NA_Age22,useNA = "always")
A$NA_Age22[is.na(A$NA_Age22) &A$AGE99==22]<-A$A99[is.na(A$NA_Age22)
&A$AGE99==22]
table(A$NA_Age22,useNA = "always")
A$NA_Age22[is.na(A$NA_Age22) & A$AGE00==22]<-A$A00[is.na(A$NA_Age22)
&A$AGE00==22]
table(A$NA_Age22,useNA = "always")
A$NA_Age22[is.na(A$NA_Age22) & A$AGE01==22]<-A$A01[is.na(A$NA_Age22)
&A$AGE01==22]
table(A$NA_Age22,useNA = "always")
A$NA_Age22[is.na(A$NA_Age22) & A$AGE02==22]<-A$A02[is.na(A$NA_Age22)
&A$AGE02==22]
table(A$NA_Age22,useNA = "always")
A$NA_Age22[is.na(A$NA_Age22) & A$AGE03==22]<-A$A03[is.na(A$NA_Age22)
&A$AGE03==22]
table(A$NA_Age22,useNA = "always")
A$NA_Age22[is.na(A$NA_Age22) & A$AGE04==22]<-A$A04[is.na(A$NA_Age22)
&A$AGE04==22]
table(A$NA_Age22,useNA = "always")
A$NA_Age22[is.na(A$NA_Age22) & A$AGE05==22]<-A$A05[is.na(A$NA_Age22)
&A$AGE05==22]
table(A$NA_Age22,useNA = "always")
```

© 2023 Silver IA et al. *JAMA Network Open.*

```
A$NA_Age22[is.na(A$NA_Age22) & A$AGE06==22]<-A$A06[is.na(A$NA_Age22)
&A$AGE06==22]
table(A$NA_Age22,useNA = "always")
A$NA_Age22[is.na(A$NA_Age22) & A$AGE07==22]<-A$A07[is.na(A$NA_Age22)
&A$AGE07==22]
table(A$NA_Age22,useNA = "always")
A$NA_Age22[A$Dec_Age22==1]<-NA
table(A$NA_Age22,useNA = "always")
## NA_Age23
A$NA_Age23<-NA
A$NA_Age23[is.na(A$NA_Age23) & A$AGE92==23]<-A$A92[is.na(A$NA_Age23) &
A$AGE92==23]
table(A$NA_Age23,useNA = "always")
A$NA_Age23[is.na(A$NA_Age23) &A$AGE93==23]<-A$A93[is.na(A$NA_Age23)
&A$AGE93==23]
table(A$NA_Age23,useNA = "always")
A$NA_Age23[is.na(A$NA_Age23) &A$AGE94==23]<-A$A94[is.na(A$NA_Age23)
&A$AGE94==23]
table(A$NA_Age23,useNA = "always")
A$NA_Age23[is.na(A$NA_Age23) &A$AGE95==23]<-A$A95[is.na(A$NA_Age23)
&A$AGE95==23]
table(A$NA_Age23,useNA = "always")
A$NA_Age23[is.na(A$NA_Age23) &A$AGE96==23]<-A$A96[is.na(A$NA_Age23)
&A$AGE96==23]
table(A$NA_Age23,useNA = "always")
A$NA_Age23[is.na(A$NA_Age23) &A$AGE97==23]<-A$A97[is.na(A$NA_Age23)
&A$AGE97==23]
table(A$NA_Age23,useNA = "always")
A$NA_Age23[is.na(A$NA_Age23) &A$AGE98==23]<-A$A98[is.na(A$NA_Age23)
&A$AGE98==23]
table(A$NA_Age23,useNA = "always")
A$NA_Age23[is.na(A$NA_Age23) &A$AGE99==23]<-A$A99[is.na(A$NA_Age23)
&A$AGE99==23]
table(A$NA_Age23,useNA = "always")
A$NA_Age23[is.na(A$NA_Age23) & A$AGE00==23]<-A$A00[is.na(A$NA_Age23)
&A$AGE00==23]
table(A$NA_Age23,useNA = "always")
A$NA_Age23[is.na(A$NA_Age23) & A$AGE01==23]<-A$A01[is.na(A$NA_Age23)
&A$AGE01==23]
table(A$NA_Age23,useNA = "always")
A$NA_Age23[is.na(A$NA_Age23) & A$AGE02==23]<-A$A02[is.na(A$NA_Age23)
&A$AGE02==23]
table(A$NA_Age23,useNA = "always")
A$NA_Age23[is.na(A$NA_Age23) & A$AGE03==23]<-A$A03[is.na(A$NA_Age23)
&A$AGE03==23]
table(A$NA_Age23,useNA = "always")
A$NA_Age23[is.na(A$NA_Age23) & A$AGE04==23]<-A$A04[is.na(A$NA_Age23)
&A$AGE04==23]
table(A$NA_Age23,useNA = "always")
A$NA_Age23[is.na(A$NA_Age23) & A$AGE05==23]<-A$A05[is.na(A$NA_Age23)
&A$AGE05==23]
table(A$NA_Age23,useNA = "always")
A$NA_Age23[is.na(A$NA_Age23) & A$AGE06==23]<-A$A06[is.na(A$NA_Age23)
&A$AGE06==23]
table(A$NA_Age23,useNA = "always")
```

© 2023 Silver IA et al. *JAMA Network Open.*

```
A$NA_Age23[is.na(A$NA_Age23) & A$AGE07==23]<-A$A07[is.na(A$NA_Age23)
&A$AGE07==23]
table(A$NA_Age23,useNA = "always")
A$NA_Age23[is.na(A$NA_Age23) & A$AGE08==23]<-A$A08[is.na(A$NA_Age23)
&A$AGE08==23]
table(A$NA_Age23,useNA = "always")
A$NA_Age23[A$Dec_Age23==1]<-NA
table(A$NA_Age23,useNA = "always")
## NA_Age24
A$NA_Age24<-NA
A$NA_Age24[is.na(A$NA_Age24) & A$AGE92==24]<-A$A92[is.na(A$NA_Age24) &
A$AGE92==24]
table(A$NA_Age24,useNA = "always")
A$NA_Age24[is.na(A$NA_Age24) &A$AGE93==24]<-A$A93[is.na(A$NA_Age24)
&A$AGE93==24]
table(A$NA_Age24,useNA = "always")
A$NA_Age24[is.na(A$NA_Age24) &A$AGE94==24]<-A$A94[is.na(A$NA_Age24)
&A$AGE94==24]
table(A$NA_Age24,useNA = "always")
A$NA_Age24[is.na(A$NA_Age24) &A$AGE95==24]<-A$A95[is.na(A$NA_Age24)
&A$AGE95==24]
table(A$NA_Age24,useNA = "always")
A$NA_Age24[is.na(A$NA_Age24) &A$AGE96==24]<-A$A96[is.na(A$NA_Age24)
&A$AGE96==24]
table(A$NA_Age24,useNA = "always")
A$NA_Age24[is.na(A$NA_Age24) &A$AGE97==24]<-A$A97[is.na(A$NA_Age24)
&A$AGE97==24]
table(A$NA_Age24,useNA = "always")
A$NA_Age24[is.na(A$NA_Age24) &A$AGE98==24]<-A$A98[is.na(A$NA_Age24)
&A$AGE98==24]
table(A$NA_Age24,useNA = "always")
A$NA_Age24[is.na(A$NA_Age24) &A$AGE99==24]<-A$A99[is.na(A$NA_Age24)
&A$AGE99==24]
table(A$NA_Age24,useNA = "always")
A$NA_Age24[is.na(A$NA_Age24) & A$AGE00==24]<-A$A00[is.na(A$NA_Age24)
&A$AGE00==24]
table(A$NA_Age24,useNA = "always")
A$NA_Age24[is.na(A$NA_Age24) & A$AGE01==24]<-A$A01[is.na(A$NA_Age24)
&A$AGE01==24]
table(A$NA_Age24,useNA = "always")
A$NA_Age24[is.na(A$NA_Age24) & A$AGE02==24]<-A$A02[is.na(A$NA_Age24)
&A$AGE02==24]
table(A$NA_Age24,useNA = "always")
A$NA_Age24[is.na(A$NA_Age24) & A$AGE03==24]<-A$A03[is.na(A$NA_Age24)
&A$AGE03==24]
table(A$NA_Age24,useNA = "always")
A$NA_Age24[is.na(A$NA_Age24) & A$AGE04==24]<-A$A04[is.na(A$NA_Age24)
&A$AGE04==24]
table(A$NA_Age24,useNA = "always")
A$NA_Age24[is.na(A$NA_Age24) & A$AGE05==24]<-A$A05[is.na(A$NA_Age24)
&A$AGE05==24]
table(A$NA_Age24,useNA = "always")
A$NA_Age24[is.na(A$NA_Age24) & A$AGE06==24]<-A$A06[is.na(A$NA_Age24)
&A$AGE06==24]
table(A$NA_Age24,useNA = "always")
```

© 2023 Silver IA et al. *JAMA Network Open.*

```
A$NA_Age24[is.na(A$NA_Age24) & A$AGE07==24]<-A$A07[is.na(A$NA_Age24)
&A$AGE07==24]
table(A$NA_Age24,useNA = "always")
A$NA_Age24[is.na(A$NA_Age24) & A$AGE08==24]<-A$A08[is.na(A$NA_Age24)
&A$AGE08==24]
table(A$NA_Age24,useNA = "always")
A$NA_Age24[is.na(A$NA_Age24) & A$AGE09==24]<-A$A09[is.na(A$NA_Age24)
&A$AGE09==24]
table(A$NA_Age24,useNA = "always")
A$NA_Age24[A$Dec_Age24==1]<-NA
table(A$NA_Age24,useNA = "always")
## NA_Age25

A$NA_Age25<-NA
A$NA_Age25[is.na(A$NA_Age25) & A$AGE92==25]<-A$A92[is.na(A$NA_Age25) &
A$AGE92==25]
table(A$NA_Age25,useNA = "always")
A$NA_Age25[is.na(A$NA_Age25) &A$AGE93==25]<-A$A93[is.na(A$NA_Age25)
&A$AGE93==25]
table(A$NA_Age25,useNA = "always")
A$NA_Age25[is.na(A$NA_Age25) &A$AGE94==25]<-A$A94[is.na(A$NA_Age25)
&A$AGE94==25]
table(A$NA_Age25,useNA = "always")
A$NA_Age25[is.na(A$NA_Age25) &A$AGE95==25]<-A$A95[is.na(A$NA_Age25)
&A$AGE95==25]
table(A$NA_Age25,useNA = "always")
A$NA_Age25[is.na(A$NA_Age25) &A$AGE96==25]<-A$A96[is.na(A$NA_Age25)
&A$AGE96==25]
table(A$NA_Age25,useNA = "always")
A$NA_Age25[is.na(A$NA_Age25) &A$AGE97==25]<-A$A97[is.na(A$NA_Age25)
&A$AGE97==25]
table(A$NA_Age25,useNA = "always")
A$NA_Age25[is.na(A$NA_Age25) &A$AGE98==25]<-A$A98[is.na(A$NA_Age25)
&A$AGE98==25]
table(A$NA_Age25,useNA = "always")
A$NA_Age25[is.na(A$NA_Age25) &A$AGE99==25]<-A$A99[is.na(A$NA_Age25)
&A$AGE99==25]
table(A$NA_Age25,useNA = "always")
A$NA_Age25[is.na(A$NA_Age25) & A$AGE00==25]<-A$A00[is.na(A$NA_Age25)
&A$AGE00==25]
table(A$NA_Age25,useNA = "always")
A$NA_Age25[is.na(A$NA_Age25) & A$AGE01==25]<-A$A01[is.na(A$NA_Age25)
&A$AGE01==25]
table(A$NA_Age25,useNA = "always")
A$NA_Age25[is.na(A$NA_Age25) & A$AGE02==25]<-A$A02[is.na(A$NA_Age25)
&A$AGE02==25]
table(A$NA_Age25,useNA = "always")
A$NA_Age25[is.na(A$NA_Age25) & A$AGE03==25]<-A$A03[is.na(A$NA_Age25)
&A$AGE03==25]
table(A$NA_Age25,useNA = "always")
A$NA_Age25[is.na(A$NA_Age25) & A$AGE04==25]<-A$A04[is.na(A$NA_Age25)
&A$AGE04==25]
table(A$NA_Age25,useNA = "always")
A$NA_Age25[is.na(A$NA_Age25) & A$AGE05==25]<-A$A05[is.na(A$NA_Age25)
&A$AGE05==25]
table(A$NA_Age25,useNA = "always")
```

© 2023 Silver IA et al. *JAMA Network Open.*

```
A$NA_Age25[is.na(A$NA_Age25) & A$AGE06==25]<-A$A06[is.na(A$NA_Age25)
&A$AGE06==25]
table(A$NA_Age25,useNA = "always")
A$NA_Age25[is.na(A$NA_Age25) & A$AGE07==25]<-A$A07[is.na(A$NA_Age25)
&A$AGE07==25]
table(A$NA_Age25,useNA = "always")
A$NA_Age25[is.na(A$NA_Age25) & A$AGE08==25]<-A$A08[is.na(A$NA_Age25)
&A$AGE08==25]
table(A$NA_Age25,useNA = "always")
A$NA_Age25[is.na(A$NA_Age25) & A$AGE09==25]<-A$A09[is.na(A$NA_Age25)
&A$AGE09==25]
table(A$NA_Age25,useNA = "always")
A$NA_Age25[is.na(A$NA_Age25) & A$AGE10==25]<-A$A10[is.na(A$NA_Age25)
&A$AGE10==25]
table(A$NA_Age25,useNA = "always")
A$NA_Age25[A$Dec_Age25==1]<-NA
table(A$NA_Age25,useNA = "always")
## NA_Age26
table(A$AGE11)
A$NA_Age26<-NA
A$NA_Age26[is.na(A$NA_Age26) & A$AGE92==26]<-A$A92[is.na(A$NA_Age26) &
A$AGE92==26]
table(A$NA_Age26,useNA = "always")
A$NA_Age26[is.na(A$NA_Age26) &A$AGE93==26]<-A$A93[is.na(A$NA_Age26)
&A$AGE93==26]
table(A$NA_Age26,useNA = "always")
A$NA_Age26[is.na(A$NA_Age26) &A$AGE94==26]<-A$A94[is.na(A$NA_Age26)
&A$AGE94==26]
table(A$NA_Age26,useNA = "always")
A$NA_Age26[is.na(A$NA_Age26) &A$AGE95==26]<-A$A95[is.na(A$NA_Age26)
&A$AGE95==26]
table(A$NA_Age26,useNA = "always")
A$NA_Age26[is.na(A$NA_Age26) &A$AGE96==26]<-A$A96[is.na(A$NA_Age26)
&A$AGE96==26]
table(A$NA_Age26,useNA = "always")
A$NA_Age26[is.na(A$NA_Age26) &A$AGE97==26]<-A$A97[is.na(A$NA_Age26)
&A$AGE97==26]
table(A$NA_Age26,useNA = "always")
A$NA_Age26[is.na(A$NA_Age26) &A$AGE98==26]<-A$A98[is.na(A$NA_Age26)
&A$AGE98==26]
table(A$NA_Age26,useNA = "always")
A$NA_Age26[is.na(A$NA_Age26) &A$AGE99==26]<-A$A99[is.na(A$NA_Age26)
&A$AGE99==26]
table(A$NA_Age26,useNA = "always")
A$NA_Age26[is.na(A$NA_Age26) & A$AGE00==26]<-A$A00[is.na(A$NA_Age26)
&A$AGE00==26]
table(A$NA_Age26,useNA = "always")
A$NA_Age26[is.na(A$NA_Age26) & A$AGE01==26]<-A$A01[is.na(A$NA_Age26)
&A$AGE01==26]
table(A$NA_Age26,useNA = "always")
A$NA_Age26[is.na(A$NA_Age26) & A$AGE02==26]<-A$A02[is.na(A$NA_Age26)
&A$AGE02==26]
table(A$NA_Age26,useNA = "always")
A$NA_Age26[is.na(A$NA_Age26) & A$AGE03==26]<-A$A03[is.na(A$NA_Age26)
&A$AGE03==26]
table(A$NA_Age26,useNA = "always")
```

© 2023 Silver IA et al. *JAMA Network Open.*

```
A$NA_Age26[is.na(A$NA_Age26) & A$AGE04==26]<-A$A04[is.na(A$NA_Age26)
&A$AGE04==26]
table(A$NA_Age26,useNA = "always")
A$NA_Age26[is.na(A$NA_Age26) & A$AGE05==26]<-A$A05[is.na(A$NA_Age26)
&A$AGE05==26]
table(A$NA_Age26,useNA = "always")
A$NA_Age26[is.na(A$NA_Age26) & A$AGE06==26]<-A$A06[is.na(A$NA_Age26)
&A$AGE06==26]
table(A$NA_Age26,useNA = "always")
A$NA_Age26[is.na(A$NA_Age26) & A$AGE07==26]<-A$A07[is.na(A$NA_Age26)
&A$AGE07==26]
table(A$NA_Age26,useNA = "always")
A$NA_Age26[is.na(A$NA_Age26) & A$AGE08==26]<-A$A08[is.na(A$NA_Age26)
&A$AGE08==26]
table(A$NA_Age26,useNA = "always")
A$NA_Age26[is.na(A$NA_Age26) & A$AGE09==26]<-A$A09[is.na(A$NA_Age26)
&A$AGE09==26]
table(A$NA_Age26,useNA = "always")
A$NA_Age26[is.na(A$NA_Age26) & A$AGE10==26]<-A$A10[is.na(A$NA_Age26)
&A$AGE10==26]
table(A$NA_Age26,useNA = "always")
A$NA_Age26[is.na(A$NA_Age26) & A$AGE11==26]<-A$A11[is.na(A$NA_Age26)
&A$AGE11==26]
table(A$NA_Age26,useNA = "always")
A$NA_Age26[A$Dec_Age26==1]<-NA
table(A$NA_Age26,useNA = "always")
## NA_Age27
table(A$AGE17)
A$NA_Age27<-NA
A$NA_Age27[is.na(A$NA_Age27) & A$AGE92==27]<-A$A92[is.na(A$NA_Age27) &
A$AGE92==27]
table(A$NA_Age27,useNA = "always")
A$NA_Age27[is.na(A$NA_Age27) &A$AGE93==27]<-A$A93[is.na(A$NA_Age27)
&A$AGE93==27]
table(A$NA_Age27,useNA = "always")
A$NA_Age27[is.na(A$NA_Age27) &A$AGE94==27]<-A$A94[is.na(A$NA_Age27)
&A$AGE94==27]
table(A$NA_Age27,useNA = "always")
A$NA_Age27[is.na(A$NA_Age27) &A$AGE95==27]<-A$A95[is.na(A$NA_Age27)
&A$AGE95==27]
table(A$NA_Age27,useNA = "always")
A$NA_Age27[is.na(A$NA_Age27) &A$AGE96==27]<-A$A96[is.na(A$NA_Age27)
&A$AGE96==27]
table(A$NA_Age27,useNA = "always")
A$NA_Age27[is.na(A$NA_Age27) &A$AGE97==27]<-A$A97[is.na(A$NA_Age27)
&A$AGE97==27]
table(A$NA_Age27,useNA = "always")
A$NA_Age27[is.na(A$NA_Age27) &A$AGE98==27]<-A$A98[is.na(A$NA_Age27)
&A$AGE98==27]
table(A$NA_Age27,useNA = "always")
A$NA_Age27[is.na(A$NA_Age27) &A$AGE99==27]<-A$A99[is.na(A$NA_Age27)
&A$AGE99==27]
table(A$NA_Age27,useNA = "always")
A$NA_Age27[is.na(A$NA_Age27) & A$AGE00==27]<-A$A00[is.na(A$NA_Age27)
&A$AGE00==27]
table(A$NA_Age27,useNA = "always")
```

```
A$NA_Age27[is.na(A$NA_Age27) & A$AGE01==27]<-A$A01[is.na(A$NA_Age27)
&A$AGE01==27]
table(A$NA_Age27,useNA = "always")
A$NA_Age27[is.na(A$NA_Age27) & A$AGE02==27]<-A$A02[is.na(A$NA_Age27)
&A$AGE02==27]
table(A$NA_Age27,useNA = "always")
A$NA_Age27[is.na(A$NA_Age27) & A$AGE03==27]<-A$A03[is.na(A$NA_Age27)
&A$AGE03==27]
table(A$NA_Age27,useNA = "always")
A$NA_Age27[is.na(A$NA_Age27) & A$AGE04==27]<-A$A04[is.na(A$NA_Age27)
&A$AGE04==27]
table(A$NA_Age27,useNA = "always")
A$NA_Age27[is.na(A$NA_Age27) & A$AGE05==27]<-A$A05[is.na(A$NA_Age27)
&A$AGE05==27]
table(A$NA_Age27,useNA = "always")
A$NA_Age27[is.na(A$NA_Age27) & A$AGE06==27]<-A$A06[is.na(A$NA_Age27)
&A$AGE06==27]
table(A$NA_Age27,useNA = "always")
A$NA_Age27[is.na(A$NA_Age27) & A$AGE07==27]<-A$A07[is.na(A$NA_Age27)
&A$AGE07==27]
table(A$NA_Age27,useNA = "always")
A$NA_Age27[is.na(A$NA_Age27) & A$AGE08==27]<-A$A08[is.na(A$NA_Age27)
&A$AGE08==27]
table(A$NA_Age27,useNA = "always")
A$NA_Age27[is.na(A$NA_Age27) & A$AGE09==27]<-A$A09[is.na(A$NA_Age27)
&A$AGE09==27]
table(A$NA_Age27,useNA = "always")
A$NA_Age27[is.na(A$NA_Age27) & A$AGE10==27]<-A$A10[is.na(A$NA_Age27)
&A$AGE10==27]
table(A$NA_Age27,useNA = "always")
A$NA_Age27[is.na(A$NA_Age27) & A$AGE11==27]<-A$A11[is.na(A$NA_Age27)
&A$AGE11==27]
table(A$NA_Age27,useNA = "always")
A$NA_Age27[is.na(A$NA_Age27) & A$AGE12==27]<-A$A12[is.na(A$NA_Age27)
&A$AGE12==27]
table(A$NA_Age27,useNA = "always")
A$NA_Age27[A$Dec_Age27==1]<-NA
table(A$NA_Age27,useNA = "always")
## NA_Age28
table(A$AGE17)
A$NA_Age28<-NA
A$NA_Age28[is.na(A$NA_Age28) & A$AGE92==28]<-A$A92[is.na(A$NA_Age28) &
A$AGE92==28]
table(A$NA_Age28,useNA = "always")
A$NA_Age28[is.na(A$NA_Age28) &A$AGE93==28]<-A$A93[is.na(A$NA_Age28)
&A$AGE93==28]
table(A$NA_Age28,useNA = "always")
A$NA_Age28[is.na(A$NA_Age28) &A$AGE94==28]<-A$A94[is.na(A$NA_Age28)
&A$AGE94==28]
table(A$NA_Age28,useNA = "always")
A$NA_Age28[is.na(A$NA_Age28) &A$AGE95==28]<-A$A95[is.na(A$NA_Age28)
&A$AGE95==28]
table(A$NA_Age28,useNA = "always")
A$NA_Age28[is.na(A$NA_Age28) &A$AGE96==28]<-A$A96[is.na(A$NA_Age28)
&A$AGE96==28]
table(A$NA_Age28,useNA = "always")
```

© 2023 Silver IA et al. *JAMA Network Open.*

```
A$NA_Age28[is.na(A$NA_Age28) &A$AGE97==28]<-A$A97[is.na(A$NA_Age28)
&A$AGE97==28]
table(A$NA_Age28,useNA = "always")
A$NA_Age28[is.na(A$NA_Age28) &A$AGE98==28]<-A$A98[is.na(A$NA_Age28)
&A$AGE98==28]
table(A$NA_Age28,useNA = "always")
A$NA_Age28[is.na(A$NA_Age28) &A$AGE99==28]<-A$A99[is.na(A$NA_Age28)
&A$AGE99==28]
table(A$NA_Age28,useNA = "always")
A$NA_Age28[is.na(A$NA_Age28) & A$AGE00==28]<-A$A00[is.na(A$NA_Age28)
&A$AGE00==28]
table(A$NA_Age28,useNA = "always")
A$NA_Age28[is.na(A$NA_Age28) & A$AGE01==28]<-A$A01[is.na(A$NA_Age28)
&A$AGE01==28]
table(A$NA_Age28,useNA = "always")
A$NA_Age28[is.na(A$NA_Age28) & A$AGE02==28]<-A$A02[is.na(A$NA_Age28)
&A$AGE02==28]
table(A$NA_Age28,useNA = "always")
A$NA_Age28[is.na(A$NA_Age28) & A$AGE03==28]<-A$A03[is.na(A$NA_Age28)
&A$AGE03==28]
table(A$NA_Age28,useNA = "always")
A$NA_Age28[is.na(A$NA_Age28) & A$AGE04==28]<-A$A04[is.na(A$NA_Age28)
&A$AGE04==28]
table(A$NA_Age28,useNA = "always")
A$NA_Age28[is.na(A$NA_Age28) & A$AGE05==28]<-A$A05[is.na(A$NA_Age28)
&A$AGE05==28]
table(A$NA_Age28,useNA = "always")
A$NA_Age28[is.na(A$NA_Age28) & A$AGE06==28]<-A$A06[is.na(A$NA_Age28)
&A$AGE06==28]
table(A$NA_Age28,useNA = "always")
A$NA_Age28[is.na(A$NA_Age28) & A$AGE07==28]<-A$A07[is.na(A$NA_Age28)
&A$AGE07==28]
table(A$NA_Age28,useNA = "always")
A$NA_Age28[is.na(A$NA_Age28) & A$AGE08==28]<-A$A08[is.na(A$NA_Age28)
&A$AGE08==28]
table(A$NA_Age28,useNA = "always")
A$NA_Age28[is.na(A$NA_Age28) & A$AGE09==28]<-A$A09[is.na(A$NA_Age28)
&A$AGE09==28]
table(A$NA_Age28,useNA = "always")
A$NA_Age28[is.na(A$NA_Age28) & A$AGE10==28]<-A$A10[is.na(A$NA_Age28)
&A$AGE10==28]
table(A$NA_Age28,useNA = "always")
A$NA_Age28[is.na(A$NA_Age28) & A$AGE11==28]<-A$A11[is.na(A$NA_Age28)
&A$AGE11==28]
table(A$NA_Age28,useNA = "always")
A$NA_Age28[is.na(A$NA_Age28) & A$AGE12==28]<-A$A12[is.na(A$NA_Age28)
&A$AGE12==28]
table(A$NA_Age28,useNA = "always")
A$NA_Age28[is.na(A$NA_Age28) & A$AGE13==28]<-A$A13[is.na(A$NA_Age28)
&A$AGE13==28]
table(A$NA_Age28,useNA = "always")
A$NA_Age28[A$Dec_Age28==1]<-NA
table(A$NA_Age28,useNA = "always")
## NA_Age29
table(A$AGE17)
A$NA_Age29<-NA
```

© 2023 Silver IA et al. *JAMA Network Open.*

```
A$NA_Age29[is.na(A$NA_Age29) & A$AGE92==29]<-A$A92[is.na(A$NA_Age29) &
A$AGE92==29]
table(A$NA_Age29,useNA = "always")
A$NA_Age29[is.na(A$NA_Age29) &A$AGE93==29]<-A$A93[is.na(A$NA_Age29)
&A$AGE93==29]
table(A$NA_Age29,useNA = "always")
A$NA_Age29[is.na(A$NA_Age29) &A$AGE94==29]<-A$A94[is.na(A$NA_Age29)
&A$AGE94==29]
table(A$NA_Age29,useNA = "always")
A$NA_Age29[is.na(A$NA_Age29) &A$AGE95==29]<-A$A95[is.na(A$NA_Age29)
&A$AGE95==29]
table(A$NA_Age29,useNA = "always")
A$NA_Age29[is.na(A$NA_Age29) &A$AGE96==29]<-A$A96[is.na(A$NA_Age29)
&A$AGE96==29]
table(A$NA_Age29,useNA = "always")
A$NA_Age29[is.na(A$NA_Age29) &A$AGE97==29]<-A$A97[is.na(A$NA_Age29)
&A$AGE97==29]
table(A$NA_Age29,useNA = "always")
A$NA_Age29[is.na(A$NA_Age29) &A$AGE98==29]<-A$A98[is.na(A$NA_Age29)
&A$AGE98==29]
table(A$NA_Age29,useNA = "always")
A$NA_Age29[is.na(A$NA_Age29) &A$AGE99==29]<-A$A99[is.na(A$NA_Age29)
&A$AGE99==29]
table(A$NA_Age29,useNA = "always")
A$NA_Age29[is.na(A$NA_Age29) & A$AGE00==29]<-A$A00[is.na(A$NA_Age29)
&A$AGE00==29]
table(A$NA_Age29,useNA = "always")
A$NA_Age29[is.na(A$NA_Age29) & A$AGE01==29]<-A$A01[is.na(A$NA_Age29)
&A$AGE01==29]
table(A$NA_Age29,useNA = "always")
A$NA_Age29[is.na(A$NA_Age29) & A$AGE02==29]<-A$A02[is.na(A$NA_Age29)
&A$AGE02==29]
table(A$NA_Age29,useNA = "always")
A$NA_Age29[is.na(A$NA_Age29) & A$AGE03==29]<-A$A03[is.na(A$NA_Age29)
&A$AGE03==29]
table(A$NA_Age29,useNA = "always")
A$NA_Age29[is.na(A$NA_Age29) & A$AGE04==29]<-A$A04[is.na(A$NA_Age29)
&A$AGE04==29]
table(A$NA_Age29,useNA = "always")
A$NA_Age29[is.na(A$NA_Age29) & A$AGE05==29]<-A$A05[is.na(A$NA_Age29)
&A$AGE05==29]
table(A$NA_Age29,useNA = "always")
A$NA_Age29[is.na(A$NA_Age29) & A$AGE06==29]<-A$A06[is.na(A$NA_Age29)
&A$AGE06==29]
table(A$NA_Age29,useNA = "always")
A$NA_Age29[is.na(A$NA_Age29) & A$AGE07==29]<-A$A07[is.na(A$NA_Age29)
&A$AGE07==29]
table(A$NA_Age29,useNA = "always")
A$NA_Age29[is.na(A$NA_Age29) & A$AGE08==29]<-A$A08[is.na(A$NA_Age29)
&A$AGE08==29]
table(A$NA_Age29,useNA = "always")
A$NA_Age29[is.na(A$NA_Age29) & A$AGE09==29]<-A$A09[is.na(A$NA_Age29)
&A$AGE09==29]
table(A$NA_Age29,useNA = "always")
A$NA_Age29[is.na(A$NA_Age29) & A$AGE10==29]<-A$A10[is.na(A$NA_Age29)
&A$AGE10==29]
```

```
table(A$NA_Age29,useNA = "always")
A$NA_Age29[is.na(A$NA_Age29) & A$AGE11==29]<-A$A11[is.na(A$NA_Age29)
&A$AGE11==29]
table(A$NA_Age29,useNA = "always")
A$NA_Age29[is.na(A$NA_Age29) & A$AGE12==29]<-A$A12[is.na(A$NA_Age29)
&A$AGE12==29]
table(A$NA_Age29,useNA = "always")
A$NA_Age29[is.na(A$NA_Age29) & A$AGE13==29]<-A$A13[is.na(A$NA_Age29)
&A$AGE13==29]
table(A$NA_Age29,useNA = "always")
A$NA_Age29[is.na(A$NA_Age29) & A$AGE14==29]<-A$A14[is.na(A$NA_Age29)
&A$AGE14==29]
table(A$NA_Age29,useNA = "always")
A$NA_Age29[A$Dec_Age29==1]<-NA
table(A$NA_Age29,useNA = "always")
## NA_Age30
table(A$AGE17)
A$NA_Age30<-NA
A$NA_Age30[is.na(A$NA_Age30) & A$AGE92==30]<-A$A92[is.na(A$NA_Age30) &
A$AGE92==30]
table(A$NA_Age30,useNA = "always")
A$NA_Age30[is.na(A$NA_Age30) &A$AGE93==30]<-A$A93[is.na(A$NA_Age30)
&A$AGE93==30]
table(A$NA_Age30,useNA = "always")
A$NA_Age30[is.na(A$NA_Age30) &A$AGE94==30]<-A$A94[is.na(A$NA_Age30)
&A$AGE94==30]
table(A$NA_Age30,useNA = "always")
A$NA_Age30[is.na(A$NA_Age30) &A$AGE95==30]<-A$A95[is.na(A$NA_Age30)
&A$AGE95==30]
table(A$NA_Age30,useNA = "always")
A$NA_Age30[is.na(A$NA_Age30) &A$AGE96==30]<-A$A96[is.na(A$NA_Age30)
&A$AGE96==30]
table(A$NA_Age30,useNA = "always")
A$NA_Age30[is.na(A$NA_Age30) &A$AGE97==30]<-A$A97[is.na(A$NA_Age30)
&A$AGE97==30]
table(A$NA_Age30,useNA = "always")
A$NA_Age30[is.na(A$NA_Age30) &A$AGE98==30]<-A$A98[is.na(A$NA_Age30)
&A$AGE98==30]
table(A$NA_Age30,useNA = "always")
A$NA_Age30[is.na(A$NA_Age30) &A$AGE99==30]<-A$A99[is.na(A$NA_Age30)
&A$AGE99==30]
table(A$NA_Age30,useNA = "always")
A$NA_Age30[is.na(A$NA_Age30) & A$AGE00==30]<-A$A00[is.na(A$NA_Age30)
&A$AGE00==30]
table(A$NA_Age30,useNA = "always")
A$NA_Age30[is.na(A$NA_Age30) & A$AGE01==30]<-A$A01[is.na(A$NA_Age30)
&A$AGE01==30]
table(A$NA_Age30,useNA = "always")
A$NA_Age30[is.na(A$NA_Age30) & A$AGE02==30]<-A$A02[is.na(A$NA_Age30)
&A$AGE02==30]
table(A$NA_Age30,useNA = "always")
A$NA_Age30[is.na(A$NA_Age30) & A$AGE03==30]<-A$A03[is.na(A$NA_Age30)
&A$AGE03==30]
table(A$NA_Age30,useNA = "always")
A$NA_Age30[is.na(A$NA_Age30) & A$AGE04==30]<-A$A04[is.na(A$NA_Age30)
&A$AGE04==30]
```

© 2023 Silver IA et al. *JAMA Network Open.*

```
table(A$NA_Age30,useNA = "always")
A$NA_Age30[is.na(A$NA_Age30) & A$AGE05==30]<-A$A05[is.na(A$NA_Age30)
&A$AGE05==30]
table(A$NA_Age30,useNA = "always")
A$NA_Age30[is.na(A$NA_Age30) & A$AGE06==30]<-A$A06[is.na(A$NA_Age30)
&A$AGE06==30]
table(A$NA_Age30,useNA = "always")
A$NA_Age30[is.na(A$NA_Age30) & A$AGE07==30]<-A$A07[is.na(A$NA_Age30)
&A$AGE07==30]
table(A$NA_Age30,useNA = "always")
A$NA_Age30[is.na(A$NA_Age30) & A$AGE08==30]<-A$A08[is.na(A$NA_Age30)
&A$AGE08==30]
table(A$NA_Age30,useNA = "always")
A$NA_Age30[is.na(A$NA_Age30) & A$AGE09==30]<-A$A09[is.na(A$NA_Age30)
&A$AGE09==30]
table(A$NA_Age30,useNA = "always")
A$NA_Age30[is.na(A$NA_Age30) & A$AGE10==30]<-A$A10[is.na(A$NA_Age30)
&A$AGE10==30]
table(A$NA_Age30,useNA = "always")
A$NA_Age30[is.na(A$NA_Age30) & A$AGE11==30]<-A$A11[is.na(A$NA_Age30)
&A$AGE11==30]
table(A$NA_Age30,useNA = "always")
A$NA_Age30[is.na(A$NA_Age30) & A$AGE12==30]<-A$A12[is.na(A$NA_Age30)
&A$AGE12==30]
table(A$NA_Age30,useNA = "always")
A$NA_Age30[is.na(A$NA_Age30) & A$AGE13==30]<-A$A13[is.na(A$NA_Age30)
&A$AGE13==30]
table(A$NA_Age30,useNA = "always")
A$NA_Age30[is.na(A$NA_Age30) & A$AGE14==30]<-A$A14[is.na(A$NA_Age30)
&A$AGE14==30]
table(A$NA_Age30,useNA = "always")
A$NA_Age30[is.na(A$NA_Age30) & A$AGE15==30]<-A$A15[is.na(A$NA_Age30)
&A$AGE15==30]
table(A$NA_Age30,useNA = "always")
A$NA_Age30[A$Dec_Age30==1]<-NA
table(A$NA_Age30,useNA = "always")
## NA_Age31
table(A$AGE17)
A$NA_Age31<-NA
A$NA_Age31[is.na(A$NA_Age31) & A$AGE92==31]<-A$A92[is.na(A$NA_Age31) &
A$AGE92==31]
table(A$NA_Age31,useNA = "always")
A$NA_Age31[is.na(A$NA_Age31) &A$AGE93==31]<-A$A93[is.na(A$NA_Age31)
&A$AGE93==31]
table(A$NA_Age31,useNA = "always")
A$NA_Age31[is.na(A$NA_Age31) &A$AGE94==31]<-A$A94[is.na(A$NA_Age31)
&A$AGE94==31]
table(A$NA_Age31,useNA = "always")
A$NA_Age31[is.na(A$NA_Age31) &A$AGE95==31]<-A$A95[is.na(A$NA_Age31)
&A$AGE95==31]
table(A$NA_Age31,useNA = "always")
A$NA_Age31[is.na(A$NA_Age31) &A$AGE96==31]<-A$A96[is.na(A$NA_Age31)
&A$AGE96==31]
table(A$NA_Age31,useNA = "always")
A$NA_Age31[is.na(A$NA_Age31) &A$AGE97==31]<-A$A97[is.na(A$NA_Age31)
&A$AGE97==31]
```

```
table(A$NA_Age31,useNA = "always")
A$NA_Age31[is.na(A$NA_Age31) &A$AGE98==31]<-A$A98[is.na(A$NA_Age31)
&A$AGE98==31]
table(A$NA_Age31,useNA = "always")
A$NA_Age31[is.na(A$NA_Age31) &A$AGE99==31]<-A$A99[is.na(A$NA_Age31)
&A$AGE99==31]
table(A$NA_Age31,useNA = "always")
A$NA_Age31[is.na(A$NA_Age31) & A$AGE00==31]<-A$A00[is.na(A$NA_Age31)
&A$AGE00==31]
table(A$NA_Age31,useNA = "always")
A$NA_Age31[is.na(A$NA_Age31) & A$AGE01==31]<-A$A01[is.na(A$NA_Age31)
&A$AGE01==31]
table(A$NA_Age31,useNA = "always")
A$NA_Age31[is.na(A$NA_Age31) & A$AGE02==31]<-A$A02[is.na(A$NA_Age31)
&A$AGE02==31]
table(A$NA_Age31,useNA = "always")
A$NA_Age31[is.na(A$NA_Age31) & A$AGE03==31]<-A$A03[is.na(A$NA_Age31)
&A$AGE03==31]
table(A$NA_Age31,useNA = "always")
A$NA_Age31[is.na(A$NA_Age31) & A$AGE04==31]<-A$A04[is.na(A$NA_Age31)
&A$AGE04==31]
table(A$NA_Age31,useNA = "always")
A$NA_Age31[is.na(A$NA_Age31) & A$AGE05==31]<-A$A05[is.na(A$NA_Age31)
&A$AGE05==31]
table(A$NA_Age31,useNA = "always")
A$NA_Age31[is.na(A$NA_Age31) & A$AGE06==31]<-A$A06[is.na(A$NA_Age31)
&A$AGE06==31]
table(A$NA_Age31,useNA = "always")
A$NA_Age31[is.na(A$NA_Age31) & A$AGE07==31]<-A$A07[is.na(A$NA_Age31)
&A$AGE07==31]
table(A$NA_Age31,useNA = "always")
A$NA_Age31[is.na(A$NA_Age31) & A$AGE08==31]<-A$A08[is.na(A$NA_Age31)
&A$AGE08==31]
table(A$NA_Age31,useNA = "always")
A$NA_Age31[is.na(A$NA_Age31) & A$AGE09==31]<-A$A09[is.na(A$NA_Age31)
&A$AGE09==31]
table(A$NA_Age31,useNA = "always")
A$NA_Age31[is.na(A$NA_Age31) & A$AGE10==31]<-A$A10[is.na(A$NA_Age31)
&A$AGE10==31]
table(A$NA_Age31,useNA = "always")
A$NA_Age31[is.na(A$NA_Age31) & A$AGE11==31]<-A$A11[is.na(A$NA_Age31)
&A$AGE11==31]
table(A$NA_Age31,useNA = "always")
A$NA_Age31[is.na(A$NA_Age31) & A$AGE12==31]<-A$A12[is.na(A$NA_Age31)
&A$AGE12==31]
table(A$NA_Age31,useNA = "always")
A$NA_Age31[is.na(A$NA_Age31) & A$AGE13==31]<-A$A13[is.na(A$NA_Age31)
&A$AGE13==31]
table(A$NA_Age31,useNA = "always")
A$NA_Age31[is.na(A$NA_Age31) & A$AGE14==31]<-A$A14[is.na(A$NA_Age31)
&A$AGE14==31]
table(A$NA_Age31,useNA = "always")
A$NA_Age31[is.na(A$NA_Age31) & A$AGE15==31]<-A$A15[is.na(A$NA_Age31)
&A$AGE15==31]
table(A$NA_Age31,useNA = "always")
```

© 2023 Silver IA et al. *JAMA Network Open.*

```
A$NA_Age31[is.na(A$NA_Age31) & A$AGE16==31]<-A$A16[is.na(A$NA_Age31)
&A$AGE16==31]
table(A$NA_Age31,useNA = "always")
A$NA_Age31[A$Dec_Age31==1]<-NA
table(A$NA_Age31,useNA = "always")
## NA_Age32
table(A$AGE17)
A$NA_Age32<-NA
A$NA_Age32[is.na(A$NA_Age32) & A$AGE92==32]<-A$A92[is.na(A$NA_Age32) &
A$AGE92==32]
table(A$NA_Age32,useNA = "always")
A$NA_Age32[is.na(A$NA_Age32) &A$AGE93==32]<-A$A93[is.na(A$NA_Age32)
&A$AGE93==32]
table(A$NA_Age32,useNA = "always")
A$NA_Age32[is.na(A$NA_Age32) &A$AGE94==32]<-A$A94[is.na(A$NA_Age32)
&A$AGE94==32]
table(A$NA_Age32,useNA = "always")
A$NA_Age32[is.na(A$NA_Age32) &A$AGE95==32]<-A$A95[is.na(A$NA_Age32)
&A$AGE95==32]
table(A$NA_Age32,useNA = "always")
A$NA_Age32[is.na(A$NA_Age32) &A$AGE96==32]<-A$A96[is.na(A$NA_Age32)
&A$AGE96==32]
table(A$NA_Age32,useNA = "always")
A$NA_Age32[is.na(A$NA_Age32) &A$AGE97==32]<-A$A97[is.na(A$NA_Age32)
&A$AGE97==32]
table(A$NA_Age32,useNA = "always")
A$NA_Age32[is.na(A$NA_Age32) &A$AGE98==32]<-A$A98[is.na(A$NA_Age32)
&A$AGE98==32]
table(A$NA_Age32,useNA = "always")
A$NA_Age32[is.na(A$NA_Age32) &A$AGE99==32]<-A$A99[is.na(A$NA_Age32)
&A$AGE99==32]
table(A$NA_Age32,useNA = "always")
A$NA_Age32[is.na(A$NA_Age32) & A$AGE00==32]<-A$A00[is.na(A$NA_Age32)
&A$AGE00==32]
table(A$NA_Age32,useNA = "always")
A$NA_Age32[is.na(A$NA_Age32) & A$AGE01==32]<-A$A01[is.na(A$NA_Age32)
&A$AGE01==32]
table(A$NA_Age32,useNA = "always")
A$NA_Age32[is.na(A$NA_Age32) & A$AGE02==32]<-A$A02[is.na(A$NA_Age32)
&A$AGE02==32]
table(A$NA_Age32,useNA = "always")
A$NA_Age32[is.na(A$NA_Age32) & A$AGE03==32]<-A$A03[is.na(A$NA_Age32)
&A$AGE03==32]
table(A$NA_Age32,useNA = "always")
A$NA_Age32[is.na(A$NA_Age32) & A$AGE04==32]<-A$A04[is.na(A$NA_Age32)
&A$AGE04==32]
table(A$NA_Age32,useNA = "always")
A$NA_Age32[is.na(A$NA_Age32) & A$AGE05==32]<-A$A05[is.na(A$NA_Age32)
&A$AGE05==32]
table(A$NA_Age32,useNA = "always")
A$NA_Age32[is.na(A$NA_Age32) & A$AGE06==32]<-A$A06[is.na(A$NA_Age32)
&A$AGE06==32]
table(A$NA_Age32,useNA = "always")
A$NA_Age32[is.na(A$NA_Age32) & A$AGE07==32]<-A$A07[is.na(A$NA_Age32)
&A$AGE07==32]
table(A$NA_Age32,useNA = "always")
```

© 2023 Silver IA et al. *JAMA Network Open.*

```
A$NA_Age32[is.na(A$NA_Age32) & A$AGE08==32]<-A$A08[is.na(A$NA_Age32)
&A$AGE08==32]
table(A$NA_Age32,useNA = "always")
A$NA_Age32[is.na(A$NA_Age32) & A$AGE09==32]<-A$A09[is.na(A$NA_Age32)
&A$AGE09==32]
table(A$NA_Age32,useNA = "always")
A$NA_Age32[is.na(A$NA_Age32) & A$AGE10==32]<-A$A10[is.na(A$NA_Age32)
&A$AGE10==32]
table(A$NA_Age32,useNA = "always")
A$NA_Age32[is.na(A$NA_Age32) & A$AGE11==32]<-A$A11[is.na(A$NA_Age32)
&A$AGE11==32]
table(A$NA_Age32,useNA = "always")
A$NA_Age32[is.na(A$NA_Age32) & A$AGE12==32]<-A$A12[is.na(A$NA_Age32)
&A$AGE12==32]
table(A$NA_Age32,useNA = "always")
A$NA_Age32[is.na(A$NA_Age32) & A$AGE13==32]<-A$A13[is.na(A$NA_Age32)
&A$AGE13==32]
table(A$NA_Age32,useNA = "always")
A$NA_Age32[is.na(A$NA_Age32) & A$AGE14==32]<-A$A14[is.na(A$NA_Age32)
&A$AGE14==32]
table(A$NA_Age32,useNA = "always")
A$NA_Age32[is.na(A$NA_Age32) & A$AGE15==32]<-A$A15[is.na(A$NA_Age32)
&A$AGE15==32]
table(A$NA_Age32,useNA = "always")
A$NA_Age32[is.na(A$NA_Age32) & A$AGE16==32]<-A$A16[is.na(A$NA_Age32)
&A$AGE16==32]
table(A$NA_Age32,useNA = "always")
A$NA_Age32[is.na(A$NA_Age32) & A$AGE17==32]<-A$A17[is.na(A$NA_Age32)
&A$AGE17==32]
table(A$NA_Age32,useNA = "always")
A$NA_Age32[A$Dec_Age32==1]<-NA
table(A$NA_Age32,useNA = "always")
## NA_Age33
table(A$AGE17)
A$NA_Age33<-NA
A$NA_Age33[is.na(A$NA_Age33) & A$AGE92==33]<-A$A92[is.na(A$NA_Age33) &
A$AGE92==33]
table(A$NA_Age33,useNA = "always")
A$NA_Age33[is.na(A$NA_Age33) &A$AGE93==33]<-A$A93[is.na(A$NA_Age33)
&A$AGE93==33]
table(A$NA_Age33,useNA = "always")
A$NA_Age33[is.na(A$NA_Age33) &A$AGE94==33]<-A$A94[is.na(A$NA_Age33)
&A$AGE94==33]
table(A$NA_Age33,useNA = "always")
A$NA_Age33[is.na(A$NA_Age33) &A$AGE95==33]<-A$A95[is.na(A$NA_Age33)
&A$AGE95==33]
table(A$NA_Age33,useNA = "always")
A$NA_Age33[is.na(A$NA_Age33) &A$AGE96==33]<-A$A96[is.na(A$NA_Age33)
&A$AGE96==33]
table(A$NA_Age33,useNA = "always")
A$NA_Age33[is.na(A$NA_Age33) &A$AGE97==33]<-A$A97[is.na(A$NA_Age33)
&A$AGE97==33]
table(A$NA_Age33,useNA = "always")
A$NA_Age33[is.na(A$NA_Age33) &A$AGE98==33]<-A$A98[is.na(A$NA_Age33)
&A$AGE98==33]
table(A$NA_Age33,useNA = "always")
```

© 2023 Silver IA et al. *JAMA Network Open.*

```
A$NA_Age33[is.na(A$NA_Age33) &A$AGE99==33]<-A$A99[is.na(A$NA_Age33)
&A$AGE99==33]
table(A$NA_Age33,useNA = "always")
A$NA_Age33[is.na(A$NA_Age33) & A$AGE00==33]<-A$A00[is.na(A$NA_Age33)
&A$AGE00==33]
table(A$NA_Age33,useNA = "always")
A$NA_Age33[is.na(A$NA_Age33) & A$AGE01==33]<-A$A01[is.na(A$NA_Age33)
&A$AGE01==33]
table(A$NA_Age33,useNA = "always")
A$NA_Age33[is.na(A$NA_Age33) & A$AGE02==33]<-A$A02[is.na(A$NA_Age33)
&A$AGE02==33]
table(A$NA_Age33,useNA = "always")
A$NA_Age33[is.na(A$NA_Age33) & A$AGE03==33]<-A$A03[is.na(A$NA_Age33)
&A$AGE03==33]
table(A$NA_Age33,useNA = "always")
A$NA_Age33[is.na(A$NA_Age33) & A$AGE04==33]<-A$A04[is.na(A$NA_Age33)
&A$AGE04==33]
table(A$NA_Age33,useNA = "always")
A$NA_Age33[is.na(A$NA_Age33) & A$AGE05==33]<-A$A05[is.na(A$NA_Age33)
&A$AGE05==33]
table(A$NA_Age33,useNA = "always")
A$NA_Age33[is.na(A$NA_Age33) & A$AGE06==33]<-A$A06[is.na(A$NA_Age33)
&A$AGE06==33]
table(A$NA_Age33,useNA = "always")
A$NA_Age33[is.na(A$NA_Age33) & A$AGE07==33]<-A$A07[is.na(A$NA_Age33)
&A$AGE07==33]
table(A$NA_Age33,useNA = "always")
A$NA_Age33[is.na(A$NA_Age33) & A$AGE08==33]<-A$A08[is.na(A$NA_Age33)
&A$AGE08==33]
table(A$NA_Age33,useNA = "always")
A$NA_Age33[is.na(A$NA_Age33) & A$AGE09==33]<-A$A09[is.na(A$NA_Age33)
&A$AGE09==33]
table(A$NA_Age33,useNA = "always")
A$NA_Age33[is.na(A$NA_Age33) & A$AGE10==33]<-A$A10[is.na(A$NA_Age33)
&A$AGE10==33]
table(A$NA_Age33,useNA = "always")
A$NA_Age33[is.na(A$NA_Age33) & A$AGE11==33]<-A$A11[is.na(A$NA_Age33)
&A$AGE11==33]
table(A$NA_Age33,useNA = "always")
A$NA_Age33[is.na(A$NA_Age33) & A$AGE12==33]<-A$A12[is.na(A$NA_Age33)
&A$AGE12==33]
table(A$NA_Age33,useNA = "always")
A$NA_Age33[is.na(A$NA_Age33) & A$AGE13==33]<-A$A13[is.na(A$NA_Age33)
&A$AGE13==33]
table(A$NA_Age33,useNA = "always")
A$NA_Age33[is.na(A$NA_Age33) & A$AGE14==33]<-A$A14[is.na(A$NA_Age33)
&A$AGE14==33]
table(A$NA_Age33,useNA = "always")
A$NA_Age33[is.na(A$NA_Age33) & A$AGE15==33]<-A$A15[is.na(A$NA_Age33)
&A$AGE15==33]
table(A$NA_Age33,useNA = "always")
A$NA_Age33[is.na(A$NA_Age33) & A$AGE16==33]<-A$A16[is.na(A$NA_Age33)
&A$AGE16==33]
table(A$NA_Age33,useNA = "always")
A$NA_Age33[is.na(A$NA_Age33) & A$AGE17==33]<-A$A17[is.na(A$NA_Age33)
&A$AGE17==33]
```

© 2023 Silver IA et al. *JAMA Network Open.*

```
table(A$NA_Age33,useNA = "always")
A$NA_Age33[A$Dec_Age33==1]<-NA
table(A$NA_Age33,useNA = "always")
## NA_Age34
table(A$AGE17)
A$NA_Age34<-NA
A$NA_Age34[is.na(A$NA_Age34) & A$AGE92==34]<-A$A92[is.na(A$NA_Age34) &
A$AGE92==34]
table(A$NA_Age34,useNA = "always")
A$NA_Age34[is.na(A$NA_Age34) &A$AGE93==34]<-A$A93[is.na(A$NA_Age34)
&A$AGE93==34]
table(A$NA_Age34,useNA = "always")
A$NA_Age34[is.na(A$NA_Age34) &A$AGE94==34]<-A$A94[is.na(A$NA_Age34)
&A$AGE94==34]
table(A$NA_Age34,useNA = "always")
A$NA_Age34[is.na(A$NA_Age34) &A$AGE95==34]<-A$A95[is.na(A$NA_Age34)
&A$AGE95==34]
table(A$NA_Age34,useNA = "always")
A$NA_Age34[is.na(A$NA_Age34) &A$AGE96==34]<-A$A96[is.na(A$NA_Age34)
&A$AGE96==34]
table(A$NA_Age34,useNA = "always")
A$NA_Age34[is.na(A$NA_Age34) &A$AGE97==34]<-A$A97[is.na(A$NA_Age34)
&A$AGE97==34]
table(A$NA_Age34,useNA = "always")
A$NA_Age34[is.na(A$NA_Age34) &A$AGE98==34]<-A$A98[is.na(A$NA_Age34)
&A$AGE98==34]
table(A$NA_Age34,useNA = "always")
A$NA_Age34[is.na(A$NA_Age34) &A$AGE99==34]<-A$A99[is.na(A$NA_Age34)
&A$AGE99==34]
table(A$NA_Age34,useNA = "always")
A$NA_Age34[is.na(A$NA_Age34) & A$AGE00==34]<-A$A00[is.na(A$NA_Age34)
&A$AGE00==34]
table(A$NA_Age34,useNA = "always")
A$NA_Age34[is.na(A$NA_Age34) & A$AGE01==34]<-A$A01[is.na(A$NA_Age34)
&A$AGE01==34]
table(A$NA_Age34,useNA = "always")
A$NA_Age34[is.na(A$NA_Age34) & A$AGE02==34]<-A$A02[is.na(A$NA_Age34)
&A$AGE02==34]
table(A$NA_Age34,useNA = "always")
A$NA_Age34[is.na(A$NA_Age34) & A$AGE03==34]<-A$A03[is.na(A$NA_Age34)
&A$AGE03==34]
table(A$NA_Age34,useNA = "always")
A$NA_Age34[is.na(A$NA_Age34) & A$AGE04==34]<-A$A04[is.na(A$NA_Age34)
&A$AGE04==34]
table(A$NA_Age34,useNA = "always")
A$NA_Age34[is.na(A$NA_Age34) & A$AGE05==34]<-A$A05[is.na(A$NA_Age34)
&A$AGE05==34]
table(A$NA_Age34,useNA = "always")
A$NA_Age34[is.na(A$NA_Age34) & A$AGE06==34]<-A$A06[is.na(A$NA_Age34)
&A$AGE06==34]
table(A$NA_Age34,useNA = "always")
A$NA_Age34[is.na(A$NA_Age34) & A$AGE07==34]<-A$A07[is.na(A$NA_Age34)
&A$AGE07==34]
table(A$NA_Age34,useNA = "always")
A$NA_Age34[is.na(A$NA_Age34) & A$AGE08==34]<-A$A08[is.na(A$NA_Age34)
&A$AGE08==34]
```

```
table(A$NA_Age34,useNA = "always")
A$NA_Age34[is.na(A$NA_Age34) & A$AGE09==34]<-A$A09[is.na(A$NA_Age34)
&A$AGE09==34]
table(A$NA_Age34,useNA = "always")
A$NA_Age34[is.na(A$NA_Age34) & A$AGE10==34]<-A$A10[is.na(A$NA_Age34)
&A$AGE10==34]
table(A$NA_Age34,useNA = "always")
A$NA_Age34[is.na(A$NA_Age34) & A$AGE11==34]<-A$A11[is.na(A$NA_Age34)
&A$AGE11==34]
table(A$NA_Age34,useNA = "always")
A$NA_Age34[is.na(A$NA_Age34) & A$AGE12==34]<-A$A12[is.na(A$NA_Age34)
&A$AGE12==34]
table(A$NA_Age34,useNA = "always")
A$NA_Age34[is.na(A$NA_Age34) & A$AGE13==34]<-A$A13[is.na(A$NA_Age34)
&A$AGE13==34]
table(A$NA_Age34,useNA = "always")
A$NA_Age34[is.na(A$NA_Age34) & A$AGE14==34]<-A$A14[is.na(A$NA_Age34)
&A$AGE14==34]
table(A$NA_Age34,useNA = "always")
A$NA_Age34[is.na(A$NA_Age34) & A$AGE15==34]<-A$A15[is.na(A$NA_Age34)
&A$AGE15==34]
table(A$NA_Age34,useNA = "always")
A$NA_Age34[is.na(A$NA_Age34) & A$AGE16==34]<-A$A16[is.na(A$NA_Age34)
&A$AGE16==34]
table(A$NA_Age34,useNA = "always")
A$NA_Age34[is.na(A$NA_Age34) & A$AGE17==34]<-A$A17[is.na(A$NA_Age34)
&A$AGE17==34]
table(A$NA_Age34,useNA = "always")
A$NA_Age34[A$Dec_Age34==1]<-NA
table(A$NA_Age34,useNA = "always")
## NA_Age35
table(A$AGE17)
A$NA_Age35<-NA
A$NA_Age35[is.na(A$NA_Age35) & A$AGE92==35]<-A$A92[is.na(A$NA_Age35) &
A$AGE92==35]
table(A$NA_Age35,useNA = "always")
A$NA_Age35[is.na(A$NA_Age35) &A$AGE93==35]<-A$A93[is.na(A$NA_Age35)
&A$AGE93==35]
table(A$NA_Age35,useNA = "always")
A$NA_Age35[is.na(A$NA_Age35) &A$AGE94==35]<-A$A94[is.na(A$NA_Age35)
&A$AGE94==35]
table(A$NA_Age35,useNA = "always")
A$NA_Age35[is.na(A$NA_Age35) &A$AGE95==35]<-A$A95[is.na(A$NA_Age35)
&A$AGE95==35]
table(A$NA_Age35,useNA = "always")
A$NA_Age35[is.na(A$NA_Age35) &A$AGE96==35]<-A$A96[is.na(A$NA_Age35)
&A$AGE96==35]
table(A$NA_Age35,useNA = "always")
A$NA_Age35[is.na(A$NA_Age35) &A$AGE97==35]<-A$A97[is.na(A$NA_Age35)
&A$AGE97==35]
table(A$NA_Age35,useNA = "always")
A$NA_Age35[is.na(A$NA_Age35) &A$AGE98==35]<-A$A98[is.na(A$NA_Age35)
&A$AGE98==35]
table(A$NA_Age35,useNA = "always")
A$NA_Age35[is.na(A$NA_Age35) &A$AGE99==35]<-A$A99[is.na(A$NA_Age35)
&A$AGE99==35]
```

© 2023 Silver IA et al. *JAMA Network Open.*

```
table(A$NA_Age35,useNA = "always")
A$NA_Age35[is.na(A$NA_Age35) & A$AGE00==35]<-A$A00[is.na(A$NA_Age35)
&A$AGE00==35]
table(A$NA_Age35,useNA = "always")
A$NA_Age35[is.na(A$NA_Age35) & A$AGE01==35]<-A$A01[is.na(A$NA_Age35)
&A$AGE01==35]
table(A$NA_Age35,useNA = "always")
A$NA_Age35[is.na(A$NA_Age35) & A$AGE02==35]<-A$A02[is.na(A$NA_Age35)
&A$AGE02==35]
table(A$NA_Age35,useNA = "always")
A$NA_Age35[is.na(A$NA_Age35) & A$AGE03==35]<-A$A03[is.na(A$NA_Age35)
&A$AGE03==35]
table(A$NA_Age35,useNA = "always")
A$NA_Age35[is.na(A$NA_Age35) & A$AGE04==35]<-A$A04[is.na(A$NA_Age35)
&A$AGE04==35]
table(A$NA_Age35,useNA = "always")
A$NA_Age35[is.na(A$NA_Age35) & A$AGE05==35]<-A$A05[is.na(A$NA_Age35)
&A$AGE05==35]
table(A$NA_Age35,useNA = "always")
A$NA_Age35[is.na(A$NA_Age35) & A$AGE06==35]<-A$A06[is.na(A$NA_Age35)
&A$AGE06==35]
table(A$NA_Age35,useNA = "always")
A$NA_Age35[is.na(A$NA_Age35) & A$AGE07==35]<-A$A07[is.na(A$NA_Age35)
&A$AGE07==35]
table(A$NA_Age35,useNA = "always")
A$NA_Age35[is.na(A$NA_Age35) & A$AGE08==35]<-A$A08[is.na(A$NA_Age35)
&A$AGE08==35]
table(A$NA_Age35,useNA = "always")
A$NA_Age35[is.na(A$NA_Age35) & A$AGE09==35]<-A$A09[is.na(A$NA_Age35)
&A$AGE09==35]
table(A$NA_Age35,useNA = "always")
A$NA_Age35[is.na(A$NA_Age35) & A$AGE10==35]<-A$A10[is.na(A$NA_Age35)
&A$AGE10==35]
table(A$NA_Age35,useNA = "always")
A$NA_Age35[is.na(A$NA_Age35) & A$AGE11==35]<-A$A11[is.na(A$NA_Age35)
&A$AGE11==35]
table(A$NA_Age35,useNA = "always")
A$NA_Age35[is.na(A$NA_Age35) & A$AGE12==35]<-A$A12[is.na(A$NA_Age35)
&A$AGE12==35]
table(A$NA_Age35,useNA = "always")
A$NA_Age35[is.na(A$NA_Age35) & A$AGE13==35]<-A$A13[is.na(A$NA_Age35)
&A$AGE13==35]
table(A$NA_Age35,useNA = "always")
A$NA_Age35[is.na(A$NA_Age35) & A$AGE14==35]<-A$A14[is.na(A$NA_Age35)
&A$AGE14==35]
table(A$NA_Age35,useNA = "always")
A$NA_Age35[is.na(A$NA_Age35) & A$AGE15==35]<-A$A15[is.na(A$NA_Age35)
&A$AGE15==35]
table(A$NA_Age35,useNA = "always")
A$NA_Age35[is.na(A$NA_Age35) & A$AGE16==35]<-A$A16[is.na(A$NA_Age35)
&A$AGE16==35]
table(A$NA_Age35,useNA = "always")
A$NA_Age35[is.na(A$NA_Age35) & A$AGE17==35]<-A$A17[is.na(A$NA_Age35)
&A$AGE17==35]
table(A$NA_Age35,useNA = "always")
A$NA_Age35[A$Dec_Age35==1]<-NA
```

© 2023 Silver IA et al. *JAMA Network Open.*

```
table(A$NA_Age35,useNA = "always")
## NA_Age36
table(A$AGE17)
A$NA_Age36<-NA
A$NA_Age36[is.na(A$NA_Age36) & A$AGE92==36]<-A$A92[is.na(A$NA_Age36) &
A$AGE92==36]
table(A$NA_Age36,useNA = "always")
A$NA_Age36[is.na(A$NA_Age36) &A$AGE93==36]<-A$A93[is.na(A$NA_Age36)
&A$AGE93==36]
table(A$NA_Age36,useNA = "always")
A$NA_Age36[is.na(A$NA_Age36) &A$AGE94==36]<-A$A94[is.na(A$NA_Age36)
&A$AGE94==36]
table(A$NA_Age36,useNA = "always")
A$NA_Age36[is.na(A$NA_Age36) &A$AGE95==36]<-A$A95[is.na(A$NA_Age36)
&A$AGE95==36]
table(A$NA_Age36,useNA = "always")
A$NA_Age36[is.na(A$NA_Age36) &A$AGE96==36]<-A$A96[is.na(A$NA_Age36)
&A$AGE96==36]
table(A$NA_Age36,useNA = "always")
A$NA_Age36[is.na(A$NA_Age36) &A$AGE97==36]<-A$A97[is.na(A$NA_Age36)
&A$AGE97==36]
table(A$NA_Age36,useNA = "always")
A$NA_Age36[is.na(A$NA_Age36) &A$AGE98==36]<-A$A98[is.na(A$NA_Age36)
&A$AGE98==36]
table(A$NA_Age36,useNA = "always")
A$NA_Age36[is.na(A$NA_Age36) &A$AGE99==36]<-A$A99[is.na(A$NA_Age36)
&A$AGE99==36]
table(A$NA_Age36,useNA = "always")
A$NA_Age36[is.na(A$NA_Age36) & A$AGE00==36]<-A$A00[is.na(A$NA_Age36)
&A$AGE00==36]
table(A$NA_Age36,useNA = "always")
A$NA_Age36[is.na(A$NA_Age36) & A$AGE01==36]<-A$A01[is.na(A$NA_Age36)
&A$AGE01==36]
table(A$NA_Age36,useNA = "always")
A$NA_Age36[is.na(A$NA_Age36) & A$AGE02==36]<-A$A02[is.na(A$NA_Age36)
&A$AGE02==36]
table(A$NA_Age36,useNA = "always")
A$NA_Age36[is.na(A$NA_Age36) & A$AGE03==36]<-A$A03[is.na(A$NA_Age36)
&A$AGE03==36]
table(A$NA_Age36,useNA = "always")
A$NA_Age36[is.na(A$NA_Age36) & A$AGE04==36]<-A$A04[is.na(A$NA_Age36)
&A$AGE04==36]
table(A$NA_Age36,useNA = "always")
A$NA_Age36[is.na(A$NA_Age36) & A$AGE05==36]<-A$A05[is.na(A$NA_Age36)
&A$AGE05==36]
table(A$NA_Age36,useNA = "always")
A$NA_Age36[is.na(A$NA_Age36) & A$AGE06==36]<-A$A06[is.na(A$NA_Age36)
&A$AGE06==36]
table(A$NA_Age36,useNA = "always")
A$NA_Age36[is.na(A$NA_Age36) & A$AGE07==36]<-A$A07[is.na(A$NA_Age36)
&A$AGE07==36]
table(A$NA_Age36,useNA = "always")
A$NA_Age36[is.na(A$NA_Age36) & A$AGE08==36]<-A$A08[is.na(A$NA_Age36)
&A$AGE08==36]
table(A$NA_Age36,useNA = "always")
```

© 2023 Silver IA et al. *JAMA Network Open.*

```
A$NA_Age36[is.na(A$NA_Age36) & A$AGE09==36]<-A$A09[is.na(A$NA_Age36)
&A$AGE09==36]
table(A$NA_Age36,useNA = "always")
A$NA_Age36[is.na(A$NA_Age36) & A$AGE10==36]<-A$A10[is.na(A$NA_Age36)
&A$AGE10==36]
table(A$NA_Age36,useNA = "always")
A$NA_Age36[is.na(A$NA_Age36) & A$AGE11==36]<-A$A11[is.na(A$NA_Age36)
&A$AGE11==36]
table(A$NA_Age36,useNA = "always")
A$NA_Age36[is.na(A$NA_Age36) & A$AGE12==36]<-A$A12[is.na(A$NA_Age36)
&A$AGE12==36]
table(A$NA_Age36,useNA = "always")
A$NA_Age36[is.na(A$NA_Age36) & A$AGE13==36]<-A$A13[is.na(A$NA_Age36)
&A$AGE13==36]
table(A$NA_Age36,useNA = "always")
A$NA_Age36[is.na(A$NA_Age36) & A$AGE14==36]<-A$A14[is.na(A$NA_Age36)
&A$AGE14==36]
table(A$NA_Age36,useNA = "always")
A$NA_Age36[is.na(A$NA_Age36) & A$AGE15==36]<-A$A15[is.na(A$NA_Age36)
&A$AGE15==36]
table(A$NA_Age36,useNA = "always")
A$NA_Age36[is.na(A$NA_Age36) & A$AGE16==36]<-A$A16[is.na(A$NA_Age36)
&A$AGE16==36]
table(A$NA_Age36,useNA = "always")
A$NA_Age36[is.na(A$NA_Age36) & A$AGE17==36]<-A$A17[is.na(A$NA_Age36)
&A$AGE17==36]
table(A$NA_Age36,useNA = "always")
A$NA_Age36[A$Dec_Age36==1]<-NA
table(A$NA_Age36,useNA = "always")
## NA_Age37
table(A$AGE17)
A$NA_Age37<-NA
A$NA_Age37[is.na(A$NA_Age37) & A$AGE92==37]<-A$A92[is.na(A$NA_Age37) &
A$AGE92==37]
table(A$NA_Age37,useNA = "always")
A$NA_Age37[is.na(A$NA_Age37) &A$AGE93==37]<-A$A93[is.na(A$NA_Age37)
&A$AGE93==37]
table(A$NA_Age37,useNA = "always")
A$NA_Age37[is.na(A$NA_Age37) &A$AGE94==37]<-A$A94[is.na(A$NA_Age37)
&A$AGE94==37]
table(A$NA_Age37,useNA = "always")
A$NA_Age37[is.na(A$NA_Age37) &A$AGE95==37]<-A$A95[is.na(A$NA_Age37)
&A$AGE95==37]
table(A$NA_Age37,useNA = "always")
A$NA_Age37[is.na(A$NA_Age37) &A$AGE96==37]<-A$A96[is.na(A$NA_Age37)
&A$AGE96==37]
table(A$NA_Age37,useNA = "always")
A$NA_Age37[is.na(A$NA_Age37) &A$AGE97==37]<-A$A97[is.na(A$NA_Age37)
&A$AGE97==37]
table(A$NA_Age37,useNA = "always")
A$NA_Age37[is.na(A$NA_Age37) &A$AGE98==37]<-A$A98[is.na(A$NA_Age37)
&A$AGE98==37]
table(A$NA_Age37,useNA = "always")
A$NA_Age37[is.na(A$NA_Age37) &A$AGE99==37]<-A$A99[is.na(A$NA_Age37)
&A$AGE99==37]
table(A$NA_Age37,useNA = "always")
```

© 2023 Silver IA et al. *JAMA Network Open.*

```
A$NA_Age37[is.na(A$NA_Age37) & A$AGE00==37]<-A$A00[is.na(A$NA_Age37)
&A$AGE00==37]
table(A$NA_Age37,useNA = "always")
A$NA_Age37[is.na(A$NA_Age37) & A$AGE01==37]<-A$A01[is.na(A$NA_Age37)
&A$AGE01==37]
table(A$NA_Age37,useNA = "always")
A$NA_Age37[is.na(A$NA_Age37) & A$AGE02==37]<-A$A02[is.na(A$NA_Age37)
&A$AGE02==37]
table(A$NA_Age37,useNA = "always")
A$NA_Age37[is.na(A$NA_Age37) & A$AGE03==37]<-A$A03[is.na(A$NA_Age37)
&A$AGE03==37]
table(A$NA_Age37,useNA = "always")
A$NA_Age37[is.na(A$NA_Age37) & A$AGE04==37]<-A$A04[is.na(A$NA_Age37)
&A$AGE04==37]
table(A$NA_Age37,useNA = "always")
A$NA_Age37[is.na(A$NA_Age37) & A$AGE05==37]<-A$A05[is.na(A$NA_Age37)
&A$AGE05==37]
table(A$NA_Age37,useNA = "always")
A$NA_Age37[is.na(A$NA_Age37) & A$AGE06==37]<-A$A06[is.na(A$NA_Age37)
&A$AGE06==37]
table(A$NA_Age37,useNA = "always")
A$NA_Age37[is.na(A$NA_Age37) & A$AGE07==37]<-A$A07[is.na(A$NA_Age37)
&A$AGE07==37]
table(A$NA_Age37,useNA = "always")
A$NA_Age37[is.na(A$NA_Age37) & A$AGE08==37]<-A$A08[is.na(A$NA_Age37)
&A$AGE08==37]
table(A$NA_Age37,useNA = "always")
A$NA_Age37[is.na(A$NA_Age37) & A$AGE09==37]<-A$A09[is.na(A$NA_Age37)
&A$AGE09==37]
table(A$NA_Age37,useNA = "always")
A$NA_Age37[is.na(A$NA_Age37) & A$AGE10==37]<-A$A10[is.na(A$NA_Age37)
&A$AGE10==37]
table(A$NA_Age37,useNA = "always")
A$NA_Age37[is.na(A$NA_Age37) & A$AGE11==37]<-A$A11[is.na(A$NA_Age37)
&A$AGE11==37]
table(A$NA_Age37,useNA = "always")
A$NA_Age37[is.na(A$NA_Age37) & A$AGE12==37]<-A$A12[is.na(A$NA_Age37)
&A$AGE12==37]
table(A$NA_Age37,useNA = "always")
A$NA_Age37[is.na(A$NA_Age37) & A$AGE13==37]<-A$A13[is.na(A$NA_Age37)
&A$AGE13==37]
table(A$NA_Age37,useNA = "always")
A$NA_Age37[is.na(A$NA_Age37) & A$AGE14==37]<-A$A14[is.na(A$NA_Age37)
&A$AGE14==37]
table(A$NA_Age37,useNA = "always")
A$NA_Age37[is.na(A$NA_Age37) & A$AGE15==37]<-A$A15[is.na(A$NA_Age37)
&A$AGE15==37]
table(A$NA_Age37,useNA = "always")
A$NA_Age37[is.na(A$NA_Age37) & A$AGE16==37]<-A$A16[is.na(A$NA_Age37)
&A$AGE16==37]
table(A$NA_Age37,useNA = "always")
A$NA_Age37[is.na(A$NA_Age37) & A$AGE17==37]<-A$A17[is.na(A$NA_Age37)
&A$AGE17==37]
table(A$NA_Age37,useNA = "always")
A$NA_Age37[A$Dec_Age37==1]<-NA
table(A$NA_Age37,useNA = "always")
```

© 2023 Silver IA et al. *JAMA Network Open.*

```
## NA_Age38
table(A$AGE17)
A$NA_Age38<-NA
A$NA_Age38[is.na(A$NA_Age38) & A$AGE92==38]<-A$A92[is.na(A$NA_Age38) &
A$AGE92==38]
table(A$NA_Age38,useNA = "always")
A$NA_Age38[is.na(A$NA_Age38) &A$AGE93==38]<-A$A93[is.na(A$NA_Age38)
&A$AGE93==38]
table(A$NA_Age38,useNA = "always")
A$NA_Age38[is.na(A$NA_Age38) &A$AGE94==38]<-A$A94[is.na(A$NA_Age38)
&A$AGE94==38]
table(A$NA_Age38,useNA = "always")
A$NA_Age38[is.na(A$NA_Age38) &A$AGE95==38]<-A$A95[is.na(A$NA_Age38)
&A$AGE95==38]
table(A$NA_Age38,useNA = "always")
A$NA_Age38[is.na(A$NA_Age38) &A$AGE96==38]<-A$A96[is.na(A$NA_Age38)
&A$AGE96==38]
table(A$NA_Age38,useNA = "always")
A$NA_Age38[is.na(A$NA_Age38) &A$AGE97==38]<-A$A97[is.na(A$NA_Age38)
&A$AGE97==38]
table(A$NA_Age38,useNA = "always")
A$NA_Age38[is.na(A$NA_Age38) &A$AGE98==38]<-A$A98[is.na(A$NA_Age38)
&A$AGE98==38]
table(A$NA_Age38,useNA = "always")
A$NA_Age38[is.na(A$NA_Age38) &A$AGE99==38]<-A$A99[is.na(A$NA_Age38)
&A$AGE99==38]
table(A$NA_Age38,useNA = "always")
A$NA_Age38[is.na(A$NA_Age38) & A$AGE00==38]<-A$A00[is.na(A$NA_Age38)
&A$AGE00==38]
table(A$NA_Age38,useNA = "always")
A$NA_Age38[is.na(A$NA_Age38) & A$AGE01==38]<-A$A01[is.na(A$NA_Age38)
&A$AGE01==38]
table(A$NA_Age38,useNA = "always")
A$NA_Age38[is.na(A$NA_Age38) & A$AGE02==38]<-A$A02[is.na(A$NA_Age38)
&A$AGE02==38]
table(A$NA_Age38,useNA = "always")
A$NA_Age38[is.na(A$NA_Age38) & A$AGE03==38]<-A$A03[is.na(A$NA_Age38)
&A$AGE03==38]
table(A$NA_Age38,useNA = "always")
A$NA_Age38[is.na(A$NA_Age38) & A$AGE04==38]<-A$A04[is.na(A$NA_Age38)
&A$AGE04==38]
table(A$NA_Age38,useNA = "always")
A$NA_Age38[is.na(A$NA_Age38) & A$AGE05==38]<-A$A05[is.na(A$NA_Age38)
&A$AGE05==38]
table(A$NA_Age38,useNA = "always")
A$NA_Age38[is.na(A$NA_Age38) & A$AGE06==38]<-A$A06[is.na(A$NA_Age38)
&A$AGE06==38]
table(A$NA_Age38,useNA = "always")
A$NA_Age38[is.na(A$NA_Age38) & A$AGE07==38]<-A$A07[is.na(A$NA_Age38)
&A$AGE07==38]
table(A$NA_Age38,useNA = "always")
A$NA_Age38[is.na(A$NA_Age38) & A$AGE08==38]<-A$A08[is.na(A$NA_Age38)
&A$AGE08==38]
table(A$NA_Age38,useNA = "always")
A$NA_Age38[is.na(A$NA_Age38) & A$AGE09==38]<-A$A09[is.na(A$NA_Age38)
&A$AGE09==38]
```

© 2023 Silver IA et al. *JAMA Network Open.*

```
table(A$NA_Age38,useNA = "always")
A$NA_Age38[is.na(A$NA_Age38) & A$AGE10==38]<-A$A10[is.na(A$NA_Age38)
&A$AGE10==38]
table(A$NA_Age38,useNA = "always")
A$NA_Age38[is.na(A$NA_Age38) & A$AGE11==38]<-A$A11[is.na(A$NA_Age38)
&A$AGE11==38]
table(A$NA_Age38,useNA = "always")
A$NA_Age38[is.na(A$NA_Age38) & A$AGE12==38]<-A$A12[is.na(A$NA_Age38)
&A$AGE12==38]
table(A$NA_Age38,useNA = "always")
A$NA_Age38[is.na(A$NA_Age38) & A$AGE13==38]<-A$A13[is.na(A$NA_Age38)
&A$AGE13==38]
table(A$NA_Age38,useNA = "always")
A$NA_Age38[is.na(A$NA_Age38) & A$AGE14==38]<-A$A14[is.na(A$NA_Age38)
&A$AGE14==38]
table(A$NA_Age38,useNA = "always")
A$NA_Age38[is.na(A$NA_Age38) & A$AGE15==38]<-A$A15[is.na(A$NA_Age38)
&A$AGE15==38]
table(A$NA_Age38,useNA = "always")
A$NA_Age38[is.na(A$NA_Age38) & A$AGE16==38]<-A$A16[is.na(A$NA_Age38)
&A$AGE16==38]
table(A$NA_Age38,useNA = "always")
A$NA_Age38[is.na(A$NA_Age38) & A$AGE17==38]<-A$A17[is.na(A$NA_Age38)
&A$AGE17==38]
table(A$NA_Age38,useNA = "always")
A$NA_Age38[A$Dec_Age38==1]<-NA
table(A$NA_Age38,useNA = "always")

# Number of Months Incarcerated by Age Measures
## NMI_Age7
names(A)
table(A$AGE92)
table(A$AGE93)
A$NMI_Age7<-NA
A$NMI_Age7[is.na(A$NMI_Age7) & A$AGE92==7]<-A$I92[is.na(A$NMI_Age7) &
A$AGE92==7]
table(A$NMI_Age7,useNA = "always")
A$NMI_Age7[A$Dec_Age7==1]<-NA
table(A$NMI_Age7,useNA = "always")
## NMI_Age8
table(A$AGE92)
table(A$AGE93)
table(A$AGE94)
A$NMI_Age8<-NA
A$NMI_Age8[is.na(A$NMI_Age8) & A$AGE92==8]<-A$I92[is.na(A$NMI_Age8) &
A$AGE92==8]
table(A$NMI_Age8,useNA = "always")
A$NMI_Age8[is.na(A$NMI_Age8) & A$AGE93==8]<-A$I93[is.na(A$NMI_Age8) &
A$AGE93==8]
table(A$NMI_Age8,useNA = "always")
A$NMI_Age8[A$Dec_Age8==1]<-NA
table(A$NMI_Age8,useNA = "always")
## NMI_Age9
table(A$AGE92)
table(A$AGE93)
table(A$AGE94)
```

© 2023 Silver IA et al. *JAMA Network Open.*

```
A$NMI_Age9<-NA
A$NMI_Age9[is.na(A$NMI_Age9) & A$AGE92==9]<-A$I92[is.na(A$NMI_Age9) &
A$AGE92==9]
table(A$NMI_Age9,useNA = "always")
A$NMI_Age9[is.na(A$NMI_Age9) & A$AGE93==9]<-A$I93[is.na(A$NMI_Age9) &
A$AGE93==9]
table(A$NMI_Age9,useNA = "always")
A$NMI_Age9[is.na(A$NMI_Age9) & A$AGE94==9]<-A$I94[is.na(A$NMI_Age9) &
A$AGE94==9]
table(A$NMI_Age9,useNA = "always")
A$NMI_Age9[A$Dec_Age9==1]<-NA
table(A$NMI_Age9,useNA = "always")
## NMI_Age10
table(A$AGE92)
table(A$AGE93)
table(A$AGE94)
table(A$AGE95)
A$NMI_Age10<-NA
A$NMI_Age10[is.na(A$NMI_Age10) & A$AGE92==10]<-A$I92[is.na(A$NMI_Age10) &
A$AGE92==10]
table(A$NMI_Age10,useNA = "always")
A$NMI_Age10[is.na(A$NMI_Age10) &A$AGE93==10]<-A$I93[is.na(A$NMI_Age10)
&A$AGE93==10]
table(A$NMI_Age10,useNA = "always")
A$NMI_Age10[is.na(A$NMI_Age10) &A$AGE94==10]<-A$I94[is.na(A$NMI_Age10)
&A$AGE94==10]
table(A$NMI_Age10,useNA = "always")
A$NMI_Age10[is.na(A$NMI_Age10) &A$AGE95==10]<-A$I95[is.na(A$NMI_Age10)
&A$AGE95==10]
table(A$NMI_Age10,useNA = "always")
A$NMI_Age10[A$Dec_Age10==1]<-NA
table(A$NMI_Age10,useNA = "always")
## NMI_Age11
table(A$AGE92)
table(A$AGE93)
table(A$AGE94)
table(A$AGE95)
table(A$AGE96)
A$NMI_Age11<-NA
A$NMI_Age11[is.na(A$NMI_Age11) & A$AGE92==11]<-A$I92[is.na(A$NMI_Age11) &
A$AGE92==11]
table(A$NMI_Age11,useNA = "always")
A$NMI_Age11[is.na(A$NMI_Age11) &A$AGE93==11]<-A$I93[is.na(A$NMI_Age11)
&A$AGE93==11]
table(A$NMI_Age11,useNA = "always")
A$NMI_Age11[is.na(A$NMI_Age11) &A$AGE94==11]<-A$I94[is.na(A$NMI_Age11)
&A$AGE94==11]
table(A$NMI_Age11,useNA = "always")
A$NMI_Age11[is.na(A$NMI_Age11) &A$AGE95==11]<-A$I95[is.na(A$NMI_Age11)
&A$AGE95==11]
table(A$NMI_Age11,useNA = "always")
A$NMI_Age11[is.na(A$NMI_Age11) &A$AGE96==11]<-A$I96[is.na(A$NMI_Age11)
&A$AGE96==11]
table(A$NMI_Age11,useNA = "always")
A$NMI_Age11[A$Dec_Age11==1]<-NA
table(A$NMI_Age11,useNA = "always")
```

© 2023 Silver IA et al. *JAMA Network Open.*

```
## NMI_Age12
table(A$AGE92)
table(A$AGE93)
table(A$AGE94)
table(A$AGE95)
table(A$AGE96)
table(A$AGE97)
A$NMI_Age12<-NA
A$NMI_Age12[is.na(A$NMI_Age12) & A$AGE92==12]<-A$I92[is.na(A$NMI_Age12) &
A$AGE92==12]
table(A$NMI_Age12,useNA = "always")
A$NMI_Age12[is.na(A$NMI_Age12) &A$AGE93==12]<-A$I93[is.na(A$NMI_Age12)
&A$AGE93==12]
table(A$NMI_Age12,useNA = "always")
A$NMI_Age12[is.na(A$NMI_Age12) &A$AGE94==12]<-A$I94[is.na(A$NMI_Age12)
&A$AGE94==12]
table(A$NMI_Age12,useNA = "always")
A$NMI_Age12[is.na(A$NMI_Age12) &A$AGE95==12]<-A$I95[is.na(A$NMI_Age12)
&A$AGE95==12]
table(A$NMI_Age12,useNA = "always")
A$NMI_Age12[is.na(A$NMI_Age12) &A$AGE96==12]<-A$I96[is.na(A$NMI_Age12)
&A$AGE96==12]
table(A$NMI_Age12,useNA = "always")
A$NMI_Age12[is.na(A$NMI_Age12) &A$AGE97==12]<-A$I97[is.na(A$NMI_Age12)
&A$AGE97==12]
table(A$NMI_Age12,useNA = "always")
A$NMI_Age12[A$Dec_Age12==1]<-NA
table(A$NMI_Age12,useNA = "always")
## NMI_Age13
table(A$AGE92)
table(A$AGE93)
table(A$AGE94)
table(A$AGE95)
table(A$AGE96)
table(A$AGE97)
table(A$AGE98)
A$NMI_Age13<-NA
A$NMI_Age13[is.na(A$NMI_Age13) & A$AGE92==13]<-A$I92[is.na(A$NMI_Age13) &
A$AGE92==13]
table(A$NMI_Age13,useNA = "always")
A$NMI_Age13[is.na(A$NMI_Age13) &A$AGE93==13]<-A$I93[is.na(A$NMI_Age13)
&A$AGE93==13]
table(A$NMI_Age13,useNA = "always")
A$NMI_Age13[is.na(A$NMI_Age13) &A$AGE94==13]<-A$I94[is.na(A$NMI_Age13)
&A$AGE94==13]
table(A$NMI_Age13,useNA = "always")
A$NMI_Age13[is.na(A$NMI_Age13) &A$AGE95==13]<-A$I95[is.na(A$NMI_Age13)
&A$AGE95==13]
table(A$NMI_Age13,useNA = "always")
A$NMI_Age13[is.na(A$NMI_Age13) &A$AGE96==13]<-A$I96[is.na(A$NMI_Age13)
&A$AGE96==13]
table(A$NMI_Age13,useNA = "always")
A$NMI_Age13[is.na(A$NMI_Age13) &A$AGE97==13]<-A$I97[is.na(A$NMI_Age13)
&A$AGE97==13]
table(A$NMI_Age13,useNA = "always")
```

© 2023 Silver IA et al. *JAMA Network Open.*

```
A$NMI_Age13[is.na(A$NMI_Age13) &A$AGE98==13]<-A$I98[is.na(A$NMI_Age13)
&A$AGE98==13]
table(A$NMI_Age13,useNA = "always")
A$NMI_Age13[A$Dec_Age13==1]<-NA
table(A$NMI_Age13,useNA = "always")
## NMI_Age14
table(A$AGE92)
table(A$AGE93)
table(A$AGE94)
table(A$AGE95)
table(A$AGE96)
table(A$AGE97)
table(A$AGE98)
table(A$AGE99)
A$NMI_Age14<-NA
A$NMI_Age14[is.na(A$NMI_Age14) & A$AGE92==14]<-A$I92[is.na(A$NMI_Age14) &
A$AGE92==14]
table(A$NMI_Age14,useNA = "always")
A$NMI_Age14[is.na(A$NMI_Age14) &A$AGE93==14]<-A$I93[is.na(A$NMI_Age14)
&A$AGE93==14]
table(A$NMI_Age14,useNA = "always")
A$NMI_Age14[is.na(A$NMI_Age14) &A$AGE94==14]<-A$I94[is.na(A$NMI_Age14)
&A$AGE94==14]
table(A$NMI_Age14,useNA = "always")
A$NMI_Age14[is.na(A$NMI_Age14) &A$AGE95==14]<-A$I95[is.na(A$NMI_Age14)
&A$AGE95==14]
table(A$NMI_Age14,useNA = "always")
A$NMI_Age14[is.na(A$NMI_Age14) &A$AGE96==14]<-A$I96[is.na(A$NMI_Age14)
&A$AGE96==14]
table(A$NMI_Age14,useNA = "always")
A$NMI_Age14[is.na(A$NMI_Age14) &A$AGE97==14]<-A$I97[is.na(A$NMI_Age14)
&A$AGE97==14]
table(A$NMI_Age14,useNA = "always")
A$NMI_Age14[is.na(A$NMI_Age14) &A$AGE98==14]<-A$I98[is.na(A$NMI_Age14)
&A$AGE98==14]
table(A$NMI_Age14,useNA = "always")
A$NMI_Age14[is.na(A$NMI_Age14) &A$AGE99==14]<-A$I99[is.na(A$NMI_Age14)
&A$AGE99==14]
table(A$NMI_Age14,useNA = "always")
A$NMI_Age14[A$Dec_Age14==1]<-NA
table(A$NMI_Age14,useNA = "always")
## NMI_Age15
table(A$AGE92)
table(A$AGE93)
table(A$AGE94)
table(A$AGE95)
table(A$AGE96)
table(A$AGE97)
table(A$AGE98)
table(A$AGE99)
table(A$AGE00)
A$NMI_Age15<-NA
A$NMI_Age15[is.na(A$NMI_Age15) & A$AGE92==15]<-A$I92[is.na(A$NMI_Age15) &
A$AGE92==15]
table(A$NMI_Age15,useNA = "always")
```

© 2023 Silver IA et al. *JAMA Network Open.*

```
A$NMI_Age15[is.na(A$NMI_Age15) &A$AGE93==15]<-A$I93[is.na(A$NMI_Age15)
&A$AGE93==15]
table(A$NMI_Age15,useNA = "always")
A$NMI_Age15[is.na(A$NMI_Age15) &A$AGE94==15]<-A$I94[is.na(A$NMI_Age15)
&A$AGE94==15]
table(A$NMI_Age15,useNA = "always")
A$NMI_Age15[is.na(A$NMI_Age15) &A$AGE95==15]<-A$I95[is.na(A$NMI_Age15)
&A$AGE95==15]
table(A$NMI_Age15,useNA = "always")
A$NMI_Age15[is.na(A$NMI_Age15) &A$AGE96==15]<-A$I96[is.na(A$NMI_Age15)
&A$AGE96==15]
table(A$NMI_Age15,useNA = "always")
A$NMI_Age15[is.na(A$NMI_Age15) &A$AGE97==15]<-A$I97[is.na(A$NMI_Age15)
&A$AGE97==15]
table(A$NMI_Age15,useNA = "always")
A$NMI_Age15[is.na(A$NMI_Age15) &A$AGE98==15]<-A$I98[is.na(A$NMI_Age15)
&A$AGE98==15]
table(A$NMI_Age15,useNA = "always")
A$NMI_Age15[is.na(A$NMI_Age15) &A$AGE99==15]<-A$I99[is.na(A$NMI_Age15)
&A$AGE99==15]
table(A$NMI_Age15,useNA = "always")
A$NMI_Age15[is.na(A$NMI_Age15) & A$AGE00==15]<-A$I00[is.na(A$NMI_Age15)
&A$AGE00==15]
table(A$NMI_Age15,useNA = "always")
A$NMI_Age15[A$Dec_Age15==1]<-NA
table(A$NMI_Age15,useNA = "always")
## NMI_Age16
table(A$AGE92)
table(A$AGE93)
table(A$AGE94)
table(A$AGE95)
table(A$AGE96)
table(A$AGE97)
table(A$AGE98)
table(A$AGE99)
table(A$AGE00)
table(A$AGE01)
A$NMI_Age16<-NA
A$NMI_Age16[is.na(A$NMI_Age16) & A$AGE92==16]<-A$I92[is.na(A$NMI_Age16) &
A$AGE92==16]
table(A$NMI_Age16,useNA = "always")
A$NMI_Age16[is.na(A$NMI_Age16) &A$AGE93==16]<-A$I93[is.na(A$NMI_Age16)
&A$AGE93==16]
table(A$NMI_Age16,useNA = "always")
A$NMI_Age16[is.na(A$NMI_Age16) &A$AGE94==16]<-A$I94[is.na(A$NMI_Age16)
&A$AGE94==16]
table(A$NMI_Age16,useNA = "always")
A$NMI_Age16[is.na(A$NMI_Age16) &A$AGE95==16]<-A$I95[is.na(A$NMI_Age16)
&A$AGE95==16]
table(A$NMI_Age16,useNA = "always")
A$NMI_Age16[is.na(A$NMI_Age16) &A$AGE96==16]<-A$I96[is.na(A$NMI_Age16)
&A$AGE96==16]
table(A$NMI_Age16,useNA = "always")
A$NMI_Age16[is.na(A$NMI_Age16) &A$AGE97==16]<-A$I97[is.na(A$NMI_Age16)
&A$AGE97==16]
table(A$NMI_Age16,useNA = "always")
```

© 2023 Silver IA et al. *JAMA Network Open.*

```
A$NMI_Age16[is.na(A$NMI_Age16) &A$AGE98==16]<-A$I98[is.na(A$NMI_Age16)
&A$AGE98==16]
table(A$NMI_Age16,useNA = "always")
A$NMI_Age16[is.na(A$NMI_Age16) &A$AGE99==16]<-A$I99[is.na(A$NMI_Age16)
&A$AGE99==16]
table(A$NMI_Age16,useNA = "always")
A$NMI_Age16[is.na(A$NMI_Age16) & A$AGE00==16]<-A$I00[is.na(A$NMI_Age16)
&A$AGE00==16]
table(A$NMI_Age16,useNA = "always")
A$NMI_Age16[is.na(A$NMI_Age16) & A$AGE01==16]<-A$I01[is.na(A$NMI_Age16)
&A$AGE01==16]
table(A$NMI_Age16,useNA = "always")
A$NMI_Age16[A$Dec_Age16==1]<-NA
table(A$NMI_Age16,useNA = "always")
## NMI_Age17
table(A$AGE92)
table(A$AGE93)
table(A$AGE94)
table(A$AGE95)
table(A$AGE96)
table(A$AGE97)
table(A$AGE98)
table(A$AGE99)
table(A$AGE00)
table(A$AGE01)
A$NMI_Age17<-NA
A$NMI_Age17[is.na(A$NMI_Age17) & A$AGE92==17]<-A$I92[is.na(A$NMI_Age17) &
A$AGE92==17]
table(A$NMI_Age17,useNA = "always")
A$NMI_Age17[is.na(A$NMI_Age17) &A$AGE93==17]<-A$I93[is.na(A$NMI_Age17)
&A$AGE93==17]
table(A$NMI_Age17,useNA = "always")
A$NMI_Age17[is.na(A$NMI_Age17) &A$AGE94==17]<-A$I94[is.na(A$NMI_Age17)
&A$AGE94==17]
table(A$NMI_Age17,useNA = "always")
A$NMI_Age17[is.na(A$NMI_Age17) &A$AGE95==17]<-A$I95[is.na(A$NMI_Age17)
&A$AGE95==17]
table(A$NMI_Age17,useNA = "always")
A$NMI_Age17[is.na(A$NMI_Age17) &A$AGE96==17]<-A$I96[is.na(A$NMI_Age17)
&A$AGE96==17]
table(A$NMI_Age17,useNA = "always")
A$NMI_Age17[is.na(A$NMI_Age17) &A$AGE97==17]<-A$I97[is.na(A$NMI_Age17)
&A$AGE97==17]
table(A$NMI_Age17,useNA = "always")
A$NMI_Age17[is.na(A$NMI_Age17) &A$AGE98==17]<-A$I98[is.na(A$NMI_Age17)
&A$AGE98==17]
table(A$NMI_Age17,useNA = "always")
A$NMI_Age17[is.na(A$NMI_Age17) &A$AGE99==17]<-A$I99[is.na(A$NMI_Age17)
&A$AGE99==17]
table(A$NMI_Age17,useNA = "always")
A$NMI_Age17[is.na(A$NMI_Age17) & A$AGE00==17]<-A$I00[is.na(A$NMI_Age17)
&A$AGE00==17]
table(A$NMI_Age17,useNA = "always")
A$NMI_Age17[is.na(A$NMI_Age17) & A$AGE01==17]<-A$I01[is.na(A$NMI_Age17)
&A$AGE01==17]
table(A$NMI_Age17,useNA = "always")
```

© 2023 Silver IA et al. *JAMA Network Open.*

```
A$NMI_Age17[is.na(A$NMI_Age17) & A$AGE02==17]<-A$I02[is.na(A$NMI_Age17)
&A$AGE02==17]
table(A$NMI_Age17,useNA = "always")
A$NMI_Age17[A$Dec_Age17==1]<-NA
table(A$NMI_Age17,useNA = "always")
## NMI_Age18
table(A$AGE92)
table(A$AGE93)
table(A$AGE94)
table(A$AGE95)
table(A$AGE96)
table(A$AGE97)
table(A$AGE98)
table(A$AGE99)
table(A$AGE00)
table(A$AGE01)
A$NMI_Age18<-NA
A$NMI_Age18[is.na(A$NMI_Age18) & A$AGE92==18]<-A$I92[is.na(A$NMI_Age18) &
A$AGE92==18]
table(A$NMI_Age18,useNA = "always")
A$NMI_Age18[is.na(A$NMI_Age18) &A$AGE93==18]<-A$I93[is.na(A$NMI_Age18)
&A$AGE93==18]
table(A$NMI_Age18,useNA = "always")
A$NMI_Age18[is.na(A$NMI_Age18) &A$AGE94==18]<-A$I94[is.na(A$NMI_Age18)
&A$AGE94==18]
table(A$NMI_Age18,useNA = "always")
A$NMI_Age18[is.na(A$NMI_Age18) &A$AGE95==18]<-A$I95[is.na(A$NMI_Age18)
&A$AGE95==18]
table(A$NMI_Age18,useNA = "always")
A$NMI_Age18[is.na(A$NMI_Age18) &A$AGE96==18]<-A$I96[is.na(A$NMI_Age18)
&A$AGE96==18]
table(A$NMI_Age18,useNA = "always")
A$NMI_Age18[is.na(A$NMI_Age18) &A$AGE97==18]<-A$I97[is.na(A$NMI_Age18)
&A$AGE97==18]
table(A$NMI_Age18,useNA = "always")
A$NMI_Age18[is.na(A$NMI_Age18) &A$AGE98==18]<-A$I98[is.na(A$NMI_Age18)
&A$AGE98==18]
table(A$NMI_Age18,useNA = "always")
A$NMI_Age18[is.na(A$NMI_Age18) &A$AGE99==18]<-A$I99[is.na(A$NMI_Age18)
&A$AGE99==18]
table(A$NMI_Age18,useNA = "always")
A$NMI_Age18[is.na(A$NMI_Age18) & A$AGE00==18]<-A$I00[is.na(A$NMI_Age18)
&A$AGE00==18]
table(A$NMI_Age18,useNA = "always")
A$NMI_Age18[is.na(A$NMI_Age18) & A$AGE01==18]<-A$I01[is.na(A$NMI_Age18)
&A$AGE01==18]
table(A$NMI_Age18,useNA = "always")
A$NMI_Age18[is.na(A$NMI_Age18) & A$AGE02==18]<-A$I02[is.na(A$NMI_Age18)
&A$AGE02==18]
table(A$NMI_Age18,useNA = "always")
A$NMI_Age18[is.na(A$NMI_Age18) & A$AGE03==18]<-A$I03[is.na(A$NMI_Age18)
&A$AGE03==18]
table(A$NMI_Age18,useNA = "always")
A$NMI_Age18[A$Dec_Age18==1]<-NA
table(A$NMI_Age18,useNA = "always")
## NMI_Age19
```

© 2023 Silver IA et al. *JAMA Network Open.*

```
A$NMI_Age19<-NA
A$NMI_Age19[is.na(A$NMI_Age19) & A$AGE92==19]<-A$I92[is.na(A$NMI_Age19) &
A$AGE92==19]
table(A$NMI_Age19,useNA = "always")
A$NMI_Age19[is.na(A$NMI_Age19) &A$AGE93==19]<-A$I93[is.na(A$NMI_Age19)
&A$AGE93==19]
table(A$NMI_Age19,useNA = "always")
A$NMI_Age19[is.na(A$NMI_Age19) &A$AGE94==19]<-A$I94[is.na(A$NMI_Age19)
&A$AGE94==19]
table(A$NMI_Age19,useNA = "always")
A$NMI_Age19[is.na(A$NMI_Age19) &A$AGE95==19]<-A$I95[is.na(A$NMI_Age19)
&A$AGE95==19]
table(A$NMI_Age19,useNA = "always")
A$NMI_Age19[is.na(A$NMI_Age19) &A$AGE96==19]<-A$I96[is.na(A$NMI_Age19)
&A$AGE96==19]
table(A$NMI_Age19,useNA = "always")
A$NMI_Age19[is.na(A$NMI_Age19) &A$AGE97==19]<-A$I97[is.na(A$NMI_Age19)
&A$AGE97==19]
table(A$NMI_Age19,useNA = "always")
A$NMI_Age19[is.na(A$NMI_Age19) &A$AGE98==19]<-A$I98[is.na(A$NMI_Age19)
&A$AGE98==19]
table(A$NMI_Age19,useNA = "always")
A$NMI_Age19[is.na(A$NMI_Age19) &A$AGE99==19]<-A$I99[is.na(A$NMI_Age19)
&A$AGE99==19]
table(A$NMI_Age19,useNA = "always")
A$NMI_Age19[is.na(A$NMI_Age19) & A$AGE00==19]<-A$I00[is.na(A$NMI_Age19)
&A$AGE00==19]
table(A$NMI_Age19,useNA = "always")
A$NMI_Age19[is.na(A$NMI_Age19) & A$AGE01==19]<-A$I01[is.na(A$NMI_Age19)
&A$AGE01==19]
table(A$NMI_Age19,useNA = "always")
A$NMI_Age19[is.na(A$NMI_Age19) & A$AGE02==19]<-A$I02[is.na(A$NMI_Age19)
&A$AGE02==19]
table(A$NMI_Age19,useNA = "always")
A$NMI_Age19[is.na(A$NMI_Age19) & A$AGE03==19]<-A$I03[is.na(A$NMI_Age19)
&A$AGE03==19]
table(A$NMI_Age19,useNA = "always")
A$NMI_Age19[is.na(A$NMI_Age19) & A$AGE04==19]<-A$I04[is.na(A$NMI_Age19)
&A$AGE04==19]
table(A$NMI_Age19,useNA = "always")
A$NMI_Age19[A$Dec_Age19==1]<-NA
table(A$NMI_Age19,useNA = "always")
## NMI_Age20
A$NMI_Age20<-NA
A$NMI_Age20[is.na(A$NMI_Age20) & A$AGE92==20]<-A$I92[is.na(A$NMI_Age20) &
A$AGE92==20]
table(A$NMI_Age20,useNA = "always")
A$NMI_Age20[is.na(A$NMI_Age20) &A$AGE93==20]<-A$I93[is.na(A$NMI_Age20)
&A$AGE93==20]
table(A$NMI_Age20,useNA = "always")
A$NMI_Age20[is.na(A$NMI_Age20) &A$AGE94==20]<-A$I94[is.na(A$NMI_Age20)
&A$AGE94==20]
table(A$NMI_Age20,useNA = "always")
A$NMI_Age20[is.na(A$NMI_Age20) &A$AGE95==20]<-A$I95[is.na(A$NMI_Age20)
&A$AGE95==20]
table(A$NMI_Age20,useNA = "always")
```

© 2023 Silver IA et al. *JAMA Network Open.*

```
A$NMI_Age20[is.na(A$NMI_Age20) &A$AGE96==20]<-A$I96[is.na(A$NMI_Age20)
&A$AGE96==20]
table(A$NMI_Age20,useNA = "always")
A$NMI_Age20[is.na(A$NMI_Age20) &A$AGE97==20]<-A$I97[is.na(A$NMI_Age20)
&A$AGE97==20]
table(A$NMI_Age20,useNA = "always")
A$NMI_Age20[is.na(A$NMI_Age20) &A$AGE98==20]<-A$I98[is.na(A$NMI_Age20)
&A$AGE98==20]
table(A$NMI_Age20,useNA = "always")
A$NMI_Age20[is.na(A$NMI_Age20) &A$AGE99==20]<-A$I99[is.na(A$NMI_Age20)
&A$AGE99==20]
table(A$NMI_Age20,useNA = "always")
A$NMI_Age20[is.na(A$NMI_Age20) & A$AGE00==20]<-A$I00[is.na(A$NMI_Age20)
&A$AGE00==20]
table(A$NMI_Age20,useNA = "always")
A$NMI_Age20[is.na(A$NMI_Age20) & A$AGE01==20]<-A$I01[is.na(A$NMI_Age20)
&A$AGE01==20]
table(A$NMI_Age20,useNA = "always")
A$NMI_Age20[is.na(A$NMI_Age20) & A$AGE02==20]<-A$I02[is.na(A$NMI_Age20)
&A$AGE02==20]
table(A$NMI_Age20,useNA = "always")
A$NMI_Age20[is.na(A$NMI_Age20) & A$AGE03==20]<-A$I03[is.na(A$NMI_Age20)
&A$AGE03==20]
table(A$NMI_Age20,useNA = "always")
A$NMI_Age20[is.na(A$NMI_Age20) & A$AGE04==20]<-A$I04[is.na(A$NMI_Age20)
&A$AGE04==20]
table(A$NMI_Age20,useNA = "always")
A$NMI_Age20[is.na(A$NMI_Age20) & A$AGE05==20]<-A$I05[is.na(A$NMI_Age20)
&A$AGE05==20]
table(A$NMI_Age20,useNA = "always")
A$NMI_Age20[A$Dec_Age20==1]<-NA
table(A$NMI_Age20,useNA = "always")
## NMI_Age21
A$NMI_Age21<-NA
A$NMI_Age21[is.na(A$NMI_Age21) & A$AGE92==21]<-A$I92[is.na(A$NMI_Age21) &
A$AGE92==21]
table(A$NMI_Age21,useNA = "always")
A$NMI_Age21[is.na(A$NMI_Age21) &A$AGE93==21]<-A$I93[is.na(A$NMI_Age21)
&A$AGE93==21]
table(A$NMI_Age21,useNA = "always")
A$NMI_Age21[is.na(A$NMI_Age21) &A$AGE94==21]<-A$I94[is.na(A$NMI_Age21)
&A$AGE94==21]
table(A$NMI_Age21,useNA = "always")
A$NMI_Age21[is.na(A$NMI_Age21) &A$AGE95==21]<-A$I95[is.na(A$NMI_Age21)
&A$AGE95==21]
table(A$NMI_Age21,useNA = "always")
A$NMI_Age21[is.na(A$NMI_Age21) &A$AGE96==21]<-A$I96[is.na(A$NMI_Age21)
&A$AGE96==21]
table(A$NMI_Age21,useNA = "always")
A$NMI_Age21[is.na(A$NMI_Age21) &A$AGE97==21]<-A$I97[is.na(A$NMI_Age21)
&A$AGE97==21]
table(A$NMI_Age21,useNA = "always")
A$NMI_Age21[is.na(A$NMI_Age21) &A$AGE98==21]<-A$I98[is.na(A$NMI_Age21)
&A$AGE98==21]
table(A$NMI_Age21,useNA = "always")
```

© 2023 Silver IA et al. *JAMA Network Open.*

```
A$NMI_Age21[is.na(A$NMI_Age21) &A$AGE99==21]<-A$I99[is.na(A$NMI_Age21)
&A$AGE99==21]
table(A$NMI_Age21,useNA = "always")
A$NMI_Age21[is.na(A$NMI_Age21) & A$AGE00==21]<-A$I00[is.na(A$NMI_Age21)
&A$AGE00==21]
table(A$NMI_Age21,useNA = "always")
A$NMI_Age21[is.na(A$NMI_Age21) & A$AGE01==21]<-A$I01[is.na(A$NMI_Age21)
&A$AGE01==21]
table(A$NMI_Age21,useNA = "always")
A$NMI_Age21[is.na(A$NMI_Age21) & A$AGE02==21]<-A$I02[is.na(A$NMI_Age21)
&A$AGE02==21]
table(A$NMI_Age21,useNA = "always")
A$NMI_Age21[is.na(A$NMI_Age21) & A$AGE03==21]<-A$I03[is.na(A$NMI_Age21)
&A$AGE03==21]
table(A$NMI_Age21,useNA = "always")
A$NMI_Age21[is.na(A$NMI_Age21) & A$AGE04==21]<-A$I04[is.na(A$NMI_Age21)
&A$AGE04==21]
table(A$NMI_Age21,useNA = "always")
A$NMI_Age21[is.na(A$NMI_Age21) & A$AGE05==21]<-A$I05[is.na(A$NMI_Age21)
&A$AGE05==21]
table(A$NMI_Age21,useNA = "always")
A$NMI_Age21[is.na(A$NMI_Age21) & A$AGE06==21]<-A$I06[is.na(A$NMI_Age21)
&A$AGE06==21]
table(A$NMI_Age21,useNA = "always")
A$NMI_Age21[A$Dec_Age21==1]<-NA
table(A$NMI_Age21,useNA = "always")
## NMI_Age22
A$NMI_Age22<-NA
A$NMI_Age22[is.na(A$NMI_Age22) & A$AGE92==22]<-A$I92[is.na(A$NMI_Age22) &
A$AGE92==22]
table(A$NMI_Age22,useNA = "always")
A$NMI_Age22[is.na(A$NMI_Age22) &A$AGE93==22]<-A$I93[is.na(A$NMI_Age22)
&A$AGE93==22]
table(A$NMI_Age22,useNA = "always")
A$NMI_Age22[is.na(A$NMI_Age22) &A$AGE94==22]<-A$I94[is.na(A$NMI_Age22)
&A$AGE94==22]
table(A$NMI_Age22,useNA = "always")
A$NMI_Age22[is.na(A$NMI_Age22) &A$AGE95==22]<-A$I95[is.na(A$NMI_Age22)
&A$AGE95==22]
table(A$NMI_Age22,useNA = "always")
A$NMI_Age22[is.na(A$NMI_Age22) &A$AGE96==22]<-A$I96[is.na(A$NMI_Age22)
&A$AGE96==22]
table(A$NMI_Age22,useNA = "always")
A$NMI_Age22[is.na(A$NMI_Age22) &A$AGE97==22]<-A$I97[is.na(A$NMI_Age22)
&A$AGE97==22]
table(A$NMI_Age22,useNA = "always")
A$NMI_Age22[is.na(A$NMI_Age22) &A$AGE98==22]<-A$I98[is.na(A$NMI_Age22)
&A$AGE98==22]
table(A$NMI_Age22,useNA = "always")
A$NMI_Age22[is.na(A$NMI_Age22) &A$AGE99==22]<-A$I99[is.na(A$NMI_Age22)
&A$AGE99==22]
table(A$NMI_Age22,useNA = "always")
A$NMI_Age22[is.na(A$NMI_Age22) & A$AGE00==22]<-A$I00[is.na(A$NMI_Age22)
&A$AGE00==22]
table(A$NMI_Age22,useNA = "always")
```

© 2023 Silver IA et al. *JAMA Network Open.*

```
A$NMI_Age22[is.na(A$NMI_Age22) & A$AGE01==22]<-A$I01[is.na(A$NMI_Age22)
&A$AGE01==22]
table(A$NMI_Age22,useNA = "always")
A$NMI_Age22[is.na(A$NMI_Age22) & A$AGE02==22]<-A$I02[is.na(A$NMI_Age22)
&A$AGE02==22]
table(A$NMI_Age22,useNA = "always")
A$NMI_Age22[is.na(A$NMI_Age22) & A$AGE03==22]<-A$I03[is.na(A$NMI_Age22)
&A$AGE03==22]
table(A$NMI_Age22,useNA = "always")
A$NMI_Age22[is.na(A$NMI_Age22) & A$AGE04==22]<-A$I04[is.na(A$NMI_Age22)
&A$AGE04==22]
table(A$NMI_Age22,useNA = "always")
A$NMI_Age22[is.na(A$NMI_Age22) & A$AGE05==22]<-A$I05[is.na(A$NMI_Age22)
&A$AGE05==22]
table(A$NMI_Age22,useNA = "always")
A$NMI_Age22[is.na(A$NMI_Age22) & A$AGE06==22]<-A$I06[is.na(A$NMI_Age22)
&A$AGE06==22]
table(A$NMI_Age22,useNA = "always")
A$NMI_Age22[is.na(A$NMI_Age22) & A$AGE07==22]<-A$I07[is.na(A$NMI_Age22)
&A$AGE07==22]
table(A$NMI_Age22,useNA = "always")
A$NMI_Age22[A$Dec_Age22==1]<-NA
table(A$NMI_Age22,useNA = "always")
## NMI_Age23
A$NMI_Age23<-NA
A$NMI_Age23[is.na(A$NMI_Age23) & A$AGE92==23]<-A$I92[is.na(A$NMI_Age23) &
A$AGE92==23]
table(A$NMI_Age23,useNA = "always")
A$NMI_Age23[is.na(A$NMI_Age23) &A$AGE93==23]<-A$I93[is.na(A$NMI_Age23)
&A$AGE93==23]
table(A$NMI_Age23,useNA = "always")
A$NMI_Age23[is.na(A$NMI_Age23) &A$AGE94==23]<-A$I94[is.na(A$NMI_Age23)
&A$AGE94==23]
table(A$NMI_Age23,useNA = "always")
A$NMI_Age23[is.na(A$NMI_Age23) &A$AGE95==23]<-A$I95[is.na(A$NMI_Age23)
&A$AGE95==23]
table(A$NMI_Age23,useNA = "always")
A$NMI_Age23[is.na(A$NMI_Age23) &A$AGE96==23]<-A$I96[is.na(A$NMI_Age23)
&A$AGE96==23]
table(A$NMI_Age23,useNA = "always")
A$NMI_Age23[is.na(A$NMI_Age23) &A$AGE97==23]<-A$I97[is.na(A$NMI_Age23)
&A$AGE97==23]
table(A$NMI_Age23,useNA = "always")
A$NMI_Age23[is.na(A$NMI_Age23) &A$AGE98==23]<-A$I98[is.na(A$NMI_Age23)
&A$AGE98==23]
table(A$NMI_Age23,useNA = "always")
A$NMI_Age23[is.na(A$NMI_Age23) &A$AGE99==23]<-A$I99[is.na(A$NMI_Age23)
&A$AGE99==23]
table(A$NMI_Age23,useNA = "always")
A$NMI_Age23[is.na(A$NMI_Age23) & A$AGE00==23]<-A$I00[is.na(A$NMI_Age23)
&A$AGE00==23]
table(A$NMI_Age23,useNA = "always")
A$NMI_Age23[is.na(A$NMI_Age23) & A$AGE01==23]<-A$I01[is.na(A$NMI_Age23)
&A$AGE01==23]
table(A$NMI_Age23,useNA = "always")
```

© 2023 Silver IA et al. *JAMA Network Open.*

```
A$NMI_Age23[is.na(A$NMI_Age23) & A$AGE02==23]<-A$I02[is.na(A$NMI_Age23)
&A$AGE02==23]
table(A$NMI_Age23,useNA = "always")
A$NMI_Age23[is.na(A$NMI_Age23) & A$AGE03==23]<-A$I03[is.na(A$NMI_Age23)
&A$AGE03==23]
table(A$NMI_Age23,useNA = "always")
A$NMI_Age23[is.na(A$NMI_Age23) & A$AGE04==23]<-A$I04[is.na(A$NMI_Age23)
&A$AGE04==23]
table(A$NMI_Age23,useNA = "always")
A$NMI_Age23[is.na(A$NMI_Age23) & A$AGE05==23]<-A$I05[is.na(A$NMI_Age23)
&A$AGE05==23]
table(A$NMI_Age23,useNA = "always")
A$NMI_Age23[is.na(A$NMI_Age23) & A$AGE06==23]<-A$I06[is.na(A$NMI_Age23)
&A$AGE06==23]
table(A$NMI_Age23,useNA = "always")
A$NMI_Age23[is.na(A$NMI_Age23) & A$AGE07==23]<-A$I07[is.na(A$NMI_Age23)
&A$AGE07==23]
table(A$NMI_Age23,useNA = "always")
A$NMI_Age23[is.na(A$NMI_Age23) & A$AGE08==23]<-A$I08[is.na(A$NMI_Age23)
&A$AGE08==23]
table(A$NMI_Age23,useNA = "always")
A$NMI_Age23[A$Dec_Age23==1]<-NA
table(A$NMI_Age23,useNA = "always")
## NMI_Age24
A$NMI_Age24<-NA
A$NMI_Age24[is.na(A$NMI_Age24) & A$AGE92==24]<-A$I92[is.na(A$NMI_Age24) &
A$AGE92==24]
table(A$NMI_Age24,useNA = "always")
A$NMI_Age24[is.na(A$NMI_Age24) &A$AGE93==24]<-A$I93[is.na(A$NMI_Age24)
&A$AGE93==24]
table(A$NMI_Age24,useNA = "always")
A$NMI_Age24[is.na(A$NMI_Age24) &A$AGE94==24]<-A$I94[is.na(A$NMI_Age24)
&A$AGE94==24]
table(A$NMI_Age24,useNA = "always")
A$NMI_Age24[is.na(A$NMI_Age24) &A$AGE95==24]<-A$I95[is.na(A$NMI_Age24)
&A$AGE95==24]
table(A$NMI_Age24,useNA = "always")
A$NMI_Age24[is.na(A$NMI_Age24) &A$AGE96==24]<-A$I96[is.na(A$NMI_Age24)
&A$AGE96==24]
table(A$NMI_Age24,useNA = "always")
A$NMI_Age24[is.na(A$NMI_Age24) &A$AGE97==24]<-A$I97[is.na(A$NMI_Age24)
&A$AGE97==24]
table(A$NMI_Age24,useNA = "always")
A$NMI_Age24[is.na(A$NMI_Age24) &A$AGE98==24]<-A$I98[is.na(A$NMI_Age24)
&A$AGE98==24]
table(A$NMI_Age24,useNA = "always")
A$NMI_Age24[is.na(A$NMI_Age24) &A$AGE99==24]<-A$I99[is.na(A$NMI_Age24)
&A$AGE99==24]
table(A$NMI_Age24,useNA = "always")
A$NMI_Age24[is.na(A$NMI_Age24) & A$AGE00==24]<-A$I00[is.na(A$NMI_Age24)
&A$AGE00==24]
table(A$NMI_Age24,useNA = "always")
A$NMI_Age24[is.na(A$NMI_Age24) & A$AGE01==24]<-A$I01[is.na(A$NMI_Age24)
&A$AGE01==24]
table(A$NMI_Age24,useNA = "always")
```

© 2023 Silver IA et al. *JAMA Network Open.*

```
A$NMI_Age24[is.na(A$NMI_Age24) & A$AGE02==24]<-A$I02[is.na(A$NMI_Age24)
&A$AGE02==24]
table(A$NMI_Age24,useNA = "always")
A$NMI_Age24[is.na(A$NMI_Age24) & A$AGE03==24]<-A$I03[is.na(A$NMI_Age24)
&A$AGE03==24]
table(A$NMI_Age24,useNA = "always")
A$NMI_Age24[is.na(A$NMI_Age24) & A$AGE04==24]<-A$I04[is.na(A$NMI_Age24)
&A$AGE04==24]
table(A$NMI_Age24,useNA = "always")
A$NMI_Age24[is.na(A$NMI_Age24) & A$AGE05==24]<-A$I05[is.na(A$NMI_Age24)
&A$AGE05==24]
table(A$NMI_Age24,useNA = "always")
A$NMI_Age24[is.na(A$NMI_Age24) & A$AGE06==24]<-A$I06[is.na(A$NMI_Age24)
&A$AGE06==24]
table(A$NMI_Age24,useNA = "always")
A$NMI_Age24[is.na(A$NMI_Age24) & A$AGE07==24]<-A$I07[is.na(A$NMI_Age24)
&A$AGE07==24]
table(A$NMI_Age24,useNA = "always")
A$NMI_Age24[is.na(A$NMI_Age24) & A$AGE08==24]<-A$I08[is.na(A$NMI_Age24)
&A$AGE08==24]
table(A$NMI_Age24,useNA = "always")
A$NMI_Age24[is.na(A$NMI_Age24) & A$AGE09==24]<-A$I09[is.na(A$NMI_Age24)
&A$AGE09==24]
table(A$NMI_Age24,useNA = "always")
A$NMI_Age24[A$Dec_Age24==1]<-NA
table(A$NMI_Age24,useNA = "always")
## NMI_Age25
A$NMI_Age25<-NA
A$NMI_Age25[is.na(A$NMI_Age25) & A$AGE92==25]<-A$I92[is.na(A$NMI_Age25) &
A$AGE92==25]
table(A$NMI_Age25,useNA = "always")
A$NMI_Age25[is.na(A$NMI_Age25) &A$AGE93==25]<-A$I93[is.na(A$NMI_Age25)
&A$AGE93==25]
table(A$NMI_Age25,useNA = "always")
A$NMI_Age25[is.na(A$NMI_Age25) &A$AGE94==25]<-A$I94[is.na(A$NMI_Age25)
&A$AGE94==25]
table(A$NMI_Age25,useNA = "always")
A$NMI_Age25[is.na(A$NMI_Age25) &A$AGE95==25]<-A$I95[is.na(A$NMI_Age25)
&A$AGE95==25]
table(A$NMI_Age25,useNA = "always")
A$NMI_Age25[is.na(A$NMI_Age25) &A$AGE96==25]<-A$I96[is.na(A$NMI_Age25)
&A$AGE96==25]
table(A$NMI_Age25,useNA = "always")
A$NMI_Age25[is.na(A$NMI_Age25) &A$AGE97==25]<-A$I97[is.na(A$NMI_Age25)
&A$AGE97==25]
table(A$NMI_Age25,useNA = "always")
A$NMI_Age25[is.na(A$NMI_Age25) &A$AGE98==25]<-A$I98[is.na(A$NMI_Age25)
&A$AGE98==25]
table(A$NMI_Age25,useNA = "always")
A$NMI_Age25[is.na(A$NMI_Age25) &A$AGE99==25]<-A$I99[is.na(A$NMI_Age25)
&A$AGE99==25]
table(A$NMI_Age25,useNA = "always")
A$NMI_Age25[is.na(A$NMI_Age25) & A$AGE00==25]<-A$I00[is.na(A$NMI_Age25)
&A$AGE00==25]
table(A$NMI_Age25,useNA = "always")
```

© 2023 Silver IA et al. *JAMA Network Open.*

```
A$NMI_Age25[is.na(A$NMI_Age25) & A$AGE01==25]<-A$I01[is.na(A$NMI_Age25)
&A$AGE01==25]
table(A$NMI_Age25,useNA = "always")
A$NMI_Age25[is.na(A$NMI_Age25) & A$AGE02==25]<-A$I02[is.na(A$NMI_Age25)
&A$AGE02==25]
table(A$NMI_Age25,useNA = "always")
A$NMI_Age25[is.na(A$NMI_Age25) & A$AGE03==25]<-A$I03[is.na(A$NMI_Age25)
&A$AGE03==25]
table(A$NMI_Age25,useNA = "always")
A$NMI_Age25[is.na(A$NMI_Age25) & A$AGE04==25]<-A$I04[is.na(A$NMI_Age25)
&A$AGE04==25]
table(A$NMI_Age25,useNA = "always")
A$NMI_Age25[is.na(A$NMI_Age25) & A$AGE05==25]<-A$I05[is.na(A$NMI_Age25)
&A$AGE05==25]
table(A$NMI_Age25,useNA = "always")
A$NMI_Age25[is.na(A$NMI_Age25) & A$AGE06==25]<-A$I06[is.na(A$NMI_Age25)
&A$AGE06==25]
table(A$NMI_Age25,useNA = "always")
A$NMI_Age25[is.na(A$NMI_Age25) & A$AGE07==25]<-A$I07[is.na(A$NMI_Age25)
&A$AGE07==25]
table(A$NMI_Age25,useNA = "always")
A$NMI_Age25[is.na(A$NMI_Age25) & A$AGE08==25]<-A$I08[is.na(A$NMI_Age25)
&A$AGE08==25]
table(A$NMI_Age25,useNA = "always")
A$NMI_Age25[is.na(A$NMI_Age25) & A$AGE09==25]<-A$I09[is.na(A$NMI_Age25)
&A$AGE09==25]
table(A$NMI_Age25,useNA = "always")
A$NMI_Age25[is.na(A$NMI_Age25) & A$AGE10==25]<-A$I10[is.na(A$NMI_Age25)
&A$AGE10==25]
table(A$NMI_Age25,useNA = "always")
A$NMI_Age25[A$Dec_Age25==1]<-NA
table(A$NMI_Age25,useNA = "always")
## NMI_Age26
table(A$AGE11)
A$NMI_Age26<-NA
A$NMI_Age26[is.na(A$NMI_Age26) & A$AGE92==26]<-A$I92[is.na(A$NMI_Age26) &
A$AGE92==26]
table(A$NMI_Age26,useNA = "always")
A$NMI_Age26[is.na(A$NMI_Age26) &A$AGE93==26]<-A$I93[is.na(A$NMI_Age26)
&A$AGE93==26]
table(A$NMI_Age26,useNA = "always")
A$NMI_Age26[is.na(A$NMI_Age26) &A$AGE94==26]<-A$I94[is.na(A$NMI_Age26)
&A$AGE94==26]
table(A$NMI_Age26,useNA = "always")
A$NMI_Age26[is.na(A$NMI_Age26) &A$AGE95==26]<-A$I95[is.na(A$NMI_Age26)
&A$AGE95==26]
table(A$NMI_Age26,useNA = "always")
A$NMI_Age26[is.na(A$NMI_Age26) &A$AGE96==26]<-A$I96[is.na(A$NMI_Age26)
&A$AGE96==26]
table(A$NMI_Age26,useNA = "always")
A$NMI_Age26[is.na(A$NMI_Age26) &A$AGE97==26]<-A$I97[is.na(A$NMI_Age26)
&A$AGE97==26]
table(A$NMI_Age26,useNA = "always")
A$NMI_Age26[is.na(A$NMI_Age26) &A$AGE98==26]<-A$I98[is.na(A$NMI_Age26)
&A$AGE98==26]
table(A$NMI_Age26,useNA = "always")
```

```
A$NMI_Age26[is.na(A$NMI_Age26) &A$AGE99==26]<-A$I99[is.na(A$NMI_Age26)
&A$AGE99==26]
table(A$NMI_Age26,useNA = "always")
A$NMI_Age26[is.na(A$NMI_Age26) & A$AGE00==26]<-A$I00[is.na(A$NMI_Age26)
&A$AGE00==26]
table(A$NMI_Age26,useNA = "always")
A$NMI_Age26[is.na(A$NMI_Age26) & A$AGE01==26]<-A$I01[is.na(A$NMI_Age26)
&A$AGE01==26]
table(A$NMI_Age26,useNA = "always")
A$NMI_Age26[is.na(A$NMI_Age26) & A$AGE02==26]<-A$I02[is.na(A$NMI_Age26)
&A$AGE02==26]
table(A$NMI_Age26,useNA = "always")
A$NMI_Age26[is.na(A$NMI_Age26) & A$AGE03==26]<-A$I03[is.na(A$NMI_Age26)
&A$AGE03==26]
table(A$NMI_Age26,useNA = "always")
A$NMI_Age26[is.na(A$NMI_Age26) & A$AGE04==26]<-A$I04[is.na(A$NMI_Age26)
&A$AGE04==26]
table(A$NMI_Age26,useNA = "always")
A$NMI_Age26[is.na(A$NMI_Age26) & A$AGE05==26]<-A$I05[is.na(A$NMI_Age26)
&A$AGE05==26]
table(A$NMI_Age26,useNA = "always")
A$NMI_Age26[is.na(A$NMI_Age26) & A$AGE06==26]<-A$I06[is.na(A$NMI_Age26)
&A$AGE06==26]
table(A$NMI_Age26,useNA = "always")
A$NMI_Age26[is.na(A$NMI_Age26) & A$AGE07==26]<-A$I07[is.na(A$NMI_Age26)
&A$AGE07==26]
table(A$NMI_Age26,useNA = "always")
A$NMI_Age26[is.na(A$NMI_Age26) & A$AGE08==26]<-A$I08[is.na(A$NMI_Age26)
&A$AGE08==26]
table(A$NMI_Age26,useNA = "always")
A$NMI_Age26[is.na(A$NMI_Age26) & A$AGE09==26]<-A$I09[is.na(A$NMI_Age26)
&A$AGE09==26]
table(A$NMI_Age26,useNA = "always")
A$NMI_Age26[is.na(A$NMI_Age26) & A$AGE10==26]<-A$I10[is.na(A$NMI_Age26)
&A$AGE10==26]
table(A$NMI_Age26,useNA = "always")
A$NMI_Age26[is.na(A$NMI_Age26) & A$AGE11==26]<-A$I11[is.na(A$NMI_Age26)
&A$AGE11==26]
table(A$NMI_Age26,useNA = "always")
A$NMI_Age26[A$Dec_Age26==1]<-NA
table(A$NMI_Age26,useNA = "always")
## NMI_Age27
table(A$AGE17)
A$NMI_Age27<-NA
A$NMI_Age27[is.na(A$NMI_Age27) & A$AGE92==27]<-A$I92[is.na(A$NMI_Age27) &
A$AGE92==27]
table(A$NMI_Age27,useNA = "always")
A$NMI_Age27[is.na(A$NMI_Age27) &A$AGE93==27]<-A$I93[is.na(A$NMI_Age27)
&A$AGE93==27]
table(A$NMI_Age27,useNA = "always")
A$NMI_Age27[is.na(A$NMI_Age27) &A$AGE94==27]<-A$I94[is.na(A$NMI_Age27)
&A$AGE94==27]
table(A$NMI_Age27,useNA = "always")
A$NMI_Age27[is.na(A$NMI_Age27) &A$AGE95==27]<-A$I95[is.na(A$NMI_Age27)
&A$AGE95==27]
table(A$NMI_Age27,useNA = "always")
```

© 2023 Silver IA et al. *JAMA Network Open.*

```
A$NMI_Age27[is.na(A$NMI_Age27) &A$AGE96==27]<-A$I96[is.na(A$NMI_Age27)
&A$AGE96==27]
table(A$NMI_Age27,useNA = "always")
A$NMI_Age27[is.na(A$NMI_Age27) &A$AGE97==27]<-A$I97[is.na(A$NMI_Age27)
&A$AGE97==27]
table(A$NMI_Age27,useNA = "always")
A$NMI_Age27[is.na(A$NMI_Age27) &A$AGE98==27]<-A$I98[is.na(A$NMI_Age27)
&A$AGE98==27]
table(A$NMI_Age27,useNA = "always")
A$NMI_Age27[is.na(A$NMI_Age27) &A$AGE99==27]<-A$I99[is.na(A$NMI_Age27)
&A$AGE99==27]
table(A$NMI_Age27,useNA = "always")
A$NMI_Age27[is.na(A$NMI_Age27) & A$AGE00==27]<-A$I00[is.na(A$NMI_Age27)
&A$AGE00==27]
table(A$NMI_Age27,useNA = "always")
A$NMI_Age27[is.na(A$NMI_Age27) & A$AGE01==27]<-A$I01[is.na(A$NMI_Age27)
&A$AGE01==27]
table(A$NMI_Age27,useNA = "always")
A$NMI_Age27[is.na(A$NMI_Age27) & A$AGE02==27]<-A$I02[is.na(A$NMI_Age27)
&A$AGE02==27]
table(A$NMI_Age27,useNA = "always")
A$NMI_Age27[is.na(A$NMI_Age27) & A$AGE03==27]<-A$I03[is.na(A$NMI_Age27)
&A$AGE03==27]
table(A$NMI_Age27,useNA = "always")
A$NMI_Age27[is.na(A$NMI_Age27) & A$AGE04==27]<-A$I04[is.na(A$NMI_Age27)
&A$AGE04==27]
table(A$NMI_Age27,useNA = "always")
A$NMI_Age27[is.na(A$NMI_Age27) & A$AGE05==27]<-A$I05[is.na(A$NMI_Age27)
&A$AGE05==27]
table(A$NMI_Age27,useNA = "always")
A$NMI_Age27[is.na(A$NMI_Age27) & A$AGE06==27]<-A$I06[is.na(A$NMI_Age27)
&A$AGE06==27]
table(A$NMI_Age27,useNA = "always")
A$NMI_Age27[is.na(A$NMI_Age27) & A$AGE07==27]<-A$I07[is.na(A$NMI_Age27)
&A$AGE07==27]
table(A$NMI_Age27,useNA = "always")
A$NMI_Age27[is.na(A$NMI_Age27) & A$AGE08==27]<-A$I08[is.na(A$NMI_Age27)
&A$AGE08==27]
table(A$NMI_Age27,useNA = "always")
A$NMI_Age27[is.na(A$NMI_Age27) & A$AGE09==27]<-A$I09[is.na(A$NMI_Age27)
&A$AGE09==27]
table(A$NMI_Age27,useNA = "always")
A$NMI_Age27[is.na(A$NMI_Age27) & A$AGE10==27]<-A$I10[is.na(A$NMI_Age27)
&A$AGE10==27]
table(A$NMI_Age27,useNA = "always")
A$NMI_Age27[is.na(A$NMI_Age27) & A$AGE11==27]<-A$I11[is.na(A$NMI_Age27)
&A$AGE11==27]
table(A$NMI_Age27,useNA = "always")
A$NMI_Age27[is.na(A$NMI_Age27) & A$AGE12==27]<-A$I12[is.na(A$NMI_Age27)
&A$AGE12==27]
table(A$NMI_Age27,useNA = "always")
A$NMI_Age27[A$Dec_Age27==1]<-NA
table(A$NMI_Age27,useNA = "always")
## NMI_Age28
table(A$AGE17)
A$NMI_Age28<-NA
```

© 2023 Silver IA et al. *JAMA Network Open.*

```
A$NMI_Age28[is.na(A$NMI_Age28) & A$AGE92==28]<-A$I92[is.na(A$NMI_Age28) &
A$AGE92==28]
table(A$NMI_Age28,useNA = "always")
A$NMI_Age28[is.na(A$NMI_Age28) &A$AGE93==28]<-A$I93[is.na(A$NMI_Age28)
&A$AGE93==28]
table(A$NMI_Age28,useNA = "always")
A$NMI_Age28[is.na(A$NMI_Age28) &A$AGE94==28]<-A$I94[is.na(A$NMI_Age28)
&A$AGE94==28]
table(A$NMI_Age28,useNA = "always")
A$NMI_Age28[is.na(A$NMI_Age28) &A$AGE95==28]<-A$I95[is.na(A$NMI_Age28)
&A$AGE95==28]
table(A$NMI_Age28,useNA = "always")
A$NMI_Age28[is.na(A$NMI_Age28) &A$AGE96==28]<-A$I96[is.na(A$NMI_Age28)
&A$AGE96==28]
table(A$NMI_Age28,useNA = "always")
A$NMI_Age28[is.na(A$NMI_Age28) &A$AGE97==28]<-A$I97[is.na(A$NMI_Age28)
&A$AGE97==28]
table(A$NMI_Age28,useNA = "always")
A$NMI_Age28[is.na(A$NMI_Age28) &A$AGE98==28]<-A$I98[is.na(A$NMI_Age28)
&A$AGE98==28]
table(A$NMI_Age28,useNA = "always")
A$NMI_Age28[is.na(A$NMI_Age28) &A$AGE99==28]<-A$I99[is.na(A$NMI_Age28)
&A$AGE99==28]
table(A$NMI_Age28,useNA = "always")
A$NMI_Age28[is.na(A$NMI_Age28) & A$AGE00==28]<-A$I00[is.na(A$NMI_Age28)
&A$AGE00==28]
table(A$NMI_Age28,useNA = "always")
A$NMI_Age28[is.na(A$NMI_Age28) & A$AGE01==28]<-A$I01[is.na(A$NMI_Age28)
&A$AGE01==28]
table(A$NMI_Age28,useNA = "always")
A$NMI_Age28[is.na(A$NMI_Age28) & A$AGE02==28]<-A$I02[is.na(A$NMI_Age28)
&A$AGE02==28]
table(A$NMI_Age28,useNA = "always")
A$NMI_Age28[is.na(A$NMI_Age28) & A$AGE03==28]<-A$I03[is.na(A$NMI_Age28)
&A$AGE03==28]
table(A$NMI_Age28,useNA = "always")
A$NMI_Age28[is.na(A$NMI_Age28) & A$AGE04==28]<-A$I04[is.na(A$NMI_Age28)
&A$AGE04==28]
table(A$NMI_Age28,useNA = "always")
A$NMI_Age28[is.na(A$NMI_Age28) & A$AGE05==28]<-A$I05[is.na(A$NMI_Age28)
&A$AGE05==28]
table(A$NMI_Age28,useNA = "always")
A$NMI_Age28[is.na(A$NMI_Age28) & A$AGE06==28]<-A$I06[is.na(A$NMI_Age28)
&A$AGE06==28]
table(A$NMI_Age28,useNA = "always")
A$NMI_Age28[is.na(A$NMI_Age28) & A$AGE07==28]<-A$I07[is.na(A$NMI_Age28)
&A$AGE07==28]
table(A$NMI_Age28,useNA = "always")
A$NMI_Age28[is.na(A$NMI_Age28) & A$AGE08==28]<-A$I08[is.na(A$NMI_Age28)
&A$AGE08==28]
table(A$NMI_Age28,useNA = "always")
A$NMI_Age28[is.na(A$NMI_Age28) & A$AGE09==28]<-A$I09[is.na(A$NMI_Age28)
&A$AGE09==28]
table(A$NMI_Age28,useNA = "always")
A$NMI_Age28[is.na(A$NMI_Age28) & A$AGE10==28]<-A$I10[is.na(A$NMI_Age28)
&A$AGE10==28]
```

© 2023 Silver IA et al. *JAMA Network Open.*

```
table(A$NMI_Age28,useNA = "always")
A$NMI_Age28[is.na(A$NMI_Age28) & A$AGE11==28]<-A$I11[is.na(A$NMI_Age28)
&A$AGE11==28]
table(A$NMI_Age28,useNA = "always")
A$NMI_Age28[is.na(A$NMI_Age28) & A$AGE12==28]<-A$I12[is.na(A$NMI_Age28)
&A$AGE12==28]
table(A$NMI_Age28,useNA = "always")
A$NMI_Age28[is.na(A$NMI_Age28) & A$AGE13==28]<-A$I13[is.na(A$NMI_Age28)
&A$AGE13==28]
table(A$NMI_Age28,useNA = "always")
A$NMI_Age28[A$Dec_Age28==1]<-NA
table(A$NMI_Age28,useNA = "always")
## NMI_Age29
table(A$AGE17)
A$NMI_Age29<-NA
A$NMI_Age29[is.na(A$NMI_Age29) & A$AGE92==29]<-A$I92[is.na(A$NMI_Age29) &
A$AGE92==29]
table(A$NMI_Age29,useNA = "always")
A$NMI_Age29[is.na(A$NMI_Age29) &A$AGE93==29]<-A$I93[is.na(A$NMI_Age29)
&A$AGE93==29]
table(A$NMI_Age29,useNA = "always")
A$NMI_Age29[is.na(A$NMI_Age29) &A$AGE94==29]<-A$I94[is.na(A$NMI_Age29)
&A$AGE94==29]
table(A$NMI_Age29,useNA = "always")
A$NMI_Age29[is.na(A$NMI_Age29) &A$AGE95==29]<-A$I95[is.na(A$NMI_Age29)
&A$AGE95==29]
table(A$NMI_Age29,useNA = "always")
A$NMI_Age29[is.na(A$NMI_Age29) &A$AGE96==29]<-A$I96[is.na(A$NMI_Age29)
&A$AGE96==29]
table(A$NMI_Age29,useNA = "always")
A$NMI_Age29[is.na(A$NMI_Age29) &A$AGE97==29]<-A$I97[is.na(A$NMI_Age29)
&A$AGE97==29]
table(A$NMI_Age29,useNA = "always")
A$NMI_Age29[is.na(A$NMI_Age29) &A$AGE98==29]<-A$I98[is.na(A$NMI_Age29)
&A$AGE98==29]
table(A$NMI_Age29,useNA = "always")
A$NMI_Age29[is.na(A$NMI_Age29) &A$AGE99==29]<-A$I99[is.na(A$NMI_Age29)
&A$AGE99==29]
table(A$NMI_Age29,useNA = "always")
A$NMI_Age29[is.na(A$NMI_Age29) & A$AGE00==29]<-A$I00[is.na(A$NMI_Age29)
&A$AGE00==29]
table(A$NMI_Age29,useNA = "always")
A$NMI_Age29[is.na(A$NMI_Age29) & A$AGE01==29]<-A$I01[is.na(A$NMI_Age29)
&A$AGE01==29]
table(A$NMI_Age29,useNA = "always")
A$NMI_Age29[is.na(A$NMI_Age29) & A$AGE02==29]<-A$I02[is.na(A$NMI_Age29)
&A$AGE02==29]
table(A$NMI_Age29,useNA = "always")
A$NMI_Age29[is.na(A$NMI_Age29) & A$AGE03==29]<-A$I03[is.na(A$NMI_Age29)
&A$AGE03==29]
table(A$NMI_Age29,useNA = "always")
A$NMI_Age29[is.na(A$NMI_Age29) & A$AGE04==29]<-A$I04[is.na(A$NMI_Age29)
&A$AGE04==29]
table(A$NMI_Age29,useNA = "always")
A$NMI_Age29[is.na(A$NMI_Age29) & A$AGE05==29]<-A$I05[is.na(A$NMI_Age29)
&A$AGE05==29]
```

© 2023 Silver IA et al. *JAMA Network Open.*

```
table(A$NMI_Age29,useNA = "always")
A$NMI_Age29[is.na(A$NMI_Age29) & A$AGE06==29]<-A$I06[is.na(A$NMI_Age29)
&A$AGE06==29]
table(A$NMI_Age29,useNA = "always")
A$NMI_Age29[is.na(A$NMI_Age29) & A$AGE07==29]<-A$I07[is.na(A$NMI_Age29)
&A$AGE07==29]
table(A$NMI_Age29,useNA = "always")
A$NMI_Age29[is.na(A$NMI_Age29) & A$AGE08==29]<-A$I08[is.na(A$NMI_Age29)
&A$AGE08==29]
table(A$NMI_Age29,useNA = "always")
A$NMI_Age29[is.na(A$NMI_Age29) & A$AGE09==29]<-A$I09[is.na(A$NMI_Age29)
&A$AGE09==29]
table(A$NMI_Age29,useNA = "always")
A$NMI_Age29[is.na(A$NMI_Age29) & A$AGE10==29]<-A$I10[is.na(A$NMI_Age29)
&A$AGE10==29]
table(A$NMI_Age29,useNA = "always")
A$NMI_Age29[is.na(A$NMI_Age29) & A$AGE11==29]<-A$I11[is.na(A$NMI_Age29)
&A$AGE11==29]
table(A$NMI_Age29,useNA = "always")
A$NMI_Age29[is.na(A$NMI_Age29) & A$AGE12==29]<-A$I12[is.na(A$NMI_Age29)
&A$AGE12==29]
table(A$NMI_Age29,useNA = "always")
A$NMI_Age29[is.na(A$NMI_Age29) & A$AGE13==29]<-A$I13[is.na(A$NMI_Age29)
&A$AGE13==29]
table(A$NMI_Age29,useNA = "always")
A$NMI_Age29[is.na(A$NMI_Age29) & A$AGE14==29]<-A$I14[is.na(A$NMI_Age29)
&A$AGE14==29]
table(A$NMI_Age29,useNA = "always")
A$NMI_Age29[A$Dec_Age29==1]<-NA
table(A$NMI_Age29,useNA = "always")
## NMI_Age30
table(A$AGE17)
A$NMI_Age30<-NA
A$NMI_Age30[is.na(A$NMI_Age30) & A$AGE92==30]<-A$I92[is.na(A$NMI_Age30) &
A$AGE92==30]
table(A$NMI_Age30,useNA = "always")
A$NMI_Age30[is.na(A$NMI_Age30) &A$AGE93==30]<-A$I93[is.na(A$NMI_Age30)
&A$AGE93==30]
table(A$NMI_Age30,useNA = "always")
A$NMI_Age30[is.na(A$NMI_Age30) &A$AGE94==30]<-A$I94[is.na(A$NMI_Age30)
&A$AGE94==30]
table(A$NMI_Age30,useNA = "always")
A$NMI_Age30[is.na(A$NMI_Age30) &A$AGE95==30]<-A$I95[is.na(A$NMI_Age30)
&A$AGE95==30]
table(A$NMI_Age30,useNA = "always")
A$NMI_Age30[is.na(A$NMI_Age30) &A$AGE96==30]<-A$I96[is.na(A$NMI_Age30)
&A$AGE96==30]
table(A$NMI_Age30,useNA = "always")
A$NMI_Age30[is.na(A$NMI_Age30) &A$AGE97==30]<-A$I97[is.na(A$NMI_Age30)
&A$AGE97==30]
table(A$NMI_Age30,useNA = "always")
A$NMI_Age30[is.na(A$NMI_Age30) &A$AGE98==30]<-A$I98[is.na(A$NMI_Age30)
&A$AGE98==30]
table(A$NMI_Age30,useNA = "always")
A$NMI_Age30[is.na(A$NMI_Age30) &A$AGE99==30]<-A$I99[is.na(A$NMI_Age30)
&A$AGE99==30]
```

© 2023 Silver IA et al. *JAMA Network Open.*

```
table(A$NMI_Age30,useNA = "always")
A$NMI_Age30[is.na(A$NMI_Age30) & A$AGE00==30]<-A$I00[is.na(A$NMI_Age30)
&A$AGE00==30]
table(A$NMI_Age30,useNA = "always")
A$NMI_Age30[is.na(A$NMI_Age30) & A$AGE01==30]<-A$I01[is.na(A$NMI_Age30)
&A$AGE01==30]
table(A$NMI_Age30,useNA = "always")
A$NMI_Age30[is.na(A$NMI_Age30) & A$AGE02==30]<-A$I02[is.na(A$NMI_Age30)
&A$AGE02==30]
table(A$NMI_Age30,useNA = "always")
A$NMI_Age30[is.na(A$NMI_Age30) & A$AGE03==30]<-A$I03[is.na(A$NMI_Age30)
&A$AGE03==30]
table(A$NMI_Age30,useNA = "always")
A$NMI_Age30[is.na(A$NMI_Age30) & A$AGE04==30]<-A$I04[is.na(A$NMI_Age30)
&A$AGE04==30]
table(A$NMI_Age30,useNA = "always")
A$NMI_Age30[is.na(A$NMI_Age30) & A$AGE05==30]<-A$I05[is.na(A$NMI_Age30)
&A$AGE05==30]
table(A$NMI_Age30,useNA = "always")
A$NMI_Age30[is.na(A$NMI_Age30) & A$AGE06==30]<-A$I06[is.na(A$NMI_Age30)
&A$AGE06==30]
table(A$NMI_Age30,useNA = "always")
A$NMI_Age30[is.na(A$NMI_Age30) & A$AGE07==30]<-A$I07[is.na(A$NMI_Age30)
&A$AGE07==30]
table(A$NMI_Age30,useNA = "always")
A$NMI_Age30[is.na(A$NMI_Age30) & A$AGE08==30]<-A$I08[is.na(A$NMI_Age30)
&A$AGE08==30]
table(A$NMI_Age30,useNA = "always")
A$NMI_Age30[is.na(A$NMI_Age30) & A$AGE09==30]<-A$I09[is.na(A$NMI_Age30)
&A$AGE09==30]
table(A$NMI_Age30,useNA = "always")
A$NMI_Age30[is.na(A$NMI_Age30) & A$AGE10==30]<-A$I10[is.na(A$NMI_Age30)
&A$AGE10==30]
table(A$NMI_Age30,useNA = "always")
A$NMI_Age30[is.na(A$NMI_Age30) & A$AGE11==30]<-A$I11[is.na(A$NMI_Age30)
&A$AGE11==30]
table(A$NMI_Age30,useNA = "always")
A$NMI_Age30[is.na(A$NMI_Age30) & A$AGE12==30]<-A$I12[is.na(A$NMI_Age30)
&A$AGE12==30]
table(A$NMI_Age30,useNA = "always")
A$NMI_Age30[is.na(A$NMI_Age30) & A$AGE13==30]<-A$I13[is.na(A$NMI_Age30)
&A$AGE13==30]
table(A$NMI_Age30,useNA = "always")
A$NMI_Age30[is.na(A$NMI_Age30) & A$AGE14==30]<-A$I14[is.na(A$NMI_Age30)
&A$AGE14==30]
table(A$NMI_Age30,useNA = "always")
A$NMI_Age30[is.na(A$NMI_Age30) & A$AGE15==30]<-A$I15[is.na(A$NMI_Age30)
&A$AGE15==30]
table(A$NMI_Age30,useNA = "always")
A$NMI_Age30[A$Dec_Age30==1]<-NA
table(A$NMI_Age30,useNA = "always")
## NMI_Age31
table(A$AGE17)
A$NMI_Age31<-NA
A$NMI_Age31[is.na(A$NMI_Age31) & A$AGE92==31]<-A$I92[is.na(A$NMI_Age31) &
A$AGE92==31]
```

© 2023 Silver IA et al. *JAMA Network Open.*

```
table(A$NMI_Age31,useNA = "always")
A$NMI_Age31[is.na(A$NMI_Age31) &A$AGE93==31]<-A$I93[is.na(A$NMI_Age31)
&A$AGE93==31]
table(A$NMI_Age31,useNA = "always")
A$NMI_Age31[is.na(A$NMI_Age31) &A$AGE94==31]<-A$I94[is.na(A$NMI_Age31)
&A$AGE94==31]
table(A$NMI_Age31,useNA = "always")
A$NMI_Age31[is.na(A$NMI_Age31) &A$AGE95==31]<-A$I95[is.na(A$NMI_Age31)
&A$AGE95==31]
table(A$NMI_Age31,useNA = "always")
A$NMI_Age31[is.na(A$NMI_Age31) &A$AGE96==31]<-A$I96[is.na(A$NMI_Age31)
&A$AGE96==31]
table(A$NMI_Age31,useNA = "always")
A$NMI_Age31[is.na(A$NMI_Age31) &A$AGE97==31]<-A$I97[is.na(A$NMI_Age31)
&A$AGE97==31]
table(A$NMI_Age31,useNA = "always")
A$NMI_Age31[is.na(A$NMI_Age31) &A$AGE98==31]<-A$I98[is.na(A$NMI_Age31)
&A$AGE98==31]
table(A$NMI_Age31,useNA = "always")
A$NMI_Age31[is.na(A$NMI_Age31) &A$AGE99==31]<-A$I99[is.na(A$NMI_Age31)
&A$AGE99==31]
table(A$NMI_Age31,useNA = "always")
A$NMI_Age31[is.na(A$NMI_Age31) & A$AGE00==31]<-A$I00[is.na(A$NMI_Age31)
&A$AGE00==31]
table(A$NMI_Age31,useNA = "always")
A$NMI_Age31[is.na(A$NMI_Age31) & A$AGE01==31]<-A$I01[is.na(A$NMI_Age31)
&A$AGE01==31]
table(A$NMI_Age31,useNA = "always")
A$NMI_Age31[is.na(A$NMI_Age31) & A$AGE02==31]<-A$I02[is.na(A$NMI_Age31)
&A$AGE02==31]
table(A$NMI_Age31,useNA = "always")
A$NMI_Age31[is.na(A$NMI_Age31) & A$AGE03==31]<-A$I03[is.na(A$NMI_Age31)
&A$AGE03==31]
table(A$NMI_Age31,useNA = "always")
A$NMI_Age31[is.na(A$NMI_Age31) & A$AGE04==31]<-A$I04[is.na(A$NMI_Age31)
&A$AGE04==31]
table(A$NMI_Age31,useNA = "always")
A$NMI_Age31[is.na(A$NMI_Age31) & A$AGE05==31]<-A$I05[is.na(A$NMI_Age31)
&A$AGE05==31]
table(A$NMI_Age31,useNA = "always")
A$NMI_Age31[is.na(A$NMI_Age31) & A$AGE06==31]<-A$I06[is.na(A$NMI_Age31)
&A$AGE06==31]
table(A$NMI_Age31,useNA = "always")
A$NMI_Age31[is.na(A$NMI_Age31) & A$AGE07==31]<-A$I07[is.na(A$NMI_Age31)
&A$AGE07==31]
table(A$NMI_Age31,useNA = "always")
A$NMI_Age31[is.na(A$NMI_Age31) & A$AGE08==31]<-A$I08[is.na(A$NMI_Age31)
&A$AGE08==31]
table(A$NMI_Age31,useNA = "always")
A$NMI_Age31[is.na(A$NMI_Age31) & A$AGE09==31]<-A$I09[is.na(A$NMI_Age31)
&A$AGE09==31]
table(A$NMI_Age31,useNA = "always")
A$NMI_Age31[is.na(A$NMI_Age31) & A$AGE10==31]<-A$I10[is.na(A$NMI_Age31)
&A$AGE10==31]
table(A$NMI_Age31,useNA = "always")
```

© 2023 Silver IA et al. *JAMA Network Open.*

```
A$NMI_Age31[is.na(A$NMI_Age31) & A$AGE11==31]<-A$I11[is.na(A$NMI_Age31)
&A$AGE11==31]
table(A$NMI_Age31,useNA = "always")
A$NMI_Age31[is.na(A$NMI_Age31) & A$AGE12==31]<-A$I12[is.na(A$NMI_Age31)
&A$AGE12==31]
table(A$NMI_Age31,useNA = "always")
A$NMI_Age31[is.na(A$NMI_Age31) & A$AGE13==31]<-A$I13[is.na(A$NMI_Age31)
&A$AGE13==31]
table(A$NMI_Age31,useNA = "always")
A$NMI_Age31[is.na(A$NMI_Age31) & A$AGE14==31]<-A$I14[is.na(A$NMI_Age31)
&A$AGE14==31]
table(A$NMI_Age31,useNA = "always")
A$NMI_Age31[is.na(A$NMI_Age31) & A$AGE15==31]<-A$I15[is.na(A$NMI_Age31)
&A$AGE15==31]
table(A$NMI_Age31,useNA = "always")
A$NMI_Age31[is.na(A$NMI_Age31) & A$AGE16==31]<-A$I16[is.na(A$NMI_Age31)
&A$AGE16==31]
table(A$NMI_Age31,useNA = "always")
A$NMI_Age31[A$Dec_Age31==1]<-NA
table(A$NMI_Age31,useNA = "always")
## NMI_Age32
table(A$AGE17)
A$NMI_Age32<-NA
A$NMI_Age32[is.na(A$NMI_Age32) & A$AGE92==32]<-A$I92[is.na(A$NMI_Age32) &
A$AGE92==32]
table(A$NMI_Age32,useNA = "always")
A$NMI_Age32[is.na(A$NMI_Age32) &A$AGE93==32]<-A$I93[is.na(A$NMI_Age32)
&A$AGE93==32]
table(A$NMI_Age32,useNA = "always")
A$NMI_Age32[is.na(A$NMI_Age32) &A$AGE94==32]<-A$I94[is.na(A$NMI_Age32)
&A$AGE94==32]
table(A$NMI_Age32,useNA = "always")
A$NMI_Age32[is.na(A$NMI_Age32) &A$AGE95==32]<-A$I95[is.na(A$NMI_Age32)
&A$AGE95==32]
table(A$NMI_Age32,useNA = "always")
A$NMI_Age32[is.na(A$NMI_Age32) &A$AGE96==32]<-A$I96[is.na(A$NMI_Age32)
&A$AGE96==32]
table(A$NMI_Age32,useNA = "always")
A$NMI_Age32[is.na(A$NMI_Age32) &A$AGE97==32]<-A$I97[is.na(A$NMI_Age32)
&A$AGE97==32]
table(A$NMI_Age32,useNA = "always")
A$NMI_Age32[is.na(A$NMI_Age32) &A$AGE98==32]<-A$I98[is.na(A$NMI_Age32)
&A$AGE98==32]
table(A$NMI_Age32,useNA = "always")
A$NMI_Age32[is.na(A$NMI_Age32) &A$AGE99==32]<-A$I99[is.na(A$NMI_Age32)
&A$AGE99==32]
table(A$NMI_Age32,useNA = "always")
A$NMI_Age32[is.na(A$NMI_Age32) & A$AGE00==32]<-A$I00[is.na(A$NMI_Age32)
&A$AGE00==32]
table(A$NMI_Age32,useNA = "always")
A$NMI_Age32[is.na(A$NMI_Age32) & A$AGE01==32]<-A$I01[is.na(A$NMI_Age32)
&A$AGE01==32]
table(A$NMI_Age32,useNA = "always")
A$NMI_Age32[is.na(A$NMI_Age32) & A$AGE02==32]<-A$I02[is.na(A$NMI_Age32)
&A$AGE02==32]
table(A$NMI_Age32,useNA = "always")
```

```
A$NMI_Age32[is.na(A$NMI_Age32) & A$AGE03==32]<-A$I03[is.na(A$NMI_Age32)
&A$AGE03==32]
table(A$NMI_Age32,useNA = "always")
A$NMI_Age32[is.na(A$NMI_Age32) & A$AGE04==32]<-A$I04[is.na(A$NMI_Age32)
&A$AGE04==32]
table(A$NMI_Age32,useNA = "always")
A$NMI_Age32[is.na(A$NMI_Age32) & A$AGE05==32]<-A$I05[is.na(A$NMI_Age32)
&A$AGE05==32]
table(A$NMI_Age32,useNA = "always")
A$NMI_Age32[is.na(A$NMI_Age32) & A$AGE06==32]<-A$I06[is.na(A$NMI_Age32)
&A$AGE06==32]
table(A$NMI_Age32,useNA = "always")
A$NMI_Age32[is.na(A$NMI_Age32) & A$AGE07==32]<-A$I07[is.na(A$NMI_Age32)
&A$AGE07==32]
table(A$NMI_Age32,useNA = "always")
A$NMI_Age32[is.na(A$NMI_Age32) & A$AGE08==32]<-A$I08[is.na(A$NMI_Age32)
&A$AGE08==32]
table(A$NMI_Age32,useNA = "always")
A$NMI_Age32[is.na(A$NMI_Age32) & A$AGE09==32]<-A$I09[is.na(A$NMI_Age32)
&A$AGE09==32]
table(A$NMI_Age32,useNA = "always")
A$NMI_Age32[is.na(A$NMI_Age32) & A$AGE10==32]<-A$I10[is.na(A$NMI_Age32)
&A$AGE10==32]
table(A$NMI_Age32,useNA = "always")
A$NMI_Age32[is.na(A$NMI_Age32) & A$AGE11==32]<-A$I11[is.na(A$NMI_Age32)
&A$AGE11==32]
table(A$NMI_Age32,useNA = "always")
A$NMI_Age32[is.na(A$NMI_Age32) & A$AGE12==32]<-A$I12[is.na(A$NMI_Age32)
&A$AGE12==32]
table(A$NMI_Age32,useNA = "always")
A$NMI_Age32[is.na(A$NMI_Age32) & A$AGE13==32]<-A$I13[is.na(A$NMI_Age32)
&A$AGE13==32]
table(A$NMI_Age32,useNA = "always")
A$NMI_Age32[is.na(A$NMI_Age32) & A$AGE14==32]<-A$I14[is.na(A$NMI_Age32)
&A$AGE14==32]
table(A$NMI_Age32,useNA = "always")
A$NMI_Age32[is.na(A$NMI_Age32) & A$AGE15==32]<-A$I15[is.na(A$NMI_Age32)
&A$AGE15==32]
table(A$NMI_Age32,useNA = "always")
A$NMI_Age32[is.na(A$NMI_Age32) & A$AGE16==32]<-A$I16[is.na(A$NMI_Age32)
&A$AGE16==32]
table(A$NMI_Age32,useNA = "always")
A$NMI_Age32[is.na(A$NMI_Age32) & A$AGE17==32]<-A$I17[is.na(A$NMI_Age32)
&A$AGE17==32]
table(A$NMI_Age32,useNA = "always")
A$NMI_Age32[A$Dec_Age32==1]<-NA
table(A$NMI_Age32,useNA = "always")
## NMI_Age33
table(A$AGE17)
A$NMI_Age33<-NA
A$NMI_Age33[is.na(A$NMI_Age33) & A$AGE92==33]<-A$I92[is.na(A$NMI_Age33) &
A$AGE92==33]
table(A$NMI_Age33,useNA = "always")
A$NMI_Age33[is.na(A$NMI_Age33) &A$AGE93==33]<-A$I93[is.na(A$NMI_Age33)
&A$AGE93==33]
table(A$NMI_Age33,useNA = "always")
```

© 2023 Silver IA et al. *JAMA Network Open.*

```
A$NMI_Age33[is.na(A$NMI_Age33) &A$AGE94==33]<-A$I94[is.na(A$NMI_Age33)
&A$AGE94==33]
table(A$NMI_Age33,useNA = "always")
A$NMI_Age33[is.na(A$NMI_Age33) &A$AGE95==33]<-A$I95[is.na(A$NMI_Age33)
&A$AGE95==33]
table(A$NMI_Age33,useNA = "always")
A$NMI_Age33[is.na(A$NMI_Age33) &A$AGE96==33]<-A$I96[is.na(A$NMI_Age33)
&A$AGE96==33]
table(A$NMI_Age33,useNA = "always")
A$NMI_Age33[is.na(A$NMI_Age33) &A$AGE97==33]<-A$I97[is.na(A$NMI_Age33)
&A$AGE97==33]
table(A$NMI_Age33,useNA = "always")
A$NMI_Age33[is.na(A$NMI_Age33) &A$AGE98==33]<-A$I98[is.na(A$NMI_Age33)
&A$AGE98==33]
table(A$NMI_Age33,useNA = "always")
A$NMI_Age33[is.na(A$NMI_Age33) &A$AGE99==33]<-A$I99[is.na(A$NMI_Age33)
&A$AGE99==33]
table(A$NMI_Age33,useNA = "always")
A$NMI_Age33[is.na(A$NMI_Age33) & A$AGE00==33]<-A$I00[is.na(A$NMI_Age33)
&A$AGE00==33]
table(A$NMI_Age33,useNA = "always")
A$NMI_Age33[is.na(A$NMI_Age33) & A$AGE01==33]<-A$I01[is.na(A$NMI_Age33)
&A$AGE01==33]
table(A$NMI_Age33,useNA = "always")
A$NMI_Age33[is.na(A$NMI_Age33) & A$AGE02==33]<-A$I02[is.na(A$NMI_Age33)
&A$AGE02==33]
table(A$NMI_Age33,useNA = "always")
A$NMI_Age33[is.na(A$NMI_Age33) & A$AGE03==33]<-A$I03[is.na(A$NMI_Age33)
&A$AGE03==33]
table(A$NMI_Age33,useNA = "always")
A$NMI_Age33[is.na(A$NMI_Age33) & A$AGE04==33]<-A$I04[is.na(A$NMI_Age33)
&A$AGE04==33]
table(A$NMI_Age33,useNA = "always")
A$NMI_Age33[is.na(A$NMI_Age33) & A$AGE05==33]<-A$I05[is.na(A$NMI_Age33)
&A$AGE05==33]
table(A$NMI_Age33,useNA = "always")
A$NMI_Age33[is.na(A$NMI_Age33) & A$AGE06==33]<-A$I06[is.na(A$NMI_Age33)
&A$AGE06==33]
table(A$NMI_Age33,useNA = "always")
A$NMI_Age33[is.na(A$NMI_Age33) & A$AGE07==33]<-A$I07[is.na(A$NMI_Age33)
&A$AGE07==33]
table(A$NMI_Age33,useNA = "always")
A$NMI_Age33[is.na(A$NMI_Age33) & A$AGE08==33]<-A$I08[is.na(A$NMI_Age33)
&A$AGE08==33]
table(A$NMI_Age33,useNA = "always")
A$NMI_Age33[is.na(A$NMI_Age33) & A$AGE09==33]<-A$I09[is.na(A$NMI_Age33)
&A$AGE09==33]
table(A$NMI_Age33,useNA = "always")
A$NMI_Age33[is.na(A$NMI_Age33) & A$AGE10==33]<-A$I10[is.na(A$NMI_Age33)
&A$AGE10==33]
table(A$NMI_Age33,useNA = "always")
A$NMI_Age33[is.na(A$NMI_Age33) & A$AGE11==33]<-A$I11[is.na(A$NMI_Age33)
&A$AGE11==33]
table(A$NMI_Age33,useNA = "always")
A$NMI_Age33[is.na(A$NMI_Age33) & A$AGE12==33]<-A$I12[is.na(A$NMI_Age33)
&A$AGE12==33]
```

© 2023 Silver IA et al. *JAMA Network Open.*

```
table(A$NMI_Age33,useNA = "always")
A$NMI_Age33[is.na(A$NMI_Age33) & A$AGE13==33]<-A$I13[is.na(A$NMI_Age33)
&A$AGE13==33]
table(A$NMI_Age33,useNA = "always")
A$NMI_Age33[is.na(A$NMI_Age33) & A$AGE14==33]<-A$I14[is.na(A$NMI_Age33)
&A$AGE14==33]
table(A$NMI_Age33,useNA = "always")
A$NMI_Age33[is.na(A$NMI_Age33) & A$AGE15==33]<-A$I15[is.na(A$NMI_Age33)
&A$AGE15==33]
table(A$NMI_Age33,useNA = "always")
A$NMI_Age33[is.na(A$NMI_Age33) & A$AGE16==33]<-A$I16[is.na(A$NMI_Age33)
&A$AGE16==33]
table(A$NMI_Age33,useNA = "always")
A$NMI_Age33[is.na(A$NMI_Age33) & A$AGE17==33]<-A$I17[is.na(A$NMI_Age33)
&A$AGE17==33]
table(A$NMI_Age33,useNA = "always")
A$NMI_Age33[A$Dec_Age33==1]<-NA
table(A$NMI_Age33,useNA = "always")
## NMI_Age34
table(A$AGE17)
A$NMI_Age34<-NA
A$NMI_Age34[is.na(A$NMI_Age34) & A$AGE92==34]<-A$I92[is.na(A$NMI_Age34) &
A$AGE92==34]
table(A$NMI_Age34,useNA = "always")
A$NMI_Age34[is.na(A$NMI_Age34) &A$AGE93==34]<-A$I93[is.na(A$NMI_Age34)
&A$AGE93==34]
table(A$NMI_Age34,useNA = "always")
A$NMI_Age34[is.na(A$NMI_Age34) &A$AGE94==34]<-A$I94[is.na(A$NMI_Age34)
&A$AGE94==34]
table(A$NMI_Age34,useNA = "always")
A$NMI_Age34[is.na(A$NMI_Age34) &A$AGE95==34]<-A$I95[is.na(A$NMI_Age34)
&A$AGE95==34]
table(A$NMI_Age34,useNA = "always")
A$NMI_Age34[is.na(A$NMI_Age34) &A$AGE96==34]<-A$I96[is.na(A$NMI_Age34)
&A$AGE96==34]
table(A$NMI_Age34,useNA = "always")
A$NMI_Age34[is.na(A$NMI_Age34) &A$AGE97==34]<-A$I97[is.na(A$NMI_Age34)
&A$AGE97==34]
table(A$NMI_Age34,useNA = "always")
A$NMI_Age34[is.na(A$NMI_Age34) &A$AGE98==34]<-A$I98[is.na(A$NMI_Age34)
&A$AGE98==34]
table(A$NMI_Age34,useNA = "always")
A$NMI_Age34[is.na(A$NMI_Age34) &A$AGE99==34]<-A$I99[is.na(A$NMI_Age34)
&A$AGE99==34]
table(A$NMI_Age34,useNA = "always")
A$NMI_Age34[is.na(A$NMI_Age34) & A$AGE00==34]<-A$I00[is.na(A$NMI_Age34)
&A$AGE00==34]
table(A$NMI_Age34,useNA = "always")
A$NMI_Age34[is.na(A$NMI_Age34) & A$AGE01==34]<-A$I01[is.na(A$NMI_Age34)
&A$AGE01==34]
table(A$NMI_Age34,useNA = "always")
A$NMI_Age34[is.na(A$NMI_Age34) & A$AGE02==34]<-A$I02[is.na(A$NMI_Age34)
&A$AGE02==34]
table(A$NMI_Age34,useNA = "always")
A$NMI_Age34[is.na(A$NMI_Age34) & A$AGE03==34]<-A$I03[is.na(A$NMI_Age34)
&A$AGE03==34]
```

© 2023 Silver IA et al. *JAMA Network Open.*

```
table(A$NMI_Age34,useNA = "always")
A$NMI_Age34[is.na(A$NMI_Age34) & A$AGE04==34]<-A$I04[is.na(A$NMI_Age34)
&A$AGE04==34]
table(A$NMI_Age34,useNA = "always")
A$NMI_Age34[is.na(A$NMI_Age34) & A$AGE05==34]<-A$I05[is.na(A$NMI_Age34)
&A$AGE05==34]
table(A$NMI_Age34,useNA = "always")
A$NMI_Age34[is.na(A$NMI_Age34) & A$AGE06==34]<-A$I06[is.na(A$NMI_Age34)
&A$AGE06==34]
table(A$NMI_Age34,useNA = "always")
A$NMI_Age34[is.na(A$NMI_Age34) & A$AGE07==34]<-A$I07[is.na(A$NMI_Age34)
&A$AGE07==34]
table(A$NMI_Age34,useNA = "always")
A$NMI_Age34[is.na(A$NMI_Age34) & A$AGE08==34]<-A$I08[is.na(A$NMI_Age34)
&A$AGE08==34]
table(A$NMI_Age34,useNA = "always")
A$NMI_Age34[is.na(A$NMI_Age34) & A$AGE09==34]<-A$I09[is.na(A$NMI_Age34)
&A$AGE09==34]
table(A$NMI_Age34,useNA = "always")
A$NMI_Age34[is.na(A$NMI_Age34) & A$AGE10==34]<-A$I10[is.na(A$NMI_Age34)
&A$AGE10==34]
table(A$NMI_Age34,useNA = "always")
A$NMI_Age34[is.na(A$NMI_Age34) & A$AGE11==34]<-A$I11[is.na(A$NMI_Age34)
&A$AGE11==34]
table(A$NMI_Age34,useNA = "always")
A$NMI_Age34[is.na(A$NMI_Age34) & A$AGE12==34]<-A$I12[is.na(A$NMI_Age34)
&A$AGE12==34]
table(A$NMI_Age34,useNA = "always")
A$NMI_Age34[is.na(A$NMI_Age34) & A$AGE13==34]<-A$I13[is.na(A$NMI_Age34)
&A$AGE13==34]
table(A$NMI_Age34,useNA = "always")
A$NMI_Age34[is.na(A$NMI_Age34) & A$AGE14==34]<-A$I14[is.na(A$NMI_Age34)
&A$AGE14==34]
table(A$NMI_Age34,useNA = "always")
A$NMI_Age34[is.na(A$NMI_Age34) & A$AGE15==34]<-A$I15[is.na(A$NMI_Age34)
&A$AGE15==34]
table(A$NMI_Age34,useNA = "always")
A$NMI_Age34[is.na(A$NMI_Age34) & A$AGE16==34]<-A$I16[is.na(A$NMI_Age34)
&A$AGE16==34]
table(A$NMI_Age34,useNA = "always")
A$NMI_Age34[is.na(A$NMI_Age34) & A$AGE17==34]<-A$I17[is.na(A$NMI_Age34)
&A$AGE17==34]
table(A$NMI_Age34,useNA = "always")
A$NMI_Age34[A$Dec_Age34==1]<-NA
table(A$NMI_Age34,useNA = "always")
## NMI_Age35
table(A$AGE17)
A$NMI_Age35<-NA
A$NMI_Age35[is.na(A$NMI_Age35) & A$AGE92==35]<-A$I92[is.na(A$NMI_Age35) &
A$AGE92==35]
table(A$NMI_Age35,useNA = "always")
A$NMI_Age35[is.na(A$NMI_Age35) &A$AGE93==35]<-A$I93[is.na(A$NMI_Age35)
&A$AGE93==35]
table(A$NMI_Age35,useNA = "always")
A$NMI_Age35[is.na(A$NMI_Age35) &A$AGE94==35]<-A$I94[is.na(A$NMI_Age35)
&A$AGE94==35]
```

© 2023 Silver IA et al. *JAMA Network Open.*

```
table(A$NMI_Age35,useNA = "always")
A$NMI_Age35[is.na(A$NMI_Age35) &A$AGE95==35]<-A$I95[is.na(A$NMI_Age35)
&A$AGE95==35]
table(A$NMI_Age35,useNA = "always")
A$NMI_Age35[is.na(A$NMI_Age35) &A$AGE96==35]<-A$I96[is.na(A$NMI_Age35)
&A$AGE96==35]
table(A$NMI_Age35,useNA = "always")
A$NMI_Age35[is.na(A$NMI_Age35) &A$AGE97==35]<-A$I97[is.na(A$NMI_Age35)
&A$AGE97==35]
table(A$NMI_Age35,useNA = "always")
A$NMI_Age35[is.na(A$NMI_Age35) &A$AGE98==35]<-A$I98[is.na(A$NMI_Age35)
&A$AGE98==35]
table(A$NMI_Age35,useNA = "always")
A$NMI_Age35[is.na(A$NMI_Age35) &A$AGE99==35]<-A$I99[is.na(A$NMI_Age35)
&A$AGE99==35]
table(A$NMI_Age35,useNA = "always")
A$NMI_Age35[is.na(A$NMI_Age35) & A$AGE00==35]<-A$I00[is.na(A$NMI_Age35)
&A$AGE00==35]
table(A$NMI_Age35,useNA = "always")
A$NMI_Age35[is.na(A$NMI_Age35) & A$AGE01==35]<-A$I01[is.na(A$NMI_Age35)
&A$AGE01==35]
table(A$NMI_Age35,useNA = "always")
A$NMI_Age35[is.na(A$NMI_Age35) & A$AGE02==35]<-A$I02[is.na(A$NMI_Age35)
&A$AGE02==35]
table(A$NMI_Age35,useNA = "always")
A$NMI_Age35[is.na(A$NMI_Age35) & A$AGE03==35]<-A$I03[is.na(A$NMI_Age35)
&A$AGE03==35]
table(A$NMI_Age35,useNA = "always")
A$NMI_Age35[is.na(A$NMI_Age35) & A$AGE04==35]<-A$I04[is.na(A$NMI_Age35)
&A$AGE04==35]
table(A$NMI_Age35,useNA = "always")
A$NMI_Age35[is.na(A$NMI_Age35) & A$AGE05==35]<-A$I05[is.na(A$NMI_Age35)
&A$AGE05==35]
table(A$NMI_Age35,useNA = "always")
A$NMI_Age35[is.na(A$NMI_Age35) & A$AGE06==35]<-A$I06[is.na(A$NMI_Age35)
&A$AGE06==35]
table(A$NMI_Age35,useNA = "always")
A$NMI_Age35[is.na(A$NMI_Age35) & A$AGE07==35]<-A$I07[is.na(A$NMI_Age35)
&A$AGE07==35]
table(A$NMI_Age35,useNA = "always")
A$NMI_Age35[is.na(A$NMI_Age35) & A$AGE08==35]<-A$I08[is.na(A$NMI_Age35)
&A$AGE08==35]
table(A$NMI_Age35,useNA = "always")
A$NMI_Age35[is.na(A$NMI_Age35) & A$AGE09==35]<-A$I09[is.na(A$NMI_Age35)
&A$AGE09==35]
table(A$NMI_Age35,useNA = "always")
A$NMI_Age35[is.na(A$NMI_Age35) & A$AGE10==35]<-A$I10[is.na(A$NMI_Age35)
&A$AGE10==35]
table(A$NMI_Age35,useNA = "always")
A$NMI_Age35[is.na(A$NMI_Age35) & A$AGE11==35]<-A$I11[is.na(A$NMI_Age35)
&A$AGE11==35]
table(A$NMI_Age35,useNA = "always")
A$NMI_Age35[is.na(A$NMI_Age35) & A$AGE12==35]<-A$I12[is.na(A$NMI_Age35)
&A$AGE12==35]
table(A$NMI_Age35,useNA = "always")
```

© 2023 Silver IA et al. *JAMA Network Open.*

```
A$NMI_Age35[is.na(A$NMI_Age35) & A$AGE13==35]<-A$I13[is.na(A$NMI_Age35)
&A$AGE13==35]
table(A$NMI_Age35,useNA = "always")
A$NMI_Age35[is.na(A$NMI_Age35) & A$AGE14==35]<-A$I14[is.na(A$NMI_Age35)
&A$AGE14==35]
table(A$NMI_Age35,useNA = "always")
A$NMI_Age35[is.na(A$NMI_Age35) & A$AGE15==35]<-A$I15[is.na(A$NMI_Age35)
&A$AGE15==35]
table(A$NMI_Age35,useNA = "always")
A$NMI_Age35[is.na(A$NMI_Age35) & A$AGE16==35]<-A$I16[is.na(A$NMI_Age35)
&A$AGE16==35]
table(A$NMI_Age35,useNA = "always")
A$NMI_Age35[is.na(A$NMI_Age35) & A$AGE17==35]<-A$I17[is.na(A$NMI_Age35)
&A$AGE17==35]
table(A$NMI_Age35,useNA = "always")
A$NMI_Age35[A$Dec_Age35==1]<-NA
table(A$NMI_Age35,useNA = "always")
## NMI_Age36
table(A$AGE17)
A$NMI_Age36<-NA
A$NMI_Age36[is.na(A$NMI_Age36) & A$AGE92==36]<-A$I92[is.na(A$NMI_Age36) &
A$AGE92==36]
table(A$NMI_Age36,useNA = "always")
A$NMI_Age36[is.na(A$NMI_Age36) &A$AGE93==36]<-A$I93[is.na(A$NMI_Age36)
&A$AGE93==36]
table(A$NMI_Age36,useNA = "always")
A$NMI_Age36[is.na(A$NMI_Age36) &A$AGE94==36]<-A$I94[is.na(A$NMI_Age36)
&A$AGE94==36]
table(A$NMI_Age36,useNA = "always")
A$NMI_Age36[is.na(A$NMI_Age36) &A$AGE95==36]<-A$I95[is.na(A$NMI_Age36)
&A$AGE95==36]
table(A$NMI_Age36,useNA = "always")
A$NMI_Age36[is.na(A$NMI_Age36) &A$AGE96==36]<-A$I96[is.na(A$NMI_Age36)
&A$AGE96==36]
table(A$NMI_Age36,useNA = "always")
A$NMI_Age36[is.na(A$NMI_Age36) &A$AGE97==36]<-A$I97[is.na(A$NMI_Age36)
&A$AGE97==36]
table(A$NMI_Age36,useNA = "always")
A$NMI_Age36[is.na(A$NMI_Age36) &A$AGE98==36]<-A$I98[is.na(A$NMI_Age36)
&A$AGE98==36]
table(A$NMI_Age36,useNA = "always")
A$NMI_Age36[is.na(A$NMI_Age36) &A$AGE99==36]<-A$I99[is.na(A$NMI_Age36)
&A$AGE99==36]
table(A$NMI_Age36,useNA = "always")
A$NMI_Age36[is.na(A$NMI_Age36) & A$AGE00==36]<-A$I00[is.na(A$NMI_Age36)
&A$AGE00==36]
table(A$NMI_Age36,useNA = "always")
A$NMI_Age36[is.na(A$NMI_Age36) & A$AGE01==36]<-A$I01[is.na(A$NMI_Age36)
&A$AGE01==36]
table(A$NMI_Age36,useNA = "always")
A$NMI_Age36[is.na(A$NMI_Age36) & A$AGE02==36]<-A$I02[is.na(A$NMI_Age36)
&A$AGE02==36]
table(A$NMI_Age36,useNA = "always")
A$NMI_Age36[is.na(A$NMI_Age36) & A$AGE03==36]<-A$I03[is.na(A$NMI_Age36)
&A$AGE03==36]
table(A$NMI_Age36,useNA = "always")
```

© 2023 Silver IA et al. *JAMA Network Open.*

```
A$NMI_Age36[is.na(A$NMI_Age36) & A$AGE04==36]<-A$I04[is.na(A$NMI_Age36)
&A$AGE04==36]
table(A$NMI_Age36,useNA = "always")
A$NMI_Age36[is.na(A$NMI_Age36) & A$AGE05==36]<-A$I05[is.na(A$NMI_Age36)
&A$AGE05==36]
table(A$NMI_Age36,useNA = "always")
A$NMI_Age36[is.na(A$NMI_Age36) & A$AGE06==36]<-A$I06[is.na(A$NMI_Age36)
&A$AGE06==36]
table(A$NMI_Age36,useNA = "always")
A$NMI_Age36[is.na(A$NMI_Age36) & A$AGE07==36]<-A$I07[is.na(A$NMI_Age36)
&A$AGE07==36]
table(A$NMI_Age36,useNA = "always")
A$NMI_Age36[is.na(A$NMI_Age36) & A$AGE08==36]<-A$I08[is.na(A$NMI_Age36)
&A$AGE08==36]
table(A$NMI_Age36,useNA = "always")
A$NMI_Age36[is.na(A$NMI_Age36) & A$AGE09==36]<-A$I09[is.na(A$NMI_Age36)
&A$AGE09==36]
table(A$NMI_Age36,useNA = "always")
A$NMI_Age36[is.na(A$NMI_Age36) & A$AGE10==36]<-A$I10[is.na(A$NMI_Age36)
&A$AGE10==36]
table(A$NMI_Age36,useNA = "always")
A$NMI_Age36[is.na(A$NMI_Age36) & A$AGE11==36]<-A$I11[is.na(A$NMI_Age36)
&A$AGE11==36]
table(A$NMI_Age36,useNA = "always")
A$NMI_Age36[is.na(A$NMI_Age36) & A$AGE12==36]<-A$I12[is.na(A$NMI_Age36)
&A$AGE12==36]
table(A$NMI_Age36,useNA = "always")
A$NMI_Age36[is.na(A$NMI_Age36) & A$AGE13==36]<-A$I13[is.na(A$NMI_Age36)
&A$AGE13==36]
table(A$NMI_Age36,useNA = "always")
A$NMI_Age36[is.na(A$NMI_Age36) & A$AGE14==36]<-A$I14[is.na(A$NMI_Age36)
&A$AGE14==36]
table(A$NMI_Age36,useNA = "always")
A$NMI_Age36[is.na(A$NMI_Age36) & A$AGE15==36]<-A$I15[is.na(A$NMI_Age36)
&A$AGE15==36]
table(A$NMI_Age36,useNA = "always")
A$NMI_Age36[is.na(A$NMI_Age36) & A$AGE16==36]<-A$I16[is.na(A$NMI_Age36)
&A$AGE16==36]
table(A$NMI_Age36,useNA = "always")
A$NMI_Age36[is.na(A$NMI_Age36) & A$AGE17==36]<-A$I17[is.na(A$NMI_Age36)
&A$AGE17==36]
table(A$NMI_Age36,useNA = "always")
A$NMI_Age36[A$Dec_Age36==1]<-NA
table(A$NMI_Age36,useNA = "always")
## NMI_Age37
table(A$AGE17)
A$NMI_Age37<-NA
A$NMI_Age37[is.na(A$NMI_Age37) & A$AGE92==37]<-A$I92[is.na(A$NMI_Age37) &
A$AGE92==37]
table(A$NMI_Age37,useNA = "always")
A$NMI_Age37[is.na(A$NMI_Age37) &A$AGE93==37]<-A$I93[is.na(A$NMI_Age37)
&A$AGE93==37]
table(A$NMI_Age37,useNA = "always")
A$NMI_Age37[is.na(A$NMI_Age37) &A$AGE94==37]<-A$I94[is.na(A$NMI_Age37)
&A$AGE94==37]
table(A$NMI_Age37,useNA = "always")
```

© 2023 Silver IA et al. *JAMA Network Open.*

```
A$NMI_Age37[is.na(A$NMI_Age37) &A$AGE95==37]<-A$I95[is.na(A$NMI_Age37)
&A$AGE95==37]
table(A$NMI_Age37,useNA = "always")
A$NMI_Age37[is.na(A$NMI_Age37) &A$AGE96==37]<-A$I96[is.na(A$NMI_Age37)
&A$AGE96==37]
table(A$NMI_Age37,useNA = "always")
A$NMI_Age37[is.na(A$NMI_Age37) &A$AGE97==37]<-A$I97[is.na(A$NMI_Age37)
&A$AGE97==37]
table(A$NMI_Age37,useNA = "always")
A$NMI_Age37[is.na(A$NMI_Age37) &A$AGE98==37]<-A$I98[is.na(A$NMI_Age37)
&A$AGE98==37]
table(A$NMI_Age37,useNA = "always")
A$NMI_Age37[is.na(A$NMI_Age37) &A$AGE99==37]<-A$I99[is.na(A$NMI_Age37)
&A$AGE99==37]
table(A$NMI_Age37,useNA = "always")
A$NMI_Age37[is.na(A$NMI_Age37) & A$AGE00==37]<-A$I00[is.na(A$NMI_Age37)
&A$AGE00==37]
table(A$NMI_Age37,useNA = "always")
A$NMI_Age37[is.na(A$NMI_Age37) & A$AGE01==37]<-A$I01[is.na(A$NMI_Age37)
&A$AGE01==37]
table(A$NMI_Age37,useNA = "always")
A$NMI_Age37[is.na(A$NMI_Age37) & A$AGE02==37]<-A$I02[is.na(A$NMI_Age37)
&A$AGE02==37]
table(A$NMI_Age37,useNA = "always")
A$NMI_Age37[is.na(A$NMI_Age37) & A$AGE03==37]<-A$I03[is.na(A$NMI_Age37)
&A$AGE03==37]
table(A$NMI_Age37,useNA = "always")
A$NMI_Age37[is.na(A$NMI_Age37) & A$AGE04==37]<-A$I04[is.na(A$NMI_Age37)
&A$AGE04==37]
table(A$NMI_Age37,useNA = "always")
A$NMI_Age37[is.na(A$NMI_Age37) & A$AGE05==37]<-A$I05[is.na(A$NMI_Age37)
&A$AGE05==37]
table(A$NMI_Age37,useNA = "always")
A$NMI_Age37[is.na(A$NMI_Age37) & A$AGE06==37]<-A$I06[is.na(A$NMI_Age37)
&A$AGE06==37]
table(A$NMI_Age37,useNA = "always")
A$NMI_Age37[is.na(A$NMI_Age37) & A$AGE07==37]<-A$I07[is.na(A$NMI_Age37)
&A$AGE07==37]
table(A$NMI_Age37,useNA = "always")
A$NMI_Age37[is.na(A$NMI_Age37) & A$AGE08==37]<-A$I08[is.na(A$NMI_Age37)
&A$AGE08==37]
table(A$NMI_Age37,useNA = "always")
A$NMI_Age37[is.na(A$NMI_Age37) & A$AGE09==37]<-A$I09[is.na(A$NMI_Age37)
&A$AGE09==37]
table(A$NMI_Age37,useNA = "always")
A$NMI_Age37[is.na(A$NMI_Age37) & A$AGE10==37]<-A$I10[is.na(A$NMI_Age37)
&A$AGE10==37]
table(A$NMI_Age37,useNA = "always")
A$NMI_Age37[is.na(A$NMI_Age37) & A$AGE11==37]<-A$I11[is.na(A$NMI_Age37)
&A$AGE11==37]
table(A$NMI_Age37,useNA = "always")
A$NMI_Age37[is.na(A$NMI_Age37) & A$AGE12==37]<-A$I12[is.na(A$NMI_Age37)
&A$AGE12==37]
table(A$NMI_Age37,useNA = "always")
A$NMI_Age37[is.na(A$NMI_Age37) & A$AGE13==37]<-A$I13[is.na(A$NMI_Age37)
&A$AGE13==37]
```

© 2023 Silver IA et al. *JAMA Network Open.*

```
table(A$NMI_Age37,useNA = "always")
A$NMI_Age37[is.na(A$NMI_Age37) & A$AGE14==37]<-A$I14[is.na(A$NMI_Age37)
&A$AGE14==37]
table(A$NMI_Age37,useNA = "always")
A$NMI_Age37[is.na(A$NMI_Age37) & A$AGE15==37]<-A$I15[is.na(A$NMI_Age37)
&A$AGE15==37]
table(A$NMI_Age37,useNA = "always")
A$NMI_Age37[is.na(A$NMI_Age37) & A$AGE16==37]<-A$I16[is.na(A$NMI_Age37)
&A$AGE16==37]
table(A$NMI_Age37,useNA = "always")
A$NMI_Age37[is.na(A$NMI_Age37) & A$AGE17==37]<-A$I17[is.na(A$NMI_Age37)
&A$AGE17==37]
table(A$NMI_Age37,useNA = "always")
A$NMI_Age37[A$Dec_Age37==1]<-NA
table(A$NMI_Age37,useNA = "always")
## NMI_Age38
table(A$AGE17)
A$NMI_Age38<-NA
A$NMI_Age38[is.na(A$NMI_Age38) & A$AGE92==38]<-A$I92[is.na(A$NMI_Age38) &
A$AGE92==38]
table(A$NMI_Age38,useNA = "always")
A$NMI_Age38[is.na(A$NMI_Age38) &A$AGE93==38]<-A$I93[is.na(A$NMI_Age38)
&A$AGE93==38]
table(A$NMI_Age38,useNA = "always")
A$NMI_Age38[is.na(A$NMI_Age38) &A$AGE94==38]<-A$I94[is.na(A$NMI_Age38)
&A$AGE94==38]
table(A$NMI_Age38,useNA = "always")
A$NMI_Age38[is.na(A$NMI_Age38) &A$AGE95==38]<-A$I95[is.na(A$NMI_Age38)
&A$AGE95==38]
table(A$NMI_Age38,useNA = "always")
A$NMI_Age38[is.na(A$NMI_Age38) &A$AGE96==38]<-A$I96[is.na(A$NMI_Age38)
&A$AGE96==38]
table(A$NMI_Age38,useNA = "always")
A$NMI_Age38[is.na(A$NMI_Age38) &A$AGE97==38]<-A$I97[is.na(A$NMI_Age38)
&A$AGE97==38]
table(A$NMI_Age38,useNA = "always")
A$NMI_Age38[is.na(A$NMI_Age38) &A$AGE98==38]<-A$I98[is.na(A$NMI_Age38)
&A$AGE98==38]
table(A$NMI_Age38,useNA = "always")
A$NMI_Age38[is.na(A$NMI_Age38) &A$AGE99==38]<-A$I99[is.na(A$NMI_Age38)
&A$AGE99==38]
table(A$NMI_Age38,useNA = "always")
A$NMI_Age38[is.na(A$NMI_Age38) & A$AGE00==38]<-A$I00[is.na(A$NMI_Age38)
&A$AGE00==38]
table(A$NMI_Age38,useNA = "always")
A$NMI_Age38[is.na(A$NMI_Age38) & A$AGE01==38]<-A$I01[is.na(A$NMI_Age38)
&A$AGE01==38]
table(A$NMI_Age38,useNA = "always")
A$NMI_Age38[is.na(A$NMI_Age38) & A$AGE02==38]<-A$I02[is.na(A$NMI_Age38)
&A$AGE02==38]
table(A$NMI_Age38,useNA = "always")
A$NMI_Age38[is.na(A$NMI_Age38) & A$AGE03==38]<-A$I03[is.na(A$NMI_Age38)
&A$AGE03==38]
table(A$NMI_Age38,useNA = "always")
A$NMI_Age38[is.na(A$NMI_Age38) & A$AGE04==38]<-A$I04[is.na(A$NMI_Age38)
&A$AGE04==38]
```

© 2023 Silver IA et al. *JAMA Network Open.*

```
table(A$NMI_Age38,useNA = "always")
A$NMI_Age38[is.na(A$NMI_Age38) & A$AGE05==38]<-A$I05[is.na(A$NMI_Age38)
&A$AGE05==38]
table(A$NMI_Age38,useNA = "always")
A$NMI_Age38[is.na(A$NMI_Age38) & A$AGE06==38]<-A$I06[is.na(A$NMI_Age38)
&A$AGE06==38]
table(A$NMI_Age38,useNA = "always")
A$NMI_Age38[is.na(A$NMI_Age38) & A$AGE07==38]<-A$I07[is.na(A$NMI_Age38)
&A$AGE07==38]
table(A$NMI_Age38,useNA = "always")
A$NMI_Age38[is.na(A$NMI_Age38) & A$AGE08==38]<-A$I08[is.na(A$NMI_Age38)
&A$AGE08==38]
table(A$NMI_Age38,useNA = "always")
A$NMI_Age38[is.na(A$NMI_Age38) & A$AGE09==38]<-A$I09[is.na(A$NMI_Age38)
&A$AGE09==38]
table(A$NMI_Age38,useNA = "always")
A$NMI_Age38[is.na(A$NMI_Age38) & A$AGE10==38]<-A$I10[is.na(A$NMI_Age38)
&A$AGE10==38]
table(A$NMI_Age38,useNA = "always")
A$NMI_Age38[is.na(A$NMI_Age38) & A$AGE11==38]<-A$I11[is.na(A$NMI_Age38)
&A$AGE11==38]
table(A$NMI_Age38,useNA = "always")
A$NMI_Age38[is.na(A$NMI_Age38) & A$AGE12==38]<-A$I12[is.na(A$NMI_Age38)
&A$AGE12==38]
table(A$NMI_Age38,useNA = "always")
A$NMI_Age38[is.na(A$NMI_Age38) & A$AGE13==38]<-A$I13[is.na(A$NMI_Age38)
&A$AGE13==38]
table(A$NMI_Age38,useNA = "always")
A$NMI_Age38[is.na(A$NMI_Age38) & A$AGE14==38]<-A$I14[is.na(A$NMI_Age38)
&A$AGE14==38]
table(A$NMI_Age38,useNA = "always")
A$NMI_Age38[is.na(A$NMI_Age38) & A$AGE15==38]<-A$I15[is.na(A$NMI_Age38)
&A$AGE15==38]
table(A$NMI_Age38,useNA = "always")
A$NMI_Age38[is.na(A$NMI_Age38) & A$AGE16==38]<-A$I16[is.na(A$NMI_Age38)
&A$AGE16==38]
table(A$NMI_Age38,useNA = "always")
A$NMI_Age38[is.na(A$NMI_Age38) & A$AGE17==38]<-A$I17[is.na(A$NMI_Age38)
&A$AGE17==38]
table(A$NMI_Age38,useNA = "always")
A$NMI_Age38[A$Dec_Age38==1]<-NA
table(A$NMI_Age38,useNA = "always")

# Other Measures

# General Health
table(A$AGE97)
## General Health (1997)
table(A$R0320600)
A$GH97<-A$R0320600
A$GH97[A$GH97<0]<-NA
table(A$GH97)
A$RGH97<-5-A$GH97
table(A$GH97)
table(A$RGH97)
```

© 2023 Silver IA et al. *JAMA Network Open.*

```
## Parental Incarceration
#### Measures
("T8218800","T8219200","U0113400","U0113800","U1966600","U1967000")

table(A$T8218800)
table(A$T8219200)
table(A$U0113400)
table(A$U0113800)
table(A$U1966600)
table(A$U1967000)

### Parent Incarceration before 16
A$PIB16<-NA
A$PIB16[A$T8218800 == 1|A$T8219200 == 1|A$U0113400 == 1|A$U0113800 ==
1|A$U1966600 == 1|A$U1967000 ==1]<-1
table(A$PIB16, useNA = "always")
A$PIB16[is.na(A$PIB16) & A$T8218800 == 0]<-0
table(A$PIB16, useNA = "always")
A$PIB16[is.na(A$PIB16) & A$T8219200 == 0]<-0
table(A$PIB16, useNA = "always")
A$PIB16[is.na(A$PIB16) & A$U0113400 == 0]<-0
table(A$PIB16, useNA = "always")
A$PIB16[is.na(A$PIB16) & A$U0113800 == 0]<-0
table(A$PIB16, useNA = "always")
A$PIB16[is.na(A$PIB16) & A$U1966600 == 0]<-0
table(A$PIB16, useNA = "always")
A$PIB16[is.na(A$PIB16) & A$U1967000 == 0]<-0
table(A$PIB16, useNA = "always")


### Paternal Incarceration before 16 (contains valid skips if R knew father)
A$PATIB16<-NA
A$PATIB16[A$T8219200 == 1|A$U0113800 == 1|A$U1967000 ==1]<-1
table(A$PATIB16, useNA = "always")
A$PATIB16[is.na(A$PATIB16) & A$T8219200 == 0]<-0
table(A$PATIB16, useNA = "always")
A$PATIB16[is.na(A$PATIB16) & A$U0113800 == 0]<-0
table(A$PATIB16, useNA = "always")
A$PATIB16[is.na(A$PATIB16) & A$U1967000 == 0]<-0
table(A$PATIB16, useNA = "always")
A$PATIB16[is.na(A$PATIB16) & A$PIB16 == 0]<-0
table(A$PATIB16, useNA = "always")

table(A$PIB16, A$PATIB16, useNA = "always")

### Maternal Incarceration before 16
A$MATIB16<-NA
A$MATIB16[A$T8218800 == 1|A$U0113400 == 1|A$U1966600 == 1]<-1
table(A$MATIB16, useNA = "always")
A$MATIB16[is.na(A$MATIB16) & A$T8218800 == 0]<-0
table(A$MATIB16, useNA = "always")
A$MATIB16[is.na(A$MATIB16) & A$U0113400 == 0]<-0
table(A$MATIB16, useNA = "always")
A$MATIB16[is.na(A$MATIB16) & A$U1966600 == 0]<-0
table(A$MATIB16, useNA = "always")
A$MATIB16[is.na(A$MATIB16) & A$PIB16 == 0]<-0
```

© 2023 Silver IA et al. *JAMA Network Open.*

```
table(A$MATIB16, useNA = "always")

table(A$PIB16, A$MATIB16, useNA = "always")

## Ethnicity (Hispanic)
table(A$R0538600)

A$Hispanic<-A$R0538600
A$Hispanic[A$Hispanic<0]<-NA
table(A$Hispanic, useNA="always")
## Race
table(A$R0538700)
A$R0538700[A$R0538700==-1|A$R0538700==-2]<-NA
table(A$R0538700)
A$White[A$R0538700==1]<-1
A$White[A$R0538700>1]<-0
table(A$White)
A$Black[A$R0538700==2]<-1
A$Black[A$R0538700==1|A$R0538700>2]<-0
table(A$Black)
A$AIndian[A$R0538700==3]<-1
A$AIndian[A$R0538700<3|A$R0538700>3]<-0
table(A$AIndian)
A$Asian[A$R0538700==4]<-1
A$Asian[A$R0538700<4|A$R0538700==5]<-0
table(A$Asian)
A$Other[A$R0538700==5]<-1
A$Other[A$R0538700<5]<-0
table(A$Other)
table(A$NonW, useNA="always")

## Sex
table(A$R0536300)
A$Male<-A$R0536300
A$Male[A$Male==2]<-0
table(A$Male, useNA="always")
## Sample
table(A$R1235800)
A$Sample<-A$R1235800
A$Sample[A$Sample==0]<-2
table(A$Sample)

## Both Bio Parents in Household (1997)
table(A$R0532200)

A$BBPHH<-A$R0532200
A$BBPHH[A$BBPHH == -4]<-NA
table(A$BBPHH, useNA="always")

## Child Care (+20 hours first year; 1997)
table(A$R0649100)

A$CC1Y<-A$R0649100
A$CC1Y[A$CC1Y == -4|A$CC1Y == -2| A$CC1Y == -1]<-NA
table(A$CC1Y, useNA="always")
```

© 2023 Silver IA et al. *JAMA Network Open.*

```
## Hard Times (1997)
table(A$R0648900)

A$HT97<-A$R0648900
A$HT97[A$HT97 == -4|A$HT97 == -2| A$HT97 == -1]<-NA
table(A$HT97, useNA="always")



### Household Income 1997 (from Respondent or Parent Survey)
#### Measures ("R1204500","R1204600","R1204700","R1204800")

table(A$R1204500)
table(A$R1204600)
table(A$R1204700)
table(A$R1204800)

A$HHNW97<-A$R1204700
A$HHNW97[A$R1204700 == -5|A$R1204700 == -4| A$R1204700 == -3]<-NA
table(A$R1204700, useNA = "always")
table(A$HHNW97, useNA = "always")

A$R1204800[A$R1204800 == -5|A$R1204800 == -4| A$R1204800 == -3]<-NA
table(A$R1204800, useNA = "always")


A$HHNW97[is.na(A$HHNW97)]<-A$R1204800[A$R1204800>=0 | A$R1204800<=0]
table(A$HHNW97, useNA = "always")

# Clean Data

fwrite(A,"Data/Clean Data.csv", buffMB = 1024L, append = T, sep = ",", quote
= F, compress = "none")







# Limiting Data ####
names(A)

B<-(A[, c("R0000100",
"AGE92","AGE93","AGE94","AGE95","AGE96","AGE97","AGE98","AGE99",

"AGE00","AGE01","AGE02","AGE03","AGE04","AGE05","AGE06","AGE07","AGE08","AGE0
9",

"AGE10","AGE11","AGE12","AGE13","AGE14","AGE15","AGE16","AGE17","AGE18","AGE1
9",
```

© 2023 Silver IA et al. *JAMA Network Open.*

```
"Dec_Age9","Dec_Age10","Dec_Age11","Dec_Age12","Dec_Age13","Dec_Age14","Dec_A
ge15","Dec_Age16","Dec_Age17","Dec_Age18",

"Dec_Age19","Dec_Age20","Dec_Age21","Dec_Age22","Dec_Age23","Dec_Age24","Dec_
Age25","Dec_Age26","Dec_Age27","Dec_Age28",

"Dec_Age29","Dec_Age30","Dec_Age31","Dec_Age32","Dec_Age33","Dec_Age34","Dec_
Age35","Dec_Age36","Dec_Age37","Dec_Age38",
          "Dec_Age39","Age_DEC","DEC_CEN","NA_Age7",

"NA_Age8","NA_Age9","NA_Age10","NA_Age11","NA_Age12","NA_Age13","NA_Age14","N
A_Age15","NA_Age16","NA_Age17",

"NA_Age18","NA_Age19","NA_Age20","NA_Age21","NA_Age22","NA_Age23","NA_Age24",
"NA_Age25","NA_Age26","NA_Age27",

"NA_Age28","NA_Age29","NA_Age30","NA_Age31","NA_Age32","NA_Age33","NA_Age34",
"NA_Age35","NA_Age36","NA_Age37",

"NA_Age38","NMI_Age7","NMI_Age8","NMI_Age9","NMI_Age10","NMI_Age11","NMI_Age1
2","NMI_Age13","NMI_Age14","NMI_Age15",

"NMI_Age16","NMI_Age17","NMI_Age18","NMI_Age19","NMI_Age20","NMI_Age21","NMI_
Age22","NMI_Age23","NMI_Age24","NMI_Age25",

"NMI_Age26","NMI_Age27","NMI_Age28","NMI_Age29","NMI_Age30","NMI_Age31","NMI_
Age32","NMI_Age33","NMI_Age34","NMI_Age35",

"NMI_Age36","NMI_Age37","NMI_Age38","GH97","RGH97","PIB16","PATIB16",
"MATIB16","Hispanic","White",

"Black","AIndian","Asian","Other","Male","Sample","BBPHH","CC1Y","HT97","HHNW
97")])


# Calculating Sums ####
## Number of Arrests Before 18

B$NA_B18<-rowSums(data.frame(B$NA_Age7,B$NA_Age8,B$NA_Age9,
                             B$NA_Age10,B$NA_Age11,B$NA_Age12,
                             B$NA_Age13,B$NA_Age14,B$NA_Age15,
                             B$NA_Age16,B$NA_Age17), na.rm = T)

## Number of Arrests After 18
B$NA_A18<-rowSums(data.frame(B$NA_Age18,B$NA_Age19,B$NA_Age20,
                             B$NA_Age21,B$NA_Age22,B$NA_Age23,
                             B$NA_Age24,B$NA_Age25,B$NA_Age26,
                             B$NA_Age27,B$NA_Age28,B$NA_Age29,
                             B$NA_Age30,
                             B$NA_Age31,B$NA_Age32,B$NA_Age33,
                             B$NA_Age34,B$NA_Age35,B$NA_Age36,
                             B$NA_Age37,B$NA_Age38), na.rm = T)

## Number of Months Incarcerated Before 18
B$NMI_B18<-rowSums(data.frame(B$NMI_Age7,B$NMI_Age8,B$NMI_Age9,
```

© 2023 Silver IA et al. *JAMA Network Open.*

```
                              B$NMI_Age10,B$NMI_Age11,B$NMI_Age12,
                              B$NMI_Age13,B$NMI_Age14,B$NMI_Age15,
                              B$NMI_Age16,B$NMI_Age17), na.rm = T)

## Number of Months Incarcerated After 18
B$NMI_A18<-rowSums(data.frame(B$NMI_Age18,B$NMI_Age19,B$NMI_Age20,
                              B$NMI_Age21,B$NMI_Age22,B$NMI_Age23,
                              B$NMI_Age24,B$NMI_Age25,B$NMI_Age26,
                              B$NMI_Age27,B$NMI_Age28,B$NMI_Age29,
                              B$NMI_Age30,
                              B$NMI_Age31,B$NMI_Age32,B$NMI_Age33,
                              B$NMI_Age34,B$NMI_Age35,B$NMI_Age36,
                              B$NMI_Age37,B$NMI_Age38), na.rm = T)


# Limiting Sample to Age in 1997 is less than 18 ####
## Maintaining Temporal Order for Covariates
## 16 cases removed
table(B$AGE97)
C<-B[which(B$AGE97<18),]

# Limiting Sample to those deceased after the age of 18 ####
table(C$Age_DEC)
D<-C[which(C$Age_DEC>=18),]


# Limiting Data Frame to Key Variables Only ####

E<-(D[, c("R0000100","Age_DEC","DEC_CEN","RGH97","PIB16","Hispanic","White",

"Black","AIndian","Asian","Other","Male","Sample","BBPHH","CC1Y","HT97",
          "HHNW97","NA_B18","NA_A18","NMI_B18","NMI_A18")])

# Descriptive Statistics Before Imputation ####

sink("Results/Descriptive Statistics Before Imputation.txt")
stat.desc(E)
sink()

# Missing Case Analysis ####

names(E)
stat.desc(E)

Z<-E[complete.cases(E[,c(1:21)]),]
names(Z)

Z.1<-Z[, c(1)]

Z.1$CCDI<-1
names(Z.1)

Z.1<-Z.1[, c("R0000100","CCDI")]

MCDF<-merge(E, Z.1, by = c("R0000100"), all.x = T)
```

© 2023 Silver IA et al. *JAMA Network Open.*

```
MCDF$CCDI<-as.numeric(MCDF$CCDI)

table(MCDF$CCDI, useNA="always")

MCDF$CCDI[is.na(MCDF$CCDI)]<-0

table(MCDF$CCDI, useNA="always")

nlevels(MCDF$CCDI)

library(finalfit)

jpeg("Results/Missing Values Plot.jpeg", width = 500, height = 500, quality =
100)
missing_plot(MCDF)
dev.off()

jpeg("Results/Missing Pattern Plot.jpeg", width = 1000, height = 1000,
quality = 100)
missing_pattern(MCDF)
dev.off()

sink("Results/Missing Pattern Results.txt")
missing_pattern(MCDF)
sink()


names(MCDF)

sink("Results/Missing Case Analysis (t_test).txt")
stat.desc(MCDF[which(MCDF$CCDI == 1),])
stat.desc(MCDF[which(MCDF$CCDI == 0),])
print("# t-test")
print("# Complete Cases (0 = incomeplete cases; 1 = complete cases)")
table(MCDF$CCDI)
sapply(MCDF[, c(4:17, 18, 20)], function(x, na.rm = FALSE)
t.test(x~MCDF$CCDI))
sink()


sink("Results/Missing Case Analysis (Cohen's d).txt")
print("# Year_1")
print("# Complete Cases (0 = incomeplete cases; 1 = complete cases)")
table(MCDF$CCDI)
sapply(MCDF[, c(4:17, 18, 20)], function(x, na.rm = FALSE)
cohen.d(x~MCDF$CCDI))
sink()

cohen.d(MCDF$RGH97,MCDF$CCDI,na.rm = T)


names(MCDF)
MCDF$Arrested<-NA
MCDF$Arrested[MCDF$NA_B18==0 & MCDF$NMI_B18==0]<-0
MCDF$Arrested[MCDF$NA_B18>0 & MCDF$NMI_B18==0]<-1
table(MCDF$Arrested, useNA = "always")
```

© 2023 Silver IA et al. *JAMA Network Open.*

```
MCDF$Incarcerated<-NA
MCDF$Incarcerated[MCDF$NA_B18==0 & MCDF$NMI_B18==0]<-0
MCDF$Incarcerated[MCDF$NMI_B18>0]<-1
table(MCDF$Incarcerated, useNA = "always")

table(MCDF$CCDI)

table(MCDF$Arrested,MCDF$CCDI)

names(MCDF)

t.test(MCDF$DEC_CEN~MCDF$CCDI, na.rm = T)
cohen.d(MCDF$DEC_CEN~MCDF$CCDI,na.rm = T)


t.test(MCDF$Arrested~MCDF$CCDI, na.rm = T)
cohen.d(MCDF$Arrested~MCDF$CCDI,na.rm = T)

t.test(MCDF$Incarcerated~MCDF$CCDI, na.rm = T)
cohen.d(MCDF$Incarcerated~MCDF$CCDI,na.rm = T)

# Imputing Data ####
names(E)
stat.desc(E)


IM_E<-mice(E, m=5, maxit = 10, method = c("rf","rf","rf","rf",

"logreg","logreg","logreg","logreg","logreg",

"logreg","logreg","logreg","logreg","logreg",
                                          "logreg","logreg","rf","rf","rf",
                                          "rf","rf"), seed = 1992)


IM_F<-complete(IM_E)

fwrite(IM_F,"Data/Imputed Analytical Data.csv", buffMB = 1024L, append = T,
sep = ",", quote = F, compress = "none")


# Loading Imputed Data (USE ME) ####
G<-fread("Data/Imputed Analytical Data.csv", sep = ",", fill=TRUE, quote=F,
na.strings="NA")
names(G)
G<-data.frame(G)
stat.desc(G)

G<-G[complete.cases(G[,c(1:21)]),]



# Creating Criminal Group ####
names(G)
G$Criminal_Group<-NA
```

© 2023 Silver IA et al. *JAMA Network Open.*

```
G$Criminal_Group[G$NA_B18==0 & G$NMI_B18==0]<-"No CLS contact"
G$Criminal_Group[G$NA_B18>0 & G$NMI_B18==0]<-"Arrested < 18"
G$Criminal_Group[G$NMI_B18>0]<-"Incarcerated < 18"
table(G$Criminal_Group, useNA = "always")


G$Criminal_Group<-factor(G$Criminal_Group,levels=c("No CLS contact",
                                                    "Incarcerated < 18",
                                                    "Arrested < 18"))
SD<-G
SD$ARRA18<-0
SD$ARRA18[SD$Criminal_Group == "Arrested < 18"]<-1

SD$InN18<-0
SD$InN18[SD$Criminal_Group == "Incarcerated < 18"]<-1

table(SD$Hispanic)

SD$Non_His<-0
SD$InN18[SD$Hispanic == 0]<-1

stat.desc(SD$ARRA18)
stat.desc(SD$InN18)


# Descriptive Statistics After Imputation ####

sink("Results/Descriptive Statistics After Imputation.txt")
stat.desc(G)
sink()

stat.desc(G$Age_DEC[G$DEC_CEN==1])
table(G$Age_DEC[G$DEC_CEN==1])
# Determining the Appropriate Survival Model ####
## Loading Stephen's Function ####
survDist <- function(time, cen, data, type='right')
{
  require(survival)
  time <- data[[time]]
  cen  <- data[[cen]]
  fit.lognormal <- survreg( Surv(time=time, event=cen, type=type) ~ 1,
                            dist="lognormal")
  fit.exponential <- survreg( Surv(time=time, event=cen, type=type) ~ 1,
                              dist="exponential")
  fit.weibull <- survreg( Surv(time=time, event=cen, type=type) ~ 1,
                          dist="weibull")
  fit.gaussian <- survreg( Surv(time=time, event=cen, type=type) ~ 1,
                           dist="gaussian")
  fit.logistic <- survreg( Surv(time=time, event=cen, type=type) ~ 1,
                           dist="logistic")
  fit.loglogistic <- survreg( Surv(time=time, event=cen, type=type) ~ 1,
                              dist="loglogistic")
  aics <- c(
    extractAIC(fit.lognormal   )[2],
    extractAIC(fit.exponential )[2]         ,
    extractAIC(fit.weibull     )[2]          ,
```

© 2023 Silver IA et al. *JAMA Network Open.*

```
    extractAIC(fit.gaussian    )[2]          ,
    extractAIC(fit.logistic    )[2]          ,
    extractAIC(fit.loglogistic )[2] )
  distnms <- c('lognormal', 'exponential', 'weibull', 'gaussian', 'logistic',
'loglogistic')
  aics <- data.frame(distnms, aics)
  names(aics) <- c('Distribution', 'AIC')
  #
  w <- aics$AIC==min(aics$AIC)
  cat( paste('Best Model (AIC):\n', aics[w,1]), "\n\n")
  #
  return(aics)
}

## Running Data Through survDist ####
names(G)
survDist("Age_DEC", "DEC_CEN", G)


# Estimating Baseline Model ####

table(G$Age_DEC,G$DEC_CEN)

names(G)



Fit1<-survfit(Surv(Age_DEC, DEC_CEN)~1,data=G)

sink("Results/survival function.txt")
print(Fit1)
summary(Fit1)
sink()


Plot<-survfit(Surv(Age_DEC, DEC_CEN) ~ 1, data=G)

summary(Plot)

jpeg("results/Baseline Survival Plot.jpeg", width = 1500, height = 1250,
quality = 100)
ggsurvplot(Plot, data = G,size = 1,conf.int = TRUE,
           palette = c("black"),
           ylab = "Survival Probability",
           xlab = "Number of Years",
           surv.median.line = "hv",
           conf.int.style = "step",
           legend = "none",
           ylim=c(.90,1.00),
           xlim=c(18,40),
           ggtheme = (theme(legend.position= "none",
                            line = element_line(colour = "black", size = 1),
                            axis.line = element_line(colour = "black"),
                            panel.border= element_blank(),
                            panel.grid.major.x = element_line(colour =
"lightgray"),
```

© 2023 Silver IA et al. *JAMA Network Open.*

```
                            panel.grid.minor.x = element_line(colour =
"lightgray"),
                            panel.grid.major.y = element_line(colour =
"lightgray"),
                            panel.background = element_blank(),
                            title = element_text(family="Times New Roman",
size=42),
                            axis.title.x = element_text(family="Times New
Roman",colour = "Black", margin = margin(t = 20, r = 0, b = 0, l = 0),
size=42),
                            axis.title.y = element_text(family="Times New
Roman",colour = "Black", margin = margin(t = 0, r = 20, b = 0, l = 0),
size=42),
                            axis.text= element_text(family="Times New Roman",
size=42, color = "Black"),
                            text= element_text(family="Times New Roman",
size=42),
                            plot.margin = margin(t = 2, r = 2, b = 2, l = 2,
unit = "cm")))

)
dev.off()



# Primary Model ####

M2<-survreg(Surv(Age_DEC,
DEC_CEN)~as.factor(Criminal_Group)+NA_B18+NMI_B18+RGH97+PIB16+HT97+
              HHNW97+Male+Hispanic+White+Black+AIndian+Asian+Sample, robust =
T, data=G, dist= "lognormal")

sink("Results/Direct Effects of juvenile incarceration and Arrest on
Death.txt")
summary(M2)
confint(M2)
exp(M2$coefficients)
exp(confint(M2))
sink()

#### Predicted Survival ####

DF<-cbind(G,data.frame(predictSurvProb2survreg(M2,newdata = G,time_days =
c(18:39))))

names(DF)


Mean<-DF %>%
  group_by(as.factor(Criminal_Group)) %>%
  summarise(across(23:44, mean))

Mean<-data.frame(Mean)
names(Mean)
```

```
LMean<-reshape(Mean, varying =c(2:23), v.names = "XMean", timevar =
"Time_Years", direction = "long")

LMean$XMean_RR<-1-LMean$XMean

table(LMean$as.factor.Criminal_Group.)

LMean$Age<-rep(c(18:39), each = 3)


LMean$as.factor.Criminal_Group.<-factor(LMean$as.factor.Criminal_Group.,
                                        levels=c("Incarcerated < 18",
                                                 "Arrested < 18",
                                                 "No CLS contact"))



SD<-DF %>%
  group_by(as.factor(Criminal_Group)) %>%
  summarise(across(23:44, sd))

SD<-data.frame(SD)
names(SD)

LSD<-reshape(SD, varying =c(2:23), v.names = "XSD", timevar = "Time_Years",
direction = "long")


table(LSD$as.factor.Criminal_Group.)

LSD$Age<-rep(c(18:39), each = 3)


LSD$as.factor.Criminal_Group.<-factor(LSD$as.factor.Criminal_Group.,
                                      levels=c("Incarcerated < 18",
                                               "Arrested < 18",
                                               "No CLS contact"))

LMean$Use<-LMean$XMean_RR+LSD$XSD
LMean$Lse<-LMean$XMean_RR-LSD$XSD

win.metafile("Results/Probability of Death.wmf", width = 100, height = 50)
ggplot(LMean, aes(x = Age, y = XMean_RR, group= as.factor.Criminal_Group.,
fill=as.factor.Criminal_Group.))+
  geom_line(aes(y = XMean_RR, color = as.factor.Criminal_Group.),linewidth =
11)+
  geom_linerange(aes(x = Age, ymin = Lse,ymax = Use, color =
as.factor.Criminal_Group., group = as.factor.Criminal_Group.),position =
position_dodge(width = 0.5),linewidth = 11)+
  scale_x_continuous(breaks = c(18,21,24,27,30,33,36,39), limits = c(-
100,100))+
  scale_y_continuous(expand = c(0, 0), breaks =
c(0,.01,.02,.03,.04,.05,.06,.07,.08,.09,.10,.11,.12), limits = c(-1,1))+
  coord_cartesian(xlim = c(18,39), ylim = c(0,.12))+
  xlab("Age in Years")+
scale_color_manual(values=c("red","slategray","black"))+
  scale_fill_manual(values=c("red","slategray","black"))+
```

© 2023 Silver IA et al. *JAMA Network Open.*

```
  labs(y = paste0("Cumulative Probability", "\n", "of Death"))+
  guides(color = guide_legend(byrow = TRUE))+
  theme(legend.position= "right",
        legend.justification = 'center',
        legend.title=element_blank(),
        legend.background=element_blank(),
        legend.spacing.x = unit(2.0, 'cm'),
        legend.key.size = unit(4.0, "cm"),
        legend.key = element_rect(color="white"),
        legend.box="vertical",
        line = element_line(colour = "black", size = 1),
        axis.line = element_line(colour = "black"),
        panel.border= element_blank(),
        panel.grid.major = element_line(colour = "lightgray"),
        panel.grid.minor = element_line(colour = "lightgray"),
        panel.background = element_blank(),
        title = element_text(family="Times New Roman", size=160),
        axis.title.x = element_text(family="Times New Roman",colour =
"Black", margin = margin(t = 20, r = 0, b = 0, l = 0), size=160),
        axis.title.y = element_text(family="Times New Roman",colour =
"Black", margin = margin(t = 0, r = 20, b = 0, l = 0), size=160),
        axis.text.y= element_text(family="Times New Roman", hjust=10,
size=160, color = "Black"),
        axis.text.x= element_text(family="Times New Roman", vjust=-.50,
size=160, color = "Black"),
        text= element_text(family="Times New Roman", size=160),
        plot.margin = margin(t = 2, r = 2, b = 2, l = 2, unit = "cm"))
dev.off()


jpeg("Results/Probability of Death.jpeg", width = 8000, height = 5000,
quality = 100)
ggplot(LMean, aes(x = Age, y = XMean_RR, group= as.factor.Criminal_Group.,
fill=as.factor.Criminal_Group.))+
  geom_line(aes(y = XMean_RR, color = as.factor.Criminal_Group.),linewidth =
8)+
  geom_linerange(aes(x = Age, ymin = Lse,ymax = Use, color =
as.factor.Criminal_Group., group = as.factor.Criminal_Group.),position =
position_dodge(width = 0.5),linewidth = 8)+
  scale_x_continuous(breaks = c(18,21,24,27,30,33,36,39), limits = c(-
100,100))+
  scale_y_continuous(expand = c(0, 0), breaks =
c(0,.01,.02,.03,.04,.05,.06,.07,.08,.09,.10,.11,.12), limits = c(-1,1))+
  coord_cartesian(xlim = c(18,39), ylim = c(0,.12))+
  xlab("Age in Years")+
scale_color_manual(values=c("red","slategray","black"))+
  scale_fill_manual(values=c("red","slategray","black"))+
  labs(y = paste0("Cumulative Probability", "\n", "of Death"))+
  guides(color = guide_legend(byrow = TRUE))+
  theme(legend.position= "right",
        legend.justification = 'center',
        legend.title=element_blank(),
        legend.background=element_blank(),
        legend.spacing.x = unit(2.0, 'cm'),
        legend.key.size = unit(4.0, "cm"),
        legend.key = element_rect(color="white"),
```

© 2023 Silver IA et al. *JAMA Network Open.*

```
        legend.box="vertical",
        line = element_line(colour = "black", size = 1),
        axis.line = element_line(colour = "black"),
        panel.border= element_blank(),
        panel.grid.major = element_line(colour = "lightgray"),
        panel.grid.minor = element_line(colour = "lightgray"),
        panel.background = element_blank(),
        title = element_text(family="Times New Roman", size=160),
        axis.title.x = element_text(family="Times New Roman",colour =
"Black", margin = margin(t = 30, r = 0, b = 0, l = 0), size=160),
        axis.title.y = element_text(family="Times New Roman",colour =
"Black", margin = margin(t = 0, r = 30, b = 0, l = 0), size=160),
        axis.text.y= element_text(family="Times New Roman", hjust=10,
size=160, color = "Black"),
        axis.text.x= element_text(family="Times New Roman", vjust=-.50,
size=160, color = "Black"),
        text= element_text(family="Times New Roman", size=160),
        plot.margin = margin(t = 3, r = 3, b = 3, l = 3, unit = "cm"))
dev.off()



# Model With Number of Arrests and Months Incarcerated after 18 ####

names(G)

M3<-survreg(Surv(Age_DEC,
DEC_CEN)~as.factor(Criminal_Group)+NA_B18+NMI_B18+RGH97+PIB16+HT97+

HHNW97+Male+Hispanic+White+Black+AIndian+Asian+Sample+NA_A18+NMI_A18, robust
= T, data=G, dist= "lognormal")

sink("Results/Direct Effects of juvenile incarceration and Arrest on Death
(Number of arrests after 18 included).txt")
summary(M3)
confint(M3)
exp(M3$coefficients)
exp(confint(M3))
sink()




####________________________________________________________####
#### Revisions #####
####________________________________________________________####
# Juveniles Incarcerated in Juvenile Facilities ####
# Data Pull ####

memory.limit()
memory.limit(size=1000000)

A<-fread("G:/My Drive/Data and Statistics/Datasets/NLSY/NLSY_1997 (New; 2019-
2021 Included)/Raw Data Files/Full NLSY97 2.4.22.csv", sep = ",", fill=TRUE,
quote=F, na.strings="NA")
```

© 2023 Silver IA et al. *JAMA Network Open.*

```
A_S<-(A[,
c("R0000100","R0429300","R0429400","R0429500","R0429600","R0429700","R0429800
","R0429900",

"R0423000","R0423100","R0423200","R0423300","R0423400","R0423500",

"R2236100","R2236200","R2236300","R2236400","R2236500","R2236600",
            "R2236700","R2236800","R2236900",
            "R3540700","R3540800","R3540900","R3541000",
            "R4952500","R4952600","R4952700","R4952800","R4952900",
            "R4953000","R4953100","R4953100","R4953300",

"R6584300","R6584400","R6584500","R6584600","R6584700","R6584800","R6584900",
            "R6585000",

"S0970000","S0970100","S0970200","S0970300","S0970400","S0970500","S0970600",
            "S2999300")])
## Creating Juveniles Incarcerated in Juvenile Facilities Measure ####

table(A_S$R0429300)
table(A_S$S0970600)
A_S$JIJF<-0
A_S$JIJF[A_S$R0429300==3|A_S$R0429400==3|A_S$R0429500==3|A_S$R0429600==3|A_S$
R0429700==3|A_S$R0429800==3|A_S$R0429900==3|

A_S$R0423000==3|A_S$R0423100==3|A_S$R0423200==3|A_S$R0423300==3|A_S$R0423400=
=3|A_S$R0423500==3|

A_S$R2236100==3|A_S$R2236200==3|A_S$R2236300==3|A_S$R2236400==3|A_S$R2236500=
=3|A_S$R2236600==3|
           A_S$R2236700==3|A_S$R2236800==3|A_S$R2236900==3|
           A_S$R3540700==3|A_S$R3540800==3|A_S$R3540900==3|A_S$R3541000==3|

A_S$R4952500==3|A_S$R4952600==3|A_S$R4952700==3|A_S$R4952800==3|A_S$R4952900=
=3|
           A_S$R4953000==3|A_S$R4953100==3|A_S$R4953100==3|A_S$R4953300==3|

A_S$R6584300==3|A_S$R6584400==3|A_S$R6584500==3|A_S$R6584600==3|A_S$R6584700=
=3|A_S$R6584800==3|A_S$R6584900==3|

A_S$R6585000==3|A_S$S0970000==3|A_S$S0970100==3|A_S$S0970200==3|A_S$S0970300=
=3|
           A_S$S0970400==3|A_S$S0970500==3|A_S$S0970600==3]<-1
table(A_S$S2999300)
A_S$JIJF[A_S$S2999300==1]<-1
table(A_S$JIJF)

## Merge Back into Primary Data ####
A_T<-(A_S[, c("R0000100","JIJF")])

H<-merge(G,A_T,by = "R0000100")

# Supplemental Model
M2<-survreg(Surv(Age_DEC, DEC_CEN)~JIJF+RGH97+PIB16+HT97+
```

© 2023 Silver IA et al. *JAMA Network Open.*

```
              HHNW97+Male+Hispanic+White+Black+AIndian+Asian+Sample, robust =
T, data=H, dist= "lognormal")

sink("G:/My Drive/Articles/002-Submitted articles/001-R&R (Due
5.11.23)/Results/Juveniles in Juvenile Facilities (only) on Death.txt")
summary(M2)
confint(M2)
exp(M2$coefficients)
exp(confint(M2))
sink()


M2<-survreg(Surv(Age_DEC,
DEC_CEN)~as.factor(Criminal_Group)+NA_B18+NMI_B18+JIJF+RGH97+PIB16+HT97+
              HHNW97+Male+Hispanic+White+Black+AIndian+Asian+Sample, robust =
T, data=H, dist= "lognormal")

sink("G:/My Drive/Articles/002-Submitted articles/001-R&R (Due
5.11.23)/Results/Juveniles in Juvenile Facilities (All Measures) on
Death.txt")
summary(M2)
confint(M2)
exp(M2$coefficients)
exp(confint(M2))
sink()
```

© 2023 Silver IA et al. *JAMA Network Open.*
