# Supplementary material for: Incarceration of Youths in an Adult Correctional Facility and Risk of Premature Death
Source: JAMA Netw Open. 2023 Jul 5;6(7):e2321805. doi: 10.1001/jamanetworkopen.2023.21805 (PMC10323704; doi:10.1001/jamanetworkopen.2023.21805)
Supplement: Supplement 2. — Data Sharing Statement [file jamanetwopen-e2321805-s002.pdf]

Data Sharing Statement

Silver. Incarceration of Youths in an Adult Correctional Facility and Risk of Premature Death. *JAMA Netw Open*. Published July 05, 2023. doi:10.1001/jamanetworkopen.2023.21805

Data
Data available: Yes
Data types: Deidentified participant data
How to access data: https://www.nlsinfo.org/investigator/pages/search?s=NLSY97#
When available: beginning date: 01-01-2013, end date: 12-31-2033

Supporting Documents
Document types: Statistical/analytic code
How to access documents: Submitted alongside the manuscript.
When available: With publication

Additional Information
Who can access the data: Anyone
Types of analyses: Every Analysis
Mechanisms of data availability: Without investigator support
Any additional restrictions: None
